# Characterization of a *JAZ7* activation-tagged Arabidopsis mutant with increased susceptibility to the fungal pathogen *Fusarium oxysporum*

**DOI:** 10.1093/jxb/erw040

**Published:** 2016-02-19

**Authors:** Louise F. Thatcher, Volkan Cevik, Murray Grant, Bing Zhai, Jonathan D.G. Jones, John M. Manners, Kemal Kazan

**Affiliations:** ^1^CSIRO Agriculture, Queensland Bioscience Precinct, St. Lucia, Queensland 4067, Australia; ^2^The Sainsbury Laboratory, Norwich Research Park, Norwich NR4 7UH, UK; ^3^College of Life and Environmental Sciences, University of Exeter, UK; ^4^College of Biological Sciences, China Agricultural University, Beijing 100093, China; ^5^The Queensland Alliance for Agriculture & Food Innovation (QAAFI), The University of Queensland, Queensland Bioscience Precinct, Brisbane, Queensland 4072, Australia

**Keywords:** Defense, ERF-associated amphiphilic repressor, fungal pathogen, methyl jasmonate, *Pseudomonas syringae*, TOPLESS, transcriptional repression.

## Abstract

In Arabidopsis, jasmonate (JA)-signaling plays a key role in mediating *Fusarium oxysporum* disease outcome. However, the roles of JASMONATE ZIM-domain (JAZ) proteins that repress JA-signaling have not been characterized in host resistance or susceptibility to this pathogen. Here, we found most *JAZ* genes are induced following *F. oxysporum* challenge, and screening T-DNA insertion lines in Arabidopsis JAZ family members identified a highly disease-susceptible *JAZ7* mutant (*jaz7-1D*). This mutant exhibited constitutive *JAZ7* expression and conferred increased JA-sensitivity, suggesting activation of JA-signaling. Unlike *jaz7* loss-of-function alleles, *jaz7-1D* also had enhanced JA-responsive gene expression, altered development and increased susceptibility to the bacterial pathogen *Pst* DC3000 that also disrupts host JA-responses. We also demonstrate that JAZ7 interacts with transcription factors functioning as activators (MYC3, MYC4) or repressors (JAM1) of JA-signaling and contains a functional EAR repressor motif mediating transcriptional repression via the co-repressor TOPLESS (TPL). We propose through direct TPL recruitment, in wild-type plants JAZ7 functions as a repressor within the JA-response network and that in *jaz7-1D* plants, misregulated ectopic JAZ7 expression hyper-activates JA-signaling in part by disturbing finely-tuned COI1-JAZ-TPL-TF complexes.

## Highlight

A *JAZ7* T-DNA activation mutant confers increased JA-sensitivity, up-regulated defense and JA-mediated gene expression, and increased susceptibility to two pathogens that disrupt host JA-responses, *Fusarium oxysporum* and *Pst* DC3000.

Received 5 November 2015; Revised 13 January 2016; Accepted 20 January 2016

## Introduction

The root-infecting fungal pathogen *Fusarium oxysporum* is responsible for vascular wilt disease in over 100 different plant species, including bananas (*Musa* spp.), cotton (*Gossypium* spp.), grain legumes and horticultural crops such as tomato (*Lycopersicum esculentum*) (Di Pietro *et al.*, 2003; Agrios, 2005; Berrocal-Lobo and Molina, 2008). This pathogen also infects Arabidopsis (*Arabidopsis thaliana*) where the pathogen-host interaction can be readily studied in a model system.

Contrasting roles for jasmonate (JA) signaling and JA-mediated defense in Arabidopsis resistance to *F. oxysporum* have been proposed (Kidd *et al.*, 2009; Thatcher *et al.*, 2009). Firstly, activation of JA-mediated defense responses promotes resistance to this pathogen, most likely due to direct antimicrobial activities. Increased resistance to *F. oxysporum* can be achieved in transgenic plants through the over-expression of JA-responsive defense gene expression (e.g. thionins; *Thi2.1*) (Epple *et al.*, 1997; Chan *et al.*, 2005), or manipulation of transcription factors that activate JA-mediated defenses (e.g. defensins and chitinases; *PDF1.2*, *CHIB*). For example, mutation of MYC2, a key regulator of downstream JA-defense signaling, mutation of LBD20, a MYC2-regulated transcription factor, or overexpression of the *Ethylene Response Factors ERF1* and *AtERF2*, activators of JA-defenses, results in up-regulated expression of a specific subset of JA-dependent defense genes and increased resistance to *F. oxysporum* (Berrocal-Lobo *et al.*, 2002; Anderson *et al.*, 2004; McGrath *et al.*, 2005; Thatcher *et al.*, 2012a). Secondly, non-defensive aspects of JA-signaling such as JA-mediated senescence appear to promote susceptibility to this pathogen (Berrocal-Lobo and Molina 2004; McGrath *et al.*, 2005; Kidd *et al.*, 2009; Thatcher *et al.*, 2009, 2012a). It is proposed that in wild-type plants both defensive and non-defensive aspects of JA-signaling are activated following *F. oxysporum* infection but that non-defensive aspects have greater contribution to disease outcome (Thatcher *et al.*, 2009).

Upstream of the MYC2 and ERF transcription factors in the JA-signaling pathway is the F-box protein CORONATINE INSENSITIVE 1 (COI1), which together with JASMONATE ZIM DOMAIN (JAZ) proteins, perceives the JA-signal and forms part of the Skp1/Cullin/F-box (SCF) E3 ubiquitin ligase complex SCF^COI1^-JAZ (Yan *et al.*, 2009; Sheard *et al.* 2010). JAZ proteins provide the connection between perception of the JA signal in the SCF^COI1^-JAZ receptor complex, and downstream transcriptional regulators such as MYC2. In the absence of JA or under low JA levels, JAZ proteins repress transcriptional activators such as MYC2, MYC3 and MYC4, and/or MYC-like transcriptional repressors such as bHLH003/JA-ASSOCIATED MYC2-LIKE 3 (JAM3), bHLH013/JAM2 and bHLH017/JAM1, thereby interfering with the expression of JA-responsive genes. Upon increased JA levels, the ubiquitin-mediated degradation of JAZ proteins leads to the release of these transcription factors from repression (Chini *et al.*, 2007; Thines *et al.*, 2007; Katsir *et al.*, 2008; Melotto *et al.*, 2008; Fernandez-Calvo *et al.*, 2011; Nakata and Ohme-Takagi, 2013; Nakata *et al.*, 2013; Sasaki-Sekimoto *et al.*, 2013, 2014; Song *et al.*, 2013; Fonseca *et al.*, 2014). Although JAZ proteins characterized to date function as repressors of JA-responses, apart from JAZ5, JAZ6, JAZ7, JAZ8 and the non-conventional JAZ13, most do not contain known repression motifs. They form repressor complexes by recruiting the co-repressor TOPLESS (TPL) and TPL-related proteins. This recruitment is mediated through binding of the JAZ ZIM domain to the adaptor protein NINJA (novel interactor of JAZ), which contains an ERF-associated amphiphilic repressor (EAR) motif to recruit TPL (Kagale *et al.*, 2010; Pauwels *et al.*, 2010; Arabidopsis Interactome Mapping Consortium, 2011; Causier *et al.*, 2012; Shyu *et al.* 2012). For recent reviews and updates on JAZ proteins and JA-signaling, see Kazan and Manners (2012), Wager and Browse (2012), Wasternack and Hause (2013) and Sasaki-Sekimoto *et al.* (2014).

Mutation of COI1 and subsequent lack of JA-induced defenses results in enhanced susceptibility to most fungal necrotrophs (e.g. *Botrytis cinerea, Alternaria brassicicola*, Thomma *et al.*, 1998). Interestingly however, COI1 confers susceptibility to *F. oxysporum* with the *coi1* mutant displaying a near-immune like resistance to this pathogen (Thatcher *et al.*, 2009). *coi1*-mediated resistance to *F. oxysporum* is therefore independent of JA-dependent defense gene expression but correlates with compromised non-defensive aspects of JA-dependent responses such as reduced expression of some senescence and oxidative-stress associated genes. Other mutants with compromised JA-defenses but strong resistance to *F. oxysporum* include *pft1* carrying a mutation in the *MED25* gene encoding a subunit of the RNA polymerase II-interacting MEDIATOR complex (Kidd *et al.*, 2009; Cevik *et al.*, 2012). These results imply *F. oxysporum* hijacks the host JA-signaling pathway to promote disease symptom development.

The key role of JAZ repressors in linking COI1 and downstream transcriptional responses suggests these proteins may also play key roles in mediating disease outcome to *F. oxysporum*. Indeed, *JAZ* expression is induced by other pathogens (*Pseudomonas syringae* pv *tomato*, *Pst*), herbivory and wounding (Chini *et al.*, 2007; Thines *et al.*, 2007; Chung *et al.* 2008; Demianski *et al.*, 2012). Potential redundancy amongst the 13 JAZ family members has, however, hampered the determination of functional roles for individual members. C-terminal truncated versions of some JAZ proteins generated from alternate splicing, or in domain-deletion mutants, results in a reduced capacity to bind COI1 leading to stabilization of the JAZ protein. These mutations confer phenotypes such as reduced JA-sensitivity, compromised resistance to herbivory, and/or increased resistance to *Pst* (Chini *et al.*, 2007; Thines *et al.*, 2007; Yan *et al.*, 2007; Chung *et al.*, 2008; Chung and Howe, 2009). Further, Chung *et al.* (2011) found all JAZs except JAZ1, JAZ7 and JAZ8 contain a conserved intron that if retained, modifies the COI1-binding motif, inhibiting COI1-mediated degradation and producing dominant JAZ repressors. Altered JA-responses from overexpression or removal of JAZ proteins has only been observed for overexpression of *JAZ8* and the recently identified *JAZ13* (both resulting in reduced JA-sensitivity) or T-DNA or RNAi knockdown lines of *jaz1* or *jaz10* (resulting in increased JA-sensitivity and/or increased resistance to the fungal pathogen *Botrytis cinerea*) (Yan *et al.*, 2007; Grunewald *et al.*, 2009; Cerrudo *et al.*, 2012; Demianski *et al.*, 2012; Shyu *et al.*, 2012; Leone *et al.*, 2014; Thireault *et al.*, 2015).

In this report, we examined the roles of JAZ family members during the Arabidopsis-*F. oxysporum* interaction through the characterization of *JAZ* gene expression, and the analysis of Arabidopsis *JAZ* T-DNA insertion lines. We identified a unique *JAZ7* allele that confers increased susceptibility to *F. oxysporum* and *Pst*. Interestingly, additional work revealed the T-DNA inserted into the *JAZ7* promoter in this mutant caused constitutive *JAZ7* expression (*jaz7-1D*), conferring activation of JA-signaling and increased JA-sensitivity. However, we demonstrate JAZ7 contains a functional EAR repressor motif, recruiting the co-repressor TPL and repressing transcriptional activation. Further, JAZ7 interacted with both transcriptional activators and repressors of JA-signaling. Based on these results, we propose the misregulated JAZ7 expression in *jaz7-1D* plants resulting from the *JAZ7* T-DNA promoter insertion activates JA-signaling conferring increased JA-sensitivity through recruitment of TPL to specific transcriptional regulators, and disturbing the function of proteins acting within the multi-protein COI1-JAZ-TPL-TF complex.

## Materials and methods

### Plant material and growth conditions

Unless otherwise specified, all experiments were conducted with the *A. thaliana* Columbia-0 (Col-0) accession grown under a short daylight regime (8h light:16h dark) at 21ºC as described previously (Thatcher *et al.*, 2009). The T-DNA insertion mutants (Alonso *et al.*, 2003; Woody *et al.*, 2007) *coi1* (SALK_035548), *jaz7-1D* (SALK_040835), *jaz7-1* (WiscDsLox7H11) and other *jaz* insertion lines (Supplementary Table S1 available at *JXB* online) were obtained from the Arabidopsis Biological Resource Centre (ABRC) or the Nottingham Arabidopsis Stock Centre (NASC). T-DNA mutants were confirmed for correct loci insert and homozygous state. Backcrossed, double or triple *jaz* insertion lines were all confirmed by PCR. For generation of *JAZ7* overexpression (*35S:JAZ7*) lines, the *JAZ7* CDS was amplified using JAZ7-*HindIII*-F and JAZ7-*EcoRI*-R, cloned into pKEN (McGrath *et al.*, 2005), mobilized into *Agrobacterium* AGL1 and transformed into Col-0 by floral dipping. Transgenic plants were selected on 50mg l^−1^ Pestanal (glufosinate-ammonium) (Riedel-de Haen, Seelze, Germany) and resulting T3 lines used in subsequent experiments. For generation of *35S:CUC1-JAZ7* ^*EAR*^ plants the *JAZ7* ^*EAR*^ domain with an added stop codon was first cloned into pKEN (McGrath *et al.*, 2005) by annealing and fill-in of the two primers JAZ7-EAR-*HIII-Sma*-F and JAZ7_EAR_STOP-*EcoR*I-R to produce *35S:JAZ7* ^*EAR*^ pKEN. The length of the *JAZ7* ^*EAR*^ domain was based on the SRDX^*EAR*^ sequence (Hiratsu *et al.*, 2003). The *CUC1* CDS was amplified using CUC1-*HIII*-F and CUC1-STOPdel-*Sma*-R, which has the *CUC1* stop codon removed, cloned into *35S:JAZ7* ^*EAR*^ pKEN to make *35S:CUC1-JAZ7* ^*EAR*^ pKEN, mobilized into *Agrobacterium* AGL1, and transformed into Col-0. Primers for the generation of transgenic plants are listed in Supplementary Table S2.

### Pathogen assays

Root-dip inoculations on 3–4-week-old plants with a 1×10^6^ cell ml^−1^ spore suspension of *F. oxysporum* strain Fo5176 (Thatcher *et al.*, 2012*b*) were performed as described previously (Thatcher *et al.*, 2009). *Pseudomonas syringae* assays were performed with the strain *P. syringae* pv. *tomato* DC3000 (*Pst* DC3000) and syringe infiltrated into leaves at 5×10^6^ cells ml^−1^. Infiltrated plants were incubated at 28°C (16h light/8h dark) under a clear plastic dome and bacterial growth quantified as previously described (Gleason *et al.*, 2011). *Alternaria brassicicola* assays were performed as described in Gleason *et al.* (2011) using 5×10^6^ spores ml^−1^ of the isolate UQ4273.

### *F. oxysporum* culture filtrate assay

*F. oxysporum* culture filtrate assays were performed as per Thatcher *et al.* (2012a) on 15 leaves per line.

### Flowering time

Flowering time experiments were performed according to Kidd *et al.* (2009) under short-day conditions (8h light/16h dark).

### MeJA root elongation inhibition assays

Seeds of wild-type, *jaz7-1D, jaz7-1* or *JAZ7-OX* lines were surface sterilized and plated onto MS media in either the presence or absence of 50 µM MeJA. Root length was measured on 7-day-old seedlings using ImageJ (Schneider *et al.*, 2012).

### Quantitative RT-PCR

Quantitative-RT-PCR (qRT-PCR) experiments were performed on tissue collected after control, *F. oxysporum* (see ‘Pathogen assays’) or MeJA treatment (see ‘Microarray analysis’). Three biological replicates were taken for all experiments comprising tissue pooled from 5–30 plants. RNA extraction, cDNA synthesis and Q-RT-PCR were conducted as described by McGrath *et al.* (2005) using an Applied Biosystems 7900HT Fast Real-Time PCR System (Foster City, CA) or by Thatcher *et al.* (2015) using a CFX384 (Bio-Rad) system. Absolute gene expression levels relative to the previously validated reference genes *β-actin 2*, *β-actin 7* and *β-actin 8* (At1g49240, At3g18780 and At5g09810, respectively) were used for each cDNA sample using the equation: relative ratio gene of interest/actin=(Egene^-Ct gene^)/(Eactin^-Ct actin^) where Ct is the cycle threshold value. The gene specific primer sequences are listed in Supplementary Table S3.

### Microarray analysis

Four independent biological replicates each consisting of shoot material from 20 wild-type and *jaz7-1D* plants were harvested 6h after mock or MeJA treatments. Treatment involved enclosing trays of 4-week-old soil-grown plants under clear plastic covers with a treated cotton ball attached to the inside of the cover, either 1ml of mock solution (100% ethanol) or 1ml of 5% MeJA dissolved in 100% ethanol, and sealing each tray in two layers of opaque plastic bags. Total RNA was extracted (RNeasy Plant Mini Kit, Qiagen), then labeled, hybridized, washed and scanned by the Australian Genome Research Facility (AGRF) (Melbourne, Australia) onto 16 ATH1 GeneChip arrays and the resulting data analyzed using GenespringGX 7.3.1 (Agilent) as previously described (Dombrecht *et al.*, 2007). Briefly, the raw CEL files were normalized using the RMA algorithm, and then the resulting expression values were normalized per chip to the median across all chips. The microarray data was also analyzed using a two-way analysis of variance (ANOVA; *P*<0.05) on the entire dataset with the inclusion of the Benjamini and Hochberg false discovery rate (FDR) (microarray data is deposited under accession number GSE61884 at the NCBI Gene Expression Omnibus). Gene Ontology (GO) term enrichment analysis was performed using agriGO v1.2 (Du *et al.*, 2010) using the default FDR (*P*≤0.05) determined *P*-value significance. Functional annotations of genes and AGI symbols were sourced from TAIR9 datasets.

### Y2H assays

For Y2H experiments, *JAZ7, JAZ5, JAZ8, MYC2, MYC3, MYC4, TPL* and *JAM1* were PCR-amplified from Arabidopsis cDNA (accession Col-0) using the primers in Supplementary Table S2 followed by second amplification with pAttB1 and pAttB2, and cloned into the pDONRZeo plasmid (Invitrogen). The JAZ7mEAR motif was generated by mutating the conserved leucine residues of the EAR motif to alanine using the QuickChange II Site Directed Mutagenesis Kit (Agilent Technologies) following the manufacturer’s recommendations. The entry clones were then recombined with the Gateway-compatible yeast two-hybrid (Y2H) vectors derived from pGADT7 and pGBKT7 (Clontech). Y2H assays were performed using Clontech’s Matchmaker system with strain AH109.

### Co-IP assays

*JAZ7, JAZ7mEAR, JAZ5 and JAZ8* were constructed as described for Y2-H assays except JAZ7-R2, JAZ8-R2 or JAZ5-R2 (Supplementary Table S2) were used as reverse primers in order to remove stop codons and cloned into pDONRZeo plasmid (Invitrogen). The entry clones were recombined with the Gateway-compatible pEarleyGate 101 (with 35S promoter and C-terminal YFP fusion tag), and GFP cloned into pEarleyGate100 used as control (Earley *et al.*, 2006). TPL was amplified from Col-0 gDNA and cloned into pICH47742 (with 35S-promoter and C-terminal 4×Myc fusion tag) using the Golden Gate assembly method (Engler *et al.*, 2008). Five-week-old *N. benthamiana* plants were used for *Agrobacterium tumefaciens*-mediated transient expression of indicated constructs as described previously (Cevik and Kazan, 2013). Co-immunoprecipitation experiments were carried out as described previously (Sohn *et al.*, 2012). Leaf samples were harvested 2 d post-inoculation, and total protein extracts were incubated with 20 μl GFP-affinity matrix (Chromotek) for immunoprecipitation. HRP-conjugated anti-GFP antibody (Santa Cruz) and anti-c-Myc antibody (Santa Cruz) were used for immunoblot analyses.

### Transcriptional activation assays

Full length *MYC3* and *MYC4* were used to construct effector plasmids by fusing with the yeast *GAL4* DNA-binding domain (*GAL4DB*)-coding region under the control of the CaMV35S promoter into the *pGAL4DBGW* vector (Cevik *et al.*, 2012). Full length *JAZ7*, *JAZ7mEAR* and *JAZ8* were cloned into the Gateway-compatible *pJIT60* vector. *pGAL4UAS:GUS* was used as the reporter plasmid. The plasmid *p2X35S:fLUC* was used as a control to normalize reporter gene activity. Transcription activation assays by particle bombardment into rosette leaves of 5–6-week-old Col-0 plants were carried out as described by Cevik *et al.* (2012). Luminescence and fluorescence were determined with a Varioskan Flash multiplate reader (Thermo Scientific).

## Results

### *JAZ* genes are differentially expressed both locally and systemically in response to *F. oxysporum*

**Fig. 1.**
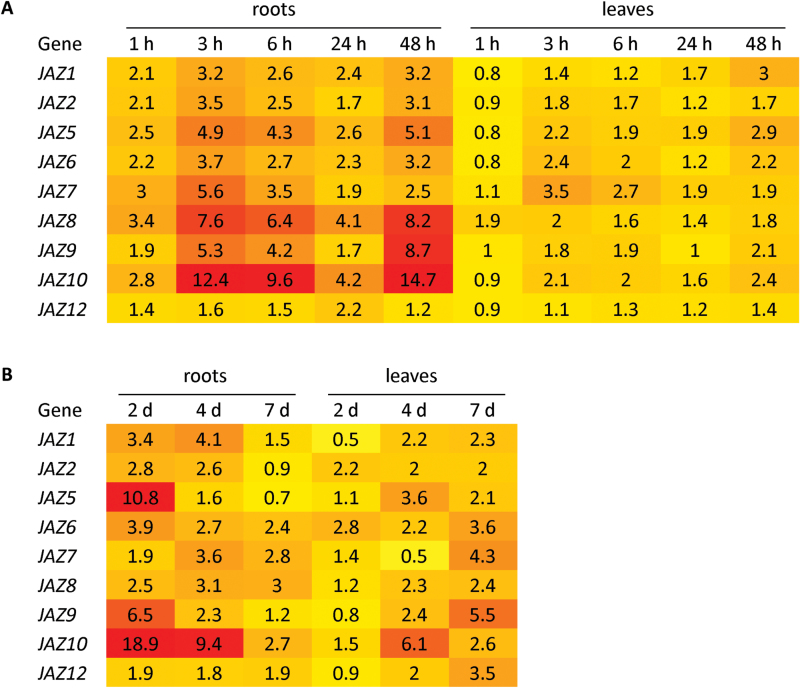
Differential *JAZ* gene expression is induced after *F. oxysporum* inoculation. Heat map of *JAZ* gene expression in roots or leaves of *F. oxysporum* inoculated wild-type plants over (A) a 1–48h or (B) 2–7 d time-course. Expression is relative to control treatment. *JAZ3*, *JAZ4* and *JAZ11* expression did not differ between inoculation or control treatments and are not shown. Values were determined by quantitative RT-PCR from three biological replicates consisting of pools of 10–20 plants.

To examine the role of *JAZ* genes in the Arabidopsis-*F. oxysporum* interaction, we first determined if their expression was responsive to pathogen infection. As *F. oxysporum* infects through the roots and spreads upwards into leaf tissues where the disease symptoms manifest, we examined root and leaf tissues separately, and initially sampled 1–48h post-inoculation (Fig. 1A) as *JAZ* expression can be rapidly induced by JA signals. Most *JAZ* genes exhibited higher inductions over control treatments in roots compared to leaves, where expression peaked at 3h post-inoculation, then rose again at 48h post-inoculation. The largest inductions of 5- to 15-fold were observed for *JAZ5, JAZ7, JAZ8, JAZ9* and *JAZ10*. The expression of *JAZ3*, *JAZ4* and *JAZ11* did not differ from those observed in control treated samples (data not shown). These results suggest some JAZ members may have roles in mediating responses to early stages of *F. oxysporum* infection, particularly in root tissues.

As *F. oxysporum* disease symptoms become evident in the leaves of wild-type Col-0 plants at 7–10 d post-inoculation, we were also interested to determine the persistence of *JAZ* expression at later time-points. In this later time-course (2, 4 and 7 d post-inoculation), fold-inductions were again typically higher in root tissues with *JAZ6*, *JAZ7, JAZ9, JAZ10* and *JAZ12* also highly induced in leaf tissues at 7 d post-inoculation (Fig. 1B). As with the earlier time-course, expression of *JAZ3*, *JAZ4* or *JAZ11* was non-responsive (data not shown). In summary, the differential expression patterns of multiple *JAZ* genes in response to *F. oxysporum* suggests that at least some members may have roles in modulating host responses to this pathogen.

### Screening of *JAZ* T-DNA insertion lines identifies a *JAZ7* line with increased susceptibility to *F. oxysporum*

**Fig. 2.**
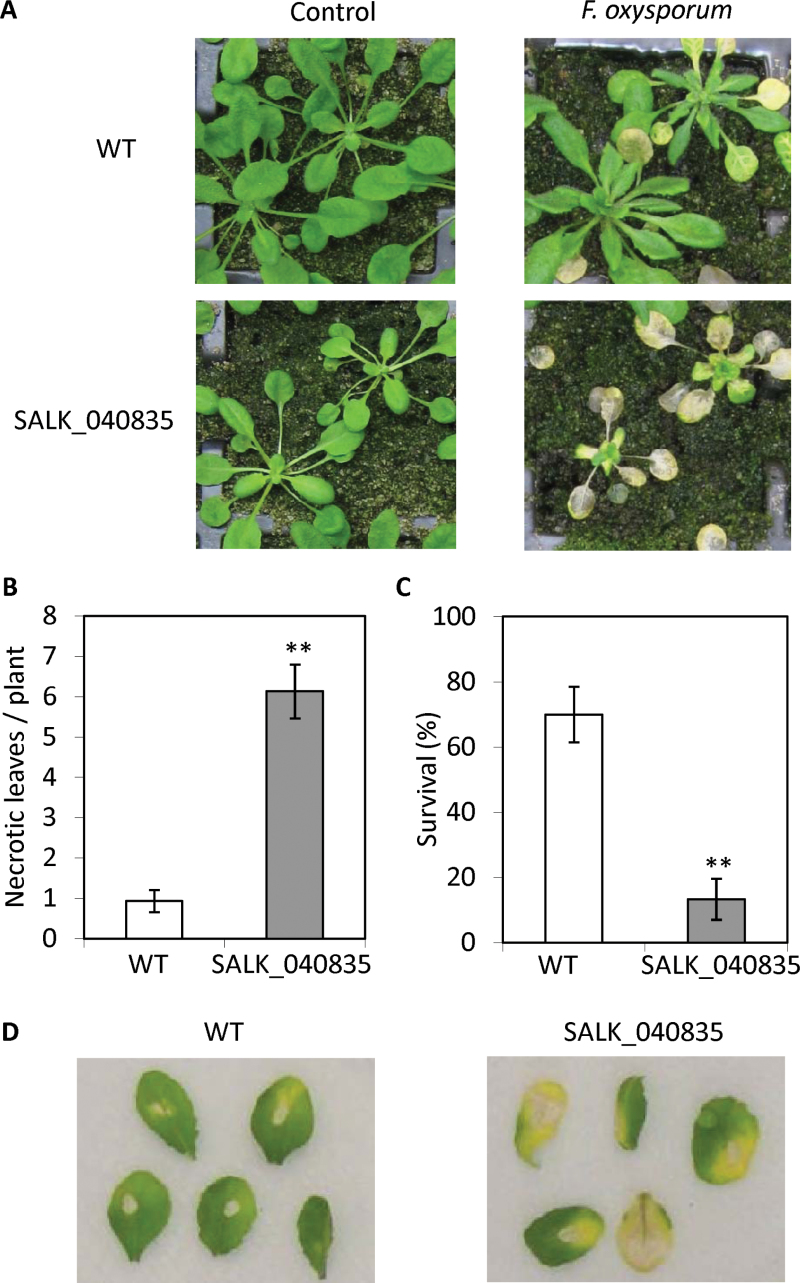
SALK_040835 is highly susceptible to *F. oxysporum*. Wild-type (WT) and SALK_040835 were inoculated with *F. oxysporum* and disease symptoms monitored over 21 d. (A) Representative images of WT and SALK_040835 plants 10 dpi or control treatment. (B) Necrotic leaves per plant at 10 d and (C) survival rates at 21 d post-inoculation. Values are averages ±SE (*n*=30). Asterisks indicate values that are significantly different (**, *P*<0.01; Student’s *t*-test) from WT. Similar results were obtained in independent experiments. (D) *F. oxysporum* culture filtrate was applied to detached WT and SALK_040835 leaves. Representative leaves are shown from three replicates 6 d post-treatment. Control treatments of potato dextrose broth (PDB) and H_2_O showed no phenotype (not shown). Similar results were obtained in an independent experiment.

To investigate possible roles of JAZ proteins in *F. oxysporum* resistance, we screened publically available *JAZ* T-DNA insertion lines with *F. oxysporum*. With the exception of *JAZ1*, *JAZ3*, *JAZ6, JAZ7, JAZ8* and *JAZ11*, the remaining T-DNA insertions are located within the transcribed regions of the *JAZ* genes (Supplementary Fig. S1; de Torres Zabala *et al.*, 2015). Of the insertion lines screened, one line (SALK_040835) with a T-DNA insertion in the promoter of the *JAZ7* gene (351bp upstream of transcription start site) displayed significantly (*P*<0.01, Student’s *t*-test) higher susceptibility to *F. oxysporum* than wild-type in three independent inoculation experiments (Fig. 2). As shown in Fig. 2A–C, both disease symptom development and plant death in inoculated SALK_040835 plants were significantly higher than those of wild-type plants. The SALK_040835 plants also had a smaller rosette size (Fig. 2A). No significantly altered resistance to *F. oxysporum* was evident in the remaining T-DNA insertion lines tested (Supplementary Fig. S2).

The culture filtrate of *F. oxysporum* is known to trigger a leaf-chlorosis phenotype that is closely correlated with the *F. oxysporum* resistance/susceptibility phenotypes (Thatcher *et al.*, 2009, 2012*a*). We found culture filtrate-treated leaves from SALK_040835 developed an earlier and stronger senescence and chlorosis phenotype than leaves from wild-type plants (Fig. 2D). These results suggest JAZ7 may be involved in regulating plant defense responses to *F. oxysporum*.

### The SALK_040835 line shows elevated *JAZ7* expression

**Fig. 3.**
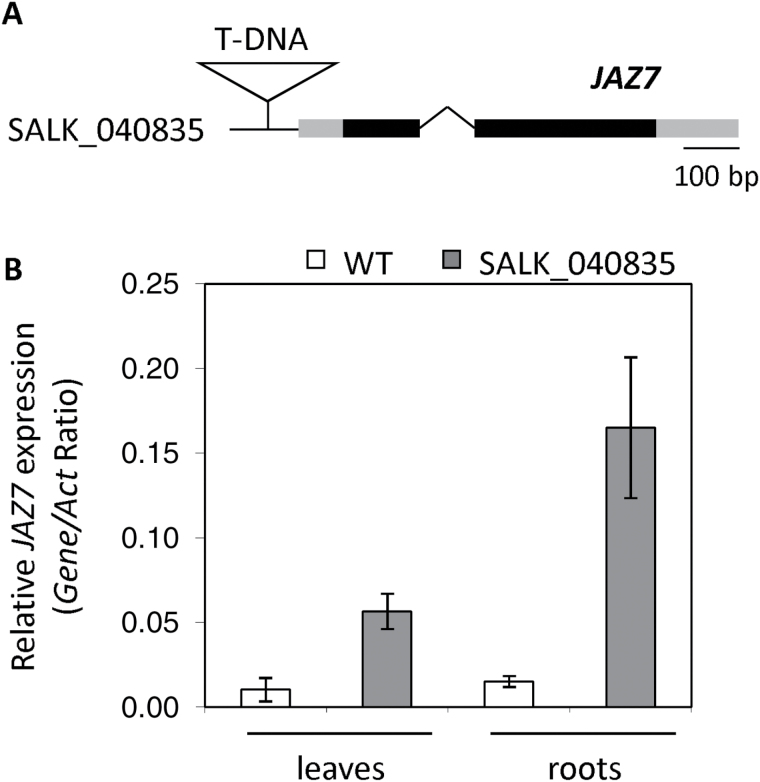
SALK_040835 shows elevated *JAZ7* expression. (A) Schematic representation of the SALK_040835 T-DNA insertion line. The insertion (open triangle) lies upstream of the *JAZ7* transcription start site. 5′ and 3′ UTR are shaded in gray, exons in black and the only intron as a removed segment. (B) *JAZ7* expression was examined in the leaves and roots of wild-type (WT) and SALK_040835 plants. Values are averages ±SE of three biological replicates comprising 5–10 plants. Gene expression levels are relative to the internal control *β-actin* genes.

To determine how the T-DNA inserted into the promoter of *JAZ7* (Fig. 3A) in SALK_040835 affects *JAZ7* expression, we examined *JAZ7* transcript levels in SALK_040835 and wild-type plants. Basal *JAZ7* expression in the roots and leaves of SALK_040835 was 10.8- and 5.4-fold higher, respectively, than those of wild-type plants (Fig. 3B). This suggests SALK_040835 contains an activation-tagged *JAZ7* allele. We therefore designated SALK_040835 as *jaz7-1D.* From the screening of over 30 plants, we were unable to isolate homozygous SALK_040835 lines suggesting *jaz7-1D* acts dominantly and that homozygous lines of this insertion mutant may be lethal, the latter of which we confirmed via detection of seed aborts in *jaz7-1D* siliques (Supplementary Fig. S3A). Independently, Yan *et al.* (2014) also recently reported SALK_040835C as a *JAZ7* activation mutant and with small stature. Progeny from two other separately isolated SALK_040835 lines also showed small rosette size and increased susceptibility to *F. oxysporum*.

Recent re-sequencing of SALK T-DNA insertion lines (O’Malley *et al.*, 2014, unpublished) suggests SALK_040835 may contain other insertions, and this raises the possibility that these additional insertions, if confirmed, may contribute to the *jaz7-1D* phenotypes. One insertion is proposed to be located within the promoter of At2g47780 (rubber elongation factor protein), one in the coding sequence of At2g47790 (*GIGANTUS*), and the others in intergenic regions. We therefore screened SALK_040835/*jaz7-1D* plants by PCR for insertions in At2g47780 and At2g47790 but were unable to identify any insertion in At2g47790, while all plants were heterozygous for the At2g47780 insertion. We also examined the Col-0 and SALK_040835C RNA sequencing data of Yan *et al.* (2014) to compare transcript levels of *At2g47780* and *At2g47790,* and genes flanking the possible intergenic T-DNA insertions, but found no differential levels or truncated transcripts. Together, these results support the conclusion that the phenotypes observed in *jaz7-1D* are related to the *JAZ7* promoter insertion.

### A null mutation in *JAZ7* does not affect resistance to *F. oxysporum*

**Fig. 4.**
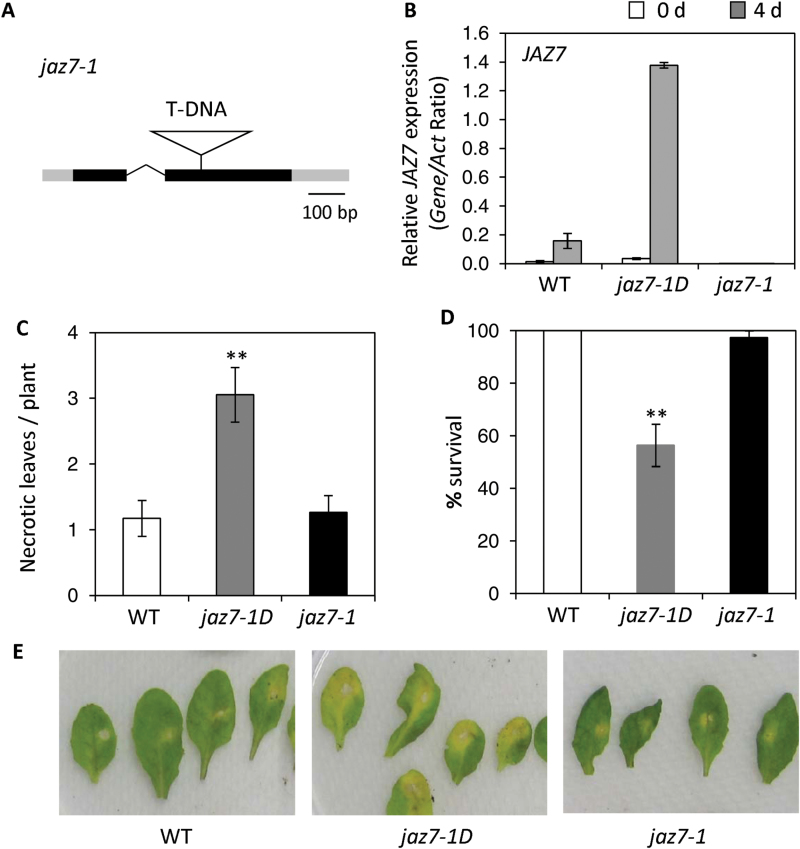
A null T-DNA insertional inactivation line of *JAZ7* does not affect resistance to *F. oxysporum*. (A) Schematic representation of the *jaz7-1* (WiscDsLox7H11) T-DNA insertion line. 5′ and 3′ UTR are shaded in gray, exons in black and the only intron as a removed segment. (B) *JAZ7* expression was examined in leaves of wild-type (WT), *jaz7-1*D and *jaz7-1* plants before or 4 d after *F. oxysporum* inoculation. Values are averages ±SE of three biological replicates comprising 5–10 plants. Gene expression levels are relative to the internal control *β-actin* genes. (C-D) WT, *jaz7-1D* and *jaz7-1* were inoculated with *F. oxysporum* and disease symptoms recorded with (C) necrotic leaves per plant at 10 d and (D) survival rates at 14 d post-inoculation. Values are averages ±SE (*n*=60). Asterisks indicate values that are significantly different (**, *P*<0.01; Student’s *t*-test) from WT. Similar results were obtained in independent experiments. (E) *F. oxysporum* culture filtrate was applied to detached WT, *jaz7-1*D and *jaz7-1* leaves. Representative leaves are shown from three replicates 6 d post-treatment.

The finding that *jaz7-1D* contains an activation-tagged *JAZ7* allele indicates the possibility that the increased expression of *JAZ7* might be responsible for increased susceptibility to *F. oxysporum* in this line. To determine whether plants with null mutations in the *JAZ7* gene could show an opposite *F. oxysporum* resistance phenotype, we isolated a homozygous *jaz7* mutant (WiscDsLox7H11) designated as *jaz7-1*, where the T-DNA is inserted into the second exon of the *JAZ7* gene (Fig. 4A). No detectable transcripts from the truncated *jaz7-1* locus could be identified before or after inoculations with *F. oxysporum* in the *jaz7-1* mutant (Fig. 4B, Supplementary Fig. S3). In contrast, *JAZ7* transcript levels were hypersensitive to induction by *F. oxysporum* in the activation tagged *jaz7-1D* mutant (Fig. 4B). Compared to wild-type plants, *jaz7-1* did not exhibit altered resistance to *F. oxysporum* in either disease or culture filtrate assays (Fig. 4C–E). The absence of any pathogen-associated phenotype in *jaz7-1* is consistent with the view that null mutations in most *JAZ*-encoding genes do not produce JA-related phenotypes (e.g. Thines *et al.*, 2007) possibly due to the functional redundancy within this gene family. We also screened *jaz7-1* in double and triple *jaz* mutant lines, as well as other combinations of *jaz* mutants in *F. oxysporum* disease assays (Supplementary Table S1; de Torres Zabala *et al.*, 2015). Most of the *JAZ* insertion lines we used have been previously characterized for loss-of-function or reduced transcript expression, and we further confirmed this for *jaz2* (SALK_025279), *jaz5* (SALK_053775) and *jaz10* (SAIL_92_D08). Although further experiments need to be conducted to determine if *JAZ* transcript levels are affected in the remaining *jaz* insertion lines, none of these lines exhibited altered disease phenotypes compared to wild-type plants (data not shown).

**Fig. 5.**
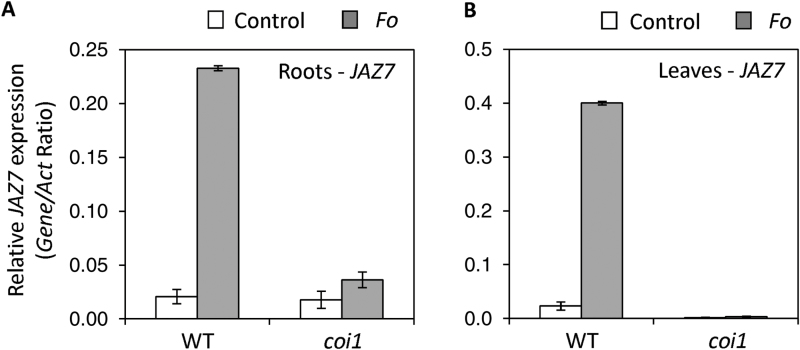
*Fusarium* induced *JAZ7* expression is COI1-dependent. *JAZ7* expression was monitored in (A) roots and (B) leaves of control or *F. oxysporum* (*Fo*)-challenged wild-type (WT) or *coi1* plants at 4 d post-infection. Values are averages ±SE of three biological replicates consisting of pools of 10–30 plants. Gene expression levels are relative to the internal control *β-actin* genes. Similar results were obtained in an independent experiment.

Given that increased *JAZ7* expression in the *jaz7-1D* mutant correlated with increased susceptibility to *F. oxysporum* and JAZ proteins act as repressors in JA-signaling, we asked whether the *Fusarium* inducibility of *JAZ7* requires COI1. As shown in Fig. 5, *F. oxysporum* inducibility of *JAZ7* was abolished in both roots and leaves of the *coi1* mutant, suggesting that COI1 (or JA-sensing) is required for pathogen inducible *JAZ7* expression.

### *jaz7-1D* shows differential resistance to other pathogens and an early flowering phenotype

**Fig. 6.**
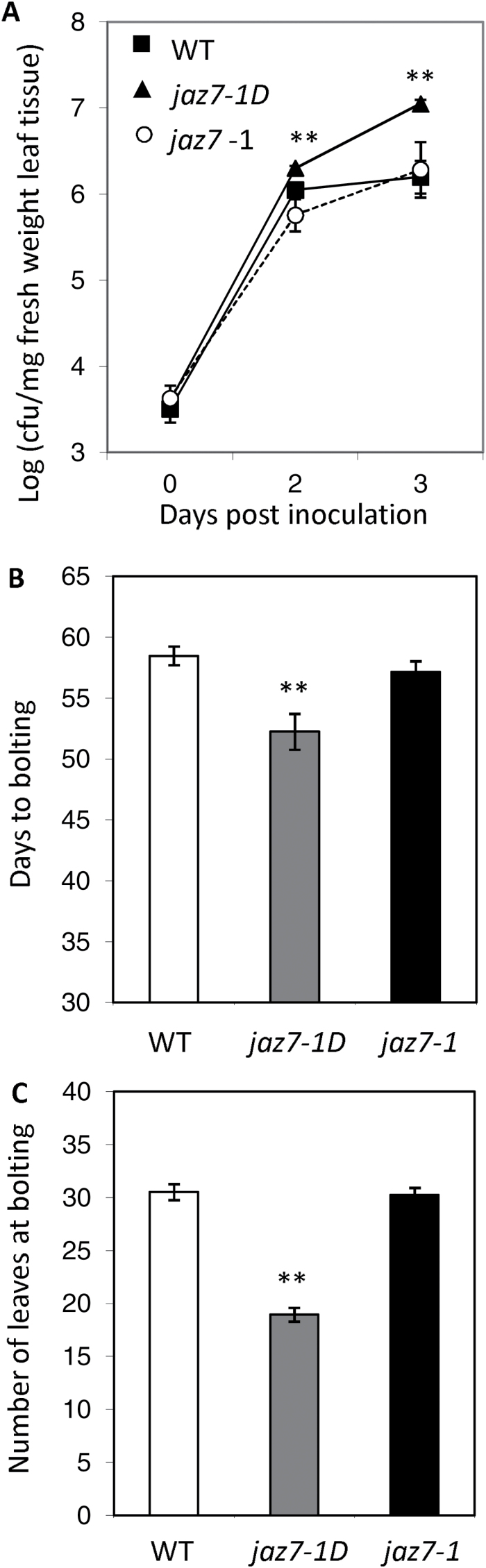
*jaz7-1D* is highly susceptible to *Pseudomonas syringae* pv tomato and exhibits an early flowering phenotype. (A) Pathogen infection of wild-type (WT), *jaz7-1D* and *jaz7-1*. Log *P. syringae* counts from leaf tissue after *Pst* DC3000 infection over 3 d. Values are averages ±SE of four biological replicates consisting of pools of four leaves. Flowering time as noted by (B) days to bolting and (C) number of rosette leaves at bolting. Values are averages ±SE of two biological replicates consisting of pools of 10 plants. Asterisks indicate values that are significantly different (**, *P*<0.01; Student’s *t*-test) from WT. Similar results were obtained in independent experiments.

JA-signaling in Arabidopsis is also known to affect resistance to pathogens other than *F. oxysporum*. For instance, as with *F. oxysporum*, JA-signaling promotes susceptibility to the bacterial pathogen *Pst* DC3000 (Kloek *et al.*, 2001) whereas intact JA-signaling is required for resistance to the leaf-infecting necrotrophic pathogen *Alternaria brassicicola* (Thomma *et al.*, 1998). We therefore tested *jaz7-1D* and *jaz7-1* mutants against both of these pathogens. Similar to its response to *F. oxysporum*, the *jaz7-1D* mutant showed significantly increased susceptibility to *Pst* (Fig. 6A) while, consistent with de Torres *et al.* (2015) no effect of the *jaz7-1* mutation on resistance was evident. In contrast, *jaz7-1D* and *jaz7-1* showed no significant difference in resistance or susceptibility to *A. brassicicola* relative to wild-type plants. Combined, these results implicate JAZ7 in resistance against specific pathogens.

In addition to compromised disease resistance, we noted that the *jaz7-1D* mutant flowered earlier than *jaz7-1* and wild-type plants under short-day conditions (Fig. 6B, C).

### The *jaz7-1D* mutant shows increased JA-sensitivity and JA-responsive gene expression

**Fig. 7.**
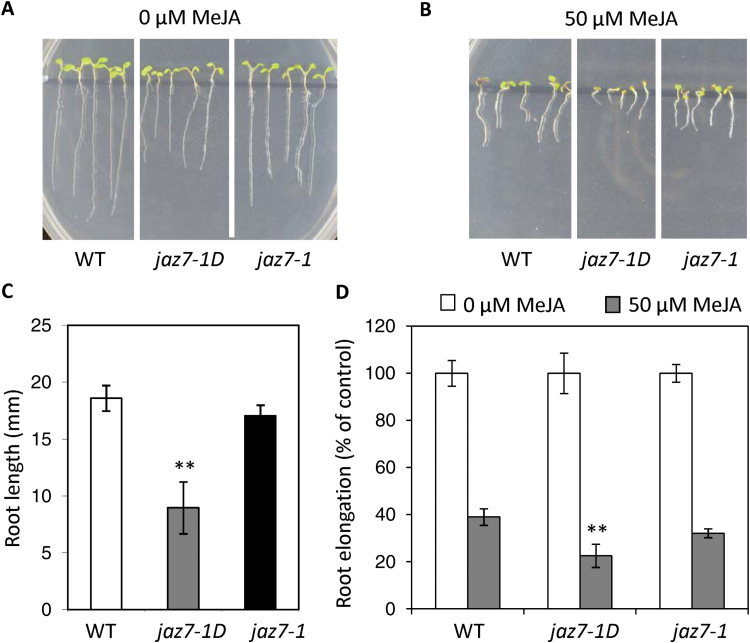
*jaz7-1D* shows increased JA-sensitivity. Sensitivity of wild-type (WT), *jaz7-1D* and *jaz7-1* seedlings to JA was determined by MeJA inhibition of root growth on control media versus media containing MeJA at 7 d post-germination. Representative images of seedlings on (A) control (0 µM MeJA) or (B) MeJA media (50 µM). *jaz7-1D* mutants have shorter roots under basal conditions (C) and their root elongation (D) shows increased sensitivity to MeJA. Root elongation of each line when grown on control media or media containing MeJA was calculated as a percentage relative to control treatment. Values are averages ±SE of three biological replicates consisting of pools of 10–15 seedlings. Values that differed significantly from the WT were identified by the one-way ANOVA and Dunnet’s post-hoc test (**, *P*<0.01). Similar results were obtained in independent experiments.

As JAZ proteins act as repressors of JA signaling, we hypothesized that JA-dependent plant responses such as JA-mediated inhibition of primary root elongation and JA-responsive gene expression may be altered in the *jaz7-1D* mutant due to constitutive *JAZ7* expression. We first tested the JA-mediated root growth inhibition phenotype of *jaz7-1D* and *jaz7-1* mutants. In the absence of MeJA, *jaz7-1D* roots were shorter than those of wild-type and *jaz7-1* (Fig. 7A, C). The percent inhibition of root elongation by MeJA was also greater in *jaz7-1D* than in wild-type and *jaz7-1* (Fig. 7B, D), suggesting activated *JAZ7* expression in the *jaz7-1D* mutant confers increased JA sensitivity rather than the decreased sensitivity expected from a repressor.

**Fig. 8.**
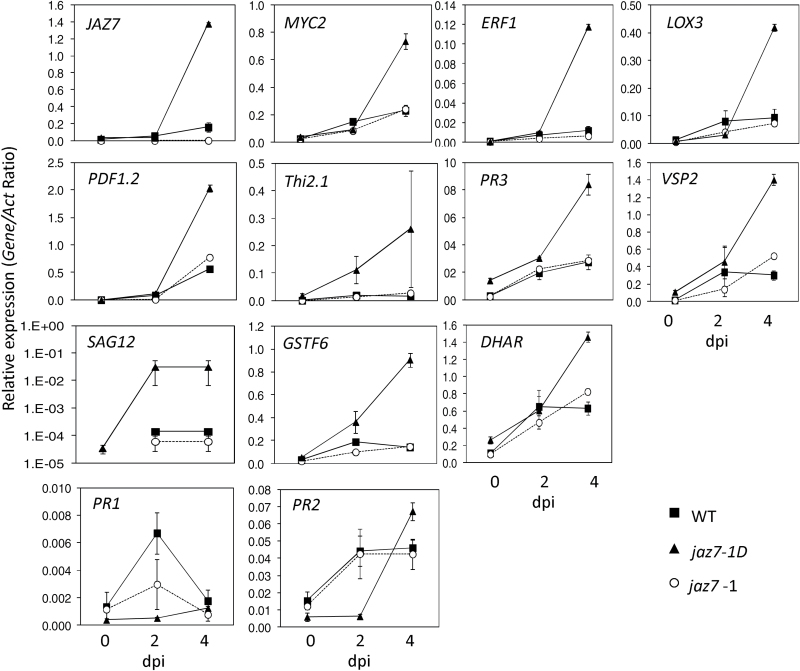
*jaz7-1D* shows increased JA-responsive gene expression under *Fusarium* infection. Gene expression was monitored in leaf tissue of untreated or *F. oxysporum*-challenged wild-type (WT), *jaz7-1D* and *jaz7-1* plants at 2 and 4 d post-infection (dpi). Values are averages ±SE of three biological replicates consisting of pools of five plants. Gene expression levels are relative to the internal control *β-actin* genes.

**Fig. 9.**
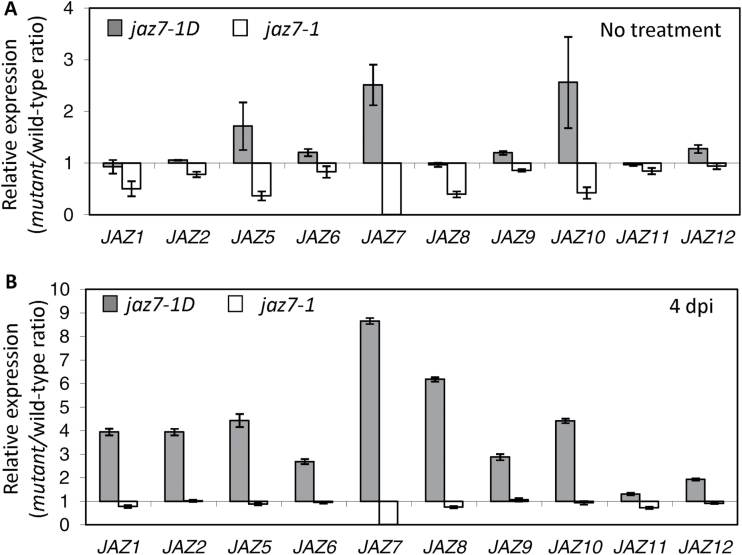
*JAZ* transcripts are up-regulated in *jaz7-1D. JAZ* expression was monitored in leaf tissue of (A) untreated or (B) *F. oxysporum*-challenged wild-type (WT), *jaz7-1D* and *jaz7-1* plants at 4 d post-infection (dpi). Values are expressed relative to WT values at that time-point. Values are averages ±SE of three biological replicates consisting of pools of five plants. Gene expression levels are relative to the internal control *β-actin* genes. *JAZ3* and *JAZ4* expression was not examined due to lack of *F. oxysporum* inducibility (Fig. 1).

We next analyzed the *F. oxysporum*-induced expression of JA-responsive genes in the two *jaz7* mutants after inoculations with *F. oxysporum* (Fig. 8). Genes encoding JA-responsive transcription factors (e.g. *MYC2* and *ERF1*), a JA-biosynthesis enzyme (e.g. *LOX3*) and JA-related defense proteins (e.g. *PDF1.2*, *Thi2.1*, *PR3* and *VSP2*) were induced more strongly in the leaves of inoculated *jaz7-1D* plants than in *jaz7-1* and wild-type plants at 4 dpi. Expression of senescence or oxidative stress associated transcripts (e.g. *SAG12, GSTF6, DHAR*) were also up-regulated in *jaz7-1D*. Furthermore, analysis of *JAZ* gene expression after *F. oxysporum* inoculations revealed that transcript levels of almost all *JAZ* genes were up-regulated in *jaz7-1D* while in *jaz7-1* levels were either reduced or did not differ from wild-type levels (Fig. 9). Overall, this indicates JA-regulated gene expression is up-regulated in *jaz7-1D* plants.

In parallel to the overall increases observed in JA-responsive gene expression, the SA marker genes *PR1* and *PR2* showed reduced or delayed induction in response to *F. oxysporum* inoculations (Fig. 8). These gene expression studies together with JA root inhibition data suggest that *jaz7-1D* plants exhibit altered regulation of the JA-pathway in response to *F. oxysporum* infection of Arabidopsis.

### Genome-wide identification of differentially expressed genes in *jaz7-1D*

To further dissect the effect of the *jaz7-1D* mutant on JA-responsive gene expression, we conducted genome-wide identification of genes differentially regulated in the *jaz7-1D* mutant following a control or MeJA treatment. This involved microarray analysis of *jaz7-1D* and wild-type plants from four independent replicates using the Arabidopsis Affymetrix ATH1 Genome Array. Stringent analysis of the expression data was performed using two-way ANOVA (*P*<0.05) on the entire dataset with the inclusion of the Benjamini and Hochberg FDR. A comparison of differentially regulated genes by genotype identified 113 up-regulated and 25 down-regulated genes showing ≥2-fold in mock-treated *jaz7-1D* relative to mock-treated wild-type plants (Supplementary Tables S4–6). To gain insight into the functions of these genes, we performed GO term enrichment analysis. Significantly enriched biological processes from genes up-regulated in *jaz7-1D* were those involved in defense responses, multi-organism processes, and responses to stress, fungi, other organisms, biotic and abiotic stimulus, and organic substances, while those from the down-regulated dataset were enriched for genes involved in response secondary metabolic processes and response to stimulus (FDR<0.05). The majority of genes up-regulated in *jaz7-1D* are also associated with JA-signaling, plant defense and/or senescence including *Thi2.1* which we previously identified as being up-regulated (Fig. 8). For example, three of the up-regulated genes in *jaz7-1D*, encoding a protease 1-like protein (AT2G38860), an alpha-beta hydrolyase superfamily/lipase protein (AT2G42690) and a cyclic nucleotide-regulated ion channel (AT2G46450), respectively, have been identified as senescence markers in Arabidopsis (Yoshida *et al.*, 2001; Gepstein *et al.*, 2003). Notably, the most highly up-regulated gene in *jaz7-1D* encoding a N-acetyltransferase (AT2G39030/NATA1) is highly JA-inducible and linked to *Pst* susceptibility (Adio *et al.*, 2011). In addition, expression of genes (e.g. *DET2/DWF6*) known to promote flowering (Chory *et al.*, 1991; Li *et al.*, 2010) are up-regulated while those (e.g. MADS Box type II protein) associated with negative regulation of flowering (Ratcliffe *et al.*, 2003) are down-regulated in the mutant, thus correlating with the *jaz7-1D* early flowering phenotype. Previously, the *det2* mutant was shown to display a lack of leaf senescence and also delayed flowering time (Chory *et al.*, 1991). The increased *DET2/DWF6* expression in the *jaz7-1D* mutant is therefore consistent with the role of this gene as a positive regulator of senescence and flowering time. Importantly, two genes encoding pectin methylesterase inhibitors were down-regulated in the *jaz7-1D* mutant (Supplementary Table S6). Again, this is consistent with the increased susceptibility phenotype of the *jaz7-1D* mutant as overexpression of methylesterase inhibitors in Arabidopsis provides increased pathogen resistance (Lionetti *et al.*, 2007).

**Fig. 10.**
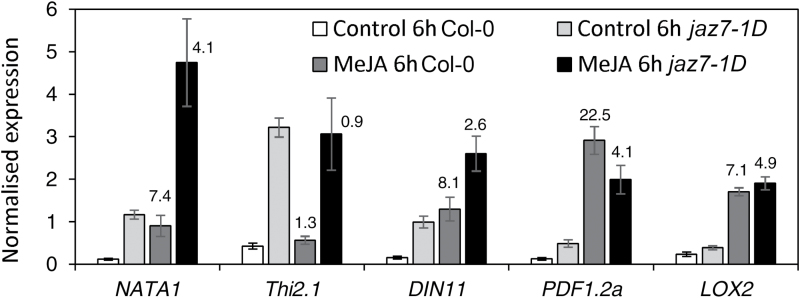
Priming of JA-regulated gene expression in *jaz7-1D.* Highly MeJA inducible genes in wild-type were generally not as inducible in *jaz7-1D.* Shown is a subset of differentially regulated genes in the *jaz7-1D* mutant following a control or MeJA (6h) treatment as identified by microarray analysis. Col-0 control and MeJA: white and dark gray boxes, respectively. *jaz7-1D* control and MeJA: light gray and black boxes, respectively. The numbers above MeJA columns represent fold-induction over control treatment. Values are averages ±SE of four biological replicates consisting of pools of 20 plants.

We next analyzed the two-way ANOVA data for genes differentially regulated by MeJA treatment and identified 56 up-regulated and 21 down-regulated ≥2-fold in MeJA-treated *jaz7-1D* relative to MeJA-treated wild-type plants (Supplementary Tables S7–9). Genes in the up-regulated dataset were enriched for lipid biosynthetic and metabolic processes, response to external stimulus, localization and transport, while the down-regulated dataset were enriched for response to stimulus, stress, chemicals and organic substances. *NATA1* was the highest up-regulated gene, as was it under control treatment. However, we noticed the MeJA inducibility of this gene and others in *jaz7-1D* over its control levels was nearly 2-fold less than its inducibility in wild-type plants, suggesting the primed expression of these genes prevents the same level of induction observed in wild-type plants (Fig. 10). To dissect this phenomenon further, we took the ANOVA data and examined MeJA-inducible expression of genes in wild-type and *jaz7-1D* relative to their levels in control samples. Highly MeJA-inducible genes in wild-type were generally not as inducible in *jaz7-1D* (Table 1, Supplementary Table S10). This included genes involved in JA-responses, defense and senescence such as the two defensins *PDF1.2a* (AT5G544420) and *PDF1.2b* (AT2G26020), *LOX2* (*lipoxygenase* 2/AT3G45140), *COR1* (*coronatine-responsive protein*/AT1G19670), a *glucan endo-1,3-beta-glucosidase* (AT4G16260) and *DIN11* (*DARK INDUCIBLE 11/*AT3G49620). Overall, these results suggest the primed JA-response in *jaz7-1D* may limit further JA-mediated fold-induction and/or that JAZ7 may have a role in inhibition of JA-regulated responses.

**Table 1.** Subset of genes differentially regulated by MeJA treatment from the microarray Shown are the top 20 wild-type MeJA/control-induced genes (data obtained from Supplementary Table S10). Colour coding: change in *jaz7-1D* over wild-type (WT) under each analysis; >2-fold, red; >1.5-fold, orange; <2-fold, green; <1.5-fold, lime.

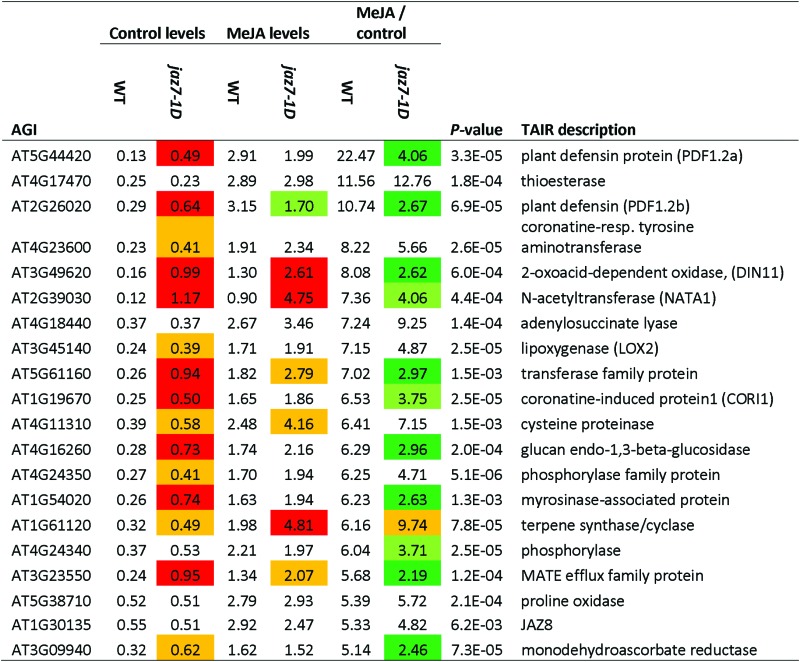

### Transgenic over-expression of *JAZ7* does not reproduce *jaz7-1D* phenotypes

The finding that *JAZ7* and JA-regulated gene expression is up-regulated in the *jaz7-1D* mutant prompted us to generate *JAZ7* overexpression lines. We generated three independent lines overexpressing *JAZ7* under the control of the constitutive *35S* promoter (*JAZ7-OX*) with expression ranging from 9-fold to 1800-fold over wild-type levels (Supplementary Fig. S4A). Interestingly, the *JAZ7-OX* lines did not exhibit the small rosette size or reduced root length phenotypes of *jaz7-1D* under normal growing conditions, but did exhibit, although not significantly, increased basal expression of some but not all JA-marker genes tested (Supplementary Fig. S4B–D). We also examined JA-sensitivity and *Fusarium* susceptibility in the overexpression lines and found only the lowest *JAZ7* expression line *JAZ7-OX1* (with *JAZ7* levels comparable to *jaz7-1D*) displayed increased JA-sensitivity and increased *Fusarium* susceptibility, but only at early stages of infection (Supplementary Fig. S4E–G).

Possibilities for the *JAZ7-OX* lines not phenocopying *jaz7-1D* may be *jaz7-1D* producing altered *JAZ7* transcripts such as those harboring mutations, or formed as a result of altered splicing or altered transcription start sites (TSSs), or the presence of additional undetected T-DNA insertions in *jaz7-1D*. Therefore, we sequenced *JAZ7* transcripts from Col-0, *jaz7-1D* and *JAZ7-OX,* but found no sequence variation. Further, inspection of RNA-seq data from Yan *et al.* (2014), who used SALK_040835C in their studies, revealed no differences in *JAZ7* transcripts (SNPs, truncations, mis-splicing or altered TSSs) compared to wild-type Col-0. Next, to consider the possibility of additional insertions (not collated by SALK) in *jaz7-1D* affecting its phenotypes, we produced a backcrossed (to Col-0) line. The F2 progeny segregated 2:1 heterozygous *jaz7-1D*:Col-0 (confirmed via PCR) as suggestive of a dominant mutation, reiterating our previous results showing that homozygous lines of this insertion mutant may be lethal. The heterozygous progeny also conferred *jaz7-1D* phenotypes of short roots (this study; Yan *et al.*, 2014) and JA-hypersensitivity (Supplementary Fig. S5). If the JA-hypersensitive phenotypes in *jaz7-1D* were due to an additional T-DNA insertion we would expect to see this phenotype segregate, unless the insertion is closely linked. Therefore, combined with our *JAZ7-OX* results, it is possible that *jaz7-1D* JA-related phenotypes are a result of ectopic cell or tissue-specific *JAZ7* expression as a consequence of the T-DNA insertion in the *JAZ7* promoter and/or high levels of *JAZ7* in *jaz7-1D* plants interfering within COI1-JAZ-TPL-TF multiprotein complexes.

### JAZ7 contains a functional EAR-repression motif

**Fig. 11.**
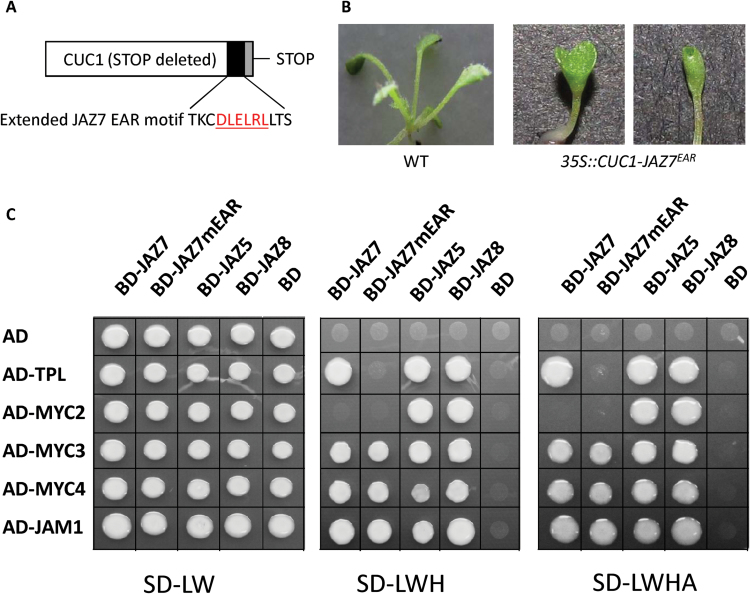
JAZ7 contains an active EAR-repression motif mediating its interaction with TPL, and interacts *in vitro* with MYC3, MYC4 and JAM1. (A) JAZ7 repression domain sequence used to generate a Cup-Shaped Cotyledon (CUC) repression construct. The EAR motif is red underlined. (B) The chimeric protein in which CUC1 was fused to the JAZ7^EAR^ domain was expressed in transgenic plants (*35S:CUC1-JAZ7* ^*EAR*^). Representative wild-type (WT) and *35S:CUC1-JAZ7* ^*EAR*^ seedlings are shown. (C) Y2H assays between Topless (TPL), MYC2, MYC3, MYC4 or JAM1 and JAZ7, a version of JAZ7 containing a mutated EAR-motif (JAZ7mEAR), JAZ5 or JAZ8. Y2Hs were performed on non-selective SD-Leu-Trp media as well as selective SD-Leu-Trp-His or SD-Leu-Trp-His-Ade media. No interactions were seen for the binding domain (BD) only or when an empty activation domain (AD) was used on selective media. JAZ8 and JAZ5 were used as positive controls for TPL interactions. Results were replicated in several independent experiments.

Increased JA-sensitivity and JA-mediated gene expression in *jaz7-1D* suggests a role for JAZ7 in activation of JA responses. However, JAZ proteins repress JA-responses through direct (EAR repressor motif) or indirect (NINJA-mediated) recruitment of co-repressor(s) and the EAR motif associated with gene repression is present in JAZ7 (Kazan, 2006; Kagale *et al.*, 2010). JAZ5, JAZ6, JAZ8 and JAZ13 also contain EAR motifs required for interactions with the co-repressor protein TOPLESS (TPL) (Kagale *et al.*, 2010; Causier *et al.*, 2012; Shyu *et al.* 2012; Thireault *et al.*, 2015) and the transcriptional repression function of this motif in JAZ8 has been confirmed (Shyu *et al.*, 2012). The functionality of the predicted EAR motif found in JAZ7 has, however, not been experimentally tested. To determine whether the JAZ7 EAR motif acts as a functional repressor, we fused an extended version of this motif to the carboxy terminus of the CUC1 (CUP-SHAPED COTYLEDON1) transcription factor which should convert this transcriptional activator to an active repressor and produce plants with a cup-shaped cotyledon phenotype (Hiratsu *et al.*, 2003). As shown in Fig. 11A, B, Arabidopsis plants transformed with *35S:CUC1-JAZ7* ^*EAR*^ showed a typical cup-shaped cotyledon formation, indicating that the *JAZ7* ^*EAR*^ motif can act as a repression domain. Of the 13 selected T1 seedlings, severity of fused cotyledons varied, with eight displaying the cup-shaped cotyledon phenotype.

### JAZ7 interacts with the co-repressor TPL

**Fig. 12.**
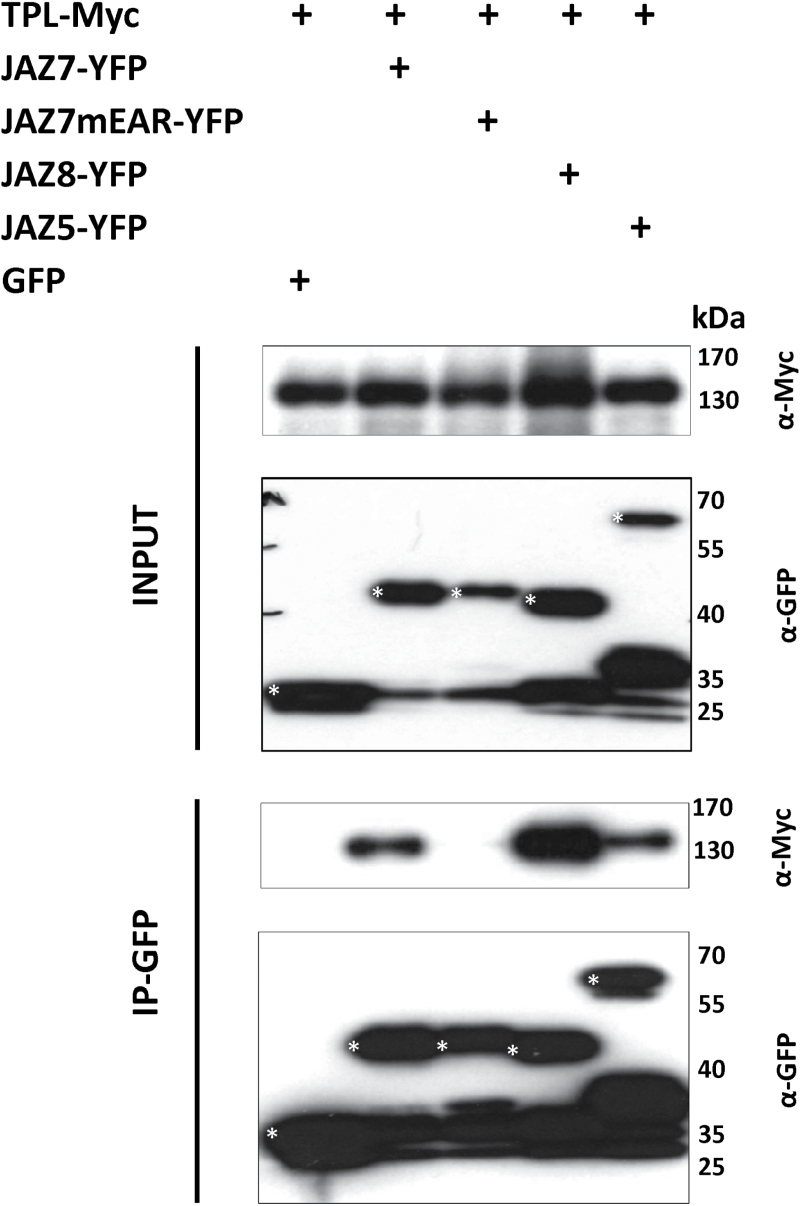
JAZ7 binds TPL *in vivo*. Co-immunoprecipitation analysis from transiently expressed proteins in *N. benthamiana* leaves showing association between TPL and JAZ7, JAZ8 or JAZ5 but not JAZ7mEAR. TPL was fused to C-terminal Myc tag while JAZ7, JAZ5, JAZ8 and JAZ7mEAR are fused to C-terminal YFP tag. Immunoblots show the presence of proteins in total extracts (input, top panels) and after immunoprecipitation with anti-GFP beads (IP-GFP, bottom panels). JAZ8 and JAZ5 were used as positive controls and GFP vector as a background control. Protein molecular mass ladder is shown to the right of each blot. Results were replicated in an independent experiment. Asterisks indicate the expected protein bands.

The finding that ectopic overexpression of the JAZ7 EAR motif has repressor activity prompted us to determine whether JAZ7 could interact with the co-repressor TPL, an interaction which had not yet been demonstrated. Using JAZ5-TPL and JAZ8-TPL interactions as positive controls (Causier *et al.*, 2012; Shyu *et al.*, 2012), an interaction between JAZ7 and TPL in Y2H studies was observed (Fig. 11C). To determine if the interaction is mediated through the EAR motif of JAZ7, we generated a JAZ7 construct containing a mutated version of the EAR motif (JAZ7mEAR). No interaction between JAZ7mEAR and TPL was observed (Fig. 11C). In follow up *in vivo* studies, we conducted co-immunoprecipitation experiments between TPL and JAZ7, JAZ7mEAR, JAZ8 and JAZ5 through transient expression in *Nicotiana benthamiana*. Correlating with the Y2H results, TPL interactions with JAZ7, JAZ5 and JAZ8 were observed, but when the EAR motif of JAZ7 was mutated, the TPL and JAZ7 interaction was strongly diminished (Fig. 12). These results strongly support an *in planta* interaction between TPL and JAZ7 mediated via its EAR-motif.

**Fig. 13.**
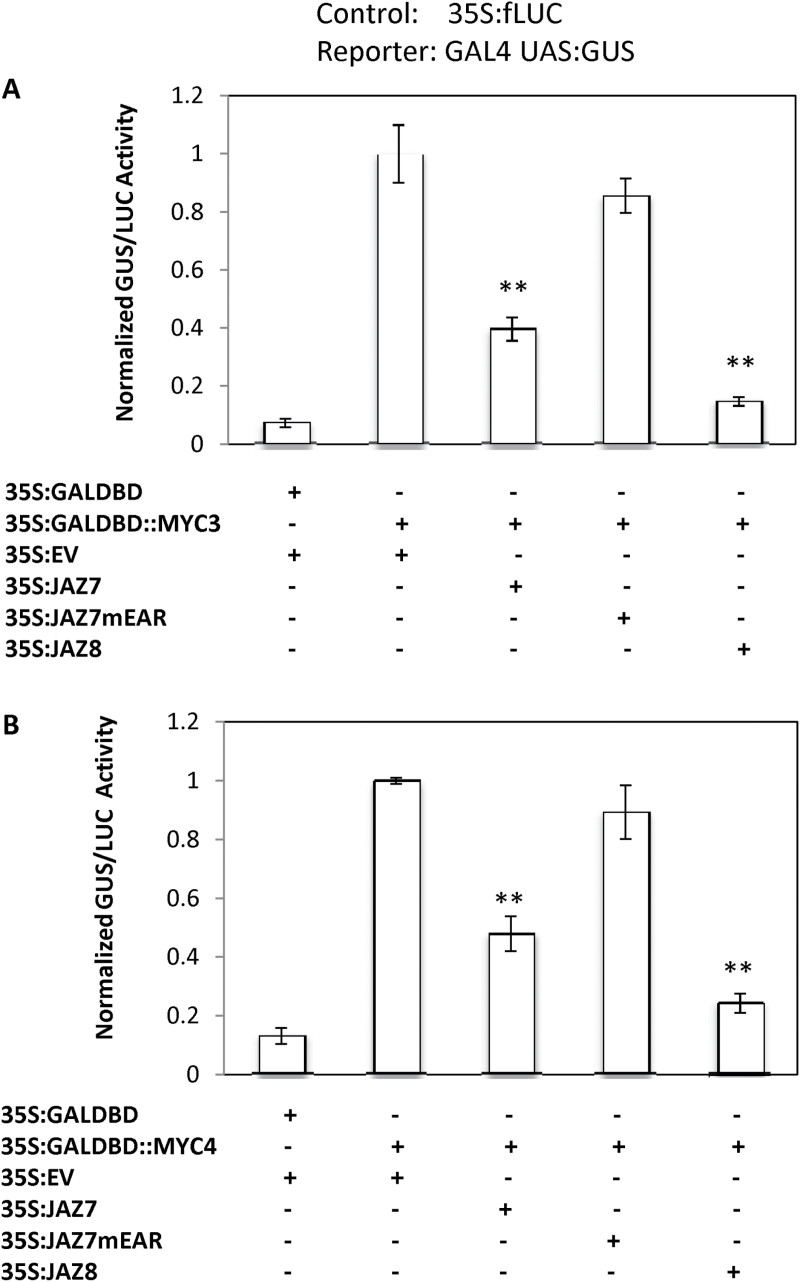
MYC3 and MYC4 transcription activities are repressed by JAZ7 and JAZ8 but not by JAZ7mutEAR in transient activation assays. Transient expression assays in *Arabidopsis thaliana* leaves show that JAZ7 and JAZ8 but not JAZ7mutEAR suppress (A) MYC3- and (B) MYC4-mediated transcription activation using the GAL4 binding domain (DBD) and upstream GAL4-binding sequences (*GAL4-UAS*) fused to the *GUS* gene. The activity of the reporter gene (*GUS*) was normalized to the activity of the firefly *LUC* gene. Data are means (±SD) of three biological replicates of two bombarded leaves. Statistical significance was assessed using the unpaired Student’s *t*-test (**, *P*<0.01). These experiments were carried out twice with similar results.

### JAZ7 interacts with the transcriptional activators MYC3 and MYC4, and the transcriptional repressor JAM1

To dissect the potential mechanism of JAZ7 in JA-responses we tested for JAZ7 interactions with the transcriptional activators MYC2, MYC3 and MYC4 that can bind to most JAZ proteins (Chini *et al.*, 2009; Cheng *et al.*, 2011; Fernandez-Calvo *et al.*, 2011; Niu *et al.*, 2011). Using Y2H approaches, several groups have reported JAZ7 binding to MYC2, MYC3 and MYC4, while others have not detected these interactions (Chini *et al.*, 2009; Arabidopsis Interactome Mapping Consortium, 2011; Cheng *et al.*, 2011; Fernandez-Calvo *et al.*, 2011; Qi *et al.*, 2011). To address this, we conducted Y2H studies using JAZ5 and JAZ8 as positive controls; both interact with MYC2, MYC3 and MYC4 in all published studies to our knowledge (Cheng *et al.*, 2011; Fernandez-Calvo *et al.*, 2011). We found a strong interaction between JAZ7-MYC3 and JAZ7-MYC4, but failed to identify a JAZ7-MYC2 interaction (Fig. 11C).

To determine whether JAZ7 has the capacity to repress these transcriptional activators we conducted transcriptional activation assays with JAZ7 against MYC3 and MYC4. In these experiments, we co-bombarded a reporter gene construct containing the GAL4 upstream activation sequence (*pGAL4UAS*) linked to the *GUS* gene (*pGAL4UAS-GUS*), together with *CaMV35S* expression constructs of *MYC3* or *MYC4* fused to the *GAL4* DNA binding domain (*GAL4BD*) or *GAL4BD* alone, as well as empty vector, *JAZ7*, *JAZ7mEAR* or *JAZ8* under CaMV35S promoter (Fig. 13). In addition, an expression construct of the firefly luciferase (*LUC*) gene was co-bombarded as a normalization control. The addition of the vector constructs expressing either *MYC3*- or *MYC4-GAL4BD* produced significantly higher transcription activity of the GUS reporter gene compared to the control effector plasmid (*GAL4BD* only) when co-bombarded with the empty vector. However, transcription activation abilities of the MYC3 and MYC4-GAL4BD fusion proteins were significantly reduced when co-bombarded with JAZ7 or JAZ8, but not with JAZ7mEAR. These experiments demonstrate JAZ7 and JAZ8, but not JAZ7mEAR, repress MYC3 and MYC4-mediated transcriptional activities.

JAZ7 appears not to interact with the JA-ASSOCIATED MYC2-like transcriptional repressors (JAM) bHLH003/JAM3 or bHLH013/JAM2 (Song *et al.*, 2013; Fonseca *et al.*, 2014; Sasaki-Sekimoto *et al.*, 2014), but one group has reported bHLH017/JAM1-JAZ7 binding (Fonseca *et al.*, 2014). We therefore next tested for JAZ7-JAM1 binding, again using JAZ5 and JAZ8 as positive controls as both interact with JAM1 (Song *et al.*, 2013; Fonseca *et al.*, 2014), and confirmed that JAZ7 can bind to the transcriptional repressor JAM1 (Fig. 11C).

Combined, our results demonstrate through direct recruitment of TPL, in wild-type plants JAZ7 functions as a repressor within the JA-response network via its interaction with specific transcriptional regulators (e.g. MYC3, MYC4, JAM1). In *jaz7-1D* plants, we propose the misregulated expression of *JAZ7* would obstruct the finely-tuned nature of the COI1-JAZ-TPL-TF multi-protein complex resulting in hyperactivation of JA-signaling.

## Discussion

JA-signaling functions as a major determinant of disease outcome in Arabidopsis to the fungal pathogen *F. oxysporum* (Anderson *et al.*, 2004; Berrocal-Lobo and Molina 2004; McGrath *et al.*, 2005; Kidd *et al.*, 2009; Thatcher *et al.*, 2009, 2012a). In this study we analyzed the roles of JAZ proteins, repressors of JA-signaling, in *F. oxysporum* resistance or susceptibility. We identified a highly susceptible T-DNA insertion line (*jaz7-1D*) with a promoter insertion resulting in constitutive *JAZ7* expression and enhanced susceptibility to *F. oxysporum*. The *jaz7-1D* line also conferred increased JA-sensitivity, up-regulation of defense and JA-mediated gene expression, and increased susceptibility to the bacterial pathogen *Pst* DC3000. Both *F. oxysporum* and *Pst* DC3000 appear to target host JA- signaling to elicit disease, the first to hyperactivate JA-signaling and senescence processes, and the second to antagonistically suppress defense responses mediated by salicylic acid signaling. Thus the *jaz7-1D* line interferes with defense responses that integrate signals downstream of pathogens with two different virulence strategies.

We found the majority of *JAZ* genes were induced following *F. oxysporum* inoculation, with the largest inductions observed in root tissues for *JAZ5* and *JAZ10* (Fig. 1). There were also differences in individual *JAZ* root and leaf temporal expression patterns suggesting that some *JAZ* proteins may play unique roles in different tissue types. The largest inductions were observed for *JAZ5, JAZ7, JAZ8, JAZ9* and *JAZ10* (Fig. 1). These genes are also highly induced by *B. cinerea*, *Pst*, and/or herbivory (Chung *et al.*, 2008; data extracted from Genevestigator in Hruz *et al.*, 2008; Demianski *et al.*, 2012). *JAZ7* and *JAZ9* are also highly induced during senescence, which is promoted by *F. oxysporum* infection (data extracted from Genevestigator in Hruz *et al.*, 2008).

The strong inducibility of several *JAZ* genes by *F. oxysporum* and other pathogens/pests led us to screen available T-DNA insertion lines in *JAZ* genes for altered *F. oxysporum* disease phenotypes. While most overexpression or knockout lines of individual *JAZ* genes lack observable JA-related phenotypes, suggesting functional redundancy amongst the JAZ proteins (reviewed in Wasternack and Hause, 2013), we identified the *jaz7-1D* T-DNA insertional activation mutant which conferred hyper-activation of JA-signaling including up-regulation of JA-regulated biosynthesis, defense and senescence-associated genes (Fig. 8), as well as up-regulation of most other *JAZ* genes (Fig. 9). In an unbiased approach to identify genes differentially regulated in *jaz7-1D*, our microarray analysis identified genes up-regulated ≥2-fold in *jaz7-1D* over wild-type to be significantly enriched for involvement in stress and defense responses. The most highly up-regulated gene (9.5-fold) *NATA1* in the *jaz7-1D* mutant encodes a N-acetyltransferase, which acetylates ornithine to produce the defense-related metabolite Nδ-acetylornithine. Yan *et al.* (2014) also found this metabolite is more abundant in SALK_040835 (*jaz7-1D*) and its levels are highly up-regulated over wild-type following MeJA treatment. *NATA1* expression is highly responsive to JA, *Pst* and herbivory (Adio *et al.*, 2011) and a knockout mutant of *NATA1* has increased resistance to *Pst* DC3000 (Adio *et al.*, 2011), supporting our results for *jaz7-1D*. Adio *et al.* (2011) suggest that *Pst* DC3000 infection is promoted by coronatine/MeJA-induced expression of *NATA1* and subsequent production of Nδ-acetylornithine. Although *Thi2.1*, the second most highly up-regulated gene in *jaz7-1D*, has been linked to increased *F. oxysporum* resistance (Epple *et al.*, 1997; Chan *et al.*, 2005; Thatcher *et al.*, 2012a), *Thi2.1* is not a single determinant of *F. oxysporum* resistance. Indeed, other mutants with constitutive *Thi2.1* expression (e.g. *cpr5*) are highly susceptible while *coi1* plants with severely compromised *Thi2.1* expression are highly resistant (Bowling *et al.*, 1997; Schenk *et al.*, 2005; Thatcher *et al.*, 2009). Another gene highly up-regulated in *jaz7-1D* was *Histone1-3* (*HIS1-3*). *HIS1-3* encodes a linker histone which functions as a stabilizer of chromatin structure and its expression is highly drought inducible, suggestive of a role in stress tolerance (Ascenzi and Gantt, 1999). Recently it was found that JAZ7 plays a role in negative regulation of dark-induced leaf senescence (Yu *et al.*, 2015). Through analysis of the *jaz7-1* (WiscDsLox7H11) knockout line, Yu and colleagues found senescence and H_2_O_2_-mediated responses and genes involved in these processes such as *NATA1* and *DIN11* were significantly up-regulated in *jaz7-1* in darkness but not under light conditions. We found no alteration in *Fusarium*-induced senescence responses or oxidative stress responsive gene expression in *jaz7-1* compared to wild-type plants (Figs 4, 8). Thus it appears JAZ7 plays contrasting roles in pathogen and dark-induced senescence responses.

In addition to hyperactivation of JA-responses, the *jaz7-1D* mutant displayed an early flowering phenotype (Fig. 6). Links between flowering time and altered JA-mediated pathogen resistance have been reported previously. For example, the *pft1/med25* mutant is delayed in flowering, exhibits down-regulated JA-defense responses and increased resistance to *F. oxysporum* (Kidd *et al.*, 2009). It has been shown COI1-dependent signaling delays flowering time via JAZ degradation and inhibiting the expression of *FLOWERING LOCUS T* (*FT*) (Zhai *et al.*, 2015). Although increased JA-signaling and *JAZ* expression is evident in *jaz7-1D* plants, we did not detect altered expression of *FT* in our microarray analysis. However, other genes known to regulate flowering were altered (e.g. *DET2/DWF6*). The constitutive activation of JA-signaling in *jaz7-1D* may also be responsible for its small rosette phenotype and reduced root-length (Figs 2A, 7C). Many other mutants with constitutive JA-defense gene expression (e.g. *cpr5, cev1, cet1, dnd1, dnd2*) also show stunted growth (Bowling *et al.*, 1997; Ellis and Turner, 2001; Hilpert *et al.*, 2001; Genger *et al.*, 2008). Without stringent regulation, constant activation of JA responses would place large demands on plant resources, repressing growth, and likely contribute to these dwarf phenotypes (Baldwin, 1998; Kazan and Manners, 2012; Pieterse *et al.*, 2014). This is supported by the finding that defense and stress-related metabolites are increased in *jaz7-1D*/SALK_040835C which may limit resources available for growth (Yan *et al.*, 2014). Basal expression of JA-marker genes in the *JAZ7* overexpression lines (*JAZ7-OX*) that we generated was also increased, but not to the significantly high levels observed in *jaz7-1D*, and may account for why the *JAZ7-OX* lines did not exhibit the stunted *jaz7-1D* root and leaf phenotypes. To rule out the possibilities that altered *JAZ7* transcripts (e.g. mutated, mis-spliced) or other T-DNA insertions in *jaz7-1D* are responsible for its JA-hyperactivation phenotypes, we conducted several additional analyses and backcrossed *jaz7-1D* to wild-type plants. Our results suggest the T-DNA insertion within the *JAZ7* promoter is associated with the *jaz7-1D* phenotypes. However we cannot exclude the possibility that undetected secondary mutations or possible chromosomal rearrangements resulting from T-DNA transformation may contribute.

**Fig. 14.**
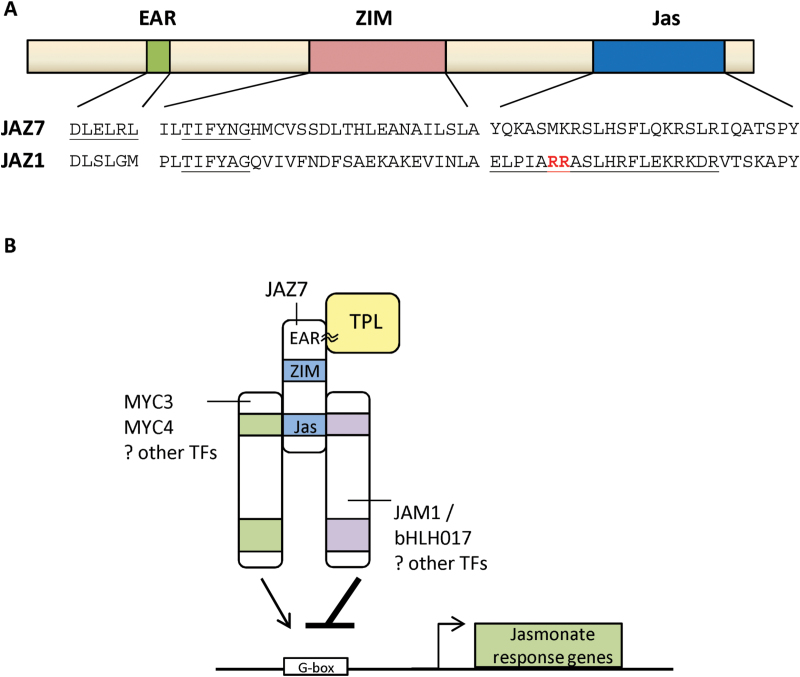
JAZ7 domain structure and proposed mode of JA-hyper-activation in *jaz7-1D* plants. (A) JAZ7 domain structure highlighting the N-terminal EAR motif, ZIM and Jas domains, and a comparison against conserved JAZ interaction domains in JAZ1. The EAR motif, TIFY motif and JAZ degron for the ZIM and Jas domains respectively are underlined. Residues in the JAZ1 Jas motif shown in bold red are required for COI1-binding. In JAZ1, the ZIM domain mediates NINJA binding and JAZ homo- and heterodimerization, and the Jas domain mediates COI1 binding and interactions with several transcription factors. (B) Proposed model for JA-responses in *jaz7-1D* plants. Through its EAR domain, JAZ7 binds with the co-repressor TPL to facilitate transcriptional repression. High levels of JAZ7 are associated with hyper-activation of JA-signaling possibly through JAZ7 disturbing components of this network (e.g. TPL, JAM1).

For other JAZ proteins characterized to date, JA-related phenotypes such as JA-insensitivity, sterility or altered tolerance to pathogens or pests have only been identified for *JAZ8* and *JAZ13* overexpressing lines (Shyu *et al.*, 2012; Thireault *et al.*, 2015), *jaz10* T-DNA or RNAi knockdown lines (Cerrudo *et al.*, 2012; Leone *et al.*, 2014), or in modified JAZ proteins in which the conserved C-terminal Jas motif has been deleted or its critical amino acids modified. These alterations stabilize the JAZ protein by preventing its interaction with COI1 and subsequent ubiquitin-mediated degradation following JA-stimulation (Chini *et al.*, 2007; Thines *et al.*, 2007; Yan *et al.*, 2007; Chung *et al.*, 2008; Chung and Howe, 2009). More specifically, the N-terminal domain of the Jas motif was identified as the COI1 and JA-Ile/COR binding site and termed the JAZ degron (Sheard *et al.*, 2010). A LPIARR sequence in the JAZ degron binds JA-Ile and COI1 in a clamp (Sheard *et al.*, 2010). This sequence is diverged in JAZ5, JAZ6, JAZ7 (Fig. 14A) and JAZ8, with both JAZ7 and JAZ8 lacking the RR or RK amino acid combination shown to be critical for COI1 binding (Melotto *et al.*, 2008; Sheard *et al.*, 2010; Shyu *et al.*, 2012). Shyu *et al.* (2012) attributed the JAZ8 Jas motif’s divergence from the canonical JAZ degron to its very weak ability to associate with COI1. To our knowledge, the COI1-binding capacity of the JAZ7 Jas motif is unknown. However, with high similarity to the divergent JAZ8 Jas motif (Shyu *et al.* 2012), it is likely JAZ7 either does not or only weakly associates with COI1. JAZ13 was recently identified as a non-TIFY domain containing JAZ protein that represses JA-signaling and is most similar to JAZ8. It appears JAZ13 may also be resistant to JA-COI1-mediated degradation (Thireault *et al.*, 2015). How poorly COI1-interacting JAZ proteins are degraded remains unknown, but Shyu *et al.* (2012) propose either they are eliminated via a COI1-independent mechanism, are degraded only under very high JA-Ile levels or through COI1-interactions mediated by molecules other than JA-Ile or COR. It is possible that JAZ7 escapes degradation through this mechanism and this allows JAZ7 to fine-tune the signaling pathway. Coupled with the high levels of *JAZ7* expression in *jaz7-1D*, the low degradability of JAZ7 protein would produce a highly modified JA-signaling environment, as observed through our microarray data and *jaz7-1D* phenotypes.

Expected from JAZ repressors, knockdown of *jaz1* or *jaz10*, and overexpression of *JAZ8* or *JAZ13* result in increased or reduced JA-sensitivity respectively (Yan *et al.*, 2007; Grunewald *et al.*, 2009; Demianski *et al.*, 2012; Shyu *et al.*, 2012; Thireault *et al.*, 2015). The *jaz7-1D* mutant, however, displays increased JA-sensitivity coupled with up-regulated defense and JA-mediated gene expression. Our results, showing JAZ7 indeed acts as repressor and can bind to both transcriptional activators (e.g. MYC3) and repressors (e.g. JAM1) of JA-responses (Figs 10–12), suggest that JA-sensitivity in *jaz7-1D* may result from the high and/or ectopic levels of JAZ7 inhibiting normal COI1-JAZ-TPL-TF interactions and highlights one of the difficulties in dissecting the individual roles of proteins that act within multiprotein complexes. The MYC-like bHLH transcription factors bHLH017/JAM1, bHLH013/JAM2 and bHLH003/JAM3 are phylogenetically closely related to MYC2, MYC3 and MYC4, but they lack the conserved activation domain present in the later MYC transcription factors (Fonseca *et al.*, 2014; Sasaki-Sekimoto *et al.*, 2014). Interestingly, *jaz7-1D* and a loss-of-function *jam1* mutant exhibit similar JA-related phenotypes, including small rosette size, increased JA-regulated gene expression (e.g. *MYC2*, *VSP*, *JAZ10*) and increased susceptibility to *Pst* DC3000 (Nakata *et al.*, 2013; Fonseca *et al.*, 2014). Other individual or triple loss-of-function *jam* mutants also display similar JA-related phenotypes (Nakata *et al.*, 2013; Sasaki-Sekimoto *et al.*, 2013; Song *et al.*, 2013; Fonseca *et al.*, 2014). The overlap in enhanced JA-responsiveness between *jaz7-1D* and these *jam* mutants (Sasaki-Sekimoto *et al.*, 2013) suggests the *jaz7-1D* phenotypes we observed may be mediated by the high levels of JAZ7 titrating out transcriptional repressors such as JAM1.

JAM1, JAM2 and JAM3 bind the same DNA motif (G-box, CACGTG) as MYC2, MYC3 and MYC4 (Nakata *et al.*, 2013; Fonseca *et al.*, 2014), and through competitive binding for the same DNA-binding site, these transcriptional repressors and activators can fine-tune JA-mediated responses. An unbiased *in silico* search (TAIR motif analysis: Statistical Motif Analysis in Promoter or Upstream Gene Sequences, 1000bp) for G-box motifs (Dombrecht *et al.*, 2007; Fernandez-Calvo *et al.*, 2011) in the promoters of the up-regulated genes in *jaz7-1D* (Supplementary Table S5) identified 19 to contain the CACGTG G-box motif, and 43 and 38 to contain the MYC2 binding variants CACATG and CACGTT, respectively (Dombrecht *et al.*, 2007). The promoters of down-regulated *jaz7-1D* (Supplementary Table S6) genes also contained these motifs (CACGTG: 7; CACATG: 8; CACGTT: 4). These findings suggest JAZ7 co-ordinates the expression of stress-responsive genes through its interaction with specific MYC or JAM transcription factors and their binding to G-box DNA motifs.

The ZIM domain of JAZ proteins mediates their homo- or heterodimerization (Chini *et al.*, 2009; Chung and Howe, 2009; Chung *et al.*, 2009), but JAZ7 appears to be the only JAZ protein incapable of homodimerizing or forming heterodimers with other JAZ proteins (Chini *et al.*, 2009; Chung and Howe, 2009; reviewed by Pauwels and Goossens, 2011). Another TIFY-containing protein not capable of interacting with JAZ proteins is the non-JAZ protein TIFY8 (Cuéllar Pérez *et al.*, 2014). Although TIFY8 has a functional ZIM domain that mediates transcriptional repression by recruiting TPL via NINJA, its ZIM domain does not confer interactions with JAZ proteins. The differences in JAZ7 protein-protein interactions suggest JAZ7 does not function like the other JAZ repressors. Further to this, although Jas and ZIM motifs in JAZ7 and JAZ8 are similar, suggestive of similar binding activity (Shyu *et al.*, 2012; Wager and Browse, 2012), they regulate binding to different transcription factors. For example, we found JAZ7 and JAZ8 interacted with MYC3/4 and JAM1, but only JAZ8 interacted with MYC2. JAZ8 but not JAZ7, also interacts with JAM2 (Song *et al.*, 2013; Fonseca *et al.*, 2014), with two regulators of stamen development (MYB21 and MYB24) (Song *et al.*, 2011) and with WD-repeat/bHLH/MYB complex members that regulate anthocyanin biosynthesis and trichome initiation (EGL3, GL3, TT8, MYB75, GL1, TTG1) (Qi *et al.*, 2011). These differences in transcription factor binding may explain why JAZ8 overexpression confers reduced JA-sensitivity (Shyu *et al.*, 2012) while high levels of *JAZ7* in *jaz7-1D* plants confers increased JA-sensitivity (this work).

In summary, our results support a model in which *F. oxysporum* stimulates JA-signaling, resulting in increased *JAZ7* expression and JAZ7-TPL-mediated repression contributing to the control of JA-responses and disease progression. Our characterization of the *jaz7-1D* mutant suggests the ectopic or non-wild-type high levels of *JAZ7* in *jaz7-1D* is a major determinant of its phenotypes and that these abnormal levels may be detrimental to the normal COI1-JAZ-TPL-MYC/JAM regulatory network leading to hyperactivation of JA-signaling (Fig. 14B). Additionally, the unusual protein binding properties of JAZ7 compared to other JAZs may exacerbate this phenotype (e.g. lack of homo- or heterodimerization, divergent JAZ degron). Our results also exemplify the need to use caution when interpreting results from T-DNA insertion lines and proteins that act in multiprotein complexes. Nonetheless, identification of JA-hyperactivation in the *jaz7-1D* mutant has provided new insight into JA-signaling and why a plant needs many JAZ proteins to fine-tune JA-responses. Future research on *JAZ7* expression (tissue/cell specificity) and its interacting partners should reveal mechanistic details on how JAZ7 functions in wild-type plants.

## Supplementary data

Supplementary data are available at *JXB* online.

Fig. S1. Schematic representation of *jaz* T-DNA insertion lines. 

Fig. S2. Screening of *jaz* T-DNA insertion lines in *F. oxysporum* disease assays.

Fig. S3. Detection of seed aborts in *jaz7-1D* and confirmation of *jaz7-1.*

Fig. S4. Ectopic overexpression of *JAZ7* in wild-type plants.

Fig. S5. Backcrossed F2 *jaz7-1D* seedlings have short roots and are JA-hypersensitive. 

Table S1. *jaz* double and triple mutant lines screened in *F. oxysporum* disease assays.

Table S2. Primers used for the generation of transgenic plants and Y2-H and Co-IP constructs.

Table S3. Primers used for qRT-PCR.

Table S4. List of genes differentially regulated by genotype from the microarray.

Table S5. Genes differentially expressed ≥ 2-fold in the *jaz7-1D* line relative to wild-type.

Table S6. Genes differentially expressed ≤ 2-fold in the *jaz7-1D* line relative to wild-type.

Table S7. List of genes differentially regulated by MeJA treatment from the microarray.

Table S8. Genes differentially expressed ≥ 2-fold in the *jaz7-1D* line relative to wild-type under MeJA treatment.

Table S9. Genes differentially expressed ≤ 2-fold in the *jaz7-1D* line relative to wild-type under MeJA treatment.

Table S10. Differentially regulated by MeJA treatment genes sorted by MeJA inducibility in wild-type plants.

Supplementary Data
