# Supplementary material for: Characterization of a JAZ7 activation-tagged Arabidopsis mutant with increased susceptibility to the fungal pathogen Fusarium oxysporum
Source: J Exp Bot. 2016 Feb 19;67(8):2367–86. doi: 10.1093/jxb/erw040 (PMC4809290; doi:10.1093/jxb/erw040)
Supplement: Supplementary Data [file supp_67_8_2367__index.html]

Characterization of a JAZ7 activation-tagged Arabidopsis mutant with increased susceptibility to the fungal pathogen Fusarium oxysporum — Characterization of a JAZ7 activation-tagged Arabidopsis mutant with increased susceptibility to the fungal pathogen Fusarium oxysporum — Supplementary Data 

# Characterization of a *JAZ7* activation-tagged Arabidopsis mutant with increased susceptibility to the fungal pathogen *Fusarium oxysporum*

## Supplementary Data

Data files

- supplementary\_figures\_S1\_S5.pdf - Supplementary Data
- supplementary\_tables\_S1\_S10.pdf - Supplementary Data
