# Supplementary material for: Characterization of a JAZ7 activation-tagged Arabidopsis mutant with increased susceptibility to the fungal pathogen Fusarium oxysporum
Source: J Exp Bot. 2016 Feb 19;67(8):2367–86. doi: 10.1093/jxb/erw040 (PMC4809290; doi:10.1093/jxb/erw040)
Supplement: Supplementary Data [file supp_erw040_supplementary_figures_S1_S5.pdf]

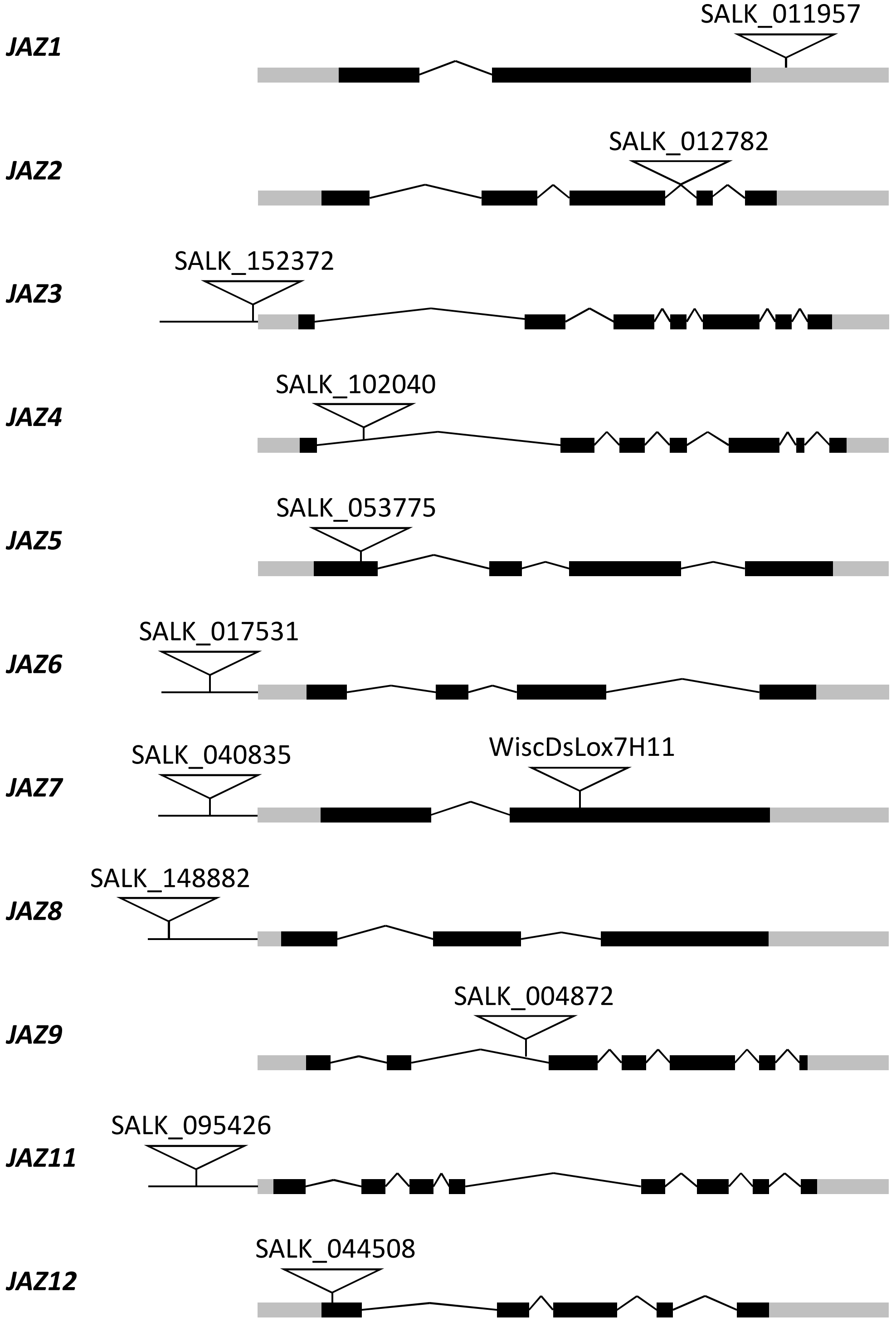

Supplementary Fig. S1. Schematic representation of *jaz* T-DNA insertion lines. Shown are details of T-DNA insertion lines used in this study. The exact location of the T-DNA inserts is not depicted and are not to scale. UTRs, exons and introns are represented by grey bars, black bars and raised lines, respectively.

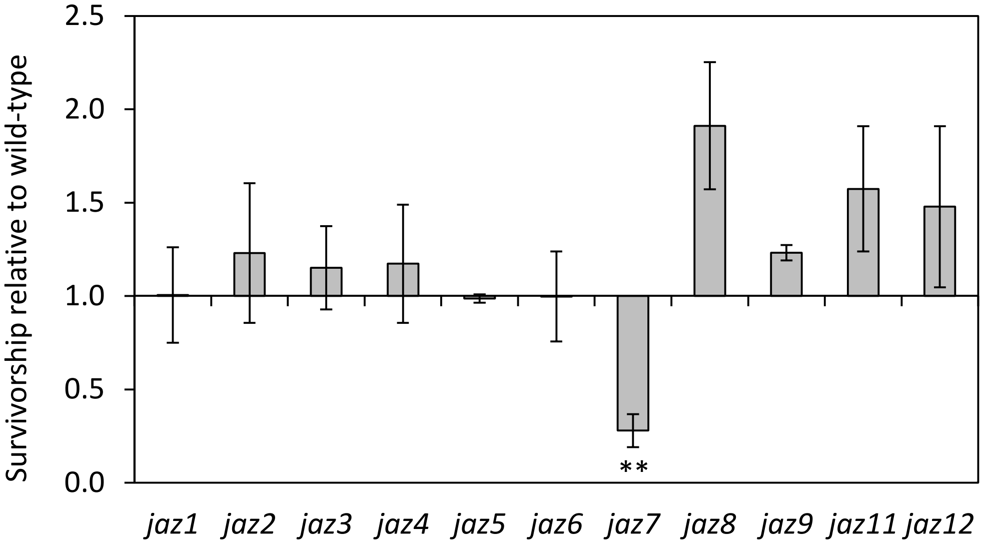

Supplementary Fig. S2. Screening of *jaz* T-DNA insertion lines in *F. oxysporum* disease assays. *jaz* T-DNA insertion lines were screened in *F. oxysporum* disease assays and survivorship recorded relative to wild-type plants at 21-days post inoculation. Shown is the average of two biological replicates ± SE (n=55). Asterisks indicate values that are significantly different (**P<0.01 Student's *t*-test) from WT.

A

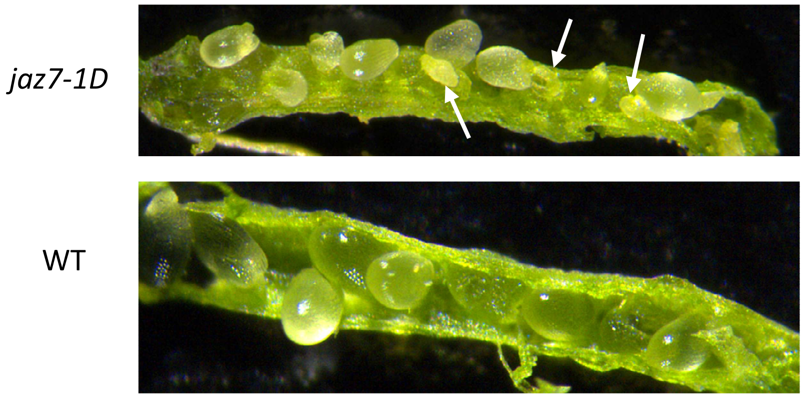

B

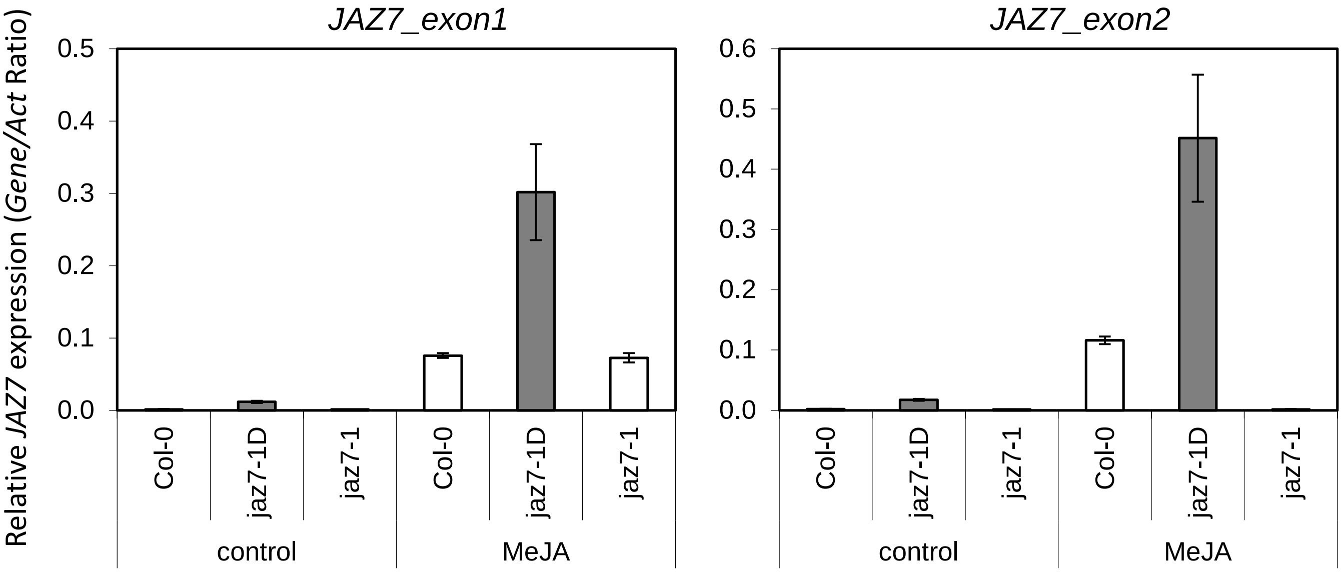

Supplementary Fig. S3. Detection of seed aborts in *jaz7-1D* and confirmation of *jaz7-1*. (A) Siliques of *jaz7-1D* and WT were examined for seed aborts with a 1:3 ratio (7:19) of aborts (indicated by arrows) to normal ovules observed in j*az7-1D*. (B) Primers specific to the first or second exon of *JAZ7* were used to screen for full or truncated transcripts derived from the *jaz7-1* T-DNA line WiscDsLox7H11. *JAZ7* transcript 5′ to the T-DNA insertion site was detected at levels similar to Col-0 however, it is unlikely 5′ *JAZ7* transcripts would produce a truncated protein as non-polyadenylated transcripts would be degraded if not correctly processed. No transcript was detected with primers specific to the second exon (3′ to T-DNA insertion site). Values are averages ± SE of three biological replicates consisting of pools of 10 plants. Gene expression levels are relative to the internal control *β-actin* genes.

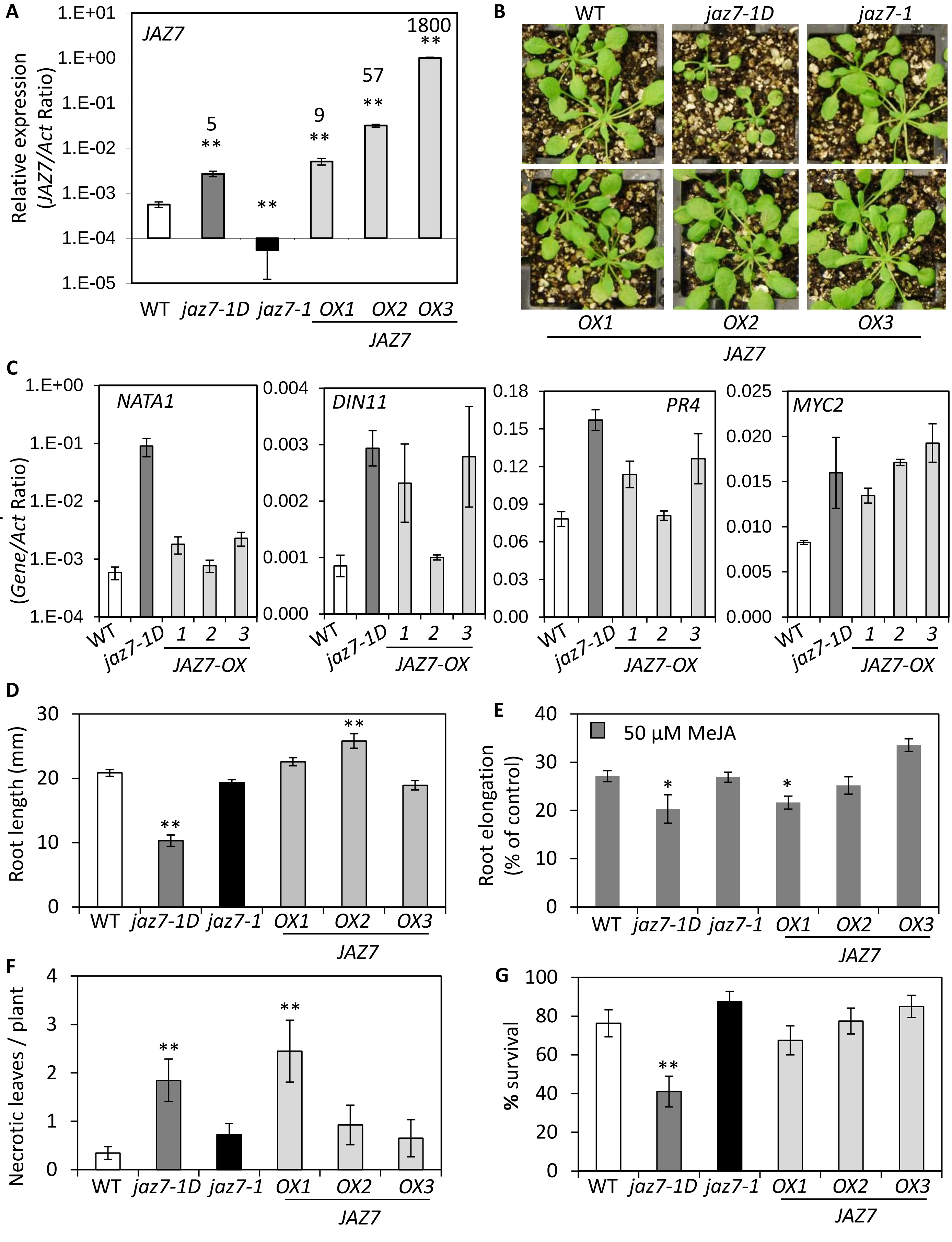

Supplementary Fig. S4. Ectopic over-expression of *JAZ7* in wild-type plants.

Supplementary Fig. S4. Ectopic over-expression of *JAZ7* in wild-type plants.

(A) Basal *JAZ7* expression in *35S:JAZ7* overexpression (*JAZ7-OX*) lines. Values are averages ± SE of three biological replicates consisting of pools of 10-20 plants. Shown also is fold increase in *JAZ7* expression over wild-type (WT). Gene expression levels are relative to the internal control *β-actin* genes. (B) Phenotypes of *jaz7* mutant lines. (C) Basal expression of JA-marker genes was examined in shoot tissue of wild-type (WT), *jaz7-1D* and *35S:JAZ7* over-expression (*JAZ7-OX*) plants. Values are averages ± SE of three biological replicates consisting of pools of 10 plants. Gene expression levels are relative to the internal control *β-actin* genes. (D-E) (D) Root growth on control media and (E) sensitivity of WT, *jaz7-1D*, *jaz7-1* and *JAZ7-OX* seedlings to MeJA 7-days post germination. Values are averages ± SE of three biological replicates consisting of pools of 10-15 seedlings. (F-G) *F. oxysporum* disease assays with (F) necrotic leaves per plant at 14-days post inoculation and (G) survival rates at 28-days. Values are averages ± SE (n=40). Asterisks indicate values that are significantly different (***P*<0.01, **P*<0.05, Student's *t*-test) from WT. Similar results were obtained in independent experiments.

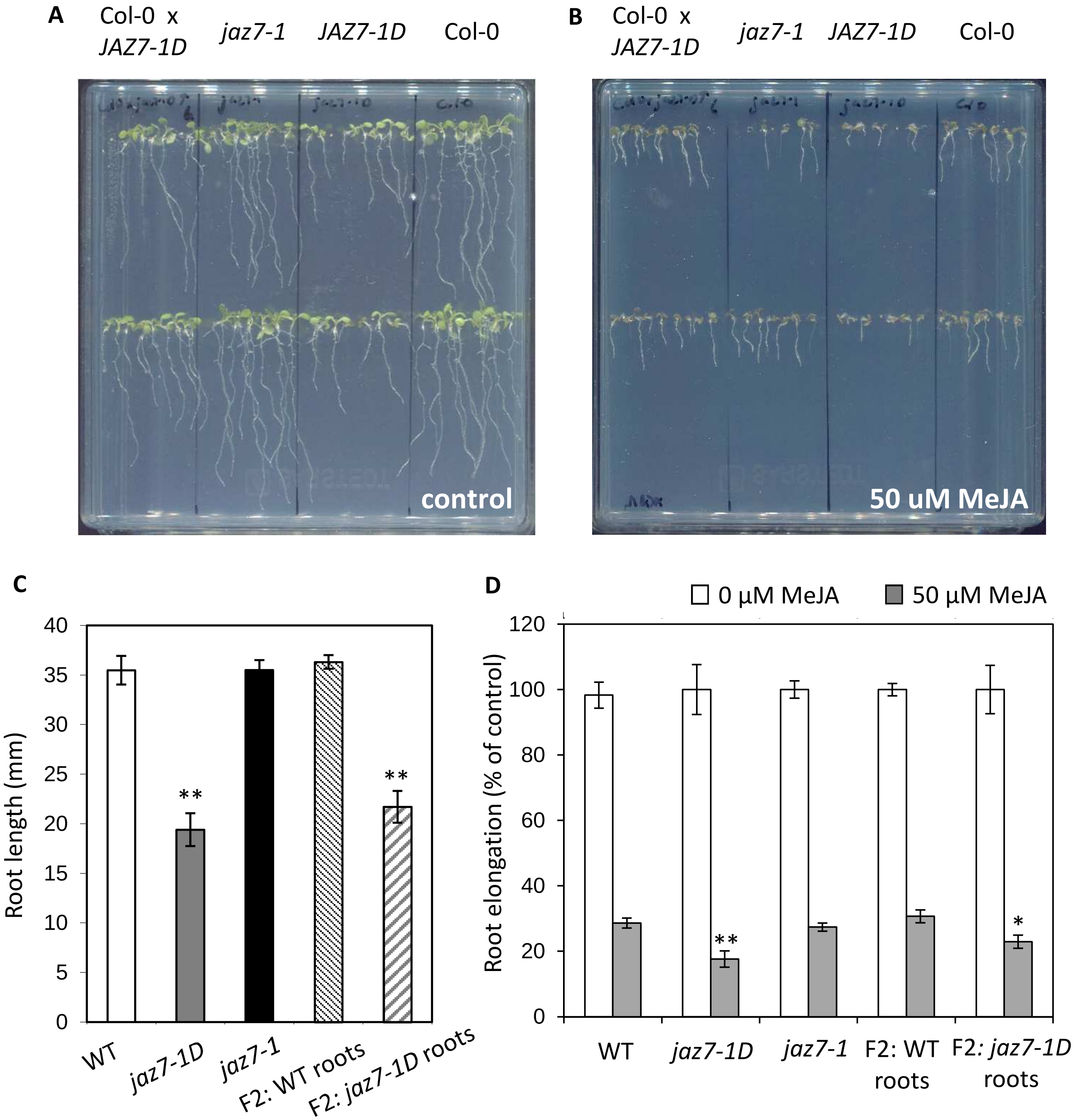

Supplementary Fig. S5. Backcrossed F2 *jaz7-1D* seedlings have short roots and are JA-hypersensitive. (A-D) Growth of wild-type (Col-0), *jaz7-1D*, *jaz7-1* and F_2 seedlings from a cross between Col-0 and *jaz7-1D* on (A) control media and (B) MeJA (50 uM) containing media 7-days post germination. (C-D) F_2 seedlings segregated 2:1 for heterozygous *jaz7-1D:*WT root lengths under control and MeJA treatments. F_2 Seedlings with *jaz7-1D* root phenotype were significantly different from WT but not from *jaz7-1D*. Root elongation of each line when grown on control media or media containing MeJA was calculated as a percentage relative to control treatment. Values are averages ± SE of 10-15 seedlings. Values that differed significantly from the WT were identified by the one-way Anova and Dunnet's post-hoc test (***P*<0.01, **P*<0.05). Similar results were obtained in an independent experiment.
