# Supplementary material for: Characterization of a JAZ7 activation-tagged Arabidopsis mutant with increased susceptibility to the fungal pathogen Fusarium oxysporum
Source: J Exp Bot. 2016 Feb 19;67(8):2367–86. doi: 10.1093/jxb/erw040 (PMC4809290; doi:10.1093/jxb/erw040)
Supplement: Supplementary Data [file supp_erw040_supplementary_tables_S1_S10.pdf]

Supplementary Table S1. *jaz* double and triple mutant lines screened in *F. oxysporum* disease assays.

Insertion lines used for double and triple mutants are jaz1 (SALK_011957), jaz2 (SALK_025279), jaz3 (SALK_139337), jaz4 (SALK_026633), jaz5 (SALK_053775), jaz6

***jaz* double mutants**	***jaz* triple mutants**
jaz2/7	*jaz2/5/10*
jaz5/7	*jaz3/5/10*
jaz6/7	*jaz5/6/10*
jaz3/10	*jaz5/7/10*
jaz5/10	
jaz7/10	
jaz1/3	
jaz2/5	
jaz3/5	

(SAIL_1156_C06), jaz7 (WiscDsLox7H11), jaz10 (SAIL_92_D08).

Supplementary Table S2. Primers used for the generation of transgenic plants and Y2-H and Co-IP constructs.

Primer ID	Primer sequence (5'-3')
JAZ7-*HindIII* -F	TGACAAGCTTAACAATGATCATCATCATCAAA
JAZ7-*EcoRI* -R	ATAGGAATTCCTATCGGTAACGGTGGTA
JAZ7-EAR-*HIII* - *Sma* -F	CAAAAAGCTTGAGCCCGGGACAAAATGCGACTTGGAACTTCGCC
JAZ7_EAR_STOP-*EcoRI* -R	CAGAGAATTCTCAAGAAGTAAGAAGGCGAAGTTCCAAGTCGCAT
CUC1-*HIII* -F	TTCTAAGCTTAACAATGGATGTTGATGTGTTTAACGGTTG
CUC1-STOPdel-*Sma* -R	CATGGCCCGGGGAGAGTAAACGGCCACACACTCACGGCGG
TPL-F	AAAAAGCAGGCTTCACCATGTCTTCTCTTAGTAGAGAGCTCG
TPL-R	CAAGAAAGCTGGGTTCATCTCTGAGGCTGATCAGATGC
JAM1-F	AAAAAGCAGGCTTCACCATGAATATGAGTGATTTAGGTTGG
JAM1-R	CAAGAAAGCTGGGTTTATATATCACCAGAGACCTGTG
MYC2-F	AAAAAGCAGGCTTCACCATGACTGATTACCGGCTACAAC
MYC2-R	CAAGAAAGCTGGGTACCGATTTTTGAAATCAAACTTGC
MYC3-F	AAAAAGCAGGCTTCACCATGAACGGCACAACATCATCAATC
MYC3-R	CAAGAAAGCTGGGTATAGTTTTCTCCGACTTTCGTC
MYC4-F	AAAAAGCAGGCTTCACCATGTCTCCGACGAATGTTCAAG
MYC4-R	CAAGAAAGCTGGGTTGGACATTCTCCAACTTTCTCC
JAZ7-F	AAAAAGCAGGCTTCACCATGATCATCATCATCAAAAACTG
JAZ7-R	CAAGAAAGCTGGGTCTATCGGTAACGGTGGTA
JAZ7-R2	CAAGAAAGCTGGGTCTCGGTAACGGTGGTAAGG
JAZ8-F	AAAAAGCAGGCTTCACCATGAAGCTACAGCAAAATTGTG
JAZ8-R	CAAGAAAGCTGGGTTTATCGTCGTGAATGGTAC
JAZ8-R2	CAAGAAAGCTGGGTCTCGTCGTGAATGGTACGG
JAZ5-F	AAAAAGCAGGCTTCACCATGTCGTCGAGCAATGAAAATGC
JAZ5-R	CAAGAAAGCTGGGTCTATAGCCTTAGATCGAGATCT
JAZ5-R2	CAAGAAAGCTGGGTCTAGCCTTAGATCGAGATCT
pAttB1	GGGGACAAGTTTGTACAAAAAAGCAGGCT
pAttB2	GGGGACCACTTTGTACAAGAAAGCTGGGT

Supplementary Table S3. Primers used for qRT-PCR.

Primer ID	Gene Locus (AGI)	Forward primer sequence (5'-3')	Reverse primer sequence (5'-3')
ACT-8	At1g49240		GAGGATAGCATGTGGAACTGAGAA
ACT-2	At3g18780		GATGGCATGGAGGAAGAGAGAAAC
ACT-7	At5g09810		GAGGAAGAGCATTCCCCTCGTA
ACTuni		AGTGGTCGTACAACCGGTATTGT	
JAZ1	At1g19180	CGGTAGCTTTGGAGATCTGAG	TCACAAGGGAATAAACTCATGG
JAZ2	At1g74950	CCTCTGGGACCAAAGAAGAT	CCATAACTCGACCACCGTAG
JAZ3	At3g17860	TTTATGATTCAACCCGCAAA	CGACATGGAAACTGCGTTAG
JAZ4	At1g48500	CAATCTTTTATGCCGGTTCA	CGGTTTAGCATGAGGTCCAT
JAZ5	At1g17380	CACGTAGAGCTTCCCTCCAT	TGGGAGGATAACGATGATGA
JAZ6	At1g72450	GGAACGCATTGCAAGAAGA	GGAAGATGACTACCGTGTTGG
JAZ7	At2g34600	GACTTGGAACTTCGCCTTCTT	TCTGAGATTCTTGCTTTGGTTG
JAZ7 exon1 specific	At2g34600	CATCATCAAAAACTGCGACAA	GAAGTAAGAAGGCGAAGTTCCA
JAZ7 exon2 specific	At2g34600	CAACAATCCCAAACAATTCG	GAAGTTGCTTGAATCCGAAGA
JAZ8	At1g30135	CAGCAAAATTGTGACTTGGAA	TGGATTTCCAGAACTGGTTG
JAZ9	At1g70700	CACAATCTTTTATGGCGGAAC	TCCAGTTTCACCTTTCAAACC
JAZ10	At5g13220	ATCTCGTTCGGTTCCGTCTA	ACAGTTCCCGAAACGAGTTC
JAZ11	At3g43440	TTTGGTGGGAGTTTTAGCGTA	TTGTCTCAGTGGCTTTAGCTG
JAZ12	At5g20900	GCTGCACAGCCATTTCCTA	CTCGAGGAATCGTTGAAGC
MYC2	At1g32640	TCATACGACGGTTGCCAGAA	AGCAACGTTTACAAGCTTTGATTG
ERF1	At3g23240	TCAGAAGACCCCAAAAGCTCCTCA	TTGATCACCGCTCCGTGAAGTTAG
LOX3	At1G17420	TAACGATGCTGGTGTCCATC	AATTGCCTGTGTGCAGCTAA
PDF1.2	At5g44420	TTTGCTGCTTTCGACGCAC	CGCAAACCCCTGACCATG
Thi2.1	At1g72260	CTCAGCTGATGCTACCAATGAGC	GCTCCATTCACAATTTCACTTGC
PR3-F	At3g12500	ATCAGCGCTGCAAAGTCCTTC	GTGCTGTAGCCCATCCACCTG
VSP2	At5g24770	CCTAAAGAACGACACCGTCA	TCGGTCTTCTCTGTTCCGTA
SAG12	At5g45890	AAAGGCGAAGACGCTACTTG	TCATCAGTGCTTGCTCATCA
GSTF6	At1g02930	GTCGACTTTGAATTCGTTCATG	CCTTGCCAGTTGAGAGAAGG
DHAR1	At1g19570	CTCTGACAAACCCCAGTGGTTC	CAACGATGACGTCGGAATCA
PR1	AT2G14610	AAGAGGCAACTGCAGACTCA	TCTCGCTAACCCACATGTTC
PR2	At3g57260	AGCTTAGCCTCACCACCAAT	CCCGTAGCATACTCCGATTT
NATA1	AT2G39030	TTCTTCCCGAACTACCCAAG	GCGGTCAGTAGCAGTTTTCC

Supplementary Table S4. List of genes differentially regulated by genotype from the microarray.

Genes were obtained using a 2-way ANOVA with a p-value cut-off of 0.05 and the addition of the Benjamini and Hochberg False Discovery rate. The list represents the genes that were differentially expressed by the genotypes from the entire microarray data set and are calculated irrespective of the treatment.

First parameter (Genotype) test.
All samples

Treatment ()*S		MeJA 6h	MeJA 6h	MeJA 6h	MeJA 6h	MeJA 6h	MeJA 6h	MeJA 6h	MeJA 6h	Control 6h	Control 6h	Control 6h	Control 6h	Control 6h	Control 6h	Control 6h	Control 6h	averages		averages		Fold change			
Genotype ()*S		Col-0	Col-0	Col-0	Col-0	*jaz7-1D*	*jaz7-1D*	*jaz7-1D*	*jaz7-1D*	Col-0	Col-0	Col-0	Col-0	*jaz7-1D*	*jaz7-1D*	*jaz7-1D*	*jaz7-1D*	MeJA 6h		Control 6h		MeJA	Control		
Replicate ()*		rep 1	rep 2	rep 3	rep 4	rep 1	rep 2	rep 3	rep 4	rep 1	rep 2	rep 3	rep 4	rep 1	rep 2	rep 3	rep 4								
Systematic	P-value	Normalized	Normalized	Normalized	Normalized	Normalized	Normalized	Normalized	Normalized	Normalized	Normalized	Normalized	Normalized	Normalized	Normalized	Normalized	Normalized	Col-0	*jaz7-1D*	Col-0	*jaz7-1D*	*jaz7-1D* /Col-0	*jaz7-1D* /Col-0	ATG#	TAIR description
263421_at	0.0498	1.4862722	1.5047331	1.3547609	1.5919921	1.0045558	1.0966278	0.77639776	1.0912569	0.5763131	0.80789566	0.99544406	1.1190491	0.42873004	0.54775065	0.8915885	0.5221	1.48444	0.99221	0.874675	0.597542	0.67	0.68	AT2G17230	phosphate-responsive 1 family protein similar to phi-1 (phosphate-induced gene) [Nicotiana tabacum] GI:3759184; contains Pfam profile PF04674: Phosphate-induced protein 1 conserved region
259105_at	0.0498	1.0821157	0.99285173	1.2444988	1.3169528	0.8878354	0.9283188	1.0071481	0.7569792	1.0102719	1.1218823	0.9261508	1.0134857	0.8288497	0.9004073	0.87026423	1.0329062	1.159105	0.89507	1.017948	0.908107	0.77	0.89	AT3G05500	rubber elongation factor (REF) family protein contains Pfam profile: PF05755 rubber elongation factor protein (REF)
264742_at	0.0497	0.92551476	0.82777905	0.9195633	0.80331236	1.0089419	1.0283083	1.2095639	1.3547981	1.020216	1.0618954	0.94024134	0.9165876	1.0417453	0.9910581	1.0367923	0.9785438	0.869042	1.150403	0.984735	1.012035	1.32	1.03	AT1G62130	AAA-type ATPase family protein contains Pfam domain, PF00004: ATPase, AAA family
249391_at	0.0497	0.8980152	0.97272366	0.8611959	0.9800382	1.2945895	1.1242725	1.0509506	1.2695016	0.97351706	1.1642373	0.96789706	0.9504187	1.0199618	1.1152251	0.9785926	1.137586	0.927993	1.18829	1.014018	1.062841	1.28	1.05	AT5G40140	armadillo/beta-catenin repeat family protein / U-box domain-containing protein contains Pfam domain, PF00514: Armadillo/beta-catenin-like repeats and Pfam, PF04564: U-box domain
260608_at	0.0496	0.75834036	0.89888895	0.7752425	0.8341978	0.98609155	1.4003341	1.0139084	0.8673056	0.75102544	1.3452836	0.9548409	1.1281571	1.2160655	2.3479943	2.3078442	1.5043497	0.816667	1.06691	1.044827	1.844063	1.31	1.76	AT2G43870	polygalacturonase, putative / pectinase, putative similar to SP|P48979 Polygalacturonase precursor (EC 3.2.1.15) (PG) (Pectinase) (Prunus persica); contains PF00295: Glycosyl hydrolases family 28 (polygalacturonases)
249346_at	0.0496	1.094892	1.1592052	1.171127	0.97568417	0.99611354	0.9876521	0.9491765	0.71368355	1.0219034	1.0565232	1.044175	1.1174922	0.8835339	1.0038866	0.9260213	0.8445257	1.100227	0.911656	1.060023	0.914492	0.83	0.86	AT5G40770	prohibitin identical to prohibitin [Arabidopsis thaliana] GI:1946331
257426_at	0.0496	1.1635492	1.2841889	1.2367219	1.1427772	1.0273955	0.91269267	0.9986612	0.52882516	1.0302165	1.1808836	1.0013388	0.94306487	0.90463257	0.70692515	0.7864233	0.8660615	1.206809	0.866894	1.038876	0.816011	0.72	0.79	AT1G54850	expressed protein contains similarity to LMW heat shock protein GI:2326354 from (Arabidopsis thaliana)
254023_at	0.0496	1.2350034	1.2441876	1.2094198	1.0618038	1.0051038	1.108592	0.9475731	1.0608022	0.9948963	1.0832149	0.97047657	0.8925292	0.934402	0.8630037	0.92505324	0.85115933	1.187604	1.030518	0.985279	0.893405	0.87	0.91	AT4G25770	expressed protein
250479_at	0.0494	1.0241455	1.0376686	1.1932002	1.1253026	0.93215525	0.9605392	0.88126963	0.72392964	0.99189675	1.1641768	1.2345003	1.0114574	0.7066454	1.0081031	0.9649561	0.9598577	1.095079	0.874473	1.100508	0.909891	0.80	0.83	AT5G10260	Ras-related GTP-binding protein, putative similar to Ras-related protein Rab-6A SP:P20340 from [Homo sapiens]
247362_at	0.0494	1.2754213	1.617904	1.0035796	1.1836594	0.8426498	0.9407055	0.9593338	1.0049646	1.1341051	0.93634593	1.3537076	1.408035	0.99642044	0.994894	0.91763794	0.98054355	1.270141	0.936913	1.208048	0.972374	0.74	0.80	AT5G63140	calcineurin-like phosphoesterase family protein contains Pfam profile: PF00149 calcineurin-like phosphoesterase
245299_at	0.0494	0.9619952	1.710937	1.1541921	1.1028078	0.8224669	0.6388104	1.0048221	0.51905245	1.410187	1.2714512	0.995178	1.0355017	1.1690276	0.8418572	0.8026878	0.82491076	1.197522	0.746288	1.178079	0.909621	0.68	0.77	AT5G47200;AT4G17	[AT5G47200, Ras-related GTP-binding protein, putative similar to GTP-binding protein GI:303750 from [Pisum sativum]];[AT4G17530, Ras-related GTP-binding protein, putative very strong similarity to RAB1C [Lotus corniculatus var. japonicus] GI:1370166; contains Pfam profile PF00071: Ras family]
263219_at	0.0493	1.0818533	0.8138229	0.92989135	0.81665593	1.1578718	1.131553	1.1066414	1.2302617	0.9421182	0.9877175	1.0044873	0.95299804	1.0311588	1.206049	0.9955128	0.94436944	0.910556	1.155733	0.97183	1.043773	1.27	1.07	AT1G30600	subtilase family protein Strong similarity to gb|U80583 proteinase TMP from Lycopersicon esculentum and is a member of the PF|00082 subtilase family
255082_at	0.0493	0.9588409	1.0700443	1.1822869	1.1788219	0.9753396	0.9182682	0.69821995	1.120739	1.0461837	1.0246605	1.0623857	1.210083	0.75701	0.848363	0.95457405	0.7495633	1.097499	0.928	1.085828	0.827378	0.85	0.76	AT4G09160	SEC14 cytosolic factor family protein / phosphoglyceride transfer family protein similar to polyphosphoinositide binding protein Ssh1p (GI:2739044) [Glycine max]; similar to polyphosphoinositide binding protein Ssh2, Glycine max, gb:T05953; contains Pfam PF00650 : CRAL/TRIO domain; contains Pfam PF03765 : CRAL [illegible]
264886_at	0.0492	1.757844	1.2487004	1.8716174	3.0498557	3.9200373	2.5113072	4.5322666	8.276384	0.39356676	0.27134967	0.26652253	0.35602188	0.42890537	0.42412502	0.37110794	0.75129974	1.982004	4.809999	0.321865	0.49386	2.43	1.53	AT1G61120	terpene synthase/cyclase family protein similar to S-linalool synthase GI:1491939 from [Clarkia breweri][PMID: 8768373]
247442_s_at	0.0491	1.0643959	1.1820515	1.0022572	1.0225179	1.0753497	0.9687122	0.9264661	1.0613691	0.9468729	1.0826755	0.99774265	1.18353	0.7922325	0.84042674	0.93519473	0.76299256	1.067806	1.007974	1.052705	0.832712	0.94	0.79	AT5G62700;AT5G62	[AT5G62700, tubulin beta-2/beta-3 chain (TUB3) nearly identical to SP|P29512 Tubulin beta-2/beta-3 chain (Arabidopsis thaliana)];[AT5G62690, tubulin beta-2/beta-3 chain (TUB2) nearly identical to SP|P29512 Tubulin beta-2/beta-3 chain (Arabidopsis thaliana)]
255589_s_at	0.0491	1.1473166	1.2306592	0.98168457	1.1297811	0.69816464	0.9900946	0.79944444	0.8049616	1.0504789	0.84795386	1.177588	1.1805928	1.015761	0.8462323	1.0099053	0.87312543	1.12236	0.823166	1.064153	0.936256	0.73	0.88	AT4G01590	expressed protein
246506_at	0.0491	1.0023594	0.9976406	1.2555957	1.0700967	1.0135086	0.82385826	1.0162596	0.7312236	1.0978024	1.1459165	0.9504871	1.0649654	0.92988414	0.78442997	0.9374942	0.81812596	1.081423	0.896213	1.064793	0.867484	0.83	0.81	AT5G16110	expressed protein hypothetical protein T26J14.6 - Arabidopsis thaliana, EMBL:AC011915
260546_at	0.0491	0.9510293	0.9966169	0.7808749	1.003383	2.5853229	1.4628408	2.2478418	0.59576005	0.7843593	0.61736107	0.6847802	0.5066034	1.8624731	1.2400056	1.3024056	1.2639924	0.932976	1.722941	0.648276	1.417219	1.85	2.19	AT2G43520	trypsin inhibitor, putative similar to SP|P26780 Trypsin inhibitor 2 precursor (MTI-2) (Sinapis alba)
246311_at	0.0491	0.90994984	1.2407744	1.096053	1.2051105	0.7762538	0.9529621	0.84986454	0.50050026	1.2206213	1.2906651	1.0470377	1.0505216	1.0860952	0.85792047	0.8795715	0.8743744	1.112972	0.769895	1.152211	0.92449	0.69	0.80	AT3G51880	high mobility group protein alpha (HMGalpha) / HMG protein alpha nearly identical to HMG protein (HMGalpha) [Arabidopsis thaliana] GI:2832357; contains Pfam profile PF00505: HMG (high mobility group) box
266203_at	0.049	0.8731549	1.0287268	0.90349203	1.0523783	0.80430657	0.77593386	0.8751842	0.7321283	1.19575	1.0165642	1.0261337	1.0704176	1.0603677	0.9635538	0.98343575	1.0200789	0.964438	0.796888	1.077216	1.006859	0.83	0.93	AT2G02230	F-box family protein / SKP1 interacting partner 3-related contains similarity to SKP1 interacting partner 3 GI:10716951 from [Arabidopsis thaliana]
248751_at	0.049	1.1614562	1.3848678	1.361495	1.3552303	1.1048021	0.95982504	1.0047408	0.9936474	0.9375207	0.8467048	1.033050	1.0757581	0.9952592	0.95505345	0.9608854	0.81671786	1.315762	1.015754	0.973258	0.931979	0.77	0.96	AT5G47540	Mo25 family protein similar to MO25 protein (early mouse development protein family) [Mouse] SWISS-PROT:Q06138
253938_at	0.049	1.340364	1.0507331	1.2771016	1.1116463	1.0335165	0.946361	0.97542864	0.8659194	1.0025724	1.061643	1.0395341	0.9205163	0.9892742	0.9974274	0.8305636	0.897894	1.194961	0.955306	1.006066	0.92879	0.80	0.92	AT4G26920	hypothetical protein
253441_at	0.049	1.0961655	1.089722	1.1931086	1.0531058	0.9424669	1.0347898	0.924479	0.9276669	1.1561738	1.0471112	0.9746789	0.94474983	1.0253212	0.93652755	0.83690083	0.91335016	1.108025	0.957351	1.030678	0.928025	0.86	0.90	AT4G32560	paramyosin-related contains weak similarity to Paramyosin (Swiss-Prot:P10567) [Caenorhabditis elegans]
251453_at	0.049	1.020019	0.94147784	0.87592264	0.84160995	1.0897216	1.1960826	1.1895802	0.9791994	0.9845284	0.9475479	1.0309148	0.94355	0.9718448	1.0154716	1.155704	1.075389	0.919757	1.113646	0.976587	1.054602	1.21	1.08	AT3G60070	lactose permease-related contains weak similarity to Lactose permease (Lactose-proton symport) (Lactose transport protein). (Swiss-Prot:O33814) [Staphylococcus xylosus]
261803_at	0.0489	1.0012261	1.1418461	1.0641221	0.99877405	0.6541418	0.65198267	0.7714817	0.36855423	1.1417458	0.9646756	1.0687487	1.1718717	1.2036082	0.9824009	1.0256157	0.94335407	1.051492	0.61154	1.08676	1.038745	0.58	0.96	AT1G30500	CCAAT-binding transcription factor (CBF-B/NF-YA) family protein contains Pfam profile: PF02045 CCAAT-binding transcription factor (CBF-B/NF-YA)
250383_at	0.0489	1.0547737	1.0453154	0.936137	0.99244646	0.8846485	0.8524794	0.8491858	0.68862134	1.1208171	1.0164331	1.0319086	1.0239518	1.0075557	1.0820463	0.84878165	0.9088703	1.007168	0.818734	1.048278	0.961813	0.81	0.92	AT5G11480	expressed protein
251977_at	0.0488	1.0778452	1.1099745	0.8224162	0.8449394	0.6659621	0.9171412	0.47166708	0.33942586	1.5153067	1.5063883	1.7606318	1.0790797	0.97146046	1.1568979	1.0285395	0.8051724	0.961182	0.598549	1.465352	0.990518	0.62	0.68	AT3G53250	auxin-responsive family protein similar to auxin-induced protein TGSAUR22 (GI:10185820) [Tulipa gesneriana and auxin-induced protein 6B (SP:P33083) (PIR:T10942) [Glycine max]
246069_at	0.0488	1.0606674	1.0954251	0.9023605	0.8953341	0.7072477	0.6330162	0.83040947	0.7659878	1.2835445	1.4573033	1.1487135	1.0081488	1.2613418	0.9943438	1.0056562	0.93916917	0.988397	0.734165	1.224428	1.050128	0.74	0.86	AT5G20220	zinc knuckle (CCHC-type) family protein contains Pfam domain, PF00098: Zinc knuckle
247985_at	0.0488	1.0000486	1.0088993	0.99995124	0.91345817	1.1131475	1.215957	1.1827457	1.3132476	0.96815264	0.9224285	0.9679177	0.91180956	0.9655169	1.1534976	0.9862973	1.0525341	0.980589	1.36398	0.942577	1.039461	1.39	1.10	AT5G56790	protein kinase family protein contains protein kinase domain, Pfam:PF00069
251221_at	0.0487	1.1001568	1.1609516	1.1252397	0.99869376	0.9456992	0.9455327	0.8627156	0.6201355	1.2206727	1.152419	1.1475415	0.9909131	1.0013062	0.96829206	0.99436194	1.0041192	1.09626	0.843521	1.127887	0.99202	0.77	0.88	AT3G62550	universal stress protein (USP) family protein similar to ER6 protein [Lycopersicon esculentum] GI:5669654; contains Pfam profile PF00582: universal stress protein family
[illegible]																									

258332_at	0.0363	1.0583034	1.3589739	0.9317568	1.208957	0.71847516	0.94169664	0.5204363	1.2491379	1.2525783	1.3953612	1.1741601	1.3704513	0.7651992	0.9215503	0.82676363	0.70831645	1.139498	0.857437	1.298138	0.805457	0.75	0.62	AT3G16175;AT3G16	[AT3G16175, thioesterase family protein contains Pfam profile [illegible]
261301_at	0.0363	1.1106191	1.0103644	0.964147	1.1646926	0.55150867	0.98963535	0.8484079	1.0279166	1.0240707	1.165087	1.0485014	1.0175508	0.63615406	0.85639364	0.740047	0.71875274	1.062456	0.854367	1.063802	0.737837	0.80	0.69	AT1G48570	zinc finger (Ran-binding) family protein contains Pfam domain, [illegible]
254448_at	0.0363	1.3054639	1.2336707	1.068393	1.0570648	0.9478738	0.91163814	0.7186996	0.5353404	1.2217622	1.1435766	1.1847053	0.9359948	0.9269372	0.982549	1.0174509	0.78894943	1.166148	0.778388	1.12151	0.928972	0.67	0.83	AT4G20870	fatty acid hydroxylase, putative similar to fatty acid hydroxylase [illegible]
263548_at	0.0362	1.012406	1.1019493	1.1039504	1.1409814	1.0026712	1.001202	0.98448676	0.90371376	0.9198362	1.0865352	0.99879813	1.0044849	0.8236906	0.90955424	0.9621745	0.90789825	1.089822	0.973018	1.002414	0.900829	0.89	0.90	AT2G21660	glycine-rich RNA-binding protein (GRP7) SP|Q03250 Glycine-rich R[illegible]
262370_at	0.0362	1.1870289	1.2597367	0.993632	1.0756114	0.944982	0.83953303	0.89184093	0.6674013	1.035812	0.97575206	1.1550025	1.1173167	1.0060793	0.9136921	1.0295583	0.9939207	1.129002	0.835939	1.070971	0.985813	0.74	0.92	AT1G73090	expressed protein
260741_at	0.0362	1.59581	1.5010619	3.143643	1.4727798	1.0464987	0.45665956	0.97753966	0.5029116	1.0224605	0.70992285	0.8294739	1.0637169	1.0460434	0.3999593	0.4186253	0.64627194	1.928324	0.745902	0.906394	0.627725	0.39	0.69	AT1G15040	glutamine amidotransferase-related
251985_at	0.0362	1.3342283	1.6138444	1.1954608	1.3652205	0.9096923	0.92036045	1.1872573	0.5819481	1.037717	0.9738488	1.0261512	1.1893464	0.9107926	0.91389745	0.8671691	0.85147583	1.377189	0.899815	1.056766	0.885834	0.65	0.84	AT3G53220	thioredoxin family protein low similarity to SP|P29451 Thioredoxin [illegible]
255710_at	0.0361	1.1260173	1.2238327	1.1592921	1.1291077	0.95814604	0.99716246	0.8414024	0.7831557	1.1305665	1.4198047	1.0279393	0.9431833	0.99967355	0.968086	1.0003264	0.8621708	1.159562	0.894967	1.130373	0.957564	0.77	0.85	AT4G00030	plastid-lipid associated protein PAP / fibrillin family protein conta[illegible]
267382_at	0.0361	0.93121696	0.9550244	0.81492335	1.0241036	1.1240602	1.2265917	1.0209371	0.8605271	0.87507385	0.97906286	0.9388002	0.8542566	1.3306425	1.3299345	1.1210456	1.0892448	0.931317	1.058029	0.911798	1.217717	1.14	1.34	AT2G44300	lipid transfer protein-related low similarity to lipid transfer protein [illegible]
256221_at	0.0361	1.029856	1.000189	1.015017	0.9998112	0.88775736	0.69208306	0.7312196	0.37692285	1.2102308	1.1571729	1.1147687	1.0472225	1.0139045	0.7968894	0.8283827	0.7953619	1.011218	0.671996	1.132349	0.858635	0.66	0.76	AT1G56300	DNAJ heat shock N-terminal domain-containing protein similar to [illegible]
250054_at	0.036	0.9684544	0.9089731	1.0467501	1.0537446	1.4029047	1.2101539	1.8589867	1.9986644	0.8758557	1.0001335	0.890038	0.7975195	0.93626606	0.8606632	0.9998666	1.318283	0.994481	1.617677	0.890962	1.02877	1.63	1.15	AT5G17860	cation exchanger, putative (CAX7) contains similarity to SWISS-PRO[illegible]
248132_at	0.036	0.72605616	0.6637507	0.9694453	0.8955286	0.99756336	1.1734942	1.4485046	1.256225	0.9999642	1.0700039	0.90875286	0.74563414	1.2164829	1.019799	1.0000358	1.5428559	0.813695	1.218947	0.931089	1.194793	1.50	1.28	AT5G54840	GTP-binding family protein similar to SP|P87027 Septum-promotin[illegible]
AFFX-r2-Bs-dap-5	0.036	1.1145096	1.3276485	1.3431215	1.2927148	0.39067405	0.42779708	0.335371	0.6455624	0.7957535	0.7485739	1.0934132	1.122427	1.1617076	1.3209743	0.47117102	0.9065868	1.269499	0.449851	0.940042	0.96511	0.35	1.03		
254243_at	0.036	1.0112642	1.1304606	1.2069623	0.98547244	0.9887358	0.9393552	0.9710535	1.0364832	1.4063329	1.2347206	1.0820076	1.0615785	0.87900037	0.9796886	0.7668561	0.9687076	1.08354	0.983907	1.19616	0.898563	0.91	0.75	AT4G23210	protein kinase family protein contains Pfam domain, PF00069: Pro[illegible]
264788_at	0.036	1.1764826	1.3928425	1.1527616	1.0798966	0.7505164	0.7580457	0.7887854	0.30268487	0.9201035	1.1837144	1.2970054	1.4449507	0.79162735	0.7069525	1.118634	0.78400385	1.200496	0.650017	1.211455	0.850304	0.54	0.70	AT2G17880	DNAJ heat shock protein, putative similar to J11 protein [Arabidop[illegible]
253165_at	0.036	1.5160005	1.9034011	1.2461903	1.7859511	0.66996324	1.1237708	0.56188667	0.48233533	0.6855819	0.9280523	1.1519905	1.4861535	0.71029556	0.98566866	1.0143315	0.6355186	1.612886	0.709489	1.062945	0.836454	0.44	0.79	AT4G35320	expressed protein predicted protein, Arabidopsis thaliana
251475_at	0.036	0.9114602	0.83160764	1.0286425	0.9712984	1.106368	1.2203418	1.7129279	2.0663042	0.90649086	0.9733054	0.7895711	0.85108364	1.0507227	0.91053355	1.0266947	1.1992145	0.935752	1.526485	0.880113	1.046791	1.63	1.19	AT3G59660	C2 domain-containing protein / GRAM domain-containing protein [illegible]
261624_at	0.0359	1.1629307	1.1448705	1.4140971	1.2848012	1.0394446	0.96701336	1.0647748	0.9460886	0.9079804	1.1116589	0.95527965	1.0150613	0.88756686	0.9849387	0.9502935	0.9156553	1.251675	1.00433	0.997495	0.934614	0.80	0.94	AT1G02000	NAD-dependent epimerase/dehydratase family protein similar to [illegible]
248228_at	0.0359	1.0800155	1.6431358	1.4760336	1.5754513	1.0449276	0.94135183	0.96589446	0.8297865	0.89512	0.9656143	1.0594347	1.1116091	0.9749717	0.8166632	1.0121144	0.9878856	1.443659	0.94549	1.007948	0.947909	0.65	0.94	AT5G53800	expressed protein
267231_at	0.0359	0.9071741	0.80846214	0.832402	0.79035753	0.9745573	1.1038326	0.99271905	1.250911	1.1183379	0.86629754	0.8275567	1.007281	1.0863143	1.316574	1.1758924	1.0423472	0.834599	1.080505	0.971129	1.155282	1.29	1.19	AT2G43900	endonuclease/exonuclease/phosphatase family protein belongs to [illegible]
253424_at	0.0358	1.1214164	1.245014	0.9328232	1.014944	0.83291215	0.82212913	0.63197166	0.9850561	1.4713914	1.0290195	1.0838466	1.1740633	1.0394592	0.92603314	0.84248286	0.7715803	1.078549	0.818017	1.18958	0.894889	0.76	0.75	AT4G32330	expressed protein
262780_at	0.0358	0.9176502	1.2071917	1.1479102	1.010095	0.9722964	0.6372937	0.6125159	0.62998235	1.4329877	1.0861388	1.302867	1.0629207	1.223904	0.98990494	0.9447451	0.93757415	1.070712	0.713022	1.221229	1.024032	0.67	0.84	AT1G13090	cytochrome P450 71B28, putative (CYP71B28) identical to Cytochro[illegible]
249196_at	0.0358	1.0760638	1.0385326	1.0637635	1.1440332	0.8907372	0.91136956	1.0713041	0.765257	1.1419686	1.1588689	0.9595884	1.0335629	0.96643716	0.931804	0.85060805	0.7369115	1.080603	0.909667	1.073497	0.87144	0.84	0.81	AT5G42560	abscisic acid-responsive HVA22 family protein weak similarity to S[illegible]
250290_at	0.0357	1.1601998	0.9990603	1.4359334	1.319834	0.90088063	1.0434965	1.0955396	0.8671901	1.0009398	1.0762764	0.94800943	1.0894264	0.88851595	0.8971118	0.8396804	0.86373603	1.228757	0.976777	1.028663	0.872261	0.79	0.85	AT5G13310	expressed protein
267370_at	0.0357	0.9549057	0.82009387	1.0457094	0.9301623	1.741043	1.3990732	1.5778763	0.70802236	0.683261	0.8728846	0.8055778	0.70185536	1.2151676	1.2469283	1.3868647	1.2154022	0.937564	1.356504	0.765895	1.266091	1.45	1.65	AT2G44525	expressed protein
246993_at	0.0355	0.5059184	0.5640022	0.64802593	0.6292521	0.9736552	1.3129829	0.9468518	0.77463615	0.5972185	1.3139998	1.0263447	1.07847	1.1227837	1.5229964	1.191787	1.7299542	0.5868	1.002032	1.004008	1.39188	1.71	1.39	AT5G67450	zinc finger (C2H2 type) protein 1 (AZF1) identical to Cys2/His2-typ[illegible]
257315_at	0.0355	1.7823961	1.7963738	2.8597505	2.895858	1.069095	0.82132626	1.5886667	1.9134756	0.83643085	0.6695347	0.9305906	1.5548061	0.47589016	0.31949344	0.6615439	0.59298426	2.333595	1.34822	0.897841	0.512478	0.58	0.57	AT3G30775	proline oxidase, mitochondrial / osmotic stress-responsive proline [illegible]
254597_at	0.0355	1.0144322	2.0149503	1.6462617	2.2187452	0.89123225	0.9410834	1.2056975	0.7113283	0.98556787	1.020404	1.2519033	0.9175936	0.83565223	0.9265811	0.7513342	0.8762259	1.723597	0.937335	1.068867	0.847448	0.54	0.79	AT4G18980	expressed protein contains Pfam profile PF04520: Protein of unkno[illegible]
263589_at	0.0354	1.2198926	1.0664377	1.0665148	1.0944269	0.9336271	0.8231697	1.0387458	0.73564893	1.1276256	1.1072441	0.9754753	0.9971375	1.0028626	0.9591277	0.8807479	0.80783564	1.111818	0.882798	1.051871	0.912643	0.79	0.87	AT2G25280	expressed protein
247136_at	0.0354	1.0417507	1.7865826	1.8009459	1.7641236	1.1267533	0.7862956	1.3754861	0.6909639	1.034618	1.2587211	0.8295004	0.96538204	0.8739515	0.5575483	0.50491005	0.64147407	1.598351	0.994875	1.022055	0.644471	0.62	0.63	AT5G66170	senescence-associated family protein contains similarity to ketoco[illegible]
253475_at	0.0354	0.99672663	0.8404044	0.7506757	0.854542	1.2578558	1.0464699	1.5591346	1.4526548	1.0032734	1.0361592	0.7985927	0.8679166	1.1720552	1.0760756	0.8759236	0.94324255	0.860587	1.329029	0.926485	1.016824	1.54	1.10	AT4G32290	expressed protein contains Pfam profile PF03267: Arabidopsis prot[illegible]
259005_at	0.0353	1.3189168	1.2586037	1.0712347	1.4068645	1.6791755	1.4814911	1.6189996	1.8246009	0.6019619	0.7768006	0.6967733	0.6437507	0.64073706	0.8743834	0.92876536	0.9197702	1.263905	1.651067	0.679822	0.840914	1.31	1.24	AT3G01930;AT3G01	[AT3G01930, nodulin family protein similar to nodulin-like protein [illegible]
251338_at	0.0352	0.995138	1.0093844	1.2318015	1.1101131	1.0678836	0.97389996	0.9364091	0.90984476	1.1591812	1.0707289	1.004862	1.1389644	0.96566886	0.8698931	0.9696718	0.8777992	1.086609	0.972032	1.093434	0.920758	0.89	0.84	AT3G60600	vesicle-associated membrane protein, putative / VAMP, putative si[illegible]
256678_at	0.0352	1.0810649	1.0980625	1.1034683	1.0206859	0.97951406	0.9147618	0.90041864	0.7791966	0.9339617	1.0897106	1.1899526	1.0837	0.90814835	1.0576972	0.9487541	0.9246993	1.07577	0.893473	1.074331	0.959825	0.83	0.89	AT3G52380	33 kDa ribonucleoprotein, chloroplast, putative / RNA-binding prot[illegible]
262947_at	0.0352	1.225536	1.1847936	1.3453904	1.5523801	1.0629996	0.9740802	1.150535	0.44897527	0.94773895	0.99882996	1.0011699	1.075102	0.6450755	0.6419925	0.73751724	0.549074	1.327025	0.909148	1.00571	0.643415	0.69	0.64	AT1G75750	gibberellin-regulated protein 1 (GASA1) / gibberellin-responsive pr[illegible]
245684_at	0.0352	1.0524	0.9629132	1.1253865	1.1389698	0.9852423	0.9044009	0.9999842	0.97074026	1.1529409	1.0409559	1.0396973	1.0396302	1.0000159	0.98743683	0.92419386	0.92525823	1.069917	0.965092	1.068306	0.959226	0.90	0.90	AT5G22000	zinc finger (C3HC4-type RING finger) family protein contains Pfam [illegible]
258948_at	0.0352	1.1470469	1.0765966	1.1155225	1.2610135	1.0767967	1.028078	0.9714074	0.99553704	1.1776726	0.99323595	1.004463	0.973808	0.92391986	0.9478092	0.9409784	0.9059495	1.150045	1.017955	1.037295	0.929664	0.89	0.90	AT3G01910	sulfite oxidase, putative similar to sulfite oxidase GB:3212610 SP|[illegible]
265244_at	0.0352	0.95885754	1.0745068	0.9471457	0.72876424	1.4520822	0.97636336	1.0236368	1.8346238	0.86635137	0.8481447	0.8756216	0.9532908	1.2407123	1.2606984	1.1365147	1.2793994	0.927319	1.321677	0.885852	1.229331	1.43	1.39	AT2G43020	amine oxidase family protein similar to polyamine oxidase SP:O64[illegible]
260015_at	0.0352	0.76112527	0.7026779	1.22843	0.9181076	1.2029452	1.0531601	1.923565	1.0624653	0.7595068	0.3933421	0.6082046	0.7882946	1.9486672	0.96971196	1.0302882	1.9846941	0.902585	1.310534	0.637337	1.48334	1.45	2.33	AT1G67980	caffeoyl-CoA 3-O-methyltransferase, putative similar to GI:296035[illegible]
257990_at	0.0351	1.1934899	1.1732129	1.3554504	1.2697729	1.1491425	1.0245408	1.0171325	0.91631913	0.78312004	1.0212252	0.86590004	0.98286754	0.7326967	0.8205603	0.8500505	0.7485815	1.247982	1.026784	0.913278	0.787972	0.82	0.86	AT3G19860	basic helix-loop-helix (bHLH) family protein contains Pfam profile: [illegible]
267468_at	0.035	1.1250952	1.0792341	1.0898422	0.9941299	0.78559273	0.8395476	1.0058701	0.93114924	1.2448858	1.0982759	0.985622	1.1171572	1.0797312	0.9306605	0.95479393	0.9165558	1.072075	0.89054	1.111485	0.970435	0.83	0.87	AT2G30530	expressed protein
266010_at	0.035	0.3313188	0.28732437	0.5366814	1.5141685	2.0745296	4.7518477	1.0569736	1.7451124	0.6316061	0.42100227	0.79082984	0.96980774	3.5993032	1.0137188	0.9862811	3.2388744	0.667373	2.407116	0.703311	2.209544	3.61	3.14	AT2G37430	zinc finger (C2H2 type) family protein (ZAT11) contains Pfam doma[illegible]
251225_at	0.0349	0.9973558	1.2574012	1.0026643	1.072665	0.83291113	0.8578826	0.73810893	1.2789575	1.2062086	1.1011642	1.048364	1.1263982	0.7646918	0.70555604	0.8936318	0.74834514	1.082517	0.926841	1.120534	0.778056	0.86	0.69	AT3G62660	glycosyl transferase family 8 protein low similarity to glycosyl trans[illegible]
265458_at	0.0348	0.8502267	1.0145571	0.8525606	0.98544294	1.6616564	1.4549184	1.5546666	0.72497267	0.77273685	0.9586708	0.7603136	0.84115726	1.5535903	1.2030979	1.2909317	1.2897564	0.925697	1.349054	0.83322	1.334344	1.46	1.60	AT2G46580	pyridoxine 5'-phosphate oxidase-related similar to pyridoxine 5'-ph[illegible]
245198_at	0.0347	1.0988848	1.0598199	1.0472234	1.0507424	0.8832313	0.79072005	0.7221386	0.51846635	1.2044499	1.1375067	1.1128113	0.92629385	1.1207179	0.95277655	0.87508607	0.8876434	1.064168	0.728639	1.095265	0.959056	0.68	0.88	AT1G67700	expressed protein
250117_at	0.0347	1.1040413	1.2202218	1.2495438	1.3147439	0.9962099	0.77365744	0.86681014	0.5923802	1.0037901	0.92593986	1.0333108	1.0178409	0.7460524	0.97196174	1.0576911	0.88067484	1.222138	0.807264	1.01272	0.914095	0.66	0.90	AT5G16440	isopentenyl-diphosphate delta-isomerase I / isopentenyl diphosph[illegible]
245499_at	0.0347	1.0139521	0.80565506	0.8463745	0.8126559	1.2206765	1.1628202	1.1319768	1.720112	0.8309978	1.0829612	0.8305953	0.91514146	0.93051404	0.99038805	1.0980936	1.0096109	0.869659	1.308896	0.914924	1.007152	1.51	1.10	AT4G16480	sugar transporter family protein similar to SP|Q96QE2 Proton myo[illegible]
248943_s_at	0.0347	1.0860759	0.8739841	1.0720673	1.0164411	0.96651757	0.7243885	0.9410331	0.7787915	1.3783717	1.0651052	1.1534462	1.2061001	0.9918608	0.8412183	1.0081393	0.74733686	1.012142	0.852683	1.200756	0.897139	0.84	0.75	AT5G45490	disease resistance protein-related contains Pfam profile, PF00931[illegible]
250385_at	0.0345	1.0229028	1.2213564	2.0543609	1.3820208	0.7855454	0.71872133	1.0717207	1.2496274	1.0278978	0.970962	0.95348203	1.04653	0.7070991	0.6158671	0.62488556	0.7743892	1.42016	0.956404	1.001252	0.68056	0.67	0.68	AT5G11520	aspartate aminotransferase, chloroplast / transaminase A (ASP3) ([illegible]
263598_at	0.0345	1.2762982	1.2412518	1.3178355	1.213397	0.9781528	1.0218472	0.9273093	1.0954937	0.8617331	0.8016073	1.091237	1.0379078	0.75694317	0.84124535	0.91746926	0.8264949	1.262196	1.005701	0.948121	0.835538	0.80	0.88	AT2G01850	xyloglucan:xyloglucosyl transferase / xyloglucan endotransglycosyl[illegible]
AFFX-r2-Bs-lys-M	0.0345	1.0624002	1.0030195	1.165323	1.3278886	0.4302443	0.48207155	0.6698181	0.4424773	0.7965587	1.1849946	1.3915769	0.99698055	1.2618346	1.320436	0.62830424	0.83082765	1.139658	0.506153	1.092528	1.010351	0.44	0.92		
250197_at	0.0344	1.0632194	1.1850938	1.1340983	1.0886883	0.9950656	0.9416873	1.0049344	1.200909	1.0137572	1.0624662	0.92039716	0.993318	0.7655982	0.85929036	0.80039847	0.8689188	1.117775	1.035649	0.997484	0.823551	0.93	0.83	AT5G14590	isocitrate dehydrogenase, putative / NADP+ isocitrate dehydrogen[illegible]
248290_at	0.0343	0.9037439	0.9629124	1.0407366	1.0953759	1.2352482	1.1801407	1.3108779	1.5841961	0.89626926	1.0287058	0.9013566	0.91412115	0.97589564	0.94777584	0.96797101	1.0241042	1.000692	1.327616	0.935113	0.977121	1.33	1.04	AT5G53010	calcium-transporting ATPase, putative
255590_at	0.0343	0.9924457	1.3764153	1.2968687	1.371487	1.1193526	0.86044717	0.9577128	1.0445232	1.0075543	1.12676	1.0369114	0.9910683	0.8516817	0.8025817	0.89332753	0.95488995	1.259304	0.995509	1.040574	0.87562	0.79	0.84	AT4G01610	cathepsin B-like cysteine protease, putative similar to cathepsin B-[illegible]
253124_at	0.0343	0.7778221	0.65725046	0.78518146	0.99673843	1.0630674	1.3722992	0.88450193	2.035229	0.7703946	0.8366084	1.009811	0.8729105	1.2185582	1.5558383	1.0032616	1.5546105	0.804248	1.338774	0.872431	1.333067	1.66	1.53	AT4G36030	armadillo/beta-catenin repeat family protein contains Pfam profile[illegible]
263702_at	0.0342	1.0932517	1.0825402	0.9820396	1.053784	0.8888216	0.94962174	1.1039515	0.94232184	1.0545666	1.3084409	1.0740112	1.0178903	0.85244936	0.8350061	0.8930815	0.9525812	1.052921	0.971179	1.113727	0.88328	0.92	0.79	AT1G31240	expressed protein identical to hypothetical protein GB:AAD21690 [illegible]
249454_at	0.0342	0.8232273	1.0962769	3.7293854	2.69273	0.59608364	0.3545056	1.1752511	0.33907026	1.4453034	1.580395	1.1286482	1.3138944	0.9037231	0.5239192	0.64530987	0.80293036	2.085405	0.616228	1.36706	0.718971	0.30	0.53	AT5G39520	expressed protein predicted protein, Synechocystis sp., PIR:S7152[illegible]
253351_at	0.0342	1.293464	1.2476027	1.2658533	0.94748145	0.8214905	0.8036442	0.87368804	1.025479	1.3922095	0.94986856	1.0709692	1.1145254	0.9868742	1.0131259	0.8249757	0.8664611	1.1886	0.881075	1.131893	0.907359	0.74	0.80	AT4G33700	CBS domain-containing protein contains Pfam profiles PF00571: CB[illegible]
256249_at	0.0342	1.0827999	1.1773163	1.1817392	0.9982424	0.92300797	0.879998	1.0408188	0.77886313	1.0880518	0.97504514	1.1339278	1.0031643	1.0017576	0.8834534	0.9329669	0.93139607	1.110024	0.905672	1.050047	0.937393	0.82	0.89	AT3G11270	26S proteasome non-ATPase regulatory subunit 7, putative / 26S p[illegible]

[illegible]

260172_s_at	0.0234	1.1224836	1.359894	1.0442814	1.041288	0.94481397	0.7709459	0.99775803	0.80535245	1.002242	1.2469333	1.1467624	1.0578582	0.99416083	0.97716886	0.90697515	0.8891553	1.141987	0.879718	1.113449	0.941865	0.77	0.85	AT5G10330;AT1G71	[AT5G10330, histidinol-phosphate aminotransferase, putative [illegible]
265852_at	0.0233	0.9167957	0.8740815	0.95626813	1.1742579	1.1756411	1.4876212	1.4253405	1.0178741	0.7358671	0.8275866	0.9062261	0.74801075	1.2191801	1.1067233	0.98212576	1.6796912	0.980351	1.276619	0.804423	1.24693	1.30	1.55	AT2G42350	zinc finger (C3HC4-type RING finger) family protein contains Pfam [illegible]
250233_at	0.0233	1.1357732	1.5460036	0.9632261	1.2293988	0.89837736	0.84394246	0.84707874	0.9224615	1.101251	1.2748672	1.14421	1.8323609	0.9691157	0.88300824	1.0308841	0.7817732	1.2186	0.877965	1.338172	0.916195	0.72	0.68	AT5G13460	calmodulin-binding family protein low similarity to SF16 protein [illegible]
262432_at	0.0233	0.8546114	0.78885764	1.119496	0.7558363	1.3379517	1.1007708	1.3411267	1.8833627	0.9080341	0.8238917	0.85341257	0.8305755	1.0919658	0.8420381	1.1278816	1.3381023	0.879701	1.415803	0.853978	1.098472	1.61	1.29	AT1G47530	ripening-responsive protein, putative similar to ripening regulated [illegible]
246343_at	0.0231	1.0773441	1.0072365	1.0338055	1.0126528	0.8150507	0.79990757	0.99276346	1.033664	1.1562299	1.0494273	0.9867163	1.133064	0.889597	0.875889	0.92396426	0.8911769	1.03276	0.910346	1.081359	0.895157	0.88	0.83	AT3G56720	expressed protein
246374_at	0.0231	1.0888147	0.98358846	1.1949134	1.1772543	0.97470665	0.9884625	0.9627249	0.9303906	1.0257807	1.0916972	1.0346688	1.0335014	0.8326103	1.0115377	0.91859716	0.9527167	1.111168	0.964071	1.046412	0.928865	0.87	0.89	AT1G51840	protein kinase-related contains similarity to light repressible recep[illegible]
261763_at	0.0231	0.9158749	0.8356483	1.1476299	1.0841249	1.1746114	1.4261147	1.8002893	2.1337466	0.8563094	0.60342646	0.7976926	0.66826725	1.297978	0.8820557	0.83168256	1.1901889	0.99582	1.633691	0.731424	1.050476	1.64	1.44	AT1G15520	ABC transporter family protein similar to ABC1 protein GI:1433111 [illegible]
259049_at	0.0231	1.1671911	1.0554789	1.1333947	1.035693	0.9492105	0.9676176	0.8905028	1.0323825	1.1544243	1.0530738	1.0467238	0.9052865	0.8497887	0.90414774	0.93857795	0.78598344	1.097939	0.959928	1.039877	0.869624	0.87	0.84	AT3G03370	expressed protein
254596_at	0.0229	1.0531671	1.1187397	1.0050794	0.9949205	0.98596036	0.9220601	0.9444537	0.83093756	1.120338	1.0143616	1.1413996	1.1093326	0.96788098	1.0299041	0.94160175	0.85178494	1.042977	0.920853	1.092358	0.947795	0.88	0.87	AT4G18975	pentatricopeptide (PPR) repeat-containing protein contains Pfam p[illegible]
254037_at	0.0229	1.290922	1.0567211	1.1476643	1.0941346	0.95626134	0.9357736	0.92086315	0.77716553	1.104897	1.2267241	1.0501554	0.91936475	0.8466273	1.0437387	0.8807254	0.8643606	1.147361	0.897516	1.075285	0.908858	0.78	0.85	AT4G25760	expressed protein
266034_at	0.0229	0.9192704	1.0328736	0.81963295	0.8003282	1.0749768	1.1033772	1.2892898	1.0558792	0.9471702	0.9581387	0.9461466	0.8631651	1.2701395	0.96712625	1.048431	1.0915544	0.893026	1.130881	0.928655	1.094313	1.27	1.18	AT2G06005	expressed protein
260676_at	0.0229	0.7312399	0.77378017	0.51843446	0.5144037	0.8716378	0.92926544	0.74956673	0.9826459	1.0226842	1.1429799	1.017354	1.3224484	1.3965783	1.3772972	1.6317482	1.3668984	0.634465	0.883279	1.126367	1.443131	1.39	1.28	AT1G19450	integral membrane protein, putative / sugar transporter family pro[illegible]
251150_at	0.0229	1.1730133	1.2941338	1.1819371	1.2700782	0.93274	0.96725225	0.766662	0.53023684	1.0280812	1.2837932	1.0452156	1.1460314	0.9172616	0.9644324	0.9719189	0.6785135	1.229791	0.799223	1.12578	0.883027	0.65	0.78	AT3G63120	cyclin family protein similar to cyclin 2 [Trypanosoma brucei] GI:7[illegible]
245286_at	0.0228	1.0716232	1.151714	1.3069416	1.0405613	0.999298	0.85505	0.9430124	0.9226074	1.1675448	1.0007019	1.093214	0.9691449	1.0045979	0.79914415	0.92248785	0.89454424	1.14271	0.929992	1.057651	0.905194	0.81	0.86	AT4G14880	cysteine synthase / O-acetylserine (thiol)-lyase / O-acetylserine sul[illegible]
260580_at	0.0228	0.8149176	0.79844105	0.9713033	0.76205355	1.3578362	1.0286965	1.0436015	2.2505808	0.92031115	0.83519316	0.8111016	0.7812537	1.1205046	1.1668267	1.1124653	1.2104301	0.836679	1.420179	0.836965	1.152557	1.70	1.38	AT2G47390	expressed protein
251057_at	0.0228	1.1344565	1.3551192	1.4339094	1.4335854	0.9470393	1.0074908	1.1298177	1.1298454	0.9708379	1.0346917	0.98026097	0.9925093	0.9612414	0.96181446	0.8168483	0.8329644	1.339268	1.053548	0.994575	0.893217	0.79	0.90	AT5G01780	oxidoreductase, 2OG-Fe(II) oxygenase family protein low similarity [illegible]
265193_at	0.0228	1.1320413	1.1596359	0.9856484	1.062316	0.7414954	0.8665036	0.73861927	0.36895064	1.3676416	1.2175987	1.2576519	1.2427329	1.0143516	0.90914655	0.97785085	0.8251148	1.08491	0.678892	1.271406	0.931616	0.63	0.73	AT1G05070	expressed protein
257203_at	0.0228	1.7682333	2.3449132	1.3697592	2.2888181	1.0906626	1.1242899	0.8296377	0.6207123	0.8362736	0.8729829	1.2923149	1.3180766	0.897073	0.9093373	0.9026524	0.5978653	1.942931	0.916326	1.079912	0.826732	0.47	0.77	AT3G23730	xyloglucan:xyloglucosyl transferase, putative / xyloglucan endotran[illegible]
250703_at	0.0228	1.1332803	1.1064178	1.2718642	0.9992344	0.93253136	0.8694127	0.95041597	0.7189621	1.2376904	1.0381123	1.0007656	1.2050743	1.0813264	0.8921516	0.8703732	0.85749334	1.127699	0.867831	1.120411	0.925336	0.77	0.83	AT5G06360	ribosomal protein S8e family protein contains Pfam profile PF0120[illegible]
247339_at	0.0227	1.4032184	1.1002897	1.023032	1.2463342	0.8037141	1.0517212	0.9030161	0.62519723	1.1825436	0.97696817	1.0943356	1.1815329	0.93606645	0.8765655	0.9031981	0.83695436	1.193219	0.845912	1.108845	0.887221	0.71	0.80	AT5G63690	hypothetical protein
262032_at	0.0227	1.1157756	1.0017855	1.075807	1.1202824	1.0601861	0.9982143	0.93763894	0.8649403	1.0097746	1.1613802	1.1638335	0.95983785	0.8384145	0.92250526	0.8708044	0.80232966	1.078413	0.965245	1.098707	0.858513	0.90	0.78	AT1G37150	holocarboxylase synthetase 2 (HCS2.d) identical to holocarboxylase[illegible]
254478_at	0.0226	1.2253014	1.4308444	1.35795	1.2348219	0.9779226	0.99426967	1.1856668	0.7884258	0.9446586	1.1365954	1.0034184	1.1471101	0.99658155	0.8094446	0.90502936	0.8580626	1.312229	0.986571	1.057946	0.89228	0.75	0.84	AT4G20330	transcription initiation factor-related contains weak similarity to Tr[illegible]
262413_at	0.0226	1.1066232	1.1360874	0.99567825	1.0043218	0.92689425	0.7913849	0.7335248	0.545592	1.2423294	1.1334803	1.1209641	1.033652	1.046213	0.97854966	0.88262737	0.9520415	1.060678	0.749333	1.132591	0.964858	0.71	0.85	AT1G34780	protein disulfide isomerase-related contains weak similarity to Pfa[illegible]
252394_at	0.0226	1.0850568	1.129285	1.2417793	1.1247873	0.9600372	0.98029685	0.7484199	0.9445648	1.0048504	1.0309813	1.0685081	1.232065	0.90386087	0.81824285	0.99514973	0.9927056	1.145227	0.90833	1.084101	0.92749	0.79	0.86	AT3G47940	DNAJ heat shock protein, putative similar to SP[O89114 DnaJ homo[illegible]
252971_at	0.0225	1.1954718	1.2001941	1.1056585	1.1791384	0.8920063	0.9491432	0.5944788	1.1028524	1.2198749	1.0967075	1.0478991	0.979475	0.7400696	0.89727134	0.91136575	0.8273757	1.170116	0.86412	1.085907	0.844021	0.74	0.78	AT4G38770	proline-rich family protein (PRP4) similar to proline-rich protein [A[illegible]
250124_at	0.0225	1.262925	1.2363943	1.3767229	1.3564922	1.039312	0.96068805	0.7582727	0.5925949	0.77980113	1.1426864	1.1377398	1.0666838	0.75012416	0.78356993	0.96016854	0.7830015	1.308134	0.837717	1.031728	0.819216	0.64	0.79	AT5G16480	tyrosine specific protein phosphatase family protein contains tyros[illegible]
248757_at	0.0225	1.0695078	1.2066097	1.238938	1.1967368	1.0012096	0.9162017	1.0088586	0.8959435	1.0847237	0.99879026	0.9052602	1.0803936	0.9914406	0.85610485	0.98820806	0.8697558	1.177948	0.955553	1.017292	0.926377	0.81	0.91	AT5G47680	expressed protein contains Pfam profile PF04243: Protein of unkno[illegible]
253617_at	0.0225	1.0661669	0.84896183	0.80543786	0.93383306	0.6289934	0.67464757	0.47991097	0.32197192	1.8629689	1.9634659	1.5258602	1.8732715	1.5376195	1.0933628	1.3374933	0.68284535	0.9136	0.526381	1.806392	1.16283	0.58	0.64	AT4G30410	expressed protein similar to cDNA bHLH transcription factor (bHLH[illegible]
262478_at	0.0225	1.0689156	0.8581945	1.029446	1.0608956	0.6739265	0.7949434	0.8971171	0.9437979	1.7986633	1.7915689	1.4256517	1.127704	1.1215558	0.97055393	0.87047374	0.96703863	1.004363	0.827446	1.535897	0.982406	0.82	0.64	AT1G11170	expressed protein contains Pfam profile PF05212: Protein of unkno[illegible]
265854_at	0.0225	0.92090684	1.0667176	0.8516514	0.8161809	0.9535185	1.1420141	1.2824935	1.3819281	0.96291393	0.982521	0.89562696	0.93927944	1.3669341	1.017479	1.2738056	1.1599061	0.913864	1.189989	0.945085	1.204531	1.30	1.27	AT2G42370	expressed protein
248276_at	0.0224	0.7292683	0.9229195	1.1038249	1.0770806	1.1928939	1.6148651	1.5305325	2.5778947	0.5450072	0.62097645	0.6585717	0.6742396	0.67841697	0.85701036	1.2456092	1.2706758	0.958273	1.729047	0.624699	1.012928	1.80	1.62	AT5G53550	transporter, putative similar to iron-phytosiderophore transporter [illegible]
257722_at	0.0224	0.74375695	0.7681916	0.7449925	0.5716522	1.0185107	0.9599036	0.8831475	1.285484	1.3008008	1.110412	0.9814891	0.91988003	1.3523071	1.3775312	1.2586097	1.1612507	0.707148	1.036761	1.078145	1.287425	1.47	1.19	AT3G18490	aspartyl protease family protein contains Pfam domain, PF00026: e[illegible]
260743_at	0.0223	1.645471	1.3585302	2.6237223	1.5165801	0.9807337	0.6244526	0.9862368	0.77981484	1.0305268	0.79603416	1.0137631	1.1574981	1.1198398	0.62633836	0.6235114	0.7753195	1.786076	0.842809	0.999456	0.786252	0.47	0.79	AT1G15040;AT1G15	[AT1G15040, glutamine amidotransferase-related];[AT1G15030, e[illegible]
250315_at	0.0223	1.0737772	1.1554055	0.9987421	0.9381052	0.9077308	0.73164105	0.85970205	0.76040894	1.4007512	1.2043074	1.1204231	1.0780263	1.150395	0.9402001	0.97030073	1.0012579	1.041508	0.814871	1.200877	1.015989	0.78	0.85	AT5G12130	integral membrane TerC family protein contains Pfam profile PF03[illegible]
244943_at	0.0223	0.99822676	1.4310827	1.2146424	1.1968511	1.0017734	0.8865069	0.8389515	1.0338012	1.3665016	0.9414228	1.1551336	1.113523	0.96259284	0.8000651	0.8255505	0.89710805	1.210201	0.940258	1.144145	0.871329	0.78	0.76	ATMG00070	nad9 NADH dehydrogenase subunit 9
264140_at	0.0223	1.1618872	1.061377	1.2899346	1.3217108	1.0025884	0.8829464	0.9131237	0.61404854	1.2325282	1.0567068	1.2993088	0.9974117	0.7307371	0.91565984	0.9833017	0.9210962	1.208727	0.853177	1.146489	0.887699	0.71	0.77	AT1G79210	20S proteasome alpha subunit B, putative nearly identical to SP[O[illegible]
248802_at	0.0223	1.1891948	1.2618113	0.9804057	1.0529402	0.98593897	0.8818017	0.88070744	0.8025656	1.0548097	1.0140611	1.04686	1.0212494	1.0185033	0.94922775	0.9345622	0.91643715	1.121088	0.887753	1.033702	0.954683	0.79	0.92	AT5G47435	formyltetrahydrofolate deformylase, putative similar to formyltetra[illegible]
266205_s_at	0.0223	0.9913004	1.2237402	1.2239087	1.1290234	1.1431493	0.8555143	0.81968415	1.0386014	1.0713055	1.0086998	0.98121613	1.0138041	0.8554035	0.7611778	0.8264201	0.7864704	1.141993	0.964237	1.018756	0.807368	0.84	0.79	AT2G27860	expressed protein
245148_at	0.0223	0.7399956	0.83431876	1.2349889	0.7562455	1.5880463	0.98075587	1.5179601	1.1916056	0.8680483	0.6270194	0.6011662	0.80904496	1.0723407	1.019244	1.0707593	1.5769974	0.891387	1.319592	0.72632	1.184835	1.48	1.63	AT2G45220	pectinesterase family protein contains Pfam profile: PF01095 pecti[illegible]
251074_at	0.0223	1.1864144	1.104319	1.4388652	1.3259469	0.9674148	1.0029833	1.0422431	0.74368274	1.1592002	1.1993409	0.99701655	0.97871315	0.8902151	0.98945624	0.90786576	0.791989	1.263886	0.939081	1.083568	0.894882	0.74	0.83	AT5G01800	saposin B domain-containing protein contains Pfam profiles: PF000[illegible]
265536_at	0.0223	0.6728369	0.45972884	0.8947856	0.7348134	1.5359905	1.2655592	1.3079695	1.0080268	1.2675399	0.8907835	0.737841	0.92238766	1.4763455	1.2937039	1.1552061	0.99197316	0.690541	1.279387	0.954638	1.229307	1.85	1.29	AT2G15880	leucine-rich repeat family protein / extensin family protein similar t[illegible]
248133_at	0.0221	1.1224797	1.0580338	1.0875896	1.2198008	0.6779264	1.0612208	0.8367726	0.7759008	0.96721107	1.0327889	1.2362568	1.0593222	0.9050364	0.8908887	0.80090684	0.96403396	1.122018	0.837955	1.073895	0.890216	0.75	0.83	AT5G54850	expressed protein
254574_at	0.0221	0.23347466	0.80725133	0.39855894	0.60611004	1.1456908	1.3385829	1.1646358	0.4613602	0.6915442	1.1288552	0.8711449	0.76040184	2.0942907	2.4302576	1.8744632	1.7808603	0.511349	1.027567	0.862987	2.044968	2.01	2.37	AT4G19430	expressed protein
248460_at	0.0221	1.402782	1.0007939	0.78278345	1.7046352	0.79670334	0.5685297	0.55602586	0.4765125	1.4573615	1.2889676	1.9937074	1.4319223	1.0946128	0.99920607	1.022491	0.89053166	0.972749	0.599443	1.54299	1.00171	0.62	0.65	AT5G50915	basic helix-loop-helix (bHLH) family protein contains Pfam profile: [illegible]
265478_at	0.0221	1.0982212	1.1806029	1.2857084	1.1830903	1.0657498	0.9853016	0.896338	0.75223523	1.114295	1.0146983	1.1387998	0.96403563	0.925586	0.92440695	0.97465765	0.89981425	1.186906	0.924906	1.057957	0.931116	0.78	0.88	AT2G15890	expressed protein
252453_at	0.0221	1.0443194	1.6515287	1.663516	1.5246346	0.9880913	0.9005295	0.97212714	0.85730195	1.0269508	1.2085699	0.95457304	1.03478	1.0119088	0.95514244	0.9848223	0.9257983	1.471	0.929512	1.056218	0.969418	0.63	0.92	AT3G47120	RNA recognition motif (RRM)-containing protein contains InterPro [illegible]
256468_at	0.0221	1.1288412	1.1895522	1.0294706	0.97052956	0.8786817	0.74010295	0.72317255	0.40275046	1.4766258	1.4067303	1.2689887	1.1970674	1.1851586	0.9479302	0.8707615	0.8916572	1.079598	0.686177	1.337353	0.973877	0.64	0.73	AT1G32550	ferredoxin family protein similar to ferredoxin from Synechocystis [illegible]
261394_at	0.0221	0.52689815	0.42831302	0.7410822	1.6411604	1.2388601	2.4955578	1.8732746	4.1916575	0.70014155	0.3107322	0.65527266	0.77602506	1.9587264	1.0206274	0.97937256	2.074465	0.834363	2.449838	0.610543	1.508298	2.94	2.47	AT1G79680	wall-associated kinase, putative similar to wall-associated kinase 2 [illegible]
260833_at	0.0221	1.0120085	0.998157	1.1405346	1.1883373	1.4249288	1.1676687	1.4633288	1.5548916	0.82797486	0.70866185	0.7590327	0.6528164	0.7544079	0.9148756	0.93193287	1.001843	1.084759	1.402704	0.737121	0.900765	1.29	1.22	AT1G06800	lipase class 3 family protein similar to DEFECTIVE IN ANTHER DEHIS[illegible]

[illegible]

262134_at	0.0118	0.9743857	1.0256144	0.847041	1.0959754	1.2692511	1.1863223	1.3052446	1.4065564	0.814805	0.6743536	0.80516523	0.9114997	0.9461408	0.87277627	1.1263725	1.0259876	0.985754	1.291844	0.801456	0.992819	1.31	1.24	AT1G77990 sulfate transporter identical to sulfate transporter [Arabidopsis thaliana] GI:1498120
246702_at	0.0118	1.2721927	1.3337551	1.7819086	1.4859021	0.99075896	0.5515755	1.009241	0.50021017	0.8055352	1.0747697	1.0661734	1.1817292	0.78406703	0.68344474	0.7227858	0.7749043	1.46844	0.762946	1.032052	0.7413	0.52	0.72	AT5G28050 cytidine/deoxycytidylate deaminase family protein similar to SP|O34598 Guanine deaminase (EC 3.5.4.3) (Guanase) (Guanine aminase) (Gua[illegible]
262247_at	0.0118	1.106609	1.123175	0.9937402	1.1382096	0.994558	0.9393712	0.87411016	0.86609083	1.0747061	1.109896	1.0653003	1.0719534	1.0053946	0.99460536	0.9440257	0.8071893	1.090433	0.918533	1.080464	0.937804	0.84	0.87	AT1G48420 desulfhydrase family similar to similar to D-cysteine desulfhydrase (EC 4.4.1.15). (Swiss-Prot:P59329) [Escherichia coli O6]; contains TIGRFAM T[illegible]
261607_at	0.0118	0.7861866	0.9474587	0.9569409	0.99888325	0.65162295	0.8844201	0.73556566	0.60727745	1.3015834	1.5441239	1.3042308	1.6409395	1.0707631	1.1676413	1.0011168	1.0476532	0.922367	0.719722	1.447719	1.071794	0.78	0.74	AT1G49660 expressed protein
245319_at	0.0118	1.0964003	1.0142984	1.0482641	0.8943618	0.66300935	0.665291	0.53572106	0.2644215	1.4825878	1.4614605	1.4470364	1.2129203	1.0431541	0.9857015	0.9543011	0.92067385	1.013331	0.532111	1.401001	0.975958	0.53	0.70	AT4G16146 expressed protein
245098_at	0.0118	0.8240163	0.8360154	1.1094553	1.0011128	1.2992605	1.2109092	1.2578417	2.176711	0.55904007	0.6041691	0.75090986	0.6945962	0.8049965	1.0774176	1.1003939	0.998887	0.94265	1.486181	0.652179	0.995424	1.58	1.53	AT2G40940 ethylene response sensor / ethylene-responsive sensor (ERS) identical to ethylene response sensor (ERS) [Arabidopsis thaliana] GI:1046225
248037_at	0.0117	0.8953533	0.7954867	1.0612357	0.9785838	1.4625825	1.5179969	2.1322773	3.477324	0.73751163	0.63995755	0.6694183	0.5862362	0.66811395	1.0340538	1.0214163	1.1154939	0.932665	2.147545	0.658281	0.959769	2.30	1.46	AT5G55930;AT5G55 [AT5G55930, oligopeptide transporter OPT family protein similar to SP|P40900 Sexual differentiation process protein isp4 (Schizosaccharomy[illegible]
258498_at	0.0117	1.3941215	1.4844607	1.3756728	1.0363109	1.0194863	0.76780534	0.9293981	0.76958436	1.047637	1.2917765	1.1188301	0.9429095	0.98051375	0.922752	0.88602567	0.85381323	1.322641	0.871569	1.100288	0.910776	0.66	0.83	AT3G02480 ABA-responsive protein-related similar to ABA-inducible protein [Fagus sylvatica] GI:3901016, cold-induced protein kin1 [Brassica napus] GI:[illegible]
252432_at	0.0117	1.2570156	1.0871171	1.101704	0.9232206	0.7689879	1.0168505	0.9831494	0.9006841	1.4752252	1.1960664	1.2954364	1.085182	0.84603435	0.90931636	0.9252248	0.7465896	1.092264	0.917418	1.262978	0.856791	0.84	0.68	AT3G47680 expressed protein contains similarity to hypothetical proteins of [Arabidopsis thaliana]
267586_at	0.0116	0.8167164	0.938418	0.7822842	0.7786219	1.2530282	1.1941582	1.0392638	0.7157199	0.93058956	1.0419996	0.96073604	0.8761646	1.6281936	1.757418	1.3621393	1.4422446	0.82901	1.050543	0.952372	1.547499	1.27	1.62	AT2G41950 expressed protein
251013_at	0.0115	0.8994408	0.87361556	0.7204943	0.98115003	0.5202774	0.7353559	0.4722724	0.4362738	1.7890397	1.7226207	1.7165474	1.9660019	1.4121144	1.6357421	1.3568515	1.01885	0.868675	0.541045	1.798552	1.35589	0.62	0.75	AT5G02540 short-chain dehydrogenase/reductase (SDR) family protein contains INTERPRO family IPR002198 Short-chain dehydrogenase/reductase (SDR) [illegible]
251826_at	0.0115	0.9868425	0.824935	0.8352622	0.7598099	1.3462628	1.3512217	1.1721115	1.9012455	1.0275061	0.75310075	0.8992311	0.7822767	1.131749	1.0131575	0.88824165	1.0211954	0.851712	1.44271	0.865529	1.013586	1.69	1.17	AT3G55110 ABC transporter family protein ATP-binding cassette-sub-family G-member 2, Mus musculus, EMBL:AF140218
263963_at	0.0114	1.0799131	0.67741483	0.7049483	0.57282364	1.4637582	1.5031086	1.5905921	0.78484464	0.92008686	0.7013086	0.7667607	0.7210337	1.4454851	1.3555139	1.1475017	1.2123624	0.758775	1.335576	0.777297	1.290216	1.76	1.66	AT2G36080 DNA-binding protein, putative strong similarity to DNA-binding proteins from [Arabidopsis thaliana] RAV1 GI:3868857, RAV2 GI:3868859; con[illegible]
257466_at	0.0113	1.1093223	1.0618763	1.2364134	1.1886686	0.77079177	0.9955184	0.9998303	0.721692	1.0001696	1.2309221	1.1445386	1.28332	0.7695872	0.83643365	0.9324038	0.99084103	1.14907	0.871958	1.164738	0.882316	0.76	0.76	AT1G62840 expressed protein
262657_at	0.0113	1.2901694	1.2586334	1.0298595	1.072989	0.7587674	0.86233383	0.7480399	0.5673873	1.1916444	0.9435299	1.041568	1.0207167	1.0098295	0.99017066	0.7633029	0.82249624	1.162913	0.734132	1.049365	0.89645	0.63	0.85	AT1G14210 ribonuclease T2 family protein contains similarity to RNase GI:7768564 from [Nicotiana tabacum]; contains Pfam profile PF00445: Ribonuclea[illegible]
247524_at	0.0113	1.4926808	1.3884298	1.4390717	1.2925448	0.9804168	0.6743283	1.0195833	0.57538605	0.7887756	1.2208235	1.4262493	1.4147043	0.7809281	0.7546199	0.90618235	0.7979358	1.403182	0.812429	1.212638	0.809917	0.58	0.67	AT5G61440 thioredoxin family protein low similarity to thioredoxin [Callithrix jacchus] GI:13560979; contains Pfam profile: PF00085 Thioredoxin
252980_at	0.0113	1.1520094	1.1569886	1.1142951	1.030497	1.1347688	0.96077824	0.9709023	1.0199736	0.98002625	1.0496173	1.0871904	0.97764236	0.8452259	0.86028314	0.8993441	0.83186555	1.113448	1.021606	1.023619	0.85918	0.92	0.84	AT4G38040 exostosin family protein contains Pfam profile: PF03016 Exostosin family
255607_at	0.0113	0.8761078	0.97357893	0.76597995	0.8277602	0.7555395	0.68728864	0.63673115	0.73601526	1.4666364	1.2687845	1.3793919	1.4254329	1.2968355	1.036421	1.116053	1.0317482	0.860857	0.703894	1.385061	1.117764	0.82	0.81	AT4G01130 acetylesterase, putative similar to lanatoside 15'-O-acetylesterase [Digitalis lanata] GI:3688284; contains InterPro Entry IPR001087 Lipolytic en[illegible]
252820_at	0.0113	0.88171285	0.7568189	0.6745234	0.9174528	1.0164956	1.0864489	1.2957164	1.7619839	0.945542	0.9835044	0.81071055	0.97061574	1.148053	1.1513342	1.3244789	1.1856451	0.807627	1.290161	0.927593	1.202378	1.60	1.30	AT3G42640 ATPase, plasma membrane-type, putative / proton pump, putative strong similarity to P-type H+-ATPase from [Lycopersicon esculentum] GI:[illegible]
250843_at	0.0112	1.207038	1.154295	1.1209905	1.1150768	1.0361044	0.9916552	0.8657738	0.8853924	1.0321522	1.1948742	1.0083448	0.98310465	0.80009717	0.8855279	0.98665386	0.90845424	1.14935	0.944731	1.054619	0.895183	0.82	0.85	AT5G04430 KH domain-containing protein NOVA, putative astrocytic NOVA-like RNA-binding protein, Homo sapiens, U70477
254690_at	0.0112	1.0646881	1.1301203	1.3521203	1.1528447	1.0223694	0.80914754	0.93585175	0.9852155	1.1717567	1.1115315	1.0115784	0.9884216	0.9504223	0.90420824	0.8087234	0.8631036	1.174943	0.938146	1.070822	0.881614	0.80	0.82	AT4G17830 peptidase M20/M25/M40 family protein similar to acetylornithine deacetylase [Acetylornithinase, AO; N-acetylornithinase, NAO] (Dictyostel[illegible]
256089_at	0.0112	0.99788743	0.9729789	1.0021125	0.95349294	0.8043079	0.8690261	0.77423507	0.6787767	1.175959	1.0411537	1.1881014	1.0894371	1.0145355	0.8637445	1.0227897	1.0619781	0.981618	0.781586	1.123663	0.990762	0.80	0.88	AT1G20830 expressed protein
266860_at	0.0112	0.89644176	0.7926613	0.7761109	0.7149606	0.97553307	1.0598913	1.1349338	1.4078723	1.0244669	0.95672756	0.9067791	0.7637078	1.4459876	1.1350611	1.0746669	1.0758379	0.795044	1.144558	0.91292	1.182888	1.44	1.30	AT2G26870 phosphoesterase family protein low similarity to SP|Q9RGS8 Non-hemolytic phospholipase C precursor (EC 3.1.4.3) (Phosphatidylcholine chol[illegible]
251735_at	0.0112	1.3020177	1.2569879	1.5956556	1.5997002	0.96808076	0.95831066	0.92207754	0.6556831	0.9576307	1.224783	1.3653885	1.0319194	0.8649368	1.0963188	0.8837375	0.89382094	1.43859	0.876038	1.14493	0.934704	0.61	0.82	AT3G56090 ferritin, putative similar to ferritin subunit cowpea2 precursor [Vigna unguiculata] GI:2970654; contains Pfam profile PF00210: Ferritin-like do[illegible]
260536_at	0.0112	0.90876657	0.7101429	1.137742	0.92441213	1.3758379	1.0340061	1.6562384	1.9038401	0.8273877	0.70965743	0.65329	0.85183805	1.2937028	0.965994	1.3210791	1.0682347	0.920266	1.492481	0.760543	1.162253	1.62	1.53	AT2G43400 electron transfer flavoprotein-ubiquinone oxidoreductase family protein contains Pfam profile: PF05187 Electron transfer flavoprotein-ubiqui[illegible]
262908_at	0.0111	1.0098153	1.0486227	1.1580945	1.1245621	1.03479	0.86970925	0.8967414	0.9954644	1.0045357	1.0740825	0.904642	1.0103192	0.83770436	0.83638173	0.878158	0.8159846	1.085274	0.949176	0.998395	0.842057	0.87	0.84	AT1G59900 pyruvate dehydrogenase E1 component alpha subunit, mitochondrial (PDHE1-A) identical to SP|P52901 Pyruvate dehydrogenase E1 compone[illegible]
265071_at	0.0111	0.9722138	1.2069134	1.0723841	1.2419491	0.7651828	0.8336607	0.7906841	0.70582044	1.4117635	1.2095537	1.0277863	1.0635663	1.1550908	0.9380832	0.8051013	0.7809272	1.123365	0.773837	1.178167	0.919801	0.69	0.78	AT1G55520 transcription initiation factor IID-2 (TFIID-2) / TATA-box factor 2 / TATA sequence-binding protein 2 (TBP2) identical to Swiss-Prot:P28148|TF2[illegible]
264307_at	0.0111	1.1980674	1.1406839	1.1480011	1.4117005	1.0142893	0.92948925	0.96378773	1.1353625	0.9974844	0.9957004	1.0025156	1.1555982	0.8649706	0.83254844	0.90498763	0.7394797	1.224613	1.010732	1.037825	0.835497	0.83	0.81	AT1G61900 expressed protein contains similarity to glutamic acid/alanine-rich protein GI:6707830 from [Trypanosoma congolense]
265707_at	0.0111	1.0392236	1.080538	1.1202569	0.92730284	0.872394	0.6303932	0.8751976	0.69427466	1.4590316	1.3124806	1.1295257	1.217326	1.1610895	0.9607763	0.9080136	0.9509877	1.04183	0.768065	1.279591	0.995217	0.74	0.78	AT2G03390 uvrB/uvrC motif-containing protein contains Pfam profile PF02151: UvrB/uvrC motif
266791_at	0.011	1.1649848	1.2617409	1.2811478	1.2342018	0.98674285	1.0286802	1.1219867	1.0875599	0.9547947	0.9900099	1.0099902	0.93874747	0.8263646	0.9030361	0.92464685	0.95488673	1.235519	1.056242	0.973386	0.902234	0.85	0.93	AT2G02960 zinc finger (C3HC4-type RING finger) family protein contains Pfam profile PF00097: Zinc finger, C3HC4 type (RING finger); contains PROSITE PS[illegible]
263549_at	0.011	1.2194939	0.90910095	0.85043657	0.7730888	0.48291606	0.56899047	0.32332784	0.2853255	1.1497846	1.824674	1.5173965	1.090899	1.09199	1.1121612	0.8861301	1.1601763	0.93803	0.41514	1.395689	1.062614	0.44	0.76	AT2G21650 myb family transcription factor contains PFAM profile: PF00249 myb-like DNA-binding domain
262119_s_at	0.011	0.8025535	0.8599646	1.1577535	1.2227108	1.3646622	1.0757077	1.3615401	1.2434803	0.7039379	0.54031503	0.76129043	0.6276705	1.0205657	0.9794343	0.9694306	1.1754749	1.010746	1.261348	0.658303	1.036226	1.25	1.57	AT1G02920;AT1G02 [AT1G02920, glutathione S-transferase, putative similar to glutathione S-transferase GI:860955 from [Hyoscyamus muticus]; supported by cD[illegible]
252944_at	0.0109	1.0543569	1.0302448	0.9860393	1.1217113	0.8221076	0.9429627	0.86882037	1.0139606	1.029756	1.1448478	1.2104064	1.0384772	0.95102584	0.90373224	0.9376325	0.91624993	1.048088	0.911963	1.105872	0.92716	0.87	0.84	AT4G39320 microtubule-associated protein-related contains weak similarity to microtubule-associated protein 1B (MAP 1B) (Swiss-Prot:P46821) [Homo s[illegible]
254153_at	0.0109	1.2621509	1.5845026	1.3683326	1.4462932	0.817412	0.8742427	0.68801904	1.4800453	1.0542336	1.0645347	1.0007576	0.9992424	0.7120167	0.54205745	0.5939338	0.7567086	1.41532	0.96493	1.029682	0.651179	0.68	0.63	AT4G24450 starch excess protein-related similar to SEX1 [Arabidopsis thaliana] GI:1204358
[illegible]																								

263184_at	0.00357	0.96379805	1.0218915	2.1092567	0.9781086	0.45100278	0.3450574	0.5280745	0.5504689	4.434536	2.6464448	2.4890194	1.7530087	1.6652703	0.8498108	0.724349	1.0927721	1.268264	0.468651	2.830752	1.083051	0.37	0.38	AT1G05560	UDP-glucose transferase (UGT75B2) similar to UDP-glucose:indole-3-acetate beta-D-glucosyltransferase GI:2149127 from (Arabidopsis thaliana); identical to cDNA UDP-glucosyltransferase (UGT75B2) GI:13661274
250936_at	0.00357	1.2685323	1.4654319	1.4029833	1.9630142	0.61995804	0.8671897	0.6584255	0.35879445	1.0179915	1.3580834	1.3083587	1.3421675	0.7172223	0.93944824	0.9820085	0.6053465	1.52499	0.626092	1.25665	0.811006	0.41	0.65	AT5G03120	expressed protein
260526_at	0.00356	0.8889515	0.67245454	0.90335894	0.89668256	1.0966411	1.4561123	1.1871766	2.2492068	0.8976665	0.86201453	0.84508497	0.85440284	1.2230887	1.6440614	1.6145413	1.3660538	0.840362	1.497284	0.864792	1.461936	1.78	1.69	AT2G47410	transducin family protein / WD-40 repeat family protein contains 5 WD-40 repeats (PF00400); similar to WDR protein, form B (GI:14970593) [Mus musculus]
247657_at	0.00355	1.2528071	1.4438559	0.9970085	1.058855	0.86673576	0.81666386	0.7798534	0.6447868	1.0840641	1.2232795	1.3537642	1.1772567	1.0029914	0.9019853	0.97074306	0.82114094	1.188157	0.77701	1.209591	0.924215	0.65	0.76	AT5G59845	gibberellin-regulated family protein similar to SP|P27057 GAST1 protein precursor (Lycopersicon esculentum); contains Pfam profile PF02704: Gibberellin regulated protein
267635_at	0.00344	0.9027345	0.9705468	0.67332906	0.81468314	1.452597	1.4599944	1.3426646	0.9034256	1.0182375	0.9817625	0.89729106	0.88325036	1.4594768	1.5826169	1.5515728	1.2583019	0.840323	1.28967	0.945135	1.462992	1.53	1.55	AT2G42220	rhodanese-like domain-containing protein contains rhodanese-like domain PF:00581
263804_at	0.00341	0.45893082	0.4765758	0.569817	0.9943231	1.0436945	1.0056767	1.0715107	1.8169663	0.5081548	0.6758022	0.6801411	0.80138373	1.2762315	1.4883133	1.201543	1.7896451	0.624912	1.234462	0.66887	1.438933	1.98	2.15	AT2G40270	protein kinase family protein contains Pfam domain, PF00069: Protein kinase domain
253958_at	0.00332	1.2587874	1.0024228	1.2422101	1.312434	0.9975771	0.8124259	0.89860743	0.8177425	1.0375793	1.2234643	1.3448067	1.1642364	0.98583794	0.85152763	0.962953	0.88468903	1.203964	0.881588	1.192522	0.921252	0.73	0.77	AT4G26400	zinc finger (C3HC4-type RING finger) family protein contains Pfam domain, PF00097: Zinc finger, C3HC4 type (RING finger)
267357_at	0.00331	0.77559185	0.77648515	0.802462	0.9398222	1.4267044	1.1691238	1.0260941	1.5696354	0.64046246	0.88919246	0.9739059	0.93045706	1.3668209	1.2336434	1.12203	1.408258	0.82359	1.297889	0.858504	1.282688	1.58	1.49	AT2G40000	expressed protein
267186_at	0.0033	0.8356891	0.71049964	0.7944131	0.6329954	1.3021815	1.2854273	1.05225	2.0930367	0.94774985	0.9275193	0.71946424	0.73753154	1.1455504	1.1487131	1.2723824	1.1966653	0.743399	1.433224	0.833066	1.190828	1.93	1.43	AT2G44090	expressed protein
266369_at	0.00328	0.7524221	0.6331275	0.68405485	0.76512235	0.96487486	1.1520422	1.0492245	1.5574139	1.0351251	0.9524758	0.78815335	0.77171534	1.4412807	1.2462662	1.0938963	1.1539013	0.708682	1.180889	0.886867	1.233836	1.67	1.39	AT2G41370	ankyrin repeat family protein / BTB/POZ domain-containing protein contains Pfam domain, PF00023: Ankyrin repeat and Pfam domain, PF00651: BTB/POZ domain
266188_at	0.00328	0.71205466	0.7987487	0.73778147	0.7291947	1.3599136	1.0656044	1.3279696	0.82855606	1.1297909	0.9343957	0.84798837	0.8819074	1.6361966	1.358905	1.3151278	1.3269856	0.744695	1.145511	0.948521	1.409304	1.54	1.49	AT2G39000	GCN5-related N-acetyltransferase (GNAT) family protein contains Pfam profile PF00583: acetyltransferase, GNAT family
264019_at	0.00328	1.2481842	1.5023601	1.1837623	1.2245767	0.8324166	0.67726374	0.6596292	0.32709032	1.3724657	1.1694735	1.1687578	1.1600455	0.83995444	0.7666944	0.6836331	0.7925029	1.289721	0.6241	1.217686	0.770696	0.48	0.63	AT2G21130	peptidyl-prolyl cis-trans isomerase / cyclophilin (CYP2) / rotamase identical to cyclophilin [Arabidopsis thaliana] gi|2443757|gb|AAB71402; similar to peptidyl-prolyl cis-trans isomerase, PPIase (cyclophilin, cyclosporin A-binding protein) [Arabidopsis thaliana] SWISS-PROT:P34790
261449_at	0.00326	0.60069054	0.5139265	0.69262666	0.8424769	1.0669047	1.3285402	1.522418	1.350454	0.79256636	0.7984025	0.84192026	0.9330954	3.5942557	1.2346286	1.4752527	2.326975	0.66243	1.317079	0.841496	2.157778	1.99	2.56	AT1G21120	O-methyltransferase, putative similar to GI:2781394
246146_at	0.00324	0.74135166	0.7646337	0.95092386	0.9545187	1.072022	1.0760568	1.0685865	1.0811968	0.6842964	0.7012091	0.9041017	0.8505807	1.0552292	1.0944498	1.0454813	1.2129924	0.852857	1.074666	0.785047	1.102038	1.26	1.40	AT5G20050	protein kinase family protein contains protein kinase domain, Pfam:PF00069
251975_at	0.00324	1.1941453	1.1662916	1.5148401	0.9638607	0.84743047	0.76518184	0.81190044	0.80970997	1.4607675	1.6795936	1.205269	1.1320373	1.0361391	0.9066852	0.8156206	0.8725591	1.209784	0.808556	1.369417	0.907751	0.67	0.66	AT3G53230	cell division cycle protein 48, putative / CDC48, putative very strong similarity to SP|P54609 Cell division cycle protein 48 homolog (Arabidopsis thaliana); contains Pfam profiles PF00004: ATPase AAA family, PF02359: Cell division protein 48 (CDC48) N-terminal domain
247232_at	0.00324	0.739487	0.85431176	0.6754029	0.5360109	1.307994	1.2411664	0.95618016	2.0053031	1.0438198	0.82021666	0.7900328	0.8284451	1.3839653	1.4451357	1.2788246	1.3104963	0.701303	1.377661	0.870629	1.354605	1.96	1.56	AT5G64940	ABC1 family protein contains Pfam domain, PF03109: ABC1 family
251797_at	0.00322	0.8145267	0.94762605	1.1763724	1.0534807	1.150686	1.3824549	1.3444203	1.5974522	0.7904833	0.7862361	0.6991332	0.6938991	0.981256	1.0121626	0.9878375	1.118324	0.998001	1.368753	0.742438	1.024895	1.37	1.38	AT3G55560	DNA-binding protein-related contains Pfam domain PF03479: Domain of unknown function (DUF296), found in AT-hook motifs Pfam:PF02178
245174_at	0.00322	0.66695386	0.8421685	0.6617036	0.8425689	1.4026206	1.2182881	1.0124569	1.9410574	0.90010357	0.98754317	0.73072946	0.7885981	1.217502	1.2386928	1.2274871	1.1623244	0.753349	1.393606	0.851744	1.211502	1.85	1.42	AT2G47500	kinesin motor protein-related
[illegible]																									

[illegible]

256601_s_at	0.00066	1.2163801	1.3300642	1.3291954	1.1418657	0.9993147	0.71792555	0.78337336	0.89580685	1.4583906	1.0006852	1.2038702	1.2043176	0.79550576	0.7226765	0.8412832	0.7768068	1.254376	0.849105	1.216816	0.784068	0.68	0.64	AT3G28290;AT3G28	[AT3G28290, integrin-related protein 14a identical to At14a prot [illegible]
246597_at	0.000658	0.9810261	0.86304414	0.818191	0.80932	1.1069732	1.2810167	1.3686662	1.4959567	0.89151365	0.8749913	0.8702209	0.90124166	1.3072278	1.2457554	1.0189741	1.1011512	0.867895	1.313153	0.884492	1.168467	1.51	1.32	AT5G14760	L-aspartate oxidase family protein similar to L-aspartate oxidase, E [illegible]
267015_at	0.00065	0.848192	0.74800444	0.9427576	0.89647	1.7201515	1.5356401	1.5247087	3.0038452	0.77346456	0.60632753	0.7137307	0.69617873	1.1079205	1.0572823	1.1864684	1.215745	0.85889	1.946086	0.697425	1.141854	2.27	1.64	AT2G39340	SAC3/GANP family protein contains Pfam profile: PF03399 SAC3/G [illegible]
265800_at	0.00065	0.8716685	0.7140585	0.939712	0.7979805	1.2046008	1.1338585	1.5195048	1.9706547	0.91260606	0.7718822	0.81584597	0.8901633	1.1957368	1.4882207	1.4130621	1.060288	0.830855	1.564521	0.847624	1.289327	1.88	1.52	AT2G35630	microtubule organization 1 protein (MOR1) identical to microtubul [illegible]
256415_at	0.000632	1.7184775	1.7005978	1.7750919	1.8613993	1.0076361	1.0242976	0.992364	0.6596744	0.98368096	0.8302785	1.0476694	1.0398592	0.69328415	0.8573168	0.7348239	0.7137718	1.763892	0.920993	0.975359	0.749799	0.52	0.77	AT3G11210	GDSL-motif lipase/hydrolase family protein contains Pfam profile P [illegible]
266413_at	0.000627	0.8770251	0.9464707	0.6469163	0.8151216	1.8330793	1.5310143	1.5377191	0.99148124	0.76174086	0.77739	1.0085188	0.79776007	1.4159693	1.7048775	1.7283102	1.8326499	0.821383	1.473323	0.836315	1.670463	1.79	2.00	AT2G38740	haloacid dehalogenase-like hydrolase family protein similar to SP| [illegible]
254776_at	0.000622	1.1921333	1.3000859	1.2047526	1.27695	1.0671118	0.91107357	0.9643488	0.97790325	1.1577647	1.0166327	0.9833673	1.0209376	0.9254079	0.87862235	0.831242	0.8198264	1.242417	0.980109	1.044676	0.863775	0.79	0.83	AT4G13360	enoyl-CoA hydratase/isomerase family protein similar to CoA-thioe [illegible]
266437_at	0.000622	0.735089	0.7696551	0.91951036	0.9991052	1.4833182	1.6221477	1.8062432	2.7529464	0.68113565	0.8560711	0.60273355	0.6714618	1.0008948	1.123218	1.2416776	1.1956859	0.85584	1.916164	0.702851	1.140369	2.24	1.62	AT2G43160	epsin N-terminal homology (ENTH) domain-containing protein con [illegible]
263986_at	0.000622	0.9346117	0.8116196	1.046958	0.9530423	1.5039625	1.2967821	1.3735855	1.9176556	0.6625145	0.7954687	0.782945	0.70924973	0.94353056	1.1903002	1.2585531	1.2828045	0.936558	1.522996	0.737544	1.168797	1.63	1.58	AT2G42790	citrate synthase, glyoxysomal, putative strong similarity to SP|P492 [illegible]
252852_at	0.00062	1.1641343	1.0764571	1.0828196	1.0548475	0.7020302	0.8039996	0.80295056	0.59613264	1.0574199	1.2702177	1.1298428	1.3166002	0.93487513	0.9451525	0.91043663	0.800842	1.094565	0.726278	1.19352	0.897827	0.66	0.75	AT4G39900	expressed protein
261459_at	0.000618	0.67302	0.86291975	0.9447417	0.9464358	1.9373355	1.5759071	1.6145176	1.774633	0.6048046	0.22395726	0.46104598	0.44520566	1.2572379	1.0913061	1.0535643	1.6796666	0.856779	1.725598	0.433753	1.270444	2.01	2.93	AT1G21100;AT1G21	[AT1G21100, O-methyltransferase, putative similar to GI:2781394 [illegible]
263979_at	0.000618	0.68109274	0.7709605	0.6197562	0.60245216	1.6678	1.6883098	1.375048	0.9754398	0.8320494	0.6945354	0.76542896	1.0245602	1.319671	1.1884394	1.4726288	1.269673	0.668565	1.426649	0.829143	1.312603	2.13	1.58	AT2G42840	protodermal factor 1 (PDF1) identical to protodermal factor 1 [Ara [illegible]
266760_at	0.000615	0.6443009	0.7575198	0.81745017	0.8510527	1.0647396	1.0732381	1.0220572	1.3161402	0.82614446	0.9779428	0.7815239	0.7181506	1.1351905	1.2807856	1.2801147	1.264063	0.767581	1.119044	0.82594	1.240038	1.46	1.50	AT2G46870	DNA-binding protein, putative similar to DNA-binding proteins fro [illegible]
255827_at	0.000615	0.975087	0.9385748	0.9271496	0.87566084	1.3996797	1.5440696	1.7124414	1.0284914	0.89150566	0.96432805	0.8138277	0.89451516	1.4989508	1.3265203	1.4919193	1.35037	0.928223	1.421171	0.891044	1.41694	1.53	1.59	AT2G40600	appr-1-p processing enzyme family protein contains Pfam domain [illegible]
263582_at	0.000615	0.8981604	0.8007039	0.95544827	0.8158829	1.2178794	1.339296	1.6207972	1.1942005	0.88072217	0.8359402	0.87945855	0.9350378	1.2046169	1.0445516	1.2923313	1.4266468	0.867963	1.343043	0.88279	1.242037	1.55	1.41	AT2G17120	peptidoglycan-binding LysM domain-containing protein contains P [illegible]
267499_at	0.000615	0.8778219	0.7505067	0.806093	0.8160466	1.3981409	1.0736609	1.4623652	1.601802	0.8877871	0.92633915	0.6322515	0.7001105	1.2946038	1.3607705	1.1241125	1.16771	0.812636	1.383992	0.786622	1.236799	1.70	1.57	AT2G45500	AAA-type ATPase family protein similar to SP|Q9QYY8 Spastin (Fra [illegible]
266101_at	0.000613	0.5534888	0.44533168	0.6957193	0.8721762	1.2640684	1.5015786	1.6335588	1.9878626	0.4308396	0.66472566	0.7879436	0.6957314	1.1278238	1.3928497	1.7756644	2.0677245	0.641679	1.596767	0.64481	1.590966	2.49	2.47	AT2G37940	expressed protein
266986_at	0.000613	0.8833729	0.8146438	1.018886	0.9376241	1.2812673	1.3080107	1.1440716	0.98111403	0.88027215	0.864153	0.93821406	0.77408916	1.3724043	1.3619756	1.3282565	1.2217209	0.913632	1.178616	0.864182	1.321089	1.29	1.53	AT2G39630	glycosyl transferase family 2 protein similar to dolichyl-phosphate [illegible]
266394_at	0.000605	0.9422358	0.94857043	0.8455999	0.8723335	1.4919035	1.6689804	1.5888749	1.0514295	0.64139247	0.8006135	0.79913026	0.8324625	1.1364259	1.7146767	1.7726362	1.5682565	0.902185	1.450297	0.7684	1.547999	1.61	2.01	AT2G43130	Ras-related protein (ARA-4) / small GTP-binding protein, putative i [illegible]
251739_at	0.000605	1.1051368	1.0520858	1.2408185	1.1396679	0.84540645	0.9056696	0.9479142	0.6911944	1.1140285	1.2851121	1.1606451	1.1358336	0.8755554	0.86782384	0.8140642	0.8491627	1.134427	0.847546	1.173905	0.851652	0.75	0.73	AT3G56170	Ca(2+)-dependent nuclease identical to Ca(2+)-dependent nuclease [illegible]
265884_at	0.000605	0.7005285	0.76394	0.6809266	0.7618057	1.2493976	1.7164435	1.107102	2.037719	0.89289784	0.88295996	0.728583	0.8069507	1.4345691	1.6225342	1.2963265	1.1383867	0.726794	1.527666	0.827848	1.372954	2.10	1.66	AT2G42320	nucleolar protein gar2-related contains weak similarity to Swiss-Pr [illegible]
267629_at	0.000605	0.82455045	0.993372	0.78545666	0.69279724	1.2435458	1.3032725	1.0073993	1.2721145	0.6572949	0.6604297	0.7951809	0.7575039	1.3005226	1.207305	1.0066279	1.1237384	0.824064	1.206583	0.717602	1.159548	1.46	1.62	AT2G42120	DNA polymerase delta small subunit-related similar to DNA polyme [illegible]
260499_at	0.000605	0.8939831	0.6641561	0.9127601	0.81825066	1.66506	1.5075562	1.5480342	1.0231816	0.7359678	0.9768184	0.78925824	0.73023367	1.4216793	1.4976218	1.4043273	1.2513194	0.822287	1.435958	0.80807	1.393737	1.75	1.72	AT2G41760	expressed protein
255093_s_at	0.000605	1.0694507	1.0794216	1.188941	1.1470549	0.8252749	0.8582022	0.9338282	0.91695887	1.1481099	1.021509	1.0336223	1.108055	0.9784911	0.937913	0.8612723	0.842324	1.121217	0.883566	1.077824	0.9	0.79	0.84	AT4G08580	microfibrillar-associated protein-related similar to Microfibrillar-as [illegible]
260570_at	0.000604	0.7235633	0.8652514	0.87271365	0.8188567	1.6194293	1.3572897	0.992985	1.8087366	1.007015	0.96370494	0.90252703	0.7647745	1.5209599	1.6686587	1.521856	1.5755588	0.820096	1.44461	0.909505	1.571758	1.76	1.73	AT2G43710	acyl-[acyl-carrier-protein] desaturase / stearoyl-ACP desaturase (S [illegible]
254093_at	0.000604	1.017387	1.0612842	0.9674323	0.81887496	1.3249658	1.6489818	1.7292678	1.6942533	0.648407	0.85694677	0.735971	0.69254845	1.3131337	0.95421565	0.9826129	1.2942189	0.966245	1.599367	0.733468	1.090595	1.66	1.49	AT4G25110	latex-abundant family protein (AMC2) / caspase family protein cont [illegible]
257375_at	0.000604	0.75275946	0.72312474	0.79953074	0.8021783	1.1207939	1.2319154	1.2433316	0.7475751	0.9718993	1.0281007	0.95136696	0.9430422	2.806355	2.3761797	1.6947606	2.4090152	0.769398	1.085904	0.973602	2.321578	1.41	2.38	AT2G38640	expressed protein contains Pfam profile PF04525: Protein of unkno [illegible]
253173_at	0.000601	0.6814724	0.52857864	0.84091866	0.7287574	1.113132	1.3736649	1.7679608	2.0119495	0.84310496	0.84176004	0.82840127	0.8868794	1.3941512	1.275431	1.5486334	1.8141721	0.694932	1.566677	0.850034	1.446843	2.25	1.70	AT4G35110	expressed protein
245137_at	0.000595	0.8818834	0.7717166	0.73224735	0.6414017	1.1852015	1.4232342	1.3962262	2.0510404	0.85968006	0.7684447	0.8552549	0.87625194	1.1622056	1.1635364	1.130129	1.1181195	0.756811	1.513926	0.839908	1.143498	2.00	1.36	AT2G45460	forkhead-associated domain-containing protein / FHA domain-con [illegible]
255380_at	0.000588	1.2613717	1.7003244	1.643826	1.3382335	0.9503434	0.83748925	0.7326176	0.99187636	1.2080176	1.2150506	1.3069878	1.0081236	0.62900126	0.8058897	0.8559011	0.7746579	1.485939	0.878082	1.184545	0.766362	0.59	0.65	AT3G16650	PP1/PP2A phosphatases pleiotropic regulator 2 (PRL2) identical to [illegible]
267144_at	0.000588	0.7886464	0.5716478	0.66316444	0.5974587	1.1606715	1.3511305	1.0081837	1.4382678	0.87985015	0.9918162	0.8240753	0.9628035	1.1059451	1.2191075	1.1566632	1.4346883	0.655229	1.239563	0.914636	1.229101	1.89	1.34	AT2G38110	phospholipid/glycerol acyltransferase family protein low similarity [illegible]
246365_at	0.000588	0.6345062	0.74912626	0.9045167	0.80447876	1.2684414	1.5432842	1.2705646	2.2113183	0.7335915	0.8892294	0.6800986	0.72623444	1.0954834	1.4652625	1.3917791	1.3943963	0.773157	1.573395	0.757288	1.33673	2.04	1.77	[illegible]	glutamate synthase, chloroplast (GLU1) / ferredoxin-dependent gl [illegible]
245121_at	0.000588	0.83242786	0.9601323	0.84746194	0.8076978	1.5280954	1.2773991	1.3972044	1.0398676	0.6121596	0.7497211	0.72330827	0.89963937	1.1903616	1.293777	1.2539372	1.1102743	0.86193	1.310642	0.746207	1.212088	1.52	1.62	AT2G47610	60S ribosomal protein L7A (RPL7aA)
245114_at	0.00058	0.7549444	0.6599025	0.994872	0.85465956	1.029987	1.0051279	1.1493424	1.1343995	0.8131552	0.8054414	0.82145774	0.86688894	1.5013124	1.278233	1.2461858	1.2720032	0.816095	1.079714	0.826736	1.326434	1.32	1.60	AT2G41630	transcription initiation factor IIB-1 / general transcription factor TF [illegible]
245117_at	0.000562	0.74534696	0.8441487	0.8029337	0.7794529	1.3974434	1.5589895	1.3317778	2.5825713	0.7754515	0.68155175	0.83469325	0.836919	1.1558512	1.1903058	1.3899248	1.3029606	0.792971	1.717696	0.781343	1.259761	2.17	1.61	AT2G41560	calcium-transporting ATPase 4, plasma membrane-type / Ca2+-ATP [illegible]
246317_at	0.000556	1.0808061	1.0534692	1.0596967	1.0233706	0.84683084	0.7898184	0.848676	0.88150585	1.175992	1.117528	1.1530247	1.0293126	0.97662944	0.95316505	0.9140782	0.7981534	1.054336	0.841708	1.118964	0.910507	0.80	0.81	AT3G56900	aladin-related / adracalin-related weak similarity to SP|Q9NRG9 Al [illegible]
252671_at	0.000556	1.0823882	1.1746686	1.2868811	1.2110714	0.76420546	0.68162817	0.9008383	0.8232568	1.5344332	1.6669871	1.3598047	1.101635	0.91511416	0.9168419	0.79407376	0.91761184	1.188752	0.792482	1.415715	0.88591	0.67	0.63	AT3G44190	pyridine nucleotide-disulphide oxidoreductase family protein low s [illegible]
263846_at	0.000554	0.7053258	0.8316248	0.7249532	0.6988045	1.146312	1.0593722	1.808033	1.6709627	0.9350923	0.9406277	0.7747675	0.9062609	1.4827216	1.5994452	1.2637784	1.3644233	0.740177	1.239363	0.889187	1.427592	1.67	1.61	AT2G36990	RNA polymerase sigma subunit SigF (sigF) / sigma-like factor (SIG6) [illegible]
260588_at	0.000554	1.2055286	1.3300065	1.2559738	1.257474	0.97694755	0.8705818	1.0170894	1.0038017	1.0237708	0.9961981	0.9909748	1.0222701	0.94859964	0.81667125	0.75702965	0.7997161	1.262246	0.967105	1.008303	0.830504	0.77	0.82	AT1G53320	F-box family protein / tubby family protein (TULP7) similar to Tubb [illegible]
266441_at	0.000554	0.62309617	0.6548729	0.8175936	0.6817105	1.488853	1.3223602	1.5682665	2.4179604	0.8609073	0.46715745	0.75877124	0.7428737	1.28312	1.1390928	1.2273499	1.2856046	0.694318	1.699368	0.707427	1.233792	2.45	1.74	AT2G43250	expressed protein and genefinder
250813_at	0.000552	1.041118	1.1647726	1.248016	1.1857826	0.89512384	0.891335	0.92597115	0.8203193	1.0142386	1.0894165	1.0785611	1.0980062	0.98576134	0.96610427	0.9857051	0.9510637	1.159922	0.883187	1.070056	0.972159	0.76	0.91	AT5G05000	translocon of chloroplast 34 (TOC34) / GTP-binding protein (OEP34 [illegible]
265414_at	0.000544	1.1480284	1.2065762	1.568683	1.0354671	0.77497673	0.75364304	0.7842931	0.96453303	1.1020782	1.0607861	1.151143	1.2205954	0.54510146	0.65278536	0.74576575	0.60577154	1.239689	0.819361	1.18435	0.637356	0.66	0.54	AT2G16660	nodulin family protein similar to nodulin-like protein [Arabidopsis t [illegible]
260562_at	0.000543	0.8396795	0.7276532	0.7328764	0.8355452	1.155166	1.1481274	1.6663499	2.196941	0.8518725	0.76494884	0.77423203	0.7938668	1.3574017	1.5674782	1.4356759	1.3535981	0.783939	1.541646	0.79623	1.428538	1.97	1.79	AT2G43850	ankyrin protein kinase, putative (APK1) similar to ankyrin-kinase [M [illegible]
267163_at	0.000536	0.7831263	0.6584754	0.8325904	0.77449775	1.2465315	1.3664125	1.3903295	2.5331163	0.7059102	0.6783229	0.76584077	0.72997856	1.2396561	1.2767227	1.1674098	1.4105655	0.762173	1.634097	0.720013	1.273589	2.14	1.77	AT2G37520	PHD finger family protein contains Pfam domain, PF00628: PHD-fin [illegible]
263962_at	0.000532	0.7190407	0.55610164	0.6815112	0.6702202	1.5342929	1.1140764	1.2898388	1.9027194	0.8676375	0.8466412	0.655628	0.9085619	1.4050559	1.091438	1.1361837	1.1394042	0.656718	1.460232	0.819617	1.19317	2.22	1.46	AT2G36350	protein kinase, putative similar to protein kinase KIPK (KCBP-intera [illegible]
265824_at	0.000532	0.7979085	0.81016695	0.79449266	0.7286216	1.374958	1.1968824	1.2153596	1.7788546	0.64852864	0.83735085	0.8554363	0.9098875	1.0901514	1.1159785	1.3432547	1.3061616	0.782797	1.391514	0.812791	1.213887	1.78	1.49	AT2G35650	glycosyl transferase family 2 protein similar to beta-(1-3)-glucosyl t [illegible]
267312_at	0.000529	0.776234	0.75237936	0.9790438	1.0063858	1.4907336	1.4304668	1.2809262	2.1517837	0.70833755	0.6449014	0.6879938	0.73345757	0.993614	1.1408943	1.1819737	1.1566597	0.878511	1.588478	0.693673	1.118285	1.81	1.61	AT2G34750	RNA polymerase I specific transcription initiation factor RRN3 famil [illegible]
263805_at	0.000528	0.6672865	0.6229987	0.37678864	0.617662	1.2913572	1.1139677	1.2648319	2.2137284	0.8860324	0.775557	0.7699859	0.76911783	1.5441209	1.2895261	1.4821618	1.5236397	0.571184	1.470971	0.800173	1.459862	2.58	1.82	AT2G40400	expressed protein similar to GI:7572912 (At3g56140)[Arabidopsis t [illegible]
257352_at	0.000526	0.7560088	0.9306173	0.8332167	0.9011176	1.2779925	1.0484779	1.5088453	1.2740523	0.8120984	0.8761838	0.8352885	0.951522	1.3423151	1.3398281	1.3924197	1.1046655	0.85524	1.277342	0.868773	1.294802	1.49	1.49	AT2G34900	DNA-binding bromodomain-containing protein contains Pfam prof [illegible]
259165_at	0.000526	1.0308279	1.0098569	1.2880526	1.2262719	0.6926473	0.8742535	0.80169106	0.8386182	1.3018363	1.6665164	1.2779764	1.411905	0.94354904	0.9901431	0.93909335	0.8874638	1.138752	0.801803	1.414559	0.940062	0.70	0.66	AT3G01470	homeobox-leucine zipper protein 5 (HAT5) / HD-ZIP protein 5 / HD [illegible]
267392_at	0.000523	0.6744064	0.6924643	0.750497	0.9333596	1.1552451	1.068937	1.066664	1.4357773	0.6432103	0.6663963	0.8498992	0.76101494	1.0836561	1.1483349	1.1723907	1.2371793	0.762434	1.181656	0.73013	1.16039	1.55	1.59	AT2G44490	glycosyl hydrolase family 1 protein contains Pfam PF00232 : Glyco [illegible]
267009_at	0.000514	0.6843955	0.7257228	0.83550805	0.843509	1.0979911	1.2225022	1.2128406	1.8107303	0.9020089	0.71187705	0.70215136	0.67115194	1.167701	1.2452447	1.2691984	1.2120204	0.773295	1.336016	0.746797	1.223541	1.73	1.64	AT2G39260	MIF4G domain-containing protein similar to hUPF2 [Homo sapiens] [illegible]
258100_at	0.000514	0.96827906	1.031721	1.600725	1.7412508	1.7841454	1.9552225	2.1056764	2.4545877	0.20786104	0.1937417	0.27823064	0.2612203	0.66690356	0.7566725	0.9622779	1.4073861	1.335494	2.074908	0.235263	0.94831	1.55	4.03	AT3G23550;AT3G23	[AT3G23550, MATE efflux family protein similar to ripening regula [illegible]
265135_at	0.000513	0.7319379	0.49058136	0.770228	0.6155783	1.3704113	1.088335	1.2214631	1.6334646	0.6712198	0.91166484	0.7756752	0.6615909	1.1719881	1.4127772	1.7961371	1.2390007	0.652081	1.328419	0.755038	1.404976	2.04	1.86	AT1G51270	vesicle-associated membrane protein, putative / VAMP, putative si [illegible]
245063_at	0.000498	0.9502122	0.7858042	0.94678515	0.91025436	1.50148	1.7761347	1.4986007	1.0231737	0.7960912	0.695192	0.9768263	0.9349059	1.5014564	1.7449329	1.5970253	1.6454276	0.898264	1.449847	0.850754	1.622211	1.61	1.91	AT2G39795	mitochondrial glycoprotein family protein / MAM33 family protein [illegible]
267580_at	0.000487	0.9075259	0.8733461	0.7151455	0.80051684	1.2186848	1.7235341	1.1981263	1.0640268	0.9359731	0.62671465	0.86596274	0.8754597	1.5624132	1.4885985	1.8048427	1.7829025	0.824134	1.301093	0.826125	1.659689	1.58	2.01	AT2G41990	expressed protein
255625_at	0.00048	1.1284364	1.2137512	1.39154	1.6641542	0.6292358	0.7944081	0.7206209	0.72928935	1.2443428	1.3512044	1.0699707	1.3607606	0.89701897	0.9300292	0.9147015	0.6749946	1.34947	0.718389	1.25657	0.854186	0.53	0.68	AT4G01120	G-box binding factor 2 (GBF2) identical to G-box binding factor 2 [[illegible]
266977_at	0.000472	1.1984665	1.0787038	0.95527077	1.235555	2.81451	1.6073806	2.2114916	1.8486637	0.22137646	0.17240685	0.3747028	0.2865886	0.74649644	0.7453132	0.6648185	1.0447291	1.116999	2.120511	0.263769	0.797839	1.90	3.02	AT2G39420	esterase/lipase/thioesterase family protein similar to monoglyceri [illegible]
245127_at	0.000467	1.0183262	1.1590271	1.0999615	1.2012438	2.785684	1.5780413	2.6153343	3.0661438	0.31845853	0.4167071	0.50873685	0.5107678	0.7579188	0.9143724	0.8172996	0.98167284	1.11964	2.511301	0.438668	0.867816	2.24	1.98	AT2G47600	magnesium/proton exchanger (MHX1) identical to magnesium/pro [illegible]
265467_at	0.000463	0.7042668	0.5719237	0.50419855	0.55861986	1.1372138	1.2447534	1.1514223	2.0551782	0.8310403	0.90168196	0.69128126	0.72272116	1.2689532	1.5066506	1.4410039	1.098318	0.584752	1.397142	0.786681	1.328731	2.39	1.69	AT2G37050	leucine-rich repeat family protein / protein kinase family protein co [illegible]
254175_at	0.000463	1.066472	1.1391746	1.2251637	1.3100475	0.7242421	0.62744033	0.8317555	0.52316487	1.652808	1.1779481	1.2458241	1.3993901	0.93352807	0.8091186	0.66381437	0.704233	1.185214	0.676651	1.369013	0.778471	0.57	0.57	AT4G24050	short-chain dehydrogenase/reductase (SDR) family protein contain [illegible]
260525_at	0.000463	0.7643439	0.67705625	0.79565984	0.72033775	1.1477169	1.4865985	1.493808	2.427521	0.8522831	0.7651963	0.6883131	0.73292226	1.2208991	1.1645907	1.3527333	1.3334463	0.739349	1.638911	0.759678	1.267917	2.22	1.67	AT2G47250	RNA helicase, putative similar to SP|P53131 Pre-mRNA splicing fac [illegible]
266547_at	0.000461	0.84251994	0.8508958	1.0141618	0.9226825	1.6077656	1.4166548	1.2065828	0.9858381	0.8522496	0.7409587	0.8363872	0.77179366	1.4164728	1.4260753	1.559987	1.3085979	0.907565	1.304183	0.812847	1.427783	1.44	1.76	AT2G35320	expressed protein
246212_at	0.000459	1.0913726	1.318306	1.2825016	1.2282941	0.8456211	0.9765245	0.82350963	0.9462707	1.2660619	1.0234754	1.2241602	1.1356119	0.7898991	0.6920682	0.74146887	0.9081696	1.230119	0.897981	1.162327	0.782901	0.73	0.67	AT4G36930	basic helix-loop-helix (bHLH) protein SPATULA (SPT) identical to SP [illegible]
260585_at	0.000459	0.5702397	0.89452773	0.7112123	0.6454757	1.2849041	1.1054723	1.2600533	1.6536568	0.5591931	0.8295751	0.68302515	0.78596	1.1402357	1.2078469	1.2145741	1.2483231	0.705364	1.326022	0.714663	1.202745	1.88	1.68	AT2G43650	Sas10/U3 ribonucleoprotein (Utp) family protein contains Pfam pro [illegible]
265450_at	0.000458	0.7334407	0.46039695	0.7231172	0.6687798	1.3685387	1.767198	1.7538536	3.4266562	0.5272912	0.7565417	0.6872791	0.67565256	1.3823799	1.5309215	1.2434583	2.0899878	0.646434	2.079062	0.661691	1.561687	3.22	2.36	AT2G46620	AAA-type ATPase family protein contains Pfam profile: ATPase fam [illegible]

[illegible]

267432_at	3.45E-05	0.67049015	0.69155747	0.64468193	0.51465845	1.2491021	1.1169719	1.1150815	1.5461761	0.6824239	0.7654302	0.71978205	0.88491845	1.3389661	1.3709534	1.4583108	1.3553679	0.630347	1.256833	0.763139	1.3809	1.99	1.81	AT2G35020	UTP--glucose-1-phosphate uridylyltransferase family protein similar to SP|Q16222 UDP-N-acetylhexosamine pyrophosphorylase (Antigen X) (Homo sapiens); contains Pfam profile PF01704: UTP--glucose-1-phosphate uridylyltransferase
267358_at	0.000034	0.6575192	0.599498	0.56941456	0.5561289	1.216747	1.0777013	1.194255	1.4168985	0.90832055	0.9222986	0.6807706	0.8551291	1.5936961	1.4094806	1.3787374	1.2106214	0.59564	1.2264	0.84163	1.398134	2.06	1.66	AT2G39890	proline transporter 1 (ProT1) identical to proline transporter 1 GI:1769901 from [Arabidopsis thaliana]
266542_at	0.000034	0.6642931	0.7161541	0.7538565	0.64631814	1.342278	1.3896332	1.4001836	1.9734764	0.6462975	0.59933484	0.6129705	0.8281305	1.1718695	1.1824429	1.3057163	1.3991002	0.695155	1.526393	0.671683	1.264782	2.20	1.88	AT2G35100	exostosin family protein contains Pfam profile: PF03016 Exostosin family
266194_at	0.000034	0.8311374	0.68857396	0.6189475	0.6055886	1.243634	1.2253779	1.1688625	1.6686476	0.7079812	0.6206863	0.66195804	0.7561415	1.4250755	1.430209	1.3967826	1.2119162	0.686062	1.326631	0.686692	1.365996	1.93	1.99	AT2G39090	tetratricopeptide repeat (TPR)-containing protein low similarity to prediabetic NOD sera-reactive autoantigen [Mus musculus] GI:6670773, anaphase-promoting complex subunit 7 [Homo sapiens] GI:6180015; contains Pfam profile PF00515: TPR Domain
267018_at	3.39E-05	0.8204184	0.7367808	0.65331584	0.7804617	1.7076993	1.194359	1.2107258	1.2878528	0.77262324	0.7188418	0.7297157	0.69746584	1.226526	1.3157198	1.3277292	1.1795814	0.747744	1.350159	0.729662	1.262389	1.81	1.73	AT2G39140	pseudouridine synthase family protein
245147_at	3.37E-05	0.9739218	0.9604982	0.96555454	0.9095688	1.4632403	1.760856	1.7257146	2.002799	0.54806405	0.5734615	0.58922946	0.5840451	1.0260782	1.03365	1.3910676	1.5355964	0.952386	1.738152	0.5737	1.246598	1.83	2.17	AT2G45280	DNA repair family protein contains similarity to Swiss-Prot:O43502 DNA repair protein RAD51 homolog 3 [Homo sapiens]
265795_at	3.37E-05	0.7435465	0.6001037	0.6594153	0.6315948	1.2800013	1.1978172	1.1569608	1.1599413	0.86726177	0.9039898	0.678728	0.73100287	1.2617397	1.2764138	1.1651701	1.0960101	0.658665	1.19868	0.795246	1.199833	1.82	1.51	AT2G35780	serine carboxypeptidase S10 family protein similar to Serine carboxypeptidase II chains A and B (SP:P08819) (EC 3.4.16.6) [Triticum aestivum (Wheat)];
263975_at	0.000032	0.79043573	0.79734206	0.79894465	0.7382423	1.2563417	1.1300411	1.0871749	1.1505905	0.7155849	0.91282505	0.74651474	0.731877	1.0895967	1.0936636	1.1495141	1.1127169	0.781241	1.156037	0.7767	1.111373	1.48	1.43	AT2G42710	ribosomal protein L1 family protein
266985_at	0.000032	0.79455674	0.6796041	0.83038485	0.8268577	1.2814783	1.3988888	1.3786427	1.190634	0.61566156	0.8151532	0.7389626	0.5981336	1.1696151	1.2952529	1.3311143	1.1943758	0.782851	1.312411	0.691978	1.24759	1.68	1.80	AT2G39550	geranylgeranyl transferase type I beta subunit (GGT-IB) nearly identical to GI:11878247
266131_at	0.000032	0.64057034	0.6304901	0.8172912	0.7651737	1.4481804	1.2936339	1.897041	1.8746426	0.7555973	0.62236303	0.61030775	0.7165301	1.4643843	1.182709	1.2837929	1.3082348	0.713381	1.628374	0.6762	1.30978	2.28	1.94	AT2G45160	scarecrow transcription factor family protein
267188_at	0.000032	0.8992351	0.87107956	0.7418322	0.7628449	1.4539362	1.3601019	1.3199946	1.1007649	0.8178186	0.784123	0.7498187	0.6573339	1.3346798	1.2671629	1.2885839	1.2528785	0.818748	1.308699	0.752274	1.285826	1.60	1.71	AT2G44050	6,7-dimethyl-8-ribityllumazine synthase / DMRL synthase / lumazine synthase / riboflavin synthase identical to 6,7-dimethyl-8-ribityllumazine synthase, chloroplast [precursor] SP:O80575 from [Arabidopsis thaliana]
265454_at	0.000032	0.63493115	0.70693684	0.55189687	0.5839901	1.4320557	1.1450757	1.1227844	1.7823019	0.81828964	0.87721574	0.87263036	0.7619193	1.4816221	1.8882719	1.7738708	1.7378134	0.619439	1.370554	0.832514	1.720395	2.21	2.07	AT2G46530	transcriptional factor B3 family protein / auxin-responsive factor AUX/IAA-related contains Pfam profiles: PF02309 AUX/IAA family, PF02362 B3 DNA binding domain
266420_at	0.000032	0.6721135	0.82501364	0.7527231	0.77079284	1.3192879	1.2737136	1.2582388	1.3623674	0.77669555	0.7438664	0.5459574	0.7856774	1.1749864	1.2168313	1.2490898	1.2255164	0.755361	1.303402	0.713049	1.216606	1.73	1.71	AT2G38610	KH domain-containing protein
260606_at	0.000032	0.768881	0.7452884	0.7658856	0.7575329	1.0894308	1.2166935	1.3739834	1.2711587	0.91056913	0.8632943	0.7593434	0.87315084	1.38647	1.2305657	1.1882381	1.1195846	0.759397	1.237817	0.851589	1.231215	1.63	1.45	AT2G43770	transducin family protein / WD-40 repeat family protein contains 7 WD-40 repeats (PF00400); similar to U5 snRNP-specific 40 kDa protein (GI:3820594) [Homo sapiens]
265457_at	0.000032	0.740108	0.80259895	0.73661923	0.7538521	1.4765403	1.1046627	1.1501076	1.4485339	0.8953373	0.8637528	0.8612494	0.8382024	1.2458007	1.260532	1.3744578	1.4329311	0.758295	1.294961	0.864635	1.32843	1.71	1.54	AT2G46550	expressed protein
263987_at	2.98E-05	0.6808965	0.5938828	0.5269027	0.4421183	1.2896934	1.3195466	1.0342227	1.2873354	0.9657773	0.82832855	0.7816216	0.76983786	2.13476	1.8380128	1.5440587	1.667643	0.56095	1.2327	0.836391	1.796119	2.20	2.15	AT2G42690	lipase, putative similar to lipase [Dianthus caryophyllus] GI:4103627; contains Pfam profile PF01764: Lipase
260543_at	2.92E-05	0.84390926	0.67142034	0.8517695	0.731596	1.5099062	1.2318101	1.444736	1.6920763	0.61431015	0.75266993	0.77410156	0.72871155	1.1482304	1.2936817	1.3520497	1.2736652	0.774674	1.469632	0.717448	1.266907	1.90	1.77	AT2G43330	sugar transporter family protein similar to SP|Q96QE2 Proton myo-inositol co-transporter (Hmit) [Homo sapiens], SP|Q01440 Membrane transporter D1 [Leishmania donovani]; contains Pfam profile PF00083: major facilitator superfamily protein
263778_at	2.92E-05	0.7312604	0.8510976	0.8832792	0.7991601	1.4627277	1.3054926	1.4080367	1.6297953	0.76671606	0.656305	0.7377865	0.8417949	1.1167208	1.1517073	1.3557507	1.1392019	0.816199	1.451513	0.750651	1.190845	1.78	1.59	AT2G46470	OXA1 protein, putative similar to Oxa1 protein [Arabidopsis thaliana] GI:10176922
267310_at	2.92E-05	0.78405416	0.70352304	0.7243432	0.74782085	1.4227285	1.4726598	1.3281629	2.1275473	0.70301867	0.66421276	0.6866812	0.75399643	1.2159457	1.2933092	1.4981104	1.2308204	0.739935	1.587775	0.701977	1.309546	2.15	1.87	AT2G34680	leucine-rich repeat family protein contains leucine rich repeat (LRR) domains, Pfam:PF00560; identical to cDNA hypothetical protein (AIR9) mRNA, partial cds GI:3695020
267351_at	2.89E-05	0.7141141	0.7514537	0.652881	0.61737037	1.2522029	1.6011449	1.4086663	1.6842151	0.81561315	0.8465689	0.72785616	0.6075826	1.153431	1.2777421	1.3249444	1.2052084	0.683955	1.486557	0.749405	1.240331	2.17	1.66	AT2G40060	defective chloroplasts and leaves protein-related / DCL protein-related similar to DCL protein, chloroplast precursor (Defective chloroplasts and leaves protein) (Swiss-Prot:Q42463) [Lycopersicon esculentum]
266634_at	2.89E-05	0.69291914	0.71206844	0.670159	0.5157417	1.3841573	1.3968949	1.446457	1.9097089	0.7922483	0.77725166	0.61340743	0.625605	1.2077515	1.3267016	1.2773328	1.2402987	0.647722	1.534305	0.702128	1.263021	2.37	1.80	AT2G35530	bZIP transcription factor family protein contains Pfam domain PF00170: bZIP transcription factor; similar to G-Box binding protein 2 (GI:5381313) [Catharanthus roseus].
266167_at	2.89E-05	0.5246172	0.6373409	0.9632162	0.8624241	1.7314478	2.105709	1.913361	2.2867475	0.3577129	0.39645746	0.36123732	0.27725837	1.0367838	1.1603818	1.0679992	1.6437542	0.7469	2.009316	0.348167	1.22723	2.69	3.52	AT2G38860	protease I (pfpI)-like protein (YLS5) contains Pfam profile PF01965: DJ-1/PfpI family; supporting cDNA gi|13122287|dbj|AB047808.1|; identical to protease I (pfpI)-like protein [Arabidopsis thaliana] GI:13122288, cDNA protease I (pfpI)-like protein GI:13122287
245122_at	2.89E-05	0.7387905	0.85068893	0.9437939	0.8062699	1.5423625	1.4760735	1.6263759	1.3963416	0.6353345	0.80217123	0.7368621	0.7342647	1.0562061	1.3265282	1.4198873	1.3965775	0.834886	1.510288	0.727158	1.2998	1.81	1.79	AT2G47420	dimethyladenosine transferase, putative similar to SP|P41819 Dimethyladenosine transferase (EC 2.1.1.-) (S-adenosylmethionine-6-N', N'-adenosyl(rRNA) dimethyltransferase) [Saccharomyces cerevisiae]; contains Pfam profile PF00398: ribosomal RNA adenine dimethylase family protein
266608_at	2.72E-05	0.7595614	0.5524868	0.61869526	0.5802709	1.1099633	1.4669702	1.187458	1.3512027	0.769895	0.89003676	0.77389115	0.63379246	1.4089594	1.5869533	1.4644295	1.4292133	0.627754	1.278899	0.766904	1.472389	2.04	1.92	AT2G35500	shikimate kinase-related low similarity to shikimate kinase precursor from Lycopersicon esculentum [SP|Q00497]
255826_at	2.71E-05	0.8064713	0.78705055	0.6835662	0.6512208	1.3040131	1.1558706	1.2571601	1.5326658	0.68506825	0.7816937	0.8441295	0.6929009	1.1874608	1.3080574	1.3135417	1.241907	0.732077	1.312427	0.750948	1.262742	1.79	1.68	AT2G40490	uroporphyrinogen decarboxylase, putative / UPD, putative similar to uroporphyrinogen decarboxylase (chloroplast) from Nicotiana tabacum [SP|Q42967], Hordeum vulgare [SP|Q42855], Zea mays [SP|O81220]; contains Pfam domain uroporphyrinogen decarboxylase (URO-D) PF01208
263956_at	2.64E-05	0.7895483	0.7131519	0.6744111	0.592545	1.258619	1.1760422	1.066327	1.190537	0.9336731	0.8835591	0.7975067	0.7657972	1.5004675	1.4597967	1.2653853	1.4099361	0.692414	1.172881	0.845134	1.408896	1.69	1.67	AT2G35840	sucrose-phosphatase 1 (SPP1) identical to sucrose-phosphatase (SPP1) [Arabidopsis thaliana] GI:11127757
263777_at	0.000026	0.48844314	0.519122	0.46490216	0.48779747	1.2271018	1.6469827	1.2775221	2.6403825	0.77289814	0.6293765	0.6435289	0.52019083	1.8832289	2.1525958	1.7360665	2.1233964	0.490066	1.697997	0.641499	1.973822	3.46	3.08	AT2G46450	cyclic nucleotide-regulated ion channel, putative (CNGC12) similar to cyclic nucleotide and calmodulin-regulated ion channel (cngc3) GI:4581201 from [Arabidopsis thaliana]
267362_at	2.59E-05	0.7960157	0.7366566	0.85869306	0.60285693	1.3934329	1.3131037	1.4499636	1.3489144	0.82300955	0.79592526	0.7271503	0.7776926	1.4060295	1.141307	1.234345	1.2158631	0.748556	1.376354	0.780944	1.249386	1.84	1.60	AT2G39910	expressed protein
245087_at	2.55E-05	0.7648626	0.74237376	0.5883199	0.78506887	1.5963801	1.2657697	1.4530165	1.7943571	0.7398946	0.60625356	0.7496464	0.7344969	1.4300494	1.2366153	1.2935343	1.2149311	0.720156	1.527381	0.707573	1.293783	2.12	1.83	AT2G39830	LIM domain-containing protein contains Pfam profile PF00412: LIM domain
266057_at	2.55E-05	0.682848	0.86162645	0.74299794	0.7464927	1.1214023	1.3538061	1.3480982	1.4503952	0.70084316	0.8517559	0.7331193	0.8785977	1.3317624	1.4567587	1.3972013	1.2959154	0.758491	1.318425	0.791079	1.370409	1.74	1.73	AT2G40660	tRNA-binding region domain-containing protein similar to SP|Q12904 Multisynthetase complex auxiliary component p43 [Contains: Endothelial-monocyte activating polypeptide II (EMAP-II) (Small inducible cytokine subfamily E member 1)] [Homo sapiens]; contains Pfam profile PF01588: Putative tRNA binding domain
245062_at	2.53E-05	0.8033545	0.7848563	0.8196059	0.6864263	1.3138255	1.2871712	1.3010268	1.7578596	0.8018527	0.67758346	0.6930214	0.6367018	1.2209332	1.2999984	1.3104706	1.180394	0.773561	1.414971	0.70229	1.252949	1.83	1.78	AT2G39760	speckle-type POZ protein-related contains Pfam PF00651 : BTB/POZ domain; contains Pfam PF00917: MATH domain; similar to Speckle-type POZ protein (SP:O43791) [Homo sapiens]
263919_at	2.49E-05	0.6716778	0.8791179	0.7155546	0.8334294	1.1208822	1.3716775	1.392359	1.5258585	0.8224684	0.6355334	0.78499055	0.7517387	1.4200236	1.6053582	1.6354212	1.5951655	0.774945	1.352694	0.748683	1.563992	1.75	2.09	AT2G36470	expressed protein
265965_at	2.45E-05	0.8438305	0.64896107	0.680454	0.67625386	1.1756288	1.398865	1.2929577	1.6147455	0.7898808	0.78007036	0.7559818	0.75083107	1.1561695	1.3416631	1.2244967	1.1909126	0.712375	1.370549	0.769191	1.22831	1.92	1.60	AT2G37500	arginine biosynthesis protein ArgJ family contains Pfam profile: PF01960 ArgJ family
245066_at	2.24E-05	0.7856336	0.717915	0.7779679	0.87272274	1.4631155	1.067924	1.2890371	1.3453513	0.7684172	0.77282894	0.9320759	0.7982771	1.471042	1.622268	1.550207	1.7833381	0.78856	1.291357	0.8179	1.606714	1.64	1.96	AT2G39840	serine/threonine protein phosphatase PP1 isozyme 4 (TOPP4) / phosphoprotein phosphatase 1 identical to SP|P48484 Serine/threonine protein phosphatase PP1 isozyme 4 (EC 3.1.3.16) (Arabidopsis thaliana), phosphoprotein phosphatase 1 GI:166801 (Arabidopsis thaliana); contains a Ser/Thr protein phosphatase signature (PDOC00115); contains a metallo-phosphoesterase motif (PDOC50185)
263990_at	2.17E-05	0.7836545	0.7176673	0.8407637	0.7846408	1.3841288	1.3751626	1.3711985	1.9072053	0.7900749	0.8169991	0.71972454	0.72630054	1.1592363	1.3005962	1.2677423	1.289257	0.781682	1.509424	0.763275	1.254208	1.93	1.64	AT2G42810	serine/threonine protein phosphatase, putative similar to SP|P53042 Serine/threonine protein phosphatase 5 (EC 3.1.3.16) (PP5) (Protein phosphatase T) (PPT) (Rattus norvegicus); contains Pfam profiles PF00149: Ser/Thr protein phosphatase, PF00515: TPR Domain
266638_at	2.15E-05	0.7871196	0.750726	0.831791	0.6639017	1.2158462	1.1207902	1.2553954	1.2324975	0.8792098	0.7606279	0.7654258	0.71806234	1.3279717	1.2690746	1.1320803	1.1877073	0.758385	1.206132	0.780831	1.229208	1.59	1.57	AT2G35490	plastid-lipid associated protein PAP, putative similar to plastid-lipid associated protein PAP3 [Brassica rapa] GI:14248552; contains Pfam profile PF04755: PAP_fibrillin
266450_s_at	2.15E-05	0.7663828	0.83611894	0.7884581	0.72021234	1.1638811	1.3210156	1.6016189	1.3008796	0.79069984	0.69605076	0.69455916	0.8236137	1.735297	1.3319246	1.4046011	1.4696562	0.777793	1.346849	0.751231	1.48537	1.73	1.98	AT2G43190	ribonuclease P family protein similar to Ribonuclease P protein subunit p29 (EC 3.1.26.5) (hPOP4) (Swiss-Prot:O95707) [Homo sapiens]
245132_at	2.15E-05	0.8138858	0.78714526	0.76741105	0.7332464	1.3945956	1.2696804	1.3867242	1.1550876	0.8449125	0.7255537	0.70583034	0.81334746	1.4844764	1.2075138	1.2071925	1.1792825	0.775422	1.301522	0.772411	1.269616	1.68	1.64	AT2G45320	expressed protein
266192_at	2.15E-05	0.8419748	0.7673549	0.6774479	0.77925795	1.5503649	1.1637907	1.3100296	1.2846224	0.62327796	0.7615917	0.72073704	0.7003652	1.2222267	1.4056135	1.2371088	1.1580251	0.766509	1.327202	0.701493	1.255744	1.73	1.79	AT2G39080	expressed protein
248686_at	2.04E-05	0.37234846	0.4206364	0.43066135	0.5332531	2.0598488	2.768288	1.9938296	2.2387798	0.76122016	0.53764	0.5266895	0.5095707	2.636272	1.6155177	2.0307066	2.5731423	0.439225	2.015187	0.58378	2.21391	4.59	3.79	AT5G48540	33 kDa secretory protein-related contains Pfam PF01657: Domain of unknown function, duplicated in 33 KDa secretory proteins
266079_at	2.03E-05	0.5784193	0.8080746	0.68345314	0.5612908	1.4742954	1.3928711	1.2171186	1.4865303	0.79474497	0.804519	0.6991946	0.7058336	1.3226448	1.1919254	1.4247777	1.4051442	0.657809	1.392704	0.75051	1.336123	2.12	1.78	AT2G37860	expressed protein
266442_at	1.98E-05	0.7028284	0.87800163	0.7218899	0.74990064	1.4602035	1.1841359	1.4768037	1.1219983	0.6704568	0.7574036	0.67305684	0.74954975	1.4516345	1.2745976	1.3075225	1.3397856	0.76318	1.310785	0.712631	1.343385	1.72	1.89	AT2G43260	F-box family protein / S locus-related weak similarity to S locus F-box (SLF)-S2 protein [Antirrhinum hispanicum] GI:13161526; contains TIGRFAM TIGR01640: F-box protein interaction domain
262322_at	1.98E-05	1.5565083	1.3245323	1.2602997	1.1388048	0.7044789	0.80212176	0.7919923	0.86119527	1.4444069	1.4159698	1.2693768	1.2313906	0.7131322	0.60281646	0.6772149	0.55259603	1.320036	0.789947	1.340286	0.63644	0.60	0.47	AT1G27590	expressed protein similar to hypothetical protein GB:AAD45997 GI:5668770 from (Arabidopsis thaliana)
265206_at	1.81E-05	0.7985265	0.7165779	0.7642815	0.8173787	1.0774449	1.1034164	1.2565336	1.3568633	0.87730044	0.91600233	0.9225552	0.9038819	1.3479105	1.4254869	1.3708284	1.2550765	0.774191	1.198565	0.904935	1.349826	1.55	1.49	AT2G36670	aspartyl protease family protein contains Pfam profile: PF00026 eukaryotic aspartyl protease
265270_at	1.79E-05	0.86241513	0.94035894	0.90280133	0.89114296	1.6827602	1.533418	1.6124396	1.551013	0.69210666	0.8586678	0.77753115	0.83234084	1.3462555	1.059641	1.4440503	1.3134029	0.89918	1.594908	0.790162	1.290837	1.77	1.63	AT2G42910	ribose-phosphate pyrophosphokinase 4 / phosphoribosyl diphosphate synthetase 4 (PRS4) identical to phosphoribosyl diphosphate synthase (prs4) [Arabidopsis thaliana] GI:4902472
266819_at	1.79E-05	0.80599546	0.8769136	0.7784276	0.8095243	1.5215305	1.2761431	1.445821	1.1137085	0.88629144	0.7722509	0.7951757	0.77782524	1.4764045	1.3115287	1.3912122	1.4626124	0.817715	1.339301	0.807886	1.410439	1.64	1.75	AT2G44870	expressed protein
267386_at	1.79E-05	0.8112582	0.8887825	0.80075514	0.7514842	1.3891299	1.2420868	1.3947414	1.2322006	0.7099686	0.6663908	0.73228294	0.8164415	1.2049947	1.4136044	1.3328731	1.1112175	0.81307	1.31454	0.731271	1.265672	1.62	1.73	AT2G44270	expressed protein contains Pfam profile PF01171: PP-loop family
265817_at	1.74E-05	0.29764724	0.48022592	0.6697283	0.5967919	2.648742	2.4480472	2.2794077	1.3302716	0.6639644	0.4396617	0.4265253	0.5198262	3.551631	2.70237	3.0051558	3.0757346	0.511098	2.176617	0.512494	3.083723	4.26	6.02	AT2G18050	histone H1-3 (HIS1-3) similar to histone H1 [Lycopersicon pennellii] SWISS-PROT:P40267; identical to cDNA histone H1-3 (His1-3) GI:1809314, histone H1-3 [Arabidopsis thaliana] GI:1809305
267349_at	1.74E-05	0.7924184	0.7374911	0.6324247	0.6233723	1.52351	1.3889948	1.5385464	1.2621876	0.68363255	0.7123992	0.5918861	0.6675631	1.7906938	1.2075816	1.5409411	1.322137	0.696427	1.42831	0.66387	1.465338	2.05	2.21	AT2G40010	60S acidic ribosomal protein P0 (RPP0A)
263826_at	1.74E-05	0.84842825	0.71149784	0.84001356	0.8194888	1.4954436	1.4911327	1.2743052	1.4491146	0.74513644	0.8170081	0.7591206	0.60367554	1.1515716	1.2770001	1.2517473	1.2198534	0.804857	1.427499	0.731235	1.225043	1.77	1.68	AT2G40410	Ca(2+)-dependent nuclease, putative similar to Ca(2+)-dependent nuclease [Arabidopsis thaliana] GI:7684292
260571_at	1.61E-05	0.76575744	0.72261864	0.8901484	0.83710265	1.4181186	1.3583977	1.2017866	1.6311202	0.73314226	0.6916465	0.665292	0.6319535	1.1098516	1.2424604	1.2215507	1.1466558	0.803907	1.402356	0.680509	1.18013	1.74	1.73	AT2G43790	mitogen-activated protein kinase, putative / MAPK, putative (MPK6) identical to mitogen-activated protein kinase homolog 6 (AtMPK6)[Arabidopsis thaliana] SWISS-PROT:Q39026; PMID:12119167
266582_at	1.23E-05	0.84467804	0.7905015	0.85616964	0.87056553	1.7276186	1.4880912	1.7206391	1.3193222	0.77645844	0.74244165	0.7575224	0.7091937	1.5075823	1.1294345	1.3533918	1.3249612	0.840479	1.563918	0.746404	1.328842	1.86	1.78	AT2G46090	diacylglycerol kinase family protein contains INTERPRO domain, IPR001206, DAG-kinase catalytic domain
266319_s_at	1.23E-05	0.6769218	0.5714563	0.57601315	0.6998317	1.2216507	1.4109462	1.525681	1.5707308	0.78212637	0.70914745	0.69180936	0.55732685	1.5569497	1.5182924	1.330046	1.2178737	0.631056	1.432252	0.685103	1.40579	2.27	2.05	AT2G46720;AT3G10	[AT2G46720, fatty acid elongase 3-ketoacyl-CoA synthase, putative similar to GI:4091810; contains Pfam profile PF02797: Chalcone and stilbene synthases, C-terminal domain];[AT3G10280, fatty acid elongase 3-ketoacyl-CoA synthase, putative similar to fatty acid elongase 3-ketoacyl-CoA synthase 1 GB:AAC99312 [Arabidopsis thaliana]]
266957_at	1.23E-05	0.6717373	0.7606646	0.54855454	0.66084146	1.2393354	1.4014488	1.315364	1.7699369	0.7135669	0.6290328	0.7021287	0.62672454	1.3482882	1.4717698	1.2485527	1.3094593	0.660449	1.431521	0.667863	1.344518	2.17	2.01	AT2G34640	expressed protein
267027_at	1.23E-05	0.6706421	0.67853314	0.5496597	0.5430781	1.1766003	1.1549815	1.2059718	1.3318496	0.8450184	0.8072162	0.70553213	0.7087931	1.4867474	1.3203765	1.2287935	1.2028452	0.610478	1.217351	0.76664	1.309691	1.99	1.71	AT2G38330	MATE efflux family protein low similarity to enhanced disease susceptibility 5 [Arabidopsis thaliana] GI:16589070; contains Pfam profile PF01554: Uncharacterized membrane protein family
260566_at	1.17E-05	0.8593891	0.8053062	0.8320587	0.8280836	1.4900032	1.1944302	1.263378	1.2561166	0.8030572	0.830518	0.7699206	0.6892665	1.140611	1.2135159	1.2428969	1.1577518	0.831209	1.300982	0.773191	1.188694	1.57	1.54	AT2G43750	cysteine synthase, chloroplast / O-acetylserine (thiol)-lyase / O-acetylserine sulfhydrylase / cpACS1 (OASB) identical to SP|P47999 Cysteine synthase, chloroplast precursor (EC 4.2.99.8) (O-acetylserine sulfhydrylase) (O-acetylserine (Thiol)-lyase) (cpACS1) [Arabidopsis thaliana]; identical to cDNA O-acetylserine lyase (At.OAS.7-4) GI:6983575
260552_at	1.13E-05	0.8015132	0.7825267	0.78021467	0.9147695	1.5707006	1.260339	1.4463859	1.3282737	0.6431071	0.71089166	0.6378287	0.7581982	1.2716082	1.1353372	1.2117969	1.0852305	0.819756	1.401425	0.687506	1.175993	1.71	1.71	AT2G43430	hydroxyacylglutathione hydrolase, mitochondrial / glyoxalase II (GLX2-1) identical to SP|O24495 Hydroxyacylglutathione hydrolase, mitochondrial precursor (EC 3.1.2.6) (Glyoxalase II) (Glx II) (Arabidopsis thaliana)
266735_at	1.11E-05	0.8187802	0.7712725	0.67637604	0.6385155	1.438586	1.2200174	1.2840893	1.3526064	0.8060976	0.8010692	0.7134032	0.70463353	1.2841723	1.4206259	1.2428632	1.1812199	0.726236	1.323825	0.756301	1.28222	1.82	1.70	AT2G46930	pectinacetylesterase, putative similar to pectinacetylesterase precursor GI:1431629 from [Vigna radiata]
263984_at	1.11E-05	0.7947089	0.86287594	0.7891617	0.75036556	1.5085636	1.4074538	1.4102713	1.137124	0.75972843	0.8200659	0.6915669	0.7250636	1.2740097	1.204054	1.2113584	1.2055707	0.799277	1.365853	0.749106	1.228748	1.71	1.64	AT2G42670	expressed protein
263927_s_at	1.11E-05	0.7186028	0.6752506	0.68985134	0.6278075	1.0540115	1.1963508	1.163998	1.2767954	0.79301405	0.9459886	0.8932523	0.8948093	2.2165425	1.6313931	1.613275	1.7810625	0.677878	1.172789	0.881766	1.810568	1.73	2.05	AT2G21890;AT2G21	[AT2G21890, mannitol dehydrogenase, putative similar to ELI3-2 (SP|Q02972), sinapyl alcohol dehydrogenase (Populus tremuloides) (gi:14279694); contains Pfam zinc-binding dehydrogenase domain PF00107];[AT2G21730, mannitol dehydrogenase, putative similar to ELI3-2 (SP|Q02972), sinapyl alcohol dehydrogenase (Populus tremuloides) (gi:14279694); contains Pfam zinc-binding dehydrogenase domain PF00107]
245123_at	9.27E-06	0.72784346	0.70649964	0.69100124	0.5720655	1.3034207	1.3903589	1.2053998	1.1657789	0.7786341	0.83422107	0.69187105	0.7279835	1.4439218	1.3409436	1.3244064	1.1869401	0.674352	1.26624	0.758177	1.324053	1.88	1.75	AT2G47450	chloroplast signal recognition particle component (CAO) nearly identical to CAO [Arabidopsis thaliana] GI:4102582
266910_at	8.6E-06	0.6610544	0.50882647	0.66857994	0.85333174	1.7643645	1.3508981	1.7449808	1.6546401	0.61967903	0.56118816	0.6356809	0.6721151	1.5108712	1.4190723	1.3526525	1.1466682	0.695448	1.628721	0.622166	1.357316	2.34	2.18	AT2G45920	U-box domain-containing protein contains Pfam profile PF04564: U-box domain
263958_at	8.22E-06	0.6889249	0.72880363	0.8310428	0.68568754	1.1689571	1.2960706	1.3134078	1.6212463	0.7456439	0.70512134	0.7155451	0.7053714	1.3503482	1.2041631	1.3227711	1.3701783	0.733615	1.34992	0.71792	1.311865	1.84	1.83	AT2G36240	pentatricopeptide (PPR) repeat-containing protein contains Pfam profile PF01535: PPR repeat
245228_at	8.22E-06	1.3109375	1.4043759	1.5394989	1.601955	0.62430245	0.48927101	0.5206277	0.48764443	1.5687718	1.3485248	1.3372415	1.2565395	0.7434605	0.66479664	0.50514854	0.47284183	1.464192	0.530461	1.377769	0.592062	0.36	0.43	AT3G29810	phytochelatin synthetase family protein / COBRA cell expansion protein COBL2 similar to phytochelatin synthetase [Hordeum vulgare subsp. vulgare] GI:29570314; identified in Roudier, et al, Plant Phys. (2002) 130:538-548 (PMID:12376623); contains Pfam profile PF04833: Phytochelatin synthetase-like conserved region
245059_at	8.22E-06	0.777723	0.82025135	0.9407649	0.7960454	1.4388609	1.5752817	1.4172593	1.4894435	0.67150617	0.71790284	0.7807759	0.7218733	1.1069286	1.059235	1.2212056	1.3380136	0.833696	1.480211	0.723015	1.181346	1.78	1.63	AT2G39720	zinc finger (C3HC4-type RING finger) family protein contains Pfam domain, PF00097: Zinc finger, C3HC4 type (RING finger)
263835_at	7.61E-06	0.8489801	0.8365635	0.7926526	0.7062376	1.216168	1.3315166	1.3350426	1.430986	0.6232258	0.7815087	0.69564635	0.7404031	1.1915703	1.2756276	1.253067	1.15102	0.796108	1.328428	0.710196	1.217821	1.67	1.71	AT2G40290	eukaryotic translation initiation factor 2 subunit 1, putative / eIF-2A, putative / eIF-2-alpha, putative similar to Swiss-Prot:P05198 eukaryotic translation initiation factor 2 subunit 1 (Eukaryotic translation initiation factor 2 alpha subunit) (eIF-2-alpha) (EIF- 2alpha) (EIF-2A) [Homo sapiens]
266324_at	7.61E-06	0.64356613	0.551507	0.5459281	0.49195704	1.236469	1.1002663	1.1563702	1.6259778	0.8132862	0.8997336	0.8105232	0.8829879	1.7209036	1.7105275	1.6680726	1.6047854	0.55824	1.279771	0.851633	1.676072	2.29	1.97	AT2G46710	rac GTPase activating protein, putative similar to rac GTPase activating protein 2 [Lotus japonicus] GI:3695061; contains Pfam profiles PF00620: RhoGAP domain, PF00786: P21-Rho-binding domain
266889_at	7.28E-06	0.72483426	0.77331173	0.60604596	0.6453963	1.1962045	1.3177104	1.2395848	1.3606722	0.6874907	0.80379564	0.7242735	0.7826525	1.2984301	1.4473511	1.2393152	1.199934	0.687397	1.278543	0.749553	1.296258	1.86	1.73	AT2G44640	expressed protein
267142_at	7.28E-06	0.7293665	0.631457	0.8554545	0.7001279	1.8089361	1.9789431	2.511455	2.4214358	0.54158455	0.6769678	0.6339722	0.6185911	1.1445456	1.610973	1.4373263	1.6330763	0.729101	2.180193	0.617779	1.45648	2.99	2.36	AT2G38290	ammonium transporter 2 (AMT2) identical to SP|Q9M6N7 Ammonium transporter 2 (AtAMT2) (Arabidopsis thaliana)
263868_at	7.28E-06	0.5863998	0.5032477	0.5727999	0.8065808	1.6046189	1.399303	1.2995707	1.457821	0.5042919	0.598292	0.55546194	0.58336276	1.1934192	1.2707038	1.216611	1.1973711	0.617257	1.440328	0.560352	1.219526	2.33	2.18	AT2G36840	ACT domain-containing protein contains Pfam profile ACT domain PF01842
266993_at	7.28E-06	0.4119198	0.49395832	0.5116614	0.5437424	1.3478013	1.5286825	1.4761592	2.3967896	0.6521988	0.5960829	0.6120977	0.60431975	1.5074326	1.9771119	1.835829	2.1980526	0.49032	1.687358	0.616175	1.879607	3.44	3.05	AT2G39210	nodulin family protein similar to nodulin-like protein [Arabidopsis thaliana] GI:3329368, nodule-specific protein Nlj70 [Lotus japonicus] GI:3329366
266417_at	7.28E-06	0.8531898	0.8252249	0.77216	0.7548246	1.2927487	1.3731614	1.1820734	1.4420986	0.73476785	0.7750539	0.8236496	0.72796273	1.1468103	1.2005341	1.3450328	1.3124099	0.80135	1.322521	0.765359	1.251197	1.65	1.63	AT2G38550	expressed protein contains Pfam profile PF03647: Uncharacterised protein family (UPF0136)
267423_at	7.28E-06	0.43084437	0.39892906	0.49102694	0.43648088	1.157577	1.3939898	1.2986513	1.8171967	0.8424228	0.7377422	0.568742	0.72921383	1.7542108	1.613339	1.7327431	1.4360424	0.43932	1.416854	0.71953	1.634084	3.23	2.27	AT2G35060	potassium transporter family protein similar to HAK2 [Hordeum vulgare] GI:7108599, potassium transporter HAK2p [Mesembryanthemum crystallinum] gi|14091471|gb|AAK53759; KUP/HAK/KT Transporter family member, PMID:11500563; contains Pfam profile PF02705: K+ potassium transporter
245145_at	7.21E-06	0.6739736	0.7875267	0.8073491	0.6272408	1.3257796	1.4058867	1.4968083	1.7424119	0.67385334	0.6779186	0.71747905	0.7136822	1.192651	1.3964868	1.452262	1.4078372	0.724023	1.492722	0.695733	1.362309	2.06	1.96	AT2G45440	dihydrodipicolinate synthase 2 (DHDPS2) identical to dihydrodipicolinate synthase 2 (DHDPS2) [Arabidopsis thaliana] GI:11066382
266136_at	6.05E-06	0.7917126	0.77014697	0.7695445	0.6715009	1.6171732	1.2576816	1.514268	1.2082874	0.7259303	0.7429847	0.7140871	0.7669303	1.3223022	1.3187879	1.359	1.2911372	0.750726	1.399353	0.737483	1.322807	1.86	1.79	AT2G45060	expressed protein
245144_at	5.55E-06	0.7155695	0.92461926	0.7595646	0.84460837	1.2387304	1.3427273	1.3491735	1.3839816	0.60412663	0.6226119	0.69693714	0.628422	1.0753808	1.1018358	1.1244069	1.1710714	0.81109	1.328653	0.638024	1.118174	1.64	1.75	AT2G45240	methionyl aminopeptidase, putative / methionine aminopeptidase, putative / peptidase M, putative similar to SP|Q01662 Methionine aminopeptidase 1 precursor (EC 3.4.11.18) (Saccharomyces cerevisiae); contains Pfam profile PF00557: metallopeptidase family M24
266874_at	5.5E-06	0.73282135	0.8150737	0.7035124	0.72322965	1.2894905	1.2398171	1.3150901	1.5486294	0.7753858	0.7784674	0.68748605	0.6651297	1.1849263	1.3422794	1.2322906	1.2353258	0.743659	1.348082	0.726617	1.248706	1.81	1.72	AT2G44760	expressed protein
266090_at	4.98E-06	0.8368449	0.8584886	0.9041657	0.8395611	1.3228117	1.0958343	1.2724066	1.2246453	0.76675594	0.79673076	0.7327501	0.699474	1.2339718	1.222898	1.1691053	1.1688511	0.859765	1.228924	0.748928	1.198707	1.43	1.60	AT2G38000	chaperone protein dnaJ-related weak similarity to Chaperone protein dnaJ (Swiss-Prot:Q9ZFC5) [Methylovorus sp.]
267538_at	4.98E-06	0.7002323	0.6812019	0.67565024	0.6560444	1.3774781	1.3217262	1.4901893	1.1847851	0.6901649	0.8152148	0.62166286	0.6256055	1.4642476	1.5449034	1.2812941	1.6330291	0.678282	1.343545	0.688162	1.480869	1.98	2.15	AT2G41870	remorin family protein contains Pfam domain, PF03763: Remorin, C-terminal region
266519_at	4.32E-06	0.6945624	0.61990476	0.7333168	0.73315215	1.567561	1.2361821	1.3700526	1.6106744	0.67472255	0.7554639	0.7638179	0.64667255	1.4150765	1.3636608	1.2854712	1.2719339	0.695234	1.446118	0.710169	1.334036	2.08	1.88	AT2G35155	expressed protein
266400_at	3.74E-06	0.76763606	0.74221836	0.69560534	0.6332692	1.2390392	1.4050967	1.4140064	1.3330388	0.5996658	0.7578206	0.7581506	0.7006608	1.2323639	1.3456417	1.272276	1.368356	0.709932	1.347795	0.704074	1.304659	1.90	1.85	AT2G38660	tetrahydrofolate dehydrogenase/cyclohydrolase, putative similar to SP|P09440 C-1-tetrahydrofolate synthase, mitochondrial precursor (C1-THF synthase) [Includes: Methylenetetrahydrofolate dehydrogenase (EC 1.5.1.5); Methenyltetrahydrofolate cyclohydrolase (EC 3.5.4.9); Formyltetrahydrofolate synthetase (EC 6.3.4.3)] (Saccharomyces cerevisiae); contains Pfam profiles PF02882: Tetrahydrofolate dehydrogenase/cyclohydrolase, NAD(P)-binding domain, PF00763: Tetrahydrofolate dehydrogenase/cyclohydrolase, catalytic domain
263836_at	3.16E-06	0.81434083	0.946616	0.90267354	0.8799935	1.8742567	1.9913212	1.6581737	1.5232819	0.6033468	0.57448184	0.55711955	0.48065892	1.1094309	1.1134986	1.0533842	1.110307	0.885906	1.761758	0.553902	1.096655	1.99	1.98	AT2G40330	Bet v I allergen family protein contains Pfam profile PF00407: Pathogenesis-related protein Bet v I family
263946_at	3.09E-06	0.81264305	0.7541452	0.7652016	0.739149	1.3081995	1.207402	1.1643395	1.3055323	0.8184029	0.8356603	0.76395625	0.7798024	1.3632909	1.5445318	1.3740076	1.233678	0.767785	1.246368	0.799455	1.378877	1.62	1.72	AT2G36000	mitochondrial transcription termination factor-related / mTERF-related contains Pfam profile PF02536: mTERF
266811_at	3.09E-06	0.79770917	0.89715797	0.90302974	0.9623305	1.6044693	1.473674	1.6028219	1.6577166	0.6949327	0.64642423	0.6249494	0.61644137	1.2061152	1.114243	1.229573	1.0376694	0.890057	1.58492	0.645687	1.1469	1.78	1.78	AT2G44850	expressed protein
266467_at	3.09E-06	0.7466353	0.7381225	0.8168828	0.78474486	1.6671392	1.348301	1.6270193	1.7895142	0.66401047	0.59529996	0.6582687	0.70372283	1.1832083	1.1830173	1.4168239	1.2774345	0.771621	1.607993	0.655325	1.265121	2.08	1.93	AT2G47760	ALG3 family protein contains Pfam profile: PF05208 ALG3 protein
267169_at	3.09E-06	0.7515516	0.73408103	0.6946183	0.79529357	1.5505422	1.4124336	1.4234538	1.3360292	0.81065106	0.6854711	0.7537243	0.6828345	1.4480436	1.1893489	1.2420485	1.3647846	0.743886	1.430615	0.73317	1.311056	1.92	1.79	AT2G37540	short-chain dehydrogenase/reductase (SDR) family protein contains INTERPRO family IPR002198 Short-chain dehydrogenase/reductase (SDR) superfamily
267365_at	3.09E-06	0.71977174	0.78700364	0.7618358	0.8139884	1.3161246	1.2524518	1.5162395	1.328569	0.70700246	0.8016519	0.6521696	0.71012115	1.1876193	1.227299	1.2673347	1.1860117	0.77065	1.353346	0.717736	1.217066	1.76	1.70	AT2G44530	ribose-phosphate pyrophosphokinase, putative / phosphoribosyl diphosphate synthetase, putative very strong similarity to phosphoribosyl pyrophosphate synthase [Spinacia oleracea] GI:4902849; contains Pfam profile PF00156: Phosphoribosyl transferase domain
266193_at	3.09E-06	0.8017115	0.7784406	0.7401058	0.7579334	1.6692997	1.3875619	1.354924	1.4067012	0.7027013	0.68447	0.6118094	0.5821359	1.1982883	1.3424399	1.3327428	1.2461591	0.769548	1.454622	0.645279	1.279908	1.89	1.98	AT2G39080	expressed protein
266370_at	3.09E-06	0.7347612	0.73137367	0.64689136	0.59026086	1.2304329	1.2555889	1.3108534	1.2343384	0.76956713	0.7488401	0.6719964	0.7400765	1.5620329	1.2908409	1.4293963	1.4734566	0.675822	1.257803	0.73262	1.438932	1.86	1.96	AT2G41350	expressed protein
266923_at	3.09E-06	0.81923497	0.787885	0.88835603	0.8187201	1.2310139	1.2082992	1.2605652	1.1169169	0.7993599	0.78570944	0.75704	0.79848236	1.197436	1.120479	1.2235638	1.111644	0.828549	1.204199	0.785148	1.163281	1.45	1.48	AT2G45980	expressed protein
267533_at	3.09E-06	0.69761497	0.81119156	0.837167	0.80439836	1.2977288	1.2894267	1.1953461	1.3993146	0.7144418	0.76716274	0.7026838	0.7244195	1.175428	1.2566425	1.2114623	1.1628329	0.787593	1.295454	0.727177	1.201591	1.64	1.65	AT2G42070	MutT/nudix family protein similar to SP|Q58549 ADP-ribose pyrophosphatase (EC 3.6.1.13) [Methanococcus jannaschii]; contains Pfam profile PF00293: NUDIX domain
253394_at	3.09E-06	1.9345438	1.8301908	1.4933813	1.4222091	0.5269546	0.4543041	0.57779086	0.423428	2.3513763	1.7774427	1.8080568	1.4884038	0.5550135	0.57603174	0.44981694	0.52686065	1.670081	0.495619	1.85632	0.526931	0.30	0.28	AT4G32770	tocopherol cyclase, chloroplast / vitamin E deficient 1 (VTE1) / sucrose export defective 1 (SXD1) identical to SP|Q94FY7 Tocopherol cyclase, chloroplast precursor (Vitamin E deficient 1) (Sucrose export defective 1) (Arabidopsis thaliana)
245152_at	2.46E-06	0.59268916	0.59927684	0.5907705	0.66750914	1.2287183	1.3394133	1.3720752	1.187852	0.81214786	0.7176807	0.705312	0.6700868	1.7815928	1.9442888	1.5309105	1.6910037	0.612561	1.282015	0.726307	1.736949	2.09	2.39	AT2G47490	mitochondrial substrate carrier family protein contains Pfam profile: PF00153 mitochondrial carrier protein

Supplementary Table S5. Genes differentially expressed ≥ 2-fold in the *jaz7-1D* line relative to wild-type.

AGI	TAIR description	*jaz7-1D* / WT	P-value
AT2G39030	N-acetyltransferase Activity1 (NATA1)	9.53	8.10E-05
AT1G72260	thionin (THI2.1)	7.52	1.50E-04
AT3G49620	2-oxoacid-dependent oxidase (DIN11)	6.18	1.50E-03
AT2G18050	histone H1-3 (HIS1-3)	6.02	2.70E-05
AT1G26380	FAD-binding domain-containing protein	4.14	1.80E-03
AT3G23550	MATE efflux	4.03	6.00E-04
AT5G48540	33 kDa secretory protein-related	3.79	3.00E-05
AT5G61160	transferase family protein similar to anthocyanin 5-aromatic acyltransferase	3.62	3.20E-02
AT2G38860	proteaseI (pfpI)-like protein (YLS5)	3.52	3.80E-05
AT1G21120	O-methyltransferase	3.46	1.00E-03
AT2G44480	glycosyl hydrolase family 1 protein	3.45	6.20E-05
AT5G24110	WRKY family transcription factor	3.4	8.90E-03
AT3G26830	cytochrome P450 (CYP71B15), PHYTOALEXIN DEFICIENT 3 (PAD3)	3.24	1.00E-02
AT1G05310	pectinesterase	3.22	3.00E-04
AT2G37710	lectin protein kinase	3.15	3.10E-04
AT2G37430	zinc finger (C2H2 type) family protein (ZAT11)	3.14	3.50E-02
AT2G46430	cyclic nucleotide-regulated ion channel / cyclic nucleotide-gated channel (CNGC3)	3.08	2.90E-04
AT2G46450	cyclic nucleotide-regulated ion channel, putative (CNGC12)	3.08	3.40E-05
AT2G39210	nodulin family protein	3.05	1.80E-05
AT2G39420	esterase/lipase/thioesterase	3.02	5.80E-04
AT1G21100	O-methyltransferase	2.93	7.20E-04
AT3G16530	legume lectin	2.88	3.00E-04
AT2G35980	harpin-induced family protein (YLS9) / HIN1	2.84	9.80E-03
AT2G47130	short-chain dehydrogenase/reductase (SDR)	2.82	7.90E-05
AT2G35930	U-box domain-containing protein similar to immediate-early fungal elicitor protein	2.8	1.10E-02
AT2G36970	UDP-glucoronosyl/UDP-glucosyl transferase	2.75	8.20E-05
AT2G46650	cytochrome b5	2.64	7.00E-03
AT1G66690	S-adenosyl-L-methionine:carboxyl methyltransferase	2.64	2.40E-02
AT4G16260	glycosyl hydrolase family 17 protein similar to glucan endo-1,3-beta-glucosidase	2.63	3.40E-02
AT2G39660	protein kinase	2.57	9.40E-04

AT2G39200	seven transmembrane MLO family protein / MLO-like protein 12 (MLO12)	2.56	1.60E-04
AT1G21120	O-methyltransferase	2.56	3.90E-03
AT2G37950	zinc finger (C3HC4-type RING finger)	2.51	4.60E-04
AT2G40460	proton-dependent oligopeptide transport (POT) family protein	2.5	9.70E-05
AT2G37040	phenylalanine ammonia-lyase 1 (PAL1)	2.48	4.20E-04
AT5G57220	cytochrome P450	2.47	1.80E-02
AT1G79680	wall-associated kinase	2.47	2.20E-02
AT2G29720	monooxygenase	2.47	1.00E-03
AT2G37940	expressed protein	2.47	6.90E-04
AT2G41180	sigA-binding protein-related	2.44	2.80E-03
AT2G40150	expressed protein	2.43	9.80E-05
AT1G26420	FAD-binding domain-containing protein	2.43	1.60E-04
AT2G40110	yippee family protein	2.4	2.30E-03
AT2G47490	NAD+ TRANSPORTER 1 (NDT1)	2.39	7.30E-06
AT2G38640	expressed protein contains	2.38	6.50E-04
AT2G38020	vacuoleless1 (VCL1), mangled (MAN)	2.37	6.50E-05
AT5G24420	glucosamine/galactosamine-6-phosphate isomerase	2.37	2.80E-02
AT4G19430	expressed protein	2.37	2.20E-02
AT3G05360	disease resistance family protein / LRR family protein	2.36	2.10E-02
AT2G46620	AAA-type ATPase	2.36	5.40E-04
AT2G38290	ammonium transporter 2 (AMT2)	2.36	1.80E-05
AT1G67980	caffeoyl-CoA 3-O-methyltransferase	2.33	3.50E-02
AT2G41250	haloacid dehalogenase-like hydrolase	2.3	5.40E-04
AT4G19370	hypothetical protein	2.29	1.30E-02
AT2G43200	dehydration-responsive family protein	2.28	3.80E-04
AT2G35060	potassium transporter	2.27	1.70E-05
AT4G21390	S-locus lectin protein kinase	2.27	5.40E-03
AT1G80130	expressed protein	2.27	7.20E-03
AT3G18710	U-box domain-containing protein similar to immediate-early fungal elicitor protein	2.24	4.40E-02
AT2G35710	glycogenin glucosyltransferase (glycogenin)	2.24	4.70E-02
AT2G38790	expressed protein	2.22	8.90E-03
AT2G46690	SMALL AUXIN UPREGULATED RNA 32 (SAUR32)	2.21	7.50E-03
AT2G43590	chitinase	2.21	1.50E-04

AT2G40010	60S acidic ribosomal protein P0 (RPP0A)	2.21	2.70E-05
AT2G43520	trypsin inhibitor	2.19	4.90E-02
AT2G45920	U-box domain-containing protein	2.18	2.20E-05
AT1G02360	chitinase	2.18	4.10E-03
AT2G36840	ACT domain-containing protein	2.18	1.80E-05
AT2G45280	DNA repair family protein	2.17	4.50E-05
AT2G41870	remorin family protein	2.15	1.20E-05
AT2G40270	protein kinase	2.15	4.00E-03
AT2G42690	lipase	2.15	4.20E-05
AT2G39690	expressed protein	2.14	1.20E-04
AT2G39480	ABC transporter family protein related to multi drug resistance proteins and P-glycoproteins	2.14	5.10E-04
AT2G38410	VHS domain-containing protein	2.13	5.70E-05
AT2G41890	curculin-like (mannose-binding)	2.13	2.70E-04
AT2G26440	pectinesterase	2.12	2.30E-03
AT3G09410	pectinacetylesterase	2.12	1.10E-03
AT2G41700	ABC transporter family protein related to multi drug resistance proteins and P-glycoproteins	2.12	3.10E-04
AT2G41980	seven in absentia (SINA) family protein	2.12	2.00E-04
AT2G39870	expressed protein	2.1	7.10E-05
AT2G36400	expressed protein	2.1	7.10E-05
AT2G36470	expressed protein	2.09	3.20E-05
AT2G43800	formin homology 2 domain-containing protein	2.09	1.10E-04
AT4G39950	cytochrome P450 (CYP79B2)	2.08	7.60E-03
AT2G44790	uclacyanin II	2.07	3.90E-02
AT1G51280	disease resistance protein (TIR class)	2.07	2.90E-04
AT2G39360	protein kinase	2.07	3.60E-03
AT2G46530	transcriptional factor B3 family protein / auxin-responsive factor 11 (ARF11)	2.07	4.30E-05
AT2G42760	expressed protein	2.07	1.80E-02
AT1G74590	glutathione S-transferase	2.06	6.70E-03
AT2G38050	3-oxo-5-alpha-steroid 4-dehydrogenase, de-etiolated 2 (DET2), dwarf6 (DWF6)	2.06	5.10E-04
AT2G36220	expressed protein	2.06	3.20E-03
AT2G21890	CINNAMYL ALCOHOL DEHYDROGENASE HOMOLOG 3 (CAD3)	2.05	2.20E-05
AT2G46720	fatty acid elongase 3-ketoacyl-CoA synthase, 3-KETOACYL-COA SYNTHASE 13 (KCS13)	2.05	2.60E-05
AT2G37970	SOUL heme-binding family protein, SOUL-1	2.05	8.30E-03

AT2G41640	expressed protein	2.05	3.00E-02
AT2G44660	ALG6, ALG8 glycosyltransferase	2.03	5.40E-04
AT3G04720	hevein-like protein (HEL), pathogenesis-related 4 (PR4)	2.02	3.10E-03
AT2G34810	FAD-binding domain-containing protein	2.02	2.80E-02
AT2G38280	AMP deaminase, putative / myoadenylate deaminase	2.02	1.10E-03
AT2G37240	expressed protein	2.02	3.70E-04
AT2G43130	Ras-related protein (ARA-4) / small GTP-binding protein	2.01	6.80E-04
AT2G34640	expressed protein	2.01	2.60E-05
AT2G38870	protease inhibitor	2.01	6.50E-03
AT3G22060	receptor protein kinase	2.01	1.30E-03
AT2G41990	expressed protein	2.01	5.90E-04
AT3G12910	expressed protein	2.01	1.10E-03
AT2G34620	mitochondrial transcription termination factor-related	2.01	6.20E-03
AT2G45010	expressed protein	2	6.20E-05
AT1G63530	hypothetical protein	2	2.30E-03
AT2G38740	haloacid dehalogenase-like hydrolase	2	7.50E-04
AT2G46420	expressed protein	2	4.20E-04

Supplementary Table S6. Genes differentially expressed ≤ 2-fold in the *jaz7-1D* line relative to wild-type.

AGI	TAIR description	*jaz7-1D* / WT	P-value
AT4G34135	UDP-glucoronosyl/UDP-glucosyl transferase	0.24	1.90E-03
AT2G29490	glutathione S-transferase	0.27	9.60E-03
AT4G32770	tocopherol cyclase, chloroplast / vitamin E deficient 1 (VTE1) / sucrose export defective 1 (SXD1)	0.28	7.30E-06
AT1G05680	UDP-glucoronosyl/UDP-glucosyl transferase	0.34	2.40E-02
AT5G65080	MADS-box family protein, mads affecting flowering 5 (MAF5)	0.37	1.10E-04
AT1G62770	invertase/pectin methylesterase inhibitor	0.38	7.90E-05
AT1G05560	UDP-glucose transferase (UGT75B2)	0.38	4.00E-03
AT1G17170	glutathione S-transferase	0.41	1.60E-03
AT5G16990	NADP-dependent oxidoreductase	0.41	4.40E-03
AT1G24575	expressed protein	0.42	8.80E-04
AT4G37220	stress-responsive protein, similar to cold acclimation WCOR413-like protein	0.42	2.30E-03
AT3G29810	phytochelatin synthetase family protein / COBRA cell expansion protein COBL2	0.43	2.00E-05
AT5G62360	invertase/pectin methylesterase inhibitor	0.43	9.00E-05
AT5G09220	amino acid permease 2 (AAP2)	0.43	6.10E-05
AT1G09500	cinnamyl-alcohol dehydrogenase	0.44	2.10E-02
AT1G09240	nicotianamine synthase	0.46	1.40E-02
AT4G12470	protease inhibitor/seed storage/lipid transfer protein (LTP)	0.46	1.50E-03
AT4G14130	xyloglucan:xyloglucosyl transferase	0.47	2.60E-02
AT3G28310	expressed protein	0.47	3.60E-03
AT1G27590	expressed protein	0.47	2.90E-05
AT1G53100	glycosyltransferase family 14	0.49	3.20E-04
AT4G30140	GDSL-motif lipase/hydrolase	0.49	1.30E-02
AT1G34760	14-3-3 protein GF14 omicron (GRF11)	0.5	1.60E-04
AT2G29420	glutathione S-transferase	0.5	3.10E-02
AT1G64950	cytochrome P450 (CYP89A)	0.5	2.90E-02

Supplementary Table S7. List of genes differentially regulated by MeJA treatment from the microarray.

Genes were obtained using a 2-way ANOVA with a p-value cut-off of 0.05 and the addition of the Benjamini and Hochberg False Discovery rate. The list represents the genes that were differentially expressed by the MeJA treatment from the entire microarray data set and are calculated irrespective of the genotype.

All samples

Treatment ()*S		MeJA 6h	MeJA 6h	MeJA 6h	MeJA 6h	MeJA 6h	MeJA 6h	MeJA 6h	MeJA 6h	Control 6h	Control 6h	Control 6h	Control 6h	Control 6h	Control 6h	Control 6h	Control 6h								
Genotype ()*S		Col-0	Col-0	Col-0	Col-0	*jaz7-1D*	*jaz7-1D*	*jaz7-1D*	*jaz7-1D*	Col-0	Col-0	Col-0	Col-0	*jaz7-1D*	*jaz7-1D*	*jaz7-1D*	*jaz7-1D*	averages		averages		Fold change			
Replicate ()*		rep 1	rep 2	rep 3	rep 4	rep 1	rep 2	rep 3	rep 4	rep 1	rep 2	rep 3	rep 4	rep 1	rep 2	rep 3	rep 4	MeJA 6h		Control 6h		MeJA/control			
Systematic	P-value	Normalized	Normalized	Normalized	Normalized	Normalized	Normalized	Normalized	Normalized	Normalized	Normalized	Normalized	Normalized	Normalized	Normalized	Normalized	Normalized	Col-0	*jaz7-1D*	Col-0	*jaz7-1D*	Col-0	*jaz7-1D*	ATG#	TAIR description
262483_at	0.05	0.8433662	0.8031145	0.8699997	0.6961471	0.72629625	0.8738524	0.84519047	1.3029494	1.5553093	1.0539494	1.1425427	0.97857916	1.1014222	1.1994877	1.0363613	1.021421	0.803157	0.937072	1.182595	1.089673	0.68	0.86	AT1G17220	translation initiation factor IF-2, chloroplast, putative similar to SP|P57997|IF2C_PHAVU Translation initiation factor IF-2, chloroplast precursor (PvIF2cp) (Phaseolus vulgaris)
258541_at	0.0499	1.2029192	1.0966296	1.0701537	1.0276359	0.9343227	1.1579607	1.0372587	0.95729965	0.9014221	0.89069986	1.0212094	0.8993449	0.7787879	1.011816	0.98818403	0.79950476	1.099335	1.02171	0.928169	0.894573	1.18	1.14	AT3G07000	DC1 domain-containing protein contains Pfam profile PF03107: DC1 domain
265499_at	0.0499	0.57548094	0.6571208	1.1375049	0.73622465	0.8723025	0.9155858	0.81890565	1.1770767	1.6178318	1.5026704	1.4927194	1.0844141	1.3279693	0.8081613	0.89639515	1.7175779	0.776583	0.945968	1.424409	1.187526	0.55	0.80	AT2G15480	UDP-glucoronosyl/UDP-glucosyl transferase family protein contains Pfam profile: PF00201 UDP-glucoronosyl and UDP-glucosyl transferase
256298_at	0.0498	1.0832158	1.1479154	1.0465711	1.045256	0.975372	1.0922662	1.058589	0.9464356	0.9435783	1.024628	1.03892	0.87624264	0.97516745	0.9618446	0.89332795	0.91767657	1.08074	1.018166	0.970842	0.937004	1.11	1.09	AT1G69540	MADS-box family protein contains Pfam profile: PF00319: SRF-type transcription factor (DNA-binding and dimerisation domain)
255702_at	0.0498	1.0858669	1.0693136	1.1185335	0.92982584	1.0283787	1.098193	1.0133153	1.5558977	0.84704626	1.0957521	0.82428545	0.8579474	0.8242194	0.895204	0.98668456	0.8005868	1.050885	1.173946	0.906258	0.876674	1.16	1.34	AT4G00230	subtilisin-like serine endopeptidase (XSP1) identical to subtilisin-type serine endopeptidase XSP1 GI:6708179 from (Arabidopsis thaliana)
267421_at	0.0498	0.71841913	0.79834664	0.84627813	0.8443833	1.5156372	1.7259855	1.4719415	2.3165896	0.59129256	0.748843	0.7320659	0.6908434	1.1537218	1.4834744	1.3870518	1.2329402	0.801857	1.757538	0.690761	1.314297	1.16	1.34	AT2G35040	AICARFT/IMPCHase bienzyme family protein similar to SP|P12048 Bifunctional purine biosynthesis protein purH (Includes: Phosphoribosylaminoimidazolecarboxamide formyltransferase (EC 2.1.2.3) (AICAR transformylase); IMP cyclohy [illegible]
246617_at	0.0498	1.2288963	0.9993347	1.3333334	1.0364032	1.5670404	1.1581625	1.0711344	0.8499806	0.8724577	0.80448145	1.0649855	0.9000823	1.0006654	0.8356484	0.7849828	0.75157964	1.149492	1.161579	0.910502	0.843219	1.26	1.38	AT5G36270	dehydroascorbate reductase, putative similar to dehydroascorbate reductase (Spinacia oleracea) gi:10952511 gb:AF195783, PMID:11148269
251934_at	0.0497	1.6012981	1.5517626	1.4530545	1.2552134	1.2025659	0.93688464	0.89853054	0.71177274	1.0631155	0.6735418	1.1403896	1.0947272	0.8809406	0.6340886	0.6056199	0.8617467	1.465332	0.937438	0.992944	0.745599	1.48	1.26	AT3G54070	ankyrin repeat family protein contains ankyrin repeats, Pfam domain PF00023
261464_at	0.0497	1.1282395	0.94362026	0.9542486	1.159428	1.153182	1.1460261	1.0572467	1.4524051	0.9772981	1.022702	0.9756281	0.8766289	0.9046481	1.0237739	0.9694409	0.8808938	1.046384	1.202215	0.963064	0.944689	1.09	1.27	AT1G07740;AT1G [illegible]	[AT1G07740, pentatricopeptide (PPR) repeat-containing protein contains Pfam profile PF01535: PPR repeat; possible frameshift prevents accurate annotation of the gene product];[AT1G07730, disease resistance-responsive family pr [illegible]
256624_at	0.0497	1.0057169	1.1247684	1.3902984	1.1999754	1.0778444	0.9109063	1.0497963	0.9412725	1.0870266	1.1508498	0.93157023	0.99428314	0.8862019	0.7466173	0.7706115	0.8589056	1.18019	0.994955	1.040932	0.815584	1.13	1.22	AT3G19990	expressed protein
246579_at	0.0496	1.0462933	1.0252054	1.0351486	1.1044785	1.1520509	1.0422511	1.054899	0.8900563	0.88318676	0.9455804	0.990389	0.8980654	0.96374667	1.0096109	0.8857461	0.88888747	1.052781	1.034814	0.929305	0.936998	1.13	1.10	AT1G31620	hypothetical protein
262780_at	0.0496	0.9176502	1.2071917	1.1479102	1.010095	0.9722964	0.6372937	0.6125159	0.62998235	1.4329877	1.0861388	1.302867	1.0629207	1.223904	0.98990494	0.9447451	0.93757415	1.070712	0.713022	1.221229	1.024032	0.88	0.70	AT1G13090	cytochrome P450 71B28, putative (CYP71B28) identical to Cytochrome P450 (SP:Q9SAE3) (Arabidopsis thaliana); strong similarity to gb|X97864 cytochrome P450 from Arabidopsis thaliana and is a member of the PF|00067 Cytochrom [illegible]
252981_at	0.0496	1.1634371	1.0754484	1.1918072	1.1587559	1.0595685	1.1985923	1.2597104	0.8098872	0.9575636	1.0110097	0.86267483	0.83474296	0.89122474	0.9349339	0.98899037	0.9272912	1.147362	1.08194	0.916498	0.93561	1.25	1.16	AT4G38260	expressed protein contains Pfam PF05742: Protein of unknown function (DUF833)
262575_at	0.0496	1.0223389	1.098851	1.019506	1.0853702	1.211221	0.98628974	1.1096517	0.9700974	1.0202957	0.8265574	0.9471189	0.944678	0.91070706	0.85523474	1.0137103	0.96807355	1.056517	1.069315	0.934663	0.936931	1.13	1.14	AT1G15210	ABC transporter family protein Similar to gb|Z70524 GI:1514643 PDR5-like ABC transporter from Spirodela polyrrhiza and is a member of the PF|00005 ABC transporter family. ESTs gb|N97039 and gb|T43169 come from this gene
265788_at	0.0495	1.1638706	1.0933036	1.1033021	0.98222846	1.281471	1.01826	1.0617932	1.0061399	0.9430519	0.99386007	1.1518264	0.97737765	0.90985763	0.81007725	0.9105395	0.91737145	1.085676	1.091916	1.016529	0.886961	1.07	1.23	AT2G01200	auxin-responsive AUX/IAA family protein contains Pfam profile: PF02309 AUX/IAA family
255683_at	0.0495	1.0177749	1.1120538	1.2603713	1.0802473	0.9572159	1.0088854	1.0459441	1.4912219	0.77698374	0.8790569	1.0221	0.99111456	0.72182643	0.902441	0.9450144	0.90678823	1.117612	1.125817	0.917314	0.869018	1.22	1.30	AT4G00570	malate oxidoreductase, putative similar to NAD-dependent malic enzyme 59 kDa isoform, mitochondrial precursor (EC 1.1.1.39) (NAD-ME) (SP:P37225) (Solanum tuberosum)
260651_at	0.0494	1.3553116	1.2998786	1.5077839	1.5764617	1.2565233	1.1055267	1.4215728	0.66199976	0.8661733	0.900166	0.95254135	0.9852099	0.9477768	0.7717804	1.01479	0.9435665	1.434859	1.111406	0.926023	0.919478	1.55	1.21	AT1G32460	expressed protein
249328_at	0.0494	1.0642797	0.91081893	1.3462498	1.1982459	1.2175484	1.1220167	1.1506749	1.0046161	0.9048388	1.1895436	0.99538374	0.8125631	0.91983044	0.87650216	0.8804901	0.7865201	1.129899	1.123714	0.975582	0.865836	1.16	1.30	AT5G40820	FAT domain-containing protein / phosphatidylinositol 3- and 4-kinase family protein similar to Atr protein (Xenopus laevis) GI:11385422; contains Pfam profiles PF00454 Phosphatidylinositol 3- and 4-kinase, PF02259 FAT domain, PF02 [illegible]
251645_at	0.0494	1.0901946	1.1603165	1.0843382	1.0900189	0.9112513	0.9076285	1.1834371	1.1747772	1.1427407	0.9681206	1.0318793	0.95145184	0.8103639	0.7808191	0.90143585	0.85226226	1.106217	1.044274	1.023548	0.83622	1.08	1.25	AT3G57790	glycoside hydrolase family 28 protein / polygalacturonase (pectinase) family protein weak similarity to SP|P15922 Exo-poly-alpha-D-galacturonosidase precursor (EC 3.2.1.82) (Exo-PG) (Erwinia chrysanthemi); contains PF00295: Glycos [illegible]
263382_at	0.0493	0.74502194	0.96693933	0.9043235	0.72219044	1.0034049	1.0851228	0.9974841	1.1139432	1.0025159	0.83610064	0.75934625	0.8926892	1.347917	1.3933793	1.298848	1.4726954	0.834619	1.049989	0.872663	1.37821	0.96	0.76	AT2G40230	transferase family protein similar to taxadienol acetyl transferase from Taxus cuspidata (gi:6978038); contains Pfam transferase family domain PF002458
250498_at	0.0493	1.0031047	0.82760674	1.0218803	0.9862296	0.9968952	0.782511	0.7439698	0.9099394	1.2189662	1.2563668	1.0933137	0.93349034	1.0593325	1.0721359	0.9851721	1.0114686	0.959705	0.858329	1.125534	1.032027	0.85	0.83	AT5G09660	malate dehydrogenase, glyoxysomal identical to SP|Q9ZP05; identical to cDNA microbody NAD-dependent malate dehydrogenase GI:3929650
248948_at	0.0493	1.0488131	1.1919956	1.2638264	0.94853055	1.2655189	0.9305017	1.2166702	1.2448834	0.8869854	0.9764046	0.88237697	1.0145926	1.0068172	0.97865903	0.93318284	0.92425054	1.113291	1.164394	0.94009	0.975727	1.18	1.19	AT5G45560	pleckstrin homology (PH) domain-containing protein / lipid-binding START domain-containing protein weak similarity to SP|P79245 Steroidogenic acute regulatory protein, mitochondrial precursor (StAR) (Ovis aries); contains Pfam pro [illegible]
265684_at	0.0493	0.9731295	0.98874915	1.0921994	1.072719	1.0977973	1.0729268	1.084442	1.0792694	0.97344315	0.99086446	1.0091355	0.96380514	0.957778	1.0823281	0.8987645	0.9109563	1.031699	1.083609	0.984312	0.962457	1.05	1.13	AT2G24410	hypothetical protein
265654_s_at	0.0493	1.0807257	1.0324031	1.152661	1.0229982	1.0203454	1.011711	0.95274127	0.92592365	0.9994839	1.0005159	1.024831	0.8971078	0.90675104	0.9022728	0.7698227	0.924648	1.072197	0.97768	0.980485	0.875874	1.09	1.12	AT5G28785	hypothetical protein
249309_at	0.0493	0.50505364	0.7696916	0.82519245	0.71000344	0.5098652	0.5149222	0.7597827	1.4948311	1.3400629	1.0204188	1.1097858	0.99473137	1.1708602	1.1264095	1.0052686	1.3898476	0.702485	0.81985	1.11625	1.173096	0.63	0.70	AT5G41410	homeodomain protein (BEL1) identical to cDNA homeobox protein (BEL1) GI:28202124
264238_at	0.0493	1.0754098	1.609852	1.6530362	1.4860163	1.1369904	0.9096685	0.9908851	0.94807726	0.91664857	0.70980597	1.2214257	1.0639718	0.8716548	0.724213	1.0091149	0.83190984	1.456079	0.996405	0.977963	0.859223	1.49	1.16	AT1G54740	expressed protein
263664_at	0.0491	1.2821147	0.9815092	1.0184908	0.93671703	0.80086887	0.8865973	0.6141906	0.46469977	1.4183488	1.4179378	1.4595358	1.0535729	0.8769562	1.1276778	1.112567	0.84786415	1.054708	0.691589	1.337349	0.991266	0.79	0.70	AT1G04250	auxin-responsive protein / indoleacetic acid-induced protein 17 (IAA17) identical to SP|P93830 Auxin-responsive protein IAA17 (Indoleacetic acid-induced protein 17) (Arabidopsis thaliana); ESTs gb|H36782 and gb|F14074 come from [illegible]
251251_at	0.0491	1.0390164	0.91336787	0.96839255	1.1277726	0.8282444	0.98353255	0.8785584	0.7834541	1.0308715	1.2645206	1.2148865	1.2036543	0.8976201	1.0917318	1.0164673	0.96903235	1.012137	0.868447	1.178483	0.993713	0.86	0.87	AT3G62220	serine/threonine protein kinase, putative similar to Pto kinase interactor 1 (Pti1)(Lycopersicon esculentum) gi|3668069|gb|AAC61805
248369_at	0.0491	1.1302412	1.1188421	1.4157395	1.1344775	0.88398194	0.9987099	1.0542494	1.1884766	0.9855909	1.1231546	0.92355984	1.0012901	0.801112	0.8264108	0.9830264	0.8964276	1.199825	1.031354	1.008399	0.876744	1.19	1.18	AT5G52040	arginine/serine-rich splicing factor RSP41 (RSP41) nearly identical to SP|P92966 Arginine/serine-rich splicing factor RSP41 (Arabidopsis thaliana)
254879_at	0.0491	1.0495399	1.012491	1.0670488	0.96630067	1.0225245	0.98766357	1.1633848	1.3977678	1.0123365	1.0421523	0.87739563	0.88962066	0.83705145	0.9823109	0.8874922	0.82904464	1.023845	1.142835	0.955376	0.883975	1.07	1.29	AT4G11670	expressed protein contains Pfam PF05664: Protein of unknown function (DUF810)
246573_at	0.0491	1.6699615	1.6169444	0.9975646	1.0024353	1.6221563	1.0118552	1.0502595	1.9909931	0.9645347	1.1190842	0.9582241	0.69569296	0.7645324	0.9728916	0.78270185	0.92537624	1.321726	1.418816	0.934384	0.861376	1.41	1.65	AT1G31680	copper amine oxidase family protein contains similarity to amine oxidase (copper-containing) precursor (Pisum sativum) SWISS-PROT:Q43077

[illegible]

249742_at	0.0429	1.0292326	1.0170476	1.1149759	0.9302678	0.9052574	0.89011526	0.9167456	0.91031504	1.3462876	1.1649337	1.1101314	1.0941178	1.1480182	0.9829522	0.97684443	0.93882823	1.022881	0.905608	1.178868	1.011661	0.87	0.90	AT5G24490	30S ribosomal protein, putative similar to SP|P19954 Plastid-specific 30S ribosomal protein 1, chloroplast precursor (CS-S5) (CS5) (S22) (Ribosomal protein 1) (PSRP-1) (Spinacia oleracea); contains Pfam profile PF02482: Sigma 54 modulation protein / S30EA ribosomal protein
256960_at	0.0429	0.9685892	0.82331795	0.8485998	0.83157116	0.85845506	0.75844294	0.8481207	1.2074476	1.1758099	1.1686825	0.9984842	1.1934412	1.0298008	1.001516	1.0240452	1.0022273	0.86802	0.918117	1.134104	1.014397	0.77	0.91	AT3G13510	expressed protein Pfam profile PF03080: Arabidopsis proteins of unknown function
253305_at	0.0429	0.91042185	0.7182902	0.65289336	0.45659414	1.0333691	0.80608165	0.69013536	0.20009182	1.8378305	1.3777775	1.3688952	1.0739574	1.6878757	1.0010239	1.0305319	0.99897623	0.68455	0.682419	1.414615	1.179602	0.48	0.58	AT4G33666;AT4G	[AT4G33666, expressed protein];[AT4G33670, L-galactose dehydrogenase (L-GalDH) identical to L-galactose dehydrogenase [Arabidopsis thaliana] GI:16555790; similar to L-fucose dehydrogenase [Pseudomonas sp.] GI:829054; contains Pfam profile PF00248: oxidoreductase, aldo/keto reductase family]
252212_at	0.0429	0.9812555	1.5882269	0.879582	1.135422	1.1379516	1.7047561	1.064425	1.4646257	0.8057847	1.0187447	0.776332	0.7818855	0.9337821	0.80866104	0.66848624	1.1268488	1.146122	1.34294	0.845687	0.884445	1.36	1.52	AT3G50310	protein kinase-related contains eukaryotic protein kinase domain, INTERPRO:IPR000719
253675_at	0.0429	1.0885518	1.1038859	1.0674473	1.2363324	1.0165913	1.0236492	0.98959494	1.5384116	0.9094084	0.7901095	0.98632866	0.94041425	0.8131184	0.9653681	1.0104052	0.8164293	1.124054	1.142062	0.906565	0.90133	1.24	1.27	AT4G29560	expressed protein
247027_at	0.0429	1.1783571	0.8878341	1.0141805	1.2529461	1.0086833	1.0959798	1.0627707	1.1354054	1.0094817	0.7829524	0.8942933	0.8770975	0.99131656	0.9610148	0.81181115	0.90612495	1.083329	1.07571	0.890956	0.917567	1.22	1.17	AT5G67090	subtilase family protein contains similarity to subtilisin-like protease ag12 GI:757522 from [Alnus glutinosa]
255285_at	0.0429	1.116472	0.8313073	0.7383001	0.8865632	0.9952831	0.66740626	0.7421437	0.49201298	1.5756959	1.0047169	1.0303962	1.050193	1.1964836	1.200717	1.022393	0.8983646	0.893161	0.724212	1.16525	1.099451	0.77	0.66	AT4G04630	expressed protein contains Pfam profile PF04520: Protein of unknown function, DUF584
253654_at	0.0427	1.2378073	1.0730969	0.995114	1.1331123	1.2678679	1.1658282	1.0048859	1.8618226	0.44153875	0.6158681	0.88387203	0.8057935	0.54969174	0.8905821	0.97293824	1.245596	1.109783	1.325101	0.686768	0.914702	1.62	1.45	AT4G30060	expressed protein contains Pfam profile PF03267: Arabidopsis protein of unknown function, DUF266
248036_at	0.0427	0.9659785	1.670613	1.4991194	1.3518093	1.0079097	1.0173159	0.96192044	1.3213859	0.8088177	0.5177462	1.0430145	0.9209034	0.7214429	0.8454796	0.7954486	1.0327543	1.37188	1.07733	0.840417	0.848781	1.63	1.27	AT5G55920	nucleolar protein, putative similar to SP|P46087 Proliferating-cell nucleolar antigen p120 (Proliferation-associated nucleolar protein p120) (Homo sapiens), SP|P40991 Nucleolar protein NOP2 (Saccharomyces cerevisiae); contains Pfam profile PF01189: NOL1/NOP2/sun family
265253_at	0.0427	1.0047739	1.2843202	0.93758065	1.0717918	1.2142982	1.4566693	0.99522626	1.6057585	0.8200967	0.93900347	0.9830242	0.8963173	0.8165859	1.0282295	1.0521401	0.9081752	1.074617	1.317988	0.90961	0.951283	1.18	1.39	AT2G02020	proton-dependent oligopeptide transport (POT) family protein contains Pfam profile: PF00854 POT family
257806_at	0.0427	1.0902414	1.0564469	1.3045433	1.1434342	1.2640973	1.0624355	1.0119005	0.9372451	0.9880996	0.9848538	0.9263287	0.93908334	0.9594581	1.0414853	0.87294745	0.798386	1.148666	1.06892	0.959591	0.918069	1.20	1.16	AT3G18670	ankyrin repeat family protein contains ankyrin repeats, Pfam domain PF00023
247510_at	0.0427	0.8009774	0.91648346	1.0338699	0.9515212	0.9617028	0.75251704	0.67646194	0.9870067	1.3826747	1.2043375	1.0129935	1.1371431	1.0360402	1.0898057	0.89440656	1.0386496	0.925713	0.844422	1.184287	1.014751	0.78	0.83	AT5G62030	diphthamide synthesis DPH2 family protein contains InterPro accession IPR002728: Diphthamide synthesis DPH2 protein
251471_at	0.0427	1.0937837	0.91771406	1.1011455	1.0860492	1.3601215	1.1478525	1.0322578	1.4757962	0.9543066	1.1553173	0.8665753	0.89221346	0.96774226	0.90759593	0.9613104	0.81561786	1.049673	1.254007	0.967103	0.913067	1.09	1.37	AT3G59570	RabGAP/TBC domain-containing protein similar to GTPase activating protein [Yarrowia lipolytica] GI:2370595; contains Pfam profile PF00566: TBC domain
245879_at	0.0427	1.0530959	1.1853393	1.2121019	1.2228214	0.9894287	1.321748	1.24921	2.1295955	0.99588037	0.74169207	1.0000161	0.999839	0.9184065	0.9849709	0.9311797	0.9338797	1.16834	1.422496	0.934393	0.942109	1.25	1.51	AT5G09420	chloroplast outer membrane translocon subunit, putative similar to component of chloroplast outer membrane translocon Toc64 [Pisum sativum] GI:7453538; contains Pfam profiles PF01425: Amidase, PF00515: TPR Domain
259729_at	0.0427	1.5143929	1.567552	2.187712	1.3840556	1.2227832	1.3843828	0.91623324	0.98942256	0.5849101	0.85599625	0.9938281	1.7230656	0.80741686	0.7579188	1.0061718	0.80276877	2.163428	1.128205	1.03945	0.843569	2.08	1.34	AT1G77640	AP2 domain-containing transcription factor, putative Similar to DREB1A (GP:3660548) [Arabidopsis thaliana]
252740_at	0.0427	1.2643362	1.2971203	1.2561653	1.5680511	0.87918043	0.99149364	0.9604975	1.3876384	0.73322743	1.2439891	1.0770589	1.0085064	0.5946121	0.78913957	0.8179622	0.8893477	1.346418	1.054701	1.015695	0.772765	1.33	1.36	AT3G43270	pectinesterase family protein contains Pfam profile: PF01095 pectinesterase
263974_at	0.0427	1.1330739	0.92767465	0.9719096	0.9385167	1.1723858	1.3222834	1.1534259	1.1715257	0.9737925	0.92150956	1.0262073	0.8839134	1.0353662	0.9594663	0.91176075	1.0430415	0.992794	1.204905	0.951356	0.987409	1.04	1.22	AT2G42720	F-box family protein contains F-box domain Pfam:PF00646
250154_at	0.0427	1.0657543	1.0111306	0.97879434	1.1156107	1.0652547	1.1822348	1.2243378	0.96776897	0.9122798	0.8694473	0.98886937	0.8478339	1.0705072	1.0169771	0.9223757	0.88261366	1.042772	1.109899	0.904608	0.973118	1.15	1.14	AT5G15140	aldose 1-epimerase family protein similar to SP|P05149 Aldose 1-epimerase precursor (EC 5.1.3.3) (Mutarotase) from Acinetobacter calcoaceticus; contains Pfam profile PF01263 Aldose 1-epimerase
258025_at	0.0427	1.0260624	0.7778895	0.6904353	0.6172809	0.7987567	0.9341712	0.7891034	1.2204748	1.3640022	1.1658487	1.0534409	0.9739375	1.2332262	1.1767846	0.97267264	1.0680671	0.777917	0.935627	1.139307	1.112688	0.68	0.84	AT3G19490;AT3G	[AT3G19490, sodium hydrogen antiporter, putative similar to NhaD [Vibrio parahaemolyticus] gi|3123728|dbj|BAA25994; Na+/H+ antiporter (NhaD) family member, PMID:11500563];[AT3G19480, D-3-phosphoglycerate dehydrogenase, putative / 3-PGDH, putative similar to SP:O04130 from [Arabidopsis thaliana]]
265663_at	0.0427	1.0050286	1.0045984	0.802586	0.9958604	0.8273851	0.855705	0.82869387	0.5408394	1.1214	1.4603791	1.0040395	1.2329199	1.1124307	0.9184059	1.1139101	0.8980342	0.952043	0.763156	1.204685	1.010695	0.79	0.76	AT2G24290	expressed protein
259512_at	0.0427	1.0532533	1.0667658	1.4393195	1.2778027	1.168595	0.9020591	1.0750979	1.8992125	0.9805703	0.8176529	0.9233264	1.0194298	0.78630733	0.76508015	0.93828876	0.85735315	1.209285	1.261241	0.935245	0.83676	1.29	1.51	AT1G12360	cytokinesis-related Sec1 protein (KEULE) similar to cytokinesis-related Sec1 protein KEULE [Arabidopsis thaliana] gi|12659318|gb|AAK01291; contains Pfam domain, PF00995: Sec1 family
247088_at	0.0427	1.0306188	1.0016081	1.0754082	1.2587582	1.1276411	1.1008863	0.95971406	0.99709576	0.9983918	1.0115834	1.0204142	0.8504521	0.9658624	0.7968882	0.91637415	0.80743045	1.091598	1.046334	0.97021	0.871639	1.13	1.20	AT5G66340	hypothetical protein
254827_at	0.0427	1.0256059	1.3113284	1.2155427	1.1315509	1.1367273	1.1127318	0.9391617	1.7324075	0.8461789	0.9893296	0.99060696	1.093931	0.67188746	0.884122	0.9471234	0.90040964	1.171007	1.230257	0.958877	0.850886	1.22	1.45	AT4G12650	endomembrane protein 70, putative TM4 family;
254125_at	0.0427	0.9256033	0.9904581	0.72690024	0.8154106	0.9991453	1.0651878	0.9054648	0.92321014	1.0234942	0.980423	1.0631791	1.1527542	1.2230967	1.0008548	1.0262784	1.1351738	0.864608	0.973252	1.054963	1.096351	0.82	0.89	AT4G24670	alliinase family protein contains Pfam profiles: PF04864 alliinase C-terminal domain, PF04863 alliinase EGF-like domain
AFFX-PheX-M_at	0.0427	1.0004259	1.0328115	1.002951	0.9995742	0.37014318	0.4281159	0.40642217	0.38976613	0.71028954	1.0404702	1.1190622	1.0790119	1.1983981	1.2348243	0.513215	0.838079	1.008941	0.398612	0.987208	0.946129	1.02	0.42		
251129_at	0.0427	1.0755386	1.1137425	1.0035155	0.9964846	1.1079656	1.0671414	1.1423099	0.87848425	0.92246854	0.9004506	1.0188434	0.83839405	1.0063196	0.85492766	0.7988174	0.8763547	1.04732	1.048975	0.920039	0.884105	1.14	1.19	AT5G01150	hypothetical protein contains Pfam profile PF05056: Protein of unknown function (DUF674)
258538_at	0.0427	1.0442656	0.9600264	1.0022247	0.9459572	0.86031085	0.9977752	0.82968087	0.7149893	1.161354	1.0296667	1.0982918	0.96866536	1.1423144	1.07939	0.97172415	1.0328257	0.988118	0.850679	1.064494	1.056701	0.93	0.81	AT3G06950	tRNA pseudouridine synthase family protein similar to SP|P07649 tRNA pseudouridine synthase A (EC 4.2.1.70) (Uracil hydrolyase) (Escherichia coli); contains Pfam profile PF01416: tRNA pseudouridine synthase
260808_at	0.0427	1.1067034	0.99274355	1.0702997	1.1895401	1.0870162	1.176036	1.0071679	0.96114296	0.8776743	0.9209413	0.8982504	0.9928321	0.9209413	1.0214806	0.83332235	0.9064134	1.089822	1.057841	0.922425	0.950299	1.18	1.11		
261803_at	0.0427	1.0012261	1.1418461	1.0641221	0.99877405	0.6541418	0.65198267	0.7714817	0.36855423	1.1417458	0.9646756	1.0687487	1.1718717	1.2036082	0.9824009	1.0256157	0.94335407	1.051492	0.61154	1.08676	1.038745	0.97	0.59	AT1G30500	CCAAT-binding transcription factor (CBF-B/NF-YA) family protein contains Pfam profile: PF02045 CCAAT-binding transcription factor (CBF-B/NF-YA)
251655_at	0.0427	1.0955412	1.408221	1.2786634	1.0425894	1.2060434	1.0960355	1.0152787	0.8801244	0.9624136	1.0181977	0.9498468	0.81366754	0.89380556	0.9847213	0.9236525	0.9283579	1.208495	1.049371	0.936031	0.932134	1.29	1.13	AT3G57160	expressed protein
265112_at	0.0427	1.1050388	1.2786634	1.0672432	1.1548488	1.008235	0.9510773	0.9893741	1.0046724	1.0303762	0.9953274	1.0592362	0.8932619	0.9738612	0.8384333	0.95841354	0.9209511	1.151449	0.98834	0.99455	0.922915	1.16	1.07	AT1G62530	hypothetical protein
246592_at	0.0427	1.1055409	1.133842	1.6190176	1.661497	1.1705078	1.086102	0.9844431	1.0155568	0.8657098	0.94082874	0.9591041	1.2109435	0.96181476	0.9386612	0.8733721	0.9079766	1.379974	1.064152	0.994147	0.920456	1.39	1.16	AT5G14890	NHL repeat-containing protein contains Pfam profile PF01436: NHL repeat
264215_at	0.0427	1.180663	0.99459344	1.2289561	1.1435958	1.1984832	1.0106571	1.0764182	0.94643056	0.9107139	1.1243762	0.93134266	0.8561521	1.0054065	0.8959565	0.88894045	0.87947977	1.136952	1.057997	0.955646	0.917446	1.19	1.15	AT1G65340	cytochrome P450, putative similar to cytochrome P450 GI:4688670 from [Catharanthus roseus]
255317_at	0.0427	1.0319417	1.1889317	1.0508513	1.0064181	0.9935818	1.1187222	0.9691636	1.3561021	1.011984	0.9150914	1.027674	0.91655326	0.87774277	0.95808333	0.8407303	0.87277335	1.069536	1.109392	0.967826	0.887332	1.11	1.25	AT4G04180	AAA-type ATPase family protein contains Pfam domain, PF00004: ATPase, AAA family
[illegible]	[illegible]	[illegible]	[illegible]	[illegible]	[illegible]	[illegible]	[illegible]	[illegible]	[illegible]	[illegible]	[illegible]	[illegible]	[illegible]	[illegible]	[illegible]	[illegible]	[illegible]	[illegible]	[illegible]	[illegible]	[illegible]	[illegible]	[illegible]	[illegible]	[illegible]

256627_at	0.037	1.4290679	0.8738824	1.0121182	2.1194363	1.8436332	1.0300331	1.0662017	1.3725867	0.4659857	0.6497686	0.98788184	0.98251253	0.5855412	0.632697	0.7202325	1.046982	1.358626	1.328114	0.771537	0.746363	1.76	1.78	AT3G19970	expressed protein
262544_at	0.037	1.1928214	1.3778806	1.4315512	1.3620744	1.1677926	0.90478927	1.0017377	0.7519794	0.7560711	0.81406665	1.0060606	1.0059313	0.811969	0.884063	0.9982623	0.9271071	1.341082	0.956575	0.895532	0.90535	1.50	1.06	AT1G15420	expressed protein
248018_at	0.0369	1.1764405	1.1378065	1.0500118	1.246719	1.3857254	1.1645856	1.0293896	0.90700984	0.9706104	0.9094194	0.9488655	0.9228187	0.94854414	1.0488778	0.8023495	0.89784914	1.152744	1.121678	0.937929	0.924405	1.23	1.21	AT5G56470	FAD-dependent oxidoreductase family protein contains TIGRfam [illegible]
247394_at	0.0368	1.3034458	1.1212529	1.4948418	1.7320174	1.2092582	1.0679286	1.3993827	0.6892062	0.7116395	0.8582256	0.86705863	1.085868	0.73109164	0.73467207	0.93207145	0.9086155	1.412889	1.091444	0.880693	0.826613	1.60	1.32	AT5G62860	F-box family protein-related contains TIGRFAM TIGR01640: F-box [illegible]
253947_at	0.0368	1.0034513	1.2067201	0.89962304	0.9811912	0.9208775	0.7082696	0.65847695	0.973477	1.1273406	1.2924197	1.2443768	1.6448038	0.9965487	1.3865387	1.0585951	0.96381587	1.022746	0.815415	1.327235	1.101375	0.77	0.74	AT4G26760	microtubule associated protein (MAP65/ASE1) family protein low [illegible]
250418_at	0.0368	0.93417275	1.1225028	1.1270831	0.9882246	1.1064276	1.0855368	1.118898	1.4916538	0.9065977	0.9062458	0.9086019	1.0286633	0.83198005	0.93518174	1.0117754	0.93768847	1.042996	1.200629	0.937527	0.929156	1.11	1.29	AT5G11240	transducin family protein / WD-40 repeat family protein contains [illegible]
256450_at	0.0368	1.110317	1.1350003	0.9743083	1.1801802	1.1374837	1.0912516	1.0256917	0.94622725	1.0348971	0.9455179	1.0754753	0.8776899	0.9301975	0.92997235	0.87829155	0.87575305	1.099951	1.050164	0.883395	0.903554	1.12	1.16	AT1G75290	isoflavone reductase, putative similar to SP[P52577 isoflavone red[illegible]
267099_at	0.0367	0.6481739	0.932556	0.82478166	0.9363308	1.3069432	1.2520864	1.3931501	1.8759656	0.6856555	0.64930326	0.72802347	0.6116153	1.127195	1.0636693	1.0649996	1.2209808	0.835461	1.457036	0.668649	1.119211	1.25	1.30	AT2G41460	apurinic endonuclease-redox protein / DNA-(apurinic or apyrimidi[illegible]
247819_at	0.0367	0.9882928	0.9631102	1.1371107	0.85052836	1.7939422	1.1892492	1.690142	2.2630014	1.0117072	0.88373595	0.74225026	0.7153397	1.1647277	0.9027935	0.92629343	1.251302	0.984761	1.734084	0.838258	1.061279	1.17	1.63	AT5G58350	protein kinase family protein contains protein kinase domain, Pfam[illegible]
262077_at	0.0367	1.0388149	1.0354272	1.116246	1.0910097	1.0407424	0.9833896	1.0061034	1.6108694	1.1038592	0.7790505	0.94500417	0.9938966	0.7846487	0.6665301	0.8415387	0.7894717	1.070374	1.160276	0.955453	0.770547	1.12	1.51	AT1G59610	dynamin-like protein, putative (ADL3) strong similarity to dynamin[illegible]
263348_at	0.0367	1.0013874	1.1285183	1.499539	1.1585114	1.2544378	1.1592212	1.1548748	2.3370366	0.9647699	0.9769186	0.9450442	0.83108884	0.7586387	0.8710893	0.9527483	0.99861264	1.196989	1.476393	0.929455	0.895272	1.29	1.65	AT2G05710	aconitate hydratase, cytoplasmic, putative / citrate hydro-lyase/ac[illegible]
247114_at	0.0367	0.988809	1.1069642	1.1830577	1.3560337	0.8942925	1.1011909	0.9743716	1.0540732	1.0710974	1.0134759	0.97775596	1.0348642	0.88409746	0.81109655	0.8547338	0.7821567	1.158716	0.983482	1.024298	0.833021	1.13	1.18	AT5G65910	BSD domain-containing protein contains Pfam profile PF03909: BS[illegible]
263883_at	0.0366	1.0536107	1.0503105	1.0791981	0.8172579	0.5187554	0.7562126	0.59707835	0.9864468	1.4382079	1.7226839	1.1667869	1.3112869	0.8522533	0.91331565	1.0135534	0.9034573	1.000094	0.714848	1.409741	0.920645	0.71	0.78	AT2G21830	DC1 domain-containing protein contains Pfam profile PF03107: DC[illegible]
248636_at	0.0366	0.9985672	1.1637866	1.2601167	1.1370251	1.1764747	1.0968155	1.0014329	0.92676306	1.0151998	0.84445477	0.9756919	0.8948149	0.94102293	0.8602733	0.9325701	1.0221235	1.139874	1.050372	0.93254	0.938997	1.22	1.12	AT5G49080	proline-rich extensin-like family protein contains proline-rich exten[illegible]
246333_at	0.0366	1.2308102	1.0483377	1.01024	1.1109023	1.0480251	0.96287036	1.1384686	1.044524	0.9552922	0.91813636	0.98942506	0.8926884	1.0270942	0.86642116	0.969592	0.9897601	1.100073	1.048472	0.938886	0.963159	1.17	1.09	AT3G44840	S-adenosyl-L-methionine:carboxyl methyltransferase family protei[illegible]
259822_at	0.0366	1.0128846	1.0400207	0.79054785	0.82763287	0.93109465	0.9711526	0.878352	0.6254267	0.94268304	0.9871153	1.0154516	1.0636051	1.2042264	1.4300078	1.2522423	1.071265	0.920267	0.851506	1.002214	1.239435	0.92	0.69	AT1G66230	myb family transcription factor (MYB20) similar to myb-related tra[illegible]
250163_at	0.0365	1.0802199	0.9969481	1.1202068	1.0035051	1.1759034	0.95088285	1.0588671	1.310931	0.9513443	1.0093473	0.9010649	0.87348133	1.003052	0.8254379	0.96357983	0.8465362	1.05022	1.124146	0.933809	0.909651	1.12	1.24	AT5G15270	KH domain-containing protein various predicted proteins, Arabido[illegible]
254155_at	0.0363	1.1740963	1.079297	1.1881518	1.2002462	0.97273034	1.1063745	0.9919775	1.4557	0.95373416	0.9887834	1.0470246	0.86406004	0.86101425	1.0080225	0.9460207	0.8601816	1.160448	1.131696	0.963401	0.91881	1.20	1.23	AT4G24480	serine/threonine protein kinase, putative similar to serine/threonin[illegible]
250068_at	0.0363	1.0505941	1.1326501	1.2341627	1.1653056	1.0265602	1.1539358	0.99429923	0.9162913	0.8022961	1.1524293	1.0057006	0.86236185	0.7979472	0.9142174	0.8944355	0.8268584	1.145678	1.022772	0.955059	0.858365	1.20	1.19	AT5G17960	DC1 domain-containing protein contains Pfam profile PF03107: DC[illegible]
267393_at	0.0363	1.0439047	1.0148795	1.315758	1.5290252	1.4434836	1.3875412	0.98512053	2.582718	0.2485158	0.60942817	0.7422107	0.85044146	0.58255947	0.97828114	0.9490444	1.0723301	1.225892	1.599716	0.611649	0.895554	2.00	1.79	AT2G44500	expressed protein contains Pfam PF03138: Plant protein family. Th[illegible]
250054_at	0.0363	0.9684544	0.9089731	1.0467501	1.0537446	1.4029047	1.2101539	1.8589867	1.9986644	0.8758557	1.0001335	0.890338	0.7975195	0.93626606	0.8606632	0.9998666	1.318283	0.994481	1.617677	0.890962	1.02877	1.12	1.57	AT5G17860	cation exchanger, putative (CAX7) contains similarity to SWISS-PRO[illegible]
249616_s_at	0.0363	0.93785367	1.1395146	0.9171808	1.0523003	0.82688063	0.6986314	0.79642594	0.7877594	1.0615726	1.0461749	1.016424	1.3342576	1.0111108	0.9888892	0.88885146	1.1079398	1.011712	0.777424	1.114607	0.999198	0.91	0.78	AT5G37750;AT5G[illegible]	[AT5G37750, DNAJ heat shock N-terminal domain-containing prot[illegible]
262746_at	0.0362	1.2625461	1.2088788	1.4214224	1.2031894	1.3199877	1.0417178	1.0886387	0.7886134	1.0201232	0.96058583	0.9577858	0.9798767	0.9261915	0.8257771	0.9699949	0.8580397	1.274009	1.059739	0.979593	0.895293	1.30	1.18	AT1G28960	MutT/nudix family protein similar to coenzyme A diphosphatase [[illegible]
250461_at	0.0362	1.0946753	1.1866432	1.4258267	1.11691	0.9936219	1.1296628	0.8964754	1.3021507	0.81789017	0.9233108	1.0063782	0.9537136	0.6814185	0.8406359	1.0336232	0.9040977	1.206014	1.080478	0.925323	0.864944	1.30	1.25	AT5G10010	expressed protein
247631_at	0.0362	0.92649543	0.9755529	1.0574292	1.1255437	1.0658867	1.0631405	1.1088071	1.0574548	0.96214026	1.0244471	0.8763838	1.051376	0.86809915	0.9417998	0.90946305	0.8892541	1.021255	1.073822	0.978587	0.902154	1.04	1.19	AT5G60440	MADS-box protein (AGL62) contains Pfal profile PF00319: SRF-type[illegible]
256544_at	0.0362	1.0832819	1.0183234	1.0581079	0.8593488	1.2488376	0.9573082	1.2834034	1.0561223	0.8876893	0.7450777	0.8612215	0.7858409	0.99958897	1.000411	0.91723645	1.0076807	1.004766	1.136418	0.819957	0.981229	1.23	1.16	AT1G42560	seven transmembrane MLO family protein / MLO-like protein 9 (M[illegible]
254738_at	0.0362	1.2691925	1.3951946	1.249271	1.1632866	2.391694	1.2256249	1.3603911	0.9860763	0.9160255	1.0113525	0.9237984	0.9486792	0.98702276	0.8972382	0.94977295	0.98864746	1.269236	1.490947	0.949964	0.95567	1.34	1.56	AT4G13860	glycine-rich RNA-binding protein, putative similar to Glycine-rich R[illegible]
257392_at	0.0362	1.0873605	1.1898867	1.1115423	1.4698175	1.6414366	1.1032917	1.2224165	0.8860944	0.8853874	0.89621866	0.95834285	0.8761973	0.9901392	1.0098608	0.8524817	0.87677693	1.214652	1.21331	0.904037	0.932315	1.34	1.30	AT2G24450	fasciclin-like arabinogalactan family protein similar to fasciclin-like[illegible]
262641_at	0.0361	1.2177681	1.1732277	1.076779	1.1338046	0.9066372	1.0645212	1.0567216	0.9327756	0.87448514	1.017326	1.0807635	0.90159214	0.75116676	0.9826739	0.83680004	0.8903367	1.150395	0.990164	0.968542	0.865244	1.19	1.14	AT1G62730	expressed protein
254197_at	0.0361	1.0002904	1.1531723	1.5364047	1.3590525	1.0021638	0.99115366	1.4324129	1.6617053	1.0226687	0.9726124	0.91575885	0.9789531	0.92134404	0.6994409	0.82411534	0.99970955	1.26223	1.271859	0.972498	0.861152	1.30	1.48	AT4G24040	glycosyl hydrolase family protein 37 / trehalase, putative similar to [illegible]
253339_at	0.0361	0.9991618	0.9864406	0.8668574	1.0168798	1.2885975	1.2282357	1.156347	1.1011939	0.9017927	0.8361842	0.9179471	0.91842103	1.0008382	0.9232912	1.059644	1.10558	0.967335	1.193594	0.893586	1.022338	1.08	1.17	AT4G33520	metal-transporting P-type ATPase, putative (PAA1) nearly identical[illegible]
260673_at	0.036	1.004177	1.1117293	0.80089813	0.97829795	0.93378013	0.995823	0.83690524	0.89102274	0.8959731	1.3438295	1.4003999	1.3794209	0.97080135	1.4201636	1.2614572	1.6123662	0.973776	0.914383	1.254906	1.316197	0.78	0.69	AT1G19330	expressed protein
253031_at	0.036	1.1098517	1.1508878	1.2845737	1.4111601	1.4005709	1.0039593	1.0439476	0.9863941	0.914137	1.1509713	0.92679816	0.87227124	0.97933364	0.9960407	0.80008596	0.85319793	1.239118	1.108718	0.966044	0.907165	1.28	1.22	AT4G38190	cellulose synthase family protein similar to cellulose synthase catal[illegible]
249730_at	0.036	1.0044675	0.94869155	1.226918	1.3294079	1.3087947	0.99553275	1.2347575	1.4980885	0.81642914	0.77452916	0.9665051	1.0972745	0.70060784	0.8461044	0.9617391	1.0120434	1.127371	1.259293	0.913684	0.880124	1.23	1.43	AT5G24430	calcium-dependent protein kinase, putative / CDPK, putative simil[illegible]
246547_at	0.036	1.0365909	1.1629667	1.0584828	0.9989763	0.79856384	0.744597	0.7315753	0.6284867	1.2518014	1.0382639	1.1808343	0.95750815	1.0195477	1.0010238	0.8774758	0.9472642	1.064254	0.725806	1.107102	0.961328	0.96	0.76	AT5G14970	expressed protein
254899_at	0.036	1.1438596	0.9246197	1.1507038	1.0464017	1.0716246	1.2505131	1.2193673	0.99784327	1.0630707	0.9178236	1.0021567	0.8905726	0.902966	0.95230573	0.9473045	0.8807608	1.066396	1.134837	0.968406	0.920834	1.10	1.23	AT4G11490	protein kinase family protein contains Pfam PF00069: Protein kina[illegible]
254110_at	0.036	1.078509	0.9435088	0.8321037	0.88265234	0.8870907	0.8429626	0.77501947	0.3516962	1.2206872	1.459947	1.3339875	1.4573593	1.0564913	1.2038733	1.262007	0.8380021	0.934193	0.714192	1.367995	1.090242	0.68	0.66	AT4G25260	invertase/pectin methylesterase inhibitor family protein low simila[illegible]
259884_at	0.036	1.0547588	1.1451455	1.2629818	1.1111013	1.119157	0.84653515	1.1541283	1.7298117	0.93544525	0.8553581	0.94524115	1.1142415	0.7103981	0.73926556	0.8005612	0.87592053	1.143497	1.212408	0.962572	0.781536	1.19	1.55	AT1G76390	armadillo/beta-catenin repeat family protein / U-box domain-cont[illegible]
262217_at	0.0359	1.0908169	1.0179266	1.217907	1.0901829	1.09404	1.0697371	1.0652273	0.9416188	1.1181014	0.9540995	0.9629252	0.9111089	0.9820735	0.90038776	0.87758744	0.8676739	1.104208	1.042656	0.986559	0.906931	1.12	1.15	AT1G74770	expressed protein
254287_at	0.0359	1.0459341	1.0119125	1.1736095	1.1592017	1.0870504	1.1043128	1.0313947	0.96322143	0.9880875	1.122809	0.89602137	0.9337866	0.9002361	0.9582448	0.90460914	0.81072783	1.097664	1.046495	0.985176	0.893704	1.11	1.17	AT4G22960	hypothetical protein contains Pfam domain PF04424: Protein of un[illegible]
256871_at	0.0359	1.1295391	1.1680216	1.2479503	1.0018306	1.058209	1.2318347	1.0540023	0.92290354	0.9645128	0.8763232	0.9981695	0.85740995	0.9685963	0.97992355	0.92220455	1.0081187	1.136835	1.066737	0.924104	0.969711	1.23	1.10	AT3G26480	transducin family protein / WD-40 repeat family protein contains P[illegible]
262504_at	0.0359	0.93163073	1.5273386	1.0247902	1.1729591	1.1170763	1.2340032	1.0000079	1.4788404	0.7901234	1.0412348	0.8484563	0.99999213	0.6145209	0.9359514	0.87104684	0.8480328	1.16418	1.207482	0.919947	0.817388	1.27	1.48	AT1G21750	protein disulfide isomerase, putative similar to SP[P29828 Protein [illegible]
245423_at	0.0359	1.2294259	1.17616	1.1669502	1.3672303	0.9769668	1.0069907	1.0379874	0.89694136	1.0538764	0.80508816	0.9930093	1.0649638	0.9211355	0.9286678	0.81405056	0.97396207	1.234942	0.979722	0.979234	0.909454	1.26	1.08	AT4G17483	palmitoyl protein thioesterase family protein
253993_at	0.0359	0.9721782	0.974681	1.0368545	1.0450808	1.1379275	1.1319609	1.1262796	1.3152779	0.50087565	0.6749338	0.85756016	0.7984588	0.61149395	0.97692275	1.0230772	1.0885314	1.007199	1.177861	0.707957	0.925006	1.42	1.27	AT4G26070	mitogen-activated protein kinase kinase (MAPKK) (MKK1) (MEK1) [illegible]
246177_at	0.0359	1.1138136	1.0578866	1.295997	1.0323124	1.0308317	0.99005187	1.1695504	1.002705	0.98454356	0.99729496	0.9479755	0.9046627	0.88223064	1.0499238	0.91200835	0.88516647	1.125002	1.048285	0.958619	0.932332	1.17	1.12	AT5G28420	hypothetical protein predicted proteins - Arabidopsis thaliana
[illegible]																									

252223_at	0.0301	1.0144241	0.7108749	0.77786595	0.49349692	0.6295726	1.0000484	0.8539958	0.87201136	1.2458317	1.0893866	0.99451625	1.0536389	1.1379968	1.0417944	1.1912789	0.9999515	0.749165	0.838907	1.095843	1.092755	0.68	0.77	AT3G49850	myb family transcription factor contains Pfam profile: PF00249 myb-like DNA-binding domain
250507_at	0.0301	1.0060158	1.1870954	1.3693895	1.155525	1.1194824	0.9939844	1.0815247	0.9775082	0.9111763	0.97562623	1.030231	0.94324607	0.95028454	1.0223856	0.87936586	0.8194161	1.179506	1.043125	0.96507	0.917863	1.22	1.14	AT5G09940	hypothetical protein
253886_at	0.03	0.8734684	0.99502105	0.8982662	0.7536399	0.9652685	0.6990461	0.7890597	1.0816054	1.9093536	1.2274232	1.2333364	1.0049789	1.5897881	1.2892834	0.94666916	1.2073605	0.880099	0.883745	1.343773	1.258275	0.65	0.70	AT4G27710	cytochrome P450 family protein contains Pfam profile: PF00067 cytochrome P450
264186_at	0.03	1.1076357	1.3578402	1.5434865	1.318003	0.98413265	1.0148673	0.9430069	1.419001	1.2583324	0.98513275	1.1540035	0.95860046	0.8404449	0.78501344	0.7865526	0.82152355	1.331741	1.090252	1.089017	0.808384	1.22	1.35	AT1G54570	esterase/lipase/thioesterase family protein contains Interpro entry IPR000379
254780_s_at	0.03	1.0156388	1.4142721	1.3440183	1.4332962	1.176952	0.89420784	0.94918746	1.2629791	0.8630115	1.0317498	0.8969664	0.9918607	0.85594267	0.8677701	1.0081393	0.9076612	1.301806	1.070832	0.945897	0.909878	1.38	1.18	AT4G12770	auxilin-related low similarity to SP|Q27974 Auxilin (Bos taurus)
265966_at	0.03	0.7851243	0.81987214	0.8016412	0.77144307	1.2285304	1.0234915	1.0948088	1.0348362	0.9765086	0.92092556	0.92489916	0.88383716	1.2085078	1.0631081	1.1449242	1.1796254	0.79452	1.095417	0.926543	1.149041	0.86	0.95	AT2G37220	29 kDa ribonucleoprotein, chloroplast, putative / RNA-binding protein cp29, putative similar to SP|Q43349 29 kDa ribonucleoprotein, chloroplast prec [illegible]
249677_at	0.03	0.8202943	0.5022217	0.7554314	0.58044827	1.0109979	0.90227664	0.62327147	1.4054306	2.1254706	1.5093492	0.9890019	0.95446795	1.5606084	1.3547252	1.1197145	1.2891226	0.664599	0.985494	1.394572	1.331043	0.48	0.74	AT5G35970	DNA-binding protein, putative similar to SWISS-PROT:Q60560 DNA-binding protein SMUBP-2 (Immunoglobulin MU binding protein 2, SMUBP-2) [Meso [illegible]
253406_at	0.03	1.1214674	0.82125884	0.71323115	0.6767039	0.7524215	0.97890186	0.7822272	0.7263886	1.2420936	1.07256	1.0516132	1.021098	1.3534032	1.1053072	1.0213535	0.9043811	0.833165	0.809985	1.096841	1.096111	0.76	0.74	AT4G32890	zinc finger (GATA type) family protein GATA transcription factor 3, Arabidopsis thaliana, gb:Y13650
266661_at	0.0299	1.1236005	1.4301969	1.0749979	1.0257164	1.2494019	0.99945986	1.3726764	0.9953984	0.81059295	0.8670054	0.9603852	0.8322762	0.94394076	1.0005401	0.82409596	1.0697751	1.163628	1.154234	0.867565	0.959588	1.34	1.20	AT2G25820	transcription factor, putative similar to TINY [Arabidopsis thaliana] GI:1246403
262332_at	0.0298	0.98380804	1.0151786	1.1698087	1.030295	1.0883454	1.154013	1.2218486	1.0140007	0.9649213	0.9103508	0.9859993	0.8443235	0.8809462	1.0818526	0.93245184	0.9024434	1.049773	1.119552	0.926399	0.949424	1.13	1.18	AT1G64030	serpin family protein / serine protease inhibitor family protein similar to phloem serpin-1 [Cucurbita maxima] GI:9937311, serpin [Triticum aestivum] G [illegible]
245430_at	0.0298	1.0097002	0.9543416	1.0704316	1.0254685	0.99029994	1.0851362	1.2445972	1.2240847	0.8388124	1.0126996	0.96412563	1.0160534	0.82156914	0.8724336	0.9494291	0.93717664	1.014985	1.13603	0.957923	0.895152	1.06	1.27	AT4G17060	expressed protein
225587_at	0.0298	1.0885609	1.0748062	1.4044603	1.3982677	0.86071444	1.0467612	1.1829027	1.2016588	0.9833681	0.9957504	0.82892007	0.93395364	0.73699313	1.0042496	0.8782901	0.88052434	1.241524	1.073009	0.935498	0.875014	1.33	1.23	AT3G45620	transducin family protein / WD-40 repeat family protein contains 7 WD-40 repeats; similar to PC326 protein (GI:200241) [PIR2:S37694] [Mus muscul [illegible]
250315_at	0.0298	1.0737772	1.1554055	0.9987421	0.9381052	0.9077308	0.73164105	0.85970205	0.76040894	1.4007512	1.2043074	1.1204231	1.0780263	1.150395	0.942001	0.97030073	1.0012579	1.041508	0.814871	1.200877	1.015989	0.87	0.80	AT5G12130	integral membrane TerC family protein contains Pfam profile PF03741: Integral membrane protein TerC family
263890_at	0.0298	0.97629905	0.81574047	0.88270813	0.9069645	1.023701	0.9755435	0.5111754	0.49106812	0.8405868	1.4136319	1.6838261	1.989493	1.0291971	1.7395235	2.8234043	1.309496	0.895428	0.750372	1.481884	1.725405	0.60	0.43	AT2G37030	auxin-responsive family protein similar to auxin-induced protein TGSAUR22 (GI:10185820) [Tulipa gesneriana]
262116_at	0.0298	1.4704859	1.2760485	1.533203	1.7983724	1.0975448	1.1001854	1.1752344	0.5741815	0.9456948	0.9753237	1.0246763	0.84131926	0.8023876	0.59822214	0.861008	0.5923761	1.519527	0.986787	0.946754	0.713498	1.60	1.38	AT1G02816	expressed protein contains Pfam profile PF04398: Protein of unknown function, DUF538
257253_at	0.0298	0.8558613	0.8460422	0.83203197	0.71668583	0.8266552	0.922895	0.8631097	1.249508	1.3916403	1.2079699	1.0385953	1.0388973	1.3232672	1.1677464	1.0420768	0.96140474	0.812655	0.965542	1.169276	1.123624	0.70	0.86	AT3G24190	ABC1 family protein contains Pfam domain, PF03109: ABC1 family
249128_at	0.0298	0.81937677	0.98453194	1.015468	1.0764434	0.81544816	0.6658159	0.83115387	0.77406645	1.1377004	1.0493964	1.2188802	1.0911598	1.2954911	1.0282717	0.88714236	0.92751354	0.973955	0.771621	1.124284	1.034605	0.87	0.75	AT5G43440	2-oxoglutarate-dependent dioxygenase, putative similar to 2A6 (GI:599622) and tomato ethylene synthesis regulatory protein E8 (SP|P10967)
248358_at	0.0298	1.3247532	0.9532384	1.0547408	1.3587651	1.1689066	1.0971773	1.0303307	1.1711047	0.794139	0.97293943	1.007281	0.950039	0.9326279	0.95464665	0.84204787	0.9927191	1.172874	1.11688	0.9311	0.93051	1.26	1.20	AT5G52400	cytochrome P450 family protein similar to Cytochrome P450 72A1 (SP:Q05047) (cytochrome P450 loxii hydroxylase) [ge10h] [Catharanthus roseus]
256788_at	0.0297	1.0645804	0.90647525	1.1993388	0.9059126	1.7001176	1.0822718	1.300008	1.6517022	0.7915414	0.58935565	0.7840327	0.858368	1.1578712	0.6537273	0.93641654	1.0635855	1.019077	1.433525	0.755824	0.9529	1.35	1.50	AT3G13730	cytochrome P450, putative similar to Cytochrome P450 90C1 (ROTUNDIFOLIA3) (SP:Q9M066) [Arabidopsis thaliana]; identical to CYP90D (GI:1497101 [illegible]
249035_at	0.0297	0.752247	0.8664629	0.75093704	0.6907566	0.8404459	0.8979656	0.998972	1.0856286	2.3406122	1.7292508	0.9198475	1.1185327	2.2517169	1.3065144	1.001028	1.3389149	0.765101	0.955753	1.527061	1.474544	0.50	0.65	AT5G44190	myb family transcription factor (GLK2) contains Pfam profile: PF00249 myb-like DNA-binding domain
264045_at	0.0297	1.0348533	1.1657821	1.1819015	1.1732794	0.9981214	0.87519395	0.99441	1.2302766	1.0019281	0.9764851	1.0349026	1.0018787	0.7771946	0.87467134	0.89764225	0.84747124	1.138954	1.0245	1.003799	0.849245	1.13	1.21	AT2G22450	riboflavin biosynthesis protein, putative similar to SP|P50855 Riboflavin biosynthesis protein ribA [includes: GTP cyclohydrolase II (EC 3.5.4.25); 3,4-dih [illegible]
257758_at	0.0296	1.1196802	0.9910615	1.2589542	1.0896813	1.0687107	1.0416402	1.2480779	1.9050865	1.0089386	0.79666543	0.8609058	0.89643955	0.8215847	0.842176	0.98403937	0.91058695	1.114844	1.315879	0.890737	0.889597	1.25	1.48	AT3G22980	elongation factor Tu family protein similar to eukaryotic translation elongation factor 2 GB:NP_001952 [Homo sapiens]
246595_at	0.0296	1.0626798	1.167633	1.4739536	1.3396391	0.9927497	0.97322166	1.006176	1.3314669	0.9938239	1.0327414	0.9576229	1.0236387	0.792414	0.8347657	0.96573985	0.92799044	1.260976	1.075904	1.001957	0.880227	1.26	1.22	AT5G14780	formate dehydrogenase (FDH) identical to GI:7677266
248307_at	0.0296	1.1924361	1.0460051	1.0673132	1.1919352	0.909103	0.98883307	1.1039345	1.560027	0.9366649	0.87460446	1.011167	1.0331055	0.7683124	0.7967659	0.7357011	0.8857322	1.124422	1.140474	0.963885	0.796628	1.17	1.43	AT5G52850	pentatricopeptide (PPR) repeat-containing protein contains INTERPRO:IPR002885 PPR repeats
247172_at	0.0296	1.4007326	1.3171633	1.3569317	1.4480461	1.5099789	1.1083139	0.9693413	0.93043077	0.8282813	1.2312728	0.9393787	0.843826	0.98564076	1.0143591	0.8886788	0.76411676	1.380718	1.129516	0.96069	0.913199	1.44	1.24	AT5G65550	UDP-glucoronosyl/UDP-glucosyl transferase family protein contains Pfam profile: PF00201 UDP-glucoronosyl and UDP-glucosyl transferase ;similar to f [illegible]
246888_at	0.0295	0.8773676	0.9986572	1.086285	0.8612737	1.2788523	1.484556	1.2705674	1.0776224	0.7237931	0.53491265	0.8881428	0.66145325	0.9658478	1.1249174	1.1080916	1.0013427	0.955896	1.2779	0.702075	1.05005	1.36	1.22	AT5G26270	expressed protein ; expression supported by MPSS
267548_at	0.0295	0.99828495	1.4015812	1.406483	1.3382448	1.0165231	0.9013748	1.0344322	1.121974	0.0826273	0.86933947	1.0117149	0.8679526	0.8183195	0.8839206	0.7495273	1.0628406	1.283648	1.018576	0.932909	0.87653	1.38	1.16	AT2G32660	disease resistance family protein / LRR family protein contains leucine rich-repeat domains Pfam:PF00560, INTERPRO:IPR001611; similar to Cf-4 [Lycop [illegible]
263724_at	0.0294	0.9770467	1.0308741	1.1326143	1.1023062	1.2395595	1.1100487	1.0398651	0.98059546	1.0245523	0.84803336	0.90297216	0.7755625	1.0198045	0.8945087	0.81631255	0.8476165	1.06071	1.092417	0.84278	0.894561	1.26	1.22	AT2G13510	hypothetical protein low similarity to zinc finger protein [Arabidopsis thaliana] GI:976277
263754_at	0.0294	1.0687883	0.9142057	1.2313544	1.3688018	1.0722404	1.2197139	1.1218921	1.0064508	0.9418687	0.8019039	1.0095304	0.85523915	0.85408187	0.8064682	0.8935493	0.9551144	1.145788	1.105074	0.902136	0.902303	1.27	1.22	AT2G21510	DNAJ heat shock N-terminal domain-containing protein similar to SP|P39101 CAJ1 protein, Saccharomyces cerevisiae; contains Pfam profile PF00226 [illegible]
265882_at	0.0294	0.6784215	0.75234437	0.8804093	0.85870296	1.5075636	1.2747889	1.6542547	2.4908652	0.6693583	0.65130943	0.58017284	0.5938037	1.1195908	1.2468926	1.1815946	1.1921699	0.79247	1.731868	0.623661	1.185062	1.27	1.46	AT2G42490	copper amine oxidase, putative similar to copper methylamine oxidase precursor (MAOXII) [Arthrobacter sp.] SWISS-PROT:Q07123
261033_at	0.0294	1.2814326	0.9654348	1.0345652	2.8245485	1.5991272	1.0968288	1.6099432	1.1269054	0.1416584	0.60238075	0.6386653	0.83249986	0.50952643	0.5380527	0.30572522	1.061555	1.526495	1.358201	0.553801	0.603715	2.76	2.25	AT1G17380	expressed protein
256134_at	0.0294	0.97290367	1.0166508	0.98499537	0.8563891	0.94854903	0.87504035	0.8713788	1.0075104	1.1965518	1.207663	1.0349047	1.1105397	1.1341953	0.99248946	0.9518666	1.0176761	0.957735	0.92562	1.162415	1.024057	0.82	0.90	AT1G13560	aminoalcoholphosphotransferase (AAPT1) identical to aminoalcoholphosphotransferase GI:3661593 from [Arabidopsis thaliana]
257428_at	0.0294	0.8951982	0.8080314	0.8681002	1.0047975	1.095249	0.9722486	0.9084311	0.9507836	1.4428178	1.1068441	1.1512833	1.2977076	1.1333228	0.9952025	0.88097754	1.1693392	0.894032	0.981678	1.249663	1.044716	0.72	0.94	AT1G78990	transferase family protein low similarity to acetyl CoA: benzylalcohol acetyltransferase Clarkia breweri GI:3170250, GI:6166336, Clarkia concinna GI:61 [illegible]
257320_at	0.0293	1.1241995	1.1051714	1.1502396	1.039891	1.1863345	1.2869489	1.0099442	0.93487513	0.95313525	0.9832509	0.9963555	0.95822066	1.0036443	0.91823727	0.85776496	0.8896538	1.104875	1.104526	0.972741	0.917325	1.14	1.20	AT2G07749;ATM [illegible]	[AT2G07749, hypothetical protein contains Pfam profile PF05919: Mitovirus RNA-dependent RNA polymerase];[ATMG01110, orf251 hypothetical pro [illegible]
[illegible]																									

[illegible]	[illegible]	1.0622721	1.1496826	1.2370629	1.2719382	1.089563	1.1059376	0.83949506	1.4016001	0.8107176	0.7185341	1.0773147	0.81650347	0.66831344	0.74807054	0.9266857	0.9377281	1.180239	1.109149	0.855767	0.820199	1.38	1.35	AT5G40740	expressed protein
[illegible]	[illegible]	1.0070407	0.8812706	0.9950292	1.0076247	1.284139	1.145195	1.4991299	1.5261458	0.59345096	0.81075644	0.9163065	0.9327059	0.90233177	0.9088011	1.0049708	1.0924486	0.972741	1.363652	0.813305	0.977138	1.20	1.40	AT2G13650	GDP-mannose transporter (GONST1) identical to GDP-mannose transporter SP:Q941R4 from [Arabidopsis thaliana]
[illegible]	[illegible]	1.0946817	1.0021164	1.2176578	1.354145	1.3097508	1.1507226	1.5055897	0.83576816	0.86763686	1.0268934	0.9978835	0.8856322	0.81672204	0.7581699	0.8535155	0.83226454	1.16715	1.200458	0.944511	0.815168	1.24	1.47	AT5G28930	hypothetical protein various predicted proteins, Arabidopsis thaliana
[illegible]	[illegible]	1.0194893	1.0645065	1.0637951	1.1089535	1.0540786	1.1559167	1.0519553	0.970957	1.0189303	0.9027661	0.9754692	0.98020023	0.9810697	0.9703229	0.9486233	0.86502707	1.064186	1.058227	0.969341	0.941261	1.10	1.12	AT4G12960	gamma interferon responsive lysosomal thiol reductase family protein / GILT family protein similar to SP|P13284 Gamma-interferon inducible lysosom[illegible]
[illegible]	[illegible]	1.1890138	1.295242	1.2070569	1.1947026	1.0847135	0.91136014	0.9878249	0.874933	1.0733509	1.0521678	1.0121751	0.97108245	0.97691077	0.86582977	0.8608387	0.8928415	1.221504	0.964708	1.027194	0.899105	1.19	1.07	AT1G10670	ABC transporter family protein similar to ABC transporter ATPase GB:AAC68280 [Chlamydia trachomatis]
[illegible]	[illegible]	1.0387604	1.2007695	1.3085145	1.3669237	0.98135775	1.0096589	1.1769682	1.4336053	0.9903411	1.0181609	0.9445739	0.98481387	0.8947541	0.9721586	0.93187165	0.9349351	1.228742	1.150398	0.984472	0.93343	1.25	1.23	AT1G20920	DEAD box RNA helicase, putative similar to RNA helicase [Rattus norvegicus] GI:897915; contains Pfam profiles PF00270: DEAD/DEAH box helicase, PF[illegible]
[illegible]	[illegible]	1.4222885	1.0522039	1.0695946	1.2978384	1.0380726	1.1445503	0.9575797	1.8302323	0.9299736	0.7068695	0.98947793	1.0105221	0.6511332	0.7826993	0.9046664	0.72037935	1.210481	1.242609	0.909211	0.76467	1.33	1.63	AT4G02540	DC1 domain-containing protein contains Pfam profile PF03107: DC1 domain
[illegible]	[illegible]	1.0028223	0.82225376	0.8884804	0.93761766	0.79588085	0.99717766	0.85831344	0.902	1.1435105	1.3402643	1.0171055	1.0902786	1.167793	1.0791882	1.0512483	0.89721966	0.912794	0.888343	1.14779	1.04862	0.80	0.85	AT3G58820	F-box family protein contains F-box domain Pfam:PF00646
[illegible]	[illegible]	0.9713121	1.1680887	1.4024243	1.1657282	1.1391711	0.9783698	1.0216303	1.4906323	1.0820686	0.872342	0.83998215	1.0325291	0.8898604	0.78010225	0.8182492	0.8144516	1.176888	1.157451	0.95673	0.825666	1.23	1.40	AT1G21700	SWIRM domain-containing protein / DNA-binding family protein contains similarity to SWI/SNF complex 170 KDa subunit [Homo sapiens] gi|1549241|[illegible]
[illegible]	[illegible]	0.7837249	0.84289503	0.4813472	0.52693325	1.4749161	1.0595207	0.9483607	0.6588293	1.095186	1.0516392	0.94547385	0.720718	2.1148355	1.662849	1.4435596	1.9188861	0.658725	1.035407	0.953254	1.783283	0.69	0.58	AT1G35560	harpin-induced family protein / HIN1 family protein / harpin-responsive family protein similar to harpin-induced protein hin1 (GI:1619321) [Nicotiana[illegible]
[illegible]	[illegible]	1.1720012	1.1794604	1.3200796	1.1655173	1.0244147	1.04258	0.9755854	0.95486116	1.2033707	0.86661714	0.92486805	0.96542	0.9272578	0.8798471	0.9250921	[illegible]	1.209265	1.050875	0.987429	0.924685	1.22	1.14	AT2G14010	hypothetical protein and genefinder
[illegible]	[illegible]	1.1127578	0.9860471	1.0404884	1.0651395	1.0634059	1.0139529	1.1284423	1.0439762	0.98564225	1.0807117	0.9701407	0.9068422	0.93179154	0.8999075	0.9323694	0.9616206	1.051108	1.062444	0.985834	0.931422	1.07	1.14		
[illegible]	[illegible]	1.0608339	0.95834243	0.9269768	1.0141288	0.9079475	0.9432937	0.86261857	0.8868733	1.331932	1.1786767	1.098525	1.0089902	1.1258034	1.0648953	0.97381294	0.9910098	0.99007	0.900195	1.154531	1.03888	0.86	0.87	AT3G55580	regulator of chromosome condensation (RCC1) family protein UVB-resistance protein UVR8, Arabidopsis thaliana, EMBL:AF130441; contains Pfam PF[illegible]
[illegible]	[illegible]	1.003919	1.1344961	1.0832988	1.016839	1.0421222	1.1049303	1.0440905	0.98513013	0.90791476	1.0365078	0.996081	0.8665579	0.8744614	0.9660548	0.940984	0.93029416	1.059638	1.044068	0.951765	0.927949	1.11	1.13	AT3G32903	hypothetical protein
[illegible]	[illegible]	1.2855172	1.0752484	1.371504	1.3117985	1.0397387	1.2216075	0.9988636	0.8142852	0.75647074	0.88927966	1.0011364	0.9788985	0.5402204	0.81931853	0.87800026	0.9235078	1.261017	1.243624	0.906446	0.790262	1.39	1.57	AT1G78240	dehydration-responsive protein-related similar to early-responsive to dehydration stress ERD3 protein [Arabidopsis thaliana] GI:15320410; contains PF[illegible]
[illegible]	[illegible]	1.1468428	1.0217721	1.1668402	1.0775255	1.0131667	1.2052821	0.97966635	1.1277953	0.98629296	0.97764647	1.0306009	0.8678813	0.98683345	0.960813	0.874505	0.91262096	1.103245	1.081478	0.965605	0.933693	1.14	1.16	AT4G21880	pentatricopeptide (PPR) repeat-containing protein contains Pfam profile PF01535: PPR repeat
[illegible]	[illegible]	1.0421879	1.1276166	1.287919	1.2659492	1.0045799	1.2391856	1.0818124	1.7166245	0.97458076	0.9386854	0.8225603	0.7945042	0.93967104	0.99542	0.9561711	0.99109495	1.180918	1.260551	0.882583	0.970589	1.34	1.30	AT5G05460	glycosyl hydrolase family 85 protein contains similarity to endo-b-N-acetylglucosaminidase GI:13774138 from [Mucor hiemalis]
[illegible]	[illegible]	1.0295513	0.9363584	0.8773061	0.9704488	0.9313811	0.8689003	0.76820356	0.4587561	0.9211349	1.303267	1.3349833	1.3189342	1.1323746	1.2769496	1.2028792	1.146689	0.953416	0.75681	1.21958	1.189723	0.78	0.64	AT2G21830	expressed protein EST gb|T21171 comes from this gene
[illegible]	[illegible]	1.2314498	1.5953494	0.8084451	1.000355	1.4771012	1.7205656	1.0207901	1.4611678	0.71262586	0.76650965	0.99964505	0.71935725	0.7923628	0.7902317	0.652346	1.1233006	1.1589	1.419906	0.799534	0.83956	1.45	1.69	AT5G33370	GDSL-motif lipase/hydrolase family protein similar to family II lipase EXL3 (GI:15054386), EXL1 (GI:15054382), EXL2 (GI:15054384) [Arabidopsis thalian[illegible]
[illegible]	[illegible]	1.191722	0.9617562	1.1343778	1.2753977	1.1084989	1.3341782	1.0654022	2.1401484	0.82637805	0.8268321	0.8245812	0.9223092	0.788011	1.0075667	0.9924332	0.7748178	1.140813	1.412057	0.849975	0.890707	1.34	1.59	AT1G27460	calmodulin-binding protein similar to calmodulin-binding protein MPCBP [Zea mays] GI:10086260; contains Pfam profile PF00515: TPR Domain
[illegible]	[illegible]	1.0133849	1.1235884	1.014807	1.1203954	1.1322204	1.14168	0.9866151	1.4427972	0.853052	1.1110215	0.9264161	0.92729765	0.8031751	0.82204425	0.9491668	0.892914	1.068044	1.175828	0.954447	0.866825	1.12	1.36	AT5G64360	DNAJ heat shock N-terminal domain-containing protein low similarity to AHM1 [Triticum aestivum] GI:6691467; contains Pfam profile PF00226 DnaJ d[illegible]
[illegible]	[illegible]	1.0544494	1.2018578	1.1598152	0.99626017	0.8711556	0.790291	0.6853577	0.5054589	1.4287598	1.2687963	1.2415525	1.1617428	1.2116253	0.9981421	0.89224845	0.97056997	1.053096	0.713066	1.275213	1.018146	0.83	0.70	AT4G23890	expressed protein hypothetical protein, Synechocystis sp., PIR:S76577
[illegible]	[illegible]	1.0109483	1.0728185	1.1373568	1.0828037	1.2394217	0.986564	1.2618682	1.08023	0.9967025	0.97611856	1.0032977	0.82111335	0.92275864	0.96595603	0.87175953	0.9879643	1.075982	1.142069	0.949308	0.93711	1.13	1.22	AT4G24460	expressed protein
[illegible]	[illegible]	1.2975124	0.94737834	1.1324751	1.1469216	1.2556636	1.0551087	1.10075	0.9969399	0.86832076	0.9809681	1.0030601	1.0940431	0.77162504	0.65264046	0.95389396	0.79403126	1.131072	1.102116	0.986598	0.793048	1.15	1.39	AT5G64240	latex-abundant family protein (AMC3) / caspase family protein contains similarity to latex-abundant protein [Hevea brasiliensis] gb:AAD13216; contai[illegible]
[illegible]	[illegible]	1.0268282	1.1895826	1.0756769	1.1755569	1.1512989	1.0493256	1.0997771	0.93010914	0.92107165	0.97317183	0.9653717	0.90737534	0.88613236	0.9182383	0.7924154	1.0476689	1.116911	1.057628	0.941748	0.911114	1.19	1.16	AT1G47220	cyclin, putative similar to cyclin A-like protein [Nicotiana tabacum] GI:1064931, A-type cyclin [Catharanthus roseus] GI:2190259; contains Pfam profile[illegible]
[illegible]	[illegible]	1.0689156	0.8581945	1.029446	1.0608956	0.6739265	0.7949434	0.8971171	0.9437979	1.7986633	1.7915689	1.4256517	1.127704	1.1215558	0.97055393	0.87047374	0.96703863	1.004363	0.827446	1.535897	0.982406	0.65	0.84	AT1G11170	expressed protein contains Pfam profile PF05212: Protein of unknown function (DUF707)
[illegible]	[illegible]	1.0070537	0.840881	0.9088861	0.87830997	0.85805225	0.9963271	0.844421	1.003673	0.945729	1.098629	1.2197261	1.214113	1.0515478	1.0698154	1.1541557	0.9876574	0.908783	0.925618	1.119549	1.065794	0.81	0.87	AT5G61330	rRNA processing protein-related contains weak similarity to rRNA processing protein EBP2 (EBNA1-binding protein homolog) (Swiss-Prot:P36049) [Sacc[illegible]
[illegible]	[illegible]	0.881524	0.9839678	1.0890231	1.2574222	1.1836537	1.2762992	1.2296691	1.1810322	0.9161334	0.85008407	0.9463303	0.7441273	0.9530841	1.1046417	0.92415303	1.0160323	1.052984	1.217664	0.864169	0.999478	1.22	1.22	AT1G53490	bZIP protein
[illegible]	[illegible]	1.2409246	1.0002377	1.0303708	0.99976224	1.0912728	1.0643502	1.0172614	1.2786982	0.9468181	1.0335325	0.90069187	0.84746055	0.89477104	0.9073102	0.93526196	0.9369456	1.067824	1.112896	0.932111	0.918572	1.15	1.21	AT4G17300	asparaginyl-tRNA synthetase, chloroplast, mitochondrial / asparagine-tRNA ligase / AsnRS (SYNO) nearly identical to SP|O48593
[illegible]	[illegible]	1.0240207	1.0246394	0.8909219	1.1102246	1.0576372	1.1452111	1.0706283	1.2721713	0.8729454	1.0217306	0.978289	0.7440984	0.82906634	0.9295654	0.83870196	0.9012395	1.012452	1.136412	0.904261	0.874643	1.12	1.30	AT4G02070	DNA mismatch repair protein MSH6-1 (MSH6-1) (AGAA.3) identical to SP|O04716 DNA mismatch repair protein MSH6-1 (AtMsh6-1) cress) [Arabidops[illegible]
[illegible]	[illegible]	1.1770728	1.0499408	1.1904736	1.1554812	1.0275043	1.0328374	1.0543449	1.7125099	0.8839895	0.90251446	0.9724956	0.8247949	0.7892073	0.8921241	0.9150657	0.94194925	1.143242	1.206799	0.895949	0.884587	1.28	1.36	AT1G50430	7-dehydrocholesterol reductase / 7-DHC reductase / sterol delta-7-reductase (ST7R) / dwarf5 protein (DWF5) identical to SP|Q9LDU6 7-dehydrochole[illegible]
[illegible]	[illegible]	0.9641971	1.4471924	1.3037537	1.1829813	1.2955585	1.229902	1.0079018	1.0420058	0.8503829	0.9920981	0.8921316	0.95045626	0.8153327	1.0803598	0.81747156	0.9462198	1.224531	1.143842	0.921267	0.914846	1.33	1.25	AT5G27120;AT5G[illegible]	[AT5G27120, SAR DNA-binding protein, putative strong similarity to SAR DNA-binding protein-1 [Pisum sativum] GI:3132696; contains Pfam profile PF[illegible]
[illegible]	[illegible]	1.0577179	1.1605893	1.1249608	1.3785572	1.1232023	1.0223652	1.1041478	0.8948261	0.8300527	0.9812394	1.0187607	0.924866	0.9705065	0.93614936	0.833923	0.80604434	1.180456	1.036135	0.93873	0.886656	1.26	1.17	AT2G26390	serpin, putative / serine protease inhibitor, putative similar to phloem serpin-1 [Cucurbita maxima] GI:9937311; contains Pfam profile PF00079: Serp[illegible]
[illegible]	[illegible]	1.0529991	0.98466635	0.8683871	0.7844075	0.8906685	0.86652714	0.68772274	1.0147146	1.5246898	1.2796988	1.1818413	1.1240672	1.1123462	1.0167865	0.9852854	0.9463909	0.922615	0.864908	1.277574	1.015202	0.72	0.85	AT5G57960	GTP-binding family protein similar to SP|P25519 GTP-binding protein hflX (Escherichia coli)
[illegible]	[illegible]	[illegible]	[illegible]	[illegible]	[illegible]	[illegible]	[illegible]	[illegible]	[illegible]	[illegible]	[illegible]	[illegible]	[illegible]	[illegible]	[illegible]	[illegible]	[illegible]	[illegible]	[illegible]	[illegible]	[illegible]	[illegible]	[illegible]	[illegible]	[illegible]

251744_at	0.0196	0.9959771	1.013499	0.9717965	0.8635375	0.8975995	0.74759907	0.7230088	0.5202984	1.0741569	1.1472833	1.2957245	1.004023	1.0361487	0.95358616	1.063136	1.1264931	0.961203	0.722126	1.130297	1.044841	0.85	0.69	AT3G56010	expressed protein
256789_at	0.0195	1.0025849	1.7594117	2.4475675	2.417208	1.2342334	2.0451992	2.0955794	0.9911775	0.6073932	1.1338564	0.8577227	0.78766215	0.68765336	0.9838918	0.997415	0.979728	1.906693	1.591547	0.846659	0.912172	2.25	1.74	AT3G13672	seven in absentia (SINA) family protein low similarity to SP|P21461 Developmental protein seven in absentia (Drosophila melanogaster); contains Pfam profile PF03145: Seven in absentia protein family
253046_at	0.0195	0.42318878	0.36147407	0.62558603	0.4838446	0.7993073	1.3356555	0.78484833	1.3920738	1.1532407	0.9688259	1.153705	0.93901545	1.8530862	1.0379899	1.0311742	2.2601893	0.473523	1.077971	1.053697	1.54561	0.45	0.70	AT4G37370	cytochrome P450, putative similar to Cytochrome P450 91A1 (SP:Q9FG65)[Arabidopsis thaliana]; cytochrome P450, Glycyrrhiza echinata, AB001379
267571_at	0.0195	1.0547856	1.0695409	1.0515918	1.0852212	1.0286969	1.0146103	1.0014756	0.897628	0.96125245	0.8963915	0.8895224	0.84967464	0.96280324	0.9985243	1.0049777	0.92193	1.065285	0.985603	0.89921	0.972059	1.18	1.01	AT2G30650	3-hydroxyisobutyryl-coenzyme A hydrolase, putative / CoA-thioester hydrolase, putative strong similarity to gi:8572760; contains Pfam profile PF00388 enoyl-CoA hydratase/isomerase family protein
245165_at	0.0195	0.96649134	1.2033163	0.8316049	0.75337815	0.63786507	0.6200254	0.7312457	0.33030522	1.179508	1.2419087	1.2242117	0.998815	1.092368	1.001185	1.0048813	1.0770968	0.938698	0.57986	1.16111	1.043883	0.81	0.56	AT2G33180	expressed protein
249546_at	0.0195	0.7844059	0.86669904	0.73207885	0.63741946	0.70124936	0.8264929	0.66161203	1.2387109	1.2670876	1.0440167	1.0702167	1.4408487	0.9780715	1.0219283	1.1380252	1.1297151	0.755151	0.857016	1.205542	1.066935	0.63	0.80	AT5G38150	expressed protein
260014_at	0.0195	0.9391789	0.720731	1.0068084	0.8599923	0.90698254	0.82212865	0.8690253	0.93081754	1.2397147	1.05861	1.0442308	1.003939	1.1552068	1.0409014	0.99606097	1.0110233	0.881678	0.882239	1.086624	1.050798	0.81	0.84	AT1G68010	glycerate dehydrogenase / NADH-dependent hydroxypyruvate reductase identical to hydroxypyruvate reductase (HPR) GB:D85339 [Arabidopsis thaliana] (Plant Cell Physiol 1997 Apr;38(4):449-55)
265270_at	0.0195	0.86241513	0.94035894	0.90280133	0.89114296	1.6827602	1.533418	1.6124396	1.551013	0.69210666	0.8586678	0.77753115	0.83234084	1.3462555	1.059641	1.4440503	1.3134029	0.89918	1.594908	0.790162	1.290837	1.14	1.24	AT2G42910	ribose-phosphate pyrophosphokinase 4 / phosphoribosyl diphosphate synthetase 4 (PRS4) identical to phosphoribosyl diphosphate synthase (prs4) [Arabidopsis thaliana] GI:4902472
251157_at	0.0195	1.0004716	0.8786342	0.7791758	0.7490392	0.87990457	0.89039564	0.88695484	0.9995283	1.2255707	1.0066551	1.0392653	0.9184694	1.1385709	1.076763	1.0580951	1.083132	0.85183	0.914196	1.04749	1.089186	0.81	0.84	AT3G63140	mRNA-binding protein, putative similar to mRNA binding protein precursor (GI:26453355) [Lycopersicon esculentum]
259709_at	0.0195	0.9435985	1.1001294	1.0867023	1.3613343	1.3372695	1.2665747	1.0504487	1.0842253	0.9495514	0.7977476	0.84318507	0.9077605	0.918777	1.102465	0.8713219	0.8382906	1.122941	1.18463	0.874561	0.932714	1.28	1.27	AT1G77655	expressed protein
256830_at	0.0194	1.0398307	1.0833325	1.0338439	1.0940511	1.0434412	1.0124938	1.0190457	0.88581485	1.0099177	0.9900822	0.9622365	0.8993379	0.9219672	0.8999181	0.88743013	0.86682963	1.062765	0.990199	0.965394	0.894036	1.10	1.11	AT3G22860	eukaryotic translation initiation factor 3 subunit 8, putative / eIF3c, putative similar to eukaryotic translation initiation factor 3 subunit 8 (eIF3 p110) [Arabidopsis thaliana] SWISS-PROT:O49160
246952_at	0.0194	1.006161	0.7336653	0.8401151	0.8143968	0.70814407	1.0819775	0.99383897	0.7329679	1.3621435	0.9537849	0.95260096	1.5086569	1.3302106	1.3263122	1.4027702	1.1194092	0.848585	0.879232	1.194297	1.294676	0.71	0.68	AT5G04820	ovate family protein 62% similar to ovate protein (GI:23429649) [Lycopersicon esculentum]; contains TIGRFAM TIGR01568 : uncharacterized plant-specific domain TIGR01568
252927_at	0.0194	1.0109226	0.9799973	1.0586735	1.0979342	1.0607418	1.0077645	1.03768	1.0584943	0.96794444	1.0282856	0.9922355	0.896264	0.8270599	0.9017727	0.9399238	0.95196795	1.036882	1.04117	0.971182	0.905181	1.07	1.15	AT4G39090;AT4G[illegible]	[AT4G39090, cysteine proteinase RD19a (RD19A) / thiol protease identical to cysteine proteinase RD19a, thiol protease SP:P43296, GI:435618 from [Arabidopsis thaliana]];[AT4G39080, vacuolar proton ATPase, putative similar to Swiss-Prot:Q93050 vacuolar proton translocating ATPase 116 kDa subunit A isoform 1 (Clathrin-coated vesicle/synaptic vesicle proton pump 116 kDa subunit, Vacuolar prot[illegible]
265008_at	0.0194	1.2457001	0.8926626	0.91768765	1.7770042	1.6133044	2.010195	1.5065818	2.5760233	0.661259	0.48398754	0.83610934	0.9232841	1.076716	0.8164695	0.9104719	1.3929456	1.208264	1.926526	0.726127	1.049151	1.66	1.84	AT1G61560	seven transmembrane MLO family protein / MLO-like protein 6 (MLO6) idenctical to membrane protein Mlo6 [Arabidopsis thaliana] gi|14091582|gb|AAK53799; similar to MLO protein SWISS-PROT:P93766, NCBI_gi:1877221 [Hordeum vulgare][Barley]; contains Pfam profile PF03094: Mlo family
254945_at	0.0193	1.0018711	1.1038944	0.99446553	1.0233928	1.1764522	1.0591562	1.0841091	1.1507549	0.98895394	0.9981289	1.0283722	0.85551	0.98921967	0.96659005	0.8899802	0.87590575	1.030906	1.117618	0.967991	0.930424	1.06	1.20	AT4G10940	PHD finger family protein contains Pfam profile: PF00097 zinc finger, C3HC4 type
245098_at	0.0193	0.8240163	0.8360154	1.1094553	1.0011128	1.2992605	1.2109092	1.2578417	2.176711	0.55904007	0.6041691	0.75090986	0.6945962	0.8049965	1.0774176	1.1003939	0.998887	0.94265	1.486181	0.652179	0.995424	1.45	1.49	AT2G40940	ethylene response sensor / ethylene-responsive sensor (ERS) identical to ethylene response sensor (ERS) [Arabidopsis thaliana] GI:1046225
265443_at	0.0192	0.9719974	1.0819496	0.9726236	0.9914693	0.8173551	0.893005	0.9012801	0.83392483	1.0085306	1.2405932	1.0389875	0.96810144	1.2003274	1.3464373	1.0700289	1.047218	1.00451	0.861391	1.064053	1.166002	0.94	0.74	AT2G20750	beta-expansin, putative (EXPB1) identical to beta-expansin [Arabidopsis thaliana] gi|2224913|gb|AAB61709; similar to SP:O04701 major pollen allergen, Bermuda grass [Cynodon dactylon]; beta-expansin gene family, PMID:11641069
258162_at	0.0192	1.1507826	1.2269951	1.6403334	1.1759769	1.1007915	0.81287444	1.1123378	1.1478624	0.9726725	1.0273273	0.86597586	0.8876077	0.8255433	0.8820713	0.76277685	0.91338235	1.298522	1.043467	0.93896	0.845943	1.38	1.23	AT3G17810	dihydroorotate dehydrogenase family protein / dihydroorotate oxidase family protein low similarity to SP|Q12882 Dihydropyrimidine dehydrogenase [NADP+] precursor (EC 1.3.1.2) (DPD) (DHPDHase) (Dihydrouracil dehydrogenase) (Dihydrothymine dehydrogenase) (Homo sapiens); contains Pfam profile PF01180: Dihydroorotate dehydrogenase
247889_at	0.0191	0.9659853	0.831042	0.8789929	0.76578087	0.9227862	0.91153044	0.77337563	1.0057492	1.47522	1.3019043	1.0328281	1.0515165	1.2599334	1.0259889	0.99425095	1.0149467	0.86045	0.90336	1.215367	1.07378	0.71	0.84	AT5G57930	expressed protein
259658_at	0.0191	0.98102057	1.0012943	0.99019057	0.97425586	0.91024834	0.90744936	0.87570566	0.8287767	1.4980847	1.1019615	1.250091	0.99870574	1.2233286	1.0254381	1.0625656	1.030069	0.98669	0.880545	1.212211	1.086085	0.81	0.81	AT1G55370	expressed protein
252217_at	0.0191	0.94271135	1.3174304	1.105289	1.4081733	1.2440372	1.1477581	1.0701518	1.8636959	0.74574137	0.89865273	0.9009598	0.86580586	0.86542916	0.8548728	1.0393642	0.9606357	1.193401	1.335661	0.85279	0.930075	1.40	1.44	AT3G50140	expressed protein contains Pfam profile PF03140: Plant protein of unknown function
247577_at	0.0191	1.4869356	2.2793636	1.5260453	1.2637892	0.8650637	1.0415505	0.79522306	1.0136379	1.1267934	0.98636216	1.145277	0.97766775	0.8503605	0.72421604	0.6364213	0.74549836	1.639033	0.928869	1.059025	0.739124	1.55	1.26	AT5G61290	flavin-containing monooxygenase family protein / FMO family protein low similarity to FMO3 from Homo sapiens [SP|P31513]; contains Pfam profile: PF00743 Flavin-binding monooxygenase-like; supported by full-length cDNA Ceres:14492
246464_at	0.0191	1.0058057	1.1779246	1.3408741	0.81935114	0.40560764	0.58164465	0.6664291	0.52974665	2.986861	2.5337524	1.9408938	1.1598951	0.9941943	0.7929883	0.6685224	1.1052052	1.085989	0.545857	2.155351	0.890205	0.50	0.61	AT5G16990;AT5G[illegible]	[AT5G16990, NADP-dependent oxidoreductase, putative strong similarity to probable NADP-dependent oxidoreductase (zeta-crystallin homolog) P1 [SP|Q39172][gi:886428] and P2 [SP|Q39173][gi:886430], Arabidopsis thaliana];[AT5G17000, NADP-dependent oxidoreductase, putative strong similarity to probable NADP-dependent oxidoreductase (zeta-crystallin homolog) P1 [SP|Q39172][gi:88642[illegible]
250443_at	0.0191	0.7922646	0.8790741	1.3547909	1.4499426	1.2842473	1.541574	2.1794086	3.4325716	0.58748305	0.5756755	0.609633	0.70483387	0.8946563	0.97624606	1.023754	1.4546261	1.119018	2.10945	0.619406	1.087321	1.81	1.94	AT5G10520	protein kinase family protein contains protein kinase domain, INTERPRO:IPR000719
262135_at	0.0191	0.71485054	0.5910983	0.72663516	0.9131818	0.88059855	0.7655738	0.7904763	1.6963689	1.3225201	1.0329987	0.9670013	1.7890468	1.8315804	1.4038093	1.4903495	1.98093	0.736441	1.033254	1.277892	1.676661	0.58	0.62	AT1G78080	AP2 domain-containing transcription factor RAP2.4 identical to AP2 domain containing protein GI:2281633 from [Arabidopsis thaliana]
263119_at	0.0191	1.0373021	1.4506018	1.2466336	1.0091528	1.1421139	1.0070715	1.0212672	1.1845931	0.7738125	0.83100814	0.9611049	0.9236941	0.83715576	0.99292856	0.9271782	0.96490985	1.185923	1.088761	0.872405	0.930543	1.36	1.17	AT1G03110	transducin family protein / WD-40 repeat family protein contains 4 WD-40 repeats (PF00400); similar to WD-repeat domain 4 protein (GI:9955698) [Mus musculus]
256790_at	0.019	1.0121431	1.1274176	1.2236372	1.2768979	1.0990455	0.91477644	1.2149463	1.6860905	1.0553287	0.76217574	0.8801895	0.98785686	0.81326234	0.73780686	0.8441084	0.80036837	1.160024	1.228715	0.921388	0.798886	1.26	1.54	AT3G16857	two-component responsive regulator family protein / response regulator family protein contains Pfam profile: PF00072 response regulator receiver domain; similar to ARR1 protein GB:BAA74528 from [Arabidopsis thaliana] (Plant Cell Physiol. (1998) 39 (11), 1232-1239)
246598_at	0.019	1.0145692	1.2735448	1.0939621	1.214623	1.291571	0.91478276	1.1679838	1.4861171	1.0218922	0.8172129	0.8931656	0.9854309	0.8342299	0.85942376	0.9730731	0.82657045	1.149175	1.215114	0.929425	0.873324	1.24	1.39	AT5G14850	mannosyltransferase, putative similar to PIGB from Homo sapiens [gi:1552169], Mus musculus [gi:7634741]
266277_at	0.019	0.9265517	1.0094936	0.8584345	0.8135122	0.91383314	0.96516734	0.8086725	0.55397826	1.0921391	1.2132642	1.1528554	0.99050635	1.2874037	1.1534134	1.1025172	1.0988528	0.901998	0.810413	1.112191	1.160547	0.81	0.70	AT2G29310	tropinone reductase, putative / tropine dehydrogenase, putative similar to tropinone reductase SP:P50165 from [Datura stramonium]
261216_at	0.019	0.8053455	0.80788785	1.2424147	1.3655065	1.4425647	1.1062536	2.206158	1.4075737	0.68979377	0.5766273	0.617962	0.57248545	1.1483337	0.68971545	0.89374644	1.1486486	1.055289	1.540538	0.614217	0.970111	1.72	1.59	AT1G33030	O-methyltransferase family 2 protein similar to caffeic acid 3-O-methyltransferase [SP|Q00763] [Populus tremuloides], catechol O-methyltransferase [GI:4808524][Thalictrum tuberosum]
255574_at	0.019	1.250498	1.0073858	0.9932472	1.0302137	1.0418923	1.2541326	1.0630223	1.006753	0.94001186	0.7619108	0.92451173	0.7763351	1.033942	0.8724231	0.8627626	0.8644827	1.070336	1.09145	0.850692	0.908403	1.26	1.20	AT4G01420	calcineurin B-like protein 5 (CBL5) identical to calcineurin B-like protein 5 (GI:9965366) [Arabidopsis thaliana]; similar to N. crassa calcineurin calcium-regulated protein phosphatase, GenBank accession number P87072
252286_at	0.0189	1.033618	1.0398606	0.99614197	1.1107687	1.0833657	1.0096301	1.1894522	1.0072149	0.9408382	0.98753685	0.9605062	0.88844585	0.8694106	0.94419605	1.0038581	0.84546334	1.045097	1.072416	0.944332	0.915732	1.11	1.17	AT3G49070	expressed protein similar to At14a (GI:11994571 and GI:11994573) [Arabidopsis thaliana]
263657_at	0.0189	0.9778946	1.0830853	1.1062545	1.1489456	1.0242003	1.0503443	1.2137793	1.586774	1.0221053	0.95831706	0.91554946	0.8714389	0.8439843	0.7842367	0.8818644	0.92071676	1.079045	1.218774	0.941853	0.857704	1.15	1.42	AT1G04440	casein kinase, putative similar to casein kinase I [Arabidopsis thaliana] gi|1103318|emb|CAA55395; contains protein kinase domain, Pfam:PF00069
254682_at	0.0189	1.1503662	0.9819711	0.99396074	0.9775244	0.842211	0.88682914	0.9577696	0.64240116	1.4289147	1.2761778	1.1639689	1.2516576	1.1884563	1.0060394	1.0171239	0.9790386	1.025956	0.832303	1.28018	1.047665	0.80	0.79	AT4G13640	myb family transcription factor contains Pfam profile: PF00249 myb-like DNA-binding domain
250496_at	0.0189	1.3066595	1.5186076	1.0393953	1.2457118	1.0779934	0.95347977	1.0927123	0.9974479	1.002552	0.9523735	1.0890371	0.9392119	0.9361133	0.91993374	0.8767922	0.86147994	1.277594	1.030408	0.995794	0.898579	1.28	1.15	AT5G09650	inorganic pyrophosphatase family protein similar to SP|Q15181 Inorganic pyrophosphatase (EC 3.6.1.1) (Pyrophosphate [Homo sapiens]; contains Pfam profile PF00719: inorganic pyrophosphatase
253440_at	0.0189	0.9690935	0.99899614	0.7148724	0.7879061	0.8433785	0.9101531	0.6556337	0.96988255	1.2099184	1.2872115	1.067788	1.0010039	1.1482501	1.1089052	1.0314918	1.0603245	0.867717	0.844762	1.14148	1.087243	0.76	0.78	AT4G32570	expressed protein
251443_at	0.0189	1.0847888	1.1210942	1.1107749	0.9237044	1.1449482	1.0486157	1.1702807	1.0837299	0.98757577	0.8627115	0.96910036	0.9332726	0.9617045	1.0124243	0.98335505	0.9098079	1.060091	1.111894	0.938165	0.966823	1.13	1.15	AT3G59950;AT3G[illegible]	[AT3G59950, autophagy 4b (APG4b) identical to autophagy 4b [Arabidopsis thaliana] gi|19912145|dbj|BAB88384];[AT3G59940, kelch repeat-containing F-box family protein contains Pfam profiles PF01344: Kelch motif, PF00646: F-box domain]
264307_at	0.0189	1.1980674	1.1406839	1.1480011	1.4117005	1.0142893	0.92948925	0.96378773	1.1353625	0.9974844	0.9957004	1.0025356	1.1555982	0.8649706	0.83254844	0.90498763	0.7394797	1.224613	1.010732	1.037825	0.835497	1.18	1.21	AT1G61900	expressed protein contains similarity to glutamic acid/alanine-rich protein GI:6707830 from [Trypanosoma congolense]
250702_at	0.0189	1.2374797	1.031753	1.2830389	1.4211361	1.065942	0.9984356	1.0341936	0.93771875	0.9234995	1.0015644	0.96060854	0.866061	0.9446475	1.0208544	0.86080396	0.9303154	1.243352	1.009072	0.937933	0.939155	1.33	1.07	AT5G06730	peroxidase, putative similar to peroxidase [Arabidopsis thaliana] gi|1491617|emb|CAA68212
255779_at	0.0188	0.97056234	0.952244	0.6324022	0.86023057	0.9309936	0.9331129	0.8683984	0.4251823	1.3324287	1.5628219	1.1048514	1.4140085	1.5263577	1.2116928	1.3248744	1.0294377	0.85386	0.789422	1.353528	1.273091	0.63	0.62	AT1G18650	glycosyl hydrolase family protein 17 similar to beta-1,3-glucanase GI:15150341 from [Camellia sinensis]; C-terminal homology only
258425_at	0.0188	1.148673	1.277127	1.6200438	1.4248346	1.2010113	1.0300342	0.82379013	1.135165	0.9442843	0.9699659	1.030284	0.8818324	0.77337617	0.92793506	0.90158236	0.95515746	1.36767	1.0475	0.95692	0.889513	1.43	1.18	AT3G16760	tetratricopeptide repeat (TPR)-containing protein low similarity to TPR-containing protein involved in spermatogenesis TPIS [Mus musculus] GI:6272682; contains Pfam profile PF00515: tetratricopeptide repeat (TPR) domain
[illegible]	[illegible]	[illegible]	[illegible]	[illegible]	[illegible]	[illegible]	[illegible]	[illegible]	[illegible]	[illegible]	[illegible]	[illegible]	[illegible]	[illegible]	[illegible]	[illegible]	[illegible]	[illegible]	[illegible]	[illegible]	[illegible]	[illegible]	[illegible]	[illegible]	[illegible]

247533_at	0.0139	1.2465702	1.2420236	1.4390751	0.9839196	1.1777407	1.0646164	1.23369	1.8681824	0.8298671	0.7619099	0.87200046	0.9589341	0.7245702	0.65858525	1.0160805	0.96339846	1.227897	1.336057	0.855678	0.840659	1.43	1.59	AT5G61570	protein kinase family protein contains protein kinase domain, Pfam:PF00069
254040_at	0.0139	0.9301696	0.93637747	1.2759464	1.3974167	1.153538	1.2531682	1.3510586	1.2929009	0.7541648	0.7253112	0.7951295	0.83319813	1.0017453	0.85457647	0.99825454	1.215648	1.134978	1.262666	0.776951	1.017556	1.46	1.24	AT4G25900	aldose 1-epimerase family protein similar to apospory-associated protein C; APOC [Chlamydomonas reinhardtii] GI:6970044 Pfam profile PF01263: Aldose 1-epimerase
258196_at	0.0139	0.9089275	0.9685758	0.8336608	0.8661681	1.0314242	0.8470231	0.7327704	0.7194048	1.5495342	1.5562696	1.0743239	1.4555497	1.4334204	1.2347226	1.039145	0.8300793	0.894333	0.832656	1.408919	1.134342	0.63	0.73	AT3G13980	expressed protein
253412_at	0.0139	1.1099411	0.9677699	0.87052953	0.77332664	0.89820886	0.78190404	0.64544535	0.52979016	1.3318048	1.2274848	1.1204791	1.03223	1.286461	1.2413595	1.0790973	0.93818176	0.930392	0.713837	1.178	1.136275	0.79	0.63	AT4G33000	calcineurin B-like protein 10 (CBL10) identical to calcineurin B-like protein 10 [Arabidopsis thaliana] GI:20150248
264372_at	0.0139	1.1614772	1.384647	1.0042897	0.952471	1.3154609	1.1380233	1.3379033	1.059277	0.8487299	0.9987991	0.76729393	0.7866898	1.001201	0.88628596	0.90775937	0.97495884	1.125721	1.212666	0.850378	0.942551	1.32	1.29	AT1G11840	lactoylglutathione lyase, putative / glyoxalase I, putative highly similar to putative lactoylglutathione lyase SP:Q39366 from [Brassica oleracea]
257642_at	0.0139	0.925361	0.7238279	0.82671463	0.8742671	1.033824	0.79921246	0.85961545	1.0184102	1.0339744	1.0763031	0.9815898	1.2173264	1.193053	1.23059	0.97733265	1.2999264	0.837543	0.927766	1.077298	1.175226	0.78	0.79	AT3G25710	basic helix-loop-helix (bHLH) family protein contains Pfam profile: PF00010 helix-loop-helix DNA-binding domain
253578_at	0.0138	0.8670431	0.97224677	1.3600006	1.2038463	1.2437674	1.1533161	1.4513922	1.8824403	0.7566263	0.5686899	0.72806567	0.82129896	0.8003793	1.0013427	0.9986572	1.115848	1.100784	1.432729	0.71867	0.979057	1.53	1.46	AT4G30340	diacylglycerol kinase family protein contains INTERPRO domain, IPR001206, DAG-kinase catalytic domain
263996_at	0.0138	0.9936226	1.0622659	0.96913975	1.0070106	1.2835215	1.1743546	1.2181995	1.5584573	0.8312667	0.86089826	1.0063773	0.88297355	0.98845935	0.8240717	1.0830395	0.9825946	1.00801	1.308633	0.895379	0.969541	1.13	1.35	AT2G22530	phosphatidylinositolglycan-related similar to Pig-o [Mus musculus] GI:8099973
262411_at	0.0138	0.8286558	0.9884653	0.52993184	0.78985035	0.8550302	0.7440768	0.6819335	0.30258068	1.0115346	1.4225851	1.1827531	1.3282452	1.3771682	1.3559457	1.4561125	1.065343	0.784226	0.645905	1.23628	1.313642	0.63	0.49	AT1G34640	expressed protein
256251_at	0.0138	1.0429354	1.2544451	1.4734805	1.2084872	1.2001363	0.98862904	1.0155552	1.5686336	0.97096443	0.99525076	0.97097963	0.8808068	0.8424898	0.8196271	0.81377244	1.0047493	1.244837	1.214739	0.9545	0.87016	1.30	1.40	AT3G11330	leucine-rich repeat family protein
251478_at	0.0138	1.1132436	1.0113481	1.4069158	1.037892	1.2960646	1.0274335	1.0577629	1.1348029	0.9365075	0.9037185	0.9249952	0.83391225	0.9886517	0.8872706	0.90761036	0.9275794	1.14235	1.129016	0.899783	0.927778	1.27	1.22	AT3G59690	calmodulin-binding family protein similar to SF16 protein [Helianthus annuus] GI:560150; contains Pfam profile PF00612: IQ calmodulin-binding motif
265240_s_at	0.0138	1.0457518	0.9680047	1.0372524	1.0695605	1.0243363	1.0555441	1.0693191	1.0686551	0.9698883	1.0038464	0.9961536	0.7856075	0.8935421	0.85957825	0.9051811	0.81087416	1.030142	1.054464	0.938874	0.867294	1.10	1.22	ATMG01310;AT2	[ATMG01310, orf136b hypothetical protein];[AT2G07691, hypothetical protein]
256362_at	0.0138	1.0293951	0.998903	1.2146932	1.040913	1.1093217	1.0628282	1.1396579	1.0010968	0.9634724	1.0470409	0.9363443	0.89645004	0.84434813	0.9180731	0.95036244	0.81523305	1.070976	1.078226	0.960827	0.882004	1.11	1.22	AT1G66450	DC1 domain-containing protein contains Pfam profile PF03107: DC1 domain
250291_at	0.0138	1.0274259	0.9992782	1.0868729	1.0203252	1.0532609	1.0567819	1.1845026	1.3957514	0.9407837	0.9415879	0.9310029	0.9528768	0.91944057	1.0007219	0.8924073	0.8381384	1.033476	1.172574	0.941563	0.912677	1.10	1.28	AT5G13280	aspartate kinase identical to aspartate kinase [Arabidopsis thaliana] GI:4376158
256779_at	0.0138	1.0292741	1.1643317	1.0740047	1.1937346	1.0557506	1.2030114	1.0423019	0.93603075	0.94401246	0.9967993	0.9168406	0.9167818	0.840337	1.0032007	0.8808324	0.81440145	1.115336	1.059274	0.943609	0.884693	1.18	1.20	AT3G13784	beta-fructosidase, putative / beta-fructofuranosidase, putative / cell wall invertase, putative similar to beta-fructofuranosidase GI:402740 from [Arabidopsis thaliana]
252757_at	0.0138	1.138307	1.2203065	1.1708878	1.051481	1.1790167	1.0082401	1.1164178	0.9557639	0.99175984	0.89341044	1.0680753	0.97950494	0.97410214	0.89917755	0.82921994	0.8551795	1.145246	1.06486	0.983188	0.88942	1.16	1.20	AT3G42700	hypothetical protein
264240_at	0.0138	0.8682246	0.7613123	0.46686268	0.5708162	0.8871464	0.99033237	0.7303401	1.0681657	1.1689402	0.88930994	1.0096678	1.0167584	1.4561596	1.2041637	1.2261796	1.1728674	0.666804	0.918996	1.021169	1.264843	0.65	0.73	AT1G54820	protein kinase family protein contains protein kinase domain, Pfam:PF00069
257778_at	0.0138	1.013316	1.199225	1.0384942	1.0379249	1.0575751	1.0029109	1.0156056	1.0341076	0.99312586	0.9950494	0.9810755	0.9970892	0.95671797	0.8769145	0.9252855	0.895594	1.07224	1.02755	0.991585	0.913628	1.08	1.12	AT3G30843;AT3G	[AT3G30843, hypothetical protein];[AT3G29220, hypothetical protein]
249907_at	0.0138	0.962281	0.99010247	1.0269117	1.2448809	1.1929574	1.1356076	1.1056291	1.1712916	0.934946	1.0264475	1.0098976	0.9059143	0.96549815	0.86994916	0.88768506	0.9387825	1.056044	1.151371	0.969301	0.915479	1.09	1.26	AT5G22750	SNF2 domain-containing protein / helicase domain-containing protein / RING finger domain-containing protein [illegible]
255674_at	0.0137	0.83444417	0.9867088	0.68230385	0.63468754	0.96327454	1.0132911	0.7706338	0.6825853	1.0820651	1.151802	0.96280855	1.0622334	1.209714	1.225937	1.1125427	1.0803556	0.784536	0.857446	1.064727	1.157137	0.74	0.74	AT4G00430	plasma membrane intrinsic protein, putative identical to transmembrane protein [illegible]
251703_at	0.0137	1.0998495	0.98659563	1.0852257	1.0481763	1.0418906	1.0636722	1.0134044	1.0538629	0.9125854	0.8956148	0.9494085	0.95005214	0.8353176	1.0585892	0.89828324	0.9051689	1.054962	1.043208	0.926915	0.92434	1.14	1.13	AT3G56670	hypothetical protein hypothetical proteins - Arabidopsis thaliana
264423_at	0.0137	1.020212	1.0038177	1.3559178	1.3044429	1.0888213	0.99618226	1.2123901	1.7056828	1.0241678	0.7077789	0.86215705	0.93975323	0.6534863	0.663468	0.8249107	0.87010485	1.171098	1.250769	0.883464	0.752992	1.33	1.66	AT1G61690	tetratricopeptide repeat (TPR)-containing protein contains Pfam profile PF00515: TPR Domain
255859_at	0.0137	0.93375957	1.1601298	1.5351423	1.3044255	1.3827966	1.0358982	1.6696689	2.0194561	0.84821796	0.8115018	0.87086564	0.9641018	0.7663613	0.8449138	0.8757027	1.0845838	1.233364	1.526955	0.873672	0.89289	1.41	1.71	AT5G34930	arogenate dehydrogenase identical to arogenate dehydrogenase GI:16903098 from [Arabidopsis thaliana]; contains Pfam profile PF02153: prephenate dehydrogenase
264790_at	0.0137	0.631322	0.53476846	0.68583715	0.6127058	0.8239504	0.70774686	0.9000939	1.4673553	1.0365669	0.98300457	1.0366169	1.1718929	1.0169954	1.270216	1.2181672	1.2193761	0.616158	0.974787	1.05702	1.181189	0.58	0.83	AT2G17820	histidine kinase 1 99% identical to GP:4586626
246094_at	0.0137	0.9730434	1.1953472	1.2995427	1.2350571	1.0033348	1.0032862	1.1773361	1.2360834	0.82142204	1.0058784	0.81184757	0.8892358	0.9393691	0.9489449	0.9967138	0.93930733	1.175748	1.10501	0.882096	0.956084	1.33	1.16	AT5G19300	expressed protein contains Pfam profile PF02598: Uncharacterized [illegible]
248888_at	0.0137	0.8779427	0.6767437	0.6457662	0.59338236	0.57287467	0.6780604	0.6858019	1.194817	2.1943848	1.2510222	1.1747835	1.0222874	1.6054498	1.2376777	0.9777127	1.1811858	0.698459	0.782888	1.410619	1.250507	0.50	0.63	AT5G46240	inward rectifying potassium channel (KAT1) identical to K+ channel [illegible]
246289_at	0.0137	0.46323738	0.48437905	0.6217945	1.4777948	0.45476207	0.67224956	0.49845695	0.9286482	0.9578589	1.5748395	1.3763682	1.7481174	1.5243655	1.496474	1.042141	1.6400498	0.761801	0.638529	1.414296	1.425758	0.54	0.45	AT3G56880	VQ motif-containing protein contains PF05678: VQ motif
254669_at	0.0136	1.1718006	0.7100284	1.0044792	0.9207963	0.80694884	0.79755664	0.7120068	0.6819727	1.2414412	1.2255279	1.3149463	0.9677023	1.1675365	1.1521243	0.9955209	1.0212208	0.951776	0.749621	1.187404	1.084101	0.80	0.69	AT4G18370	protease HhoA, chloroplast (SPPA) (HHOA) identical to SP:Q9SEL7 [illegible]
256967_at	0.0136	0.89576596	0.7638756	0.75331295	0.6239038	0.9397577	0.98823464	0.89134054	1.1172593	1.3373576	1.3086	1.0148668	0.9963478	1.0931368	1.1203897	1.1536762	1.0036522	0.759215	0.984123	1.164293	1.092714	0.65	0.90	AT3G21060	transducin family protein / WD-40 repeat family protein contains [illegible]
245246_at	0.0136	1.1281347	1.0798153	1.2307391	1.0181646	1.1815168	1.1451073	1.1231244	0.96314996	1.0147225	0.81623	0.8831027	0.89549303	0.9599945	0.97829497	0.82953256	0.98527753	1.114213	1.103225	0.902387	0.938275	1.23	1.18	AT1G44222	hypothetical protein
264396_at	0.0136	1.0434622	0.98339367	1.1101924	1.0215831	1.1403322	1.0709778	1.0571809	1.3107437	1.0052736	0.9947265	0.90053034	0.96768665	0.9775523	0.9298381	0.9212337	0.88842987	1.039658	1.144809	0.967054	0.929263	1.08	1.23	AT1G12050	fumarylacetoacetase, putative similar to fumarylacetoacetase [illegible]
257416_at	0.0135	1.1108242	1.0638286	1.0297412	1.0631858	1.2754365	1.08571	1.1618315	0.9390871	0.8648115	0.9054563	0.8849428	0.87661964	0.96059996	0.83837646	0.9763188	1.0236813	1.066895	1.115516	0.882958	0.949744	1.21	1.17	AT2G17750	hypothetical protein contains Pfam profile PF03080: Arabidopsis [illegible]
264367_at	0.0135	1.0221518	1.010864	1.0987656	1.1837926	0.9761572	0.99457353	1.0475827	1.1208414	0.9664616	0.85744035	1.0054265	1.0190407	0.8484168	0.82999915	0.8976014	0.9082817	1.078894	1.034789	0.962092	0.871075	1.12	1.19	AT1G03350	BSD domain-containing protein contains Pfam profile PF03909: BSD [illegible]
255991_at	0.0135	1.140108	1.0473778	1.3929788	1.0384058	1.0383551	1.0042816	0.9939051	1.0415835	0.8765996	1.0360969	0.9957184	0.97367835	0.88378197	0.82071286	0.786512	0.8770236	1.154718	1.019531	0.970523	0.842042	1.19	1.21	AT1G29820	expressed protein
[illegible]																									

251706_at	0.00972	1.2606059	1.3043942	1.1891588	0.9430968	1.466776	1.061228	1.3753029	1.3021668	0.6816915	0.6890411	0.9400142	1.0129853	0.8800002	0.9050862	0.9870147	0.9431881	1.174314	1.301368	0.830933	0.928822	1.41	1.40	AT3G56620	integral membrane family protein / nodulin MtN21-related similar to MtN21 GI:2598575 (root nodule development) from [Medicago truncatula]
256171_s_at	0.00972	1.0637225	0.97629094	1.1755742	1.0584174	1.1872544	1.2148923	1.1112038	1.0584432	0.95746475	0.93506396	0.985108	0.95507425	1.0148919	0.8322778	0.9081721	0.9755124	1.068501	1.142948	0.958178	0.932714	1.12	1.23		
258803_at	0.00972	1.0555341	0.8672904	1.2974892	1.1873269	1.322055	1.0587726	1.4276026	1.3740591	0.77744514	0.7575933	0.8113504	1.0363891	0.96361077	0.8335243	0.8967449	0.87753963	1.10191	1.295622	0.845694	0.892855	1.30	1.45	AT3G04670	WRKY family transcription factor similar to elicitor response element binding protein WRKY3 isolog GB:AAB63078 [Arabidopsis thaliana]
267292_at	0.0097	1.0712861	1.0645944	1.1448802	1.0616202	1.2608879	1.1029804	1.0346782	1.0028985	0.9971015	0.9474099	0.927979	0.8281722	0.9878826	0.9254681	0.87628156	0.90968007	1.085595	1.100361	0.925166	0.924828	1.17	1.19	AT2G23730	expressed protein
245215_at	0.00966	1.0586418	0.9259047	0.74682516	0.7587093	0.7944061	0.9355482	0.86980915	0.74416226	1.2887595	1.216463	1.1463344	1.030079	1.3392922	1.1201837	0.969921	1.0824643	0.87252	0.835981	1.170409	1.127965	0.75	0.74	AT1G67830	GDSL-motif lipase/hydrolase family protein similar to early nodulin ENOD8 [Medicago sativa] GI:304037, elicitor-induced glycoprotein iEP4 [Daucus carota] GI:1911765, lanatoside 15'-O-acetylesterase [Digitalis lanata] GI:3688284; contains Pfam profile PF00657: Lipase/Acylhydrolase with GDSL-like motif
258394_at	0.00964	0.66882235	0.56889564	0.60833544	0.99863344	0.8580709	0.9298698	0.8596774	1.0013666	0.7333334	1.340534	1.30683	1.2767903	1.2188677	1.6046554	1.4702723	1.8414702	0.711172	0.912246	1.164372	1.533816	0.61	0.59	AT3G15530	expressed protein
255508_at	0.00964	1.0461817	1.191692	0.9850905	1.1631453	1.2605935	1.0336366	1.195436	1.1812842	0.8219525	1.0010239	0.85893345	0.9989761	0.8436539	0.9743156	0.968386	0.92473066	1.096527	1.167738	0.920221	0.927772	1.19	1.26	AT4G02220	zinc finger (MYND type) family protein / programmed cell death 2 C-terminal domain-containing protein similar to SP|Q16342 Programmed cell death protein 2 (Zinc finger protein Rp-8) [Homo sapiens]; contains Pfam profiles PF01753: MYND finger, PF04194: Programmed cell death protein 2, C-terminal putative domain
250011_at	0.00963	1.0280749	1.0552771	0.9633845	1.0995511	1.0494316	1.0382041	1.0886118	1.1672966	0.9982424	1.0017576	0.9413774	0.9700416	0.9383308	0.9618478	0.8776804	0.9390574	1.036572	1.085886	0.977855	0.929229	1.06	1.17	AT5G18470;AT5G	[AT5G18470, curculin-like (mannose-binding) lectin family protein contains Pfam profile: PF01453 lectin (probable mannose binding)];[AT5G18475, pentatricopeptide (PPR) repeat-containing protein contains Pfam profile PF01535: PPR repeat]
252547_at	0.00963	1.128681	1.0304118	1.188607	1.2368881	1.0437499	1.1132429	1.0773195	0.94921094	0.92554766	0.8896923	1.028246	0.92383254	0.9657087	0.97175395	0.8407106	0.8651305	1.146172	1.045881	0.94183	0.910826	1.22	1.15	AT3G45840	DC1 domain-containing protein contains Pfam profile PF03107: DC1 domain
246114_at	0.00962	1.0735793	1.014734	1.2194058	1.1120286	1.0527128	1.0361255	1.0264947	1.2768085	0.8433976	0.75391436	0.9852661	0.89611787	0.7538414	0.7973432	0.9796954	0.9059993	1.104937	1.098035	0.869674	0.85922	1.27	1.28	AT5G20250	raffinose synthase family protein / seed imbibition protein, putative (din10) similar to seed imbibition protein GB:AAA32975 GI:167100 from [Hordeum vulgare]; contains nonconsensus AT donor splice site at intron 1; contains Pfam profile PF05691: Raffinose synthase or seed imbibition protein Sip1; identical to cDNA seed imbibition protein (din10) partial cds GI:10834551
246507_at	0.00961	1.1490551	1.14686	1.4180622	1.2334687	1.1471093	0.97703934	1.1999956	1.014049	0.8742799	0.9859509	0.981002	1.0229753	0.86872846	0.7058042	0.9582437	0.9075499	1.236862	1.084548	0.966052	0.860082	1.28	1.26	AT5G16120	hydrolase, alpha/beta fold family protein similar to monoglyceride lipase from [Homo sapiens] GI:14594904, [Mus musculus] GI:2632162; contains Pfam profile PF00561: hydrolase, alpha/beta fold family
251066_at	0.00956	0.77280116	0.9432228	0.8863639	0.8278636	0.72179556	0.8688715	0.953139	0.46142676	1.2965381	1.1709443	1.0898763	1.2681049	1.2322409	1.1880814	1.2431487	1.046861	0.857563	0.751308	1.206366	1.177583	0.71	0.64	AT5G01880	zinc finger (C3HC4-type RING finger) family protein contains Pfam domain, PF00097: Zinc finger, C3HC4 type (RING finger)
259460_at	0.00955	0.78315073	0.7652418	0.5400231	0.528786	1.0314614	0.69139916	0.77820355	0.46904933	1.5271645	0.97581583	1.0394924	1.0241842	1.635	1.1351887	1.0839591	1.2452662	0.6543	0.742528	1.141664	1.274854	0.57	0.58	AT1G44000	expressed protein
257951_at	0.00955	0.8243424	0.59412086	0.73811764	0.68625206	0.78247714	0.97659695	0.98117507	1.0365832	1.0266759	1.2305259	1.0188248	1.0220999	1.1204338	1.0771494	0.9282342	1.2457007	0.710708	0.944208	1.074532	1.09288	0.66	0.86	AT3G21700	expressed protein
257285_at	0.00953	0.808021	0.95757675	0.85272175	0.8807009	0.8158768	0.7486059	0.8461599	1.1804253	1.4539273	1.2480922	1.3130667	1.2265223	1.3524604	0.98310536	1.0168946	1.0967798	0.874755	0.897767	1.310402	1.11231	0.67	0.81	AT3G29760	NLI interacting factor (NIF) family protein contains Pfam profile PF03031: NLI interacting factor
261905_at	0.00951	0.86538434	0.8308735	0.7055066	0.81819564	0.76900333	0.9094225	0.8054701	1.1173546	1.4447886	1.3406255	1.1745666	1.0052451	1.2912484	1.1725041	0.99475497	1.0153794	0.80499	0.900313	1.241306	1.118472	0.65	0.80	AT1G65070	DNA mismatch repair MutS family protein contains Pfam profile PF00488: MutS domain V
253751_at	0.0095	1.0219516	0.8080144	0.9689253	0.81582475	0.91603243	0.6802578	0.6531882	0.53553194	1.7359083	1.385486	1.1687492	1.0253692	1.3446326	1.1578681	0.99702024	1.0029798	0.903679	0.696253	1.328878	1.125625	0.68	0.62	AT4G29070	expressed protein
246864_at	0.00948	1.3344891	1.115761	1.1705105	1.2000606	1.1476892	0.8853788	1.3070982	1.2737936	0.7636219	0.9521414	1.0478586	0.77354985	0.86138076	0.9156836	0.86522985	0.8205674	1.205205	1.15349	0.884293	0.865715	1.36	1.33	AT5G25900	ent-kaurene oxidase, putative (GA3) / cytochrome P450 identical to GA3 [Arabidopsis thaliana] GI:3342249; similar to ent-kaurene oxidase [Cucurbita maxima] GI:11934675; contains Pfam profile PF00067: Cytochrome P450
251869_at	0.00948	0.7794673	0.60714704	0.74296534	0.62620515	0.8657885	0.7914735	0.8496881	1.2022485	1.8497223	1.4972801	0.9806671	1.019333	1.8046114	1.2710645	1.320061	1.0893418	0.688946	0.9273	1.336751	1.37127	0.52	0.68	AT3G54500	expressed protein
261804_at	0.00948	1.0025481	1.7614776	1.2170519	1.2427557	1.2638559	0.96392745	1.2860813	1.170159	0.70628494	0.8011745	0.82969344	0.7965108	0.99745196	0.94855356	0.86953574	1.0501573	1.305958	1.171006	0.783416	0.966425	1.67	1.21	AT1G30530	UDP-glucoronosyl/UDP-glucosyl transferase family protein contains Pfam profile: PF00201 UDP-glucoronosyl and UDP-glucosyl transferase
247980_at	0.00948	0.7108096	0.89269507	0.8688418	0.9257633	0.943579	0.81804174	0.7699379	1.0342993	1.7613219	0.96570075	1.0885742	1.2121547	2.1888661	1.6196182	1.2432661	1.3713318	0.849527	0.891464	1.256938	1.605771	0.68	0.56	AT5G56860	zinc finger (GATA type) family protein similar to unknown protein (pir |T04270)
245671_at	0.00937	1.2989426	1.4086636	1.3476995	1.2375557	1.6901978	0.9708697	1.1659495	1.017935	1.1704712	0.8298539	0.982065	0.8762436	0.7423111	0.7539896	0.6416284	0.898292	1.323215	1.211238	0.964658	0.759055	1.37	1.60	AT1G28230	purine permease (PUP1) identical to purine permease GI:7620007 from [Arabidopsis thaliana]
245853_at	0.00932	1.0564172	1.1989936	1.1760237	1.3333563	1.1107504	0.97337043	1.2099983	1.2178179	0.78233564	0.87082756	0.9920217	0.9705021	0.7341031	0.8526512	1.0079783	0.9271012	1.191198	1.127984	0.903922	0.880458	1.32	1.28	AT5G13500	expressed protein predicted protein At2g25260 - Arabidopsis thaliana, EMBL:AC007070
263184_at	0.00932	0.96379805	1.0218915	2.1092567	0.9781086	0.45100278	0.3450574	0.5280745	0.5504689	4.434536	2.6464448	2.4890194	1.7530087	1.6652703	0.8498108	0.724349	1.0927721	1.268264	0.468651	2.830752	1.083051	0.45	0.43	AT1G05560	UDP-glucose transferase (UGT75B2) similar to UDP-glucose:indole-3-acetate beta-D-glucosyltransferase GI:2149127 from [Arabidopsis thaliana]; identical to cDNA UDP-glucosyltransferase (UGT75B2) GI:13661274
263122_at	0.00932	0.77225894	0.97496474	0.8856276	0.62620384	0.8100546	0.8539732	0.9607075	0.96711993	1.7068126	1.3308659	1.1025419	1.0250351	1.7691956	1.3058847	1.1231158	1.2645403	0.814764	0.897964	1.291314	1.365684	0.63	0.66	AT1G78510	solanesyl diphosphate synthase (SPS) identical to solanesyl diphosphate synthase [Arabidopsis thaliana] GI:19911233; contains Pfam profile PF00348: Polyprenyl synthetase
250916_at	0.00932	0.97104543	1.1074274	1.7307504	1.2708309	1.0996236	1.0289546	1.040972	1.6141115	0.68824106	0.5154255	0.695123	0.6691651	0.7868887	0.78075665	0.7284212	1.1784483	1.270014	1.195915	0.641989	0.868654	1.98	1.38	AT5G03630	monodehydroascorbate reductase, putative monodehydroascorbate reductase (NADH), cucumber, PIR:JU0182
247125_at	0.00932	0.97880524	0.5370719	0.7971115	1.069396	0.6358837	0.8868883	0.7161803	0.6555047	1.0211948	1.6704916	1.4113302	1.2846671	1.1923566	1.4523582	0.97657347	1.3728548	0.845596	0.723614	1.346921	1.248536	0.63	0.58	AT5G66070	zinc finger (C3HC4-type RING finger) family protein contains Pfam profile: PF00097 zinc finger, C3HC4 type (RING finger)
257002_at	0.00932	0.9856702	1.2074461	1.1109048	1.1627597	1.4132745	1.0614488	1.3708934	1.7763733	0.91991866	1.0044265	0.82353747	0.85479414	0.86520904	0.95975673	0.970597	0.99557364	1.116695	1.405498	0.900669	0.947784	1.24	1.48	AT3G14100	oligouridylate-binding protein, putative similar to GB:CAB75429 (GI:6996560) from [Nicotiana plumbaginifolia], contains Pfam profiles: PF00076 RNA recognition motif (3 copies)
256429_at	0.00932	1.1560805	0.98510045	1.1858386	1.1203992	1.2194268	1.1733218	1.258991	1.754218	0.86122435	0.9274505	0.82771385	0.9665866	0.6290414	0.7694027	1.0148995	0.90985125	1.111855	1.351489	0.895744	0.830799	1.24	1.63	AT3G11040	glycosyl hydrolase family 85 protein contains similarity to endo-b-N-acetylglucosaminidase GI:13774138 from [Mucor hiemalis]
252014_at	0.00932	1.6684748	1.1288033	1.1009107	1.470602	1.3393533	0.93547	1.5936786	1.2846577	0.6639259	0.8570112	0.96867687	1.0313233	0.750227	0.6009579	0.690732	0.9532574	1.342198	1.28829	0.880234	0.748794	1.52	1.72	AT3G52870	calmodulin-binding family protein contains Pfam profile PF00612: IQ calmodulin-binding motif
251143_at	0.00932	0.70152897	0.7856737	0.7345116	0.6804148	0.8084295	0.8048388	0.8217835	1.2377461	1.2120396	1.1136109	0.9847618	1.015238	1.1964056	1.1169921	1.0224917	1.0413201	0.725532	0.918199	1.081413	1.094302	0.67	0.84	AT5G01220	UDP-sulfoquinovose:DAG sulfoquinovosyltransferase / sulfolipid synthase (SQD2) identical to GI:20302857
266746_s_at	0.00924	0.90718883	0.97698766	1.3621433	1.3131607	1.3552414	1.1226641	1.2030042	1.1404269	0.82295316	0.71901894	0.7888422	0.7838965	0.94635504	0.96857136	1.0533895	1.0230124	1.13987	1.205334	0.778678	0.997832	1.46	1.21	AT4G02520;AT2G	[AT4G02520, glutathione S-transferase, putative];[AT2G02930, glutathione S-transferase, putative]
258150_at	0.00923	1.0685174	0.9374547	0.84137136	0.7402035	0.74311006	0.86336327	0.8301581	1.0040883	1.3208414	1.3992051	1.2070005	1.1785718	0.9682075	1.2207419	0.99591166	1.0271891	0.896887	0.86018	1.276405	1.053013	0.70	0.82	AT3G18160	peroxin-3 family protein contains Pfam domain, PF04882: Peroxin-3
267151_at	0.0092	1.0711166	1.1479058	1.1330726	1.022968	1.2075778	1.1438868	1.0675628	1.3974044	0.7101035	0.8970812	0.9770319	0.8852111	0.6181557	0.60689473	0.8901133	0.9463584	1.093766	1.204108	0.867357	0.765381	1.26	1.57	AT2G30970	aspartate aminotransferase, mitochondrial / transaminase A (ASP1) identical to SP|P46643 Aspartate aminotransferase, mitochondrial precursor (EC 2.6.1.1) (Transaminase A) (Arabidopsis thaliana)
[illegible]																									

259126_at	0.00549	1.0140657	1.1581497	1.3010241	1.1330541	1.3946462	1.0859681	1.1128625	1.434646	0.89983374	0.95555913	0.812367	0.89721054	0.94072795	0.7888323	0.98593426	0.96512985	1.151573	1.257031	0.891243	0.920156	1.29	1.37	AT3G02280	flavodoxin family protein low similarity to SP|Q05001 NADPH-cyto [illegible]
261107_at	0.00548	1.1705375	1.2246509	1.5148841	1.2027026	1.3051888	0.9925654	1.4447844	2.0115986	1.0074346	0.852074	0.9101826	0.7746775	0.8822767	0.7347893	0.7214451	0.88959134	1.278194	1.438534	0.886092	0.807026	1.44	1.78	AT1G63010	SPX (SYG1/Pho81/XPR1) domain-containing protein contains Pfam [illegible]
264959_at	0.00543	0.9357902	1.0086515	0.7884205	0.7456096	0.74382013	0.60596144	0.6726669	0.3811187	1.4292458	1.2610043	1.1816015	1.01622	1.2976426	1.0909107	0.99134845	1.0513091	0.869618	0.600892	1.222018	1.107803	0.71	0.54	AT1G77090	thylakoid lumenal 29.8 kDa protein identical to SP|O49292|TL30_ [illegible]
248803_at	0.00542	1.0541403	1.0180279	1.1061959	1.1595316	1.2681172	1.2128148	0.98607546	1.2281569	0.87506807	0.93430847	0.9367674	0.93516755	0.8527777	1.0139244	0.86550295	0.9499875	1.084474	1.173791	0.920328	0.920548	1.18	1.28	AT5G47460	pentatricopeptide (PPR) repeat-containing protein contains Pfam p [illegible]
259595_at	0.00542	1.5436789	1.5220611	1.7172624	1.4292946	1.285544	0.91537356	1.0649958	1.3377936	0.6460061	0.9977742	0.9616466	1.0022258	0.47510526	0.59224457	0.7511933	0.95904416	1.553076	1.150927	0.901913	0.694397	1.72	1.66	AT1G28050	zinc finger (B-box type) family protein
259790_s_at	0.00542	0.887736	0.8809679	0.7670751	0.42090577	0.5530388	0.5600214	0.39050353	0.1418939	1.1558582	1.5169994	2.0990198	1.6662723	1.1122642	1.4181787	2.2124457	1.2138761	0.739171	0.411364	1.609537	1.489191	0.46	0.28	AT1G29430	auxin-responsive family protein similar to auxin-induced protein 10 [illegible]
258061_at	0.00542	1.1527343	1.1072825	1.4553211	1.2999085	1.1611106	1.0091679	1.0398962	1.1278634	0.87741905	0.98057884	0.9908322	0.9496873	0.8059966	0.7469475	0.93685627	0.96503305	1.253812	1.08451	0.949629	0.863708	1.32	1.26	AT3G25920;AT3 [illegible]	[AT3G25920, 50S ribosomal protein L15, chloroplast (CL15) identic [illegible]
255064_at	0.00542	1.614464	1.4189059	1.3526484	1.7321199	1.3124447	1.1550255	0.99608934	0.8471728	0.5050996	0.54322207	1.0039107	1.1372633	0.5282246	0.4398523	0.76876867	0.4188965	1.529535	1.077683	0.797374	0.538936	1.92	2.00	AT4G08950	phosphate-responsive protein, putative (EXO) similar to phi-1 (pho [illegible]
255403_at	0.00542	1.7246515	1.7061566	1.5236994	1.1829047	1.3653377	0.9544495	0.97463834	1.6084213	1.0253617	0.4595236	0.5996751	0.6846991	1.0321966	0.49576223	0.5226029	0.53196895	1.534353	1.225712	0.692315	0.645633	2.22	1.90	AT4G03400	auxin-responsive GH3 family protein similar to auxin-responsive GH [illegible]
266447_at	0.0054	0.49845815	0.46001813	0.5611468	1.3046201	0.77812064	0.81336504	0.6064064	0.6664161	0.7273392	1.1866349	1.3084548	2.059174	1.7017798	3.157559	2.1175492	2.7179344	0.706061	0.716077	1.320401	2.423706	0.53	0.30	AT2G43290	calmodulin-like protein (MSS3) identical to calmodulin-like MSS3 fr [illegible]
258448_at	0.00538	1.3202913	1.4874532	1.9142749	1.3719053	1.3566586	0.9084145	1.1534351	1.0060029	0.9533664	0.90741897	1.0537171	0.99399704	0.89215446	0.9068189	0.80932075	0.8762418	1.523481	1.106128	0.977125	0.871134	1.56	1.27	AT3G22290	expressed protein
253827_at	0.00537	1.8939772	1.6388849	2.51647	3.973399	3.0922678	2.7215438	2.8821297	0.7282522	0.34525883	0.51891655	0.75919646	0.70336115	0.88050437	0.6571084	0.7170759	1.1194956	2.505683	2.356048	0.581683	0.843546	4.31	2.79	AT4G28085	expressed protein
246170_at	0.00537	1.136333	1.1478696	1.3694423	1.3620867	1.0518255	1.1264982	1.1609488	0.9789666	0.9851986	0.8592072	0.96193206	1.0139045	0.9443022	0.9613729	0.9860954	0.8849264	1.253933	1.07956	0.955061	0.944174	1.31	1.14	AT5G32450	RNA recognition motif (RRM)-containing protein various predicted [illegible]
250980_at	0.00537	0.96915866	0.6284762	0.64565766	0.69213027	0.92063737	0.99870205	0.9172732	0.54834306	1.4446021	1.1478281	1.0302571	1.001298	1.8443311	1.5701306	1.5773753	1.2231258	0.733856	0.846239	1.155996	1.553741	0.63	0.54	AT5G03130	hypothetical protein
255115_at	0.00534	1.0890627	1.1665928	1.2952546	1.1230564	1.2035991	1.1316371	1.1467195	0.98058134	0.9326229	0.911362	0.9520735	0.9996697	0.9997503	1.0002496	0.8609507	0.8866163	1.168492	1.115634	0.948932	0.936892	1.23	1.19	AT4G08840	pumilio/Puf RNA-binding domain-containing protein contains simil [illegible]
255912_at	0.00533	1.1522065	1.2116127	1.2126778	1.1010528	1.0671217	1.0831947	0.9960972	0.9375161	1.0039029	1.021803	0.9652138	0.8679284	0.96401644	0.91371614	0.8785876	0.8995782	1.169387	1.020982	0.964712	0.914225	1.21	1.12	AT1G66960	lupeol synthase, putative / 2,3-oxidosqualene-triterpenoid cyclase, [illegible]
250074_at	0.00533	1.039103	1.0695256	0.9345806	0.92549163	0.6782975	0.8462848	0.6985676	0.907849	1.6285189	1.5036712	1.4449064	1.1811798	1.1321123	1.0859754	0.8775341	0.96089685	0.992175	0.78275	1.439569	1.01413	0.69	0.77	AT5G17310	UTP--glucose-1-phosphate uridylyltransferase, putative / UDP-gluc [illegible]
255117_s_at	0.00533	0.9425337	0.9224207	1.0006934	0.91824365	0.91861415	0.99930656	0.99637747	0.89280015	1.2147826	1.0946695	1.1547441	1.0928887	1.0497884	1.00336	1.0018479	0.9664056	0.945973	0.951775	1.139271	1.00535	0.83	0.95	AT4G08700	purine permease family protein similar to purine permease [Arabid [illegible]
249987_at	0.00532	1.0050075	0.96115524	1.5692769	1.6744343	1.3791158	1.5471396	1.6977361	2.3929381	0.5453692	0.65626305	0.83213145	0.68430907	0.73303163	0.99499243	0.9662993	1.2150906	1.302468	1.754232	0.679518	0.977353	1.92	1.79	AT5G18490	expressed protein
251178_at	0.00531	1.0417621	0.70446134	0.7921457	0.718557	0.6797039	0.78121024	0.7799768	0.9273437	1.3440592	1.2838697	1.0113685	1.3975707	1.5033919	1.266493	1.050237	0.9886314	0.814232	0.792059	1.259217	1.202188	0.65	0.66	AT3G63440	FAD-binding domain-containing protein / cytokinin oxidase family [illegible]
248158_at	0.0053	0.9973712	1.0560925	1.073599	1.0026288	1.087031	1.0276407	1.1151382	1.2752496	0.8387341	0.9370739	0.898494	0.85384136	0.8445432	1.0265971	0.8110071	0.8071129	1.032423	1.126265	0.882036	0.872315	1.17	1.29	AT5G54440	expressed protein
266015_at	0.00528	1.0966204	1.0503278	0.89578223	0.87303555	0.76908934	0.8630687	0.69291073	0.63985723	1.4730417	1.4791822	1.4056702	1.0708421	1.2045472	0.9771606	0.956048	1.0228394	0.978941	0.741232	1.357184	1.040149	0.72	0.71	AT2G24190	short-chain dehydrogenase/reductase (SDR) family protein contain [illegible]
254380_at	0.00524	1.1466604	1.2239802	1.1482333	1.4035852	1.130375	1.4160221	1.2068801	0.9348858	0.80822873	0.69321275	0.90855616	0.8121862	0.9089669	0.93652326	0.88646305	1.0634768	1.230615	1.172041	0.805546	0.948858	1.53	1.24	AT4G21865	expressed protein
258395_at	0.00523	1.2736856	1.3541107	1.1413356	1.2033594	1.7360083	1.5493218	1.1827447	1.0764226	0.40680793	0.6342208	0.92357737	0.82762706	0.73442084	0.8032742	0.72881055	0.8985652	1.243123	1.386124	0.698058	0.791268	1.78	1.75	AT3G15500	no apical meristem (NAM) family protein (NAC3) identical to AtNAC [illegible]
248179_at	0.00523	1.5818942	1.4076073	1.5865436	1.6494713	0.9201008	1.2336607	1.0704439	2.1690848	0.7254999	0.8028232	0.9295561	1.1622977	0.5500288	0.6218758	0.83739555	0.4720234	1.556379	1.348323	0.905044	0.620331	1.72	2.17	AT5G54380	protein kinase family protein contains protein kinase domain, Pfam [illegible]
267258_at	0.00521	1.1745436	1.0072123	1.2332004	0.9586628	1.2160391	1.2039416	1.1054755	1.678515	1.0198724	0.86627376	0.99278766	0.92920923	0.7913683	0.8021998	0.80322945	0.80537635	1.093405	1.300993	0.952036	0.800543	1.15	1.63	AT2G23140	armadillo/beta-catenin repeat family protein / U-box domain-cont [illegible]
258481_at	0.0052	0.99253005	1.071191	1.0881754	1.0943348	1.2903874	1.0305662	1.249473	1.2542267	0.83722436	0.97382104	0.95372564	0.94545674	0.9354617	0.8920157	1.0074699	0.96693593	1.061558	1.206163	0.927557	0.950471	1.14	1.27	AT3G02600	phosphatidic acid phosphatase family protein / PAP2 family protei [illegible]
262526_at	0.00513	0.9733418	0.8817742	0.7494154	0.553724	0.6988838	0.61907625	0.92498255	0.97126365	1.6801622	1.2009804	1.3265817	1.0266583	1.951706	1.556994	1.1923829	1.4101453	0.789564	0.803577	1.308596	1.527807	0.60	0.53	AT1G17050	geranyl diphosphate synthase, putative / GPPS, putative / dimethy [illegible]
260423_at	0.00513	1.3229572	1.0931382	1.4563732	1.4327064	1.1970794	0.924286	1.0652173	1.4624813	0.7465967	0.72164255	1.0448011	0.9551988	0.79275525	0.82297647	0.80305487	0.81138	1.326294	1.162266	0.86706	0.807542	1.53	1.44	AT1G72470	exocyst subunit EXO70 family protein contains Pfam domain PF03 [illegible]
266790_at	0.00511	0.7142229	0.88747233	0.5589757	0.6258157	0.7911987	1.0429016	0.6003313	0.56620234	0.9570985	1.2647142	1.1761869	1.1455798	1.0477896	1.2410948	1.3026448	1.169152	0.696622	0.750158	1.135895	1.19017	0.61	0.63	AT2G28950	expansin, putative (EXP6) similar to expansin Gi:2828241 from [Bra [illegible]
261365_at	0.00509	0.68086916	0.84356135	0.8290702	0.74801207	0.7574315	0.7648699	0.8642787	1.1153343	1.3369073	1.1307739	1.0392634	0.9607366	1.1414733	1.1912736	1.1491289	1.178909	0.775378	0.875479	1.11692	1.165196	0.69	0.75	AT1G53110	expressed protein
250330_at	0.00508	1.3862171	1.6040463	1.405099	1.4193325	1.6275365	1.0495033	1.5039765	0.941404	0.80374163	0.9526058	0.97703207	1.0229679	0.8142433	0.8689877	0.91813385	0.91200286	1.453674	1.280605	0.939087	0.878342	1.55	1.46	AT5G11810	expressed protein
266979_at	0.00507	0.77970725	0.63177913	0.629192	0.55744255	1.0289464	1.0410975	1.1200535	0.8139916	1.133922	0.9710536	0.8719827	0.75534976	1.6017948	1.5193449	1.3029959	1.3650177	0.64953	1.001022	0.933077	1.447263	0.70	0.69	AT2G39470	photosystem II reaction center PsbP family protein
253061_at	0.00507	1.0768745	0.8898285	0.9469623	0.9455722	0.85218805	0.7260071	0.8991011	0.78104806	1.0741684	1.2655265	1.5135245	1.3239148	1.2247674	1.1208683	0.94174474	1.0530376	0.96481	0.814586	1.294284	1.085105	0.75	0.75	AT4G37610	TAZ zinc finger family protein / BTB/POZ domain-containing protei [illegible]
246929_at	0.00506	0.9694181	0.867214	0.85178024	0.87165165	0.8438915	0.84029317	0.8105464	0.454955	1.030582	1.480367	1.365533	1.4006156	1.1450276	1.215918	1.2570399	1.3037509	0.890016	0.737422	1.319274	1.230434	0.67	0.60	AT5G25210	expressed protein
254573_at	0.00504	0.9939497	1.3601518	1.3185241	1.3177587	1.3528354	1.3168029	1.0143355	1.8382941	0.7544502	0.7523727	0.8302535	0.94149446	0.71994466	0.7411367	1.0060502	0.8889271	1.247596	1.380567	0.819643	0.839265	1.52	1.64	AT4G19420	pectinacetylesterase family protein contains Pfam profile: PF0328 [illegible]
261913_at	0.00504	1.5775717	2.648353	1.1174029	0.96175104	2.4091759	1.5741154	1.2061856	1.9054496	0.40801975	0.552967	0.73780984	0.23191993	0.34913912	0.6422842	0.41021326	1.038249	1.57627	1.773732	0.482679	0.609971	3.27	2.91	AT1G65860	flavin-containing monooxygenase family protein / FMO family prot [illegible]
254947_at	0.00503	1.0069227	0.9930774	1.0863822	1.0708152	1.1603591	1.2432971	1.1123899	1.1346108	0.94618505	0.9488435	0.93232554	0.9158148	0.87618715	0.9879899	1.0596406	0.86842185	1.039299	1.162664	0.935792	0.94806	1.11	1.23	AT4G11000	ankyrin repeat family protein contains ankyrin repeats, Pfam:PF00 [illegible]
267342_at	0.00502	0.8118718	1.020885	0.96812487	0.95107216	1.6830838	1.4551421	1.8154347	1.8553772	0.64468825	0.7290527	0.7316793	0.748507	1.1729549	0.97911507	1.2685305	1.4555165	0.937988	1.702259	0.713482	1.219029	1.31	1.40	AT2G44520	UbiA prenyltransferase family protein similar to SP|Q12887 Protob [illegible]
262770_at	0.00502	1.1604902	0.9987459	1.3458738	1.1200031	1.5772431	1.2153052	1.2791096	1.8657426	0.9574971	0.9969363	0.875353	0.9125118	0.81893873	0.77936924	0.9623027	1.001254	1.156278	1.48435	0.935575	0.890466	1.24	1.67	AT1G13190	RNA recognition motif (RRM)-containing protein
250972_at	0.00502	1.033096	0.8219656	0.966904	0.83103627	0.86971486	0.7242107	0.759557	0.70523405	1.7465367	1.3206178	1.188028	1.2838483	1.5709046	1.1431097	1.0759517	0.9305273	0.91325	0.764679	1.384758	1.180123	0.66	0.65	AT5G02840	myb family transcription factor contains PFAM profile: PF00249 my [illegible]
265868_at	0.00502	1.0464541	0.99821347	1.1972135	1.2542504	1.1718667	1.0630025	1.1755193	1.4296244	0.9758755	0.8473242	0.85781463	0.75417054	0.8733645	0.81868714	0.90538	1.0017865	1.124033	1.210003	0.858796	0.899805	1.31	1.34	AT2G01650	zinc finger (C2H2 type) family protein contains Pfam domain, PF00 [illegible]
248689_at	0.00498	0.7730422	0.6281696	0.84577554	0.6877884	0.76516604	0.8667077	0.6923063	1.081749	1.205459	1.2738506	1.0340477	0.99704087	1.002959	1.1607604	1.1251441	1.1985959	0.733694	0.851482	1.1276	1.121865	0.65	0.76	AT5G48150	phytochrome A signal transduction 1 (PAT1)
266485_at	0.00496	0.8489839	0.73097426	0.8172664	0.6653876	1.0491111	1.3155155	1.1407335	0.8865875	0.99195325	0.8214818	1.0080467	0.92712635	1.6399407	1.5998957	1.5685112	1.5492177	0.765653	1.097987	0.937152	1.589391	0.82	0.69	AT2G47630	esterase/lipase/thioesterase family protein similar to monoglyceri [illegible]
[illegible]	[illegible]	[illegible]	[illegible]	[illegible]	[illegible]	[illegible]	[illegible]	[illegible]	[illegible]	[illegible]	[illegible]	[illegible]	[illegible]	[illegible]	[illegible]	[illegible]	[illegible]	[illegible]	[illegible]	[illegible]	[illegible]	[illegible]	[illegible]	[illegible]	[illegible]

[illegible]

248870_at	0.0024	0.9517395	0.8234341	0.88797814	1.0482606	0.8626424	0.79927623	0.7156853	0.535767	1.3022966	2.142347	1.4867717	1.6185467	1.435203	1.5233711	0.93957675	1.3856248	0.927853	0.728343	1.637491	1.320944	0.57	0.55	AT5G46710	zinc-binding family protein similar zinc-binding protein [Pisum sativum] GI:16117799; contains Pfam profile PF04640 : Protein of unknown function, DUF597
257194_at	0.00239	1.0766212	1.3356415	1.0529575	0.9239925	1.404506	1.3383908	1.2524563	1.4613788	0.5156084	0.6650012	0.68961895	0.7170753	0.9470425	0.9072618	0.84046197	1.2650219	1.097303	1.364183	0.646826	0.989947	1.70	1.38	AT3G13110	serine O-acetyltransferase (SAT-1) identical to serine acetyltransferase (Sat-1) GI:1184048 [Arabidopsis thaliana]
246335_at	0.00239	1.2087787	1.571113	1.3542347	1.6433246	1.5094812	0.87136775	1.3882649	1.356716	1.0049111	0.8944174	0.9950889	0.9493486	0.8363432	0.65110844	0.68640375	0.7815091	1.444363	1.281457	0.960942	0.738841	1.50	1.73	AT3G44880	Rieske [2Fe-2S] domain-containing protein similar to lethal leaf-spot 1 from Zea mays [gi:1935909]; contains Pfam PF00355 Rieske [2Fe-2S] domain
257474_at	0.00239	0.9132297	0.77869314	0.53636545	0.6819154	0.7344833	0.7621515	0.7584123	0.73271465	1.8145329	1.3812469	1.1838088	1.1678813	2.1361275	1.4145633	1.0867702	1.1969393	0.727551	0.74694	1.386867	1.4586	0.52	0.51	AT1G80850	methyladenine glycosylase family protein similar to SP|P05100 DNA-3-methyladenine glycosylase I (EC 3.2.2.20) (3-methyladenine-DNA glycosylase I, constitutive) (Escherichia coli); contains Pfam profile PF03352: Methyladenine glycosylase
266163_at	0.00239	1.000629	1.3662258	1.3944621	1.6358194	1.8340237	1.1985332	1.7680432	1.7038453	0.999371	0.5663994	0.8493941	0.8421383	0.9507609	0.6470911	0.80147976	0.79783714	1.349284	1.626111	0.814326	0.799292	1.66	2.03	AT2G28130	expressed protein
248908_at	0.00239	0.49214873	0.6201889	0.59612983	0.48038602	0.6599735	0.6712547	0.7715485	1.2408539	1.3189435	1.1687645	0.97039217	1.0296078	1.2890829	1.046667	1.1252006	1.25198	0.547213	0.835908	1.121927	1.178233	0.49	0.71	AT5G45800	leucine-rich repeat transmembrane protein kinase, putative
256527_at	0.00237	1.5427297	2.048046	1.8232251	1.8614604	1.9817486	1.2217705	1.568216	0.9209907	0.98584247	0.77445954	0.67622364	0.48731038	1.0141574	0.6579796	0.6317918	0.80429286	1.818865	1.423181	0.730959	0.777055	2.49	1.83	AT1G66100	thionin, putative similar to thionin [Arabidopsis thaliana] GI:1181533
261354_at	0.00237	1.022039	0.99617416	1.1457078	1.179313	1.5363998	1.303644	1.774752	2.3765972	0.84877604	0.9201083	0.8258097	0.8453878	1.0038259	0.91789967	0.8813241	0.9305088	1.085463	1.747848	0.86002	0.93339	1.26	1.87	AT1G79690	MutT/nudix family protein contains Pfam NUDIX domain [PF00293]; very low similarity to Chain A and Chain B of Escherichia coli isopentenyl diphosphate:dimethylallyl diphosphate isomerase [gi:15826361] [gi:15826360]
245279_at	0.00236	1.2181079	1.1794748	1.3194143	1.3814703	1.0248396	1.0010085	1.0002985	1.245429	0.89268506	0.9810007	0.9997016	0.9912895	0.8258987	0.77822363	0.9515283	0.8809864	1.274617	1.067894	0.966169	0.859159	1.32	1.24	AT4G17270	Mo25 family protein similar to MO25 protein (early mouse development protein family) [Mouse] SWISS-PROT:Q06138
258987_at	0.00236	1.1219922	1.1254065	1.1943208	1.1960756	0.9617825	1.1021206	1.1080551	1.0559709	0.813594	1.0203568	0.8576207	0.9045118	0.88604695	0.9796433	0.8999354	0.9279288	1.159449	1.056982	0.899021	0.923389	1.29	1.14	AT3G08950	electron transport SCO1/SenC family protein similar to SP|P23833 SCO1 protein, mitochondrial precursor (Saccharomyces cerevisiae); contains Pfam profile PF02630: SCO1/SenC
254298_at	0.00234	1.0008067	0.95732266	0.99919343	0.9386353	0.95272577	0.8606736	0.8204104	0.87171525	1.2933245	1.1819507	1.2272452	1.0758734	1.2210473	1.1924598	1.0520842	0.98715	0.97399	0.876381	1.194598	1.113185	0.82	0.79	AT4G22890	expressed protein
247118_at	0.00234	0.9908083	0.6345244	0.678164	0.68440425	0.64961493	0.72948956	0.8566763	0.7585034	1.5052437	1.4093928	1.1280998	1.0991695	1.4851816	1.2684213	1.0268964	1.0091918	0.746975	0.748571	1.285476	1.197423	0.58	0.63	AT5G65890	ACT domain-containing protein (ACR1) contains Pfam profile ACT domain PF01842
252858_at	0.00233	0.9562558	0.79588115	0.756846	0.6248315	0.6749772	0.6905988	0.804417	0.66085154	1.1518124	1.2739143	1.2984493	1.3572855	1.0094012	1.0573024	1.0715063	1.1111258	0.783454	0.782711	1.270365	1.062334	0.62	0.74	AT4G39770	trehalose-6-phosphate phosphatase, putative similar to trehalose-6-phosphate phosphatase (AtTPPB) [Arabidopsis thaliana] GI:2944180; contains Pfam profile PF02358: Trehalose-phosphatase
258059_at	0.00232	0.7178178	0.9316618	1.0683382	0.90134424	0.7692978	0.85487944	0.6945634	0.66221987	1.5423176	1.6825666	1.5539509	0.92167264	1.5270721	1.5183786	1.2264673	1.363293	0.904791	0.745221	1.425127	1.408803	0.63	0.53	AT3G29035	no apical meristem (NAM) family protein contains Pfam PF02365 : No apical meristem (NAM) protein; similar to cup-shaped cotyledon CUC2 (GI:1944132) [Arabidopsis thaliana]
263539_at	0.00232	1.0943864	2.6079583	1.09848	1.2438526	1.7651806	1.5020211	3.292882	3.793039	0.60422283	0.1861324	0.28954798	0.13386759	0.90560365	0.48744175	0.34449875	0.9005641	1.511172	2.588281	0.303443	0.659527	4.98	3.92	AT2G24850	aminotransferase, putative similar to nicotianamine aminotransferase from Hordeum vulgare [GI:6498122, GI:6469087]; contains Pfam profile PF00155 aminotransferase, classes I and II
249392_at	0.00232	0.9792563	0.78860986	0.7432016	0.6379128	0.7326698	0.92122656	0.5896074	0.6634338	1.0207437	1.2294275	1.2669032	1.3343207	1.1718938	1.1736716	1.0655415	1.0460557	0.787245	0.726734	1.212849	1.114291	0.65	0.65	AT5G40150	peroxidase, putative identical to peroxidase ATP26a [Arabidopsis thaliana] GP|1890317|emb|CAA72487
265990_at	0.00231	0.89275044	0.76250887	0.7482874	0.6698345	0.98976433	0.93055713	0.8552092	1.1172391	1.3691952	1.3732189	0.9930902	1.0069098	1.6193649	1.4267646	1.3096215	1.30096	0.768345	0.973192	1.185604	1.414178	0.65	0.69	AT2G24280	serine carboxypeptidase S28 family protein contains Pfam profile: PF05577 Serine carboxypeptidase S28
253435_at	0.00231	1.2946603	1.2271165	1.1659718	1.0347118	1.1758099	0.9991775	1.015143	1.0538338	0.90768737	0.9131787	1.0008224	0.9366088	0.86117333	0.9315475	0.8840886	0.87506074	1.180615	1.060991	0.939574	0.887968	1.26	1.19	AT4G32400	mitochondrial substrate carrier family protein
262883_at	0.00231	0.8861184	0.8168857	0.89406955	0.7978332	0.7406589	0.847228	0.7778862	0.7539817	2.750048	2.0915833	1.5876784	1.1059304	2.0908415	1.3171425	1.2058641	1.6045638	0.848727	0.779939	1.88381	1.554603	0.45	0.50	AT1G64780	ammonium transporter 1, member 2 (AMT1.2) nearly identical to SP|Q9ZPJ8 Ammonium transporter 1, member 2 (AtAMT1;2) (Arabidopsis thaliana)
254385_s_at	0.0023	1.5108463	3.6294508	1.8255175	1.0819232	1.5745996	1.7195582	1.9409479	0.8955787	0.46086442	0.47689256	0.42924795	0.39660895	0.95123255	1.0487674	0.68111026	0.7603625	2.011934	1.532671	0.440903	0.860368	4.56	1.78	AT4G21840;AT4G	[AT4G21840, methionine sulfoxide reductase domain-containing protein / SelR domain-containing protein weak similarity to pilin-like transcription factor [Homo sapiens] GI:5059062, SP|P14930 Peptide methionine sulfoxide reductase msrA/msrB (EC 1.8.4.6) (Neisseria gonorrhoeae); contains Pfam profile PF01641: SelR domain];[AT4G21830, methionine sulfoxide reductase domain-containing prote[illegible]
248853_at	0.0023	0.86507577	0.8565536	0.6861607	0.704183	0.69998664	0.8079806	0.746543	1.0074098	1.196726	1.1069067	1.0811667	1.260692	1.1956062	1.1661855	0.99259007	1.0215534	0.778003	0.81548	1.161373	1.093984	0.67	0.75	AT5G46570	protein kinase family protein contains protein kinase domain, Pfam:PF00069
257798_at	0.0023	1.5822064	2.3070636	1.6428658	1.2442553	1.0821457	0.9370698	1.0629302	1.2437629	1.1293949	0.52785414	0.6741202	0.85983425	0.719212	0.5565529	0.58820045	0.53804654	1.694098	1.081477	0.797801	0.600503	2.12	1.80	AT3G15950	DNA topoisomerase-related similar to DNA topoisomerase IV subunit A (GI:26454107) [Mycoplasma penetrans]
247977_at	0.00228	0.7579129	0.7240009	0.4466032	0.46065497	0.72029215	0.8621676	0.6625353	1.1378324	1.4754959	1.2326719	1.2784048	0.85084164	1.8366172	1.8394622	1.4914387	1.4093057	0.597293	0.845707	1.209354	1.644206	0.49	0.51	AT5G56850	expressed protein
254305_at	0.00224	0.69823605	0.61549306	0.49630597	0.3511995	0.7353263	0.67161894	0.5926949	0.97119415	1.7462825	1.5067289	1.0892202	1.0901079	1.7964987	1.273887	1.028806	1.1985725	0.540309	0.742709	1.358085	1.324441	0.40	0.56	AT4G22200	potassium channel protein 2 (AKT2) (AKT3) identical to potassium channel [Arabidopsis thaliana] gi|1100898|gb|AAA97865; Note: also identical to AKT3 [Arabidopsis thaliana] gi|1172218|gb|AAA96153, which is a truncated version of AKT2, PMID:10852932; member of the 1 pore, 6 transmembrane (1P/6TM- Shaker-type) K+ channel family, PMID:11500563; identical to cDNA inward-rectifying K+ ch[illegible]
260076_at	0.00224	0.6643902	0.9770361	0.58216983	0.69494283	0.6524243	0.64554787	0.6788812	0.3727799	1.5906329	1.6617693	1.0229641	1.1833792	1.7840519	1.2801663	1.0590794	1.2607039	0.729635	0.587408	1.364686	1.346	0.53	0.44	AT1G73630	calcium-binding protein, putative similar to calcium binding protein GI:14589311 from [Sesbania rostrata[; contains Pfam profile: PF00036 EF hand (4 copies)
246408_at	0.00224	1.1343056	1.1000143	1.3844807	1.2508342	1.186295	1.1091253	1.0879031	1.7783382	0.63927656	0.6722749	0.824448	0.91209674	0.59050554	0.66059225	0.9001945	0.6524726	1.217409	1.290415	0.762024	0.700941	1.60	1.84	AT1G57680	expressed protein
256415_at	0.00223	1.7184775	1.7005978	1.7750919	1.8613993	1.0076361	1.0242976	0.992364	0.6596744	0.98368096	0.83022785	1.0476694	1.0398592	0.69328415	0.8573168	0.7348239	0.7127718	1.763892	0.920993	0.975359	0.749799	1.81	1.23	AT3G11210	GDSL-motif lipase/hydrolase family protein contains Pfam profile PF00657: Lipase/Acylhydrolase with GDSL-like motif
259382_s_at	0.00223	1.7996457	2.6958344	1.8946475	1.5969198	2.0720086	1.22366	1.2837349	0.99062663	1.0093734	0.9658993	0.8729961	0.7957876	0.97153896	0.77347	0.7099879	0.7856384	1.996762	1.392508	0.911014	0.810159	2.19	1.72	AT3G16430;AT3G	[AT3G16430, jacalin lectin family protein similar to myrosinase binding protein [Brassica napus] GI:1711296; contains Pfam profile PF01419 jacalin-like lectin domain];[AT3G16420, jacalin lectin family protein similar to myrosinase binding protein [Brassica napus] GI:1711296; contains Pfam profile: PF01419 jacalin-like lectin domain]
251062_at	0.00223	1.0024514	1.0736599	1.158434	1.1709807	1.446265	1.1517466	1.2909024	1.1045344	0.9405566	0.94904244	0.92600274	0.904822	0.833053	0.88820857	0.8752076	0.9975485	1.101382	1.248362	0.930131	0.898492	1.18	1.39	AT5G01840	ovate family protein 59% similar to ovate protein (GI:23429649) [Lycopersicon esculentum]; contains TIGRFAM TIGR01568 : uncharacterized plant-specific domain TIGR01568
253203_at	0.00223	1.1776752	1.3343763	1.644185	1.3273487	2.230144	1.0550886	1.8671572	2.5192785	0.9449115	0.7109984	0.612727	0.5144228	0.87221795	0.7044133	0.59689355	0.88634086	1.370896	1.917917	0.695765	0.764966	1.97	2.51	AT4G34710	arginine decarboxylase 2 (SPE2) identical to SP|O23141 Arginine decarboxylase 2 (EC 4.1.1.19) (ARGDC 2) (ADC 2) (ADC-N) (Arabidopsis thaliana)
260547_at	0.00223	1.3788825	1.8018869	1.0765187	1.3211114	2.2324808	1.6447299	1.920167	0.9234819	0.59902006	0.47137633	0.5671238	0.33775046	1.1202651	0.71271944	0.61171496	0.82878923	1.3946	1.680217	0.493818	0.818372	2.82	2.05	AT2G43550	trypsin inhibitor, putative similar to SP|P26780 Trypsin inhibitor 2 precursor (MTI-2) (Sinapis alba)
256341_at	0.00223	1.1826218	1.1862782	1.0944959	1.0033627	1.5088114	1.2160791	1.3750837	1.930054	0.92314595	0.94224703	0.84989023	0.82365996	0.8602595	0.9260677	0.9545283	0.9966374	1.11669	1.507507	0.884736	0.934371	1.26	1.61	AT1G72040	deoxynucleoside kinase family contains Pfam profile: PF01712 deoxynucleoside kinase
261727_at	0.00223	1.4041351	1.1248415	1.2106085	1.486481	1.2336859	0.99202585	1.3738956	1.0264531	0.8149844	0.90712506	0.80018973	1.0079743	0.71944475	0.6643001	0.8772083	0.5661581	1.306517	1.156515	0.882568	0.706778	1.48	1.64	AT1G76090	S-adenosyl-methionine-sterol-C-methyltransferase identical to S-adenosyl-methionine-sterol-C-methyltransferase GI:2246456 from [Arabidopsis thaliana]
250662_at	0.00221	1.0695031	3.2781363	2.3715572	1.8196398	2.7681594	1.0657482	3.4193075	2.5593455	0.9342517	0.789807	0.57726866	0.39188504	0.6279874	0.5240642	0.41308144	0.70060027	2.134709	2.45314	0.673303	0.566433	3.17	4.33	AT5G07010	sulfotransferase family protein similar to steroid sulfotransferase 3 [Brassica napus] GI:3420008, steroid sulfotransferase 1 [Brassica napus] GI:3420004; contains Pfam profile PF00685: Sulfotransferase domain
266348_at	0.00221	1.2763494	1.0609034	1.1884619	1.279667	1.3655488	0.94855136	1.2975365	1.1483228	0.81381774	0.83628833	1.044619	0.9557387	0.7439638	0.66843945	0.8127599	0.8091328	1.201345	1.18999	0.912527	0.758574	1.32	1.57	AT2G01450	mitogen-activated protein kinase, putative / MAPK, putative (MPK17) mitogen-activated protein kinase (MAPK)(AtMPK17), PMID:12119167
258259_s_at	0.00221	1.145739	1.2792991	1.5276208	1.5405347	1.6513382	1.0107398	1.250549	1.3311145	0.77280813	0.74864244	0.9323058	0.87635183	0.83493	0.8768042	0.9362752	0.98926026	1.373298	1.310935	0.832527	0.909208	1.65	1.44	AT3G26840	esterase/lipase/thioesterase family protein contains Pfam profile PF03096: Ndr family
248795_at	0.0022	0.9258586	0.86056983	0.83741283	0.7631551	0.9867685	0.9206762	0.77816856	0.78276813	1.0908655	1.2685593	1.2486473	1.1560316	1.0753199	1.2874982	1.0476844	1.0132315	0.846749	0.867095	1.191021	1.105934	0.71	0.78	AT5G47390	myb family transcription factor contains Pfam profile: PF00249 myb-like DNA-binding domain
252520_at	0.0022	0.5927633	0.495543	0.47282985	0.4479928	0.45877674	0.7466253	0.48175794	1.068812	1.4941627	1.160366	1.04219	0.9731318	1.3312705	1.50638	1.153212	1.0268681	0.502282	0.688993	1.16752	1.254433	0.43	0.55	AT3G46370	leucine-rich repeat protein kinase, putative similar to light repressible receptor protein kinase [Arabidopsis thalian] gi|1321686|emb|CAA66376; contains leucine rich repeat (LRR) domains, Pfam:PF00560; contains protein kinase domain, Pfam:PF00069
247323_at	0.00219	0.9195508	0.5047538	0.6219247	0.4162712	0.7483005	0.69667387	0.51841974	0.8346303	2.049332	1.3423067	1.2427237	1.0804491	1.6936182	1.5985849	1.1582448	1.2671696	0.615625	0.699506	1.428703	1.429404	0.43	0.49	AT5G64170	dentin sialophosphoprotein-related contains weak similarity to Swiss-Prot:Q9NZW4 dentin sialophosphoprotein precursor [Homo sapiens]
256266_at	0.00219	0.8258795	0.68525594	0.615925	0.5178167	0.8141228	0.84905237	0.8636909	0.59566045	2.4087262	1.4501003	1.1363091	1.2029883	4.0603557	2.105436	1.4682778	1.8765988	0.661219	0.780632	1.549531	2.377667	0.43	0.33	AT3G12320	expressed protein
252011_at	0.00219	1.0853547	0.92291176	0.78247094	0.7297442	0.8140179	0.69764984	0.4257819	0.51542956	1.7809509	1.8408107	1.6886504	1.88342	1.8173608	1.0770881	1.1347613	0.87610495	0.88012	0.61322	1.798458	1.226329	0.49	0.50	AT3G52720	carbonic anhydrase family protein low similarity to storage protein (dioscorin) [Dioscorea cayenensis] GI:433463; contains Pfam profile PF00194: Eukaryotic-type carbonic anhydrase
249847_at	0.00219	1.0787555	1.2122437	1.1335417	1.6071837	1.2815694	1.5349151	1.1659876	2.0795982	0.74561083	0.7325602	0.7836935	0.8928766	0.8294802	0.81225866	0.92124444	0.86927754	1.257931	1.515518	0.788685	0.858065	1.59	1.77	AT5G23210	serine carboxypeptidase S10 family protein similar to Serine carboxypeptidase II chains A and B (SP:P08819) (EC 3.4.16.6) [Triticum aestivum (Wheat)];
263120_at	0.00219	1.6705824	2.159018	1.525613	1.1915749	1.6862799	0.9626397	1.150449	1.2541423	0.97454244	1.0254576	0.83104736	0.8335168	0.82550454	0.6257345	0.6256892	0.6652682	1.636697	1.263378	0.916141	0.685549	1.79	1.84	AT1G78490	cytochrome P450 family protein similar to Cytochrome P450 90A1 (SP:Q42569) [Arabidopsis thaliana]
255038_at	0.00219	1.0567027	1.5963777	1.0056602	1.1897914	1.5688801	1.1783156	1.2778376	1.7410942	0.69309795	0.9943397	0.7313463	0.83253795	0.6942606	0.80412805	0.78049856	0.8303529	1.21213	1.441532	0.81283	0.77731	1.49	1.85	AT4G09510	beta-fructofuranosidase, putative / invertase, putative / saccharase, putative / beta-fructosidase, putative similar to neutral invertase [Daucus carota] GI:4200165; contains Pfam profile PF04853: Plant neutral invertase
257375_at	0.00219	0.75275946	0.72312474	0.79953074	0.8021783	1.1207939	1.2319154	1.2433316	0.7475751	0.9718993	1.0281007	0.95136696	0.9430422	2.806335	2.3761797	1.6947606	2.4090152	0.769398	1.085904	0.973602	2.321578	0.79	0.47	AT2G38640	expressed protein contains Pfam profile PF04525: Protein of unknown function (DUF567)
253492_at	0.00219	1.0120381	1.3592821	1.1191956	1.227265	1.1252221	1.3519484	1.1939615	1.4737815	0.7155618	0.9879618	0.78447944	0.79155815	0.70447844	0.9686043	0.8782482	0.7638318	1.179445	1.286228	0.81989	0.828791	1.44	1.55	AT4G31810	enoyl-CoA hydratase/isomerase family protein similar to CHY1 [gi:8572760]; contains Pfam profile PF00388 enoyl-CoA hydratase/isomerase family protein
251787_at	0.00216	1.1584115	1.2582269	1.3296236	1.285931	1.2324315	1.1941451	1.2094346	2.3443034	0.82732517	0.78453636	0.8015394	0.84158856	0.621698	0.7270245	0.82385653	0.7879852	1.258048	1.495079	0.813742	0.740141	1.55	2.02	AT3G55410	2-oxoglutarate dehydrogenase E1 component, putative / oxoglutarate decarboxylase, putative / alpha-ketoglutaric dehydrogenase, putative similar to SP|P20967 2-oxoglutarate dehydrogenase E1 component, mitochondrial precursor (EC 1.2.4.2) (Alpha-ketoglutarate dehydrogenase) (Saccharomyces cerevisiae); contains Pfam profiles PF02779: Transketolase, pyridine binding domain, PF00676: Dehy[illegible]
252863_at	0.00216	0.4928098	0.6557904	0.36342606	0.3472017	0.66838384	0.8672156	0.40991876	1.0711837	1.070139	1.154942	1.0977025	0.92986095	1.4228488	1.5149895	1.4340782	1.5338453	0.464807	0.754175	1.063161	1.47644	0.44	0.51	AT4G39800	inositol-3-phosphate synthase isozyme 1 / myo-inositol-1-phosphate synthase 1 / MI-1-P synthase 1 / IPS 1 identical to SP|P42801 Inositol-3-phosphate synthase isozyme 1 (EC 5.5.1.4) (Myo-inositol-1- phosphate synthase 1) (MI-1-P synthase 1) (IPS 1) (Arabidopsis thaliana)
259209_at	0.00215	0.8920272	0.9046663	0.7535759	0.6727658	0.8329143	0.7138421	0.80917263	0.73821044	1.6291815	1.3486334	1.075609	1.1106876	1.6190456	1.2689408	1.2360294	1.0997971	0.805759	0.796404	1.291028	1.305953	0.62	0.61	AT3G09160	RNA recognition motif (RRM)-containing protein contains Pfam profile: PF00076 RNA recognition motif (aka RRM, RBD, or RNP domain)
262899_at	0.00215	0.97490937	1.0250907	1.3675007	1.1995627	1.5763413	1.1389362	1.256508	1.4302604	0.59542745	0.59150356	0.81878656	0.74172974	0.8321788	0.8233945	0.92674875	1.0740409	1.141766	1.350511	0.686862	0.914091	1.66	1.48	AT1G59870	ABC transporter family protein similar to PDR5-like ABC transporter GI:1514643 from [Spirodela polyrhiza]
266616_at	0.00214	0.90314627	1.0269556	0.75937265	0.76665723	0.751193	0.7664594	0.8303112	0.8239552	1.1437728	1.2164274	1.0227665	1.2398567	1.0597203	1.0178138	0.9821864	1.151081	0.864033	0.792966	1.155706	1.0527	0.75	0.75	AT2G29680	cell division control protein CDC6, putative almost identical to DNA replication protein CDC6 GI:18056480 from [Arabidopsis thaliana]; identical to cDNA CDC6 protein (2g29680 gene) GI:18056479
259749_at	0.00214	1.2871017	1.1578474	1.2411368	1.2097175	1.4241195	1.1518608	1.4006366	0.99044156	0.5070326	0.69117653	0.8158039	0.7665949	0.68818426	0.66059726	0.8101994	1.0095584	1.223951	1.241765	0.695152	0.792135	1.76	1.57	AT1G71100	ribose 5-phosphate isomerase-related similar to ribose-5-phosphate isomerase GI:18654317 from [Spinacia oleracea]
260048_at	0.0021	0.76407725	0.82716244	0.8077389	0.6260373	0.89765453	0.7337423	0.71872884	0.6989261	2.3233747	2.1029513	1.2871197	1.2363212	2.0556626	1.3438367	1.1023455	1.2422557	0.756254	0.762263	1.737442	1.43602	0.44	0.53	AT1G73750	expressed protein contains Pfam profile: PF00561 alpha/beta hydrolase fold
257974_at	0.00208	1.0296483	1.0311345	0.85597765	0.76848674	0.6557981	0.8498571	0.6581924	0.6805838	1.2435584	1.1985297	1.2770497	1.1763738	1.0714195	0.9703517	1.1328733	0.9278706	0.921312	0.711108	1.223878	1.025629	0.75	0.69	AT3G20820	leucine-rich repeat family protein contains similarity to Cf-2.1 [Lycopersicon pimpinellifolium] gi|1184075|gb|AAC15779; contains leucine rich-repeat domains Pfam:PF00560, INTERPRO:IPR001611
262538_at	0.00208	0.90500957	0.859373	0.65291715	0.69687116	0.70750403	0.7173496	0.62744117	0.9707672	1.0292327	1.4490657	1.1588066	1.3844422	1.1705456	1.22728	1.1944287	1.0556922	0.778543	0.755766	1.255387	1.161987	0.62	0.65	AT1G17140	tropomyosin-related similar to Tropomyosin 1. [Baker's yeast](SP:P17536) (Saccharomyces cerevisiae)
260686_at	0.00207	2.1890683	2.1463325	1.9098299	2.5232544	1.5922633	1.0478344	1.2165946	0.7754361	0.5233954	0.7833864	0.9521655	1.1288112	0.52336174	0.6045147	0.80298716	0.69941264	2.192121	1.158032	0.84694	0.657569	2.59	1.76	AT1G17620	expressed protein
260144_at	0.00206	1.0307279	1.335936	1.7627685	1.2098863	1.4421867	1.4503261	1.5613745	2.5408413	0.57396466	0.8807474	0.71118665	0.7848558	0.70701313	0.6976015	0.8962721	0.96927226	1.334855	1.748682	0.737689	0.81754	1.81	2.14	AT1G71960	ABC transporter family protein similar to breast cancer resistance protein GB:AAC97367 from [Homo sapiens]
253404_at	0.00205	1.0749158	1.2662722	1.0885218	1.4027354	1.1719811	1.4236792	1.0273919	1.2552938	0.7955089	0.8484603	0.8208825	0.93932813	0.97260827	0.8994212	0.889978	0.8693181	1.208111	1.219587	0.851045	0.907831	1.42	1.34	AT4G32840	phosphofructokinase family protein similar to phosphofructokinase [Amycolatopsis methanolica] GI:17432243; contains Pfam profile PF00365: Phosphofructokinase

[illegible]

257506_at	0.000629	0.894118	0.7614636	0.52816415	0.41236094	0.54479945	0.62115085	0.34172094	0.28946844	1.751357	1.4977993	2.0762684	1.6274261	1.2725922	1.9275107	2.143478	1.1058819	0.649027	0.449285	1.738213	1.612366	0.37	0.28	AT1G29440	auxin-responsive family protein similar to auxin-induced protein 6B (SP:P33083) [Glycine max]
256458_at	0.000629	1.260453	1.3518199	1.6561934	1.6712738	1.8463887	1.0876225	1.4226404	1.4486282	0.74824613	0.61270106	0.8721952	0.8410466	0.6441614	0.6788667	0.8141888	0.91237754	1.484935	1.45132	0.768547	0.762399	1.93	1.90	AT1G75220	integral membrane protein, putative strong similarity to integral membrane protein GI:1209756 from [Beta vulgaris]; contains Pfam profile PF00083: major facilitator superfamily protein
245743_at	0.000627	0.8417769	0.74395305	0.7867765	0.7819704	0.86890566	0.8365257	0.64716816	0.6476953	1.7242136	1.3517815	1.1652265	1.1514959	1.4100517	1.3377068	1.1310943	1.1324174	0.788619	0.750074	1.348179	1.252818	0.58	0.60	AT1G51080	expressed protein
252280_at	0.000627	0.99040544	0.87610096	0.78430456	0.8090209	0.83085006	0.7877015	0.73730457	0.85508	1.1622391	1.2598968	1.1244441	1.1126835	1.1967602	1.0095946	1.0113947	1.0856189	0.864958	0.802734	1.164815	1.075842	0.74	0.75	AT3G49260	calmodulin-binding family protein low similarity to SF16 protein [Helianthus annuus] GI:560150; contains Pfam profile PF00612: IQ calmodulin-binding motif
261149_s_at	0.000627	1.4802169	1.4438028	1.4595469	1.387479	1.7353495	1.2365754	1.6744049	1.0383415	0.8429767	0.8076331	0.8540461	0.7887248	0.9616584	0.76568997	0.7735515	0.9287081	1.442761	1.421168	0.823345	0.857402	1.75	1.66	AT1G19570	dehydroascorbate reductase, putative similar to GB:BAA90672 from (Oryza sativa)
265742_at	0.000627	0.7749434	0.7724782	0.8363795	0.63944143	0.91172904	0.8919078	0.86890846	0.7976707	1.3664489	1.3959374	1.1354569	1.088271	1.6380514	1.3074046	1.0897553	1.5094289	0.755811	0.867554	1.246529	1.38616	0.61	0.63	AT2G01290	expressed protein
247585_at	0.000627	0.7570694	0.64142877	0.53564	0.69416493	0.83878404	0.74985135	0.6333454	0.38165838	1.161216	1.7160898	1.4419031	1.9118035	1.6454121	1.5521319	1.2466944	1.4429966	0.657076	0.65091	1.557753	1.471809	0.42	0.44	AT5G60680	expressed protein contains Pfam profile PF04520: Protein of unknown function, DUF584
245451_at	0.000627	0.8613546	0.9192451	0.7664546	0.7836843	0.9061385	0.84366226	0.99029225	0.92215526	1.16134	1.1121811	1.2588986	1.134407	1.0097077	1.0268841	1.0856785	1.0446051	0.833125	0.915562	1.166707	1.041719	0.71	0.88	AT4G16890	disease resistance protein (TIR-NBS-LRR class), putative domain signature TIR-NBS-LRR exists, suggestive of a disease resistance protein.
265479_at	0.000627	1.0330356	0.96696424	1.1597718	1.7613683	1.4361918	2.139712	1.6820021	1.6781963	0.5917916	0.6280349	0.6531234	0.62652755	0.76671225	0.79865366	0.6418207	1.0384796	1.230285	1.734026	0.624869	0.811417	1.97	2.14	AT2G15760	calmodulin-binding protein similar to AR781 GI:1669593 from [Arabidopsis thaliana]; AR781 complements pheromone receptor deficient mutant of Shizosaccharomyces pombe
254389_s_at	0.000623	1.3832304	1.8675376	1.1185179	1.179673	2.3521216	1.3214359	1.5742114	2.7482412	0.6021015	0.64318395	0.7187704	0.58492255	0.8339949	0.7824253	0.69811004	0.88148224	1.38724	1.999003	0.637245	0.799003	2.18	2.50	AT4G21910	MATE efflux family protein similar to ripening regulated protein DDTFR18 [Lycopersicon esculentum] GI:12231296; contains Pfam profile PF01554: Uncharacterized membrane protein family
249790_at	0.000619	1.2345425	1.7507942	1.2763453	1.7736278	1.3917572	1.0859584	1.4435793	1.8082705	0.9140416	0.57534355	0.85547787	0.7046891	0.7376663	0.7030675	0.6920139	0.76061803	1.508827	1.432391	0.762388	0.723341	1.98	1.98	AT5G24290	integral membrane family protein contains Pfam domain PF01988: Integral membrane protein
249471_at	0.000619	1.3026619	1.2506527	1.7010884	1.6494387	1.4793427	1.2634344	1.4046892	1.2595608	0.728381	0.9311436	0.85204375	0.7183491	0.7948721	0.6711539	0.8078005	0.94043916	1.47596	1.301757	0.807479	0.803566	1.83	1.62	AT5G39360	circadian clock coupling factor-related similar to circadian clock coupling factor ZGT [Nicotiana tabacum] GI:14210079
252073_at	0.000605	0.80575293	1.0215331	0.66093	0.6503832	0.6175423	0.7657329	0.58837736	0.48077598	1.1402401	1.2741264	1.1569935	0.9784668	1.5096991	1.5190766	1.196701	1.4703302	0.78465	0.613107	1.137457	1.423952	0.69	0.43	AT3G51750	expressed protein
260833_at	0.000605	1.0120085	0.998157	1.1405346	1.1883373	1.4249288	1.1676687	1.4633288	1.5548916	0.82797486	0.70866185	0.7590327	0.6528164	0.7544079	0.9148756	0.93193287	1.001843	1.084759	1.402704	0.737121	0.900765	1.47	1.56	AT1G06800	lipase class 3 family protein similar to DEFECTIVE IN ANTHER DEHISCENCE1 [Arabidopsis thaliana] GI:16215706; contains Pfam profile PF01764: Lipase
248256_at	0.000604	1.393219	1.3894601	1.368193	1.3735198	1.3289193	1.1011261	1.3037022	1.0544738	0.6363158	0.83246565	0.9274049	0.9455261	0.74099326	0.6531051	0.83263314	0.86286366	1.381098	1.197055	0.835428	0.772399	1.65	1.55	AT5G53360	seven in absentia (SINA) family protein low similarity to siah-1A protein [Mus musculus] GI:297035; contains Pfam profile PF03145: Seven in absentia protein family
249242_at	0.000599	1.0437518	0.9562481	0.7882627	0.71543896	0.43504322	0.5674084	0.40423235	0.59268403	2.2045445	1.7967978	1.4889706	1.3022251	1.209862	1.137855	0.86573815	1.0809577	0.875925	0.499842	1.698135	1.073603	0.52	0.47	AT5G42250	alcohol dehydrogenase, putative similar to alcohol dehydrogenase ADH GI:7705214 from [Lycopersicon esculentum]; contains Pfam zinc-binding dehydrogenase domain PF00107
262661_s_at	0.000596	1.6355374	2.4353101	1.2964813	1.1624094	3.5593748	1.5118333	2.437532	2.798684	0.6219159	0.17509599	0.33330292	0.17969312	0.6359726	0.42876703	0.4898785	0.83759075	1.652435	2.576856	0.327502	0.598052	4.98	4.31	AT1G14250	nucleoside phosphatase family protein / GDA1/CD39 family protein low similarity to SP|P97687 Ectonucleoside triphosphate diphosphohydrolase 1 (EC 3.6.1.5) (Ecto-apyrase) (Rattus norvegicus); contains Pfam profile PF01150: GDA1/CD39 (nucleoside phosphatase) family
252265_at	0.000596	0.96400625	0.7411691	1.9806653	1.5139703	2.9049063	1.4922283	3.4531155	2.5751657	0.19331636	0.077841185	0.133463	0.23859341	1.372197	0.76128364	0.8066119	1.0359937	1.299953	2.606354	0.160803	0.994022	8.08	2.62	AT3G49620	2-oxoacid-dependent oxidase, putative (DIN11) identical to partial cds of 2-oxoacid-dependent oxidase (din11) from GI:10834554 [Arabidopsis thaliana]; identical to cDNA 2-oxoacid-dependent oxidase (din11) GI:10834553; contains Pfam profile PF03171: oxidoreductase, 2OG-Fe(II) oxygenase family
257653_at	0.000596	1.0376893	1.0240155	1.2432637	1.026504	1.3858911	1.2122995	1.480275	1.6067386	0.8323385	0.6850758	0.83557904	0.778555	0.8353233	0.7950649	0.9759844	0.9700456	1.082868	1.421326	0.782885	0.894105	1.38	1.59	AT3G13225	WW domain-containing protein contains Pfam profile PF00397: WW domain
254396_at	0.000593	1.8455285	1.8995304	3.3311367	1.3702042	1.49456	1.0632602	1.3725246	1.401569	0.3425749	0.34206724	0.5304753	0.93674	0.4075993	0.5131175	0.5483627	0.39190388	2.1116	1.332978	0.537964	0.465246	3.93	2.87	AT4G21680	proton-dependent oligopeptide transport (POT) family protein contains Pfam profile: PF00854 POT family
263174_at	0.000583	2.1953762	1.8388042	1.5350654	1.6010551	2.6077871	1.4410758	2.1007519	1.125392	0.874608	0.5831748	0.7041708	0.5382846	0.7815491	0.7284963	0.71179116	0.85213727	1.792575	1.818752	0.67506	0.768493	2.66	2.37	AT1G54040	kelch repeat-containing protein contains Pfam PF01344: Kelch motif (4 repeats); similar to jsimilar to epithiospecifier (GI:16118838) [Arabidopsis thaliana] isoform contains AT-AG splice sites at intron
251028_at	0.000581	0.87106985	0.8652655	0.50190413	0.5273861	0.5376999	0.56509966	0.49776098	0.4389238	2.431381	2.0795722	1.5544108	1.3775108	2.974486	1.9272493	1.1289301	1.471692	0.691406	0.509871	1.860719	1.875589	0.37	0.27	AT5G02230	haloacid dehalogenase-like hydrolase family protein contains InterPro accession IPR005834: Haloacid dehalogenase-like hydrolase
246932_at	0.000576	0.91448176	0.54683197	0.59357846	0.6146385	0.8073817	0.6487147	0.37057397	0.29155052	1.0855182	2.3013175	2.275229	3.1692991	2.106197	4.0141487	2.8785655	2.349846	0.667383	0.529555	2.207841	2.837189	0.30	0.19	AT5G25190	ethylene-responsive element-binding protein, putative ethylene responsive element binding protein homolog, Stylosanthes hamata, EMBL:U91857
266062_at	0.000576	1.2434239	1.2082669	1.2430212	1.3776778	1.1349176	1.1040845	1.1075794	1.5629321	0.78734475	0.7585177	0.83296824	0.87987226	0.70527434	0.8699252	0.8959156	0.8214557	1.268097	1.227378	0.814676	0.823143	1.56	1.49	AT2G18730	diacylglycerol kinase, putative contains INTERPRO domain, IPR001206, DAG-kinase catalytic domain
261661_at	0.000574	0.84657836	0.9072704	0.66783905	0.574365	0.73379153	0.6440829	0.65363574	0.8026578	1.4045298	1.3397082	1.0927296	1.1860362	1.3989012	1.1587365	1.0551607	1.2582273	0.749013	0.708542	1.255751	1.230256	0.60	0.58	AT1G18360	hydrolase, alpha/beta fold family protein similar to monoglyceride lipase from [Homo sapiens] GI:14594904, [Mus musculus] GI:2632162,[Rattus norvegicus] GI:19697886; contains Pfam profile PF00561: hydrolase, alpha/beta fold family
263429_at	0.000574	0.98323035	1.1126763	1.1316873	1.1021854	1.2825298	1.1753036	1.3274986	1.3824797	0.91207904	0.8710518	0.8520461	0.8100798	1.0167698	0.79952824	0.8930192	0.96444786	1.082445	1.291953	0.861314	0.918441	1.26	1.41	AT2G22250	aminotransferase class I and II family protein similar to aspartate aminotransferase from Bacillus stearothermophilus SP|Q59228, Thermus aquaticus SP|O33822; contains Pfam profile PF00155 aminotransferase, classes I and II
264609_at	0.000569	0.9033244	0.929561	0.7766647	0.7769028	0.9263069	0.94516903	0.7105841	0.7657638	1.2589818	1.222055	1.0548309	1.0984393	1.3595608	1.3312684	1.2031547	1.4385973	0.846613	0.836956	1.158577	1.333145	0.73	0.63	AT1G04530	expressed protein
251642_at	0.000562	1.4054675	1.4216849	1.879365	1.9454173	1.6562425	1.1865027	1.6020741	1.9503027	0.5063172	0.40754446	0.78724664	0.80799294	0.54122164	0.45311597	0.70605797	0.8134974	1.662984	1.598781	0.627275	0.628473	2.65	2.54	AT3G57520	alkaline alpha galactosidase, putative similar to alkaline alpha galactosidase II [Cucumis melo] GI:29838631; contains Pfam profile PF05691: Raffinose synthase or seed imbibition protein Sip1
248419_at	0.000562	1.3582659	1.6797539	1.5331374	1.7388064	1.3002023	1.0281247	1.0684206	1.32836	0.53466076	0.572589	0.7782162	0.9718752	0.5885622	0.4613055	0.77467936	0.5895298	1.577491	1.181277	0.714335	0.603419	2.21	1.96	AT5G51550	phosphate-responsive 1 family protein similar to phi-1 (phosphate-induced gene) [Nicotiana tabacum] GI:3759184; contains Pfam profile PF04674: Phosphate-induced protein 1 conserved region
255025_at	0.000562	0.9040271	0.9842863	0.72375953	0.7752553	0.6718941	0.7753787	0.80600256	0.7458157	1.4422032	1.2978448	1.1458297	1.1417722	1.2673589	1.1929108	1.0157138	1.1845332	0.846832	0.749773	1.256912	1.165129	0.67	0.64	AT4G09900	hydrolase, alpha/beta fold family protein similar to ethylene-induced esterase [Citrus sinensis] GI:14279437, polyneuridine aldehyde esterase [Rauwolfia serpentina] GI:6651393, SP|Q40708 PIR7A protein (Oryza sativa); contains Pfam profile PF00561: hydrolase, alpha/beta fold family
250960_at	0.000562	1.6002499	1.6239442	1.1787647	1.3528136	1.8614568	1.1619553	1.2625496	2.4634523	0.736859	0.4562273	0.589327	0.4348562	0.8360446	0.6369313	0.59102565	0.66888076	1.438943	1.687854	0.554317	0.683221	2.60	2.47	AT5G02940	expressed protein
264014_at	0.000556	0.7029125	0.7209062	0.4771251	0.5844371	0.5300073	0.58719546	0.45526654	0.35091025	2.977579	2.0058372	1.2790937	1.5332106	3.4883842	1.4679791	1.514546	1.4524153	0.621345	0.480845	1.94893	1.980831	0.32	0.24	AT2G21210	auxin-responsive protein, putative similar to small auxin-up regulated protein SAUR (GI:3043536) [Raphanus sativus]
255065_s_at	0.000551	1.660817	3.1588597	1.6557617	2.1334035	3.6324422	1.1666925	2.6389842	1.8961518	0.6431624	0.32766485	0.47775927	0.34286585	0.8315496	0.59477526	0.47304034	0.8333077	2.15221	2.333568	0.447863	0.683168	4.81	3.42	AT4G08870	arginase, putative similar to Swiss-Prot:P46637 arginase (EC 3.5.3.1) [Arabidopsis thaliana]
256229_at	0.000546	1.509993	1.0966688	1.6177896	1.3967974	1.5158699	1.2369161	1.4073335	2.1371884	0.8870908	0.7067393	0.810712	0.9033312	0.74364007	0.68187356	0.7043326	0.8491576	1.405312	1.574352	0.826968	0.744751	1.70	2.11	AT1G58200	mechanosensitive ion channel domain-containing protein / MS ion channel domain-containing protein contains Pfam profile PF00924: Mechanosensitive ion channel
[illegible]																									

Supplementary Table S8. Genes differentially expressed ≥ 2-fold in the *jaz7-1D* line relative to wild-type under MeJA treatment.

AGI	P-value	MeJA *jaz7-1D* / WT	MeJA inducibility WT MeJA / control	*jaz7-1D* MeJA / control	TAIR description
AT2G39030	4.37E-04	5.26	7.36	4.06	N-acetyltransferase (NATA1)
AT3G26830	1.47E-02	4.87	2.82	4.24	cytochrome P450 (CYP71B15)
AT2G24210	2.23E-02	3.9	1.56	6.17	myrcene/ocimene synthase (TPS10)
AT2G43590	1.07E-03	3.56	1.73	2.79	chitinase
AT2G35060	1.10E-02	3.23	0.61	0.87	potassium transporter
AT2G38290	1.83E-02	2.99	1.18	1.5	ammonium transporter 2 (AMT2)
AT3G09410	1.18E-02	2.78	1.61	2.1	pectinacetylesterase
AT2G46340	4.71E-03	2.77	0.42	0.68	phytochrome A supressor spa1 (SPA1)
AT2G38860	1.94E-03	2.69	2.15	1.64	proteaseI (pfpI)-like protein (YLS5)
AT4G39950	4.56E-03	2.66	2.13	2.72	cytochrome P450 (CYP79B2)
AT1G59950	2.13E-02	2.57	1.74	4.12	aldo/keto reductase
AT4G02410	1.09E-02	2.48	0.39	0.63	lectin protein kinase family protein
AT1G61120	7.78E-05	2.43	6.16	9.74	terpene synthase/cyclase family protein
AT3G18710	1.58E-02	2.39	0.42	0.45	U-box domain-containing protein
AT2G47680	2.10E-02	2.39	1.29	1.61	zinc finger (CCCH type) helicase
AT2G26440	1.02E-02	2.34	1.77	1.95	pectinesterase
AT2G46520	8.37E-03	2.34	1.39	1.82	cellular apoptosis susceptibility protein
AT5G55930	1.14E-02	2.3	1.42	2.24	oligopeptide transporter OPT family protein
AT2G46710	1.46E-03	2.29	0.66	0.76	rac GTPase activating protein
AT4G37370	1.95E-02	2.28	0.45	0.7	cytochrome P450
AT3G47420	2.11E-02	2.27	0.48	0.68	glycerol-3-phosphate transporter
AT2G39340	2.22E-02	2.27	1.23	1.7	SAC3/GANP family protein
AT2G35260	8.90E-03	2.26	0.65	0.75	expressed protein
AT2G47600	1.20E-04	2.24	2.55	2.89	magnesium/proton exchanger (MHX1)
AT2G43160	2.30E-02	2.24	1.22	1.68	epsin N-terminal homology (ENTH)
AT2G46530	2.09E-02	2.21	0.74	0.8	transcriptional factor B3 family protein
AT3G48850	3.30E-02	2.2	1.31	1.69	mitochondrial phosphate transporter
AT2G42690	3.40E-03	2.2	0.67	0.69	lipase
AT2G35040	4.98E-02	2.19	1.16	1.34	AICARFT/IMPCHase bienzyme
AT2G39710	3.06E-02	2.19	0.63	0.85	aspartyl protease
AT2G42490	2.94E-02	2.19	1.27	1.46	copper amine oxidase
AT5G24160	4.67E-02	2.18	1.08	1.5	squalene monooxygenase 1,2
AT1G19640	1.12E-04	2.18	1.94	3.56	S-adenosyl-L-methionine:jasmonic acid carboxyl methyltransferase (JMT)
AT2G35610	1.23E-02	2.16	1.25	1.45	expressed protein
AT2G41880	1.09E-02	2.14	1.27	1.46	guanylate kinase 1 (GK-1)
AT2G37690	2.59E-02	2.14	1.32	1.63	phosphoribosylaminoimidazole carboxylase
AT2G45560	6.09E-03	2.13	0.51	0.61	cytochrome P450 family protein
AT2G35230	4.43E-02	2.12	1.19	1.36	VQ motif-containing protein
AT5G65600	4.64E-02	2.12	1.3	1.7	legume lectin family protein
AT2G38050	1.53E-02	2.11	1.51	1.55	3-oxo-5-alpha-steroid 4-dehydrogenase
AT2G45290	1.27E-02	2.11	1.38	1.85	transketolase
AT2G47490	2.45E-03	2.09	0.84	0.74	mitochondrial substrate carrier family protein
AT2G46380	1.96E-02	2.09	0.73	0.82	hypothetical protein
AT2G47760	9.19E-03	2.08	1.18	1.27	ALG3 family protein
AT2G46020	4.54E-02	2.08	1.07	1.4	transcription regulatory protein SNF2
AT2G41540	1.06E-02	2.07	0.61	0.84	NAD-dependent glycerol-3-phosphate dehydrogenase
AT2G43500	3.99E-02	2.07	0.73	0.89	RWP-RK domain-containing protein low
AT2G39890	1.63E-02	2.06	0.71	0.88	proline transporter 1 (ProT1)
AT4G19170	7.66E-04	2.06	0.21	0.32	9-cis-epoxycarotenoid dioxygenase
AT2G40540	4.63E-02	2.04	0.68	0.84	potassium transporter (KT2)
AT2G36380	3.18E-04	2.03	2.23	3.31	ABC transporter family protein
AT1G21100	1.74E-02	2.01	1.98	1.36	O-methyltransferase
AT4G19430	3.33E-02	2.01	0.59	0.5	expressed protein
AT2G45590	2.01E-02	2.01	0.72	0.85	protein kinase
AT3G49620	5.96E-04	2	8.08	2.62	2-oxoacid-dependent oxidase (DIN11)
AT2G40090	8.39E-03	2	1.32	1.49	ABC1 family protein

Supplementary Table S9. Genes differentially expressed ≤ 2-fold in the *jaz7-1D* line relative to wild-type under MeJA treatment.

AGI	P-value	MeJA *jaz7-1D* / WT	MeJA inducibility WT MeJA / control	MeJA inducibility *jaz7-1D* MeJA / control	TAIR description
AT5G63450	7.19E-03	0.34	3.26	1.47	cytochrome P450
AT1G43160	1.56E-02	0.35	4.1	2.61	AP2 domain-containing protein (RAP2.6)
AT5G65080	4.03E-02	0.36	1.48	1.46	MADS-box family protein
AT1G05560	9.32E-03	0.37	0.45	0.43	UDP-glucose transferase (UGT75B2)
AT5G37770	4.01E-02	0.4	0.81	0.33	touch-responsive protein
AT3G28310	1.48E-02	0.41	0.57	0.49	expressed protein
AT4G38860	9.09E-03	0.43	0.42	0.32	auxin-responsive protein
AT2G21650	2.78E-03	0.44	0.67	0.39	myb family transcription factor
AT1G51090	3.61E-03	0.44	0.66	0.41	heavy-metal-associated domain-containing protein
AT3G45730	6.90E-03	0.45	0.66	0.45	expressed protein
AT3G48100	1.77E-02	0.45	0.47	0.32	response regulator 5 (ARR5)
AT4G34135	1.11E-02	0.46	0.33	0.63	UDP-glucoronosyl/UDP-glucosyl transferase
AT5G16970	1.83E-02	0.46	0.63	0.54	NADP-dependent oxidoreductase
AT3G06070	4.17E-02	0.46	0.78	0.43	expressed protein
AT5G62360	4.89E-05	0.47	2.67	2.92	invertase/pectin methylesterase inhibitor family protein
AT1G34760	1.82E-02	0.48	1.43	1.38	14-3-3 protein GF14 omicron (GRF11)
AT1G48330	1.46E-02	0.49	0.53	0.37	expressed protein
AT2G29490	3.19E-02	0.49	0.37	0.66	glutathione S-transferase
AT1G29510	1.73E-02	0.5	0.62	0.34	auxin-responsive protein
AT5G16990	1.91E-02	0.5	0.5	0.61	NADP-dependent oxidoreductase
AT5G24580	1.10E-03	0.5	0.57	0.48	copper-binding family protein

Supplementary Table S10. Differentially regulated by MeJA treatment genes sorted by MeJA inducibility in wild-type plants.

Table S7 data sorted by highest wild-type MeJA/control values.

AGI	P-value	Control levels WT	Control levels *jaz7-1D*	MeJA levels WT	MeJA levels *jaz7-1D*	MeJA inducibility (MeJA/control) WT	MeJA inducibility (MeJA/control) *jaz7-1D*	Difference in MeJA inducibility WT (MeJA/control) / *jaz7-1D* (MeJA/control)	TAIR description
AT5G44420	3.26E-05	0.13	0.49	2.91	1.99	22.47	4.06	0.18	plant defensin protein, putative (PDF1.2a) plant defensin protein family member, personal communication, Bart Thomma (Bart.Thomma@agr.kuleuven.ac.be); similar to antifungal protein 1 preprotein [Raphanus sativus] gi|609322|gb|AAA69541
AT4G17470	1.77E-04	0.25	0.23	2.89	2.98	11.56	12.76	1.10	palmitoyl protein thioesterase family protein
AT2G26020	6.92E-05	0.29	0.64	3.15	1.70	10.74	2.67	0.25	plant defensin-fusion protein, putative (PDF1.2b) plant defensin protein family member, personal communication, Bart Thomma (Bart.Thomma@agr.kuleuven.ac.be); similar to antifungal protein 1 preprotein [Raphanus sativus] gi|609322|gb|AAA69541
AT4G23600	2.56E-05	0.23	0.41	1.91	2.34	8.22	5.66	0.69	coronatine-responsive tyrosine aminotransferase / tyrosine transaminase similar to nicotianamine aminotransferase from Hordeum vulgare [GI:6498122, GI:6469087]; contains Pfam profile PF00155 aminotransferase, classes I and II; identical to cDNA coronatine-regulated tyrosine aminotransferase (F9D16.70) GI:15076852
AT3G49620	5.96E-04	0.16	0.99	1.30	2.61	8.08	2.62	0.32	2-oxoacid-dependent oxidase, putative (DIN11) identical to partial cds of 2-oxoacid-dependent oxidase (din11) from GI:10834554 [Arabidopsis thaliana]; identical to cDNA 2-oxoacid-dependent oxidase (din11) GI:10834553; contains Pfam profile PF03171: oxidoreductase, 2OG-Fe(II) oxygenase family
AT2G39030	4.37E-04	0.12	1.17	0.90	4.75	7.36	4.06	0.55	GCN5-related N-acetyltransferase (GNAT) family protein similar to SP|Q9SM88 Tyramine N-feruloyltransferase 4/11 (EC 2.3.1.110) (Hydroxycinnamoyl- CoA: tyramine N-hydroxycinnamoyltransferase) (Nicotiana tabacum); contains Pfam profile PF00583: acetyltransferase, GNAT family
AT4G18440	1.41E-04	0.37	0.37	2.67	3.46	7.24	9.25	1.28	adenylosuccinate lyase, putative / adenylosuccinase, putative similar to SP|P25739 Adenylosuccinate lyase (EC 4.3.2.2) (Adenylosuccinase) (Escherichia coli); contains Pfam profile PF00206: Lyase
AT3G45140	2.52E-05	0.24	0.39	1.71	1.91	7.15	4.87	0.68	lipoxygenase (LOX2) identical to SP|P38418
AT5G61160	1.48E-03	0.26	0.94	1.82	2.79	7.02	2.97	0.42	transferase family protein similar to anthocyanin 5-aromatic acyltransferase from Gentiana triflora GI:4185599, malonyl CoA:anthocyanin 5-O-glucoside-6'''-O-malonyltransferase from Perilla frutescens GI:17980232, Salvia splendens GI:17980234; contains Pfam profile PF02458 transferase family
AT1G19670	2.52E-05	0.25	0.50	1.65	1.86	6.53	3.75	0.57	coronatine-responsive protein / coronatine-induced protein 1 (CORI1) identical to coronatine-induced protein 1 (CORI1) GI:30912637 from [Arabidopsis thaliana]
AT4G11310	1.46E-03	0.39	0.58	2.48	4.16	6.41	7.15	1.12	cysteine proteinase, putative contains similarity to cysteine proteinase RD21A (thiol protease) GI:435619, SP:P43297 from [Arabidopsis thaliana]
AT4G16260	2.06E-04	0.28	0.73	1.74	2.16	6.29	2.96	0.47	glycosyl hydrolase family 17 protein similar to glucan endo-1,3-beta-glucosidase, basic vacuolar isoform precursor SP:P52407 from [Hevea brasiliensis]
AT4G24350	5.11E-06	0.27	0.41	1.70	1.94	6.25	4.71	0.75	[AT4G24350, phosphorylase family protein contains Pfam PF01048: Phosphorylase family];[AT4G24340, phosphorylase family protein contains Pfam PF01048: Phosphorylase family]
AT1G54020	1.32E-03	0.26	0.74	1.63	1.94	6.23	2.63	0.42	myrosinase-associated protein, putative strong similarity to myrosinase-associated proteins GI:1769968, GI:1769970, GI:1216389,GI:1216391 from [Brassica napus]; contains InterPro Entry IPR001087 Lipolytic enzyme, G-D-S-L family
AT1G61120	7.78E-05	0.32	0.49	1.98	4.81	6.16	9.74	1.58	terpene synthase/cyclase family protein similar to S-linalool synthase GI:1491939 from [Clarkia breweri][PMID: 8768373]
AT4G24340	2.52E-05	0.37	0.53	2.21	1.97	6.04	3.71	0.61	phosphorylase family protein contains Pfam PF01048: Phosphorylase family
AT3G23550	1.15E-04	0.24	0.95	1.34	2.07	5.68	2.19	0.39	[AT3G23550, MATE efflux family protein similar to ripening regulated protein DDTFR18 [Lycopersicon esculentum] GI:12231296; contains Pfam profile: PF01554 uncharacterized membrane protein family];[AT3G23560, MATE efflux family protein similar to ripening regulated protein DDTFR18 [Lycopersicon esculentum] GI:12231296; contains Pfam profile: [illegible]
AT5G38710	2.13E-04	0.52	0.51	2.79	2.93	5.39	5.72	1.06	proline oxidase, putative / osmotic stress-responsive proline dehydrogenase, putative similar to proline oxidase, mitochondrial precursor (Osmotic stress- induced proline dehydrogenase) [Arabidopsis thaliana] SWISS-PROT:P92983
AT1G30135	6.17E-03	0.55	0.51	2.92	2.47	5.33	4.82	0.90	expressed protein
AT3G09940	7.28E-05	0.32	0.62	1.62	1.52	5.14	2.46	0.48	monodehydroascorbate reductase, putative similar to monodehydroascorbate reductase (NADH) GB:JU0182 (Cucumis sativus)
AT4G37410	9.52E-04	0.38	0.81	1.91	1.67	5.06	2.06	0.41	cytochrome P450, putative similar to cytochrome p450 SP:O65790 from [Arabidopsis thaliana]
AT1G14250	5.96E-04	0.33	0.60	1.63	2.58	4.98	4.31	0.86	nucleoside phosphatase family protein / GDA1/CD39 family protein low similarity to SP|P97687 Ectonucleoside triphosphate diphosphohydrolase 1 (EC 3.6.1.5) (Ecto-apyrase) [Rattus norvegicus]; contains Pfam profile PF01150: GDA1/CD39 (nucleoside phosphatase) family
AT2G24850	2.32E-03	0.30	0.66	1.51	2.59	4.98	3.92	0.79	aminotransferase, putative similar to nicotianamine aminotransferase from Hordeum vulgare [GI:6498122, GI:6469087]; contains Pfam profile PF00155 aminotransferase, classes I and II
AT5G57550	1.41E-04	0.62	0.33	3.07	1.88	4.93	5.74	1.16	xyloglucan:xyloglucosyl transferase / xyloglucan endotransglycosylase / endo-xyloglucan transferase (XTR3) identical to endoxyloglucan transferase GI:5533317 from [Arabidopsis thaliana]
AT4G08870	5.51E-04	0.45	0.68	2.15	2.33	4.81	3.42	0.71	arginase, putative similar to Swiss-Prot:P46637 arginase (EC 3.5.3.1) [Arabidopsis thaliana]
AT3G44860	7.70E-04	0.39	0.50	1.85	1.70	4.79	3.40	0.71	S-adenosyl-L-methionine:carboxyl methyltransferase family protein similar to defense-related protein cjs1 [Brassica carinata][GI:14009292][Mol Plant Pathol (2001) 2(3):159-169]
AT4G31800	2.01E-03	0.37	0.46	1.79	1.52	4.77	3.34	0.70	WRKY family transcription factor
AT4G21840	2.30E-03	0.44	0.86	2.01	1.53	4.56	1.78	0.39	[AT4G21840, methionine sulfoxide reductase domain-containing protein / SeIR domain-containing protein weak similarity to pilin-like transcription factor [Homo sapiens] GI:5059062, SP|P14930 Peptide methionine sulfoxide reductase msrA/msrB (EC 1.8.4.6) [Neisseria gonorrhoeae]; contains Pfam profile PF01641: SelR domain];[AT4G21830, methionine s[illegible]
AT4G02360	8.43E-03	0.58	0.85	2.63	4.08	4.56	4.83	1.06	expressed protein contains Pfam profile PF04398: Protein of unknown function, DUF538
AT1G78370	2.03E-03	0.41	0.69	1.83	1.75	4.48	2.56	0.57	glutathione S-transferase, putative similar to 2,4-D inducible glutathione S-transferase GI:2920666 from [Glycine max]
AT4G28085	5.37E-03	0.58	0.84	2.51	2.36	4.31	2.79	0.65	expressed protein
AT2G39420	1.05E-04	0.26	0.80	1.12	2.12	4.23	2.66	0.63	esterase/lipase/thioesterase family protein similar to monoglyceride lipase from [Homo sapiens] GI:14594904, [Mus musculus] GI:2632162; contains Interpro entry IPR000379
AT2G39330	4.69E-03	0.40	0.83	1.69	1.92	4.19	2.30	0.55	jacalin lectin family protein similar to myrosinase-binding protein homolog [Arabidopsis thaliana] GI:2997767, myrosinase binding protein [Brassica napus] GI:1711296; contains Pfam profile PF01419 jacalin-like lectin domain
AT1G52410	9.10E-05	0.39	0.62	1.62	2.14	4.13	3.43	0.83	caldesmon-related weak similarity to Caldesmon (CDM) (Swiss-Prot:P12957) [Gallus gallus]
AT1G43160	1.56E-02	0.89	0.49	3.65	1.28	4.10	2.61	0.64	AP2 domain-containing protein RAP2.6 (RAP2.6) identical to AP2 domain containing protein RAP2.6 GI:2281637 from [Arabidopsis thaliana]
AT2G46650	4.34E-04	0.30	0.81	1.25	2.36	4.10	2.94	0.72	cytochrome b5, putative similar to cytochrome b5 GI:2695711 from [Olea europaea]
AT5G12340	6.34E-03	0.60	0.37	2.44	1.96	4.05	5.25	1.29	expressed protein ; expression supported by MPSS
AT5G44050	2.17E-04	0.49	0.66	1.98	2.00	4.05	3.05	0.75	MATE efflux family protein similar to ripening regulated protein DDTFR18 [Lycopersicon esculentum] GI:12231296; contains Pfam profile PF01554: Uncharacterized membrane protein family
AT2G38750	7.23E-05	0.43	0.54	1.75	1.98	4.02	3.68	0.92	annexin 4 (ANN4) nearly identical to annexin (AnnAt4) [Arabidopsis thaliana] GI:6503084; contains Pfam profile PF00191: Annexin
AT3G51450	1.12E-04	0.43	0.48	1.71	1.82	3.96	3.82	0.97	strictosidine synthase family protein similar to hemomucin [Drosophila melanogaster][GI:1280434], strictosidine synthase [Rauvolfia serpentina][SP|P15324]
AT4G21680	5.93E-04	0.54	0.47	2.11	1.33	3.93	2.87	0.73	proton-dependent oligopeptide transport (POT) family protein contains Pfam profile: PF00854 POT family
AT1G16410;AT1G	2.69E-03	0.59	0.53	2.28	2.09	3.86	3.98	1.03	[AT1G16410, cytochrome P450, putative similar to gb|AF069494 cytochrome P450 from Sinapis alba and is a member of the PF|00067 Cytochrome P450 family];[AT1G16400, cytochrome P450 family protein similar to gb|AF069494 cytochrome P450 from Sinapis alba and is a member of the PF|00067 Cytochrome P450 family; identical to cytochrome P450 [illegible]
AT2G19800	1.18E-02	0.63	0.53	2.42	1.39	3.84	2.60	0.68	expressed protein similar to myo-inositol oxygenase [Sus scrofa] gi|17432544|gb|AAL39076
AT2G29460	2.49E-03	0.55	0.69	2.06	1.63	3.77	2.38	0.63	glutathione S-transferase, putative
AT2G34810	3.41E-04	0.37	0.76	1.40	2.21	3.75	2.91	0.78	FAD-binding domain-containing protein similar to SP|P30986 reticuline oxidase precursor (Berberine-bridge-forming enzyme) (BBE) (Tetrahydroprotoberberine synthase) [Eschscholzia californica]; contains PF01565 FAD binding domain
AT5G05600	2.52E-05	0.47	0.49	1.73	1.66	3.72	3.38	0.91	oxidoreductase, 2OG-Fe(II) oxygenase family protein similar to flavonol synthase [Citrus unshiu][gi:4126403], leucoanthocyanidin dioxygenase [Daucus carota][gi:5924383]; contains PF03171 2OG-Fe(II) oxygenase superfamily domain
AT5G10300	4.35E-04	0.70	0.67	2.61	1.83	3.72	2.74	0.74	hydrolase, alpha/beta fold family protein similar to ethylene-induced esterase [Citrus sinensis] GI:14279437, alpha-hydroxynitrile lyase [Manihot esculenta] GI:2780225; contains Pfam profile PF00561: hydrolase, alpha/beta fold family
AT5G24420	1.56E-03	0.38	0.90	1.40	2.02	3.72	2.26	0.61	glucosamine/galactosamine-6-phosphate isomerase-related contains weak similarity to Swiss-Prot:O95336 6-phosphogluconolactonase (EC 3.1.1.31) (6PGL) [Homo sapiens]
AT3G22740	3.36E-04	0.58	0.58	2.15	1.89	3.70	3.26	0.88	homocysteine S-methyltransferase 3 (HMT-3) identical to homocysteine S-methyltransferase HMT-3 [Arabidopsis thaliana] GI:9966515; similar to homocysteine S-methyltransferase AtHMT-2 (GI:6685163) [Arabidopsis thaliana]; similar to selenocysteine methyltransferase GB:P56707 from [Astragalus bisulcatus]
AT1G54010;AT1G	1.78E-03	0.65	0.61	2.39	2.29	3.67	3.77	1.03	[AT1G54010, myrosinase-associated protein, putative similar to myrosinase-associated protein GI:1769969 from [Brassica napus]; contains Pfam profile PF00657: Lipase/Acylhydrolase with GDSL-like motif];[AT1G54000, myrosinase-associated protein, putative similar to myrosinase-associated proteins GI:1769968, [illegible]
AT3G19710	1.49E-03	0.43	0.56	1.58	1.85	3.66	3.31	0.90	branched-chain amino acid aminotransferase, putative / branched-chain amino acid transaminase, putative (BCAT4) similar to branched-chain amino acid transaminase 6 [Arabidopsis thaliana] GI:13810195; contains Pfam profile: PF01063 aminotransferase class IV
AT5G19110	1.38E-03	0.73	0.67	2.64	1.52	3.64	2.28	0.63	extracellular dermal glycoprotein-related / EDGP-related similar to extracellular dermal glycoprotein EDGP precursor [Daucus carota] GI:285741
AT1G11580	2.82E-04	0.49	0.69	1.76	2.29	3.63	3.31	0.91	pectin methylesterase, putative similar to pectin methylesterase GI:1617583 from [Lycopersicon esculentum]
AT1G17745	6.68E-04	0.41	0.73	1.50	2.46	3.62	3.38	0.93	D-3-phosphoglycerate dehydrogenase / 3-PGDH identical to SP|O04130
AT3G56200	8.58E-05	0.50	0.60	1.79	2.02	3.58	3.38	0.94	amino acid transporter family protein low similarity to N system amino acids transporter NAT-1 [Mus musculus] GI:7406950; belongs to INTERPRO:IPR002422 amino acid/polyamine transporter, family II
AT3G16460	1.77E-04	0.68	0.47	2.41	1.83	3.53	3.93	1.12	jacalin lectin family protein contains Pfam profile: PF01419 jacalin-like lectin domain; similar to myrosinase binding protein [Brassica napus] GI:1711296, GI:1655824, myrosinase-binding protein homolog [Arabidopsis thaliana] GI:2997767; contains Pfam profile PF01419 jacalin-like lectin family
AT2G38760	1.61E-04	0.45	0.72	1.58	2.38	3.52	3.31	0.94	annexin 3 (ANN3) nearly identical to annexin (AnnAt3) [Arabidopsis thaliana] GI:6503082; contains Pfam profile PF00191: Annexin
AT1G64660	8.42E-04	0.78	0.61	2.69	2.42	3.43	3.99	1.16	Cys/Met metabolism pyridoxal-phosphate-dependent enzyme family protein similar to SP|P13254 Methionine gamma-lyase (EC 4.4.1.11) (L-methioninase) [Pseudomonas putida]; contains Pfam profile PF01053: Cys/Met metabolism PLP-dependent enzyme
AT3G28220	7.27E-03	0.59	0.67	2.02	1.60	3.43	2.37	0.69	meprin and TRAF homology domain-containing protein / MATH domain-containing protein similar to ubiquitin-specific protease 12 [Arabidopsis thaliana] GI:11993471; contains Pfam profile PF00917: MATH domain
AT2G43100	1.78E-03	0.47	0.64	1.63	2.39	3.43	3.72	1.09	aconitase C-terminal domain-containing protein contains Pfam profile PF00694: Aconitase C-terminal domain
AT5G24780	1.08E-02	0.44	0.72	1.49	1.41	3.38	1.94	0.58	vegetative storage protein 1 (VSP1) identical to SP|O49195 Vegetative storage protein 1 precursor [Arabidopsis thaliana]; contains Pfam profile PF03767: HAD superfamily (subfamily IIIB) phosphatase
AT1G06620	4.19E-04	0.67	0.59	2.25	1.60	3.37	2.71	0.80	2-oxoglutarate-dependent dioxygenase, putative similar to 2A6 (GI:599622) and tomato ethylene synthesis regulatory protein E8 (SP|P10967); contains Pfam profile: PF00671 Iron/Ascorbate oxidoreductase family
AT5G42650	4.89E-05	0.57	0.56	1.91	1.94	3.34	3.46	1.04	allene oxide synthase (AOS) / hydroperoxide dehydrase / cytochrome P450 74A (CYP74A) identical to Allene oxide synthase, chloroplast precursor (Hydroperoxide dehydrase) (Cytochrome P450 74A) (SP:Q96242) {Arabidopsis thaliana}
AT5G07460	1.05E-03	0.50	0.68	1.66	1.81	3.34	2.64	0.79	peptide methionine sulfoxide reductase, putative similar to peptide methionine sulfoxide reductase (msr) [Arabidopsis thaliana] GI:4884033; contains Pfam profile PF01625: Peptide methionine sulfoxide reductase
AT3G02140	7.28E-05	0.75	0.66	2.49	1.46	3.33	2.20	0.66	expressed protein
AT1G52890	2.80E-03	0.59	0.40	1.93	1.42	3.29	3.53	1.07	no apical meristem (NAM) family protein contains Pfam PF02365: No apical meristem (NAM) domain; similar to NAM (no apical meristem) GB:CAA63101 from [Petunia x hybrida]
AT1G52030;AT1G	3.51E-04	0.44	0.86	1.43	2.01	3.28	2.32	0.71	[AT1G52030, myrosinase-binding protein, putative (F-ATMBP) identical to SP|Q9SAV1 Myrosinase binding protein-like f-AtMBP [Arabidopsis thaliana]; similar to myrosinase binding protein GI:1711295 from [Brassica napus]; contains Pfam profile PF01419: Jacalin-like lectin domain; identical to cDNA myrosinase-binding pro[illegible]
AT1G65860	5.04E-03	0.48	0.61	1.58	1.77	3.27	2.91	0.89	flavin-containing monooxygenase family protein / FMO family protein similar to flavin-containing monooxygenase FMO3 (dimethylaniline monoxygenase [N-oxide forming] 3) GI:349533 [SP|P32417] from Oryctolagus cuniculus, [SP|P97501] from Mus musculus; contains Pfam profile PF00743 Flavin-binding monooxy[illegible]
AT5G63450	7.19E-03	0.92	0.69	3.01	1.01	3.26	1.47	0.45	cytochrome P450, putative
AT4G12030	9.21E-04	0.48	0.53	1.56	2.01	3.26	3.78	1.16	bile acid:sodium symporter family protein low similarity to SP|Q12908 Ileal sodium/bile acid cotransporter (Homo sapiens); contains Pfam profile PF01758: Sodium Bile acid symporter family
AT4G15210	3.76E-02	0.54	0.70	1.76	1.40	3.23	1.98	0.61	beta-amylase (BMY1) / 1,4-alpha-D-glucan maltohydrolase identical to Beta-amylase (EC 3.2.1.2) (1,4-alpha-D-glucan maltohydrolase) SP:P25853 [Arabidopsis thaliana]
AT4G15440	2.49E-03	0.47	0.83	1.51	1.47	3.22	1.78	0.55	hydroperoxide lyase (HPL1) identical to hydroperoxide lyase GI:3822403 from [Arabidopsis thaliana]
AT5G07010	2.21E-03	0.67	0.57	2.13	2.45	3.17	4.33	1.37	sulfotransferase family protein similar to steroid sulfotransferase 3 [Brassica napus] GI:3420008, steroid sulfotransferase 1 [Brassica napus] GI:3420004; contains Pfam profile PF00685: Sulfotransferase domain
AT1G75960	1.52E-04	0.64	0.48	2.01	1.98	3.14	4.15	1.32	AMP-binding protein, putative similar to AMP-binding protein GI:1903034 from [Brassica napus]; contains Pfam profile: PF00501 AMP-binding enzyme; identical to cDNA adenosine monophosphate binding protein 8 AMPBP8 (AMPBP8) GI:20799724
AT2G41180	4.37E-04	0.38	0.94	1.20	1.56	3.14	1.66	0.53	sigA-binding protein-related low similarity to SigA binding protein [Arabidopsis thaliana] GI:6980074; contains Pfam domain PF05678: VQ motif
AT2G23560	6.48E-04	0.70	0.74	2.17	3.03	3.12	4.10	1.31	hydrolase, alpha/beta fold family protein similar to ethylene-induced esterase [Citrus sinensis] GI:14279437, polyneuridine aldehyde esterase [Rauvolfia serpentina] GI:6651393; contains Pfam profile PF00561: hydrolase, alpha/beta fold family
AT3G58990	1.75E-03	0.56	0.50	1.76	1.96	3.12	3.89	1.25	aconitase C-terminal domain-containing protein contains Pfam profile PF00694: Aconitase C-terminal domain
AT5G27520	9.74E-04	0.60	0.58	1.87	1.66	3.11	2.87	0.92	mitochondrial substrate carrier family protein
AT5G36220	8.58E-05	0.60	0.71	1.84	1.72	3.09	2.43	0.79	cytochrome P450 81D1 (CYP81D1) (CYP91A1) identical to Cytochrome P450 (SP:Q9FG65) [Arabidopsis thaliana];
AT3G28930	3.55E-03	0.44	0.94	1.34	1.70	3.07	1.81	0.59	avrRpt2-induced AIG2 protein (AIG2) identical to SP|P54121 AIG2 protein (avrRpt2-induced gene) (Arabidopsis thaliana)
AT1G72790	1.39E-04	0.61	0.55	1.86	1.45	3.04	2.63	0.86	hydroxyproline-rich glycoprotein family protein contains proline-rich extensin domains, INTERPRO:IPR002965
AT1G51780	7.78E-05	0.57	0.57	1.73	1.81	3.03	3.17	1.05	IAA-amino acid hydrolase 5 / auxin conjugate hydrolase (ILL5) identical to auxin conjugate hydrolase ILL5 [Arabidopsis thaliana] gi|5725649|gb|AAD48152; contains nonconsensus AT acceptor splice site at exon3
AT1G76790	8.07E-04	0.76	0.54	2.27	1.46	3.01	2.67	0.89	O-methyltransferase family 2 protein similar to caffeic acid O-methyltransferase [Catharanthus roseus][GI:18025321], catechol O-methyltransferase GB:CAA55358 [Vanilla planifolia]
AT5G23820	1.98E-03	0.76	0.80	2.27	2.07	3.00	2.60	0.87	MD-2-related lipid recognition domain-containing protein / ML domain-containing protein contains Pfam profile PF02221: ML domain
AT5G23010	2.82E-04	0.54	0.46	1.62	1.64	2.98	3.59	1.20	2-isopropylmalate synthase 3 (IMS3) identical to 2-isopropylmalate synthase (IMS3) [Arabidopsis thaliana] GI:15983745; identical to cDNA 2-isopropylmalate synthase (IMS3) GI:15983744
AT1G70700	1.68E-04	0.56	0.56	1.68	1.50	2.98	2.70	0.91	expressed protein
AT4G21850	3.41E-04	0.76	0.81	2.24	2.01	2.96	2.47	0.83	methionine sulfoxide reductase domain-containing protein / SeIR domain-containing protein low similarity to pilin-like transcription factor [Homo sapiens] GI:5059062, SP|P14930 Peptide methionine sulfoxide reductase msrA/msrB (EC 1.8.4.6) (Neisseria gonorrhoeae); contains Pfam profile PF01641: SelR domain
AT1G80160	1.80E-02	0.95	0.66	2.81	1.80	2.95	2.71	0.92	lactoylglutathione lyase family protein / glyoxalase I family protein contains glyoxalase family protein domain, Pfam:PF00903
AT2G27690	2.49E-03	0.69	0.35	2.02	1.27	2.94	3.65	1.24	cytochrome P450, putative similar to Cytochrome P450 94A1 (P450-dependent fatty acid omega-hydroxylase) (SP:O81117) (Vicia sativa); contains Pfam profile: PF00067 cytochrome P450; supported by cDNA: gi_13877668
AT1G64200	1.80E-03	0.80	0.79	2.34	1.92	2.93	2.43	0.83	vacuolar ATP synthase subunit E, putative / V-ATPase E subunit, putative / vacuolar proton pump E subunit, putative similar to SP|Q39258 Vacuolar ATP synthase subunit E (EC 3.6.3.14) (V-ATPase E subunit) (Vacuolar proton pump E subunit) [Arabidopsis thaliana]; contains Pfam profile PF01991: ATP synthase (E/31 k[illegible]
AT1G16370	8.01E-04	0.75	0.67	2.18	1.59	2.90	2.37	0.82	transporter-related low similarity to organic cation transporter OCTN1 from [Homo sapiens] GI:2605501, [Mus musculus] GI:4126605, [Rattus norvegicus] GI:5679326; contains Pfam profile PF00083: major facilitator superfamily protein
AT4G39940	1.10E-03	0.43	0.80	1.23	1.83	2.88	2.28	0.79	adenylylsulfate kinase 2 (AKN2) identical to adenylylsulfate kinase 2, chloroplast precursor (APS kinase, Adenosine-5'phosphosulfate kinase, ATP adenosine-5'- phosphosulfate 3'-phosphotransferase) [Arabidopsis thaliana] SWISS-PROT:O49196
AT1G22400	2.93E-04	0.66	0.50	1.87	1.68	2.85	3.36	1.18	UDP-glucoronosyl/UDP-glucosyl transferase family protein contains Pfam profile: PF00201 UDP-glucoronosyl and UDP-glucosyl transferase
AT2G43510	2.53E-02	0.49	0.94	1.40	1.79	2.85	1.91	0.67	trypsin inhibitor, putative similar to SP|P26780 Trypsin inhibitor 2 precursor (MTI-2) (Sinapis alba)
AT1G62660	2.93E-05	0.54	0.54	1.54	1.90	2.83	3.54	1.25	beta-fructosidase (BFRUCT3) / beta-fructofuranosidase / invertase, vacuolar identical to beta-fructosidase GB:CAA67560 GI:1429209 [Arabidopsis thaliana]; supported by full-length cDNA GI:14517549; identical to cDNA Beta-fructosidase GI:3115854
AT2G43550	2.23E-03	0.49	0.82	1.39	1.68	2.82	2.05	0.73	trypsin inhibitor, putative similar to SP|P26780 Trypsin inhibitor 2 precursor (MTI-2) (Sinapis alba)
AT3G26830	1.47E-02	0.35	1.14	0.99	4.83	2.82	4.24	1.50	cytochrome P450 71B15, putative (CYP71B15) identical to Cytochrome P450 (SP:Q9LW27) [Arabidopsis thaliana]; similar to cytochrome P450 71B2 GB:O65788 [Arabidopsis thaliana]
AT5G44720	1.39E-04	0.55	0.75	1.54	1.42	2.79	1.88	0.67	molybdenum cofactor sulfurase family protein weak similarity to molybdenum cofactor sulfurase (LOS5/ABA3) [Arabidopsis thaliana] GI:15407262; contains Pfam profiles PF03476: MOSC N-terminal beta barrel domain, PF03473: MOSC domain
AT1G17380	2.94E-02	0.55	0.60	1.53	1.36	2.76	2.25	0.82	expressed protein
AT3G55970	1.40E-02	0.75	0.57	2.05	1.47	2.74	2.56	0.93	oxidoreductase, 2OG-Fe(II) oxygenase family protein similar to leucoanthocyanidin dioxygenase, Malus domestica, SP|P51091; contains Pfam profile PF03171: oxidoreductase, 2OG-Fe(II) oxygenase family
AT3G01970	4.92E-03	0.63	0.89	1.73	1.49	2.74	1.67	0.61	WRKY family transcription factor similar to WRKY1 GB:AAC49527 [Petroselinum crispum]
AT2G29470	4.12E-03	0.79	0.70	2.15	1.72	2.73	2.45	0.90	glutathione S-transferase, putative similar to glutathione S-transferase [Euphorbia esula] gb:AAF64450.1 GI:7595790
AT1G52720	1.22E-03	0.86	0.80	2.34	2.17	2.72	2.70	0.99	expressed protein
AT1G23850	1.27E-04	0.56	0.64	1.50	1.67	2.70	2.61	0.97	expressed protein
AT4G33150	1.19E-03	0.76	0.64	2.05	1.70	2.69	2.66	0.99	lysine-ketoglutarate reductase/saccharopine dehydrogenase bifunctional enzyme identical to lysine-ketoglutarate reductase/saccharopine dehydrogenase GI:2052508 from [Arabidopsis thaliana]
AT5G62360	4.89E-05	0.93	0.40	2.49	1.17	2.67	2.92	1.09	invertase/pectin methylesterase inhibitor family protein similar to pectinesterase from Arabidosis thaliana SP|Q42534, Lycopersicon esculentum SP|Q43143; contains Pfam profile PF04043: Plant invertase/pectin methylesterase inhibitor
AT3G16530	8.54E-04	0.36	1.05	0.97	1.50	2.67	1.43	0.54	legume lectin family protein contains Pfam domain, PF00139: Legume lectins beta domain
AT2G06050	3.83E-03	0.59	0.64	1.56	1.24	2.66	1.93	0.72	12-oxophytodienoate reductase (OPR3) / delayed dehiscence1 (DDE1) nearly identical to DELAYED DEHISCENCE1 [GI:7688991] and to OPR3 [GI:10242314]; contains Pfam profile PF00724:oxidoreductase, FAD/FMN-binding; identical to cDNA OPDA-reductase homolog GI:5059114
AT1G54040	5.83E-04	0.68	0.77	1.79	1.82	2.66	2.37	0.89	kelch repeat-containing protein contains Pfam PF01344: Kelch motif (4 repeats); similar to jsimilar to epithiospecifier (GI:16118838) [Arabidopsis thaliana] isoform contains AT-AG splice sites at intron
AT3G57520	5.62E-04	0.63	0.63	1.66	1.60	2.65	2.54	0.96	alkaline alpha galactosidase, putative similar to alkaline alpha galactosidase II [Cucumis melo] GI:29838631; contains Pfam profile PF05691: Raffinose synthase or seed imbibition protein Sip1
AT1G29410	1.30E-03	0.90	0.80	2.38	1.71	2.65	2.14	0.81	phosphoribosylanthranilate isomerase, putative strong similarity to GI:619749 from [Arabidopsis thaliana] (Plant Cell 7 (4), 447-461 (1995))
AT1G31550	1.39E-04	0.63	0.59	1.67	1.52	2.64	2.60	0.99	GDSL-motif lipase, putative similar to lipase [Arabidopsis thaliana] GI:1145627; contains InterPro Entry IPR001087 Lipolytic enzyme, G-D-S-L family
AT2G30930	2.71E-04	0.67	0.59	1.76	1.31	2.64	2.24	0.85	expressed protein
AT1G52400	1.39E-04	0.53	0.77	1.38	1.38	2.62	1.80	0.69	glycosyl hydrolase family 1 protein / beta-glucosidase, putative (BG1) contains Pfam PF00232 : Glycosyl hydrolase family 1 domain; TIGRFAM TIGR01233: 6-phospho-beta-galactosidase; identical to GI:6651430 from [Arabidopsis thaliana]
AT1G31180;AT5G	2.89E-03	0.64	0.62	1.67	1.45	2.61	2.33	0.89	[AT1G31180, 3-isopropylmalate dehydrogenase, chloroplast, putative strong similarity to SP|P29102 3-isopropylmalate dehydrogenase, chloroplast precursor [Brassica napus]; EST gb|F14478 comes from this gene];[AT5G14200, 3-isopropylmalate dehydrogenase, chloroplast, putative strong similarity to SP|P29102 [illegible]
AT5G01810	3.15E-03	0.79	0.65	2.05	1.15	2.60	1.76	0.68	CBL-interacting protein kinase 15 (CIPK15) identical to CBL-interacting protein kinase 15 [Arabidopsis thaliana] gi|13249134|gb|AAK16692; identical to novel serine/threonine protein kinase [Arabidopsis thaliana] gi|1777312|dbj|BAA06311; contains Pfam profiles PF00069: Protein kinase domain and PF03822: NAF d[illegible]
AT5G53420	1.39E-04	0.64	0.54	1.66	1.54	2.60	2.87	1.10	expressed protein
AT3G04720	1.77E-04	0.44	0.89	1.14	1.31	2.60	1.48	0.57	hevein-like protein (HEL) identical to SP|P43082 Hevein-like protein precursor [Arabidopsis thaliana]; similar to SP|P09762 Wound-induced protein WIN2 precursor [Solanum tuberosum]; contains Pfam profile PF00187: Chitin recognition protein
AT3G30775	2.03E-03	0.90	0.51	2.33	1.35	2.60	2.63	1.01	proline oxidase, mitochondrial / osmotic stress-responsive proline dehydrogenase (POX) (PRO1) (ERD5) nearly identical to SP|P92983 Proline oxidase, mitochondrial precursor (EC 1.5.3.-) (Osmotic stress- induced proline dehydrogenase) [Arabidopsis thaliana]; identical to cDNA proline oxidase precursor GI:1817543
AT5G02940	5.62E-04	0.55	0.68	1.44	1.69	2.60	2.47	0.95	expressed protein
AT4G30450	1.51E-02	0.84	0.78	2.17	1.76	2.59	2.26	0.87	glycine-rich protein
AT1G17620	2.07E-03	0.85	0.66	2.19	1.16	2.59	1.76	0.68	expressed protein
AT1G18350	1.66E-04	0.81	0.71	2.10	1.45	2.59	2.03	0.79	mitogen-activated protein kinase kinase (MAPKK), putative (MKK7) mitogen-activated protein kinase kinase (MAPKK) family, PMID:12119167
AT2G47600	1.20E-04	0.44	0.87	1.12	2.51	2.55	2.89	1.13	magnesium/proton exchanger (MHX1) identical to magnesium/proton exchanger AtMHX [Arabidopsis thaliana] gi|6492237|gb|AAF14229; Ca2+:Cation Antiporter (CaCA) Family member PMID:11500563
AT4G29700	9.37E-04	0.67	0.72	1.72	1.62	2.55	2.26	0.89	type I phosphodiesterase/nucleotide pyrophosphatase family protein similar to SP|P22413 Ectonucleotide pyrophosphatase/phosphodiesterase 1 (Plasma-cell membrane glycoprotein PC-1) [Includes: Alkaline phosphodiesterase I (EC 3.1.4.1); Nucleotide pyrophosphatase (EC 3.6.1.9)] (Homo sapiens); contains Pfam p[illegible]
AT4G03060	4.26E-03	0.68	0.52	1.73	1.49	2.55	2.86	1.12	2-oxoglutarate-dependent dioxygenase, putative (AOP2) nearly identical to GI:16118891; contains Pfam profile PF03171: 2OG-Fe(II) oxygenase superfamily domain. The gene sequence is frameshifted, this could be a pseudogene or a sequencing error may exist; identical to cDNA AOP2 GI:16118890
AT5G06870	6.63E-04	0.57	0.64	1.46	1.60	2.55	2.52	0.99	polygalacturonase inhibiting protein 2 (PGIP2) identical to polygalacturonase inhibiting protein 2 (PGIP2) [Arabidopsis thaliana] gi|7800201|gb|AAF69828; contains leucine rich-repeat (LRR) domains Pfam:PF00560, INTERPRO:IPR001611
AT4G27860	4.20E-04	0.63	0.74	1.61	1.86	2.54	2.49	0.98	integral membrane family protein contains Pfam PF01988: Integral membrane protein
AT4G01080	8.37E-03	0.77	0.83	1.95	1.52	2.53	1.83	0.72	expressed protein
AT1G29330	3.18E-04	0.82	0.77	2.08	1.63	2.53	2.14	0.84	ER lumen protein retaining receptor (ERD2) / HDEL receptor identical to SP:P35402 ER lumen protein retaining receptor (HDEL receptor) (Arabidopsis thaliana)
AT2G22330	3.77E-04	0.51	0.74	1.27	1.90	2.51	2.59	1.03	cytochrome P450, putative similar to cytochrome P450 79B2 (SP:O81346) [Arabidopsis thaliana]
AT1G24070	1.20E-03	0.58	0.69	1.45	2.01	2.50	2.92	1.17	glycosyl transferase family 2 protein similar to beta-(1-3)-glucosyl transferase GB:AAC62210 GI:3687658 from [Bradyrhizobium japonicum], cellulose synthase from Agrobacterium tumeficiens [gi:710492] and Agrobacterium radiobacter [gi:710493]; contains Pfam glycosyl transferase, group 2 family protein domain P[illegible]
AT1G66100	2.37E-03	0.73	0.78	1.82	1.42	2.49	1.83	0.74	thionin, putative similar to thionin [Arabidopsis thaliana] GI:1181533
AT3G16470	8.33E-05	0.56	0.68	1.38	1.44	2.48	2.12	0.85	jacalin lectin family protein contains Pfam profile: PF01419 jacalin-like lectin domain; similar to myrosinase-binding protein homolog [Arabidopsis thaliana] GI:2997767
AT1G59710	7.78E-05	0.74	0.52	1.84	1.29	2.48	2.47	1.00	expressed protein contains Pfam profile: PF04601 protein of unknown function (DUF569)
AT5G23020	5.87E-03	0.80	0.77	1.99	1.87	2.48	2.43	0.98	2-isopropylmalate synthase 2 (IMS2) identical to 2-isopropylmalate synthase (IMS2) [Arabidopsis thaliana] GI:12330689
AT3G54640	9.62E-04	0.49	0.91	1.21	1.84	2.48	2.03	0.82	tryptophan synthase, alpha subunit (TSA1) identical to gi:619753
AT2G43530	3.15E-03	0.59	0.92	1.45	1.53	2.47	1.65	0.67	trypsin inhibitor, putative similar to SP|P26780 Trypsin inhibitor 2 precursor (MTI-2) (Sinapis alba)
AT1G70690	1.34E-04	0.56	0.71	1.37	1.64	2.46	2.32	0.94	kinase-related contains Pfam PF01657:Domain of unknown function; similar to receptor-like protein kinase 4 GI:13506745 [Arabidopsis thaliana]
AT2G34070	2.05E-04	0.83	0.74	2.03	1.65	2.44	2.22	0.91	expressed protein
AT1G78660	2.97E-03	0.84	0.79	2.06	1.50	2.43	1.90	0.78	gamma-glutamyl hydrolase, putative / gamma-Glu-X carboxypeptidase, putative / conjugase, putative similar to SP|O65355 Gamma-glutamyl hydrolase precursor (EC 3.4.19.9) (Gamma-Glu-X carboxypeptidase) (Conjugase) (GH) (Arabidopsis thaliana)
AT5G13550	2.00E-03	0.74	0.87	1.80	2.00	2.43	2.31	0.95	sulfate transporter family protein similar to sulfate transporter [Arabidopsis thaliana] GI:3777483; contains Pfam profiles PF00916: Sulfate transporter family, PF01740: STAS domain
AT1G78270	1.05E-04	0.59	0.76	1.44	1.73	2.43	2.28	0.94	UDP-glucose glucosyltransferase, putative similar to UDP-glucose glucosyltransferase GI:3928543 from [Arabidopsis thaliana]
AT1G06160	2.12E-02	0.50	0.92	1.22	1.28	2.42	1.39	0.58	ethylene-responsive factor, putative similar to ethylene response factor 1 GB:AAD03544 GI:4128208 from [Arabidopsis thaliana]
AT3G23570	1.72E-04	0.48	0.79	1.17	1.52	2.42	1.92	0.79	dienelactone hydrolase family protein similar to SP|Q9ZT66 Endo-1,3;1,4-beta-D-glucanase precursor (EC 3.2.1.-) (Zea mays); contains Pfam profile: PF01738 dienelactone hydrolase family
AT5G23860	7.78E-05	0.71	0.67	1.72	1.65	2.41	2.46	1.02	tubulin beta-8 chain (TUB8) (TUBB8) identical to SP|P29516 Tubulin beta-8 chain (Arabidopsis thaliana); supporting cDNA gi|15451225|gb|AY054693.1|
AT4G21400	1.13E-03	0.62	0.86	1.50	1.83	2.41	2.12	0.88	protein kinase family protein contains Pfam domain PF00069: Protein kinase domain
AT3G63380	1.48E-02	0.62	0.84	1.49	1.79	2.40	2.14	0.89	calcium-transporting ATPase, plasma membrane-type, putative / Ca(2+)-ATPase, putative (ACA12) identical to SP|Q9LY77 Potential calcium-transporting ATPase 12, plasma membrane-type (EC 3.6.3.8) (Ca(2+)-ATPase isoform 12) {Arabidopsis thaliana}; similar to SP|Q9LF79 Calcium-transporting ATPase 8, plasma me[illegible]
AT4G13410	3.56E-03	0.62	0.77	1.48	1.71	2.39	2.21	0.92	glycosyl transferase family 2 protein similar to beta-(1-3)-glucosyl transferase GB:AAC62210 GI:3687658 from [Bradyrhizobium japonicum], cellulose synthase from Agrobacterium tumeficiens [gi:710492] and Agrobacterium radiobacter [gi:710493]; contains Pfam glycosyl transferase, group 2 family protein domain P[illegible]
AT2G29440	2.62E-02	0.75	0.93	1.79	0.98	2.39	1.05	0.44	glutathione S-transferase, putative
AT3G62150	7.45E-03	0.59	0.94	1.41	2.63	2.39	2.79	1.17	multidrug resistant (MDR) ABC transporter, putative similar to multidrug-resistant protein CjMDR1 GI:14715462 from [Coptis japonica]; contains Pfam profiles PF00005: ABC transporter, PF00664: ABC transporter transmembrane region
AT4G13770	1.22E-03	0.62	0.64	1.46	1.97	2.36	3.09	1.31	cytochrome P450 family protein
AT5G65280	1.27E-04	0.77	0.81	1.81	1.46	2.35	1.79	0.76	lanthionine synthetase C-like family protein contains Pfam domain, PF05147: Lanthionine synthetase C-like protein
AT4G39980	2.52E-05	0.56	0.65	1.32	1.83	2.34	2.82	1.21	2-dehydro-3-deoxyphosphoheptonate aldolase 1 / 3-deoxy-D-arabino-heptulosonate 7-phosphate synthase 1 / DAHP synthetase 1 (DHS1) nearly identical to SP|P29965
AT3G22890	7.78E-05	0.52	0.69	1.21	1.40	2.33	2.03	0.87	sulfate adenylyltransferase 1 / ATP-sulfurylase 1 (APS1) nearly identical to ATP sulfurylase (APS1) [Arabidopsis thaliana] GI:6606509
AT4G38470	7.94E-04	0.77	0.62	1.81	1.34	2.33	2.17	0.93	protein kinase family protein similar to protein kinase [gi:170047] from Glycine max; contains Pfam protein kinase domain PF00069
AT4G30530	3.24E-04	0.53	0.76	1.23	1.21	2.33	1.59	0.68	defense-related protein, putative strong similarity to defense-related protein [Brassica carinata] GI:14009290; contains Pfam profile PF00117: glutamine amidotransferase class-I
AT3G16390	3.41E-04	0.70	0.63	1.62	1.57	2.33	2.52	1.08	jacalin lectin family protein similar to myrosinase-binding protein homolog [Arabidopsis thaliana] GI:2997767, epithiospecifier [Arabidopsis thaliana] GI:16118845; contains Pfam profiles PF01419 jacalin-like lectin family, PF01344 Kelch motif
AT1G80190	1.15E-04	0.75	0.70	1.72	1.49	2.31	2.12	0.92	expressed protein
AT5G67480	2.05E-04	0.66	0.61	1.53	1.45	2.31	2.37	1.03	TAZ zinc finger family protein / BTB/POZ domain-containing protein contains Pfam PF00651 : BTB/POZ domain; contains Pfam PF02135 : TAZ zinc finger; simiar to Chain A, Taz2 Domain Of The Transcriptional Adaptor Protein Cbp (GI:11514507) [Homo sapiens]; similar to (SP:Q09472) E1A-associated protein p300 (SP:[illegible]
AT4G14680	1.59E-03	0.49	0.67	1.14	1.72	2.30	2.57	1.12	sulfate adenylyltransferase 3 / ATP-sulfurylase 3 (APS3) identical to ATP sulfurylase (APS3) [Arabidopsis thaliana] GI:1575327
AT4G30460	1.26E-02	0.88	0.91	2.03	2.08	2.30	2.28	1.00	glycine-rich protein
AT1G19180	7.97E-03	0.72	0.54	1.65	1.17	2.29	2.16	0.94	expressed protein
AT1G06180	1.38E-03	0.67	0.81	1.53	1.39	2.28	1.72	0.75	myb family transcription factor identical to GB:CAA90748 GI:1263093 from [Arabidopsis thaliana];contains PFAM profile:PF00249
AT5G43170	1.07E-03	0.90	0.89	2.05	1.44	2.27	1.62	0.71	zinc finger (C2H2 type) protein 3 (AZF3) identical to Cys2/His2-type zinc finger protein 3 [Arabidopsis thaliana] gi|6009889|dbj|BAA85109
AT4G19370	1.31E-02	0.51	1.17	1.15	1.64	2.26	1.41	0.62	hypothetical protein
AT5G05730	1.23E-04	0.48	0.86	1.08	1.89	2.26	2.19	0.97	anthranilate synthase, alpha subunit, component I-1 (ASA1) identical to SP|P32068
AT1G14120	1.46E-03	0.94	0.81	2.12	1.12	2.26	1.39	0.62	2-oxoglutarate-dependent dioxygenase, putative similar to adventitious rooting related oxygenase ARRO-1 from Malus x domestica, gi|3492806; contains Pfam domain PF03171, 2OG-Fe(II) oxygenase superfamily
AT1G25083;AT1G	2.24E-04	0.51	0.87	1.15	1.43	2.26	1.63	0.72	[AT1G25083, anthranilate synthase beta subunit, putative strong similarity to anthranilate synthase beta subunit GI:403434 from (Arabidopsis thaliana); similar to ESTs dbj|AV540153.1, dbj|AV557490.1, gb|AI997696.1, gb|AW004516.1, dbj|AV521371.1];[AT1G24909, anthranilate synthase beta subunit, putative st[illegible]
AT5G50950	7.78E-05	0.54	0.77	1.23	1.43	2.25	1.85	0.82	fumarate hydratase, putative / fumarase, putative similar to SP|P55250 Fumarate hydratase, mitochondrial precursor (EC 4.2.1.2) (Fumarase) (Rhizopus oryzae); contains Pfam profile PF00206: Lyase
AT3G13672	1.95E-02	0.85	0.91	1.91	1.59	2.25	1.74	0.77	seven in absentia (SINA) family protein low similarity to SP|P21461 Developmental protein seven in absentia (Drosophila melanogaster); contains Pfam profile PF03145: Seven in absentia protein family
AT1G44790	1.26E-02	0.94	0.88	2.11	2.11	2.25	2.40	1.07	ChaC-like family protein contains Pfam profile: PF04752 ChaC-like protein
AT1G44350	1.56E-03	0.62	0.65	1.40	1.27	2.25	1.95	0.87	IAA-amino acid hydrolase 6, putative (ILL6) / IAA-Ala hydrolase, putative virtually identical to gr1-protein from [Arabidopsis thaliana] GI:3559811; similar to IAA-amino acid hydrolase GI:3421384 from [Arabidopsis thaliana]; contains TIGRfam profile TIGR01891: amidohydrolase; contains Pfam profile PF01546: Peptida[illegible]
AT1G45145	2.75E-02	0.88	0.90	1.98	1.63	2.24	1.82	0.81	thioredoxin H-type 5 (TRX-H-5) (TOUL) identical to SP|Q39241 Thioredoxin H-type 5 (TRX-H-5) {Arabidopsis thaliana}; identical to cDNA (TOUL) mRNA for thioredoxin GI:992965
AT5G07690	2.56E-02	0.82	0.79	1.83	1.39	2.24	1.76	0.79	myb family transcription factor (MYB29) similar to myb transcription factor GI:3941436 from [Arabidopsis thaliana]
AT4G21410	2.10E-04	0.66	0.82	1.47	2.45	2.24	2.99	1.34	protein kinase family protein contains Pfam PF00069: Protein kinase domain
AT2G36380	3.18E-04	0.57	0.78	1.28	2.60	2.23	3.31	1.48	ABC transporter family protein related to multi drug resistance proteins and P-glycoproteins
AT1G52000	3.65E-03	0.69	0.71	1.53	1.45	2.23	2.04	0.91	jacalin lectin family protein similar to myrosinase binding protein [Brassica napus] GI:1711296, myrosinase-binding protein homolog [Arabidopsis thaliana] GI:2997767; contains Pfam profile: PF01419 jacalin-like lectin domain
AT1G62560	1.50E-02	0.80	0.84	1.76	1.77	2.22	2.11	0.95	flavin-containing monooxygenase family protein / FMO family protein similar to flavin-containing monooxygenase GB:AAA21178 GI:349534 SP|P32417 from [Oryctolagus cuniculus]; contains Pfam profile PF00743 Flavin-binding monooxygenase-like
AT4G03400	5.42E-03	0.69	0.65	1.53	1.23	2.22	1.90	0.86	auxin-responsive GH3 family protein similar to auxin-responsive GH3 product [Glycine max] GI:18591; contains Pfam profile PF03321: GH3 auxin-responsive promoter
AT4G38540	7.04E-04	0.60	0.91	1.33	1.67	2.22	1.83	0.83	monooxygenase, putative (MO2) identical to GI:3426064
AT5G38120	2.45E-03	0.90	0.78	1.98	1.27	2.21	1.62	0.73	4-coumarate--CoA ligase family protein / 4-coumaroyl-CoA synthase family protein similar to 4CL2, Arabidopsis thaliana [gi:12229665], 4CL1, Nicotiana tabacum [gi:12229631]; contains Pfam AMP-binding enzyme domain PF00501
AT5G51550	5.62E-04	0.71	0.60	1.58	1.18	2.21	1.96	0.89	phosphate-responsive 1 family protein similar to phi-1 (phosphate-induced gene) [Nicotiana tabacum] GI:3759184; contains Pfam profile PF04674: Phosphate-induced protein 1 conserved region
AT1G23090	6.18E-03	0.60	0.85	1.32	1.89	2.20	2.21	1.01	sulfate transporter, putative similar to sulfate transporter [Arabidopsis thaliana] GI:2285885; contains Pfam profiles PF00916: Sulfate transporter family, PF01740: STAS domain
AT2G26560	2.74E-04	0.58	0.83	1.28	1.46	2.20	1.75	0.80	patatin, putative similar to patatin-like latex allergen [Hevea brasiliensis][PMID:10589016]; contains patatin domain PF01734
AT1G31690	3.29E-03	0.85	0.71	1.87	1.86	2.19	2.62	1.19	copper amine oxidase, putative similar to copper amine oxidase [Lens culinaris] gi|15451834|gb|AAB34918
AT3G16430;AT3G	2.23E-03	0.91	0.81	2.00	1.39	2.19	1.72	0.78	[AT3G16430, jacalin lectin family protein similar to myrosinase binding protein [Brassica napus] GI:1711296; contains Pfam profile PF01419 jacalin-like lectin domain];[AT3G16420, jacalin lectin family protein similar to myrosinase binding protein [Brassica napus] GI:1711296; contains Pfam profile: PF01419 jacalin-li[illegible]
AT1G22180	5.89E-03	0.68	0.84	1.48	1.26	2.19	1.50	0.69	SEC14 cytosolic factor family protein / phosphoglyceride transfer family protein contains Pfam profile: PF00650 CRAL/TRIO domain; similar to polyphosphoinositide binding protein Ssh1p (GI:2739044) [Glycine max]; similar to Phosphatidylinositol Transfer Protein Sec14p (GI:2780955) [Saccharomyces cerevisiae]
AT2G04080;AT2G	3.36E-04	0.69	0.78	1.51	1.48	2.18	1.90	0.87	[AT2G04080, MATE efflux family protein similar to hypothetical protein GB:AAC27412; contains Pfam profile PF01554: Uncharacterized membrane protein family];[AT2G04066, MATE efflux protein-related similar to multidrug secondary transporter-like TRANSPARENT TESTA 12 protein (Swiss-Prot:Q9LYT3) [Arabido[illegible]
AT5G67080	1.69E-02	0.57	0.72	1.25	1.58	2.18	2.18	1.00	protein kinase family protein contains protein kinase domain, Pfam:PF00069
AT4G21910	6.23E-04	0.64	0.80	1.39	2.00	2.18	2.50	1.15	MATE efflux family protein similar to ripening regulated protein DDTFR18 [Lycopersicon esculentum] GI:12231296; contains Pfam profile PF01554: Uncharacterized membrane protein family
AT5G16190	2.82E-04	0.66	0.81	1.44	1.81	2.17	2.24	1.03	glycosyl transferase family 2 protein similar to cellulose synthase from Agrobacterium tumeficiens [gi:710492] and Agrobacterium radiobacter [gi:710493]; contains Pfam glycosyl transferase, group 2 family protein domain PF00535
AT5G29000	2.74E-04	0.93	0.75	2.01	1.10	2.16	1.47	0.68	myb family transcription factor contains Pfam profile: PF00249 myb-like DNA-binding domain
AT5G01500	2.52E-05	0.60	0.78	1.28	1.33	2.15	1.70	0.79	mitochondrial substrate carrier family protein contains Pfam profile: PF00153 mitochondrial carrier protein
AT2G38860	1.94E-03	0.35	1.23	0.75	2.01	2.15	1.64	0.76	protease I (pfpI)-like protein (YLS5) contains Pfam profile PF01965: DJ-1/PfpI family; supporting cDNA gi|13122287|dbj|AB047808.1|; identical to protease I (pfpI)-like protein [Arabidopsis thaliana] GI:13122288, cDNA protease I (pfpI)-like protein GI:13122287
AT3G07340	1.30E-02	0.85	0.83	1.81	1.31	2.14	1.57	0.73	basic helix-loop-helix (bHLH) family protein contains Pfam profile: PF00010 helix-loop-helix DNA-binding domain
AT4G39950	4.56E-03	0.52	1.09	1.12	2.96	2.13	2.72	1.28	cytochrome P450 79B2, putative (CYP79B2) identical to cytochrome P450 (79B2) SP:O81346 from [Arabidopsis thaliana]
AT3G15950	2.30E-03	0.80	0.60	1.69	1.08	2.12	1.80	0.85	DNA topoisomerase-related similar to DNA topoisomerase IV subunit A (GI:26454107) [Mycoplasma penetrans]
AT1G22360	6.48E-04	0.61	0.81	1.28	1.64	2.11	2.02	0.96	UDP-glucoronosyl/UDP-glucosyl transferase family protein contains Pfam profile: PF00201 UDP-glucoronosyl and UDP-glucosyl transferase
AT5G67310	1.96E-02	0.78	0.95	1.65	1.73	2.11	1.82	0.86	cytochrome P450 family protein
AT3G03190	4.12E-02	0.77	0.73	1.62	1.51	2.11	2.09	0.99	glutathione S-transferase, putative identical to glutathione S-transferase GB:AAB09584 from [Arabidopsis thaliana]
AT3G60130	1.77E-04	0.81	0.71	1.70	1.61	2.11	2.27	1.08	glycosyl hydrolase family 1 protein / beta-glucosidase, putative (YLS1) contains Pfam PF00232 : Glycosyl hydrolase family 1 domain; TIGRFAM TIGR01233: 6-phospho-beta-galactosidase; similar to amygdalin hydrolase isoform AH I precursor (GI:16757966) [Prunus serotina]; identical to cDNA YLS1 mRNA for beta-gluc[illegible]
AT5G49280	5.77E-03	0.82	0.82	1.73	1.28	2.10	1.56	0.74	hydroxyproline-rich glycoprotein family protein contains proline-rich extensin domains, INTERPRO:IPR002965
AT1G27130	2.41E-03	0.59	0.88	1.25	1.40	2.10	1.59	0.76	glutathione S-transferase, putative similar to glutathione S-transferase GB: AAF22517 GI:6652870 from [Papaver somniferum]
AT4G04830	4.67E-02	0.80	0.70	1.68	1.20	2.10	1.73	0.82	methionine sulfoxide reductase domain-containing protein / SeIR domain-containing protein low similarity to pilin-like transcription factor [Homo sapiens] GI:5059062, SP|P14930 Peptide methionine sulfoxide reductase msrA/msrB (EC 1.8.4.6) (Neisseria gonorrhoeae); contains Pfam profile PF01641: SelR domain
AT1G35670	6.19E-03	0.80	0.82	1.68	1.08	2.10	1.32	0.63	calcium-dependent protein kinase 2 (CDPK2) identical to calcium-dependent protein kinase [Arabidopsis thaliana] gi|604881|dbj|BAA04830; contains protein kinase domain, Pfam:PF00069; contains EF hand domain (calcium-binding EF-hand), Pfam:PF00036, INTERPRO:IPR002048
AT3G60980	4.19E-04	0.85	0.85	1.78	1.26	2.09	1.48	0.71	pentatricopeptide (PPR) repeat-containing protein contains Pfam profile PF01535: PPR repeat
AT3G19680	7.38E-03	0.70	0.58	1.45	1.26	2.08	2.15	1.03	expressed protein
AT2G14750	4.73E-04	0.57	0.70	1.18	1.21	2.08	1.72	0.83	adenylylsulfate kinase 1 (AKN1) identical to adenylylsulfate kinase 1, chloroplast precursor (APS kinase, Adenosine-5'phosphosulfate kinase, ATP adenosine-5'- phosphosulfate 3'-phosphotransferase) [Arabidopsis thaliana] SWISS-PROT:Q43295
AT1G77640	4.27E-02	1.04	0.84	2.16	1.13	2.08	1.34	0.64	AP2 domain-containing transcription factor, putative Similar to DREB1A (GP:3660548) [Arabidopsis thaliana]
AT5G63970	1.25E-02	0.76	0.78	1.57	1.45	2.08	1.86	0.90	copine-related low similarity to SP|Q99829 Copine I (Homo sapiens)
AT5G13740	1.56E-04	0.65	0.73	1.35	1.33	2.08	1.82	0.88	sugar transporter family protein contains Pfam profile PF00083: major facilitator superfamily protein
AT4G35640;AT4G	6.48E-04	0.61	0.70	1.26	1.54	2.08	2.21	1.06	[AT4G35640, serine O-acetyltransferase, putative similar to serine acetyltransferase, Arabidopsis thaliana, GI:905391; contains Pfam profile PF00132: Bacterial transferase hexapeptide (four repeats)];[AT4G35630, phosphoserine aminotransferase, chloroplast (PSAT) identical to Phosphoserine aminotransferase, chl[illegible]
AT5G25240	3.47E-02	0.83	0.67	1.71	1.07	2.07	1.61	0.78	expressed protein
AT4G18950	3.63E-03	0.74	0.80	1.53	1.28	2.06	1.60	0.78	ankyrin protein kinase, putative similar to ankyrin-kinase [Medicago truncatula] gi|18700701|gb|AAL78674
AT1G72450	5.82E-03	0.71	0.69	1.46	1.24	2.05	1.80	0.88	expressed protein
AT1G61100	9.74E-04	0.68	0.54	1.39	1.34	2.05	2.47	1.21	disease resistance protein (TIR class), putative domain signature TIR exists, suggestive of a disease resistance protein.
AT2G39770	7.78E-05	0.54	0.92	1.10	1.62	2.04	1.77	0.87	GDP-mannose pyrophosphorylase (GMP1) identical to GDP-mannose pyrophosphorylase from Arabidopsis thaliana [GI:3598958]; updated per Conklin PL et al, PNAS 1999, 96(7):4198-203
AT1G12240	9.81E-04	0.83	0.70	1.69	1.32	2.04	1.89	0.93	beta-fructosidase (BFRUCT4) / beta-fructofuranosidase / invertase, vacuolar identical to beta-fructosidase GI:1871503 from [Arabidopsis thaliana]; contains Pfam profile PF00251:Glycosyl hydrolases family 32; identical to cDNA beta-fructosidase (vacuolar form) GI:1321683; similar to SP:Q43857
AT1G69760	1.24E-02	0.90	0.72	1.82	1.05	2.02	1.46	0.72	expressed protein
AT5G59220	1.73E-03	0.62	0.64	1.25	1.59	2.02	2.46	1.22	protein phosphatase 2C, putative / PP2C, putative ABA induced protein phosphatase 2C, Fagus sylvatica, EMBL:FSY277743
AT4G25810	3.82E-02	0.94	0.69	1.90	1.49	2.02	2.15	1.06	xyloglucan:xyloglucosyl transferase, putative / xyloglucan endotransglycosylase, putative / endo-xyloglucan transferase, putative (XTR6) identical to xyloglucan endotransglycosylase-related protein GI:1244758 from [Arabidopsis thaliana]
AT1G23480	3.34E-03	0.66	0.77	1.33	1.32	2.02	1.72	0.85	glycosyl transferase family 2 protein similar to cellulose synthase from Agrobacterium tumeficiens [gi:710492] and Agrobacterium radiobacter [gi:710493]; contains Pfam glycosyl transferase, group 2 family protein domain PF00535 isoform contains GG acceptor splice site at intron 1
AT3G49120	1.65E-02	0.95	0.87	1.92	1.03	2.02	1.19	0.59	peroxidase, putative identical to peroxidase [Arabidopsis thaliana] gi|405611|emb|CAA50677
AT1G10070	2.83E-03	0.90	0.77	1.83	1.31	2.02	1.70	0.84	branched-chain amino acid aminotransferase 2 / branched-chain amino acid transaminase 2 (BCAT2) identical to SP|Q9M439 Branched-chain amino acid aminotransferase 2, chloroplast precursor (EC 2.6.1.42) (Atbcat-2) {Arabidopsis thaliana}; contains Pfam profile: PF01063 aminotransferase class IV
AT1G62540	4.25E-03	0.66	0.85	1.33	1.43	2.01	1.68	0.83	flavin-containing monooxygenase family protein / FMO family protein similar to flavin-containing monooxygenase GB:AAA21178 GI:349534 from Oryctolagus cuniculus [SP|P32417], SP|P97501 from Mus musculus; contains Pfam profile PF00743 Flavin-binding monooxygenase-like
AT5G05440	7.30E-03	0.79	0.84	1.59	1.33	2.01	1.58	0.79	expressed protein low similarity to cytokinin-specific binding protein [Vigna radiata] GI:4190976
AT3G25760	8.61E-05	0.77	0.80	1.55	1.40	2.00	1.74	0.87	early-responsive to dehydration stress protein (ERD12) nearly identical to early-responsive to dehydration (ERD12) protein [GI:15320414]; similar to allene oxide cyclase GI:8977961 from [Lycopersicon esculentum]; identical to cDNA ERD12 partial cds GI:15320413
AT2G44500	3.63E-02	0.61	0.90	1.23	1.60	2.00	1.79	0.89	expressed protein contains Pfam PF03138: Plant protein family. The function of this family of plant proteins is unknown; previously annotated as 'axi 1 protein from Nicotiana tabacum -related' based on similarity to axi 1 protein (GB:X80301) (GI:559920) from [Nicotiana tabacum], which, due to scientific fraud was r[illegible]
AT5G55120	1.56E-04	0.55	0.74	1.09	1.66	2.00	2.25	1.13	expressed protein strong similarity to unknown protein (pir||T04808)
AT4G19390	4.70E-04	0.78	0.69	1.56	1.37	1.99	2.00	1.00	expressed protein
AT3G53180	2.71E-03	0.75	0.78	1.48	1.56	1.99	2.00	1.01	glutamine synthetase, putative similar to glutamine synthetase (glutamate--ammonia ligase) [Bacillus subtilis] SWISS-PROT:P12425
AT5G24290	6.19E-04	0.76	0.72	1.51	1.43	1.98	1.98	1.00	integral membrane family protein contains Pfam domain PF01988: Integral membrane protein
AT5G03630	9.32E-03	0.64	0.87	1.27	1.20	1.98	1.38	0.70	monodehydroascorbate reductase, putative monodehydroascorbate reductase (NADH), cucumber, PIR:JU0182
AT1G50040	2.20E-02	0.66	0.58	1.30	1.00	1.98	1.71	0.87	expressed protein
AT2G38310	9.83E-04	0.52	0.96	1.02	1.64	1.98	1.71	0.86	expressed protein low similarity to early flowering protein 1 [Asparagus officinalis] GI:1572683, SP|P80889 Ribonuclease 1 (EC 3.1.-.-) (Panax ginseng)
AT1G21100;AT1G	1.74E-02	0.43	1.27	0.86	1.73	1.98	1.36	0.69	[AT1G21100, O-methyltransferase, putative similar to GI:2781394];[AT1G21130, O-methyltransferase, putative similar to GI:2781394];[AT1G21110, O-methyltransferase, putative similar to GI:2781394]
AT3G10260	3.96E-04	0.84	0.77	1.66	1.42	1.97	1.84	0.93	reticulon family protein weak similarity to Nogo-C protein [Rattus norvegicus] GI:6822251; contains Pfam profile PF02453: Reticulon; identical to cDNA GI:32331854
AT4G34710	2.23E-03	0.70	0.76	1.37	1.92	1.97	2.51	1.27	arginine decarboxylase 2 (SPE2) identical to SP|O23141 Arginine decarboxylase 2 (EC 4.1.1.19) (ARGDC 2) (ADC 2) (ADC-N) (Arabidopsis thaliana)
AT2G15760	6.27E-04	0.62	0.81	1.23	1.73	1.97	2.14	1.09	calmodulin-binding protein similar to AR781 GI:1669593 from [Arabidopsis thaliana]; AR781 complements pheromone receptor deficient mutant of Shizosaccharomyces pombe
AT1G22380	4.42E-04	0.77	0.59	1.51	1.56	1.96	2.66	1.36	UDP-glucoronosyl/UDP-glucosyl transferase family protein contains Pfam profile: PF00201 UDP-glucoronosyl and UDP-glucosyl transferase
AT2G28120	1.46E-03	0.62	0.69	1.21	1.33	1.96	1.93	0.99	nodulin family protein similar to nodulin-like protein [Arabidopsis thaliana] GI:3329368, nodule-specific protein Nlj70 [Lotus japonicus] GI:3329366
AT1G18590	6.98E-03	0.61	0.97	1.20	1.97	1.96	2.03	1.04	sulfotransferase family protein similar to SP|P52837 Flavonol 4'-sulfotransferase (EC 2.8.2.-) (F4-ST) [Flaveria chloraefolia]; contains Pfam profile PF00685: Sulfotransferase domain
AT5G60300	7.18E-05	0.64	0.75	1.25	1.94	1.95	2.60	1.33	lectin protein kinase family protein contains Pfam domains, PF00069: Protein kinase domain and PF00139: Legume lectins beta domain
AT3G14310	1.17E-04	0.71	0.76	1.37	1.51	1.95	1.98	1.02	pectinesterase family protein contains Pfam profiles: PF01095 pectinesterase, PF04043 plant invertase/pectin methylesterase inhibitor ;similar to pectin methylesterase GB:Q42534 from [Arabidopsis thaliana]
AT1G49530	1.67E-03	0.89	0.77	1.74	1.44	1.94	1.87	0.96	geranylgeranyl pyrophosphate synthase (GGPS6) / GGPP synthetase / farnesyltranstransferase identical to gi:2578821; similar to geranyl geranyl pyrophosphate synthase GI:2578822 from [Arabidopsis thaliana]
AT1G19640	1.12E-04	0.68	0.81	1.33	2.89	1.94	3.56	1.83	S-adenosyl-L-methionine:jasmonic acid carboxyl methyltransferase (JMT) nearly identical to gi:13676829
AT1G10700	7.78E-05	0.60	0.80	1.16	1.43	1.94	1.78	0.92	ribose-phosphate pyrophosphokinase 3 / phosphoribosyl diphosphate synthetase 3 (PRS3) nearly identical to phosphoribosyl diphosphate synthase GI:4902470 from [Arabidopsis thaliana]
AT2G22770	3.80E-03	1.04	0.71	2.02	1.08	1.94	1.52	0.78	basic helix-loop-helix (bHLH) family protein contains Pfam profile: PF00010 helix-loop-helix DNA-binding domain
AT3G51440	1.56E-03	0.85	0.79	1.65	1.62	1.94	2.05	1.06	strictosidine synthase family protein similar to hemomucin [Drosophila melanogaster][GI:1280434], strictosidine synthase [Rauvolfia serpentina][SP|P15324]; contains strictosidine synthase domain PF03088
AT2G04400	3.09E-03	0.61	0.86	1.17	1.63	1.94	1.90	0.98	indole-3-glycerol phosphate synthase (IGPS) nearly identical to SP|P49572
AT1G09970	2.53E-03	0.63	0.82	1.21	1.78	1.93	2.18	1.13	leucine-rich repeat transmembrane protein kinase, putative Similar to A. thaliana receptor-like protein kinase (gb|RLK5_ARATH). ESTs gb|ATTS0475,gb|ATTS4362 come from this gene isoform contains a TG acceptor site at intron.
AT1G72360	1.07E-02	0.88	0.90	1.70	1.26	1.93	1.39	0.72	ethylene-responsive element-binding protein, putative contains Pfam profile: PF00847 AP2 domain; similar to ethylene responsive element binding protein (GI:18496063)[Fagus sylvatica]
AT1G75220	6.29E-04	0.77	0.76	1.48	1.45	1.93	1.90	0.99	integral membrane protein, putative strong similarity to integral membrane protein GI:1209756 from [Beta vulgaris]; contains Pfam profile PF00083: major facilitator superfamily protein
AT5G17280	4.78E-02	0.96	0.81	1.84	1.13	1.93	1.39	0.72	expressed protein similar to unknown protein (pir||T41692)
AT4G39030	1.95E-03	0.59	0.81	1.14	1.60	1.93	1.99	1.03	enhanced disease susceptibility 5 (EDS5) / salicylic acid induction deficient 1 (SID1) identical to SP|Q945F0; contains Pfam profile PF01554: Uncharacterized membrane protein family
AT2G20610	3.00E-03	0.62	0.77	1.19	1.32	1.93	1.71	0.89	aminotransferase, putative similar to nicotianamine aminotransferase from Hordeum vulgare [GI:6498122, GI:6469087]; contains Pfam profile PF00155 aminotransferase, classes I and II
AT4G16740	1.49E-03	0.80	0.50	1.54	1.59	1.92	3.17	1.65	terpene synthase/cyclase family protein similar to myrcene/ocimene synthase [GI:9957293]; contains Pfam profile PF01397: Terpene synthase, N-terminal domain; contains Pfam profile PF03936: Terpene synthase family, metal binding domain; identical to cDNA (partial mRNA) E-beta-ocimene synthase GI:3034913[illegible]
AT4G27070	4.62E-04	0.64	0.92	1.23	1.69	1.92	1.85	0.96	tryptophan synthase, beta subunit 2 (TSB2) identical to SP|25269
AT4G08950	5.42E-03	0.80	0.54	1.53	1.08	1.92	2.00	1.04	phosphate-responsive protein, putative (EXO) similar to phi-1 (phosphate-induced gene) [Nicotiana tabacum] GI:3759184; contains Pfam profile PF04674: Phosphate-induced protein 1 conserved region
AT5G18490	5.32E-03	0.68	0.98	1.30	1.75	1.92	1.79	0.94	expressed protein
AT1G03080	2.83E-02	0.84	0.80	1.60	1.46	1.92	1.81	0.95	kinase interacting family protein similar to kinase interacting protein 1 (GI:13936326) [Petunia integrifolia]
AT1G77420	1.34E-04	0.79	0.69	1.52	1.51	1.91	2.19	1.14	hydrolase, alpha/beta fold family protein similar to monoglyceride lipase from [Homo sapiens] GI:14594904, [Mus musculus] GI:2632162; contains Pfam profile PF00561: hydrolase, alpha/beta fold family
AT1G70090	2.88E-03	0.85	0.70	1.62	1.08	1.91	1.54	0.80	glycosyl transferase family 8 protein contains Pfam profile: PF01501 glycosyl transferase family 8
AT2G48030	2.13E-04	0.81	0.76	1.55	1.40	1.91	1.85	0.97	endonuclease/exonuclease/phosphatase family protein contains Pfam profile PF03372: Endonuclease/Exonuclease/phosphatase family
AT4G04840	2.59E-03	0.94	0.78	1.78	1.20	1.90	1.53	0.81	methionine sulfoxide reductase domain-containing protein / SeIR domain-containing protein low similarity to pilin-like transcription factor [Homo sapiens] GI:5059062; contains Pfam profile PF01641: SelR domain
AT4G31500	7.87E-03	0.60	0.90	1.13	1.55	1.89	1.71	0.91	cytochrome P450 83B1 (CYP83B1) identical to Cytochrome P450 (SP:O65782)[Arabidopsis thaliana]
AT5G61520	3.31E-03	0.63	0.95	1.19	1.90	1.89	1.99	1.05	hexose transporter, putative similar to hexose carrier protein hex6 [Ricinus communis] SP|Q07423; contains Pfam profile PF00083: major facilitator superfamily protein
AT5G66200	3.24E-03	0.77	0.77	1.45	1.35	1.89	1.77	0.93	armadillo/beta-catenin repeat family protein contains Pfam profile: PF00514 armadillo/beta-catenin-like repeat
AT1G17060	2.58E-03	0.85	0.80	1.62	1.32	1.89	1.66	0.88	cytochrome P450, putative 41% identical to Cytochrome P450 [Catharanthus roseus] (gi|404690)
AT5G63850	5.65E-03	0.89	0.67	1.67	1.36	1.89	2.05	1.08	amino acid transporter 4, putative (AAP4) identical to amino acid transporter GI:608671 from [Arabidopsis thaliana];
AT4G27570;AT4G	7.21E-03	0.75	0.82	1.41	1.29	1.88	1.57	0.83	[AT4G27570, glycosyltransferase family protein contains Pfam profile: PF00201 UDP-glucoronosyl and UDP-glucosyl transferase];[AT4G27560, glycosyltransferase family protein contains Pfam profile: PF00201 UDP-glucoronosyl and UDP-glucosyl transferase]
AT1G22550	7.78E-05	0.76	0.64	1.43	1.39	1.88	2.18	1.16	proton-dependent oligopeptide transport (POT) family protein contains Pfam profile: PF00854 POT family
AT5G26740	1.40E-03	0.88	0.74	1.65	1.15	1.88	1.55	0.83	expressed protein contains Pfam profile PF03619: Domain of unknown function
AT1G22335	1.09E-02	0.88	0.79	1.66	1.06	1.87	1.34	0.71	expressed protein ; expression supported by MPSS
AT1G06640	4.02E-04	0.78	0.79	1.46	1.37	1.87	1.72	0.92	2-oxoglutarate-dependent dioxygenase, putative similar to 2A6 (GI:599622) and tomato ethylene synthesis regulatory protein E8 (SP|P10967); contains Pfam profile: PF00671 Iron/Ascorbate oxidoreductase family
AT3G50650	1.75E-03	0.72	0.71	1.35	1.59	1.87	2.26	1.21	scarecrow-like transcription factor 7 (SCL7)
AT5G05140	2.85E-02	0.83	0.79	1.55	1.22	1.86	1.55	0.83	transcription elongation factor-related low similarity to transcription elongation factor TFIIS.h [Mus musculus] GI:3288547, elongin A [Mus musculus] GI:6009624
AT3G01930;AT3G	1.12E-04	0.68	0.84	1.26	1.65	1.86	1.96	1.06	[AT3G01930, nodulin family protein similar to nodulin-like protein [Arabidopsis thaliana] GI:3329368, nodule-specific protein Nlj70 [Lotus japonicus] GI:3329366];[AT3G01920, yrdC family protein contains Pfam PF01300: yrdC domain; similar to Protein yciO. (Swiss-Prot:P45847) [Shigella flexneri]; similar to Chain A, [illegible]
AT5G09440	1.06E-03	0.78	0.78	1.46	1.14	1.86	1.46	0.79	phosphate-responsive protein, putative similar to phi-1 (phosphate-induced gene) [Nicotiana tabacum] GI:3759184; contains Pfam profile PF04674: Phosphate-induced protein 1 conserved region
AT1G32350	4.06E-03	0.57	1.01	1.06	1.96	1.85	1.93	1.04	alternative oxidase, putative similar to Alternative oxidase 1a, mitochondrial precursor from Arabidopsis thaliana [SP|Q39219], alternative oxidase 2, mitochondrial precursor from Nicotiana tabacum [SP|Q40578]; contains Pfam profile PF01786 Alternative oxidase
AT5G64260	9.23E-04	0.90	0.76	1.67	1.33	1.85	1.75	0.95	phosphate-responsive protein, putative similar to phi-1 (phosphate-induced gene) [Nicotiana tabacum] GI:3759184; contains Pfam profile PF04674: Phosphate-induced protein 1 conserved region
AT5G11670	3.36E-04	0.59	0.86	1.09	1.40	1.85	1.62	0.88	malate oxidoreductase, putative similar to NADP-dependent malic enzyme (EC 1.1.1.40) (NADP-ME) (SP|P12628) [Phaseolus vulgaris]
AT5G67300;AT5G	1.86E-03	0.75	0.67	1.39	1.15	1.85	1.71	0.93	[AT5G67300, myb family transcription factor contains PFAM profile: myb DNA binding domain PF00249];[AT5G67310, cytochrome P450 family protein]
AT5G13750	1.67E-03	0.84	0.70	1.56	1.47	1.84	2.10	1.14	transporter-related
AT1G11380	8.85E-03	0.83	0.69	1.53	1.22	1.84	1.77	0.96	expressed protein contains Pfam profile PF04749: Protein of unknown function, DUF614
AT4G34200	8.01E-04	0.62	0.81	1.14	2.04	1.84	2.52	1.37	D-3-phosphoglycerate dehydrogenase, putative / 3-PGDH, putative similar to phosphoglycerate dehydrogenase, Arabidopsis thaliana, SP:O04130
AT5G39360	6.19E-04	0.81	0.80	1.48	1.30	1.83	1.62	0.89	circadian clock coupling factor-related similar to circadian clock coupling factor ZGT [Nicotiana tabacum] GI:14210079
ATMG01370	2.04E-02	1.05	0.66	1.91	1.08	1.82	1.64	0.90	orf111d hypothetical protein
AT2G47180	4.67E-02	0.58	1.07	1.06	2.00	1.82	1.87	1.02	galactinol synthase, putative similar to galactinol synthase, isoform GolS-1 GI:5608497 from [Ajuga reptans]
AT1G53760	1.13E-03	0.83	0.76	1.52	1.24	1.82	1.62	0.89	expressed protein
AT1G66370	1.96E-02	0.90	0.83	1.64	1.04	1.82	1.24	0.68	myb family transcription factor (MYB113) contains Pfam profile: PF00249 myb-like DNA-binding domain
AT4G36670	1.31E-02	0.59	0.80	1.07	1.29	1.82	1.60	0.88	mannitol transporter, putative similar to mannitol transporter [Apium graveolens var. dulce] GI:12004316; contains Pfam profile PF00083: major facilitator superfamily protein
AT1G27300	3.72E-02	0.95	0.82	1.72	1.12	1.81	1.37	0.75	expressed protein
AT1G71960	2.06E-03	0.74	0.82	1.33	1.75	1.81	2.14	1.18	ABC transporter family protein similar to breast cancer resistance protein GB:AAC97367 from [Homo sapiens]
AT5G43270	1.62E-03	0.82	0.62	1.49	1.19	1.81	1.91	1.05	squamosa promoter-binding protein-like 2 (SPL2) identical to squamosa promoter binding protein-like 2 [Arabidopsis thaliana] GI:5931645; contains Pfam profile PF03110: SBP domain
AT3G11210	2.23E-03	0.98	0.75	1.76	0.92	1.81	1.23	0.68	GDSL-motif lipase/hydrolase family protein contains Pfam profile PF00657: Lipase/Acylhydrolase with GDSL-like motif
AT5G10520	1.91E-02	0.62	1.09	1.12	2.11	1.81	1.94	1.07	protein kinase family protein contains protein kinase domain, INTERPRO:IPR000719
AT1G70670	3.24E-02	0.91	0.93	1.64	1.24	1.80	1.32	0.74	caleosin-related family protein similar to Ca+2-binding EF hand protein GB:AAB71227 [Glycine max]; contains Pfam profilePF05042: Caleosin related protein
AT1G43650	3.36E-04	0.80	0.79	1.45	1.31	1.80	1.65	0.92	integral membrane family protein / nodulin MtN21-related similar to MtN21 GI:2598575 (root nodule development) from [Medicago truncatula[similar to MtN21 GI:2598575 (root nodule development) from [Medicago truncatula]
AT3G04290	1.08E-03	0.75	0.95	1.36	1.78	1.80	1.87	1.04	GDSL-motif lipase/hydrolase family protein similar to family II lipases EXL3 GI:15054386, EXL1 GI:15054382, EXL2 GI:15054384 from [Arabidopsis thaliana]; contains Pfam profile: lipase/acylhydrolase with GDSL-like motif
AT1G25500	7.75E-04	0.74	0.78	1.33	2.04	1.80	2.62	1.46	choline transporter-related contains weak similarity to CD92 protein [Homo sapiens] gi|16945323|emb|CAC82175
AT5G01850	3.08E-04	0.79	0.80	1.42	1.42	1.79	1.77	0.98	protein kinase, putative similar to protein kinase [Arabidopsis thaliana] gi|1054633|emb|CAA63387; contains protein kinase domain, Pfam:PF00069
AT1G61667	3.03E-02	0.94	0.82	1.69	1.07	1.79	1.32	0.74	expressed protein contains Pfam profile PF04398: Protein of unknown function, DUF538
AT1G78490	2.19E-03	0.92	0.69	1.64	1.26	1.79	1.84	1.03	cytochrome P450 family protein similar to Cytochrome P450 90A1 (SP:Q42569) [Arabidopsis thaliana]
AT3G55290;AT3G	2.21E-02	0.81	0.89	1.44	1.25	1.79	1.40	0.78	[AT3G55290, short-chain dehydrogenase/reductase (SDR) family protein contains similarity to 3-oxoacyl-[acyl-carrier protein] reductase SP:P51831 from [Bacillus subtilis]];[AT3G55310, short-chain dehydrogenase/reductase (SDR) family protein contains similarity to 3-oxoacyl-[acyl-carrier protein] reductase SP:P518[illegible]
AT3G15500	5.23E-03	0.70	0.79	1.24	1.39	1.78	1.75	0.98	no apical meristem (NAM) family protein (NAC3) identical to AtNAC3 [Arabidopsis thaliana] GI:12060424; contains Pfam PF02365: No apical meristem (NAM) domain; similar to jasmonic acid 2 GB:AAF04915 from [Lycopersicon esculentum]
AT2G35730	1.63E-02	0.78	0.89	1.39	1.87	1.78	2.10	1.18	heavy-metal-associated domain-containing protein contains PS1047 Heavy-metal-associated domain contains Pfam profile PF00403: Heavy-metal-associated domain
AT4G25940	1.77E-04	0.66	0.81	1.17	1.48	1.78	1.82	1.03	epsin N-terminal homology (ENTH) domain-containing protein contains Pfam PF01417: ENTH domain. ENTH (Epsin N-terminal homology) domain; similar to Chain B, Crystal Structure Of N-Terminal Domain Of Drosophila Ap180 (GP:13399617) [Drosophila melanogaster]; supporting cDNA gi|20465326|gb|AY096427.[illegible]
AT1G76160	1.86E-02	0.93	0.71	1.65	1.14	1.78	1.61	0.91	multi-copper oxidase type I family protein similar to pollen-specific BP10 protein [SP|Q00624][Brassica napus]; contains Pfam profile: PF00394 Multicopper oxidase
AT4G36900	1.73E-03	0.91	0.85	1.61	1.06	1.77	1.26	0.71	AP2 domain-containing protein RAP2.10 (RAP2.10) identical to GP:2632063 and GP:7270639 [Arabidopsis thaliana]; identical to cDNA TINY-like protein GI:2632062
AT1G22530	9.10E-04	0.83	0.64	1.46	1.32	1.77	2.05	1.16	SEC14 cytosolic factor family protein / phosphoglyceride transfer family protein contains Pfam PF00650 : CRAL/TRIO domain; contains Pfam PF03765 : CRAL/TRIO, N-terminus; similar to SEC14-like protein 2 (Alpha-tocopherol associated protein) (TAP) (bTAP) (Fragment) (SP:P58875) {Bos taurus}
AT1G08940	4.22E-02	0.74	1.05	1.31	1.33	1.77	1.27	0.72	phosphoglycerate/bisphosphoglycerate mutase family protein contains Pfam profile PF00300: phosphoglycerate mutase family
AT2G26440	1.02E-02	0.53	1.12	0.93	2.19	1.77	1.95	1.10	pectinesterase family protein contains Pfam profile: PF01095 pectinesterase
AT5G61780	4.04E-03	0.73	0.62	1.29	1.43	1.77	2.30	1.30	tudor domain-containing protein / nuclease family protein contains Pfam domains PF00567: Tudor domain and PF00565: Staphylococcal nuclease homologue
AT1G61610	1.32E-02	0.76	0.85	1.34	2.01	1.76	2.35	1.33	S-locus lectin protein kinase family protein similar to KI domain interacting kinase 1 [Zea mays] gi|2735017|gb|AAB93834; contains S-locus glycoprotein family domain, Pfam:PF00954
AT3G19970	3.70E-02	0.77	0.75	1.36	1.33	1.76	1.78	1.01	expressed protein
AT1G71100	2.14E-03	0.70	0.79	1.22	1.24	1.76	1.57	0.89	ribose 5-phosphate isomerase-related similar to ribose-5-phosphate isomerase GI:18654317 from [Spinacia oleracea]
AT4G13340	1.73E-03	0.72	0.63	1.27	1.34	1.76	2.14	1.22	leucine-rich repeat family protein / extensin family protein similar to extensin-like protein [Lycopersicon esculentum] gi|5917664|gb|AAD55979; contains leucine-rich repeats, Pfam:PF00560; contains proline rich extensin domains, INTERPRO:IPR002965
AT3G03480	3.36E-02	0.82	0.82	1.43	2.37	1.76	2.88	1.64	transferase family protein similar to hypersensitivity-related gene GB:CAA64636 [Nicotiana tabacum]; contains Pfam transferase family domain PF00248
AT3G25570	1.45E-03	0.84	0.73	1.47	1.70	1.76	2.34	1.33	adenosylmethionine decarboxylase family protein contains Pfam profile: PF01536 adenosylmethionine decarboxylase
AT1G19570	6.27E-04	0.82	0.86	1.44	1.42	1.75	1.66	0.95	dehydroascorbate reductase, putative similar to GB:BAA90672 from [Oryza sativa]
AT5G17990	1.66E-04	0.66	0.78	1.15	1.71	1.75	2.20	1.25	anthranilate phosphoribosyltransferase identical to anthranilate phosphoribosyltransferase, chloroplast precursor (EC 2.4.2.18) SP:Q02166 from [Arabidopsis thaliana]
AT3G20410	1.70E-02	0.71	0.87	1.24	1.35	1.75	1.56	0.89	calmodulin-domain protein kinase isoform 9 (CPK9) identical to calmodulin-domain protein kinase CDPK isoform 9 [Arabidopsis thaliana] gi|1399265|gb|AAB03242
AT1G15670	2.52E-05	0.68	0.81	1.20	1.47	1.75	1.81	1.03	kelch repeat-containing F-box family protein similar to SP|Q9ER30 Kelch-related protein 1 (Sarcosin) {Rattus norvegicus}; contains Pfam profiles PF01344: Kelch motif, PF00646: F-box domain
AT5G42860	2.07E-02	0.86	0.69	1.50	1.04	1.75	1.50	0.86	expressed protein
AT3G59760	7.52E-05	0.72	0.81	1.25	1.28	1.75	1.58	0.91	cysteine synthase, mitochondrial, putative / O-acetylserine (thiol)-lyase, putative / O-acetylserine sulfhydrylase, putative similar to SP|Q43725 Cysteine synthase, mitochondrial precursor (EC 4.2.99.8) (O- acetylserine sulfhydrylase) (O-acetylserine (Thiol)-lyase) (Arabidopsis thaliana)
AT3G48350	6.55E-03	0.84	0.82	1.47	1.36	1.74	1.66	0.95	cysteine proteinase, putative similar to cysteine endopeptidase precursor [Ricinus communis] GI:2944446; contains Pfam profile PF00112: Papain family cysteine protease
AT5G23490;AT5G	3.09E-03	0.63	1.04	1.10	1.54	1.74	1.48	0.85	[AT5G23490, expressed protein];[AT5G23510, expressed protein]
AT5G12880	4.51E-02	0.84	0.84	1.46	1.04	1.74	1.24	0.71	proline-rich family protein similar to hydroxyproline-rich glycoprotein gi|169345|gb|AAA33763
AT3G15790	6.80E-04	0.70	0.87	1.21	1.41	1.74	1.62	0.93	methyl-CpG-binding domain-containing protein contains Pfam profile PF01429: Methyl-CpG binding domain
AT2G45680	1.41E-04	0.74	0.81	1.28	1.46	1.74	1.80	1.04	TCP family transcription factor, putative similar to PCF2 (GI:2580440) [Oryza sativa]

AT1G59950	2.13E-02	0.78	0.84	1.35	3.46	1.74	4.12	2.37	aldo/keto reductase, putative similar to NADPH-dependent codeinone reductase GI:6478210 [Papaver somniferum], NAD(P)H dependent 6'-deoxychalcone synthase [Glycine max][GI:18728]
AT4G17070	1.90E-03	0.62	0.86	1.08	1.19	1.73	1.37	0.79	expressed protein
AT2G43590	1.07E-03	0.51	1.12	0.88	3.12	1.73	2.79	1.61	chitinase, putative similar to basic endochitinase CHB4 precursor SP:Q06209 from [Brassica napus]
AT3G56930	1.49E-03	0.89	0.84	1.54	1.56	1.73	1.87	1.08	zinc finger (DHHC type) family protein low similarity to Golgi-specific DHHC zinc figer protein [Mus musculus] GI:21728103; contains Pfam profile PF01529: DHHC zinc finger domain
AT1G18500	1.65E-03	0.77	0.78	1.32	1.31	1.73	1.69	0.98	2-isopropylmalate synthase, putative strong similarity to 2-isopropylmalate synthase (IMS1) [Arabidopsis thaliana] GI:12330687; contains Pfam profile PF00682: HMGL-like
AT4G26910	7.21E-03	0.73	0.89	1.25	1.39	1.72	1.56	0.91	2-oxoacid dehydrogenase family protein similar to SP|P36957 Dihydrolipoamide succinyltransferase component of 2-oxoglutarate dehydrogenase complex, mitochondrial precursor (EC 2.3.1.61) (Homo sapiens); contains Pfam profiles PF00198: 2-oxo acid dehydrogenases acyltransferase (catalytic domain), PF00364: Biotin-requiring enzyme
AT1G28050	5.42E-03	0.90	0.69	1.55	1.15	1.72	1.66	0.96	zinc finger (B-box type) family protein
AT3G06500	1.86E-02	0.69	0.69	1.19	1.32	1.72	1.92	1.12	beta-fructofuranosidase, putative / invertase, putative / saccharase, putative / beta-fructosidase, putative similar to neutral invertase [Daucus carota] GI:4200165; contains Pfam profile PF04853: Plant neutral invertase
AT5G54380	5.23E-03	0.91	0.62	1.56	1.35	1.72	2.17	1.26	protein kinase family protein contains protein kinase domain, Pfam:PF00069
AT3G23560	5.19E-04	0.68	0.90	1.17	1.58	1.72	1.76	1.02	MATE efflux family protein similar to ripening regulated protein DDTFR18 [Lycopersicon esculentum] GI:12231296; contains Pfam profile: PF01554 uncharacterized membrane protein family
AT3G44720	1.67E-03	0.63	1.02	1.08	2.06	1.72	2.02	1.18	prephenate dehydratase family protein similar to bacterial PheA gene products
AT1G33030	1.90E-02	0.61	0.97	1.06	1.54	1.72	1.59	0.92	O-methyltransferase family 2 protein similar to caffeic acid 3-O-methyltransferase [SP|Q00763] [Populus tremuloides], catechol O-methyltransferase [GI:4808524][Thalictrum tuberosum]
AT5G21105	1.07E-02	0.92	0.87	1.57	1.68	1.72	1.92	1.12	L-ascorbate oxidase, putative similar to L-ascorbate oxidase from [Nicotiana tabacum] SP|Q40588, (Cucurbita pepo var. melopepo) SP|P37064; contains Pfam profile PF00394: Multicopper oxidase; supported by cDNA gi_15215753_gb_AY050406.1_; A false intron was added between exons 4 and 5 to circumvent the single nucleotide insertion in this BAC which, otherwise, causes a frameshift.
AT1G68600	3.88E-02	0.83	0.90	1.41	1.50	1.71	1.67	0.98	expressed protein contains Pfam profile PF01027: Uncharacterized protein family UPF0005
AT3G20660	1.27E-02	0.66	0.83	1.12	1.23	1.71	1.47	0.86	organic cation transporter family protein similar to organic cation transporter OCT3 [Rattus norvegicus] GI:3273722, organic cation transporter 3 [Mus musculus] GI:4454795; contains Pfam profile PF00083: major facilitator superfamily protein
AT4G30850	1.31E-02	0.83	0.94	1.42	1.51	1.70	1.61	0.95	expressed protein contains Pfam domain, PF03006: Uncharacterised protein family (Hly-III / UPF0073)
AT1G66760	2.88E-03	0.93	0.82	1.59	1.45	1.70	1.78	1.05	MATE efflux family protein contains TIGRfam profile: TIGR00797: MATE efflux family protein, Pfam profile PF01554: Uncharacterized membrane protein family
AT1G58200	5.46E-04	0.83	0.74	1.41	1.57	1.70	2.11	1.24	mechanosensitive ion channel domain-containing protein / MS ion channel domain-containing protein contains Pfam profile PF00924: Mechanosensitive ion channel
AT1G20090	2.81E-04	0.90	0.76	1.52	1.19	1.70	1.57	0.92	Rac-like GTP-binding protein (ARAC4) / Rho-like GTP-binding protein (ROP2) identical to SP:Q38919 RAC-like GTP binding protein ARAC4 (GTP binding protein ROP2) [Arabidopsis thaliana], Rho-like GTP binding protein [Arabidopsis thaliana] GI:2654009
AT1G53160	2.49E-02	0.66	1.05	1.12	1.28	1.70	1.22	0.72	squamosa promoter-binding protein-like 4 (SPL4) nearly identical to squamosa promoter binding protein-like 4 [Arabidopsis thaliana] GI:5931657; contains Pfam profile PF03110: SBP domain
AT4G24830	1.23E-04	0.81	0.79	1.37	1.58	1.70	2.00	1.18	arginosuccinate synthase family contains Pfam profile: PF00764 arginosuccinate synthase
AT2G17230	9.00E-03	0.87	0.60	1.48	0.99	1.70	1.66	0.98	phosphate-responsive 1 family protein similar to phi-1 (phosphate-induced gene) [Nicotiana tabacum] GI:3759184; contains Pfam profile PF04674: Phosphate-induced protein 1 conserved region
AT3G09490	1.10E-03	0.77	0.82	1.31	1.07	1.70	1.31	0.77	chloroplast lumen common family protein 2 TPR domains; similar to chloroplast lumen proteins [GI:4056493 (F3G5.19)(At2g37400)] and [GI:7413648 (T22P11.180),(At5g02590)] [Arabidopsis thaliana]; +
AT3G13110	2.39E-03	0.65	0.99	1.10	1.36	1.70	1.38	0.81	serine O-acetyltransferase (SAT-1) identical to serine acetyltransferase (Sat-1) GI:1184048 [Arabidopsis thaliana]
AT3G46280	5.64E-03	0.94	0.74	1.60	1.21	1.70	1.63	0.96	protein kinase-related contains similarity to light repressible receptor protein kinase [Arabidopsis thaliana] gi|1321686|emb|CAA66376
AT1G05620	1.59E-04	0.86	0.77	1.45	1.32	1.70	1.71	1.01	inosine-uridine preferring nucleoside hydrolase family protein similar to Chain A, Crystal Structure Of Nucleoside Hydrolase From Leishmania MajorGI:8569431; contains Pfam profile PF01156: Inosine-uridine preferring nucleoside hydrolase
AT3G46660	3.17E-02	0.94	0.81	1.60	1.07	1.69	1.32	0.78	UDP-glucoronosyl/UDP-glucosyl transferase family protein contains Pfam profile: PF00201 UDP-glucoronosyl and UDP-glucosyl transferase
AT4G15530	1.59E-02	0.76	0.83	1.29	1.44	1.69	1.73	1.02	pyruvate phosphate dikinase family protein contains Pfam profiles: PF01326 pyruvate phosphate dikinase, PEP/pyruvate binding domain, PF02896 PEP-utilizing enzyme, TIM barrel domain
AT5G56760	2.93E-05	0.77	0.79	1.30	1.33	1.69	1.69	1.00	serine O-acetyltransferase (SAT-52) identical to GI:905391
AT1G14330	8.16E-03	0.83	0.74	1.40	1.22	1.69	1.64	0.97	kelch repeat-containing F-box family protein contains Pfam profile PF01344: Kelch motif; contains weak Pfam PF00646: F-box domain; weak similarity to Kelch-like protein 1 (Swiss-Prot:Q9NR64) [Homo sapiens]
AT1G74950	9.00E-03	0.75	0.61	1.27	1.13	1.69	1.86	1.10	expressed protein
AT1G72180	3.55E-04	0.78	0.75	1.31	1.83	1.69	2.46	1.46	leucine-rich repeat transmembrane protein kinase, putative similar to GI:3641252 from [Malus x domestica] (Plant Mol. Biol. 40 (6), 945-957 (1999))
AT2G47440	3.21E-02	0.67	0.93	1.13	1.42	1.68	1.51	0.90	DNAJ heat shock N-terminal domain-containing protein contains Pfam profiles PF00226: DnaJ domain, PF00515: TPR Domain; similar to GP|2104534|AF001308 (T10M13.11)
AT4G26690	3.53E-02	0.79	0.75	1.32	1.32	1.68	1.76	1.04	glycerophosphoryl diester phosphodiesterase family protein weak similarity to glycerophosphodiester phosphodiesterase [Borrelia hermsii] GI:1399038; contains Pfam profile PF03009: Glycerophosphoryl diester phosphodiesterase family
AT3G26200	6.31E-03	0.72	0.87	1.20	1.55	1.68	1.78	1.06	cytochrome P450 71B22, putative (CYP71B22) identical to cytochrome P450 71B22 (SP:Q9LTM1)[Arabidopsis thaliana];contains Pfam profile: PF00067 cytochrome P450
AT5G04590	1.49E-03	0.69	0.84	1.16	1.59	1.68	1.89	1.13	sulfite reductase / ferredoxin (SIR) identical to sulfite reductase [Arabidopsis thaliana] GI:804953, GI:2584721
AT5G07440	8.54E-04	0.93	0.70	1.55	1.17	1.68	1.68	1.00	glutamate dehydrogenase 2 (GDH2) identical to glutamate dehydrogenase 2 (GDH 2) [Arabidopsis thaliana] SWISS-PROT:Q38946
AT3G58710	1.75E-03	0.82	0.85	1.37	1.34	1.67	1.58	0.94	WRKY family transcription factor contains Pfam profile: PF03106 WRKY DNA -binding domain
AT1G69370	2.08E-02	0.90	0.80	1.50	1.28	1.67	1.59	0.95	chorismate mutase, putative similar to gi:5732016 and SP|P42738; contains Pfam profile: PF01817: Chorismate mutase
AT4G08500;AT4G	8.90E-03	0.80	0.83	1.33	1.24	1.67	1.49	0.89	[AT4G08500, mitogen-activated protein kinase kinase, putative similar to mitogen-activated protein kinase MEKK1 GP|1255448 [Arabidopsis thaliana]];[AT4G08470, mitogen-activated protein kinase, putative similar to mitogen-activated protein kinase [Arabidopsis thaliana] gi|1255448|dbj|BAA09057; contains Pfam PF00069: Protein kinase domain]
AT4G18140	3.74E-03	0.90	0.84	1.50	1.39	1.67	1.66	0.99	NLI interacting factor (NIF) family protein contains Pfam profile PF03031: NLI interacting factor
AT1G30530	9.48E-03	0.78	0.97	1.31	1.17	1.67	1.21	0.73	UDP-glucoronosyl/UDP-glucosyl transferase family protein contains Pfam profile: PF00201 UDP-glucoronosyl and UDP-glucosyl transferase
AT2G30990	4.70E-02	0.81	0.88	1.35	1.22	1.67	1.40	0.84	expressed protein contains Pfam profile: PF05097 protein of unknown function (DUF688)
AT2G30750	3.06E-03	0.67	0.83	1.12	1.31	1.66	1.58	0.95	cytochrome P450 71A12, putative (CYP71A12) identical to Cytochrome P450 (SP:O49340) [Arabidopsis thaliana]; contains Pfam profile: PF00067 cytochrome P450
AT5G13110	9.74E-03	0.70	0.94	1.17	1.41	1.66	1.50	0.90	glucose-6-phosphate 1-dehydrogenase, putative / G6PD, putative similar to SP|Q43839 Glucose-6-phosphate 1-dehydrogenase, chloroplast precursor (EC 1.1.1.49) (G6PD) [Solanum tuberosum]; contains Pfam profiles PF02781: Glucose-6-phosphate dehydrogenase C-terminal, PF00479: Glucose-6-phosphate dehydrogenase NAD binding domain
AT1G61560	1.94E-02	0.73	1.05	1.21	1.93	1.66	1.84	1.10	seven transmembrane MLO family protein / MLO-like protein 6 (MLO6) identical to membrane protein Mlo6 [Arabidopsis thaliana] gi|14091582|gb|AAK53799; similar to MLO protein SWISS-PROT:P93766, NCBI_gi:1877221 [Hordeum vulgare][Barley]; contains Pfam profile PF03094: Mlo family
AT1G10020	2.88E-03	0.77	0.67	1.27	1.26	1.66	1.88	1.13	expressed protein
AT1G50460	1.25E-02	0.66	0.96	1.10	1.11	1.66	1.16	0.70	hexokinase, putative similar to hexokinase 1 [Spinacia oleracea] Swiss-Prot:Q9SEK3
AT2G23850	5.07E-04	0.71	0.88	1.18	1.74	1.66	1.97	1.19	expressed protein
AT1G59870	2.15E-03	0.69	0.91	1.14	1.35	1.66	1.48	0.89	ABC transporter family protein similar to PDR5-like ABC transporter GI:1514643 from [Spirodela polyrhiza]
AT2G45280	1.30E-03	0.57	1.25	0.95	1.74	1.66	1.39	0.84	DNA repair family protein contains similarity to Swiss-Prot:O43502 DNA repair protein RAD51 homolog 3 [Homo sapiens]
AT1G54030	4.19E-04	0.75	0.85	1.24	1.32	1.66	1.55	0.93	GDSL-motif lipase, putative similar to myrosinase-associated proteins GI:1769968, GI:1769970, GI:1216391, GI:1216389 from [Brassica napus]; contains Pfam profile PF00657: GDSL-like Lipase/Acylhydrolase
AT4G24000	1.63E-02	0.85	0.86	1.42	1.73	1.66	2.00	1.21	cellulose synthase family protein similar to cellulose synthase from Gossypium hirsutum [gi:1706956], cellulose synthase-5 from Zea mays [gi:9622882]
AT2G28130	2.39E-03	0.81	0.80	1.35	1.63	1.66	2.03	1.23	expressed protein
AT4G25030	1.82E-02	0.75	0.93	1.25	1.36	1.65	1.47	0.89	expressed protein
AT2G27380	1.40E-03	0.76	0.80	1.25	1.61	1.65	2.01	1.22	glutathione synthetase (GSH2) non-consensus AT donor splice site at exon 6, AC acceptor splice site at exon 7; identical to Swiss-Prot:P46416 glutathione synthetase, chloroplast precursor (Glutathione synthase) (GSH synthetase) (GSH-S) [Arabidopsis thaliana]
AT5G53360	6.04E-04	0.84	0.77	1.38	1.20	1.65	1.55	0.94	seven in absentia (SINA) family protein low similarity to siah-1A protein [Mus musculus] GI:297035; contains Pfam profile PF03145: Seven in absentia protein family
AT4G16250	9.00E-03	0.82	0.88	1.36	1.56	1.65	1.77	1.07	phytochrome D (PHYD) nearly identical to SP|P42497 Phytochrome D (Arabidopsis thaliana)
AT1G67230	1.70E-02	0.89	0.87	1.47	1.49	1.65	1.70	1.03	expressed protein
AT3G16500	8.16E-03	0.82	0.87	1.35	1.27	1.65	1.46	0.89	auxin-responsive AUX/IAA family protein similar to SP|O24408|AXI_ARATH Auxin-responsive protein IAA18 (Indoleacetic acid-induced protein 18) [Arabidopsis thaliana]; contains Pfam profile: PF02309: AUX/IAA family
AT1G78860	4.11E-02	0.67	0.96	1.10	1.32	1.65	1.38	0.84	curculin-like (mannose-binding) lectin family protein low similarity to Ser/Thr protein kinase [Zea mays] GI:2598067; contains Pfam profile PF01453: Lectin (probable mannose binding)
AT3G26840	2.21E-03	0.83	0.91	1.37	1.31	1.65	1.44	0.87	esterase/lipase/thioesterase family protein contains Pfam profile PF03096: Ndr family
AT1G18270	3.25E-02	0.96	0.80	1.58	1.20	1.65	1.50	0.91	ketose-bisphosphate aldolase class-II family protein low similarity to KbaY (tagatose-1,6-bisphosphate aldolase) [Escherichia coli] GI:8895753; contains Pfam profile PF01116: Fructose-bisphosphate aldolase class-II
AT5G27101	2.28E-02	1.01	0.89	1.65	1.20	1.64	1.36	0.82	cytochrome c oxidase-related similar to SP|P00428 Cytochrome c oxidase polypeptide Vb (EC 1.9.3.1) (VI) [Bovine] [Bos taurus]
AT1G78460	3.55E-03	0.68	0.94	1.11	1.22	1.64	1.31	0.80	SOUL heme-binding family protein weak similarity to SOUL protein [Mus musculus] GI:4886906; contains Pfam profile PF04832: SOUL heme-binding protein
AT1G07240	2.03E-03	0.77	0.88	1.26	1.24	1.64	1.42	0.86	UDP-glucoronosyl/UDP-glucosyl transferase family protein contains Pfam profile: PF00201 UDP-glucoronosyl and UDP-glucosyl transferase
AT5G02875	1.67E-03	0.93	0.81	1.53	1.28	1.64	1.58	0.96	IAA-amino acid hydrolase 1 (ILR1) identical to IAA-amino acid hydrolase 1 (ILR1) [Arabidopsis thaliana] SWISS-PROT:P54968
AT2G26750;AT2G	8.61E-05	0.87	0.86	1.44	1.21	1.64	1.40	0.85	[AT2G26750, epoxide hydrolase, putative strong similarity to AtsEH [Arabidopsis thaliana] GI:1109600];[AT2G26740, epoxide hydrolase, soluble (sEH) identical to AtsEH [Arabidopsis thaliana] GI:1109600]
AT2G17720	5.08E-04	0.70	0.90	1.15	1.30	1.64	1.45	0.88	oxidoreductase, 2OG-Fe(II) oxygenase family protein similar to prolyl 4-hydroxylase, alpha subunit, from Gallus gallus [GI:212530], Rattus norvegicus [GI:474940], Mus musculus [SP|Q60715]; contains PF03171 2OG-Fe(II) oxygenase superfamily domain
AT1G72140	1.41E-02	0.91	0.90	1.50	1.05	1.64	1.16	0.71	proton-dependent oligopeptide transport (POT) family protein contains Pfam profile: PF00854 POT family
AT5G67500	3.30E-04	0.91	0.77	1.49	1.24	1.64	1.61	0.98	porin, putative similar to SP|P42055 34 kDa outer mitochondrial membrane protein porin (Voltage-dependent anion-selective channel protein) (VDAC) [Solanum tuberosum]; contains Pfam profile PF01459: Eukaryotic porin
AT5G43745;AT5G	3.57E-03	0.83	0.84	1.36	1.61	1.64	1.91	1.16	[AT5G43745, phosphotransferase-related similar to streptomycin-3''-phosphotransferase [Streptomyces griseus] GI:153164];[AT5G02940, expressed protein]
AT2G25430	6.27E-03	0.91	0.79	1.49	1.32	1.63	1.68	1.03	epsin N-terminal homology (ENTH) domain-containing protein contains Pfam PF01417: ENTH domain. ENTH (Epsin N-terminal homology) domain;
AT2G37620;AT3G	4.31E-04	0.69	1.00	1.12	1.70	1.63	1.70	1.04	[AT2G37620, actin 1 (ACT1) identical to SP|P10671 Actin 1 (Actin 3) [Arabidopsis thaliana]];[AT3G53750, actin 3 (ACT3) identical to SP|P53493 Actin 3 [Arabidopsis thaliana]; supported by full-length cDNA: Ceres: 19581.]
AT5G55920	4.27E-02	0.84	0.85	1.37	1.08	1.63	1.27	0.78	nucleolar protein, putative similar to SP|P46087 Proliferating-cell nucleolar antigen p120 (Proliferation-associated nucleolar protein p120) [Homo sapiens], SP|P40991 Nucleolar protein NOP2 [Saccharomyces cerevisiae]; contains Pfam profile PF01189: NOL1/NOP2/sun family
AT5G39790	2.37E-02	0.96	0.88	1.56	1.09	1.63	1.24	0.76	5'-AMP-activated protein kinase beta-1 subunit-related contains similarity to Swiss-Prot:P80387 5'-AMP-activated protein kinase, beta-1 subunit (AMPK beta-1 chain) (AMPKb) (40 kDa subunit) [Sus scrofa]
AT5G65630	2.92E-02	0.90	0.80	1.47	1.37	1.63	1.71	1.05	DNA-binding bromodomain-containing protein similar to 5.9 kb fsh membrane protein [Drosophila melanogaster] GI:157455; contains Pfam profile PF00439: Bromodomain
AT4G39270	1.66E-02	0.84	0.95	1.37	2.04	1.63	2.15	1.32	leucine-rich repeat transmembrane protein kinase, putative receptor protein kinase erecta, Arabidopsis thaliana
AT3G17860	2.04E-03	0.86	0.78	1.40	1.17	1.63	1.49	0.92	expressed protein
AT1G08230	1.05E-02	0.75	0.71	1.22	1.27	1.62	1.79	1.11	amino acid transporter family protein low similarity to amino acid permease [Oryza sativa] GI:7415521; contains Pfam profile PF01490: Transmembrane amino acid transporter protein
AT5G57630	7.73E-03	0.86	0.88	1.39	1.25	1.62	1.42	0.87	CBL-interacting protein kinase 21, putative (CIPK21) identical to CBL-interacting protein kinase 21 [Arabidopsis thaliana] gi|14334390|gb|AAK59696
AT1G74650	1.31E-02	0.82	0.90	1.32	1.36	1.62	1.51	0.93	myb family transcription factor (cY13) similar to myb protein cY13 GI:928930 from [Arabidopsis thaliana]; contains Pfam profile: PF00249 myb DNA-binding domain; identical to cDNA cY13 gene GI:928929
AT5G18860	8.99E-03	0.84	0.82	1.36	1.36	1.62	1.65	1.02	inosine-uridine preferring nucleoside hydrolase family protein contains Pfam profile PF01156: Inosine-uridine preferring nucleoside hydrolase
AT5G26600	1.34E-02	0.84	0.90	1.37	1.22	1.62	1.35	0.83	expressed protein weak similarity to SP|P18549 Isopenicillin N epimerase (EC 5.-.-.-) [Streptomyces clavuligerus]
AT1G21440	2.85E-03	0.84	0.85	1.36	1.43	1.62	1.68	1.04	mutase family protein similar to carboxyvinyl-carboxyphosphonate phosphorylmutase GB:O49290 from [Arabidopsis thaliana]; similar to carboxyphosphonoenolpyruvate mutase (GI:47149) [Streptomyces hygroscopicus]; contains Prosite PS00161: Isocitrate lyase signature
AT4G30060	4.27E-02	0.69	0.91	1.11	1.33	1.62	1.45	0.90	expressed protein contains Pfam profile PF03267: Arabidopsis protein of unknown function, DUF266
AT3G48580	7.98E-03	0.90	0.82	1.44	1.07	1.61	1.31	0.81	xyloglucan:xyloglucosyl transferase, putative / xyloglucan endotransglycosylase, putative / endo-xyloglucan transferase, putative similar to endoxyloglucan transferase EXGT-A4 GI:5139002 from [Arabidopsis thaliana]
AT3G02570	8.44E-05	0.90	0.67	1.44	1.22	1.61	1.83	1.14	phosphomannose isomerase type I family protein contains Pfam profile: PF01238 phosphomannose isomerase type I
AT5G56750	1.17E-04	0.86	0.67	1.39	1.16	1.61	1.73	1.07	Ndr family protein similar to SP|O23969 Pollen specific protein SF21 [Helianthus annuus]; contains Pfam profile PF03096: Ndr family
AT2G28600	1.83E-02	0.87	0.88	1.40	1.15	1.61	1.31	0.81	expressed protein
AT3G25180	2.74E-02	0.94	0.82	1.51	1.13	1.61	1.38	0.86	cytochrome P450 family protein similar to cytochrome P450 monooxygenase GB:AAC49188 [Pisum sativum]; contains Pfam profile: PF00067 cytochrome P450
AT2G34770	4.78E-03	0.67	0.93	1.08	1.19	1.61	1.28	0.80	fatty acid hydroxylase (FAH1) identical to fatty acid hydroxylase Fah1p GB:AF021804 GI:2736147 from [Arabidopsis thaliana]
AT5G58670	1.39E-03	0.76	0.75	1.22	1.30	1.61	1.74	1.08	phosphoinositide-specific phospholipase C (PLC1) identical to phosphoinositide specific phospholipase C [Arabidopsis thaliana] GI:902923
AT4G00330	1.01E-02	0.79	0.89	1.28	1.18	1.61	1.32	0.82	protein kinase family protein contains protein kinase domain, Pfam:PF00069
AT3G09410	1.18E-02	0.55	1.17	0.88	2.45	1.61	2.10	1.31	pectinacetylesterase family protein similar to pectinacetylesterase precursor GB:CAA67728 [Vigna radiata]; contains Pfam profile: PF03283 pectinacetylesterase
AT3G01720	1.86E-03	0.77	0.81	1.23	1.48	1.61	1.83	1.14	expressed protein
AT1G02816	2.98E-02	0.95	0.71	1.52	0.99	1.60	1.38	0.86	expressed protein contains Pfam profile PF04398: Protein of unknown function, DUF538
AT5G62860	3.68E-02	0.88	0.83	1.41	1.09	1.60	1.32	0.82	F-box family protein-related contains TIGRFAM TIGR01640: F-box protein interaction domain
AT2G30870;AT2G	6.98E-04	0.72	0.86	1.15	1.12	1.60	1.31	0.82	[AT2G30870, glutathione S-transferase, putative supported by cDNA GI:443698 GB:D17673];[AT2G30880, pleckstrin homology (PH) domain-containing protein contains Pfam profile PF00169: PH domain]
AT4G18430	6.44E-03	0.81	0.87	1.30	1.20	1.60	1.38	0.86	Ras-related GTP-binding protein, putative similar to GTP-binding protein GI:303742 from [Pisum sativum]
AT2G22010	1.99E-02	0.85	0.80	1.37	1.27	1.60	1.58	0.98	zinc finger (C3HC4-type RING finger) family protein contains Pfam profile: PF00097 zinc finger, C3HC4 type (RING finger)
AT1G52580	7.20E-03	0.89	0.86	1.43	1.13	1.60	1.31	0.82	rhomboid family protein contains PFAM domain PF01694, Rhomboid family
AT1G22100	1.70E-02	0.94	0.85	1.51	1.00	1.60	1.18	0.73	glycosyl hydrolase family 1 protein contains Pfam PF00232 : Glycosyl hydrolase family 1 domain; similar to hydroxyisourate hydrolase (GI:19569603) [Glycine max]; furostanol glycoside 26-O-beta-glucosidase F26G,Costus speciosus, PATCHX:S78099
AT4G28910	4.02E-04	0.86	0.87	1.38	1.31	1.60	1.51	0.94	expressed protein
AT4G23100	1.13E-03	0.83	0.79	1.33	1.48	1.60	1.88	1.17	glutamate-cysteine ligase / gamma-glutamylcysteine synthetase (GSH1) identical to glutamate-cysteine ligase SP:P46309 from [Arabidopsis thaliana] ; contains Pfam profile: PF04107 glutamate-cysteine ligase family 2(GCS2)
AT2G40330	7.78E-05	0.55	1.10	0.89	1.76	1.60	1.61	1.00	Bet v I allergen family protein contains Pfam profile PF00407: Pathogenesis-related protein Bet v I family
AT1G72750	2.09E-02	0.96	0.87	1.54	1.03	1.60	1.18	0.74	mitochondrial import inner membrane translocase subunit Tim17/Tim22/Tim23 family protein contains Pfam PF02466: Mitochondrial import inner membrane translocase subunit Tim17
AT5G01050;AT5G	4.88E-02	0.94	0.88	1.50	1.27	1.60	1.44	0.90	[AT5G01050, laccase family protein / diphenol oxidase family protein similar to laccase [Pinus taeda][GI:13661201], lac110 laccase, Populus trichocarpa, EMBL:PTY13773];[AT5G01040, laccase family protein / diphenol oxidase family protein similar to laccase [Pinus taeda][GI:13661201], lac110 laccase, Populus trichocarpa, EMBL:PTY13773]
AT1G57680	2.24E-03	0.76	0.70	1.22	1.29	1.60	1.84	1.15	expressed protein
AT5G23210	2.19E-03	0.79	0.86	1.26	1.52	1.59	1.77	1.11	serine carboxypeptidase S10 family protein similar to Serine carboxypeptidase II chains A and B (SP:P08819) (EC 3.4.16.6) [Triticum aestivum (Wheat)];
AT1G52380	2.54E-03	0.84	0.89	1.34	1.15	1.59	1.29	0.81	Ran-binding protein 1 domain-containing protein / RanBP1 domain-containing protein weak similarity to SP|Q09717 Ran-specific GTPase-activating protein 1 (Ran binding protein 1) (RANBP1) (Spi1-binding protein) [Schizosaccharomyces pombe]; contains Pfam profile PF00638: RanBP1 domain
AT4G08170	4.85E-03	0.90	0.61	1.43	1.06	1.59	1.72	1.08	inositol 1,3,4-trisphosphate 5/6-kinase family protein similar to inositol phosphate kinase (GI:27549256) [Zea mays]; similar to inositol 1,3,4-trisphosphate 5/6-kinase (GI:3396079) [Arabidopsis thaliana]
AT3G44320	7.99E-04	0.99	0.90	1.58	1.24	1.59	1.38	0.87	nitrilase 3 (NIT3) identical to SP|P46010 Nitrilase 3 (EC 3.5.5.1) (Arabidopsis thaliana)
AT5G51070	4.19E-03	0.88	0.73	1.39	1.66	1.59	2.28	1.43	ATP-dependent Clp protease ATP-binding subunit (ClpD), (ERD1) SAG15/ERD1 identical to ERD1 protein GI:497629, SP:P42762 from [Arabidopsis thaliana]; contains Pfam profile PF02861: Clp amino terminal domain
AT1G01340	4.71E-02	0.82	0.99	1.30	1.63	1.59	1.64	1.03	cyclic nucleotide-regulated ion channel (CNGC10) (ACBK1) almost identical to CaM-regulated potassium ion channel (ACBK1) GI:8515883 from [Arabidopsis thaliana]; contains Pfam domain, PF00520: Ion transport protein
AT5G06110	2.90E-02	0.93	0.92	1.49	1.43	1.59	1.55	0.98	DNAJ heat shock N-terminal domain-containing protein / cell division protein-related similar to GlsA [Volvox carteri f. nagariensis] GI:4633129; contains Pfam profiles PF00226 DnaJ domain, PF00249 Myb-like DNA-binding domain
AT1G76640	2.58E-03	0.96	0.96	1.53	1.19	1.59	1.25	0.78	calmodulin-related protein, putative similar to regulator of gene silencing calmodulin-related protein GI:12963415 from [Nicotiana tabacum]
AT5G47560	1.32E-02	0.89	0.81	1.40	1.34	1.58	1.65	1.04	sodium/dicarboxylate cotransporter, putative similar to SWISS-PROT:Q13183 renal sodium/dicarboxylate cotransporter [Human][Homo sapiens]
AT1G17665	4.40E-03	0.95	0.84	1.51	1.11	1.58	1.33	0.84	expressed protein
AT5G52410	1.74E-02	0.79	0.83	1.25	1.30	1.58	1.56	0.99	expressed protein
AT2G20340	1.62E-02	0.77	0.91	1.21	1.58	1.58	1.74	1.10	tyrosine decarboxylase, putative similar to tyrosine/dopa decarboxylase [Papaver somniferum] GI:3282527, SP|Q06087 Tyrosine decarboxylase 3 (EC 4.1.1.25) (Petroselinum crispum); contains Pfam profile PF00282: Pyridoxal-dependent decarboxylase conserved domain
AT1G57560	4.83E-03	0.81	0.80	1.28	0.94	1.58	1.16	0.74	myb family transcription factor (MYB50) similar to DNA-binding protein GI:19058 from [Hordeum vulgare]
AT1G75760	9.05E-03	0.95	0.85	1.51	1.24	1.58	1.45	0.92	ER lumen protein retaining receptor family protein similar to SP|P33946 ER lumen protein retaining receptor 1 (KDEL receptor 1) [Bos taurus]; contains Pfam profile PF00810: ER lumen protein retaining receptor
AT1G80720	2.27E-02	0.89	0.92	1.41	1.17	1.58	1.26	0.80	mitochondrial glycoprotein family protein / MAM33 family protein similar to SUAPRGA1 [Emericella nidulans] GI:6562379; contains Pfam profile PF02330: Mitochondrial glycoprotein
AT1G03740	2.80E-02	0.66	0.99	1.04	1.92	1.58	1.93	1.23	protein kinase family protein contains protein kinase domain, Pfam:PF00069
AT4G29810	2.04E-02	0.85	0.98	1.34	1.08	1.58	1.10	0.70	mitogen-activated protein kinase kinase (MAPKK) (MKK2) identical to MAP kinase kinase 2 [Arabidopsis thaliana] gi|3219267|dbj|BAA28828; mitogen-activated protein kinase kinase (MAPKK) family, PMID:12119167
AT1G69360	4.22E-04	0.83	0.68	1.31	1.25	1.58	1.85	1.17	expressed protein
AT5G24030	3.36E-02	0.72	0.88	1.13	1.65	1.57	1.87	1.19	C4-dicarboxylate transporter/malic acid transport family protein weak similarity to SP|P25396 Tellurite resistance protein tehA. (Escherichia coli); contains Pfam profile PF03595: C4-dicarboxylate transporter/malic acid transport protein
AT3G10985	2.13E-04	0.92	0.83	1.45	1.33	1.57	1.60	1.02	wound-responsive protein-related similar to SP|P20144 Wound-induced protein 1 (Solanum tuberosum)
AT3G36860	1.40E-03	0.86	0.83	1.35	1.19	1.57	1.43	0.91	LIM domain-containing protein low similarity to LIM-domain protein [Branchiostoma floridae] GI:3360516, DRAL/Slim3/FHL2 [Homo sapiens] GI:7209525; contains Pfam profile PF00412: LIM domain
AT2G07722;ATMI	2.60E-02	0.96	0.83	1.51	1.11	1.57	1.34	0.85	[AT2G07722, hypothetical protein];[ATMG00170, orf139a hypothetical protein];[ATMG00620, orf139b hypothetical protein]
AT1G78280	9.72E-03	0.81	0.80	1.27	1.35	1.57	1.70	1.08	transcription factor jumonji (jmjC) domain-containing protein contains Pfam PF00646: F-box domain; contains Pfam PF02373: jmjC domain; similar to apoptotic cell clearance receptor PtdSerR (GI:11037740) [Mus musculus]
AT3G01290	1.96E-02	0.74	0.95	1.16	1.25	1.57	1.31	0.84	band 7 family protein similar to hypersensitive-induced response protein [Zea mays] GI:7716470; contains Pfam profile PF01145: SPFH domain / Band 7 family
AT4G38810	9.12E-04	0.84	0.88	1.31	1.27	1.57	1.45	0.92	calcium-binding EF hand family protein contains INTERPRO:IPR002048 calcium-binding EF-hand domain
AT1G19680	2.10E-04	0.87	0.81	1.36	1.34	1.57	1.65	1.05	expressed protein
AT5G44010	1.28E-02	0.87	0.88	1.37	1.04	1.56	1.18	0.75	expressed protein
AT5G03520	1.31E-02	1.01	0.93	1.59	1.02	1.56	1.10	0.70	Ras-related GTP-binding protein, putative similar to GTP-binding protein GI:871508 from [Pisum sativum]
AT5G66170	4.17E-02	1.02	0.64	1.60	0.99	1.56	1.54	0.99	senescence-associated family protein contains similarity to ketoconazole resistant protein GI:928938 and senescence-associated protein GI:1046268 from [Arabidopsis thaliana]
AT4G16760	1.82E-03	0.77	0.81	1.20	1.63	1.56	2.01	1.29	acyl-CoA oxidase (ACX1) identical to acyl-CoA oxidase [Arabidopsis thaliana] GI:3044214
AT4G17240	1.13E-03	0.75	0.82	1.18	1.40	1.56	1.72	1.10	expressed protein
AT1G20840	1.51E-02	0.94	0.69	1.47	1.25	1.56	1.80	1.15	transporter-related low similarity to D-xylose proton-symporter [Lactobacillus brevis] GI:2895856; contains Pfam profile PF00083: major facilitator superfamily protein
AT3G26670	4.64E-03	0.80	0.85	1.25	1.56	1.56	1.83	1.17	expressed protein
AT2G24210	2.23E-02	0.85	0.84	1.32	5.16	1.56	6.17	3.96	myrcene/ocimene synthase (TPS10) nearly identical to GI:9957293; contains Pfam profile: PF01397 terpene synthase family
AT3G22290	5.38E-03	0.98	0.87	1.52	1.11	1.56	1.27	0.81	expressed protein
AT3G02230	3.78E-02	0.85	0.78	1.32	1.07	1.56	1.37	0.88	reversibly glycosylated polypeptide-1 (RGP1) identical to reversibly glycosylated polypeptide-1 (AtRGP) [Arabidopsis thaliana] GI:2317729
AT1G06410	8.80E-03	0.84	0.83	1.34	1.42	1.56	1.71	1.10	glycosyl transferase family 20 protein / trehalose-phosphatase family protein contains Pfam profile: PF02358 trehalose-phosphatase
AT2G18730	5.76E-04	0.81	0.82	1.27	1.23	1.56	1.49	0.96	diacylglycerol kinase, putative contains INTERPRO domain, IPR001206, DAG-kinase catalytic domain
AT3G61210	4.59E-03	0.82	0.84	1.28	1.17	1.56	1.39	0.89	embryo-abundant protein-related similar to embryo-abundant protein [Picea glauca] GI:1350531
AT1G53580	2.32E-02	1.00	0.78	1.54	1.15	1.55	1.48	0.95	hydroxyacylglutathione hydrolase, putative / glyoxalase II, putative similar to glyoxalase II GI:1644427 from [Arabidopsis thaliana]
AT1G26730	3.46E-02	0.87	0.77	1.35	1.03	1.55	1.33	0.86	EXS family protein / ERD1/XPR1/SYG1 family protein similar to PHO1 protein [Arabidopsis thaliana] GI:20069032; contains Pfam profiles PF03105: SPX domain, PF03124: EXS family
AT1G32460	4.94E-02	0.93	0.92	1.43	1.11	1.55	1.21	0.78	expressed protein
AT5G11810	5.08E-03	0.94	0.88	1.45	1.28	1.55	1.46	0.94	expressed protein
AT5G61290	1.91E-02	1.06	0.74	1.64	0.93	1.55	1.26	0.81	flavin-containing monooxygenase family protein / FMO family protein low similarity to FMO3 from Homo sapiens [SP|P31513]; contains Pfam profile: PF00743 Flavin-binding monooxygenase-like; supported by full-length cDNA Ceres:14492
AT5G16340	2.01E-02	0.86	0.94	1.33	1.07	1.55	1.14	0.74	AMP-binding protein, putative similar to AMP-binding protein GI:1903034 from [Brassica napus]; contains Pfam profile PF00501: identical to adenosine monophosphate binding protein 6 AMPBP6 (AMPBP6) GI:20799720
AT3G55410	2.16E-03	0.81	0.74	1.26	1.50	1.55	2.02	1.31	2-oxoglutarate dehydrogenase E1 component, putative / oxoglutarate decarboxylase, putative / alpha-ketoglutaric dehydrogenase, putative similar to SP|P20967 2-oxoglutarate dehydrogenase E1 component, mitochondrial precursor (EC 1.2.4.2) (Alpha-ketoglutarate dehydrogenase) [Saccharomyces cerevisiae]; contains Pfam profiles PF02779: Transketolase, pyridine binding domain, PF00676: Dehydrogenase E1 component
AT5G07100	4.79E-02	0.85	0.96	1.31	1.19	1.55	1.25	0.81	WRKY family transcription factor SPF1 protein - Ipomoea batatas (sweet potato), PIR:S51529
AT1G69960	1.73E-03	0.83	0.87	1.28	1.15	1.55	1.33	0.86	serine/threonine protein phosphatase PP2A-5 catalytic subunit (PP2A5) identical to SP|Q06951|P2A5_ARATH Serine/threonine protein phosphatase PP2A-5 catalytic subunit (EC 3.1.3.16) [Arabidopsis thaliana]; contains Pfam profile PF00149: Ser/Thr protein phosphatase
AT1G22570	1.20E-02	0.77	0.85	1.18	1.25	1.54	1.47	0.95	proton-dependent oligopeptide transport (POT) family protein contains Pfam profile: PF00854 POT family
AT5G40890	1.14E-03	0.81	0.73	1.26	1.35	1.54	1.84	1.19	chloride channel protein (CLC-a) identical to GI:1742952 (gb|AAC05742.1)
AT4G27970	4.64E-04	0.69	1.00	1.06	1.54	1.54	1.54	0.99	C4-dicarboxylate transporter/malic acid transport family protein contains Pfam profile PF03595: C4-dicarboxylate transporter/malic acid transport protein
AT5G40850	7.87E-03	0.67	0.94	1.04	1.37	1.54	1.45	0.94	urophorphyrin III methylase (UPM1) identical to urophorphyrin III methylase [GI:1146165] [Arabidopsis thaliana]; similar to s-adenosyl-L-methionine-dependent uroporphyrinogen III methyltransferase (GI:1490606) [Arabidopsis thaliana]; similar to Diphthine synthase (Diphtamide biosynthesis methyltransferase) (DPH5) (SP:P32469) [Saccharomyces cerevisiae]; contains Pfam PF00590 : Tetrapyrrole (Corrin/Porphyrin) Methylases domain; contains TIGRFAM PF00590: Tetrapyrrole (Corrin/Porphyrin) Methylases
AT5G46760	7.20E-03	0.91	0.81	1.40	1.39	1.54	1.72	1.12	basic helix-loop-helix (bHLH) family protein
AT3G51790	1.18E-02	0.91	0.89	1.40	1.06	1.54	1.19	0.77	cytochrome c biogenesis protein CcmE family contains Pfam profile: PF03100 cytochrome c-type biogenesis protein CcmE
AT3G01830	1.34E-02	0.80	0.85	1.24	1.76	1.54	2.06	1.34	armadillo/beta-catenin repeat family protein / U-box domain-containing protein contains Pfam domain, PF00514: Armadillo/beta-catenin-like repeats and Pfam, PF04564: U-box domain
AT2G37250	9.08E-03	0.62	1.04	0.95	1.34	1.54	1.28	0.83	adenylate kinase family protein contains Pfam profile: PF00406 adenylate kinase
AT2G40435	1.17E-02	0.74	0.94	1.13	1.27	1.54	1.36	0.88	expressed protein
AT5G13760	5.31E-04	0.78	0.83	1.20	1.43	1.54	1.72	1.12	expressed protein similar to unknown protein (gb AAF63775.1)
AT1G02920;AT1G	1.67E-02	0.66	1.04	1.01	1.26	1.54	1.22	0.79	[AT1G02920, glutathione S-transferase, putative similar to glutathione S-transferase GI:860955 from [Hyoscyamus muticus]; supported by cDNA GI:443697.];[AT1G02930, glutathione S-transferase, putative similar to glutathione S-transferase GI:860955 from [Hyoscyamus muticus]]
AT5G53550	2.36E-02	0.62	1.01	0.96	1.73	1.53	1.71	1.11	transporter, putative similar to iron-phytosiderophore transporter protein yellow stripe 1 [Zea mays] GI:10770865; contains Pfam profile PF03169: OPT oligopeptide transporter protein
AT1G54100	1.84E-02	0.94	0.70	1.44	1.10	1.53	1.57	1.03	aldehyde dehydrogenase, putative / antiquitin, putative strong similarity to SP|Q41247 Aldehyde dehydrogenase family 7 member A1 (EC 1.2.1.3) (Antiquitin 1) (Brassica turgor-responsive/drought-induced gene 26 protein) (Btg-26) [Brassica napus]; similar to turgor-responsive protein 26G (aldehyde dehydrogenase family 7 member A1) [Pisum sativum] SWISS-PROT:P25795
AT4G30340	1.38E-02	0.72	0.98	1.10	1.43	1.53	1.46	0.96	diacylglycerol kinase family protein contains INTERPRO domain, IPR001206, DAG-kinase catalytic domain
AT2G39050	4.09E-04	0.67	0.99	1.03	1.96	1.53	1.97	1.29	hydroxyproline-rich glycoprotein family protein contains QXW lectin repeat domain, Pfam PF00652
AT1G73500	2.55E-03	0.83	0.72	1.27	1.14	1.53	1.59	1.04	mitogen-activated protein kinase kinase (MAPKK), putative (MKK9) mitogen-activated protein kinase kinase (MAPKK) family, PMID:12119167
AT3G26680	1.75E-02	0.66	0.90	1.01	1.56	1.53	1.73	1.13	DNA cross-link repair protein-related contains weak similarity to Swiss-Prot:P30620 DNA cross-LINK repair protein PSO2/SNM1 [Saccharomyces cerevisiae]
AT3G54120	3.46E-02	0.83	0.99	1.27	1.17	1.53	1.18	0.77	reticulon family protein (RTNLB12) contains Pfam profile PF02453: Reticulon
AT1G72470	5.13E-03	0.87	0.81	1.33	1.16	1.53	1.44	0.94	exocyst subunit EXO70 family protein contains Pfam domain PF03081: Exo70 exocyst complex subunit;
AT1G09850	2.60E-04	0.86	0.80	1.31	1.15	1.53	1.43	0.94	cysteine protease, papain-like (XBCP3) identical to papain-like cysteine peptidase XBCP3 GI:14600257 from [Arabidopsis thaliana] ; contains Pfam profiles PF00112: Papain family cysteine protease and PF00396: Granulin
AT1G67560	7.26E-04	0.79	0.78	1.20	1.51	1.53	1.94	1.27	lipoxygenase family protein similar to 13-lipoxygenase GB:CAA65269 [Solanum tuberosum], gi:1654140 [Lycopersicon esculentum]
AT4G21865	5.24E-03	0.81	0.95	1.23	1.17	1.53	1.24	0.81	expressed protein
AT1G72800	1.78E-02	0.71	0.92	1.09	1.20	1.53	1.30	0.85	nuM1-related contains similarity with nuM1 GI:1279563 from [Medicago sativa]
AT3G07980	3.25E-02	0.85	0.80	1.30	1.61	1.53	2.01	1.31	protein kinase, putative similar to MAP3K epsilon protein kinase [Arabidopsis thaliana] gi|3549652|emb|CAA12272
AT5G60270	2.17E-04	0.82	0.93	1.24	1.62	1.53	1.75	1.14	lectin protein kinase family protein contains Pfam domains, PF00069: Protein kinase domain, PF00139: Legume lectins beta domain, and PF00138: Legume lectins alpha domain
AT5G52250	7.06E-03	0.90	0.86	1.38	1.13	1.53	1.39	0.91	transducin family protein / WD-40 repeat family protein contains 7 WD-40 repeats (PF00400); similar to photomorphogenesis repressor PnCOP1 (GI:11127996) [Ipomoea nil]
AT4G38460	4.19E-04	0.76	0.86	1.39	1.23	1.53	1.44	0.94	geranylgeranyl pyrophosphate synthase, putative / GGPP synthetase, putative / farnesyltranstransferase, putative 65% similar to geranylgeranyl pyrophosphate synthase [GI:413730];
AT5G52870	9.32E-03	0.88	0.75	1.34	1.29	1.52	1.72	1.13	calmodulin-binding family protein contains Pfam profile PF00612: IQ calmodulin-binding motif
AT5G54390	3.61E-03	0.77	0.77	1.17	1.10	1.52	1.43	0.94	inositol monophosphatase family protein similar to SP|Q42546 3'(2'),5'-bisphosphate nucleotidase (EC 3.1.3.7) [Arabidopsis thaliana]; contains Pfam profile PF00459: Inositol monophosphatase family; supporting cDNA gi|1354509|gb|U55205.1|ATU55205
AT1G34300	2.32E-02	0.83	0.92	1.27	1.25	1.52	1.36	0.89	lectin protein kinase family protein contains Pfam domains, PF01453: Lectin (probable mannose binding) and PF00069: Protein kinase domain
AT4G19420	5.04E-03	0.82	0.84	1.25	1.38	1.52	1.64	1.08	pectinacetylesterase family protein contains Pfam profile: PF03283 pectinacetylesterase
AT5G52570	3.40E-02	0.76	0.96	1.16	2.01	1.52	2.10	1.38	beta-carotene hydroxylase, putative similar to GI:1575296, beta-carotene hydroxylase
AT1G49520	3.78E-02	0.90	0.92	1.37	1.39	1.52	1.51	1.00	SWIB complex BAF60b domain-containing protein contains Pfam profile PF02201: BAF60b domain of the SWIB complex
AT5G04040	3.56E-02	0.87	0.81	1.32	1.17	1.52	1.45	0.95	patatin-related contains Patatin domain PF01734
AT3G54110	1.74E-04	0.73	0.81	1.11	1.32	1.52	1.63	1.07	plant uncoupling mitochondrial protein (PUMP) identical to plant uncoupling mitochondrial protein [Arabidopsis thaliana] GI:3115108
AT1G74100	2.67E-02	0.68	0.90	1.03	1.14	1.52	1.28	0.84	sulfotransferase family protein similar to SP|P52837 Flavonol 4'-sulfotransferase (EC 2.8.2.-) (F4-ST) [Flaveria chloraefolia]; contains Pfam profile PF00685: Sulfotransferase domain
AT2G30860	2.60E-04	0.79	0.87	1.19	1.15	1.52	1.33	0.88	glutathione S-transferase, putative identical to GB:Y12295
AT2G39180	1.78E-02	0.74	0.87	1.13	1.86	1.52	2.15	1.42	protein kinase family protein contains protein kinase domain, Pfam:PF00069
AT5G07700	4.70E-04	0.83	0.82	1.26	1.43	1.52	1.74	1.15	myb family transcription factor (MYB76) contains Pfam profile: PF00249 myb-like DNA-binding domain
AT3G28007	9.96E-04	0.74	0.92	1.13	1.24	1.52	1.34	0.89	nodulin MtN3 family protein contains Pfam PF03083 MtN3/saliva family; similar to LIM7 GI:431154 (induced in meiotic prophase in lily microsporocytes) from [Lilium longiflorum]
AT3G20390	5.84E-03	0.96	0.87	1.45	1.50	1.52	1.72	1.14	endoribonuclease L-PSP family protein contains Pfam domain PF01042: Endoribonuclease L-PSP
AT2G47650	8.90E-03	0.78	0.95	1.18	1.35	1.51	1.42	0.94	NAD-dependent epimerase/dehydratase family protein similar to UDP-glucuronic acid decarboxylase Uxs1p from Filobasidiella neoformans GI:14318327; contains Pfam profile PF01370 NAD dependent epimerase/dehydratase family; contains non-consensus AT donor splice site at exon 1 and non-consensus AC acceptor splice site at exon 2
AT5G65600	1.11E-02	0.88	0.95	1.33	1.38	1.51	1.46	0.96	FAD-binding domain-containing protein strong similarity to At1g32300, At2g46740, At2g46750, At2g46760; contains PF01565: FAD binding domain
AT1G12450	1.91E-03	0.90	0.75	1.36	1.00	1.51	1.34	0.89	expressed protein
AT2G38050	1.53E-02	0.56	1.14	0.84	1.77	1.51	1.55	1.02	3-oxo-5-alpha-steroid 4-dehydrogenase, putative / steroid 5-alpha-reductase, putative identical to gi:1280611; contains a steroid 5-alpha reductase, C-terminal domain
AT1G58180	5.65E-03	0.96	0.78	1.45	1.14	1.51	1.45	0.96	carbonic anhydrase family protein / carbonate dehydratase family protein similar to SP|P46512 Carbonic anhydrase 1 (EC 4.2.1.1) (Carbonate dehydratase 1) [Flaveria linearis]; contains Pfam profile PF00484: Carbonic anhydrase
AT5G53460	1.61E-02	0.76	0.83	1.15	1.56	1.51	1.87	1.24	glutamate synthase [NADH], chloroplast, putative similar to SP|Q03460 Glutamate synthase [NADH], chloroplast precursor (EC 1.4.1.14) (NADH- GOGAT) (Medicago sativa)
AT5G40610	2.03E-03	0.80	0.92	1.21	1.03	1.51	1.12	0.75	glycerol-3-phosphate dehydrogenase [NAD+] / GPDH strong similarity to SP|P52425 Glycerol-3-phosphate dehydrogenase [NAD+] (EC 1.1.1.8) (Cuphea lanceolata); contains Pfam profile PF01210: NAD-dependent glycerol-3-phosphate dehydrogenase
AT3G28640	1.07E-03	0.78	0.88	1.17	1.31	1.51	1.50	0.99	alphavirus core protein family contains Pfam profile: PF00944 alphavirus core protein
AT3G30380	9.02E-03	0.93	0.90	1.40	1.17	1.51	1.29	0.86	expressed protein ; expression supported by MPSS
AT4G32800	3.36E-02	0.87	0.71	1.30	1.45	1.50	2.03	1.35	AP2 domain-containing transcription factor TINY, putative similar to transcription factor TINY - Arabidopsis thaliana, PIR2:T01076
AT3G44880	2.39E-03	0.96	0.74	1.44	1.28	1.50	1.73	1.15	Rieske [2Fe-2S] domain-containing protein similar to lethal leaf-spot 1 from Zea mays [gi:1935909]; contains Pfam PF00355 Rieske [2Fe-2S] domain
AT5G03270	1.32E-02	0.87	0.88	1.31	1.05	1.50	1.19	0.79	expressed protein contains Pfam profile PF03641: decarboxylase family protein
AT5G47240	3.10E-03	0.91	0.77	1.37	1.09	1.50	1.41	0.94	MutT/nudix family protein similar to SP|P53370 Nucleoside diphosphate-linked moiety X motif 6 [Homo sapiens]; contains Pfam profile PF00293: NUDIX domain
AT2G30550	8.80E-04	0.85	0.84	1.28	1.26	1.50	1.51	1.01	lipase class 3 family protein similar to DEFECTIVE IN ANTHER DEHISCENCE1 [Arabidopsis thaliana] GI:16215706; contains Pfam profile PF01764: Lipase
AT4G23590	4.06E-03	0.89	0.93	1.33	1.10	1.50	1.18	0.79	aminotransferase class I and II family protein similar to nicotianamine aminotransferase from Hordeum vulgare [GI:6498122, GI:6469087]; contains Pfam profile PF00155 aminotransferase, classes I and II
AT4G21450	2.49E-03	0.76	0.92	1.14	1.11	1.50	1.22	0.81	nodulin MtN21 family protein similar to MtN21 GI:2598575 (root nodule development) from [Medicago truncatula]
AT1G07260	1.01E-02	0.77	0.70	1.15	1.53	1.50	2.19	1.46	UDP-glucoronosyl/UDP-glucosyl transferase family protein contains Pfam profile: PF00201 UDP-glucoronosyl and UDP-glucosyl transferase
AT1G60090	1.26E-03	0.82	0.82	1.22	1.27	1.50	1.55	1.03	glycosyl hydrolase family 1 protein contains Pfam PF00232 : Glycosyl hydrolase family 1 domain; TIGRFAM TIGR01233: 6-phospho-beta-galactosidase; similar to hydroxyisourate hydrolase (GI:19569603) [Glycine max]
AT5G33290	2.01E-02	0.84	0.79	1.26	1.33	1.50	1.68	1.12	exostosin family protein contains Pfam profile: PF03016 Exostosin family
AT5G53750	3.55E-03	0.85	0.81	1.28	1.58	1.50	1.95	1.30	expressed protein strong similarity to unknown protein (pir||T08938)
AT5G12890	4.77E-04	0.89	0.87	1.33	1.22	1.50	1.40	0.93	UDP-glucoronosyl/UDP-glucosyl transferase family protein contains Pfam profile: PF00201 UDP-glucoronosyl and UDP-glucosyl transferase
AT5G47180	7.83E-04	1.06	0.69	1.59	0.99	1.50	1.44	0.96	vesicle-associated membrane family protein / VAMP family protein similar to VAP27 GI:6688926 [Nicotiana plumbaginifolia], to VAMP-associated protein B GI:4240464 [Rattus norvegicus] and to Vesicle-associated membrane protein/synaptobrevin binding protein (VAP-33) (SP:Q16943)[Aplysia californica]
AT1G15420	3.70E-02	0.90	0.91	1.34	0.96	1.50	1.06	0.71	expressed protein
ATMG01020	2.35E-02	0.98	0.94	1.46	1.12	1.50	1.20	0.80	orf135b hypothetical protein
AT3G06490	2.07E-02	0.88	0.85	1.31	1.03	1.50	1.22	0.82	myb family transcription factor (MYB108) identical to transcription factor MYB108 GI:15375290 from [Arabidopsis thaliana]
AT5G64570	1.16E-02	0.91	0.60	1.36	1.04	1.50	1.74	1.16	glycosyl hydrolase family 3 protein
AT4G29210	8.01E-03	0.81	0.80	1.21	1.37	1.50	1.72	1.15	gamma-glutamyltranspeptidase family protein similar to SP|P07314 Gamma-glutamyltranspeptidase precursor (EC 2.3.2.2) (Gamma- glutamyltransferase) (GGT) [Rattus norvegicus]; contains Pfam profilePF01019: Gamma-glutamyltranspeptidase
AT3G05390	5.64E-03	0.87	0.84	1.29	1.23	1.50	1.47	0.98	expressed protein ; expression supported by MPSS
AT5G16910	7.96E-03	0.84	0.89	1.26	1.42	1.49	1.59	1.07	cellulose synthase family protein similar to gi:2827143 cellulose synthase catalytic subunit, Arabidopsis thaliana, gi:9622886 cellulose synthase-7 from Zea mays
AT2G37900	2.32E-02	0.67	0.99	1.00	1.57	1.49	1.59	1.06	proton-dependent oligopeptide transport (POT) family protein contains Pfam profile: PF00854 POT family
AT2G42280	3.36E-02	0.59	1.10	0.88	1.39	1.49	1.26	0.84	basic helix-loop-helix (bHLH) family protein
AT1G31710	2.32E-02	0.83	0.85	1.24	1.50	1.49	1.76	1.18	copper amine oxidase, putative similar to copper amine oxidase [Lens culinaris] gi|15451834|gb|AAB34918
AT1G61640	2.17E-02	0.75	0.84	1.12	1.34	1.49	1.61	1.08	ABC1 family protein contains Pfam domain, PF03109: ABC1 family
AT1G62310	1.13E-02	0.89	0.69	1.33	1.69	1.49	2.44	1.64	transcription factor jumonji (jmjC) domain-containing protein similar to nuclear protein SqNCA [Homo sapiens] GI:13161188; contains Pfam profile PF02373: jmjC domain
AT5G64700	6.37E-03	0.76	0.94	1.14	1.48	1.49	1.57	1.05	nodulin MtN21 family protein similar to MtN21 GI:2598575 (root nodule development) from [Medicago truncatula]; contains Pfam profile PF00892: Integral membrane protein
AT4G09510	2.19E-03	0.81	0.78	1.21	1.44	1.49	1.85	1.24	beta-fructofuranosidase, putative / invertase, putative / saccharase, putative / beta-fructosidase, putative similar to neutral invertase [Daucus carota] GI:4200165; contains Pfam profile PF04853: Plant neutral invertase
AT1G54740	4.93E-02	0.98	0.86	1.46	1.00	1.49	1.16	0.78	expressed protein
AT5G26280;AT5G	3.54E-03	0.86	0.87	1.28	1.24	1.49	1.44	0.97	[AT5G26280, meprin and TRAF homology domain-containing protein / MATH domain-containing protein low similarity to ubiquitin-specific protease 12 [Arabidopsis thaliana] GI:11993471; contains Pfam profile PF00917: MATH domain];[AT5G26260, meprin and TRAF homology domain-containing protein / MATH domain-containing protein similar to ubiquitin-specific protease 12 [Arabidopsis thaliana] GI:11993471; contains Pfam profile PF00917: MATH domain]
AT3G51090	3.01E-03	0.97	0.88	1.44	1.16	1.49	1.32	0.89	expressed protein
AT3G60260	1.16E-02	0.94	0.87	1.40	1.07	1.49	1.23	0.83	phagocytosis and cell motility protein ELMO1-related contains weak similarity to ELMO1 [Mus musculus] gi|16118551|gb|AAL14464
AT5G63010	7.42E-04	0.80	0.79	1.19	1.16	1.49	1.47	0.99	WD-40 repeat family protein contains 4 WD-40 repeats (PF00400);low similarity to photomorphogenesis repressor (COP1) GI:2702280 [Arabidopsis thaliana] and COP1 GI:11127996 [Ipomoea nil]
AT5G65650	8.37E-03	0.96	0.86	1.42	1.01	1.49	1.17	0.79	expressed protein
AT2G48020	3.63E-03	0.91	0.77	1.35	1.11	1.48	1.44	0.97	sugar transporter, putative similar to ERD6 protein [Arabidopsis thaliana] GI:3123712, sugar-porter family proteins 1 and 2 [Arabidopsis thaliana] GI:14585699, GI:14585701; contains Pfam profile PF00083: major facilitator superfamily protein
AT1G34420	1.87E-02	0.84	0.86	1.25	1.39	1.48	1.62	1.09	leucine-rich repeat family protein / protein kinase family protein contains leucine rich repeat (LRR) domains, Pfam:PF00560; contains protein kinase domain, Pfam:PF00069
AT5G52450	6.18E-03	0.75	0.85	1.11	1.31	1.48	1.53	1.03	MATE efflux-related strong similarity to unknown protein (pir||T02324); contains Pfam profile PF01554 Uncharacterized membrane protein family
AT5G65080	4.03E-02	1.26	0.47	1.87	0.68	1.48	1.46	0.99	MADS-box family protein
AT5G60290	3.98E-02	0.92	0.93	1.36	1.14	1.48	1.22	0.82	hypothetical protein
AT1G76090	2.23E-03	0.88	0.71	1.31	1.16	1.48	1.64	1.11	S-adenosyl-methionine-sterol-C-methyltransferase identical to S-adenosyl-methionine-sterol-C-methyltransferase GI:2246456 from [Arabidopsis thaliana]
AT5G15410	1.14E-02	0.88	0.72	1.31	1.21	1.48	1.68	1.14	cyclic nucleotide-regulated ion channel / cyclic nucleotide-gated channel (CNGC2) identical to cyclic nucleotide-gated cation channel GI:3894399 from [Arabidopsis thaliana]
AT1G14130	7.50E-03	0.97	0.61	1.44	1.05	1.48	1.71	1.15	2-oxoglutarate-dependent dioxygenase, putative similar to adventitious rooting related oxygenase ARRO-1 from Malus x domestica, gi|3492806; contains Pfam domain PF03171, 2OG-Fe(II) oxygenase superfamily
AT2G28940	2.47E-02	0.76	1.01	1.12	1.16	1.48	1.15	0.78	avirulence-responsive protein, putative / avirulence induced gene (AIG) protein, putative strong similarity to SP|P54121 AIG2 protein (avrRpt2-induced gene) [Arabidopsis thaliana]
AT1G13210	4.45E-02	0.66	1.10	0.98	1.95	1.48	1.78	1.20	haloacid dehalogenase-like hydrolase family protein similar to Potential phospholipid-transporting ATPase (EC 3.6.3.1) (Chromaffin granule ATPase) from (Homo sapiens) SP|Q9Y2Q0, (Mus musculus) SP|P98200, (Bos taurus) SP|Q29449; contains InterPro accession IPR005834: Haloacid dehalogenase-like hydrolase; ESTs gb|T45045 and gb|AA394473 come from this gene
AT3G54070	4.97E-02	0.99	0.75	1.47	0.94	1.48	1.26	0.85	ankyrin repeat family protein contains ankyrin repeats, Pfam domain PF00023
AT4G16563	1.40E-02	0.96	0.68	1.42	1.19	1.48	1.75	1.18	aspartyl protease family protein contains Pfam profile: PF00026 eukaryotic aspartyl protease
AT1G04930	2.68E-03	0.91	0.95	1.34	1.11	1.48	1.17	0.79	hydroxyproline-rich glycoprotein family protein Common family member: At2g32840 [Arabidopsis thaliana]
AT1G14810	6.18E-05	0.84	0.80	1.23	1.26	1.47	1.58	1.07	semialdehyde dehydrogenase family protein similar to SP:O31219 Aspartate-semialdehyde dehydrogenase (EC 1.2.1.11) (ASA dehydrogenase) (ASADH) (Legionella pneumophila); contains Pfam profiles PF02774: Semialdehyde dehydrogenase dimerisation domain, PF01118: Semialdehyde dehydrogenase NAD binding domain
AT1G14290	5.89E-03	1.01	0.76	1.49	1.04	1.47	1.36	0.92	acid phosphatase, putative similar to acid phosphatase [Lupinus albus] GI:5360721; contains Pfam profile PF01598 sterol desaturase
AT5G04250	3.80E-03	0.94	0.77	1.39	1.31	1.47	1.69	1.15	OTU-like cysteine protease family protein contains Pfam profile PF02338: OTU-like cysteine protease
AT1G14060	4.41E-02	0.92	0.96	1.35	1.14	1.47	1.19	0.80	expressed protein
AT4G08930	3.78E-02	0.92	0.87	1.36	1.15	1.47	1.33	0.90	thioredoxin-related contains weak similarity to Swiss-Prot:Q39239 thioredoxin H-type 4 (TRX-H-4). [Mouse-ear cress]
AT1G06800	6.05E-04	0.74	0.90	1.08	1.40	1.47	1.56	1.06	lipase class 3 family protein similar to DEFECTIVE IN ANTHER DEHISCENCE1 [Arabidopsis thaliana] GI:16215706; contains Pfam profile PF01764: Lipase
AT1G59960	1.14E-02	0.68	1.00	1.00	1.27	1.47	1.27	0.86	aldo/keto reductase, putative similar to NADPH-dependent codeinone reductase GI:6478210 [Papaver somniferum], NAD(P)H dependent 6'-deoxychalcone synthase [Glycine max][GI:18728]
AT4G10390	2.20E-02	0.84	0.76	1.23	1.21	1.47	1.59	1.08	protein kinase family protein contains protein kinase domain, Pfam:PF00069
AT1G50490;AT1G	1.67E-03	0.89	0.85	1.30	1.22	1.47	1.44	0.98	[AT1G50490, ubiquitin-conjugating enzyme 20 (UBC20) nearly identical to ubiquitin-conjugating enzyme UBC20 [Arabidopsis thaliana] GI:22530867; contains Pfam profile PF00179: Ubiquitin-conjugating enzyme];[AT1G50480, formate--tetrahydrofolate ligase / 10-formyltetrahydrofolate synthetase (THFS) identical to 10-formyltetrahydrofolate synthetase (Arabidopsis thaliana) GI:5921663]
AT1G13870	2.14E-02	0.96	0.88	1.41	1.00	1.47	1.14	0.78	expressed protein similar to KTI12 protein (SP:P34253) [Saccharomyces cerevisiae]; contains Prosite PS00070: Aldehyde dehydrogenases cysteine active site
AT2G21630	3.09E-03	0.81	0.86	1.20	1.30	1.47	1.39	0.94	transport protein, putative similar to Swiss-Prot:Q15436 protein transport protein Sec23A [Homo sapiens]
AT2G42580	7.39E-03	0.78	1.04	1.15	1.81	1.47	1.74	1.19	tetratricopeptide repeat (TPR)-containing protein contains Pfam profile PF00515 TPR Domain
AT4G02520;AT2G	9.24E-03	0.78	1.00	1.14	1.21	1.46	1.21	0.83	[AT4G02520, glutathione S-transferase, putative];[AT2G02930, glutathione S-transferase, putative]
AT3G11180	9.07E-03	0.91	0.90	1.33	1.12	1.46	1.25	0.85	oxidoreductase, 2OG-Fe(II) oxygenase family protein similar to leucoanthocyanidin dioxygenase GB:BAA20143 [Perilla frutescens], Malus domestica, SP|P51091; contains PF03171 2OG-Fe(II) oxygenase superfamily domain
AT5G54170	2.58E-03	0.80	0.81	1.18	1.16	1.46	1.44	0.98	expressed protein weak similarity to SP|Q9UKL6 Phosphatidylcholine transfer protein (PC-TP) (Homo sapiens)
AT1G77380	1.74E-03	0.85	0.91	1.25	1.20	1.46	1.32	0.90	amino acid carrier, putative / amino acid permease, putative strong similarity to amino acid carrier GI:3293031 from [Ricinus communis]; contains Pfam profile PF01490: Transmembrane amino acid transporter protein; identical to cDNA AAP3 (Amino Acid Permease) GI:3970651
AT3G21270	2.70E-03	0.88	0.96	1.28	1.11	1.46	1.16	0.79	Dof-type zinc finger domain-containing protein (ADOF2) identical to Dof zinc finger protein ADOF2 GI:3608263 from [Arabidopsis thaliana]; identical to cDNA adof2 mRNA for Dof zinc finger protein GI:3608262; contains Pfam profile PF02701: Dof domain, zinc finger
AT4G25900	1.39E-02	0.78	0.82	1.13	1.26	1.46	1.54	1.05	aldose 1-epimerase family protein similar to apospory-associated protein C; APOC (Chlamydomonas reinhardtii) GI:6970044 Pfam profile PF01263: Aldose 1-epimerase
AT5G14130;AT5G	1.06E-03	0.81	0.76	1.19	1.22	1.46	1.61	1.10	[AT5G14130, peroxidase, putative identical to peroxidase ATP20a [Arabidopsis thaliana] gi|1546684|emb|CAA67338];[AT5G14120, nodulin family protein similar to nodulin-like protein [Arabidopsis thaliana] GI:3329368, nodule-specific protein Nlj70 [Lotus japonicus] GI:3329366]
AT5G50960	6.31E-04	0.86	0.85	1.26	1.19	1.46	1.41	0.96	nucleotide-binding family protein similar to Nucleotide-binding protein 1 (NBP 1) (SP:Q9R060) [Mus musculus]; contains Pfam PF00991 : ParA family ATPase
AT5G08300	8.26E-04	0.92	0.92	1.34	1.18	1.46	1.29	0.88	succinyl-CoA ligase [GDP-forming] alpha-chain, mitochondrial, putative / succinyl-CoA synthetase, alpha chain, putative / SCS-alpha, putative identical to SP|P53586 Succinyl-CoA ligase [GDP-forming] alpha-chain, mitochondrial precursor (EC 6.2.1.4) (Succinyl-CoA synthetase, alpha chain) (SCS-alpha) (Arabidopsis thaliana); strong similarity to SP|P13086 Succinyl-CoA ligase [GDP-forming] alpha-chain, mitochondrial precursor (Rattus norvegicus); contains Pfam profiles PF00549: CoA-ligase, PF02629: CoA binding domain
AT1G34340	1.62E-02	0.92	0.92	1.34	1.22	1.46	1.33	0.91	esterase/lipase/thioesterase family protein similar to late embryogenesis abundant protein (EMB8) GI:1350544 SP|Q40863 from (Picea glauca); contains Interpro entry IPR000379 esterase/lipase/thioesterase family
AT4G01770;AT4G	1.74E-02	0.70	0.91	1.02	1.32	1.46	1.46	0.99	[AT4G01770, hypothetical protein similar to T15B16.9];[AT4G01750, expressed protein T15B16.8]
AT2G28680	2.64E-02	0.88	0.93	1.28	1.08	1.46	1.16	0.80	cupin family protein similar to legumin (11S-globulin) from Ginkgo biloba [GI:949869], 11S globulin from Avena sativa [GI:472867]; contains a 11-S plant seed storage protein signature (PS00305)
AT2G31820	2.90E-02	0.79	0.97	1.15	1.27	1.46	1.31	0.90	ankyrin repeat family protein contains ankyrin repeat domains, Pfam:PF00023
AT1G68330	3.89E-02	0.82	0.87	1.19	1.18	1.46	1.36	0.93	expressed protein
AT5G50670;AT5G	2.59E-02	0.82	0.70	1.19	1.16	1.45	1.65	1.14	[AT5G50670, squamosa promoter-binding protein, putative similar to squamosa promoter binding protein-like 9 [Arabidopsis thaliana] GI:5931673; contains Pfam profile PF03110: SBP domain];[AT5G50570, squamosa promoter-binding protein, putative similar to squamosa promoter binding protein-like 9 [Arabidopsis thaliana] GI:5931677; contains Pfam profile PF03110: SBP domain]
AT1G70230	1.39E-03	0.82	0.87	1.19	1.37	1.45	1.58	1.09	expressed protein
AT5G51290	4.02E-03	0.83	0.91	1.21	1.43	1.45	1.57	1.08	ceramide kinase-related contains weak similarity to ceramide kinases (GI:21624342) [Mus musculus]
AT3G62420	4.85E-03	0.92	0.81	1.34	1.02	1.45	1.26	0.87	bZIP transcription factor family protein similar to common plant regulatory factor 6 GI:9650826 from [Petroselinum crispum]
AT4G12790	8.43E-03	0.93	0.89	1.36	1.24	1.45	1.40	0.96	ATP-binding family protein contains Pfam domain, PF03029: Conserved hypothetical ATP binding protein
AT1G30220	1.74E-02	0.83	0.86	1.20	1.18	1.45	1.37	0.95	sugar transporter family protein similar to SP|Q96QE2 Proton myo-inositol co-transporter (Hmit) [Homo sapiens]; contains Pfam profile PF00083: major facilitator superfamily protein
AT1G14230	6.17E-03	0.90	0.83	1.31	1.29	1.45	1.55	1.07	AP2 domain-containing protein RAP2.2 (RAP2.2) identical to AP2 domain containing protein RAP2.2 GI:2281629 from [Arabidopsis thaliana]
AT5G66760	2.32E-02	0.93	0.88	1.35	1.38	1.45	1.57	1.08	succinate dehydrogenase [ubiquinone] flavoprotein subunit, mitochondrial / flavoprotein subunit of complex II identical to SP|O82663 Succinate dehydrogenase [ubiquinone] flavoprotein subunit, mitochondrial (EC 1.3.5.1) (FP) (Flavoprotein subunit of complex II) (Arabidopsis thaliana)
AT1G72440	2.20E-02	0.89	0.79	1.29	1.23	1.45	1.55	1.07	CCAAT-box-binding transcription factor-related similar to CCAAT-box-binding transcription factor (CCAAT-binding factor) (CBF) (Swiss-Prot:Q03701) [Homo sapiens], GB:P53569 [Mus musculus]
AT4G08460	1.06E-02	0.87	0.88	1.27	1.12	1.45	1.28	0.88	expressed protein
AT5G62000	6.48E-03	0.85	0.87	1.23	1.45	1.45	1.67	1.15	transcriptional factor B3 family protein / auxin-responsive factor, putative (ARF1) contains Pfam profile PF02362 B3 DNA binding domain; identical to cDNA ARF1 (auxin response factor) binding protein GI:2245393
AT2G30600	3.56E-03	0.96	0.86	1.39	1.23	1.45	1.43	0.99	BTB/POZ domain-containing protein contains Pfam PF00651: BTB/POZ domain; contains Interpro IPR000210/ PS50097: BTB/POZ domain; similar to MigA (GI:1841872) [Dictyostelium discoideum]
AT5G33370	2.42E-02	0.80	0.84	1.16	1.42	1.45	1.69	1.17	GDSL-motif lipase/hydrolase family protein similar to family II lipase EXL3 (GI:15054386), EXL1 (GI:15054382), EXL2 (GI:15054384) [Arabidopsis thaliana]; contains Pfam profile PF00657: Lipase/Acylhydrolase with GDSL-like motif
AT3G09010	8.15E-03	0.70	1.04	1.01	1.59	1.45	1.54	1.06	protein kinase family protein contains protein kinase domain, Pfam:PF00069
AT1G20490	3.63E-03	0.81	0.82	1.17	1.43	1.45	1.74	1.20	AMP-dependent synthetase and ligase family protein similar to SP|Q42524 and SP|Q9S725; contains Pfam AMP-binding enzyme domain PF00501
AT5G55560	3.98E-02	1.00	0.79	1.44	1.02	1.45	1.29	0.89	protein kinase family protein contains protein kinase domain, Pfam:PF00069
AT2G44580	4.86E-02	0.88	0.92	1.28	1.47	1.45	1.60	1.11	zinc finger (C3HC4-type RING finger) family protein contains Pfam profile: PF00097 zinc finger, C3HC4 type (RING finger)
AT1G22330	2.52E-02	0.91	0.81	1.32	1.06	1.45	1.31	0.91	RNA recognition motif (RRM)-containing protein similar to UBP1 interacting protein 1a [Arabidopsis thaliana] GI:19574236; contains InterPro entry IPR000504: RNA-binding region RNP-1 (RNA recognition motif) (RRM)
AT5G56090	1.73E-03	0.88	0.78	1.27	1.14	1.45	1.46	1.01	cytochrome oxidase assembly family protein contains Pfam PF02628: Cytochrome oxidase assembly protein
AT5G13490	1.22E-02	0.92	0.84	1.33	1.09	1.45	1.29	0.89	ADP, ATP carrier protein 2, mitochondrial / ADP/ATP translocase 2 / adenine nucleotide translocator 2 (ANT2) identical to SWISS-PROT:P40941 ADP,ATP carrier protein 2, mitochondrial precursor (Adenine nucleotide translocator 2) [Arabidopsis thaliana]
AT1G74690	2.80E-02	0.91	0.73	1.31	1.40	1.45	1.90	1.31	gibberellin response modulator, putative / gibberellin-response modulator, putative member of the VHIID domain transcription factor family RGAL - Arabidopsis thaliana, EMBL:AJ224957
AT2G40940	1.93E-02	0.65	1.00	0.94	1.49	1.45	1.49	1.03	ethylene response sensor / ethylene-responsive sensor (ERS) identical to ethylene response sensor (ERS) [Arabidopsis thaliana] GI:1046225
AT1G74090	2.50E-02	0.86	0.86	1.24	1.17	1.44	1.36	0.94	sulfotransferase family protein similar to SP|P52837 Flavonol 4'-sulfotransferase (EC 2.8.2.-) (F4-ST) [Flaveria chloraefolia]; contains Pfam profile PF00685: Sulfotransferase domain
AT5G02830;AT5G	7.04E-04	0.82	0.88	1.19	1.17	1.44	1.33	0.92	[AT5G02830, pentatricopeptide (PPR) repeat-containing protein contains Pfam profile PF01535: PPR repeat];[AT5G02820, DNA topoisomerase VIA (SPO11) identical to topoisomerase 6 subunit A (spo11) [Arabidopsis thaliana] GI:12331186]
AT3G15510	4.62E-03	0.86	0.89	1.24	1.23	1.44	1.38	0.96	no apical meristem (NAM) family protein (NAC2) identical to AtNAC2 [Arabidopsis thaliana] GI:12060426; contains Pfam PF02365: No apical meristem (NAM) domain; similar to jasmonic acid 2 GB:AAF04915 from [Lycopersicon esculentum]
AT1G79310	4.88E-02	0.89	0.83	1.28	1.09	1.44	1.31	0.91	latex-abundant protein, putative (AMC4) / caspase family protein similar to latex-abundant protein [Hevea brasiliensis] gb:AAD13216; contains Pfam profile PF00656: ICE-like protease (caspase) p20 domain
AT4G19230	7.87E-03	0.89	0.83	1.28	1.20	1.44	1.44	1.00	cytochrome P450 family protein cytochrome P450, Arabidopsis thaliana; supported by cDNA: gi_15293092_gb_AY050980.1_
AT1G72830	1.27E-02	0.68	1.03	0.98	1.21	1.44	1.18	0.82	CCAAT-binding transcription factor (CBF-B/NF-YA) family protein contains Pfam PF02045 CCAAT-binding transcription factor (CBF-B/NF-YA) subunit B
AT1G63010	5.48E-03	0.89	0.81	1.28	1.44	1.44	1.78	1.24	SPX (SYG1/Pho81/XPR1) domain-containing protein contains Pfam profile PF03105: SPX domain
AT5G19520	2.83E-03	0.84	0.90	1.20	1.35	1.44	1.50	1.04	mechanosensitive ion channel domain-containing protein / MS ion channel domain-containing protein contains Pfam profile PF00924: Mechanosensitive ion channel
AT3G51430	2.72E-02	0.91	0.87	1.32	1.23	1.44	1.42	0.98	strictosidine synthase, putative (YLS2) similar to hemomucin [Drosophila melanogaster][GI:1280434], strictosidine synthase [Rauvolfia serpentina][SP|P15324]; contains strictosidine synthase domain PF03088; identical to cDNA YLS2 mRNA for strictosidine synthase-like protein GI:13122281
AT5G37710	2.68E-02	0.81	0.83	1.17	1.21	1.44	1.45	1.01	lipase class 3 family protein / calmodulin-binding heat-shock protein, putative similar to almodulin-binding heat-shock protein, common tobacco, PIR:T04107; contains Pfam profilesPF03893: Lipase 3 N-terminal region, PF01764: Lipase
AT5G55420	1.40E-03	0.76	0.88	1.10	1.48	1.44	1.67	1.16	hypothetical protein
AT3G24503	1.51E-02	0.81	0.98	1.17	1.42	1.44	1.45	1.00	aldehyde dehydrogenase (ALDH1a) identical to aldehyde dehydrogenase ALDH1a [Arabidopsis thaliana] gi|20530143|gb|AAM27004
AT4G16380	3.90E-02	0.92	0.96	1.33	1.06	1.44	1.10	0.77	expressed protein
AT1G77360	4.43E-03	0.85	0.88	1.23	1.41	1.44	1.60	1.11	pentatricopeptide (PPR) repeat-containing protein contains Pfam profile PF01535: PPR repeat
AT4G05390	1.28E-02	0.87	0.93	1.25	1.14	1.44	1.22	0.85	ferredoxin--NADP(+) reductase, putative / adrenodoxin reductase, putative strong similarity to SP|P41345 Ferredoxin--NADP reductase, root isozyme, chloroplast precursor (EC 1.18.1.2) (FNR) (Oryza sativa), ferredoxin-NADP reductase precursor [Zea mays] GI:500751
AT4G31810	2.19E-03	0.83	0.83	1.18	1.29	1.44	1.55	1.08	enoyl-CoA hydratase/isomerase family protein similar to CHY1 [gi:8572760]; contains Pfam profile PF00378 enoyl-CoA hydratase/isomerase family protein
AT1G19580	2.33E-02	0.99	0.91	1.43	1.21	1.44	1.32	0.92	bacterial transferase hexapeptide repeat-containing protein contains Pfam profile PF00132: Bacterial transferase hexapeptide (four repeats)
AT5G65550	2.96E-02	0.96	0.91	1.38	1.13	1.44	1.24	0.86	UDP-glucoronosyl/UDP-glucosyl transferase family protein contains Pfam profile: PF00201 UDP-glucoronosyl and UDP-glucosyl transferase ;similar to flavonol 3-O-glucosyltransferase (anthocyanin rhamnosyl transferase) from Petunia hybrida [SP|Q43716]
AT5G23810	2.49E-03	0.79	0.90	1.13	1.60	1.44	1.79	1.24	amino acid transporter family protein similar to amino acid carrier [Ricinus communis] GI:3293031; contains Pfam profile PF01490: Transmembrane amino acid transporter protein
AT4G35900	2.46E-02	0.86	0.92	1.24	1.21	1.44	1.31	0.91	DNA-binding protein-related weak similarity to DNA-binding factor gmlip15 [Zea mays] GI:14289167
AT3G19260	1.44E-02	0.80	1.04	1.15	1.59	1.44	1.52	1.06	longevity-assurance (LAG1) family protein similar to Alternaria stem canker resistance protein (ASC1) [Lycopersicon esculentum] GI:7688742; contains Pfam profile PF03798: Longevity-assurance protein (LAG1)
AT5G61570	1.39E-02	0.86	0.84	1.23	1.34	1.43	1.59	1.11	protein kinase family protein contains protein kinase domain, Pfam:PF00069
AT1G08920	3.80E-02	0.86	0.75	1.24	1.09	1.43	1.46	1.02	sugar transporter, putative similar to ERD6 protein [Arabidopsis thaliana] GI:3123712, sugar-porter family proteins 1 and 2 [Arabidopsis thaliana] GI:14585699, GI:14585701; contains Pfam profile PF00083: major facilitator superfamily protein
AT3G24550	2.64E-02	0.87	0.83	1.25	1.26	1.43	1.53	1.07	protein kinase family protein contains Pfam domain PF00069: Protein kinase domain
AT4G15850	5.65E-03	0.79	0.84	1.13	1.09	1.43	1.30	0.91	DEAD/DEAH box helicase, putative similar to D-E-A-D box protein [Drosophila melanogaster] GI:499204; contains Pfam profiles PF00270: DEAD/DEAH box helicase, PF00271: Helicase conserved C-terminal domain
AT2G05260	1.65E-03	0.86	0.92	1.23	1.18	1.43	1.28	0.89	lipase class 3 family protein contains Pfam profile PF01764: Lipase
AT1G75450	2.34E-02	0.83	0.86	1.18	1.76	1.43	2.05	1.44	FAD-binding domain-containing protein / cytokinin oxidase family protein similar to GB:CAA77151 from [Zea mays] (SP|Q9T0N8) (Plant J. 17 (6), 615-626 (1999))
AT3G16760	1.88E-02	0.96	0.89	1.37	1.05	1.43	1.18	0.82	tetratricopeptide repeat (TPR)-containing protein low similarity to TPR-containing protein involved in spermatogenesis TPIS [Mus musculus] GI:6272682; contains Pfam profile PF00515: tetratricopeptide repeat (TPR) domain
AT3G50440	2.80E-02	1.00	0.70	1.43	0.96	1.43	1.37	0.96	hydrolase, alpha/beta fold family protein similar to ethylene-induced esterase [Citrus sinensis] GI:14279437, polyneuridine aldehyde esterase [Rauvolfia serpentina] GI:6651393; contains Pfam profile PF00561: hydrolase, alpha/beta fold family
AT5G61590	2.32E-02	0.87	0.86	1.24	1.10	1.43	1.29	0.90	AP2 domain-containing transcription factor family protein contains Pfam PF00847: AP2 domain
AT1G76580	8.97E-03	0.86	0.76	1.23	1.36	1.43	1.79	1.25	SPL1-Related3 protein (SPL1R3) identical to cDNA partial mRNA for SPL1-Related3 protein (SPL1R3 gene) GI:6006428
AT1G78680	3.36E-02	1.03	0.83	1.47	1.14	1.43	1.37	0.96	gamma-glutamyl hydrolase (GGH1) / gamma-Glu-X carboxypeptidase / conjugase identical to SP|O65355 Gamma-glutamyl hydrolase precursor (EC 3.4.19.9) (Gamma-Glu-X carboxypeptidase) (Conjugase) (GH) (Arabidopsis thaliana)
AT1G34760	1.82E-02	1.15	0.57	1.64	0.79	1.43	1.38	0.97	14-3-3 protein GF14 omicron (GRF11) identical to SP:Q9S9Z8, 14-3-3-like protein GF14 omicron (General regulatory factor 11)(Arabidopsis thaliana)
AT3G09810	7.43E-03	0.84	0.81	1.20	1.13	1.43	1.39	0.98	isocitrate dehydrogenase, putative / NAD+ isocitrate dehydrogenase, putative strong similarity to isocitrate dehydrogenase (NAD+) GB:CAA65502 GI:3021506 [Nicotiana tabacum]
AT5G58730	3.95E-02	0.95	0.93	1.35	1.08	1.42	1.16	0.81	pfkB-type carbohydrate kinase family protein contains Pfam profile: PF00294 pfkB family carbohydrate kinase
AT1G71080	4.89E-03	0.99	0.92	1.41	1.16	1.42	1.27	0.89	expressed protein
AT5G01450	3.15E-03	0.86	0.86	1.23	1.18	1.42	1.37	0.96	expressed protein

AT4G26070	3.59E-02	0.71	0.93	1.01	1.18	1.42	1.27	0.90	mitogen-activated protein kinase kinase (MAPKK) (MKK1) (MEK1) identical to MEK1 [Arabidopsis thaliana] gi|2196704|gb|AAB97145; mitogen-activated protein kinase kinase (MAPKK) family, PMID:12119167
AT5G14440	4.71E-02	0.91	0.88	1.29	1.00	1.42	1.13	0.80	surfeit locus 2 protein-related / SURF2 protein-related contains weak hit to Pfam profile PF05477: Surfeit locus protein 2 (SURF2)
AT5G48880	2.89E-02	0.91	0.90	1.30	1.45	1.42	1.61	1.13	acetyl-CoA C-acyltransferase 1 / 3-ketoacyl-CoA thiolase 1 (PKT1) identical to 3-keto-acyl-CoA-thiolase 1 [Arabidopsis thaliana] GI:3169568
AT3G17380;AT3G	3.32E-02	0.79	0.89	1.12	1.25	1.42	1.40	0.98	[AT3G17380, meprin and TRAF homology domain-containing protein / MATH domain-containing protein similar to ubiquitin-specific protease 12 [Arabidopsis thaliana] GI:11993471; contains Pfam profile PF00917: MATH domain];[AT3G17390, S-adenosylmethionine synthetase, putative similar to S-adenosylmethionine synthetase 2 (Methionine adenosyltransferase 2, AdoMet synthetase 2) [Catharanthus roseus] SWISS-PROT:Q96552]
AT2G43820	1.84E-02	0.71	1.11	1.01	1.64	1.42	1.48	1.04	UDP-glucoronosyl/UDP-glucosyl transferase family protein contains Pfam profile: PF00201 UDP-glucoronosyl and UDP-glucosyl transferase
AT1G18800	1.09E-02	0.93	0.92	1.33	1.10	1.42	1.19	0.84	nucleosome assembly protein (NAP) family protein similar to SP|Q01105|SET protein (HLA-DR associated protein II) (PHAPII) (Phosphatase 2A inhibitor I2PP2A) [Homo sapiens]; contains Pfam profile PF00956: Nucleosome assembly protein (NAP)
AT4G32840	2.05E-03	0.85	0.91	1.21	1.22	1.42	1.34	0.95	phosphofructokinase family protein similar to phosphofructokinase [Amycolatopsis methanolica] GI:17432243; contains Pfam profile PF00365: Phosphofructokinase
AT3G07050	3.53E-02	0.87	0.84	1.24	1.11	1.42	1.32	0.93	GTP-binding family protein contains Pfam domain, PF01926: GTPase of unknown function
AT2G18750	1.43E-02	0.86	0.90	1.22	1.31	1.42	1.46	1.03	calmodulin-binding protein similar to calmodulin-binding protein TCB60 GI:1698548 from [Nicotiana tabacum]
AT3G52500	3.20E-02	0.87	0.73	1.24	1.00	1.42	1.37	0.97	aspartyl protease family protein contains Pfam PF00026: eukaryotic aspartyl protease
AT5G53060	3.96E-03	0.89	0.87	1.27	1.16	1.42	1.34	0.94	KH domain-containing protein
AT5G18170	1.87E-02	0.77	1.00	1.10	1.33	1.42	1.33	0.94	glutamate dehydrogenase 1 (GDH1) identical to glutamate dehydrogenase 1 (GDH 1) [Arabidopsis thaliana] SWISS-PROT:Q43314
AT5G55930;AT5G	1.14E-02	0.66	0.96	0.93	2.15	1.42	2.24	1.58	[AT5G55930, oligopeptide transporter OPT family protein similar to SP|P40900 Sexual differentiation process protein isp4 (Schizosaccharomyces pombe), oligopeptide transporter Opt1p [Candida albicans] GI:2367386; contains Pfam profile PF03169: OPT oligopeptide transporter protein];[AT5G55940, expressed protein contains Pfam PF03665: Uncharacterised protein family (UPF0172)]
AT3G03610	4.52E-02	0.86	0.94	1.22	1.05	1.42	1.12	0.79	phagocytosis and cell motility protein ELMO1-related contains weak similarity to ELMO1 [Mus musculus] gi|16118551|gb|AAL14464
AT2G34490	3.36E-02	1.03	0.69	1.46	1.20	1.42	1.73	1.22	cytochrome P450 family protein similar to Cytochrome P450 61 (C-22 sterol desaturase) (SP:P54781) [Saccharomyces cerevisiae]; contains Pfam profile: PF00067 cytochrome P450; supported by full-length cDNA: Ceres:158108.
AT4G17310	4.62E-02	0.90	0.88	1.27	1.15	1.42	1.30	0.92	expressed protein
AT5G36880	1.42E-02	0.84	0.87	1.19	1.43	1.42	1.64	1.16	acetyl-CoA synthetase, putative / acetate-CoA ligase, putative similar to SP|P27550 (Escherichia coli) and gi:8439651 (Homo sapiens); contains Pfam AMP-binding enzyme domain PF00501
AT4G09560	2.11E-02	0.80	0.92	1.13	1.31	1.41	1.42	1.00	protease-associated zinc finger (C3HC4-type RING finger) family protein contains Pfam domain, PF02225: protease-associated (PA) domain and Pfam domain, PF00097: Zinc finger, C3HC4 type (RING finger); similar to ReMembR-H2 protein JR702 [Arabidopsis thaliana] gi|6942149|gb|AAF32326
AT2G02180	1.20E-03	0.84	0.94	1.19	1.22	1.41	1.30	0.92	tobamovirus multiplication protein 3 (TOM3) identical to tobamovirus multiplication protein (TOM3) GI:15425641 from [Arabidopsis thaliana]
AT1G31680	4.91E-02	0.93	0.86	1.32	1.42	1.41	1.65	1.16	copper amine oxidase family protein contains similarity to amine oxidase [copper-containing] precursor [Pisum sativum] SWISS-PROT:Q43077
AT1G35720	2.88E-03	1.01	0.88	1.42	1.01	1.41	1.14	0.80	annexin 1 (ANN1) identical to annexin (AnnAt1) [Arabidopsis thaliana] GI:4959106
AT3G56620	9.72E-03	0.83	0.93	1.17	1.30	1.41	1.40	0.99	integral membrane family protein / nodulin MtN21-related similar to MtN21 GI:2598575 (root nodule development) from [Medicago truncatula]
AT5G34930	1.37E-02	0.87	0.89	1.23	1.53	1.41	1.71	1.21	arogenate dehydrogenase identical to arogenate dehydrogenase GI:16903098 from [Arabidopsis thaliana]; contains Pfam profile: PF02153: prephenate dehydrogenase
AT4G32070	2.85E-02	0.91	0.85	1.29	1.49	1.41	1.76	1.24	octicosapeptide/Phox/Bem1p (PB1) domain-containing protein / tetratricopeptide repeat (TPR)-containing protein similar to SP|Q99614 Tetratricopeptide repeat protein 1 [Homo sapiens]; contains Pfam profiles PF00564: PB1 domain, PF00515: TPR Domain
AT2G24200	1.45E-03	0.84	0.83	1.18	1.43	1.41	1.73	1.22	cytosol aminopeptidase identical to cytosol aminopeptidase SP:P30184 from [Arabidopsis thaliana]; contains Pfam profiles: PF00883 cytosol aminopeptidase family catalytic domain, PF02789: cytosol aminopeptidase family N-terminal domain
AT2G31010	2.04E-02	1.01	0.70	1.43	1.17	1.41	1.66	1.18	protein kinase family protein contains protein kinase domain, Pfam:PF00069
AT5G14040	1.63E-02	0.88	0.89	1.24	1.23	1.41	1.38	0.98	mitochondrial phosphate transporter identical to mitochondrial phosphate transporter GI:3318617 from [Arabidopsis thaliana]
AT2G07240	2.06E-02	0.95	0.91	1.33	1.28	1.41	1.41	1.00	Ulp1 protease family protein contains Pfam profile PF02902: Ulp1 protease family, C-terminal catalytic domain
AT5G39190	2.78E-02	0.95	0.93	1.33	1.02	1.41	1.10	0.78	germin-like protein (GER2) identical to germin-like protein subfamily 1 member 20 [SP|P92996]
AT5G05450	1.04E-02	0.85	0.88	1.20	1.20	1.41	1.36	0.97	DEAD/DEAH box helicase, putative (RH18)
AT3G17240	1.02E-02	0.94	0.86	1.33	1.29	1.41	1.50	1.06	dihydrolipoamide dehydrogenase 2, mitochondrial / lipoamide dehydrogenase 2 (MTLPD2) nearly identical to GB:AAF34796 [gi:6984216] from [Arabidopsis thaliana]; alternative splice form exists
AT5G63980	3.15E-03	0.90	0.83	1.27	1.21	1.41	1.46	1.04	3'(2'),5'-bisphosphate nucleotidase / inositol polyphosphate 1-phosphatase / FIERY1 protein (FRY1) (SAL1) identical to SP|Q42546 3'(2'),5'-bisphosphate nucleotidase (EC 3.1.3.7) (3'(2'),5- bisphosphonucleoside 3'(2')-phosphohydrolase) (DPNPase) [Arabidopsis thaliana]; identical to cDNA inositol polyphosphate 1-phosphatase FIERY1 (FRY1) GI:15281147
AT5G09890	2.19E-02	0.90	0.76	1.27	1.23	1.40	1.61	1.15	protein kinase, putative contains protein kinase domain, Pfam:PF00069
AT3G45040	1.62E-02	0.82	0.84	1.15	1.30	1.40	1.56	1.11	phosphatidate cytidylyltransferase family protein weak similarity to SP|P20048 Dolichol kinase (EC 2.7.1.108) (Saccharomyces cerevisiae); contains Pfam profile: PF01148 phosphatidate cytidylyltransferase
AT2G48010	8.46E-03	0.91	0.90	1.27	1.34	1.40	1.48	1.06	serine/threonine protein kinase (RFK3) identical to receptor-like serine/threonine kinase [Arabidopsis thaliana] gi|2465927|gb|AAC50045
AT5G63490	1.69E-02	0.78	1.00	1.09	1.31	1.40	1.32	0.94	CBS domain-containing protein / octicosapeptide/Phox/Bemp1 (PB1) domain-containing protein contains Pfam profiles: PF00571 CBS domain, PF00564: PB1 domain
AT4G00370;AT4G	4.71E-02	0.83	0.98	1.16	1.21	1.40	1.24	0.89	[AT4G00370, sugar transporter family protein contains Pfam profile PF00083: major facilitator superfamily protein];[AT4G00360, cytochrome P450, putative]
AT3G13050	1.67E-02	0.77	0.91	1.07	1.21	1.40	1.32	0.94	transporter-related low similarity to apical organic cation transporter [Sus scrofa] GI:2062135, SP|Q02563 Synaptic vesicle protein 2 (SV2) [Rattus norvegicus]; contains Pfam profile PF00083: major facilitator superfamily protein
AT3G06300	6.75E-04	0.76	0.96	1.07	1.40	1.40	1.45	1.04	oxidoreductase, 2OG-Fe(II) oxygenase family protein similar to prolyl 4-hydroxylase, alpha subunit, from Rattus norvegicus [GI:474940], Mus musculus [SP|Q60715], Homo sapiens [GI:18073925]; contains PF03171 2OG-Fe(II) oxygenase superfamily domain
AT2G44730	1.31E-02	0.69	1.06	0.97	1.33	1.40	1.25	0.89	expressed protein similar to 6b-interacting protein 1 (NtSIP1) [Nicotiana tabacum] GI:18149189
AT2G23790	3.86E-02	1.01	0.82	1.41	1.35	1.40	1.64	1.17	AMP-binding protein, putative similar to AMP-binding protein GB:CAA96521 from [Brassica napus] (Plant Mol. Biol. (1997) 33 (5), 911-922); contains Pfam AMP-binding enzyme domain PF00501; identical to cDNA acyl-CoA synthetase-like protein GI:20799732
AT5G57190	3.43E-03	0.77	0.85	1.08	1.49	1.40	1.76	1.26	phosphatidylserine decarboxylase, putative similar to SP|P53037 Phosphatidylserine decarboxylase proenzyme 2 precursor (EC 4.1.1.65) (Saccharomyces cerevisiae); contains Pfam profile PF02666: phosphatidylserine decarboxylase
AT1G55820	1.00E-02	0.89	0.97	1.25	1.38	1.40	1.43	1.02	hydroxyproline-rich glycoprotein family protein
AT2G38960	1.70E-03	0.66	1.11	0.93	1.64	1.40	1.48	1.06	endoplasmic reticulum oxidoreductin 1 (ERO1) family protein contains Pfam domain, PF04137: Endoplasmic Reticulum Oxidoreductin 1 (ERO1)
AT3G50140	1.91E-02	0.85	0.93	1.19	1.34	1.40	1.44	1.03	expressed protein contains Pfam profile PF03140: Plant protein of unknown function
AT5G58660	5.64E-03	0.91	0.87	1.27	1.14	1.40	1.31	0.94	oxidoreductase, 2OG-Fe(II) oxygenase family protein similar to ACC oxidase, Lycopersicon esculentum [SP|P05116], gibberellin 3B-hydroxylase, Latuca sativa [gi:4164145]; contains Pfam domain PF03171, 2OG-Fe(II) oxygenase superfamily
AT5G47730	9.08E-03	0.73	0.94	1.03	1.39	1.40	1.47	1.05	SEC14 cytosolic factor, putative / polyphosphoinositide-binding protein, putative similar to polyphosphoinositide binding protein SEC14 homolog Ssh1p [GI:2739044] [Glycine max]
AT5G10190	3.53E-02	0.76	0.97	1.06	1.18	1.40	1.21	0.87	transporter-related low similarity to spinster membrane proteins from [Drosophila melanogaster] GI:12003974, GI:12003976, GI:12003972, GI:12003970; contains Pfam profile PF00083: major facilitator superfamily protein
AT1G64220	3.19E-02	1.00	0.88	1.40	1.02	1.40	1.17	0.83	preprotein translocase-related similar to TOM7 protein [Solanum tuberosum] GI:3319774
AT5G42900	4.30E-03	1.06	0.65	1.48	0.89	1.40	1.38	0.98	expressed protein similar to unknown protein (pir||T05226)
AT5G24760	1.27E-02	0.79	0.93	1.11	1.25	1.40	1.35	0.96	alcohol dehydrogenase, putative similar to alcohol dehydrogenase from Solanum tuberosum [SP|p14673]; contains Pfam zinc-binding dehydrogenase domain PF00107
AT1G27980	5.78E-03	0.89	0.77	1.24	1.22	1.40	1.59	1.14	pyridoxal-dependent decarboxylase family protein similar to sphingosine-1-phosphate lyase [Homo sapiens] GI:10129683; contains Pfam profile PF00282: Pyridoxal-dependent decarboxylase conserved domain
AT1G58360	1.18E-02	0.97	0.70	1.36	1.05	1.40	1.50	1.08	amino acid permease I (AAP1) identical to amino acid permease I GI:22641 from [Arabidopsis thaliana]
AT2G35550	2.79E-02	0.64	1.08	0.89	1.33	1.39	1.23	0.89	expressed protein
AT5G13610	9.22E-04	0.87	0.84	1.21	1.17	1.39	1.40	1.00	expressed protein
ATMG00180	4.33E-02	0.96	0.78	1.33	1.12	1.39	1.43	1.03	ccb452 cytochrome c biogenesis orf452
AT2G01580	4.51E-02	0.91	0.91	1.27	1.14	1.39	1.26	0.90	expressed protein and genefinder
AT1G73480	2.50E-02	0.75	1.00	1.04	1.49	1.39	1.49	1.07	hydrolase, alpha/beta fold family protein low similarity to monoglyceride lipase from [Homo sapiens] GI:14594904, [Mus musculus] GI:2632162; contains Pfam profile PF00561: hydrolase, alpha/beta fold family
AT5G54870	4.79E-02	0.91	0.94	1.27	1.05	1.39	1.11	0.80	short-chain dehydrogenase/reductase (SDR) family protein C-terminal similar to dormancy related protein GI:1220178 from [Trollius ledebourii]
AT5G57490	4.22E-04	0.97	0.90	1.35	1.14	1.39	1.27	0.91	porin, putative similar to 36kDA porin II [Solanum tuberosum] GI:515360; contains Pfam profile PF01459: Eukaryotic porin
AT4G21790	9.35E-04	0.87	0.92	1.21	1.33	1.39	1.45	1.04	transmembrane protein-related (TOM1) contains some similarity to transmembrane protein TOM3 GI:15425641 from [Arabidopsis thaliana]; identical to cDNA TOM1 GI:9967414
AT1G78240	2.42E-02	0.91	0.79	1.26	1.24	1.39	1.57	1.13	dehydration-responsive protein-related similar to early-responsive to dehydration stress ERD3 protein [Arabidopsis thaliana] GI:15320410; contains Pfam profile PF03141: Putative methyltransferase
AT1G72340	3.50E-02	0.90	0.93	1.25	1.02	1.39	1.10	0.79	eukaryotic translation initiation factor 2B family protein / eIF-2B family protein similar to SP|Q64270 Translation initiation factor eIF-2B alpha subunit (Rattus norvegicus); contains Pfam profile PF01008: Initiation factor 2 subunit family
AT3G47340	8.83E-03	0.93	0.75	1.29	1.16	1.39	1.55	1.11	asparagine synthetase 1 [glutamine-hydrolyzing] / glutamine-dependent asparagine synthetase 1 (ASN1) identical to SP|P49078 Asparagine synthetase [glutamine-hydrolyzing] (EC 6.3.5.4) (Glutamine- dependent asparagine synthetase) (Arabidopsis thaliana)
AT1G01650	9.74E-03	0.97	0.85	1.35	1.24	1.39	1.47	1.06	protease-associated (PA) domain-containing protein contains protease associated (PA) domain, Pfam:PF02225
AT3G17250	2.96E-04	0.90	0.89	1.25	1.14	1.39	1.28	0.92	protein phosphatase 2C-related / PP2C-related similar to protein phosphatase-2C GB:AAC36698 from [Mesembryanthemum crystallinum]
AT5G14890	4.27E-02	0.99	0.92	1.38	1.06	1.39	1.16	0.83	NHL repeat-containing protein contains Pfam profile PF01436: NHL repeat
AT1G70290	2.68E-02	0.85	0.91	1.19	1.22	1.39	1.35	0.97	trehalose-6-phosphate synthase, putative similar to Alpha,alpha-trehalose-phosphate synthase [UDP-forming] (EC 2.4.1.15) (Trehalose-6-phosphate synthase) from (Kluyveromyces lactis) SP|Q07158, (Saccharomyces cerevisiae) SP|Q00764; contains Pfam profiles PF00982: Glycosyltransferase family 20, PF02358: Trehalose-phosphatase
AT3G14090	3.65E-03	0.88	0.80	1.22	1.34	1.39	1.69	1.22	exocyst subunit EXO70 family protein contains Pfam domain PF03081: Exo70 exocyst complex subunit;
AT2G46520	8.37E-03	0.63	1.12	0.87	2.03	1.39	1.82	1.31	cellular apoptosis susceptibility protein, putative / importin-alpha re-exporter, putative similar to cellular apoptosis susceptibility protein [Homo sapiens] GI:3598795, SP|Q9ERK4 Importin-alpha re-exporter (Chromosome segregation 1-like protein) (Cellular apoptosis susceptibility protein) [Mus musculus]; contains Pfam profiles PF03810: Importin-beta N-terminal domain, PF03378: CAS/CSE protein C-terminus
AT1G08830	4.40E-02	0.89	0.94	1.23	1.14	1.39	1.21	0.87	superoxide dismutase [Cu-Zn] (SODCC) / copper/zinc superoxide dismutase (CSD1) identical to SWISS-PROT: P24704
AT3G05220	1.65E-02	0.94	0.90	1.31	1.02	1.39	1.14	0.82	heavy-metal-associated domain-containing protein similar to farnesylated protein 1 (GI:23304413) [Hordeum vulgare subsp. spontaneum]; contains Pfam profile PF00403: Heavy-metal-associated domain
AT4G29980	1.27E-02	0.92	0.94	1.28	1.17	1.39	1.24	0.90	expressed protein
AT3G06930	3.09E-03	0.86	0.92	1.20	1.26	1.39	1.37	0.99	protein arginine N-methyltransferase family protein similar to protein arginine methyltransferase [Mus musculus] GI:5257221
AT2G30590	1.67E-03	0.87	0.85	1.20	1.23	1.38	1.45	1.05	WRKY family transcription factor
AT3G23600	3.58E-02	0.94	0.87	1.29	1.00	1.38	1.16	0.84	dienelactone hydrolase family protein similar to SP|Q8ZT66 Endo-1,3;1,4-beta-D-glucanase precursor (EC 3.2.1.-) (Zea mays); contains Pfam profile: PF01738 Dienelactone hydrolase family
AT3G17810	1.92E-02	0.94	0.85	1.30	1.04	1.38	1.23	0.89	dihydroorotate dehydrogenase family protein / dihydroorotate oxidase family protein low similarity to SP|Q12882 Dihydropyrimidine dehydrogenase [NADP+] precursor (EC 1.3.1.2) (DPD) (DHPDHase) (Dihydrouracil dehydrogenase) (Dihydrothymine dehydrogenase) [Homo sapiens]; contains Pfam profile PF01180: Dihydroorotate dehydrogenase
AT5G25880	1.39E-03	0.83	0.89	1.15	1.16	1.38	1.31	0.94	malate oxidoreductase, putative similar to NADP-dependent malic enzyme (EC 1.1.1.40) (NADP-ME) (SP:P12628) [Phaseolus vulgaris]
AT1G53270	1.09E-02	0.87	0.90	1.21	1.20	1.38	1.32	0.96	ABC transporter family protein similar to ABC transporter GI:10280532 from [Homo sapiens]
AT5G13225	5.96E-04	0.78	0.89	1.08	1.42	1.38	1.59	1.15	WW domain-containing protein contains Pfam profile PF00397: WW domain
AT5G53930	3.75E-02	0.86	0.91	1.19	1.30	1.38	1.43	1.04	protein kinase family protein contains eukaryotic protein kinase domain, INTERPRO:IPR000719
AT1G78560	1.70E-03	0.88	0.90	1.22	1.35	1.38	1.49	1.08	bile acid:sodium symporter family protein low similarity to SP|Q12908 Ileal sodium/bile acid cotransporter [Homo sapiens]; contains Pfam profile PF01758: Sodium Bile acid symporter family
AT2G45290	1.27E-02	0.72	1.13	0.99	2.09	1.38	1.85	1.34	transketolase, putative strong similarity to transketolase 1 [Capsicum annuum] GI:3559814; contains Pfam profiles PF02779: Transketolase, pyridine binding domain, PF02780: Transketolase, C-terminal domain, PF00456: Transketolase, thiamine diphosphate binding domain
AT5G07350	1.73E-03	0.85	0.80	1.18	1.32	1.38	1.65	1.20	tudor domain-containing protein / nuclease family protein contains Pfam domains PF00567: Tudor domain and PF00565: Staphylococcal nuclease homologue
AT5G46250	2.96E-03	0.96	0.97	1.32	1.29	1.38	1.33	0.96	RNA recognition motif (RRM)-containing protein contains similarity to RNA-binding protein; contains InterPro entry IPR000504: RNA-binding region RNP-1 (RNA recognition motif) (RRM)
AT5G40740	2.43E-02	0.86	0.82	1.18	1.11	1.38	1.35	0.98	expressed protein
AT5G48970	1.01E-02	0.86	0.85	1.18	1.10	1.38	1.29	0.94	mitochondrial substrate carrier family protein contains Pfam profile: PF00153 mitochondrial carrier protein
AT2G44850	1.39E-04	0.65	1.15	0.89	1.58	1.38	1.38	1.00	expressed protein
AT2G32400	9.74E-03	0.85	0.91	1.17	1.42	1.38	1.56	1.14	glutamate receptor family protein (GLR3.7) (GLR5) identical to GlrS [Arabidopsis thaliana] gi|6644388|gb|AAF21042; plant glutamate receptor family, PMID:11379626
AT2G33070	2.28E-02	0.88	0.79	1.21	1.11	1.38	1.41	1.02	jacalin lectin family protein similar to myrosinase-binding protein homolog [Arabidopsis thaliana] GI:2997767, epithiospecifier [Arabidopsis thaliana] GI:16118845; contains Pfam profiles PF01419 jacalin-like lectin family, PF01344 Kelch motif
AT3G59410	7.96E-03	0.80	0.95	1.10	1.32	1.38	1.39	1.01	protein kinase family protein low similarity to GCN2 eIF2alpha kinase [Mus musculus] GI:6066585; contains Pfam profiles PF03129: Anticodon binding domain, PF00069: Protein kinase domain
AT5G16560	4.51E-02	0.99	0.76	1.36	1.42	1.38	1.86	1.35	myb family transcription factor (KAN1) contains Pfam profile: PF00249 myb-like DNA-binding domain; identical to cDNA GARP-like putative transcription factor KANADI1 (KAN1) GI:15723590
AT4G12770	3.00E-02	0.95	0.91	1.30	1.07	1.38	1.18	0.86	auxilin-related low similarity to SP|Q27974 Auxilin (Bos taurus)
AT5G42340	3.09E-03	0.87	0.91	1.20	1.10	1.38	1.21	0.88	armadillo/beta-catenin repeat family protein / U-box domain-containing protein low similarity to immediate-early fungal elicitor protein CMPG1 [Petroselinum crispum] GI:14582200, GI:14582198; contains Pfam profiles PF04564: U-box domain, PF00514: Armadillo/beta-catenin-like repeat
AT2G32660	2.95E-02	0.93	0.88	1.28	1.02	1.38	1.16	0.84	disease resistance family protein / LRR family protein contains leucine rich-repeat domains Pfam:PF00560, INTERPRO:IPR001611; similar to Cf-4 [Lycopersicon hirsutum] gi|2808683|emb|CAA05268
AT1G78670	5.77E-03	1.00	0.73	1.37	1.03	1.38	1.40	1.02	gamma-glutamyl hydrolase, putative / gamma-Glu-X carboxypeptidase, putative / conjugase, putative similar to gamma glutamyl hydrolase GI:1679658 SP|P93164 from [Glycine max]
AT2G38500	2.89E-03	0.74	0.91	1.01	1.59	1.38	1.74	1.27	expressed protein
AT1G20970	1.32E-02	0.96	0.91	1.32	1.05	1.38	1.15	0.84	adhesin-related contains TIGRFAM TIGR01612: reticulocyte binding protein; contains TIGRFAM TIGR00864: polycystin cation channel protein; similar to fimbriae-associated protein Fap1 [Streptococcus parasanguinis] (GI:3929312)
AT4G19010	3.70E-03	0.95	0.82	1.31	1.12	1.38	1.36	0.99	4-coumarate--CoA ligase family protein / 4-coumaroyl-CoA synthase family protein similar to 4CL from Pinus taeda, gi:515503, gi:1143308; contains Pfam AMP-binding enzyme domain PF00501
AT5G12430	3.04E-03	0.90	0.76	1.23	1.22	1.37	1.60	1.16	hydroxyproline-rich glycoprotein family protein Common family member At4g25620 [Arabidopsis thaliana]
AT5G51420	3.10E-02	0.88	0.95	1.22	1.15	1.37	1.21	0.88	long-chain-alcohol O-fatty-acyltransferase family protein / wax synthase family protein contains similarity to wax synthase wax synthase - Simmondsia chinensis, PID:g5020219 similar to wax synthase [gi:5020219] from Simmondsia chinensis
AT4G24450	3.27E-02	1.03	0.65	1.42	0.96	1.37	1.48	1.08	starch excess protein-related similar to SEX1 [Arabidopsis thaliana] GI:12044358
AT2G36010	4.78E-02	0.71	1.08	0.97	1.35	1.37	1.25	0.91	E2F transcription factor-3 (E2F3) identical to E2F transcription factor-3 E2F3 [Arabidopsis thaliana] gi|10443853|gb|AAG17610
AT3G52290	6.18E-03	0.91	0.82	1.25	1.15	1.37	1.41	1.03	calmodulin-binding family protein similar to SF16 protein [Helianthus annuus] GI:560150; contains Pfam profile PF00612: IQ calmodulin-binding motif
AT5G17700	1.10E-02	0.94	0.87	1.29	1.07	1.37	1.24	0.90	MATE efflux family protein similar to ripening regulated protein DDTFR18 [Lycopersicon esculentum] GI:12231296; contains Pfam profile PF01554: Uncharacterized membrane protein family
AT4G29000	3.50E-02	0.85	0.90	1.17	1.32	1.37	1.47	1.07	tesmin/TSO1-like CXC domain-containing protein similar to CXC domain containing TSO1-like protein 1 (SOL1) [Arabidopsis thaliana] GI:7767427, SP|Q9Y4I5 Tesmin (Metallothionein-like 5, testis-specific) [Homo sapiens]; contains Pfam profile PF03638: Tesmin/TSO1-like CXC domain
AT1G28230	9.37E-03	0.96	0.76	1.32	1.21	1.37	1.60	1.16	purine permease (PUP1) identical to purine permease GI:7620007 from [Arabidopsis thaliana]
AT5G11800	1.28E-02	0.84	0.86	1.15	1.24	1.37	1.45	1.06	K+ efflux antiporter, putative (KEA6) Monovalent cation:proton antiporter family 2 (CPA2 family) member, PMID:11500563; similar to glutathione-regulated potassium-efflux system protein KEFB, Escherichia coli, SWISSPROT:P45522
AT4G17480	6.86E-03	0.83	0.81	1.14	1.23	1.37	1.51	1.10	palmitoyl protein thioesterase family protein
AT5G61810	8.97E-03	0.91	0.88	1.25	1.37	1.37	1.56	1.14	mitochondrial substrate carrier family protein similar to peroxisomal Ca-dependent solute carrier, Oryctolagus cuniculus,GI:2352427; contains INTERPRO:IPR001993 Mitochondrial substrate carrier family, INTERPRO:IPR002048 calcium-binding EF-hand domain
AT1G62280	3.20E-02	0.89	0.96	1.22	1.12	1.37	1.16	0.84	C4-dicarboxylate transporter/malic acid transport family protein contains Pfam profile PF03595: C4-dicarboxylate transporter/malic acid transport protein
AT4G10930	2.00E-02	0.84	0.82	1.15	1.30	1.37	1.59	1.16	expressed protein
AT1G59970	4.51E-02	0.79	1.05	1.08	1.33	1.37	1.26	0.92	matrixin family protein similar to SP|P29136 Metalloendoproteinase 1 precursor (EC 3.4.24.-) (SMEP1) [Glycine max]; contains InterPro accession IPR001818: Matrixin
AT1G28570	4.55E-02	0.83	0.97	1.13	1.22	1.37	1.25	0.91	GDSL-motif lipase, putative similar to lipase [Arabidopsis thaliana] GI:1145627; contains InterPro Entry IPR001087 Lipolytic enzyme, G-D-S-L family
AT1G17430	1.28E-02	0.92	0.78	1.25	1.20	1.37	1.54	1.12	hydrolase, alpha/beta fold family protein low similarity to SP|Q02104 Lipase 1 precursor (EC 3.1.1.3) (Triacylglycerol lipase) {Psychrobacter immobilis}; contains Pfam profile PF00561: hydrolase, alpha/beta fold family
AT1G75840;AT1G	7.06E-03	0.96	0.90	1.31	1.06	1.37	1.18	0.86	[AT1G75840, Rac-like GTP-binding protein (ARAC5) / Rho-like GTP-binding protein (ROP4) identical to RAC-like GTP-binding protein (ARAC5) SP:Q38937 [Arabidopsis thaliana], Rho-like GTP binding protein [Arabidopsis thaliana] GI:2654009; identical to cDNA Rho-like GTP binding protein (Rop4) GI:2654008];[AT1G20090, Rac-like GTP-binding protein (ARAC4) / Rho-like GTP-binding protein (ROP2) identical to SP:Q38919 RAC-like GTP binding protein ARAC4 (GTP binding protein ROP2) [Arabidopsis thaliana], Rho-like GTP binding protein [Arabidopsis thaliana] GI:2654009]
AT4G34030	2.50E-02	0.94	0.80	1.29	1.10	1.37	1.38	1.01	methylcrotonyl-CoA carboxylase beta chain, mitochondrial / 3-methylcrotonyl-CoA carboxylase 2 (MCCB) identical to SP|Q9LDD8 Methylcrotonyl-CoA carboxylase beta chain, mitochondrial precursor (EC 6.4.1.4) (3-Methylcrotonyl-CoA carboxylase 2) (MCCase beta subunit) (3-methylcrotonyl-CoA:carbon dioxide ligase beta subunit) [Arabidopsis thaliana]; contains Pfam profile PF01039 carboxyl transferase domain
AT5G19860	1.67E-03	0.91	0.79	1.25	1.03	1.37	1.30	0.95	basic helix-loop-helix (bHLH) family protein contains Pfam profile: PF00010 helix-loop-helix DNA-binding domain
AT5G48080	2.27E-02	0.93	0.90	1.27	1.06	1.37	1.18	0.86	expressed protein ; expression supported by MPSS
AT1G60090	4.16E-02	0.89	0.93	1.21	1.15	1.37	1.24	0.91	glycosyl hydrolase family 1 protein contains Pfam PF00232 : Glycosyl hydrolase family 1 domain; TIGRFAM TIGR01233: 6-phospho-beta-galactosidase; similar to hydroxyisourate hydrolase (GI:19569603) [Glycine max]
AT5G13390	1.44E-02	0.96	0.84	1.31	1.00	1.37	1.19	0.87	nitrogen fixation NifU-like family protein similar to HIRA-interacting protein 5 (NIFU FAMILY) (SP:Q9QZ23) [Mus musculus]; contains Pfam profile: PF01106 NifU-like domain
AT1G24100	1.10E-02	0.76	0.93	1.04	1.37	1.36	1.47	1.08	UDP-glucoronosyl/UDP-glucosyl transferase family protein contains Pfam profile: PF00201 UDP-glucoronosyl and UDP-glucosyl transferase
AT2G35180	3.75E-02	0.86	0.93	1.17	1.21	1.36	1.31	0.96	amino acid transporter family protein similar to amino acid permease 1 GI:976402 from [Nicotiana sylvestris]; contains Pfam profile PF01490: Transmembrane amino acid transporter protein
AT3G20370	3.30E-02	0.95	0.88	1.29	1.11	1.36	1.27	0.93	meprin and TRAF homology domain-containing protein / MATH domain-containing protein similar to ubiquitin-specific protease 12 [Arabidopsis thaliana] GI:11993471; contains Pfam profile PF00917: MATH domain
AT1G60610	2.17E-02	0.98	0.91	1.33	1.15	1.36	1.26	0.93	expressed protein
AT5G27600	4.64E-02	0.90	0.93	1.22	1.28	1.36	1.37	1.01	AMP-binding protein, putative similar to AMP-binding protein (MF39P) gi:1617274 from Brassica napus, long-chain-fatty-acid--CoA ligase - Brassica napus, EMBL:Z72152; contains Pfam AMP-binding enzyme domain PF00501
AT4G23910	1.98E-02	0.96	0.85	1.30	1.05	1.36	1.24	0.91	expressed protein various predicted proteins, Arabidopsis thaliana
AT5G25900	9.48E-03	0.88	0.87	1.21	1.15	1.36	1.33	0.98	ent-kaurene oxidase, putative (GA3) / cytochrome P450 identical to GA3 [Arabidopsis thaliana] GI:3342249; similar to ent-kaurene oxidase [Cucurbita maxima] GI:11934675; contains Pfam profile PF00067: Cytochrome P450
AT5G58690	1.69E-02	0.94	0.84	1.28	1.08	1.36	1.29	0.95	phosphoinositide-specific phospholipase C, putative similar to phosphoinositide specific phospholipase C GI:857374 from [Arabidopsis thaliana]
AT5G26270	2.95E-02	0.70	1.05	0.96	1.28	1.36	1.22	0.89	expressed protein ; expression supported by MPSS
AT2G16640	2.53E-02	0.84	0.80	1.14	1.22	1.36	1.52	1.11	chloroplast outer membrane protein, putative similar to chloroplast protein import component Toc159 [Pisum sativum] GI:8489806, chloroplast outer envelope protein 86 [Pisum sativum] GI:599958, GTP-binding protein [Pisum sativum] GI:576509
AT1G15520	2.85E-02	0.73	1.05	1.00	1.63	1.36	1.56	1.14	ABC transporter family protein similar to ABC1 protein GI:14331118 from [Nicotiana plumbaginifolia]
AT2G19880	1.01E-02	0.84	0.82	1.14	1.34	1.36	1.62	1.19	ceramide glucosyltransferase, putative similar to ceramide glucosyltransferase [GI:14718995] [Gossypium arboreum]; weak similarity to Ceramide glucosyltransferase (Glucosylceramide synthase) (GCS) (UDP-glucose:N-acylsphingosine D-glucosyltransferase) (GLCT-1) (Swiss-Prot:Q16739) [Homo sapiens]
AT1G06590	8.21E-03	0.83	0.97	1.14	1.43	1.36	1.48	1.09	expressed protein
AT4G24010	1.32E-03	0.86	0.85	1.17	1.16	1.36	1.36	1.00	cellulose synthase family protein similar to Zea mays cellulose synthase-5 [gi:9622882], -4 [gi:9622880]
AT1G03110	1.91E-02	0.87	0.93	1.19	1.09	1.36	1.17	0.86	transducin family protein / WD-40 repeat family protein contains 4 WD-40 repeats (PF00400); similar to WD-repeat domain 4 protein (GI:9955698) [Mus musculus]
AT5G59520	9.72E-03	0.89	0.92	1.22	1.15	1.36	1.25	0.92	zinc transporter (ZIP2) identical to zinc transporter ZIP2 [Arabidopsis thaliana] gi|3252868|gb|AAC24198; member of the Zinc (Zn2+)-Iron (Fe2+) permease (ZIP) family, PMID:11500563
AT5G48780	1.23E-02	0.82	0.84	1.11	1.12	1.36	1.34	0.98	hypothetical protein
AT3G05550	1.86E-02	0.86	0.94	1.17	1.12	1.36	1.19	0.87	zinc finger (C3HC4-type RING finger) family protein contains zinc finger domain, C3HC4 type (RING finger) 152633.
AT2G46640	2.34E-02	0.73	1.09	0.99	1.57	1.36	1.44	1.06	hypothetical protein and genefinder; expression supported by MPSS
AT2G17130	2.06E-02	0.84	0.82	1.15	1.17	1.36	1.42	1.05	isocitrate dehydrogenase subunit 2 / NAD+ isocitrate dehydrogenase subunit 2 nearly identical to NAD+ dependent isocitrate dehydrogenase subunit 2 [Arabidopsis thaliana] GI:1766048
AT1G53570	3.45E-03	0.93	0.74	1.26	1.48	1.36	1.99	1.47	mitogen-activated protein kinase kinase kinase (MAPKKK), putative (MAP3Ka) identical to MEK kinase (MAP3Ka)[Arabidopsis thaliana] gi|4204912|gb|AAD10848
AT3G10050	4.51E-02	0.88	0.90	1.19	1.21	1.36	1.34	0.99	threonine ammonia-lyase / threonine dehydratase / threonine deaminase (OMR1) identical to SP|Q9ZSS6 Threonine dehydratase biosynthetic, chloroplast precursor (EC 4.3.1.19, formerly EC 4.2.1.16) (Threonine deaminase) (TD) [Arabidopsis thaliana]
AT3G57360	1.86E-02	0.96	0.85	1.30	1.06	1.36	1.24	0.92	expressed protein
AT1G76470	3.63E-03	0.87	0.88	1.18	1.78	1.36	2.01	1.48	cinnamoyl-CoA reductase family similar to cinnamoyl-CoA reductase GB:CAA56103 [Eucalyptus gunnii], Pinus taeda [GI:17978649]; contains non-consensus GG acceptor splice site at exon 4
AT1G75800	7.53E-04	0.92	0.80	1.25	1.18	1.36	1.48	1.09	pathogenesis-related thaumatin family protein similar to receptor serine/threonine kinase PR5K [Arabidopsis thaliana] GI:1235680; contains Pfam profile: PF00314 Thaumatin family
AT2G29340	3.78E-02	0.69	1.08	0.94	1.38	1.36	1.29	0.95	short-chain dehydrogenase/reductase (SDR) family protein similar to tropinone reductase-I GI:424160 from [Datura stramonium]
AT2G24170	2.61E-02	0.95	0.77	1.23	0.99	1.36	1.28	0.95	glycosyl transferase family 8 protein contains Pfam profile: PF01501 glycosyl transferase family 8
AT3G50310	4.29E-02	0.85	0.88	1.15	1.34	1.35	1.52	1.12	protein kinase-related contains eukaryotic protein kinase domain, INTERPRO:IPR000719
AT2G41330	1.10E-02	0.78	0.97	1.06	1.20	1.35	1.23	0.91	glutaredoxin family protein contains Pfam profile PF00462: Glutaredoxin
AT4G03210	4.39E-02	1.11	0.81	1.51	1.05	1.35	1.29	0.96	xyloglucan:xyloglucosyl transferase, putative / xyloglucan endotransglycosylase, putative / endo-xyloglucan transferase, putative contains similarity to xyloglucan endo-transglycosylase-like protein (XET-1) GI:5070246 from [Medicago truncatula]
AT5G42320	4.66E-03	0.90	0.88	1.22	1.19	1.35	1.36	1.01	zinc carboxypeptidase family protein contains Pfam domain, PF00246: Zinc carboxypeptidase
AT2G45940	3.61E-03	0.85	0.90	1.16	1.20	1.35	1.33	0.98	hypothetical protein contains Pfam profile PF03478: Protein of unknown function (DUF295)
AT2G01190	7.21E-03	0.89	0.86	1.20	1.50	1.35	1.75	1.29	octicosapeptide/Phox/Bemp1p (PB1) domain-containing protein Pfam profile PF00564: PB1 domain
AT5G05520	1.34E-02	0.85	0.88	1.15	1.21	1.35	1.38	1.02	outer membrane OMP85 family protein contains Pfam profile PF01103: outer membrane protein, OMP85 family
AT5G47540	1.10E-02	0.97	0.93	1.32	1.02	1.35	1.09	0.81	Mo25 family protein similar to MO25 protein (early mouse development protein family) [Mouse] SWISS-PROT:Q06138
AT5G04010	4.05E-02	0.96	0.94	1.30	1.18	1.35	1.26	0.93	expressed protein
AT1G80670	7.52E-03	0.77	0.96	1.04	1.13	1.35	1.17	0.87	transducin family protein / WD-40 repeat family protein contains 5 WD-40 repeats (PF00400) (1 weak); similar to Hypothetical RAE1-like protein.(SP:Q38942) [Arabidopsis thaliana]; similar to mRNA-associated protein mrnp 41 ((mRNA export protein) (GB:AAC28126) (GI:1903456)(RAE1) (MRNP41) (SP:P78406) [Homo sapiens]
AT1G65430	1.38E-03	0.95	0.79	1.28	1.17	1.35	1.49	1.10	zinc finger protein-related contains weak similarity to zinc finger proteins and a Pfam:PF01485 IBR domain
AT2G37360	4.07E-02	0.91	0.93	1.22	1.14	1.35	1.24	0.92	ABC transporter family protein
AT5G16220	1.21E-02	0.93	0.83	1.26	1.27	1.35	1.53	1.14	octicosapeptide/Phox/Bemp1p (PB1) domain-containing protein hypothetical proteins - Arabidopsis thaliana contains Pfam profile PF00564: PB1 domain
AT5G05040	2.74E-02	0.90	0.97	1.21	1.00	1.35	1.04	0.77	senescence-associated protein-related similar to senescence-associated protein SAG102 (GI:22331931) [Arabidopsis thaliana]
AT4G02930	1.27E-02	0.90	0.86	1.21	1.19	1.35	1.39	1.03	elongation factor Tu, putative / EF-Tu, putative similar to mitochondrial elongation factor Tu [Arabidopsis thaliana] gi|1149571|emb|CAA61511
AT1G17250	3.91E-02	0.71	1.02	0.95	1.27	1.35	1.24	0.92	leucine-rich repeat family protein contains leucine rich-repeat (LRR) domains Pfam:PF00560, INTERPRO:IPR001611; similar to Hcr2-0B [Lycopersicon esculentum] gi|3894387|gb|AAC78593
AT3G26090	9.00E-03	0.89	0.90	1.20	1.34	1.35	1.49	1.10	expressed protein
AT2G34140	4.43E-02	0.94	0.85	1.27	1.13	1.35	1.34	0.99	Dof-type zinc finger domain-containing protein
AT3G13730	2.97E-02	0.76	0.95	1.02	1.43	1.35	1.50	1.12	cytochrome P450, putative similar to Cytochrome P450 90C1 (ROTUNDIFOLIA3) (SP:Q9M066) [Arabidopsis thaliana]; identical to CYP90D (GI:14971017) [Arabidopsis thaliana]
AT2G42070;AT2G	1.83E-02	0.89	0.97	1.20	1.26	1.35	1.29	0.96	[AT2G42070, MutT/nudix family protein similar to SP|Q58549 ADP-ribose pyrophosphatase (EC 3.6.1.13) (Methanococcus jannaschii); contains Pfam profile PF00293: NUDIX domain];[AT2G42060, CHP-rich zinc finger protein, putative]
AT5G04310	2.21E-02	0.94	0.97	1.26	1.22	1.35	1.25	0.93	pectate lyase family protein similar to pectate lyase GP:14531296 from [Fragaria x ananassa]
AT5G01780	4.30E-03	0.99	0.89	1.34	1.05	1.35	1.18	0.88	oxidoreductase, 2OG-Fe(II) oxygenase family protein low similarity to alkB protein - Escherichia coli, PIR:BVECKB, alkB [Caulobacter crescentus][GI:2055386]; contains Pfam domain PF03171 2OG-Fe(II) oxygenase superfamily
AT5G66050	1.39E-02	1.06	0.81	1.43	0.98	1.35	1.20	0.89	expressed protein
AT3G27960	4.30E-02	0.96	0.77	1.30	1.14	1.35	1.48	1.10	kinesin light chain-related low similarity to kinesin light chain [Plectonema boryanum] GI:2645229; contains Pfam profile PF00515: TPR Domain
AT5G60410	3.13E-02	0.82	0.92	1.10	1.51	1.35	1.63	1.21	DNA-binding family protein contains Pfam profiles: PF02037 SAP domain, PF02891 MIZ zinc finger, PF00628 PHD-finger
AT1G19160	3.48E-02	0.86	0.93	1.15	1.01	1.35	1.19	0.88	F-box family protein-related contains weak hit to TIGRFAM TIGR01640 : F-box protein interaction domain
AT3G55560	6.40E-03	0.74	1.02	1.00	1.37	1.34	1.34	0.99	DNA-binding protein-related contains Pfam PF03479: Domain of unknown function (DUF296), found in AT-hook motifs Pfam:PF02178
AT1G13830	3.76E-02	0.88	0.92	1.18	1.08	1.34	1.18	0.88	beta-1,3-glucanase-related similar to beta-1,3-glucanase-like protein [GI:14279169] [Olea europaea] similar to Glucan endo-1,3-beta-glucosidase precursor (EC 3.2.1.39) ((1-3)-beta- glucan endohydrolase) ((1-3)-beta-glucanase) (Beta-1,3- endoglucanase) (Swiss-Prot:P52409) [Triticum aestivum]
AT5G07220	1.79E-02	1.00	0.77	1.34	1.04	1.34	1.34	1.00	BAG domain-containing protein contains Pfam:PF02179 BAG domain
AT2G24450	3.62E-02	0.90	0.93	1.21	1.21	1.34	1.30	0.97	fasciclin-like arabinogalactan family protein similar to fasciclin-like arabinogalactan-protein 1 [Arabidopsis thaliana] gi|13377776|gb|AAK20857
AT5G06850	4.43E-02	1.01	0.90	1.36	0.96	1.34	1.07	0.79	ubiquitin-associated (UBA)/TS-N domain-containing protein weak similarity to SP|P45974 Ubiquitin carboxyl-terminal hydrolase 5 (EC 3.1.2.15) [Homo sapiens]; contains Pfam profile PF00627: UBA/TS-N domain
AT3G15270	2.91E-02	0.79	1.01	1.06	1.47	1.34	1.46	1.09	squamosa promoter-binding protein-like 5 (SPL5) identical to squamosa promoter binding protein-like 5 [Arabidopsis thaliana] GI:5931629; contains Pfam profile PF03110: SBP domain
AT5G17210	3.54E-02	0.93	0.90	1.25	1.00	1.34	1.11	0.83	expressed protein
AT2G01390	2.56E-02	0.91	0.94	1.22	1.18	1.34	1.26	0.94	pentatricopeptide (PPR) repeat-containing protein contains Pfam profile PF01535: PPR repeat
AT2G25450	1.10E-02	0.95	0.90	1.28	1.05	1.34	1.17	0.87	2-oxoglutarate-dependent dioxygenase, putative similar to 2A6 [GI:599622] and tomato ethylene synthesis regulatory protein E8 (SP|P10967)
AT3G12740	2.91E-02	0.83	0.99	1.12	1.20	1.34	1.21	0.90	LEM3 (ligand-effect modulator 3) family protein / CDC50 family protein Similar to GI:4585976; GI:4966357; GI:4835763; GI:9757735 from [Arabidopsis thaliana]
AT1G27460	2.41E-02	0.85	0.89	1.14	1.41	1.34	1.59	1.18	calmodulin-binding protein similar to calmodulin-binding protein MPCBP [Zea mays] GI:10086260; contains Pfam profile PF00515: TPR Domain
AT2G25820	2.99E-02	0.87	0.96	1.16	1.15	1.34	1.20	0.90	transcription factor, putative similar to TINY [Arabidopsis thaliana] GI:1246403
AT2G28440	1.09E-02	0.97	0.91	1.31	1.02	1.34	1.12	0.83	proline-rich family protein contains proline-rich extensin domains, INTERPRO:IPR002965; contains similarity to vegetative cell wall protein gp1 [Chlamydomonas reinhardtii] gi|12018147|gb|AAG45420; +
AT3G54650	2.73E-03	0.90	0.74	1.21	1.30	1.34	1.49	1.11	F-box family protein (FBL17) contains similarity to F-box protein FBL2 GI:6010699 from [Rattus norvegicus]
AT4G07410	4.03E-02	0.89	0.93	1.19	1.31	1.34	1.41	1.05	transducin family protein / WD-40 repeat family protein contains 7 WD-40 repeats (PF00400) (2 weak); similar to Vegetatible incompatibility protein HET-E-1 (SP:Q00808) {Podospora anserina}
AT3G17440	2.59E-03	1.00	0.90	1.33	1.02	1.34	1.14	0.85	novel plant SNARE 13 (NPSN13) identical to Novel plant SNARE 13 (AtNPSN13) (SP:Q9LRP1) [Arabidopsis thaliana]; contains Pfam profile: PF00190 11S plant seed storage protein
AT1G28300	4.31E-02	0.92	0.91	1.23	1.05	1.34	1.16	0.87	transcriptional factor B3 family protein / leafy cotyledon 2 (LEC2) nearly identical to LEAFY COTYLEDON 2 [Arabidopsis thaliana] GI:15987516; contains Pfam profile PF02362: B3 DNA binding domain
AT3G05290	3.53E-02	1.00	0.94	1.34	1.12	1.34	1.19	0.89	mitochondrial substrate carrier family protein contains Pfam profile: PF00153 mitochondrial carrier protein
AT1G60850	2.48E-02	0.93	0.95	1.24	1.24	1.34	1.31	0.98	DNA-directed RNA polymerase, putative identical to RNA polymerase subunit [Arabidopsis thaliana] GI:514322; contains Pfam profile PF01000: RNA polymerase Rpb3/RpoA insert domain
AT3G02990	1.14E-02	0.97	0.87	1.29	1.05	1.34	1.21	0.90	heat shock factor protein 2 (HSF2) / heat shock transcription factor 2 (HSTF2) identical to heat shock transcription factor 2 (HSF2) SP:Q9S620 from [Arabidopsis thaliana]; contains Pfam profile: PF00447 HSF-type DNA-binding domain
AT5G05460	2.42E-02	0.88	0.97	1.18	1.26	1.34	1.30	0.97	glycosyl hydrolase family 85 protein contains similarity to endo-B-N-acetylglucosaminidase GI:13774138 from [Mucor hiemalis]
AT3G58620	2.28E-02	0.85	0.84	1.14	1.11	1.34	1.32	0.99	tetratricopeptide repeat (TPR)-containing protein contains Pfam profile PF00515 TPR Domain
AT5G54900	1.42E-02	0.88	0.87	1.18	1.06	1.34	1.22	0.91	RNA-binding protein 45 (RBP45), putative contains similarity to polyadenylate-binding protein 5
AT5G07680	2.13E-02	0.87	0.97	1.16	1.09	1.34	1.13	0.84	no apical meristem (NAM) family protein contains Pfam PF02365 : No apical meristem (NAM) protein; similar to cup-shaped cotyledon CUC2 (GI:1944132) [Arabidopsis thaliana]
AT4G13850	1.62E-02	0.95	0.82	1.27	1.49	1.34	1.56	1.17	glycine-rich RNA-binding protein, putative similar to Glycine-rich RNA-binding protein 2, mitochondrial precursor (AtGRP2) (Swiss-Prot:Q9SVM8) [Arabidopsis thaliana] ; contains InterPro entry IPR000504: RNA-binding region RNP-1 (RNA recognition motif) (RRM)
AT1G48530	5.58E-03	0.93	0.80	1.25	1.03	1.34	1.29	0.97	CBS domain-containing protein low similarity to SP|Q9UGI9 5'-AMP-activated protein kinase, gamma-3 subunit (AMPK gamma-3 chain) (AMPK gamma3) [Homo sapiens]; contains Pfam profile PF00571: CBS domain
AT5G63610	2.06E-02	0.89	0.87	1.19	1.08	1.34	1.24	0.93	protein kinase, putative similar to cyclin-dependent kinase cdc2MsE [Medicago sativa] gi|1806144|emb|CAA65981; contains protein kinase domain, Pfam:PF00069
AT2G26330	3.26E-02	0.98	0.92	1.30	1.15	1.33	1.24	0.93	glycosyl hydrolase family 17 protein similar to elicitor inducible chitinase Nt-SubE76 GI:11071974 from [Nicotiana tabacum]
AT3G01280	2.05E-04	0.86	0.85	1.15	1.19	1.33	1.40	1.05	porin, putative similar to SP|P42055 34 kDa outer mitochondrial membrane protein porin (Voltage-dependent anion-selective channel protein) (VDAC) [Solanum tuberosum]; contains Pfam profile PF01459: Eukaryotic porin
AT3G48890	8.25E-03	0.85	0.88	1.14	1.16	1.33	1.32	0.99	cytochrome b5 domain-containing protein similar to SP|O00264 Membrane associated progesterone receptor component (mPR) [Homo sapiens]; contains Pfam profile PF00173: Heme/Steroid binding domain
AT5G23250	2.70E-02	0.97	0.94	1.29	1.01	1.33	1.07	0.80	succinyl-CoA ligase [GDP-forming] alpha-chain, mitochondrial, putative / succinyl-CoA synthetase, alpha chain, putative / SCS-alpha, putative similar to SP|P36967 Succinyl-CoA ligase [GDP-forming] alpha-chain, mitochondrial precursor (EC 6.2.1.4) (Succinyl-CoA synthetase, alpha chain) (SCS-alpha) {Dictyostelium discoideum}; contains Pfam profiles PF00549: CoA-ligase, PF02629: CoA binding domain
AT2G36380	2.69E-03	0.91	0.91	1.21	1.26	1.33	1.39	1.05	F-box family protein / tubby family protein similar to phosphodiesterase [GI:467578] [Mus musculus]; similar to C-terminal half of tubby protein (A mutation in the tub gene causes maturity-onset obesity, insulin resistance, and sensory deficits) (GB:P50586) (GI:6730158)[Mus musculus]; contains Pfam profile PF00646: F-box domain and Pfam PF01167: Tub family; similar to Tubby protein homolog (Swiss-Prot:O88808) [Rattus norvegicus]
AT5G58120;AT5G	2.31E-02	0.96	0.91	1.28	1.15	1.33	1.27	0.95	[AT5G58120, disease resistance protein (TIR-NBS-LRR class), putative domain signature TIR-NBS-LRR exists, suggestive of a disease resistance protein.];[AT5G58130, RNA recognition motif (RRM)-containing protein]
AT1G08090	1.69E-02	0.96	0.91	1.28	1.18	1.33	1.30	0.97	high-affinity nitrate transporter (ACH1) identical to trans-membrane nitrate transporter protein AtNRT2:1 [Arabidopsis thaliana] GI:3747058, high-affinity nitrate transporter ACH1 [Arabidopsis thaliana] GI:3608362
AT2G30010	5.36E-04	0.82	0.86	1.09	1.27	1.33	1.48	1.11	expressed protein
AT5G19300	1.37E-02	0.88	0.96	1.18	1.11	1.33	1.16	0.87	expressed protein contains Pfam profile PF02598: Uncharacterized ACR, COG2106
AT3G49150	2.61E-02	0.90	0.97	1.20	1.08	1.33	1.11	0.84	F-box family protein contains F-box domain Pfam:PF00646
AT4G28550	8.12E-03	0.85	0.95	1.13	1.28	1.33	1.34	1.01	RabGAP/TBC domain-containing protein similar to SP|P09379 GTPase-activating protein GYP7 (Fragment) {Yarrowia lipolytica}; contains Pfam profile PF00566: TBC domain
AT4G13560	2.71E-02	0.99	0.81	1.31	1.02	1.33	1.26	0.94	late embryogenesis abundant domain-containing protein / LEA domain-containing protein similar to LEA protein [Cicer arietinum] GI:2909420; contains Pfam profile PF02987: Late embryogenesis abundant protein
AT4G02540	2.43E-02	0.91	0.76	1.21	1.24	1.33	1.63	1.22	DC1 domain-containing protein contains Pfam profile PF03107: DC1 domain
AT2G01850	5.51E-03	0.95	0.84	1.26	1.01	1.33	1.20	0.90	xyloglucan:xyloglucosyl transferase / xyloglucan endotransglycosylase / endo-xyloglucan transferase (EXGT-A3) identical to endoxyloglucan transferase [Arabidopsis thaliana] GI:5533313
AT3G09660	3.70E-03	0.94	0.91	1.25	1.20	1.33	1.33	1.00	minichromosome maintenance family protein / MCM family protein similar to SP|P49717 DNA replication licensing factor MCM4 (CDC21 homolog) (Mus musculus); contains Pfam profile PF00493: MCM2/3/5 family
AT3G16690	1.03E-02	0.91	0.80	1.22	1.11	1.33	1.38	1.04	nodulin MtN3 family protein similar to MtN3 GI:1619602 (root nodule development) from [Medicago truncatula]
AT3G03520	8.33E-03	0.99	0.85	1.32	1.07	1.33	1.26	0.95	phosphoesterase family protein low similarity to SP|P95246 Phospholipase C 2 precursor (EC 3.1.4.3) {Mycobacterium tuberculosis}; contains Pfam profile PF04185: Phosphoesterase family
AT5G21090	4.03E-03	0.88	0.98	1.17	1.13	1.33	1.16	0.87	leucine-rich repeat protein, putative similar to leucine rich repeat protein (LRP) GI:1619300 from [Lycopersicon esculentum]; contains leucine-rich repeat (LRR) domains Pfam:PF00560, INTERPRO:IPR001611
AT5G27120;AT5G	2.38E-02	0.92	0.91	1.22	1.14	1.33	1.25	0.94	[AT5G27120, SAR DNA-binding protein, putative strong similarity to SAR DNA-binding protein-1 [Pisum sativum] GI:3132696; contains Pfam profile PF01798: Putative snoRNA binding domain];[AT5G27140, SAR DNA-binding protein, putative strong similarity to SAR DNA-binding protein-1 [Pisum sativum] GI:3132696; contains Pfam profile PF01798: Putative snoRNA binding domain]
AT1G02170	6.23E-03	0.93	0.91	1.23	1.19	1.33	1.30	0.98	latex-abundant family protein (AMC1) / caspase family protein contains similarity to latex-abundant protein [Hevea brasiliensis] gb:AAD13216; contains Pfam profile PF00656: ICE-like protease (caspase) p20 domain
AT3G07530	1.48E-02	0.92	0.93	1.22	1.10	1.33	1.18	0.89	expressed protein ; expression supported by MPSS
AT1G60470	3.76E-02	0.95	0.87	1.27	1.10	1.33	1.26	0.95	galactinol synthase, putative similar to galactinol synthase GI:5608497 from [Ajuga reptans]
AT2G27370	4.33E-02	0.87	0.97	1.16	1.11	1.33	1.14	0.86	integral membrane family protein contains TIGRFAM TIGR01569 : plant integral membrane protein TIGR01569; contains Pfam PF04535 : Domain of unknown function (DUF588)
AT5G19410	5.53E-03	0.94	0.92	1.25	1.14	1.33	1.24	0.94	ABC transporter family protein white membrane transporter, Bactrocera tryoni, EMBL:U97104
AT1G16390	2.23E-02	1.11	0.65	1.47	0.83	1.33	1.26	0.95	organic cation transporter-related low similarity to Organic cation/carnitine transporter 2 (Solute carrier family 22, member 5) (High-affinity sodium-dependent carnitine cotransporter) from {Homo sapiens} SP|O76082, {Rattus norvegicus} SP|O70594; contains Pfam profile PF00083: major facilitator superfamily protein
AT3G20810	3.66E-03	1.18	0.60	1.56	0.96	1.32	1.61	1.21	transcription factor jumonji (jmjC) domain-containing protein contains Pfam domain PF02373: jmjC domain
AT3G45620	2.98E-02	0.94	0.88	1.24	1.07	1.32	1.23	0.92	transducin family protein / WD-40 repeat family protein contains 7 WD-40 repeats; similar to PC326 protein (GI:200241) (PIR2:S37694) [Mus musculus];Human (H326) translated mRNA - Homo sapiens, EMBL:HS06631
AT1G61720	2.65E-02	0.93	0.95	1.23	1.19	1.32	1.25	0.94	dihydroflavonol 4-reductase (dihydrokaempferol 4-reductase) family (BAN) similar to dihydroflavonol 4-reductase GI:1332411 from [Rosa hybrida]
AT5G17450	2.35E-02	0.94	0.89	1.24	1.24	1.33	1.39	1.05	heavy-metal-associated domain-containing protein / copper chaperone (CCH)-related similar to copper homeostasis factor [Arabidopsis thaliana][GI:3168840], and farnesylated proteins GMFP7 [Glycine max][GI:4097573], ATFP7 [GI:4097555], and ATFP6 [GI:4097553]; contains heavy-metal-associated domain PF00403
AT2G27900	4.80E-02	0.85	0.90	1.13	1.30	1.33	1.44	1.09	expressed protein
AT5G19100	1.27E-02	0.94	0.82	1.25	1.23	1.33	1.51	1.13	extracellular dermal glycoprotein-related / EDGP-related low similarity to extracellular dermal glycoprotein EDGP precursor [Daucus carota] GI:285741, SP|P13917 Basic 7S globulin precursor [Glycine max]
AT2G32250	1.87E-02	0.87	0.89	1.15	1.61	1.33	1.81	1.37	far-red impaired responsive protein, putative similar to far-red impaired response protein FAR1 [Arabidopsis thaliana] gi|5764395|gb|AAD51282; contains Pfam profile PF03101: FAR1 family
AT1G24180	7.83E-04	0.87	0.83	1.15	1.20	1.33	1.44	1.08	pyruvate dehydrogenase E1 component alpha subunit, mitochondrial, putative similar to SP|P52901 Pyruvate dehydrogenase E1 component alpha subunit, mitochondrial precursor (EC 1.2.4.1) (PDHE1-A) {Arabidopsis thaliana}; contains Pfam profile PF00676: Dehydrogenase E1 component
AT5G06730	1.89E-02	0.94	0.94	1.24	1.01	1.33	1.07	0.81	peroxidase, putative similar to peroxidase [Arabidopsis thaliana] gi|1491617|emb|CAA68212
AT3G43270	4.27E-02	1.02	0.77	1.35	1.05	1.33	1.36	1.03	pectinesterase family protein contains Pfam profile: PF01095 pectinesterase
AT1G68740	1.39E-03	0.97	0.86	1.28	1.10	1.33	1.28	0.97	EXS family protein / ERD1/XPR1/SYG1 family protein similar to PHO1 protein [Arabidopsis thaliana] GI:20069032; contains Pfam profiles PF03105: SPX domain, PF03124: EXS family
AT1G61690	1.37E-02	0.88	0.75	1.17	1.25	1.33	1.66	1.25	tetratricopeptide repeat (TPR)-containing protein contains Pfam profile PF00515: TPR Domain
AT1G63260	2.32E-02	1.03	0.80	1.37	1.04	1.33	1.29	0.98	senescence-associated family protein similar to senescence-associated protein 5 [Hemerocallis hybrid cultivar] gi|3551954|gb|AAC34855
AT4G36680	1.31E-02	0.91	0.84	1.21	1.17	1.32	1.39	1.05	pentatricopeptide (PPR) repeat-containing protein low similarity to fertility restorer [Petunia x hybrida] GI:22128587; contains Pfam profile PF01535: PPR repeat; identical to cDNA membrane-associated salt-inducible protein like GI:2632060
AT1G21370;AT1G	1.21E-02	0.87	0.84	1.15	1.19	1.32	1.42	1.07	[AT1G21370, expressed protein];[AT1G21380, VHS domain-containing protein / GAT domain-containing protein weak similarity to Hrs [Rattus norvegicus] GI:8547026; contains Pfam profiles PF00790: VHS domain, PF03127: GAT domain]
AT1G11840	1.39E-02	0.85	0.94	1.13	1.21	1.32	1.29	0.97	lactoylglutathione lyase, putative / glyoxalase I, putative highly similar to putative lactoylglutathione lyase GI:2113966 from [Brassica oleracea]
AT1G03090	3.95E-02	1.00	0.77	1.32	1.06	1.32	1.38	1.03	methylcrotonyl-CoA carboxylase alpha chain, mitochondrial / 3-methylcrotonyl-CoA carboxylase 1 (MCCA) nearly identical to SP|Q42523 Methylcrotonyl-CoA carboxylase alpha chain, mitochondrial precursor (EC 6.4.1.4) (3-Methylcrotonyl-CoA carboxylase 1) (MCCase alpha subunit) (3-methylcrotonyl-CoA:carbon dioxide ligase alpha subunit) [Arabidopsis thaliana]
AT1G26090	4.37E-04	0.90	0.81	1.19	1.13	1.32	1.39	1.05	hypothetical protein
AT5G04270	1.09E-02	0.92	0.95	1.21	1.10	1.32	1.19	0.90	zinc finger (DHHC type) family protein low similarity to Golgi-specific DHHC zinc finger protein [Mus musculus] GI:21728103; contains Pfam profile PF01529: DHHC zinc finger domain
AT5G26110	1.83E-02	0.91	0.95	1.20	1.11	1.32	1.16	0.88	expressed protein
AT3G56000	4.62E-03	0.81	0.91	1.07	1.35	1.32	1.48	1.12	glycosyl transferase family 2 protein similar to beta-(1-3)-glucosyl transferase GB:AAC62210 GI:3687658 from [Bradyrhizobium japonicum], cellulose synthase from Agrobacterium tumefaciens [gi:710492] and Agrobacterium radiobacter [gi:710493]; contains Pfam glycosyl transferase, group 2 family protein domain PF00535
AT2G40090	8.39E-03	0.67	1.18	0.88	1.77	1.32	1.49	1.13	ABC1 family protein contains Pfam domain, PF03109: ABC1 family
AT2G30600	8.15E-03	0.85	0.83	1.13	1.37	1.32	1.65	1.25	BTB/POZ domain-containing protein contains Pfam PF00651: BTB/POZ domain; contains Interpro IPR000210/ PS50097: BTB/POZ domain; similar to MigA (GI:1841872) [Dictyostelium discoideum]
AT5G05100	1.42E-02	1.00	0.81	1.32	0.97	1.32	1.20	0.91	expressed protein
AT5G05160	6.72E-03	0.87	0.92	1.15	1.54	1.32	1.68	1.27	leucine-rich repeat transmembrane protein kinase, putative
AT3G25920;AT3G	5.42E-03	0.95	0.86	1.25	1.08	1.32	1.26	0.95	[AT3G25920, 50S ribosomal protein L15, chloroplast (CL15) identical to GB:P25873 from [Arabidopsis thaliana]];[AT3G25910, expressed protein]
AT2G37690	2.59E-02	0.65	1.12	0.86	1.83	1.32	1.63	1.23	phosphoribosylaminoimidazole carboxylase, putative / AIR carboxylase, putative similar to SP|P55195 Phosphoribosylaminoimidazole carboxylase, chloroplast precursor (EC 4.1.1.21) (AIR carboxylase) (AIRC) [Vigna aconitifolia]; contains Pfam profiles PF02222: ATP-grasp domain, PF00731: AIR carboxylase
AT4G17270	2.36E-03	0.97	0.86	1.27	1.07	1.32	1.24	0.94	Mo25 family protein similar to MO25 protein (early mouse development protein family) [Mouse] SWISS-PROT:Q06138
AT4G02690	1.85E-03	0.79	0.98	1.04	1.18	1.32	1.20	0.91	hypothetical protein low similarity to N-methyl-D-aspartate receptor-associated protein [Drosophila melanogaster] GI:567104, NMDA receptor glutamate-binding subunit [Rattus sp.] GI:8248741; contains Pfam profile PF01027: Uncharacterized protein family UPF0005
AT2G03820	1.02E-02	0.90	0.86	1.19	1.13	1.32	1.31	0.99	nonsense-mediated mRNA decay NMD3 family protein contains Pfam profile: PF04981 NMD3 family
AT4G29950	4.40E-02	0.87	0.76	1.14	1.12	1.32	1.46	1.11	microtubule-associated protein identical to microtubule-associated protein GI:5032258 from [Arabidopsis thaliana]; similar to TBC1 domain family member 5 (Swiss-Prot:Q92609) [Homo sapiens]; contains Pfam profile PF00566: TBC domain
AT4G12240	9.19E-03	0.93	0.86	1.23	1.10	1.32	1.28	0.97	zinc finger (C2H2 type) family protein contains Pfam domain, PF00096: Zinc finger, C2H2 type
AT5G13500	9.32E-03	0.90	0.88	1.19	1.13	1.32	1.28	0.97	expressed protein predicted protein At2g25260 - Arabidopsis thaliana, EMBL:AC007070
AT4G25110	4.85E-03	0.73	1.09	0.97	1.60	1.32	1.47	1.11	latex-abundant family protein (AMC2) / caspase family protein contains similarity to latex-abundant protein [Hevea brasiliensis] gb:AAD13216; contains Pfam profile PF00656: ICE-like protease (caspase) p20 domain
AT4G37980	2.46E-02	0.95	0.88	1.25	1.19	1.32	1.35	1.03	mannitol dehydrogenase, putative (ELI3-1) identical to GI:16267
AT2G22780	6.71E-04	0.87	0.80	1.14	1.08	1.32	1.36	1.03	malate dehydrogenase, glyoxysomal, putative strong similarity to glyoxysomal malate dehydrogenase (EC 1.1.1.37) SP|P19446 [Citrullus lanatus], SP|P46488 [Cucumis sativus], [Medicago sativa] GI:2827078, SP|Q42972 [Oryza sativa], SP|Q9ZP05 [Arabidopsis thaliana], SP|P37228 [Glycine max]; contains InterPro entry IPR001236: Lactate/malate dehydrogenase
AT4G36000	3.51E-02	0.96	0.95	1.26	1.14	1.32	1.20	0.91	pathogenesis-related thaumatin family protein similar to thaumatin-like protein precursor [Pyrus pyrifolia] GI:3241854; contains Pfam profile PF00314: Thaumatin family
AT5G54350	1.24E-02	0.93	0.86	1.23	1.18	1.32	1.38	1.04	expressed protein ; expression supported by MPSS
AT2G01450	2.21E-03	0.91	0.76	1.20	1.19	1.32	1.57	1.19	mitogen-activated protein kinase, putative / MAPK, putative (MPK17) mitogen-activated protein kinase (MAPK)(AtMPK17), PMID:12119167
AT4G34540	3.80E-03	0.87	0.94	1.15	1.23	1.32	1.30	0.99	isoflavone reductase family protein similar to phenylcoumaran benzylic ether reductase homolog Fi1 [Forsythia x intermedia][GI:7578895]; contains isoflavone reductase domain PF02716
AT3G27170	1.43E-02	0.85	0.91	1.12	1.68	1.32	1.84	1.40	chloride channel protein (CLC-b) identical to CLC-b chloride channel protein GB:CAA96058 from [Arabidopsis thaliana] (J. Biol. Chem. 271 (52), 33632-33638 (1996))
AT2G16005	2.81E-02	0.92	0.91	1.21	1.05	1.32	1.16	0.87	MD-2-related lipid recognition domain-containing protein / ML domain-containing protein contains Pfam profile PF02221: ML domain
AT3G53960	2.68E-02	0.80	0.98	1.05	1.61	1.31	1.64	1.25	proton-dependent oligopeptide transport (POT) family protein contains Pfam profile: PF00854 POT family
AT3G48850	3.30E-02	0.72	1.23	0.95	2.08	1.31	1.69	1.29	mitochondrial phosphate transporter, putative similar to mitochondrial phosphate transporter GI:3318617 from [Arabidopsis thaliana]
AT2G44520	5.02E-03	0.71	1.22	0.94	1.70	1.31	1.40	1.06	UbiA prenyltransferase family protein similar to SP|Q12887 Protoheme IX farnesyltransferase, mitochondrial precursor (EC 2.5.1.-) (Heme O synthase) {Homo sapiens}, SP|P21592 COX10 {Saccharomyces cerevisiae}
AT2G23910	2.31E-02	1.15	0.73	1.51	0.94	1.31	1.29	0.99	cinnamoyl-CoA reductase-related similar to cinnamoyl-CoA reductase from Pinus taeda [GI:17978649], Saccharum officinarum [GI:3341511]
AT4G21323	1.63E-02	0.90	0.96	1.19	1.13	1.31	1.17	0.89	subtilase family protein contains similarity to subtilase; SP1 GI:9957714 from [Oryza sativa]
AT1G77210	1.31E-02	0.83	0.87	1.10	1.14	1.31	1.30	0.99	sugar transporter, putative similar to monosaccharide transporter PaMst-1 [Picea abies] GI:2258137, sugar carrier protein GI:169735 from [Ricinus communis], glucose transporter [Saccharum hybrid cultivar H65-7052] GI:347855; contains Pfam profile PF00083: major facilitator superfamily protein
AT3G52150	5.37E-03	0.96	0.94	1.25	1.08	1.31	1.14	0.87	RNA recognition motif (RRM)-containing protein various predicted proteins, Arabidopsis thaliana and others
AT1G67300	5.58E-03	0.89	0.90	1.17	1.21	1.31	1.35	1.03	hexose transporter, putative similar to hexose transporters from Solanum tuberosum [GI:8347244], Nicotiana tabacum [GI:8347246], Arabidopsis thaliana [GI:8347250]; contains Pfam profile PF00083: major facilitator superfamily protein
AT5G53880	4.33E-03	0.96	0.92	1.26	1.03	1.31	1.12	0.85	aldo/keto reductase family protein similar to chalcone reductase [Sesbania rostrata][GI:2792155], and aldose reductase ALDRXV4 [Xerophyta viscosa][GI:4539944], [Hordeum vulgare][GI:728592]
AT3G14960	2.80E-02	0.87	0.86	1.15	1.05	1.31	1.22	0.93	galactosyltransferase family protein contains Pfam profile: PF01762 galactosyltransferase
AT2G44200	1.76E-02	0.64	1.11	0.84	1.43	1.31	1.28	0.98	expressed protein
AT5G16630	2.36E-02	0.94	0.87	1.24	1.30	1.31	1.49	1.14	DNA repair protein Rad4 family low similarity to SP|Q01831 DNA-repair protein complementing XP-C cells (Xeroderma pigmentosum group C complementing protein) [Homo sapiens]; contains Pfam profile PF03835: DNA repair protein Rad4
AT2G13610	1.14E-02	0.98	0.76	1.28	1.03	1.31	1.37	1.04	ABC transporter family protein
AT1G69250	9.72E-03	0.99	0.78	1.29	1.08	1.31	1.37	1.05	nuclear transport factor 2 (NTF2) family protein / RNA recognition motif (RRM)-containing protein contains Pfam profiles PF02136: Nuclear transport factor 2 (NTF2) domain, PF00076: RNA recognition motif (a.k.a. RRM, RBD, or RNP domain)
AT4G26930	1.34E-02	0.92	0.96	1.21	1.05	1.31	1.10	0.84	myb family transcription factor (MYB97) contains Pfam profile: PF00249 myb-like DNA-binding domain ;similar to anther-specific myb-related protein 2 GI:11066263 from [Nicotiana tabacum]
AT5G17530	4.01E-02	0.85	0.88	1.11	1.10	1.31	1.24	0.95	thiF family protein similar to SP|P30138 Adenylyltransferase thiF (EC 2.7.7.-) (Escherichia coli); contains Pfam profile PF00899: ThiF family
AT2G01650	5.02E-03	0.86	0.90	1.12	1.21	1.30	1.34	1.03	zinc finger (C2H2 type) family protein contains Pfam domain, PF00096: Zinc finger, C2H2 type
AT3G07890	1.95E-03	0.94	0.86	1.23	1.24	1.30	1.44	1.10	RabGAP/TBC domain-containing protein similar to plant adhesion molecule 1 [Arabidopsis thaliana] GI:3511223; contains Pfam profile PF00566: TBC domain
AT5G26120	5.76E-03	0.91	0.82	1.17	1.08	1.31	1.33	1.01	glycosyl hydrolase family protein 51 similar to arabinoxylan arabinofuranohydrolase isoenzyme AXAH-II from [Hordeum vulgare] GI:13398414
AT5G62740	2.80E-02	0.84	0.90	1.10	1.07	1.31	1.19	0.91	band 7 family protein strong similarity to hypersensitive-induced response protein [Zea mays] GI:7716470; contains Pfam profile PF01145: SPFH domain / Band 7 family; supporting cDNA gi|17065547|gb|AY062850.1|
AT4G15620	3.70E-02	0.87	0.89	1.14	1.14	1.31	1.27	0.97	integral membrane family protein contains TIGRFAM TIGR01569 : plant integral membrane protein TIGR01569; contains Pfam PF04535 : Domain of unknown function (DUF588), contains plant integral membrane protein, TIGR01569
AT5G46940	1.99E-02	0.92	0.93	1.20	1.05	1.30	1.12	0.86	invertase/pectin methylesterase inhibitor family protein low similarity to SP|P83326 Pectinesterase inhibitor (Pectin methylesterase inhibitor) (PMEI) {Actinidia chinensis}; contains Pfam profile PF04043: Plant invertase/pectin methylesterase inhibitor
AT5G13420	6.23E-03	0.81	0.91	1.06	1.27	1.31	1.40	1.07	transaldolase, putative similar to transaldolase [Solanum tuberosum] gi|2078350|gb|AAB54016
AT3G26030	4.78E-02	0.88	0.86	1.16	1.21	1.31	1.40	1.07	serine/threonine protein phosphatase 2A (PP2A) regulatory subunit B', putative similar to SWISS-PROT:Q28653 serine/threonine protein phosphatase 2A, 56 kDa regulatory subunit, delta isoform (PP2A, B subunit, B' delta isoform, PP2A, B subunit, B56 delta isoform, PP2A, B subunit, PR61 delta isoform, PP2A, B subunit, R5 delta isoform, PP2A, B subunit, B'-gamma) [Oryctolagus cuniculus]; contains Pfam domain, PF01603: Protein phosphatase 2A regulatory B subunit (B56 family)
AT3G24560	2.21E-02	0.92	0.99	1.20	1.06	1.31	1.06	0.81	F-box family protein / SKP1 interacting partner 3-related similar to SKP1 interacting partner 3 GI:10716951 from [Arabidopsis thaliana]
AT5G55070	2.82E-02	0.86	0.89	1.13	1.17	1.31	1.32	1.01	2-oxoacid dehydrogenase family protein similar to SP|Q01205 Dihydrolipoamide succinyltransferase component of 2-oxoglutarate dehydrogenase complex, mitochondrial precursor (EC 2.3.1.61) {Rattus norvegicus}; contains Pfam profiles PF00198: 2-oxo acid dehydrogenases acyltransferase (catalytic domain), PF00364: Biotin-requiring enzyme
AT4G18360	1.56E-02	0.77	0.98	1.00	1.32	1.31	1.34	1.03	(S)-2-hydroxy-acid oxidase, peroxisomal, putative / glycolate oxidase, putative / short chain alpha-hydroxy acid oxidase, putative similar to (S)-2-hydroxy-acid oxidase, peroxisomal (Glycolate oxidase, GOX) (Short chain alpha-hydroxy acid oxidase) [Spinacia oleracea] SWISS-PROT:P05414
AT4G35580	6.12E-03	0.94	0.94	1.23	1.13	1.31	1.19	0.91	no apical meristem (NAM) family protein similar to TIP [Arabidopsis thaliana] GI:9408601; contains Pfam profile PF02365: No apical meristem (NAM) protein
AT3G53150	4.25E-03	0.85	0.93	1.11	1.08	1.31	1.16	0.88	UDP-glucoronosyl/UDP-glucosyl transferase family protein contains Pfam profile: PF00201 UDP-glucoronosyl and UDP-glucosyl transferase
AT5G08090	2.35E-02	0.96	0.88	1.26	1.13	1.31	1.28	0.98	hypothetical protein
AT1G12790	2.26E-02	0.90	0.92	1.17	1.10	1.31	1.20	0.92	expressed protein
AT2G20900	3.28E-02	0.96	0.90	1.25	1.06	1.31	1.18	0.90	expressed protein
AT4G27640	4.48E-02	0.79	0.93	1.03	1.64	1.31	1.77	1.36	importin beta-2 subunit family protein low similarity to importin 4 GI:18700635 from [Homo sapiens]
AT3G17820	2.21E-02	0.97	0.92	1.27	1.05	1.30	1.15	0.88	glutamine synthetase (GS1) identical to glutamine synthetase, cytosolic isozyme (glutamate-- ammonia ligase, GS1) [Arabidopsis thaliana] SWISS-PROT:Q8LVI8
AT1G21710	3.29E-02	1.01	0.88	1.31	1.13	1.30	1.28	0.98	8-oxoguanine-DNA glycosylase (OGG1) similar to SP|O15527 N-glycosylase/DNA lyase [Includes: 8-oxoguanine DNA glycosylase (EC 3.2.2.-); DNA-(apurinic or apyrimidinic site) lyase (EC 4.2.99.18)] {Homo sapiens}; contains Pfam profiles PF00633: Helix-hairpin-helix motif and PF00730: HhH-GPD superfamily base excision DNA repair protein; identical to cDNA 8-oxoguanine-DNA glycosylase (OGG1 gene) GI:16074116
AT1G76380	4.40E-03	0.92	0.88	1.20	1.21	1.30	1.38	1.06	DNA-binding bromodomain-containing protein contains bromodomain, INTERPRO:IPR001487
AT3G11330	1.38E-02	0.95	0.87	1.24	1.21	1.30	1.40	1.07	leucine-rich repeat family protein
AT4G21300	1.39E-03	0.93	0.95	1.22	1.10	1.30	1.16	0.89	pentatricopeptide (PPR) repeat-containing protein contains INTERPRO:IPR002885 PPR repeats
AT5G10010	3.62E-02	0.93	0.86	1.21	1.08	1.30	1.25	0.96	expressed protein
AT4G04670	9.72E-03	0.85	0.89	1.10	1.30	1.30	1.45	1.11	WRKY family transcription factor similar to elicitor response element binding protein WRKY3 isolog GB:AAB63078 [Arabidopsis thaliana]
AT1G09100	1.44E-02	0.90	0.95	1.17	1.02	1.30	1.08	0.83	26S protease regulatory subunit 6A, putative identical to SP:O04019 from [Arabidopsis thaliana]
AT3G62040	2.44E-02	1.00	0.84	1.30	1.02	1.30	1.21	0.93	haloacid dehalogenase-like hydrolase family protein similarity to SP|P53078 SSM1 protein {Saccharomyces cerevisiae}; contains InterPro accession IPR005834: Haloacid dehalogenase-like hydrolase
AT5G50900	3.14E-02	0.91	0.85	1.19	1.12	1.30	1.33	1.02	armadillo/beta-catenin repeat family protein armadillo/beta-catenin-like repeats, Pfam:PF00514
AT3G26690	2.48E-02	0.90	0.79	1.17	1.09	1.30	1.39	1.07	hydrolase, alpha/beta fold family protein contains Pfam profile PF00561: hydrolase, alpha/beta fold family
AT1G68380	3.24E-02	0.80	0.91	1.04	1.18	1.30	1.29	0.99	expressed protein predicated by genscan+
AT1G05040	4.01E-02	0.93	0.95	1.20	1.06	1.30	1.12	0.86	hypothetical protein
AT5G57580	2.48E-02	0.83	0.93	1.08	1.21	1.30	1.31	1.00	calmodulin-binding protein similar to calmodulin-binding protein TCB60 GI:1698548 from [Nicotiana tabacum]
AT4G21160	2.89E-02	0.99	0.82	1.29	1.05	1.30	1.28	0.98	zinc finger and C2 domain protein (ZAC) identical to zinc finger and C2 domain protein GI:9957238 from [Arabidopsis thaliana]
AT2G15080	2.60E-02	0.90	0.83	1.17	1.32	1.30	1.59	1.22	disease resistance family protein contains leucine rich-repeat (LRR) domains Pfam:PF00560, INTERPRO:IPR001611; similar to Cf-2.2 [Lycopersicon pimpinellifolium] gi|1184077|gb|AAC15780
AT1G79690;AT1G	2.74E-02	0.89	0.82	1.15	1.07	1.30	1.30	1.00	[AT1G79690, MutT/nudix family protein contains Pfam NUDIX domain [PF00293]; very low similarity to Chain A and Chain B of Escherichia coli isopentenyl diphosphate:dimethylallyl diphosphate isomerase [gi:15826361] [gi:15826360]];[AT1G79700, ovule development protein, putative similar to ovule development protein AINTEGUMENTA (GI:1209099) [Arabidopsis thaliana]]
AT2G24500	9.99E-03	1.01	0.85	1.31	1.01	1.30	1.19	0.91	zinc finger (C2H2 type) family protein contains Pfam profile: PF00096 zinc finger, C2H2 type
AT3G12270	2.28E-02	0.92	0.90	1.20	1.36	1.30	1.52	1.17	protein arginine N-methyltransferase family protein similar to protein arginine N-methyltransferase 3 from {Rattus norvegicus} SP|O70467, {Homo sapiens} SP|O60678
AT1G28960	3.62E-02	0.98	0.90	1.27	1.06	1.30	1.18	0.91	MutT/nudix family protein similar to coenzyme A diphosphatase [Mus musculus] GI:12746410; contains Pfam profile PF00293: NUDIX domain
AT2G44180	2.37E-02	0.64	1.15	0.84	1.25	1.30	1.09	0.84	methionyl aminopeptidase, putative / methionine aminopeptidase, putative / peptidase M, putative similar to SP|P50579 Methionine aminopeptidase 2 (EC 3.4.11.18) (MetAP 2) {Homo sapiens}; contains Pfam profile PF00557: metallopeptidase family M24
AT5G45690	4.54E-02	0.90	0.97	1.17	1.18	1.30	1.21	0.93	expressed protein
AT4G31790	3.49E-02	0.88	0.91	1.14	1.09	1.30	1.19	0.92	diphthine synthase, putative (DPH5) similar to Diphthine synthase (Diphtamide biosynthesis methyltransferase) (DPH5) (SP:P32469) [Saccharomyces cerevisiae]; similar to CGI-30 protein (GI:4680699) [Homo sapiens]; similar to methyltransferase DPH5, Saccharomyces cerevisiae, PIR:S30890; contains Pfam PF00590 : Tetrapyrrole (Corrin/Porphyrin) Methylases domain; contains TIGRFAMS TIGR00522 : diphthine synthase
AT4G08730	3.55E-02	0.92	0.97	1.19	1.11	1.30	1.15	0.89	hypothetical protein
AT5G09300	1.77E-03	0.85	0.85	1.10	1.25	1.30	1.46	1.12	2-oxoisovalerate dehydrogenase, putative / 3-methyl-2-oxobutanoate dehydrogenase, putative / branched-chain alpha-keto acid dehydrogenase E1 alpha subunit, putative similar to branched-chain alpha-keto acid dehydrogenase E1-alpha subunit [Gallus gallus] GI:12964598; contains Pfam profile PF00676: Dehydrogenase E1 component
AT1G11570	1.44E-02	0.92	0.99	1.20	1.08	1.30	1.09	0.84	nuclear transport factor 2 (NTF2), putative similar to Swiss-Prot P33331 nuclear transport factor 2 (NTF-2) (Nuclear transport factor P10) [Saccharomyces cerevisiae]
AT5G53050	1.69E-02	0.98	0.83	1.27	1.08	1.30	1.30	1.00	hydrolase, alpha/beta fold family protein contains Pfam profile PF00561: hydrolase, alpha/beta fold family
AT4G36790	1.43E-02	0.90	0.92	1.16	1.11	1.30	1.21	0.93	transporter-related low similarity to spinster membrane proteins from [Drosophila melanogaster] GI:12003976, GI:12003972, GI:12003974, GI:12003970; contains Pfam profile PF00083: major facilitator superfamily protein
AT5G14450	2.14E-02	0.86	0.90	1.12	1.13	1.30	1.25	0.96	GDSL-motif lipase/hydrolase family protein similar to early nodulin ENOD8 [Medicago sativa] GI:304037, elicitor-induced glycoprotein iEP4 [Daucus carota] GI:1911765, pollen-expressed cell protein [Medicago sativa] GI:1110502; contains InterPro Entry IPR001087 Lipolytic enzyme, G-D-S-L family
AT4G24040	3.61E-02	0.97	0.86	1.26	1.27	1.30	1.48	1.14	glycosyl hydrolase family 37 / trehalase, putative similar to trehalase 1 GMTRE1 GI:4559292 from [Glycine max]
AT3G19930	1.43E-02	0.72	1.01	0.93	1.48	1.30	1.46	1.13	sugar transport protein (STP4) identical to GB:S25009 GI:16524 from [Arabidopsis thaliana]
AT1G73660	3.58E-02	0.90	0.94	1.17	1.25	1.30	1.34	1.03	protein kinase family protein contains Pfam profile: PF00069 eukaryotic protein kinase domain
AT5G11000	2.70E-02	0.85	0.97	1.10	1.28	1.30	1.31	1.01	expressed protein
AT1G22340	6.47E-03	0.83	0.81	1.08	1.04	1.30	1.28	0.99	UDP-glucoronosyl/UDP-glucosyl transferase family protein contains Pfam profile: PF00201 UDP-glucoronosyl and UDP-glucosyl transferase; similar to UDP-glucose glucosyltransferase GI:3928543 from [Arabidopsis thaliana]
AT5G15480	8.39E-03	0.85	0.91	1.10	1.18	1.30	1.29	1.00	DNA-binding protein, putative similar to DNA-binding protein [Triticum aestivum] GI:6958202; contains Pfam profile PF01535: PPR repeat
AT2G14060	4.67E-02	0.96	0.90	1.25	1.03	1.30	1.14	0.88	S-adenosyl-L-methionine:carboxyl methyltransferase family protein similar to SAM:salicylic acid carboxyl methyltransferase (SAMT) [GI:6002712][Clarkia breweri] and to SAM:benzoic acid carboxyl methyltransferase (BAMT)[GI:9789277][Antirrhinum majus]
AT5G65600	4.64E-02	0.72	1.17	0.94	1.98	1.30	1.70	1.31	legume lectin family protein / protein kinase family protein contains Pfam domains PF00138: Legume lectins alpha domain, PF00139: Legume lectins beta domain and PF00069: Protein kinase domain

AT1G08860	4.23E-02	0.89	0.94	1.15	1.18	1.30	1.25	0.96	copine, putative Similar to BONZAI1 [Arabidopsis thaliana] GI:15487382; contains Pfam profile PF00168: C2 domain
AT1G17500	3.03E-02	0.84	0.94	1.09	1.21	1.30	1.29	0.99	haloacid dehalogenase-like hydrolase family protein similar to Potential phospholipid-transporting ATPase (EC 3.6.3.1) from Homo sapiens [SP|O43520], Mus musculus [SP|P70704]; contains InterPro accession IPR005834: Haloacid dehalogenase-like hydrolase
AT3G52140	4.89E-03	0.94	0.89	1.22	1.55	1.30	1.76	1.35	tetratricopeptide repeat (TPR)-containing protein contains Pfam profile PF00515: TPR Domain
AT4G35830	2.92E-02	0.93	0.84	1.20	1.29	1.29	1.53	1.19	aconitate hydratase, cytoplasmic / citrate hydro-lyase / aconitase (ACO) identical to SP|Q42560 Aconitate hydratase, cytoplasmic (EC 4.2.1.3) (Citrate hydro-lyase) (Aconitase) (Arabidopsis thaliana); contains Pfam profiles PF00330: Aconitase family (aconitate hydratase), PF00[illegible]
AT5G12170	2.31E-02	0.83	0.89	1.08	1.32	1.29	1.49	1.15	expressed protein similar to unknown protein (pir||T09909)
AT2G46270	3.83E-02	0.73	1.18	0.94	1.53	1.29	1.30	1.00	G-box binding factor 3 (GBF3) identical to G-box binding factor 3 (GBF3) SP:P42776 from [Arabidopsis thaliana]; contains Pfam profile: PF00170 bZIP transcription factor
AT3G11320	2.10E-02	0.79	0.91	1.03	1.17	1.29	1.30	1.00	phosphate translocator-related low similarity to phosphoenolpyruvate/phosphate translocator precursor [Mesembryanthemum crystallinum] GI:9295275, phosphate translocator [Nicotiana tabacum] GI:403023; contains Pfam profile: PF00892 integral membrane protein DU[illegible]
AT1G74390	6.48E-03	0.88	0.83	1.14	1.32	1.29	1.58	1.22	exonuclease family protein contains exonuclease domain, Pfam:PF00929
AT5G15490	4.00E-02	0.98	0.79	1.27	1.14	1.29	1.44	1.11	UDP-glucose 6-dehydrogenase, putative very strong similarity to SP|Q96558 UDP-glucose 6-dehydrogenase (EC 1.1.1.22) (UDP-Glc dehydrogenase) (UDP-GlcDH) (UDPGDH) (Glycine max); contains Pfam profiles PF03721: UDP-glucose/GDP-mannose dehydrogenase family NAD binding domain, PF00984: UDP-glucose/GDP-mannose dehydrogenase family central domain, PF03720: UDP-glucose/GDP-mannose dehydrogenase family UDP bindi[illegible]
AT1G12360	4.27E-02	0.94	0.84	1.21	1.26	1.29	1.51	1.17	cytokinesis-related Sec1 protein (KEULE) similar to cytokinesis-related Sec1 protein KEULE [Arabidopsis thaliana] gi|12659318|gb|AAK01291; contains Pfam domain, PF00995: Sec1 family
AT5G03730	2.18E-02	0.87	0.86	1.12	1.79	1.29	2.07	1.60	serine/threonine protein kinase (CTR1) identical to serine/threonine-protein kinase CTR1 [Arabidopsis thaliana] SWISS-PROT:Q05609
AT1G16650	2.93E-03	0.85	0.86	1.10	1.25	1.29	1.45	1.12	expressed protein
AT4G13330	1.68E-03	0.87	0.90	1.13	1.21	1.29	1.33	1.03	expressed protein
AT2G29150	2.78E-02	0.91	0.92	1.17	1.09	1.29	1.18	0.92	tropinone reductase, putative / tropine dehydrogenase, putative similar to tropinone reductase SP:P50165 from [Datura stramonium]
AT4G38150	2.06E-02	0.99	0.85	1.27	1.09	1.29	1.28	0.99	pentatricopeptide (PPR) repeat-containing protein contains Pfam profile PF01535: PPR repeat
AT3G59050	4.24E-02	0.85	0.88	1.10	1.16	1.29	1.32	1.02	amine oxidase family protein similar to polyamine oxidase (EC 1.5.3.11) precursor - Zea mays [SP|O64411]; contains Pfam profile PF01593 amine oxidase, flavin-containing
AT2G31060	1.74E-02	0.92	0.82	1.19	1.27	1.29	1.54	1.20	elongation factor family protein contains Pfam profiles: PF00009 elongation factor Tu GTP binding domain, PF00679 elongation factor G C-terminus, PF03144 elongation factor Tu domain 2
AT3G02280	5.49E-03	0.89	0.92	1.15	1.26	1.29	1.37	1.06	flavodoxin family protein low similarity to SP|Q05001 NADPH-cytochrome P450 reductase (EC 1.6.2.4) (Catharanthus roseus), similar to NADPH-dependent FMN and FAD containing oxidoreductase [Homo sapiens] GI:6694369; contains Pfam profiles PF00258: flavodoxin, PF00667: FAD binding domain, PF00175: Oxidoreductase NAD-binding domain
AT5G15640	3.89E-02	0.90	0.90	1.17	1.07	1.29	1.19	0.92	mitochondrial substrate carrier family protein contains Pfam profile: PF00153 mitochondrial carrier protein
ATMG00770	3.29E-02	0.87	0.91	1.13	1.01	1.29	1.11	0.86	orf100b hypothetical protein
AT5G23830	1.69E-02	0.94	0.90	1.22	1.06	1.29	1.17	0.91	MD-2-related lipid recognition domain-containing protein / ML domain-containing protein contains Pfam profile PF02221: ML domain
AT3G54620	1.25E-02	0.86	0.84	1.11	1.20	1.29	1.42	1.10	bZIP transcription factor family protein contains Pfam profile: PF00170 bZIP transcription factor
AT1G74210	2.46E-02	0.93	0.93	1.20	1.15	1.29	1.23	0.95	glycerophosphoryl diester phosphodiesterase family protein low similarity to glycerophosphodiester phosphodiesterase [Borrelia hermsii] GI:1399038; contains Pfam profile PF03009: Glycerophosphoryl diester phosphodiesterase family
AT3G57160	4.27E-02	0.94	0.93	1.21	1.05	1.29	1.13	0.87	expressed protein
AT3G60320	1.79E-02	0.91	0.78	1.18	1.33	1.29	1.70	1.32	expressed protein contains Pfam profiles: PF04782: protein of unknown function (DUF632), PF04783: protein of unknown function (DUF630)
AT3G16330	3.15E-02	0.89	0.88	1.15	1.03	1.29	1.17	0.90	expressed protein
AT1G65930	2.58E-03	0.88	0.96	1.14	1.14	1.29	1.18	0.92	isocitrate dehydrogenase, putative / NADP+ isocitrate dehydrogenase, putative strong similarity to isocitrate dehydrogenase SP|Q40345 from [Medicago sativa]
AT5G46750	3.55E-03	0.94	0.86	1.22	1.08	1.29	1.26	0.98	human Rev interacting-like family protein / hRIP family protein contains Pfam profile PF01412: Putative GTP-ase activating protein for Arf
AT3G08950	2.36E-03	0.90	0.92	1.16	1.06	1.29	1.14	0.89	electron transport SCO1/SenC family protein similar to SP|P23833 SCO1 protein, mitochondrial precursor (Saccharomyces cerevisiae); contains Pfam profile PF02630: SCO1/SenC
AT5G04120	4.10E-02	0.90	0.95	1.15	1.09	1.29	1.15	0.89	phosphoglycerate/bisphosphoglycerate mutase family protein low similarity to SP|P36623 Phosphoglycerate mutase (EC 5.4.2.1) (Schizosaccharomyces pombe); contains Pfam profile PF00300: phosphoglycerate mutase family
AT3G46340	1.10E-02	0.93	0.89	1.20	1.13	1.29	1.28	0.99	leucine-rich repeat protein kinase, putative similar to light repressible receptor protein kinase [Arabidopsis thaliana] gi|1321686|emb|CAA66376; contains leucine rich repeat (LRR) domains, Pfam:PF00560; contains protein kinase domain, Pfam:PF00069
AT5G07280	2.58E-02	0.81	0.91	1.05	1.35	1.29	1.48	1.15	leucine-rich repeat protein kinase, putative / extra sporogenous cells (ESP) identical to extra sporogenous cells [Arabidopsis thaliana] gi|23304947|emb|CAD42912; contains Pfam domains PF00560: Leucine Rich Repeat and PF00069: Protein kinase domain
AT1G07750	2.80E-02	0.89	0.93	1.15	1.16	1.29	1.26	0.98	cupin family protein similar to legumin (11S-globulin) from Ginkgo biloba [GI:949869], 11S globulin from Avena sativa [GI:472867]
AT5G65000	1.58E-02	0.87	0.84	1.12	1.11	1.29	1.33	1.03	nucleotide-sugar transporter family protein low similarity to SP|Q9Y2D2 UDP N-acetylglucosamine transporter (Homo sapiens); contains Pfam profile PF04142: Nucleotide-sugar transporter
AT3G55130	1.87E-02	0.87	0.94	1.12	1.71	1.29	1.82	1.41	ABC transporter family protein breast cancer resistance protein 1 BCRP1, Mus musculus, EMBL:NP_036050
AT2G05710	3.67E-02	0.93	0.90	1.20	1.48	1.29	1.65	1.28	aconitate hydratase, cytoplasmic, putative / citrate hydro-lyase/aconitase, putative nearly identical to SP|P49608 Aconitate hydratase, cytoplasmic (EC 4.2.1.3) (Citrate hydro-lyase) (Aconitase) (Cucurbita maxima); contains Pfam profiles PF00330: Aconitase family (aconitate hydratase), PF00694: Aconitase C-terminal domain
AT2G20010	2.68E-02	0.89	0.94	1.15	1.50	1.29	1.60	1.25	expressed protein
AT4G13430	1.48E-02	0.91	0.84	1.17	1.18	1.29	1.40	1.09	aconitase family protein / aconitate hydratase family protein contains Pfam profile PF00330: Aconitase family (aconitate hydratase
AT4G16850	1.86E-02	0.91	0.94	1.18	1.02	1.29	1.09	0.84	expressed protein
AT1G78980	2.21E-02	0.95	0.97	1.22	1.04	1.29	1.08	0.84	leucine-rich repeat transmembrane protein kinase, putative similar to leucine-rich repeat transmembrane protein kinase 2 GI:3360291 from [Zea mays]
AT3G15260	6.32E-03	0.93	0.86	1.19	1.13	1.29	1.32	1.02	protein phosphatase 2C, putative / PP2C, putative similar to protein phosphatase type 2C GB:AAD17805 from [Lotus japonicus]
AT5G60590	3.89E-02	0.92	0.94	1.18	1.05	1.29	1.12	0.87	yrdC protein-related
AT5G59940	2.60E-02	0.91	0.95	1.17	1.14	1.29	1.20	0.93	DC1 domain-containing protein / UV-B light-insensitive protein, putative similar to ULI3 (UV-B light insensitive) [Arabidopsis thaliana] GI:17225050; contains Pfam profile PF03107: DC1 domain
AT3G54240	3.03E-02	0.88	0.90	1.14	1.16	1.29	1.29	1.00	hydrolase, alpha/beta fold family protein low similarity to SP|P22862 |ESTE_PSEFL Arylesterase (EC 3.1.1.2) (Aryl-ester hydrolase) (Pseudomonas fluorescens); contains Pfam profile PF00561: hydrolase, alpha/beta fold family
AT1G76150	1.67E-03	0.96	0.91	1.24	1.15	1.29	1.26	0.98	maoC-like dehydratase domain-containing protein contains similarity to Swiss-Prot:P51659 estradiol 17 beta-dehydrogenase 4 (17-beta-HSD 4) (17-beta-hydroxysteroid dehydrogenase 4) [Homo sapiens]; contains Pfam profile PF01575: MaoC like domain
AT3G45870	1.10E-02	0.91	0.90	1.17	1.07	1.29	1.18	0.92	integral membrane family protein / nodulin MtN21-related similar to MtN21 GI:2598575 (root nodule development) Medicago truncatula, EMBL:MTY15293
AT1G47420	3.74E-02	0.95	0.94	1.22	1.01	1.29	1.07	0.83	expressed protein identical to hypothetical protein GB:AAD46040 GI:5668814 from [Arabidopsis thaliana]
AT1G20410	1.22E-03	0.91	0.80	1.18	1.16	1.29	1.45	1.12	expressed protein
AT4G17230	4.15E-02	0.81	0.92	1.04	1.64	1.29	1.78	1.39	scarecrow-like transcription factor 13 (SCL13)
[illegible]									

AT3G58190	2.18E-02	1.01	0.89	1.24	1.10	1.23	1.23	1.00	LOB domain protein 29 / lateral organ boundaries domain protein 29 (LBD29) identical to SP|Q9M2J7 LOB domain protein 29 [Arabidopsis thaliana]; supported by full-length cDNA gi:17227167
AT1G74910	6.55E-03	0.91	0.82	1.12	1.20	1.23	1.46	1.18	ADP-glucose pyrophosphorylase family protein contains Pfam profile PF00483: Nucleotidyl transferase; low similarity to mannose-1-phosphate guanylyltransferase [Hypocrea jecorina] GI:3323397
AT3G05970	1.25E-02	0.95	0.80	1.17	1.11	1.23	1.39	1.13	long-chain-fatty-acid--CoA ligase / long-chain acyl-CoA synthetase (LACS6) strong similarity to AMP-binding protein (MF39P) gi:1617274 from Brassica napus, similar to putative long-chain-fatty-acid--CoA ligase (brain isozyme) GB:P33124 [Rattus norvegicus]; contains Pfam AMP-binding enzyme domain PF00501; identical to cDNA AtLACS6 for long-chain acyl-CoA synthetase GI:22531705
AT4G15420	2.59E-02	0.96	0.90	1.18	1.12	1.23	1.25	1.01	PRLI-interacting factor K nearly identical to PRLI-interacting factor K [Arabidopsis thaliana] GI:11139266; contains Pfam profiles PF03152: Ubiquitin fusion degradation protein UFD1, PF00096: Zinc finger, C2H2 type
AT1G31770	1.23E-02	0.92	0.85	1.13	1.07	1.23	1.26	1.02	ABC transporter family protein contains Pfam profile: PF00005: ABC transporter
AT1G34180	4.01E-02	0.97	0.83	1.20	0.98	1.23	1.18	0.96	no apical meristem (NAM) family protein contains Pfam PF02365: No apical meristem (NAM) domain; similar to NAM-like protein GI:8809651 from (Arabidopsis thaliana)
AT1G77260	2.20E-02	1.01	0.81	1.24	1.00	1.23	1.23	1.00	dehydration-responsive protein-related similar to early-responsive to dehydration stress ERD3 protein [Arabidopsis thaliana] GI:15320410; contains Pfam profile PF03141: Putative methyltransferase
AT1G12640	2.47E-02	0.92	0.81	1.13	1.16	1.23	1.44	1.17	membrane bound O-acyl transferase (MBOAT) family protein low similarity to porcupine from [Xenopus laevis] GI:6714514, GI:6714520, GI:6714518, GI:6714516; contains Pfam profile PF03062: MBOAT family
AT5G44440	3.81E-02	0.98	0.96	1.20	1.08	1.23	1.12	0.91	FAD-binding domain-containing protein similar to SP|P30986 reticuline oxidase precursor (Berberine-bridge-forming enzyme) (BBE) (Tetrahydroprotoberberine synthase) [Eschscholzia californica]; contains PF01565 FAD binding domain
AT1G06840	3.70E-02	0.81	0.98	1.00	1.55	1.23	1.59	1.29	leucine-rich repeat transmembrane protein kinase, putative similar to receptor protein kinase GB:BAA11869 GI:1389566 from [Arabidopsis thaliana]
AT2G39340	2.22E-02	0.70	1.14	0.86	1.95	1.23	1.70	1.38	SAC3/GANP family protein contains Pfam profile: PF03399 SAC3/GANP family
AT1G51790	2.91E-02	0.88	0.96	1.08	1.50	1.23	1.55	1.26	leucine-rich repeat protein kinase, putative similar to light repressible receptor protein kinase [Arabidopsis thaliana] gi|1321686|emb|CAA66376; contains leucine rich repeat (LRR) domains, Pfam:PF00560; contains protein kinase domain, Pfam:PF00069
AT4G08840	5.34E-03	0.95	0.94	1.17	1.12	1.23	1.19	0.97	pumilio/Puf RNA-binding domain-containing protein contains similarity to RNA binding protein PufA [Dictyostelium discoideum] gi|5106561|gb|AAD39751
AT4G40000	3.26E-02	0.90	0.84	1.11	1.15	1.23	1.37	1.11	NOL1/NOP2/sun family protein contains Pfam profile PF01189: NOL1/NOP2/sun family
AT4G38200	3.80E-02	0.90	0.92	1.11	1.44	1.23	1.55	1.26	guanine nucleotide exchange family protein similar to Brefeldin A-inhibited guanine nucleotide-exchange protein 2 [Homo sapiens] SP|Q9Y6D5; contains Pfam profile PF01369: Sec7 domain
AT5G25220	4.83E-02	0.94	0.94	1.16	1.08	1.23	1.15	0.94	homeobox protein knotted-1 like 3 (KNAT3) identical to homeobox protein knotted-1 like 3 (KNAT3) SP:P48000 from [Arabidopsis thaliana]
AT5G41280	3.91E-02	0.90	0.95	1.10	1.09	1.23	1.15	0.94	hypothetical protein contains Pfam profile: PF01657 domain of unknown function
AT1G77470	3.51E-03	0.95	0.89	1.17	1.07	1.23	1.19	0.97	replication factor C 36 kDA, putative similar to SWISS-PROT:P40937 activator 1 36 kDa subunit (Replication factor C 36 kDa subunit, A1 36 kDa subunit, RF-C 36 kDa subunit, RFC36) [Homo sapiens]
AT3G26480	3.59E-02	0.92	0.97	1.14	1.07	1.23	1.10	0.89	transducin family protein / WD-40 repeat family protein contains Pfam profile: PF00400 WD domain, G-beta repeat (5 copies, 2 below cutoff); related to LACK protective antigen (GI:13625467) [Leishmania donovani]
AT1G21700	2.43E-02	0.96	0.83	1.18	1.16	1.23	1.40	1.14	SWIRM domain-containing protein / DNA-binding family protein contains similarity to SWI/SNF complex 170 KDa subunit [Homo sapiens] gi|1549241|gb|AAC50694; contains Pfam domain PF04433: SWIRM domain, PF00249: Myb-like DNA-binding domain
AT1G77990	1.16E-02	0.80	0.99	0.99	1.29	1.23	1.30	1.06	sulfate transporter identical to sulfate transporter [Arabidopsis thaliana] GI:1498120
AT4G19240	3.81E-02	0.88	0.92	1.08	1.17	1.23	1.27	1.04	expressed protein
AT3G17910	8.26E-03	1.00	0.87	1.23	0.99	1.23	1.14	0.93	surfeit 1 (SURF1) identical to Surfeit 1 GB:AAF19609 from [Arabidopsis thaliana]
AT5G12420	2.81E-02	0.73	1.24	0.90	1.73	1.23	1.40	1.14	expressed protein
AT1G79590	4.55E-02	0.95	0.86	1.16	1.00	1.23	1.16	0.94	syntaxin 52 (SYP52) identical to Swiss-Prot:Q94KK7 syntaxin 52 (AtSYP52) [Arabidopsis thaliana]
AT1G28450;AT1G	2.18E-02	0.93	0.93	1.14	1.08	1.23	1.16	0.94	[AT1G28450, MADS-box family protein similar to MADS-box protein GI:2160701 from [Pinus radiata][;]AT1G28460, MADS-box family protein contains similarity to MADS-box transcription factor GI:6580947 from [Picea abies]]
AT5G10840;AT5G	4.67E-02	0.97	0.81	1.20	1.20	1.23	1.48	1.21	[AT5G10840, endomembrane protein 70, putative TM4 family;];[AT5G10830, embryo-abundant protein-related similar to embryo-abundant protein [Picea glauca] GI:1350531]
AT5G56470	3.69E-02	0.94	0.92	1.15	1.12	1.23	1.21	0.99	FAD-dependent oxidoreductase family protein contains TIGRfam TIGR01677: plant-specific FAD-dependent oxidoreductase
AT2G24740	1.86E-03	0.99	0.94	1.21	1.00	1.23	1.07	0.87	SET domain-containing protein (SUVH8) identical to SUVH8 [Arabidopsis thaliana] GI:13517757; contains Pfam profiles PF00856: SET domain, PF05033: Pre-SET motif, PF02182: YDG/SRA domain
AT5G63570	9.19E-03	0.97	0.96	1.19	1.12	1.23	1.16	0.94	glutamate-1-semialdehyde 2,1-aminomutase 1 (GSA 1) / glutamate-1-semialdehyde aminotransferase 1 (GSA-AT 1) identical to GSA 1 [SP|P42799]
AT4G38380	1.61E-02	0.91	0.88	1.12	1.20	1.23	1.37	1.11	MATE efflux protein-related T19C21.18 Arabidopsis thaliana chromosome II BAC T19C21 genomic sequence, PID:g3395439
AT2G22710	4.64E-02	0.91	0.97	1.12	1.11	1.23	1.15	0.93	myb family protein
AT3G27440	1.80E-02	0.86	1.00	1.06	1.15	1.23	1.14	0.93	uracil phosphoribosyltransferase, putative / UMP pyrophosphorylase, putative / UPRTase, putative similar to SP|O65583 Uracil phosphoribosyltransferase (EC 2.4.2.9) (UMP pyrophosphorylase) (UPRTase) [Arabidopsis thaliana]; contains Pfam profile PF00485: Phosphoribulokinase / Uridine kinase family
AT5G64560	7.33E-04	0.94	0.85	1.16	1.09	1.23	1.28	1.04	magnesium transporter CorA-like family protein (MRS2-2) weak similarity to SP|Q01926 RNA splicing protein MRS2, mitochondrial precursor [Saccharomyces cerevisiae]; contains Pfam profile PF01544: CorA-like Mg2+ transporter protein
AT5G41660	2.93E-02	0.94	0.97	1.15	1.08	1.23	1.11	0.91	hypothetical protein similar to unknown protein (emb|CAB86921.1)
AT5G10610	4.22E-02	1.00	0.92	1.22	1.04	1.23	1.13	0.92	cytochrome P450 family protein similar to Cytochrome P450 91A1 (SP:Q9FG65) [Arabidopsis thaliana[; similar to cytochrome P450, Helianthus tuberosus, EMBL:HTCYP81L
AT3G51150	8.91E-04	0.93	0.78	1.14	1.11	1.23	1.41	1.15	kinesin motor family protein contains Pfam domain, PF00225: Kinesin motor domain
AT2G07360	1.87E-02	0.94	0.91	1.15	1.23	1.23	1.35	1.10	SH3 domain-containing protein contains Pfam profile PF00018: SH3 domain
AT1G74080	7.61E-03	0.87	0.94	1.07	1.07	1.23	1.13	0.92	myb family transcription factor (MYB122) contains Pfam profile: PF00249 myb-like DNA-binding domain
AT5G18090	4.32E-02	0.93	0.91	1.14	1.02	1.23	1.12	0.91	transcriptional factor B3 family protein contains Pfam profile PF02362: B3 DNA binding domain
AT2G41430	4.81E-02	0.85	1.00	1.04	1.05	1.23	1.04	0.85	dehydration-induced protein (ERD15) identical to dehydration-induced protein ERD15 GI:710626 from [Arabidopsis thaliana]
AT2G20410	3.31E-03	0.99	0.90	1.21	1.05	1.23	1.17	0.95	activating signal cointegrator-related similar to ASC-1 (GI:6581062) [Mus musculus]; similar to Activating signal cointegrator 1 (ASC-1) (Thyroid receptor interacting protein 4) (TRIP-4) (Swiss-Prot:Q15650) [Homo sapiens]
AT1G21312	5.46E-04	0.92	0.88	1.12	1.14	1.23	1.30	1.06	RNA recognition motif (RRM)-containing protein contains Pfam profile PF00076: RNA recognition motif. (a.k.a. RRM, RBD, or RNP domain
AT1G42560	3.62E-02	0.82	0.98	1.00	1.14	1.23	1.16	0.95	seven transmembrane MLO family protein / MLO-like protein 9 (MLO9) nearly identical to membrane protein Mlo9 [Arabidopsis thaliana] GI:14091588; similar to MLO protein SWISS-PROT:P93766, NCBI_gi:1877221 [Hordeum vulgare][Barley]
AT1G23210	3.32E-02	0.97	0.93	1.18	1.08	1.23	1.16	0.95	glycosyl hydrolase family 9 protein similar to endo-1,4-beta-glucanase GB:CAA67157 GI:2440035 from (Arabidopsis thaliana)
AT3G47840	1.60E-02	0.98	0.78	1.19	1.05	1.22	1.35	1.10	pentatricopeptide (PPR) repeat-containing protein contains Pfam profile PF01535: PPR repeat
AT1G18140	4.60E-02	0.91	1.01	1.11	1.09	1.22	1.08	0.89	laccase family protein / diphenol oxidase family protein similar to high-pI laccase (LAC2-1) GI:1621460 from [Liriodendron tulipifera]
AT2G33240	4.33E-03	0.87	0.93	1.07	1.10	1.22	1.18	0.96	myosin, putative similar to myosin (GI:433663) [Arabidopsis thaliana]; myosin myxA (SP:Q02440) [Gallus gallus]
AT3G08620	1.46E-03	0.89	0.88	1.09	1.12	1.22	1.27	1.03	DEAD box RNA helicase (RH25) identical to RNA helicase [Arabidopsis thaliana] GI:3776023; contains Pfam profiles PF00270: DEAD/DEAH box helicase, PF00271: Helicase conserved C-terminal domain
AT2G14010	2.43E-02	0.99	0.92	1.21	1.05	1.22	1.14	0.93	hypothetical protein and genefinder
AT3G12670	4.12E-02	0.87	0.93	1.07	1.28	1.22	1.37	1.12	CTP synthase, putative / UTP--ammonia ligase, putative similar to SP|P17812 CTP synthase (EC 6.3.4.2) (UTP--ammonia ligase) [Homo sapiens]; contains Pfam profile PF00117: glutamine amidotransferase class-I
AT3G05160;AT3G	4.00E-02	0.94	0.88	1.15	1.12	1.22	1.27	1.04	[AT3G05160, sugar transporter, putative similar to sugar-porter family proteins 1 and 2 [Arabidopsis thaliana] GI:14585699, GI:14585701; contains Pfam profile PF00083: major facilitator superfamily protein[;]AT3G05165, sugar transporter, putative similar to sugar-porter family proteins 1 and 2 [Arabidopsis thaliana] GI:14585699, GI:14585701; contains Pfam profile PF00083: major facilitator superfamily p[illegible]
AT2G46800	2.09E-02	0.78	1.10	0.95	1.40	1.22	1.27	1.04	zinc transporter (ZAT) identical to zinc transporter ZAT [Arabidopsis thaliana] gi|4206640|gb|AAD11757; member of the cation diffusion facilitator (CDF) family, or cation efflux (CE) family, PMID:11500563
AT1G74420	4.30E-02	1.04	0.83	1.27	1.01	1.22	1.22	1.00	xyloglucan fucosyltransferase, putative (FUT3) identical to SP|Q9CA71 Probable fucosyltransferase 3 (EC 2.4.1.-) (AtFUT3) [Arabidopsis thaliana]; similar to SP|Q9SWH5 Galactoside 2-alpha-L-fucosyltransferase (EC 2.4.1.69) (Xyloglucan alpha-(1,2)-fucosyltransferase) (AtFUT1) [Arabidopsis thaliana]
AT2G05290	2.58E-02	0.95	0.92	1.17	1.15	1.22	1.25	1.02	expressed protein similar to zinc finger protein [Arabidopsis thaliana] GI:976277
AT1G54570	3.00E-02	1.09	0.81	1.33	1.09	1.22	1.35	1.10	esterase/lipase/thioesterase family protein contains Interpro entry IPR000379
AT5G53660	1.10E-02	0.93	0.91	1.14	1.16	1.22	1.28	1.05	expressed protein
AT5G17250	3.98E-02	0.84	1.00	1.03	1.13	1.22	1.12	0.92	phosphatidylinositolglycan class O (PIG-O) family protein similar to Pig-o [Mus musculus] GI:8099971
AT4G01100	2.29E-02	0.98	0.84	1.20	1.12	1.22	1.33	1.09	mitochondrial substrate carrier family protein contains Pfam profile: PF00153 mitochondrial carrier protein
AT5G49080	3.66E-02	0.93	0.94	1.14	1.05	1.22	1.12	0.92	proline-rich extensin-like family protein contains proline-rich extensin domains, INTERPRO:IPR002965
AT5G09940	3.01E-02	0.97	0.92	1.18	1.04	1.22	1.14	0.93	hypothetical protein
AT1G47240	1.84E-02	0.88	0.84	1.07	1.20	1.22	1.43	1.17	NRAMP metal ion transporter 2, putative (NRAMP2) similar to metal transporter Nramp3 [Arabidopsis thaliana] gi|6468012|gb|AAF13278; member of the natural resistance-associated macrophage protein (NRAMP) metal transporter family, PMID:11500563
AT1G08070	2.47E-02	0.99	0.89	1.21	1.08	1.22	1.21	0.99	pentatricopeptide (PPR) repeat-containing protein contains Pfam profile PF01535: PPR repeat
AT1G15000	2.30E-02	0.90	0.89	1.10	1.24	1.22	1.39	1.14	serine carboxypeptidase S10 family protein similar to Serine carboxypeptidase precursor (SP:P32826) [Arabidopsis thaliana]; similar to GB:AAD42963 from [Matricaria chamomilla]
AT4G12650	4.27E-02	0.96	0.85	1.17	1.23	1.22	1.45	1.18	endomembrane protein 70, putative TM4 family;
AT1G67420	3.96E-03	0.93	0.89	1.13	1.07	1.22	1.20	0.98	24 kDa vacuolar protein, putative similar to 24 kDa vacuolar protein VP24 [Ipomoea batatas] gi|5821406|dbj|BAA83809
AT3G08960	9.19E-03	0.85	0.97	1.03	1.46	1.22	1.51	1.24	importin beta-2 subunit family protein low similarity to Lph2p [Saccharomyces cerevisiae] GI:1163089; contains Pfam profile PF03810: Importin-beta N-terminal domain
AT3G22450	3.10E-02	0.97	0.97	1.19	1.03	1.22	1.06	0.87	expressed protein ; expression supported by MPSS
ATMG00920;AT2G	4.51E-02	0.93	0.94	1.13	1.02	1.22	1.08	0.89	[ATMG00920, orf215b hypothetical protein];[AT2G07678, hypothetical protein]
AT5G41800	4.12E-02	0.96	0.92	1.18	1.09	1.22	1.19	0.98	amino acid transporter family protein similar to amino acid permease 1 [Nicotiana sylvestris] GI:976402; belongs to INTERPRO:IPR002422 amino acid/polyamine transporter, family II
AT4G38510	1.53E-02	1.01	0.79	1.23	1.14	1.22	1.46	1.19	vacuolar ATP synthase subunit B, putative / V-ATPase B subunit, putative / vacuolar proton pump B subunit, putative / V-ATPase 57 kDa subunit, putative very strong similarity to SP|P11574 Vacuolar ATP synthase subunit B (EC 3.6.3.14) (V-ATPase B subunit) (Vacuolar proton pump B subunit) (V-ATPase 57 kDa subunit) [Arabidopsis thaliana]; contains Pfam profiles PF00006: ATP synthase alpha/beta family n[illegible]
AT5G04310	1.11E-02	0.84	0.89	1.03	1.14	1.22	1.28	1.05	pectate lyase family protein similar to pectate lyase GP:14531296 from [Fragaria x ananassa]
AT4G23010	3.82E-02	0.90	0.90	1.10	1.20	1.22	1.34	1.09	UDP-galactose transporter-related contains weak similarity to UDP-galactose transporter related isozyme 1 (GI:1669562) [Mus musculus]
AT4G24330	4.49E-02	0.96	0.91	1.17	1.17	1.22	1.29	1.06	expressed protein hypothetical protein - Caenorhabditis elegans,PID:e1350884
AT5G64390	3.53E-02	0.92	0.84	1.12	1.28	1.22	1.53	1.25	KH domain-containing RNA-binding protein (HEN4) contains similarity to RNA-binding protein; identical to cDNA HEN4 isoform 2 (HEN4) GI:28261404; contains Pfam domain PF00013: KH domain; identical to cDNA HEN4 (HEN4) GI:28261402
AT5G49110	7.75E-03	0.99	0.90	1.21	1.05	1.22	1.16	0.95	expressed protein ; expression supported by MPSS
AT4G20370	3.79E-02	0.95	0.99	1.16	1.14	1.22	1.15	0.94	hypothetical protein
ATMG00370;AT2G	1.42E-02	0.94	0.91	1.15	1.06	1.22	1.16	0.95	[ATMG00370, orf199 hypothetical protein];[AT2G07739, expressed protein]
AT1G53490	2.38E-02	0.86	1.00	1.05	1.22	1.22	1.22	1.00	bZIP protein
AT4G20770	2.77E-02	0.92	0.98	1.12	1.09	1.22	1.11	0.91	pentatricopeptide (PPR) repeat-containing protein contains Pfam profile PF01535: PPR repeat
AT4G00570	4.95E-02	0.92	0.87	1.12	1.13	1.22	1.30	1.06	malate oxidoreductase, putative similar to NAD-dependent malic enzyme 59 kDa isoform, mitochondrial precursor (EC 1.1.1.39) (NAD-ME) (SP:P37225) [Solanum tuberosum]
AT5G60730	4.40E-02	0.93	0.94	1.13	1.30	1.22	1.39	1.14	anion-transporting ATPase family protein low similarity to SP|O43681 Arsenical pump-driving ATPase (EC 3.6.3.16) (Arsenite-translocating ATPase) (Arsenical resistance ATPase) (Arsenite-transporting ATPase) (ARSA) (ASNA-I) [Homo sapiens]; contains Pfam profile PF02374: Anion-transporting ATPase
AT2G18360	2.90E-02	0.88	0.94	1.07	1.08	1.22	1.14	0.94	hydrolase, alpha/beta fold family protein low similarity to SP|P24640|LIP3_MORSP Lipase 3 precursor (EC 3.1.1.3) (Triacylglycerol lipase) [Moraxella sp]; contains Pfam profile PF00561: hydrolase, alpha/beta fold family
AT3G05960	2.18E-02	0.90	0.91	1.10	1.16	1.22	1.27	1.04	sugar transporter, putative similar to hexose transporter GI:5734440 GB:CAB52689 [Lycopersicon esculentum], Sugar carrier protein C [Ricinus communis] SP|Q41144, monosaccharide transporter [Nicotiana tabacum] GI:19885; contains Pfam profile PF00083: major facilitator superfamily protein
AT3G18540	3.17E-02	0.99	0.92	1.21	1.01	1.22	1.09	0.90	expressed protein
AT2G20700	4.76E-02	0.98	0.91	1.19	1.00	1.22	1.10	0.90	expressed protein
AT1G23970;AT1G	2.15E-02	0.91	0.93	1.11	1.04	1.22	1.12	0.92	[AT1G23970, expressed protein contains Pfam profile PF04776: Protein of unknown function (DUF626)];[AT1G23950, expressed protein]
AT1G72560	3.33E-02	0.89	0.95	1.09	1.27	1.22	1.33	1.09	tRNA export mediator exportin-t, putative (PAUSED) contains Pfam profile: PF04150 exportin-t, identical to PAUSED gi:30909318
ATMG00680	3.95E-02	0.94	0.93	1.15	1.11	1.22	1.19	0.98	orf122c hypothetical protein
AT2G43160	2.30E-02	0.70	1.14	0.86	1.92	1.22	1.68	1.38	epsin N-terminal homology (ENTH) domain-containing protein contains Pfam PF01417: ENTH domain. ENTH (Epsin N-terminal homology) domain; similar to Af10-protein (GI:1724114) [Avena fatua]
AT1G66680	1.18E-02	0.92	0.90	1.12	1.20	1.22	1.33	1.09	S locus-linked protein, putative similar to S locus-linked protein SLL2 [Brassica napus] GI:1518113
AT5G09880	2.00E-02	0.93	0.93	1.13	1.13	1.22	1.21	0.99	RNA recognition motif (RRM)-containing protein
AT3G63050	3.89E-02	0.90	0.94	1.10	1.03	1.22	1.09	0.89	expressed protein
AT3G45840	9.63E-03	0.94	0.91	1.15	1.05	1.22	1.15	0.94	DC1 domain-containing protein contains Pfam profile PF03107: DC1 domain
AT2G30840	1.30E-02	0.89	0.90	1.08	1.10	1.22	1.22	1.00	2-oxoglutarate-dependent dioxygenase, putative similar to 2A6 (GI:599622) and tomato ethylene synthesis regulatory protein E8 (SP|P10967)
AT3G10750	3.73E-02	0.99	0.89	1.21	1.13	1.22	1.26	1.04	hypothetical protein
AT5G64813	9.87E-04	0.96	0.86	1.17	1.14	1.22	1.33	1.09	GTP-binding protein-related contains weak similarity to Ras-related protein Rab-27A (Rab-27) (GTP-binding protein Ram) (Swiss-Prot:P51159) [Homo sapiens]
AT1G30780	2.59E-02	0.93	0.88	1.13	1.04	1.22	1.18	0.97	F-box family protein
AT5G67090	4.29E-02	0.89	0.92	1.08	1.08	1.22	1.17	0.96	subtilase family protein contains similarity to subtilisin-like protease ag12 GI:757522 from [Alnus glutinosa]
AT1G25360	4.20E-02	0.88	0.96	1.07	1.18	1.22	1.23	1.01	pentatricopeptide (PPR) repeat-containing protein contains INTERPRO:IPR002885 PPR repeats
AT5G43930	2.13E-02	0.92	0.88	1.11	1.16	1.22	1.32	1.09	transducin family protein / WD-40 repeat family protein contains 4 WD-40 repeats (PF00400); similar to WD-repeat protein 5 (SP:Q9UGP9) [Homo sapiens]
AT2G04430	1.33E-02	0.90	0.88	1.09	1.12	1.22	1.27	1.04	MutT/nudix family protein similar to SP|P53370 Nucleoside diphosphate-linked moiety X motif 6 [Homo sapiens]; contains Pfam profile PF00293: NUDIX domain
AT5G37620	4.65E-02	0.92	0.98	1.12	1.04	1.22	1.07	0.88	DC1 domain-containing protein contains Pfam profile PF03107: DC1 domain
AT4G35780	4.04E-02	0.98	0.86	1.19	1.04	1.22	1.21	0.99	protein kinase family protein similar to protein kinase [gi:170047] from Glycine max, [gi:13124865] from Arachis hypogaea; contains Pfam protein kinase domain PF00069
AT3G32900	1.99E-02	0.93	0.96	1.13	1.20	1.22	1.25	1.03	Ulp1 protease family protein similar to At1g32840, At4g04010, At2g06430, At2g15140, At2g04980, At2g14130, At3g44500, At2g15190, At3g47260, At5g34900, At3g29210, At2g02210
AT1G11630	2.65E-02	0.87	0.89	1.06	1.20	1.22	1.36	1.12	pentatricopeptide (PPR) repeat-containing protein contains Pfam profile PF01535: PPR repeat
AT1G79530	4.31E-02	0.98	0.99	1.19	1.12	1.21	1.13	0.93	glyceraldehyde 3-phosphate dehydrogenase, cytosolic, putative / NAD-dependent glyceraldehyde-3-phosphate dehydrogenase, putative similar to glyceraldehyde-3-phosphate dehydrogenase [Pinus sylvestris] GI:1100223; contains Pfam profiles PF02800: Glyceraldehyde 3-phosphate dehydrogenase C-terminal domain, PF00044: Glyceraldehyde 3-phosphate dehydrogenase NAD binding domain
AT5G07370	3.33E-02	1.02	0.82	1.24	0.97	1.21	1.18	0.97	inositol polyphosphate 6-/3-/5-kinase 2a (IPK2a) contains Pfam domain, PF03770: Inositol polyphosphate kinase
AT4G23730	2.88E-02	0.86	0.93	1.05	1.15	1.21	1.23	1.01	aldose 1-epimerase family protein similar to apospory-associated protein C; APOC [Chlamydomonas reinhardtii] GI:6970044 Pfam profile PF01263: Aldose 1-epimerase
AT2G22930	4.51E-02	0.91	0.89	1.11	1.01	1.21	1.13	0.93	glycosyltransferase family protein contains Pfam profile: PF00201 UDP-glucoronosyl and UDP-glucosyl transferase
AT1G73150	1.39E-02	0.91	0.91	1.11	1.22	1.21	1.33	1.10	DNA-binding bromodomain-containing protein contains bromodomain, INTERPRO:IPR001487
AT5G02310	3.12E-02	0.92	0.86	1.12	1.08	1.21	1.26	1.04	eceriferum3 protein, putative similar to eceriferum3 GI:1669655 from [Arabidopsis thaliana]
AT1G13580	2.04E-02	0.89	0.90	1.09	1.05	1.21	1.17	0.96	longevity-assurance (LAG1) family protein similar to Alternaria stem canker resistance protein (ASC1) [Lycopersicon esculentum] GI:7688742; contains Pfam profile PF03798: Longevity-assurance protein (LAG1)
AT4G36460	1.99E-02	0.90	0.90	1.09	1.18	1.21	1.32	1.09	hypothetical protein
AT5G42970	1.69E-02	0.94	0.85	1.14	1.05	1.21	1.23	1.01	COP9 signalosome complex subunit 4 / CSN complex subunit 4 (CSN4) (COP8) (FUS4) FUSCA4, COP8, CSN4; identical to CSN complex subunit 4 [Arabidopsis thaliana] GI:18056659, COP8 [Arabidopsis thaliana] GI:5802627; contains Pfam profile PF01399: PCI domain; identical to cDNA CSN complex subunit 4 (CSN4) GI:18056658
AT5G01630;AT4G	1.25E-02	0.93	0.94	1.13	1.11	1.21	1.18	0.97	[AT5G01630, BRCA2 repeat-containing protein low similarity to breast cancer susceptibility protein [Gallus gallus] GI:19568157; contains Pfam profile PF00634: BRCA2 repeat];[AT4G00020, BRCA2 repeat-containing protein low similarity to SP|P51587 Breast cancer type 2 susceptibility protein [Homo sapiens]; contains Pfam profile PF00634: BRCA2 repeat]
AT3G29796	2.68E-02	0.88	0.86	1.07	1.08	1.21	1.26	1.04	hypothetical protein
AT1G35880	4.80E-02	0.88	0.94	1.06	1.08	1.21	1.15	0.95	hypothetical protein
AT2G03060	3.70E-02	0.87	0.95	1.06	1.13	1.21	1.19	0.98	MADS-box family protein
AT1G05805	3.34E-03	0.92	0.94	1.11	1.08	1.21	1.16	0.95	basic helix-loop-helix (bHLH) family protein
AT5G57800	7.98E-03	0.88	0.88	1.06	1.56	1.21	1.76	1.45	CER1 protein, putative (WAX2) similar to maize glossy1 homolog GI:2213643 from [Oryza sativa]; contains Pfam profile PF01598: Sterol desaturase
AT3G18460	3.52E-02	0.94	0.92	1.13	1.08	1.21	1.17	0.96	hypothetical protein similar to PGPS/D12 [Petunia x hybrida] GI:4105794; contains Pfam profile PF04749: Protein of unknown function, DUF614
AT1G34480	7.79E-03	0.91	0.95	1.10	1.10	1.21	1.15	0.95	DC1 domain-containing protein contains Pfam protein PF03107 DC1 domain
AT1G59780	3.29E-03	0.92	0.97	1.11	1.17	1.21	1.20	0.99	disease resistance protein (CC-NBS-LRR class), putative domain signature CC-NBS-LRR exists, suggestive of a disease resistance protein.
AT5G22360	3.39E-02	1.00	0.90	1.21	1.00	1.21	1.11	0.91	synaptobrevin family protein similar to Synaptobrevin-like protein 1 (SP:P51809) [Homo sapiens]
AT4G36480	3.12E-02	0.93	0.92	1.12	1.27	1.21	1.37	1.13	aminotransferase class I and II family protein similar to Serine palmitoyltransferase 1 (EC 2.3.1.50) from Homo sapiens [SP|O15269], Mus musculus [SP|O35704], Cricetulus griseus [SP|O54695]
AT4G12730	4.88E-02	0.96	0.73	1.17	1.03	1.21	1.42	1.17	fasciclin-like arabinogalactan protein (FLA2) identical to gi_13377778_gb_AAK20858
AT1G66960	5.33E-03	0.96	0.91	1.17	1.02	1.21	1.12	0.92	lupeol synthase, putative / 2,3-oxidosqualene-triterpenoid cyclase, putative similar to lupeol synthase GI:1762150 from [Arabidopsis thaliana], 2,3-oxidosqualene-triterpenoid cyclase [Arabidopsis thaliana] GI:2738027
AT3G04150	2.28E-02	0.92	0.88	1.11	1.13	1.21	1.29	1.06	germin-like protein, putative contains Pfam profile: PF01072 germin family; similar to germin type2 GB:CAA63023 [SP|P92996], GLP2A [SP|P92999] [Arabidopsis thaliana]
AT4G13345	4.67E-02	0.92	0.92	1.11	1.25	1.21	1.36	1.13	TMS membrane family protein / tumour differentially expressed (TDE) family protein contains Pfam domain, PF03348: TMS membrane protein/tumour differentially expressed protein (TDE)
AT1G14040	3.81E-02	0.88	0.90	1.07	1.29	1.21	1.45	1.19	EXS family protein / ERD1/XPR1/SYG1 family protein similar to PHO1 protein [Arabidopsis thaliana] GI:20069032; contains Pfam profiles PF03105: SPX domain, PF03124: EXS family
AT2G22270	2.10E-02	0.97	0.92	1.18	1.05	1.21	1.14	0.94	expressed protein
AT3G09340;AT3G	2.00E-02	0.97	0.92	1.17	1.03	1.21	1.12	0.93	[AT3G09340, amino acid transporter family protein low similarity to vesicular GABA transporter [Rattus norvegicus] GI:2587061; belongs to INTERPRO:IPR002422 amino acid/polyamine transporter, family II];[AT3G09330, amino acid transporter family protein belongs to INTERPRO:IPR002422 amino acid/polyamine transporter, family II]
AT1G48430	1.71E-02	0.97	0.94	1.18	1.18	1.21	1.25	1.04	dihydroxyacetone kinase family protein similar to dihydroxyacetone kinases; contains Pfam profiles PF02733: DAK1 domain, PF02734: DAK2 domain
AT3G54940	1.05E-02	0.91	0.92	1.10	1.10	1.21	1.20	0.99	cysteine proteinase, putative contains similarity to cysteine proteinase GI:479060 from [Glycine max]
AT3G53670	1.16E-02	0.95	0.89	1.15	1.12	1.21	1.26	1.04	expressed protein
AT1G18230	7.85E-03	0.90	0.91	1.09	1.33	1.21	1.46	1.20	octicosapeptide/Phox/Bem1p (PB1) domain-containing protein contains Pfam profile PF00564: PB1 domain
AT4G16745	4.71E-02	1.07	0.66	1.29	0.81	1.21	1.23	1.02	exostosin family protein contains Pfam PF03016: Exostosin family
AT4G38360	3.10E-02	0.93	0.89	1.12	1.25	1.21	1.41	1.16	expressed protein contains Pfam profile PF03619: Domain of unknown function
AT5G08110	4.31E-02	0.91	0.90	1.10	1.16	1.21	1.30	1.07	DEAD/DEAH box helicase, putative several putative ATP-dependent helicases
AT1G18940	3.15E-02	0.96	0.90	1.17	1.11	1.21	1.23	1.02	nodulin family protein similar to nodulin-like protein [Arabidopsis thaliana] GI:3329368, nodule-specific protein Nlj70 [Lotus japonicus] GI:3329366
AT1G73180	2.89E-02	0.94	0.92	1.14	1.11	1.21	1.20	0.99	eukaryotic translation initiation factor-related similar to eukaryotic translation initiation factor 2A (GI:21956484) [Homo sapiens]; similar to Eukaryotic translation initiation factor 3 subunit 9 (eIF-3 eta) (eIF3 p116) (eIF3 p110) (eIF3b) (Swiss-Prot:P55884) [Homo sapiens]
AT4G29690	2.05E-03	0.86	0.81	1.04	1.07	1.21	1.33	1.10	type I phosphodiesterase/nucleotide pyrophosphatase family protein similar to SP|P22413 Ectonucleotide pyrophosphatase/phosphodiesterase 1 (Plasma-cell membrane glycoprotein PC-1) [Includes: Alkaline phosphodiesterase I (EC 3.1.4.1); Nucleotide pyrophosphatase (EC 3.6.1.9) [Homo sapiens]; contains Pfam profile PF01663: Type I phosphodiesterase / nucleotide pyrophosphatase
AT4G09420	2.49E-02	0.93	0.98	1.13	1.49	1.21	1.53	1.26	disease resistance protein (TIR-NBS class), putative domain signature TIR-NBS exists, suggestive of a disease resistance protein.
AT1G64210	4.58E-02	0.96	0.87	1.16	1.14	1.21	1.31	1.08	leucine-rich repeat transmembrane protein kinase, putative contains 1 predicted transmembrane domain; similar to receptor-like protein kinase (GI:4008006) [Arabidopsis thaliana[; similar to receptor-like kinase RHG1 (GI:21239382) [Glycine max]; similar to receptor-like protein kinase 3 (GI:13506810) [Lycopersicon esculentum]
AT5G26180	3.25E-03	0.92	0.93	1.11	1.20	1.21	1.29	1.07	NOL1/NOP2/sun family protein similar to NOL1R [Homo sapiens] GI:16226071; contains Pfam profile PF01189: NOL1/NOP2/sun family
AT4G31820	3.45E-02	0.81	0.93	0.98	1.38	1.21	1.48	1.22	phototropic-responsive NPH3 family protein contains NPH3 family domain, Pfam:PF03000
AT1G09380	2.22E-02	0.94	0.82	1.14	1.16	1.21	1.41	1.17	integral membrane family protein / nodulin MtN21-related similar to MtN21 GI:2598575 (root nodule development) from [Medicago truncatula]
AT2G17750	1.35E-02	0.88	0.95	1.07	1.12	1.21	1.17	0.97	hypothetical protein contains Pfam profile PF03080: Arabidopsis proteins of unknown function
AT1G72860	2.47E-02	0.93	0.88	1.12	1.09	1.21	1.23	1.02	disease resistance protein (TIR-NBS-LRR class), putative domain signature TIR-NBS-LRR exists, suggestive of a disease resistance protein.
AT3G26420;AT3G	3.39E-02	1.04	0.91	1.25	1.03	1.21	1.13	0.94	[AT3G26420, glycine-rich RNA-binding protein similar to RNA-binding protein (RZ-1) GB:BAA12064 [Nicotiana sylvestris]; contains Pfam profile: PF00076 RNA recognition motif. (a.k.a. RRM, RBD, or RNP domain)];[AT3G26430, GDSL-motif lipase/hydrolase family protein similar to early nodulin ENOD8 [Medicago sativa] GI:304037; contains InterPro Entry IPR001087 Lipolytic enzyme, G-D-S-L family]
AT2G40050	2.79E-02	0.92	0.97	1.11	1.05	1.21	1.08	0.90	DC1 domain-containing protein contains Pfam profile PF03107: DC1 domain
AT5G17630;AT5G	4.78E-02	0.91	0.84	1.10	1.01	1.21	1.19	0.99	[AT5G17630, glucose-6-phosphate/phosphate translocator, putative similar to glucose-6-phosphate/phosphate-translocator precursor [Solanum tuberosum] gi|2997593|gb|AAC08526];[AT5G17640, expressed protein]
AT4G04920	3.75E-02	0.86	0.97	1.03	1.14	1.21	1.17	0.97	expressed protein
AT4G35380	2.17E-02	0.89	0.96	1.07	1.28	1.21	1.33	1.10	guanine nucleotide exchange family protein similar to guanine nucleotide exchange factor [Homo sapiens] GI:5456754; contains Pfam profile PF01369: Sec7 domain
AT2G25160	2.38E-02	0.96	0.97	1.16	1.00	1.21	1.04	0.86	cytochrome P450, putative similar to cytochrome p450(CYP82C1p) GI:2739004 from [Glycine max]
AT3G31430	3.85E-02	0.95	0.94	1.15	1.11	1.21	1.18	0.98	hypothetical protein low similarity to zinc finger protein [Arabidopsis thaliana] GI:976277
AT4G07480	4.71E-02	0.97	0.99	1.18	1.13	1.21	1.13	0.94	hypothetical protein
AT2G22510	4.71E-02	0.94	0.98	1.14	1.07	1.21	1.10	0.91	hydroxyproline-rich glycoprotein family protein similar to proline-rich cell wall protein [Gossypium barbadense] gi|451544|gb|AAA79364; contains proline-rich extensin domains, INTERPRO:IPR002965
AT1G33250	2.21E-02	0.92	0.90	1.10	1.19	1.21	1.32	1.09	fringe-related protein + weak similarity to Fringe [Schistocerca gregaria](GI:6573138);Fringe encodes an extracellular protein that regulates Notch signalling.
AT3G16310	4.22E-02	0.88	0.89	1.07	1.08	1.21	1.21	1.00	mitotic phosphoprotein N' end (MPPN) family protein contains Pfam profile PF05172: MPPN (Mitotic PhosphoProtein N' end) (rrm-like) domain
AT5G07660	4.04E-03	0.91	0.93	1.10	1.12	1.21	1.20	1.00	structural maintenance of chromosomes (SMC) family protein similar to SMC-like protein (MIM) [Arabidopsis thaliana] GI:5880634; contains Pfam profile PF02463: RecF/RecN/SMC N terminal domain
AT5G67220	2.84E-02	0.99	0.86	1.20	1.03	1.21	1.19	0.99	nitrogen regulation family protein similar to unknown protein (gb|AAF51525.1) ; contains Pfam domain PF01207: Dihydrouridine synthase (Dus); similar to (SP:P45672) NIFR3-like protein (SP:P45672) [Azospirillum brasilense]
AT4G25770	8.42E-03	0.99	0.89	1.19	1.03	1.21	1.15	0.96	expressed protein
AT5G48130	3.74E-02	0.98	0.88	1.18	1.04	1.21	1.19	0.99	phototropic-responsive NPH3 family protein contains NPH3 family domain, Pfam:PF03000
AT5G63680	3.88E-02	0.96	0.87	1.15	1.14	1.21	1.31	1.09	pyruvate kinase, putative similar to pyruvate kinase, cytosolic isozyme [Glycine max] SWISS-PROT:Q42806
AT1G21160	4.71E-02	0.94	0.93	1.13	1.31	1.20	1.41	1.17	eukaryotic translation initiation factor 2 family protein / eIF-2 family protein similar to SP|O60841 Translation initiation factor IF-2 [Homo sapiens]; contains Pfam profiles PF00009: Elongation factor Tu GTP binding domain, PF03144: Elongation factor Tu domain 2
AT3G16510	4.58E-03	0.87	0.82	1.05	1.13	1.20	1.37	1.14	reversibly glycosylated polypeptide, putative similar to reversibly glycosylatable polypeptide (RGP1) [Pisum sativum] GI:2130521; contains Pfam profile PF03214: Reversibly glycosylated polypeptide
AT1G22730	2.90E-02	0.96	0.84	1.16	1.11	1.20	1.32	1.09	MA3 domain-containing protein contains Pfam profile PF02847: MA3 domain; low similarity to programmed cell death 4 protein [Gallus gallus] GI:12958564
AT4G24480	3.63E-02	0.96	0.92	1.16	1.13	1.20	1.23	1.02	serine/threonine protein kinase, putative similar to serine/threonine-protein kinase CTR1 [Arabidopsis thaliana] SWISS-PROT:Q05609
AT3G62780	4.81E-02	0.91	0.93	1.10	1.04	1.20	1.12	0.93	C2 domain-containing protein contains similarity to shock protein SRC2 [Glycine max] gi|2055230|dbj|BAA19769 ; contains Pfam profile PF00168:C2 domain
AT5G23590	7.20E-03	1.02	0.83	1.22	1.04	1.20	1.25	1.04	DNAJ heat shock N-terminal domain-containing protein similar to SP|P39101 CAJ1 protein Saccharomyces cerevisiae; contains Pfam profile PF00226 DnaJ domain
AT5G63920	8.74E-03	0.90	0.96	1.09	1.31	1.20	1.37	1.13	DNA topoisomerase III alpha, putative similar to Swiss-Prot:Q9NG98 DNA topoisomerase III alpha [Drosophila melanogaster]
AT4G28020	3.56E-02	0.98	0.93	1.18	1.22	1.20	1.32	1.10	expressed protein contains Pfam PF01980: Uncharacterised protein family
AT3G62890	2.89E-02	0.94	0.95	1.13	1.10	1.20	1.15	0.96	pentatricopeptide (PPR) repeat-containing protein contains Pfam profile PF01535: PPR repeat
AT1G34670	4.51E-02	1.00	0.93	1.20	1.04	1.20	1.12	0.93	myb family transcription factor similar to myb-related protein mixta GI:485867 from [Antirrhinum majus]
AT2G36530	8.24E-03	0.77	1.11	0.93	1.40	1.20	1.26	1.05	enolase identical to SWISS-PROT:P25696 enolase (EC 4.2.1.11) (2-phosphoglycerate dehydratase)(2-phospho-D- glycerate hydro-lyase) [Arabidopsis thaliana]
AT4G35850	1.43E-02	0.97	0.83	1.17	1.02	1.20	1.23	1.02	pentatricopeptide (PPR) repeat-containing protein low similarity to CRP1 [Zea mays] GI:3289002; contains Pfam profile PF01535: PPR repeat
AT2G29260	4.24E-02	1.01	0.80	1.21	1.00	1.20	1.24	1.03	tropinone reductase, putative / tropine dehydrogenase, putative similar to tropinone reductase SP:P50165 from [Datura stramonium]
AT3G08490	2.89E-02	0.92	0.90	1.11	1.29	1.20	1.44	1.19	hypothetical protein
AT2G28580	1.22E-03	0.90	0.91	1.08	1.27	1.20	1.39	1.16	hypothetical protein contains Pfam profile PF03140: Plant protein of unknown function
AT5G61370	8.95E-03	1.01	0.80	1.21	1.05	1.20	1.31	1.09	pentatricopeptide (PPR) repeat-containing protein contains Pfam profile PF01535: PPR repeat
AT5G04930	4.88E-02	0.96	0.93	1.15	1.05	1.20	1.14	0.95	fatty acid desaturase family protein similar to D. melanogaster Des-1 protein, GenBank accession number X94180; contains Pfam profile PF00487 Fatty acid desaturase domain
AT5G61530	1.87E-02	0.97	0.88	1.17	1.17	1.20	1.32	1.10	small G protein family protein / RhoGAP family protein contains Pfam domain, PF00620: RhoGAP domain
AT3G60570	1.10E-02	0.94	0.90	1.13	1.12	1.20	1.24	1.03	beta-expansin, putative (EXPB5) contains similarity to beta-expansin GI:8118428 from [Oryza sativa]; beta-expansin gene family, PMID:11641069
AT1G49800	3.95E-02	0.97	0.91	1.17	1.01	1.20	1.12	0.93	hypothetical protein
AT5G19580	1.44E-02	0.91	0.90	1.10	1.16	1.20	1.30	1.08	glyoxal oxidase-related contains similarity to glyoxal oxidase precursor [Phanerochaete chrysosporium] gi|1050302|gb|AAA87594
AT1G79890	7.77E-03	0.96	0.93	1.15	1.10	1.20	1.18	0.98	helicase-related similar to CHL1 potential helicase protein (GI:2632247) [Homo sapiens]; similar to helicase GB:AAB06962 [Homo sapiens]
AT5G37850	2.74E-03	0.95	0.91	1.14	1.08	1.20	1.19	0.99	pfkB-type carbohydrate kinase family protein contains Pfam profile: PF00294 pfkB family carbohydrate kinase
AT1G50440	9.82E-03	0.90	0.87	1.09	1.15	1.20	1.31	1.09	zinc finger (C3HC4-type RING finger) family protein contains Pfam profile: PF00097 zinc finger, C3HC4 type (RING finger)
AT2G21790	4.10E-02	0.92	0.86	1.11	1.23	1.20	1.43	1.19	ribonucleoside-diphosphate reductase small chain, putative / ribonucleotide reductase, putative similar to ribonucleotide reductase GI:4151068 from [Nicotiana tabacum]
AT5G48120	4.57E-02	0.93	0.83	1.11	1.41	1.20	1.70	1.41	expressed protein low similarity to MMS19 [Mus musculus] GI:14029390
AT2G27810	3.25E-02	0.91	0.85	1.10	1.04	1.20	1.22	1.02	xanthine/uracil permease family protein contains Pfam profile: PF00860 permease family
AT4G03110	1.94E-03	0.86	0.87	1.04	1.35	1.20	1.54	1.28	RNA-binding protein, putative similar to Etr-1 [Danio rerio] GI:7670536, BRUNO-like 6 RNA-binding protein [Homo sapiens] GI:15341327, CUG-BP and ETR-3 like factor 3 [Homo sapiens] GI:12746392; contains InterPro entry IPR000504: RNA-binding region RNP-1 (RNA recognition motif) (RRM)
AT4G11440	1.33E-02	0.93	0.96	1.11	1.41	1.20	1.47	1.23	mitochondrial substrate carrier family protein
AT2G33670	2.79E-02	0.95	0.88	1.14	1.08	1.20	1.22	1.02	seven transmembrane MLO family protein / MLO-like protein 5 (MLO5) identical to MLO-like protein 5 (AtMlo5) [Arabidopsis thaliana[SWISS-PROT:O22815; similar to MLO protein SWISS-PROT:P93766, NCBI_gi:1877221 [Hordeum vulgare][Barley]
AT4G21440	1.86E-02	0.97	0.98	1.16	1.03	1.20	1.05	0.88	myb family transcription factor (MYB102) contains Pfam profile: PF00249 myb-like DNA-binding domain
AT4G03010	3.72E-02	0.90	0.96	1.08	1.07	1.20	1.11	0.93	leucine-rich repeat family protein contains leucine rich-repeat domains Pfam:PF00560, INTERPRO:IPR001611; contains similarity to Hcr2-0A [Lycopersicon esculentum] gi|3894385|gb|AAC78592
AT5G42690	2.07E-02	0.94	0.92	1.13	1.11	1.20	1.21	1.01	expressed protein contains Pfam profile PF04784: Protein of unknown function, DUF547; expression supported by MPSS
AT5G17960	3.63E-02	0.96	0.86	1.15	1.02	1.20	1.19	0.99	DC1 domain-containing protein contains Pfam profile PF03107: DC1 domain
AT4G39000	4.00E-02	0.87	0.97	1.04	1.10	1.20	1.13	0.94	glycosyl hydrolase family 9 protein endo-1,4-beta-glucanase precursor - Fragariax ananassa, PID:g3549291
AT1G74040	4.04E-02	0.95	0.92	1.14	1.20	1.20	1.30	1.08	2-isopropylmalate synthase 1 (IMS1) identical to 2-isopropylmalate synthase (IMS1) [Arabidopsis thaliana[GI:12330687; identical to cDNA 2-isopropylmalate synthase (IMS1) GI:12330686
AT5G14950	1.48E-02	0.91	0.89	1.09	1.32	1.20	1.49	1.25	glycosyl hydrolase family 38 protein similar to alpha-mannosidase II SP:P27046 from [Mus musculus]
AT4G31420	1.60E-02	1.00	0.83	1.20	1.03	1.20	1.23	1.03	zinc finger (C2H2 type) family protein contains Pfam profile: PF00096 zinc finger, C2H2 type
AT1G54955	4.81E-02	0.96	0.93	1.15	1.02	1.20	1.09	0.91	hypothetical protein
AT2G31955	4.11E-02	0.92	0.98	1.10	1.38	1.20	1.41	1.18	molybdopterin biosynthesis protein, putative / molybdenum cofactor biosynthesis enzyme, putative 3' fragment; strong similarity to SP|Q39055 Molybdopterin biosynthesis CNX2 protein (Molybdenum cofactor biosynthesis enzyme CNX2) [Arabidopsis thaliana]
AT1G05510	3.15E-03	0.93	0.88	1.11	1.03	1.20	1.17	0.97	expressed protein
AT5G05400	4.70E-03	0.93	0.94	1.12	1.12	1.20	1.19	0.99	disease resistance protein (CC-NBS-LRR class), putative domain signature CC-NBS-LRR exists, suggestive of a disease resistance protein.
AT3G18670	4.27E-02	0.96	0.92	1.15	1.07	1.20	1.16	0.97	ankyrin repeat family protein contains ankyrin repeats, Pfam domain PF00023
AT1G42440	1.23E-02	0.88	0.93	1.05	1.41	1.20	1.52	1.27	expressed protein contains Pfam domain, PF04950: Protein of unknown function (DUF663)
AT1G04900	3.12E-02	0.95	0.80	1.14	1.16	1.20	1.44	1.20	expressed protein contains Pfam PF02636: Uncharacterized ACR, COG1565
AT4G29200	2.80E-02	0.88	0.91	1.06	1.15	1.20	1.25	1.05	hypothetical protein
AT1G21610	2.11E-02	1.01	0.81	1.21	1.14	1.20	1.41	1.18	wound-responsive family protein similar to wound-responsive protein 14.05 (GI:16506638) [Castanea sativa]; ESTs gb T42839 and gb|AA395192 come from this gene
AT3G13760	2.48E-02	0.93	0.91	1.11	1.09	1.20	1.19	1.00	DC1 domain-containing protein contains Pfam profile PF03107: DC1 domain
AT5G35580	2.28E-02	0.96	0.92	1.15	1.02	1.20	1.10	0.92	protein kinase, putative similar to auxin-regulated dual specificity cytosolic kinase [Lycopersicon esculentum] gi|14484938|gb|AAK62821
AT1G66660	3.08E-02	0.95	0.87	1.14	0.99	1.20	1.14	0.95	seven in absentia (SINA) protein, putative similar to SIAH2 protein [Brassica napus var. napus] GI:7657878; contains Pfam profile PF03145: Seven in absentia protein family
AT4G27650	3.87E-03	0.95	0.94	1.14	1.06	1.20	1.12	0.93	pelota (PEL1) identical to pelota [Arabidopsis thaliana] GI:3941543; contains InterPro accession IPR004403: Peptide chain release factor eRF/aRF subunit 1
AT1G55420;AT1G	1.33E-02	0.94	0.96	1.13	1.17	1.20	1.22	1.02	[AT1G55420, DC1 domain-containing protein contains Pfam profile PF03107: DC1 domain];[AT1G55380, DC1 domain-containing protein contains Pfam profile PF03107: DC1 domain]
AT2G13650	2.43E-02	0.81	0.98	0.97	1.36	1.20	1.40	1.17	GDP-mannose transporter (GONST1) identical to GDP-mannose transporter SP:Q941R4 from [Arabidopsis thaliana]
AT2G22910	3.25E-02	0.91	0.89	1.09	1.19	1.20	1.33	1.11	GCN5-related N-acetyltransferase (GNAT) family protein / amino acid kinase family protein similar to SP|P08205 Amino-acid acetyltransferase (EC 2.3.1.1) (N-acetylglutamate synthase) [Escherichia coli]; contains Pfam profiles PF00696: Amino acid kinase family, PF00583: acetyltransferase, GNAT family
AT1G34360	5.62E-03	0.92	0.91	1.10	1.09	1.20	1.19	0.99	translation initiation factor 3 (IF-3) family protein low similarity to Translation initiation factor IF-3 from [subsp. Schizaphis graminum] (Buchnera aphidicola) SP|P46243, [Salmonella typhimurium] SP|P33321; contains Pfam profiles PF05198: Translation initiation factor IF-3 N-terminal domain, PF00707: Translation initiation factor IF-3 C-terminal domain
AT5G20390	1.88E-02	0.98	0.90	1.17	1.10	1.20	1.22	1.02	beta-1,3-glucanase, putative similar to plant beta-1,3-glucanase bg4 GI:2808438 from [Arabidopsis thaliana]
AT5G42420	2.31E-02	0.91	0.86	1.09	1.05	1.20	1.23	1.03	transporter-related low similarity to phosphoenolpyruvate/phosphate translocator precursor [Mesembryanthemum crystallinum] GI:9295275, glucose-6-phosphate/phosphate-translocator precursor [Solanum tuberosum] GI:2997593
AT3G19440	4.64E-03	0.88	0.89	1.05	1.14	1.20	1.28	1.07	pseudouridine synthase family protein low similarity to SP|P23851 Ribosomal large subunit pseudouridine synthase C (EC 4.2.1.70) (Pseudouridylate synthase) (Uracil hydrolase) [Escherichia coli]; contains Pfam profile PF00849: RNA pseudouridylate synthase
AT1G30540	2.74E-02	1.01	0.88	1.21	1.07	1.19	1.22	1.02	ATPase, BadF/BadG/BcrA/BcrD-type family contains Pfam PF01869: BadF/BadG/BcrA/BcrD ATPase family; similar to Transcriptional regulators of NagC/XylR (ROK) family, sugar kinase (GI:15023013) [Clostridium acetobutylicum]; similar to N-acetylglucosamine kinase (EC 2.7.1.59) (GlcNAc kinase) (Swiss-Prot:Q9UJ70) [Homo sapiens]
AT1G17690	4.61E-02	0.97	0.89	1.16	1.16	1.19	1.29	1.08	expressed protein
AT2G25520	1.86E-02	0.95	0.94	1.13	1.15	1.19	1.23	1.03	phosphate translocator-related low similarity to SP|P52178 Triose phosphate/phosphate translocator, non-green plastid, chloroplast precursor (CTPT) [Brassica oleracea], phosphoenolpyruvate/phosphate translocator precursor [Mesembryanthemum crystallinum] GI:9295275
AT3G08840	1.77E-02	0.95	0.76	1.14	1.20	1.19	1.57	1.32	D-alanine--D-alanine ligase family similar to D-alanine--D-alanine ligase (EC 6.3.2.4) (D-alanylalanine synthetase) (D-Ala-D-Ala ligase) (Swiss-Prot:O51218) [Borrelia burgdorferi]; similar to D-alanine--D-alanine ligase (EC 6.3.2.4) (D-alanylalanine synthetase) (D-Ala-D-Ala ligase). (Swiss-Prot:P95803) [Streptococcus mutans]
AT1G27000	1.11E-02	0.92	0.89	1.10	1.10	1.19	1.23	1.03	bZIP family transcription factor
AT4G22720	1.49E-02	0.95	0.93	1.13	1.03	1.19	1.11	0.93	glycoprotease M22 family protein similar to sialylglycoprotease [Haemophilus ducreyi] GI:6942294; contains Pfam profile PF00814: Glycoprotease family
AT1G65040	3.52E-02	0.99	0.76	1.18	1.09	1.19	1.43	1.20	zinc finger (C3HC4-type RING finger) family protein contains Pfam profile: PF00097 zinc finger, C3HC4 type (RING finger)
AT1G69350	1.59E-02	0.92	0.96	1.10	1.15	1.19	1.20	1.01	pentatricopeptide (PPR) repeat-containing protein contains Pfam profile: PF01535: PPR repeat
AT5G11850	7.28E-03	0.93	0.89	1.11	1.33	1.19	1.50	1.25	protein kinase family protein contains Pfam domain, PF00069: Protein kinase domain; identical to cDNA MAP3K delta-1 protein kinase GI:2253009
AT4G08640	3.70E-02	0.88	0.95	1.05	1.04	1.19	1.09	0.92	hypothetical protein low similarity to chaperonin-containing-TCP1 theta subunit from Tetrahymena pyriformis [GI:4959731], Homo sapiens [SP|P50990]
AT5G38130	4.00E-02	0.94	0.94	1.12	1.00	1.19	1.06	0.89	transferase family protein similar to anthranilate N-hydroxycinnamoyl/benzoyltransferase, Dianthus caryophyllus [gi:2239091]; contains Pfam transferase family domain PF002458
AT3G21310	4.00E-02	0.89	0.95	1.06	1.14	1.19	1.20	1.00	expressed protein contains Pfam profile PF03267: Arabidopsis protein of unknown function, DUF266
AT2G30940	4.15E-02	0.96	0.91	1.15	1.03	1.19	1.13	0.95	protein kinase family protein contains protein kinase domain, Pfam:PF00069
AT1G09300	1.09E-02	0.91	0.92	1.09	1.25	1.19	1.36	1.14	metallopeptidase M24 family protein similar to SP|P15034 Xaa-Pro aminopeptidase (EC 3.4.11.9) (X-Pro aminopeptidase) (Aminopeptidase P II) (Aminoacylproline aminopeptidase) [Escherichia coli]; contains Pfam profiles PF00557: metallopeptidase family M24, PF05195: Aminopeptidase P, N-terminal domain
AT3G57420	2.85E-02	0.96	0.82	1.15	1.19	1.19	1.46	1.22	expressed protein contains Pfam domain PF03385: Protein of unknown function, DUF288
AT2G35230	4.43E-02	0.72	1.34	0.86	1.83	1.19	1.36	1.14	VQ motif-containing protein contains PF05678: VQ motif
AT5G50270	4.14E-02	0.91	0.93	1.09	1.13	1.19	1.21	1.02	F-box family protein contains F-box domain Pfam:PF00646
AT2G39080	1.69E-02	0.65	1.28	0.77	1.45	1.19	1.14	0.95	expressed protein
AT1G73490	3.52E-02	0.91	0.92	1.09	1.00	1.19	1.09	0.91	RNA recognition motif (RRM)-containing protein contains INTERPRO:IPR000504 RNA-binding region RNP-1 (RNA recognition motif) domain
AT4G23070	2.04E-02	0.94	0.88	1.12	1.09	1.19	1.24	1.03	rhomboid family protein contains PFAM domain PF01694, Rhomboid family
AT1G13990	3.78E-02	0.99	0.67	1.18	0.91	1.19	1.36	1.14	expressed protein
AT4G19550	4.32E-02	0.94	0.96	1.12	1.00	1.19	1.04	0.88	expressed protein
AT2G43430	1.48E-02	0.69	1.18	0.82	1.40	1.19	1.19	1.00	hydroxyacylglutathione hydrolase, mitochondrial / glyoxalase II (GLX2-1) identical to SP|O24495 Hydroxyacylglutathione hydrolase, mitochondrial precursor (EC 3.1.2.6) (Glyoxalase II) (Glx II) [Arabidopsis thaliana]
AT1G10980	1.24E-02	0.94	0.96	1.11	1.21	1.19	1.26	1.06	expressed protein ; expression supported by MPSS
AT5G56920	1.39E-02	0.95	0.92	1.14	1.11	1.19	1.21	1.01	expressed protein
AT1G04870	3.98E-02	1.01	0.87	1.20	1.07	1.19	1.22	1.03	protein arginine N-methyltransferase family protein similar to SP|Q96LA8 Protein arginine N-methyltransferase 6 (EC 2.1.1.-) [Homo sapiens]
AT1G54990	2.64E-02	0.89	0.97	1.07	1.29	1.19	1.33	1.11	expressed protein
AT4G02220	9.64E-03	0.92	0.93	1.10	1.17	1.19	1.26	1.06	zinc finger (MYND type) family protein / programmed cell death 2 C-terminal domain-containing protein similar to SP|Q16342 Programmed cell death protein 2 (Zinc finger protein Rp-8) [Homo sapiens]; contains Pfam profiles PF01753: MYND finger, PF04194: Programmed cell death protein 2, C-terminal putative domain
AT1G35290	3.28E-02	0.90	0.94	1.07	1.09	1.19	1.17	0.98	thioesterase family protein contains Pfam profile PF03061: thioesterase family protein
AT4G32140	1.25E-02	0.94	0.90	1.12	1.17	1.19	1.30	1.09	expressed protein
AT3G11010	3.72E-02	0.93	0.91	1.11	1.02	1.19	1.12	0.94	disease resistance family protein / LRR family protein contains leucine rich-repeat domains Pfam:PF00560, INTERPRO:IPR001611; similar to disease resistance protein [Lycopersicon esculentum] gi|3894383|gb|AAC78591
AT2G07290	3.83E-02	0.91	0.92	1.08	1.13	1.19	1.23	1.03	hypothetical protein
AT1G23450	4.63E-02	0.93	0.98	1.11	1.12	1.19	1.15	0.96	pentatricopeptide (PPR) repeat-containing protein contains INTERPRO:IPR002885 PPR repeats
AT2G34210	1.14E-02	0.92	0.86	1.09	1.04	1.19	1.21	1.02	KOW domain-containing transcription factor family protein
AT1G21810	2.65E-02	0.88	0.99	1.05	1.07	1.19	1.09	0.91	expressed protein
AT4G37130	2.47E-02	0.95	0.89	1.13	1.10	1.19	1.23	1.04	hydroxyproline-rich glycoprotein family protein
AT5G52040	4.91E-02	1.01	0.88	1.20	1.03	1.19	1.18	0.99	arginine/serine-rich splicing factor RSP41 (RSP41) nearly identical to SP|P92966 Arginine/serine-rich splicing factor RSP41 [Arabidopsis thaliana]
AT1G29820	1.35E-02	0.97	0.84	1.15	1.02	1.19	1.21	1.02	expressed protein
AT4G25340	2.77E-02	0.96	0.88	1.14	1.09	1.19	1.24	1.04	immunophilin-related / FKBP-type peptidyl-prolyl cis-trans isomerase-related immunophilin FKBP46 - Spodoptera frugiperda (fall armyworm),PIR2:A55320
AT1G65340	4.27E-02	0.96	0.92	1.14	1.06	1.19	1.15	0.97	cytochrome P450, putative similar to cytochrome P450 GI:4688670 from [Catharanthus roseus]
AT5G14940	4.40E-02	0.87	0.98	1.04	1.21	1.19	1.23	1.04	proton-dependent oligopeptide transport (POT) family protein contains Pfam profile: PF00854 POT family
AT5G11210	2.89E-02	0.93	0.94	1.11	1.04	1.19	1.11	0.93	glutamate receptor family protein (GLR2.5) plant glutamate receptor family, PMID:11379626
AT4G13360	6.55E-03	1.04	0.86	1.24	0.98	1.19	1.13	0.95	enoyl-CoA hydratase/isomerase family protein similar to CoA-thioester hydrolase CHY1 (beta-hydroxyisobutyryl-CoA hydrolase) [Arabidopsis thaliana] GI:8572760; contains Pfam profile PF00378: enoyl-CoA hydratase/isomerase family protein
AT5G13710	9.88E-03	1.01	0.77	1.20	1.01	1.19	1.32	1.11	sterol 24-C-methyltransferase, putative similar to SP:P25087 Sterol 24-C-methyltransferase, Delta(24)-sterol C- methyltransferase, Saccharomyces cerevisiae
AT3G10670	2.43E-02	1.03	0.90	1.22	0.96	1.19	1.07	0.90	ABC transporter family protein similar to ABC transporter ATPase GB:AAC68280 [Chlamydia trachomatis]
AT4G36920	4.24E-02	0.93	0.77	1.10	1.08	1.19	1.40	1.17	floral homeotic protein APETALA2 (AP2) identical to (SP:P47927) Floral homeotic protein APETALA2. [Mouse-ear cress] [Arabidopsis thaliana]
AT3G06810	3.29E-02	0.97	0.90	1.15	1.08	1.19	1.20	1.01	acyl-CoA dehydrogenase-related low similarity to acyl-CoA dehydrogenase [Acinetobacter sp. NCIMB9871] GI:14587418; contains Pfam profiles PF01636: Phosphotransferase enzyme family, PF00441: Acyl-CoA dehydrogenase C-terminal domain, PF02770: Acyl-CoA dehydrogenase middle domain
AT1G69020	3.14E-02	0.89	0.96	1.05	1.42	1.19	1.48	1.24	prolyl oligopeptidase family protein similar to SP|Q59536 Protease II (EC 3.4.21.83) (Oligopeptidase B) [Moraxella lacunata]; contains Pfam profiles PF00326: prolyl oligopeptidase family, PF02897: Prolyl oligopeptidase, N-terminal beta-propeller domain; contains non-consensus GA donor splice site at intron 5
AT1G58190	2.90E-02	0.93	0.89	1.11	1.09	1.19	1.22	1.03	leucine-rich repeat family protein contains leucine rich-repeat (LRR) domains Pfam:PF00560, INTERPRO:IPR001611; contains similarity to Cf-2.2 [Lycopersicon pimpinellifolium] gi|1184077|gb|AAC15780
AT1G76390	3.60E-02	0.96	0.78	1.14	1.21	1.19	1.55	1.31	armadillo/beta-catenin repeat family protein / U-box domain-containing protein contains Pfam domain, PF00514: Armadillo/beta-catenin-like repeats and Pfam, PF04564: U-box domain
AT3G05430	7.70E-03	0.94	0.89	1.11	1.17	1.19	1.31	1.10	PWWP domain-containing protein contains Pfam profile:PF00855 PWWP domain
AT1G62730	3.61E-02	0.97	0.87	1.15	0.99	1.19	1.14	0.96	expressed protein
AT5G23150	2.13E-02	0.94	0.90	1.11	1.20	1.19	1.34	1.13	PWWP domain-containing protein identical to cDNA putative transcription factor (HUA2) GI:4868119; contains Pfam profile PF00855: PWWP domain
AT3G12100	3.09E-03	0.91	0.87	1.08	1.16	1.19	1.34	1.13	cation efflux family protein / metal tolerance protein, putative member of the cation diffusion facilitator (CDF) family, or cation efflux (CE) family, PMID:11500563
AT1G64830	2.68E-02	0.94	0.95	1.12	1.09	1.19	1.14	0.96	aspartyl protease family protein contains Pfam domain, PF00026: eukaryotic aspartyl protease
AT3G44970	4.45E-02	1.05	0.75	1.25	0.92	1.19	1.22	1.03	cytochrome P450 family protein similar to Cytochrome P450 85 (SP:Q43147) [Lycopersicon esculentum]; contains Pfam profile: PF00067 cytochrome P450
AT1G03790	4.44E-02	0.89	0.94	1.06	1.04	1.19	1.10	0.93	zinc finger (CCCH-type) family protein contains Pfam domain, PF00642: Zinc finger C-x8-C-x5-C-x3-H type (and similar)
AT1G24360	3.98E-02	0.93	0.90	1.11	1.11	1.19	1.24	1.05	3-oxoacyl-[acyl-carrier protein] reductase, chloroplast / 3-ketoacyl-acyl carrier protein reductase identical to 3-oxoacyl-[acyl-carrier protein] reductase SP:P33207 from [Arabidopsis thaliana]
AT4G02300	1.05E-02	0.95	0.90	1.13	1.07	1.19	1.18	1.00	pectinesterase family protein contains Pfam profile: PF01095 pectinesterase
AT2G07280	2.78E-02	0.96	0.93	1.14	1.09	1.19	1.17	0.99	hypothetical protein
AT5G39260	2.50E-02	0.96	0.87	1.14	1.02	1.19	1.17	0.99	expansin, putative (EXP21) similar to alpha-expansin GI:6573157 from [Regnellidium diphyllum[; alpha-expansin gene family, PMID:11641069
AT1G66210	4.31E-02	0.93	0.96	1.10	1.04	1.19	1.09	0.91	subtilase family protein contains similarity to subtilase; SP1 GI:9957714 from [Oryza sativa]
AT3G53710	2.52E-02	1.00	0.86	1.18	1.08	1.19	1.26	1.06	ARF GAP-like zinc finger-containing protein ZIGA2 (ZIGA2) nearly identical to ARF GAP-like zinc finger-containing protein ZiGA2 from GI:10441356 [Arabidopsis thaliana]; contains InterPro accession IPR001164: Human Rev interacting-like protein (hRIP)
AT2G17420	2.08E-02	1.01	0.92	1.20	1.03	1.19	1.12	0.94	thioredoxin reductase 2 / NADPH-dependent thioredoxin reductase 2 (NTR2) identical to SP|Q39242
AT4G27400	4.76E-02	0.95	0.89	1.12	1.08	1.19	1.22	1.03	late embryogenesis abundant protein-related / LEA protein-related similar to late embryogenesis abundant protein [Picea glauca] GI:1350543
AT1G27940	3.23E-02	0.98	0.94	1.16	1.03	1.19	1.09	0.92	multidrug resistance P-glycoprotein, putative similar to mdr-like P-glycoprotein atpgp1 GI:3849833 from [Arabidopsis thaliana]
AT1G47220	2.40E-02	0.94	0.91	1.12	1.06	1.19	1.16	0.98	cyclin, putative similar to cyclin A-like protein [Nicotiana tabacum] GI:1064931, A-type cyclin [Catharanthus roseus] GI:2190259; contains Pfam profiles PF00134: Cyclin, N-terminal domain, PF02984: Cyclin, C-terminal domain
AT2G47550	3.81E-02	0.95	0.94	1.13	1.00	1.19	1.07	0.90	pectinesterase family protein contains Pfam profile: PF01095 pectinesterase
AT3G51460	4.41E-02	0.95	0.90	1.12	1.16	1.19	1.28	1.08	phosphoinositide phosphatase family protein contains similarity to phosphoinositide phosphatase SAC1 [Rattus norvegicus] gi|11095248|gb|AAG29810; contains Pfam domain, PF02383: SacI homology domain; contains non-consensus AT-AC splice sites at intron 8
AT1G56020	1.57E-02	0.88	0.88	1.05	1.21	1.19	1.38	1.16	expressed protein
AT5G52000	2.22E-02	0.98	0.99	1.16	1.07	1.19	1.09	0.92	importin alpha-1 subunit, putative similar to importin alpha-1 subunit (Karyopherin alpha-1 subunit, KAP alpha) [Arabidopsis thaliana] SWISS-PROT:Q96321
AT5G23840	1.01E-02	0.98	0.89	1.16	1.05	1.19	1.17	0.99	MD-2-related lipid recognition domain-containing protein / ML domain-containing protein contains Pfam profile PF02221: ML domain
AT2G33760	4.82E-02	0.97	0.91	1.15	1.08	1.19	1.19	1.00	pentatricopeptide (PPR) repeat-containing protein contains INTERPRO:IPR002885 PPR repeats
AT4G31090	4.51E-02	0.95	0.91	1.13	1.09	1.19	1.19	1.01	expressed protein
AT2G30650	1.95E-02	0.90	0.97	1.07	0.99	1.18	1.01	0.86	3-hydroxyisobutyryl-coenzyme A hydrolase, putative / CoA-thioester hydrolase, putative strong similarity to gi:8572760; contains Pfam profile PF00388 enoyl-CoA hydratase/isomerase family protein
AT3G07000	4.99E-02	0.93	0.89	1.10	1.02	1.18	1.14	0.96	DC1 domain-containing protein contains Pfam profile PF03107: DC1 domain
AT5G45560	4.93E-02	0.94	0.98	1.11	1.16	1.18	1.19	1.01	pleckstrin homology (PH) domain-containing protein / lipid-binding START domain-containing protein weak similarity to SP|P79245 Steroidogenic acute regulatory protein, mitochondrial precursor (StAR) [Ovis aries]; contains Pfam profiles PF01852: START domain, PF00169: PH domain
AT1G11650	1.56E-02	0.94	0.87	1.11	1.08	1.18	1.24	1.05	RNA-binding protein 45 (RBP45), putative similar to gb|U90212 DNA binding protein ACBF from Nicotiana tabacum and contains 3 PF|00076 RNA recognition motif domains. ESTs gb|T44278, gb|R65195, gb|N65904, gb|H37499, gb|R90487, gb|N95952, gb|T44278, gb|Z20166, gb|N96891, gb|W43137, gb|F15504, gb|F1
AT5G01840	2.23E-03	0.93	0.90	1.10	1.25	1.18	1.39	1.17	ovate family protein 59% similar to ovate protein (GI:23429649) [Lycopersicon esculentum]; contains TIGRFAM TIGR01568 : uncharacterized plant-specific domain TIGR01568
AT3G51050	4.71E-02	0.89	0.90	1.05	1.18	1.18	1.31	1.11	FG-GAP repeat-containing protein
AT4G37340	4.57E-02	0.94	0.91	1.12	1.06	1.18	1.17	0.99	cytochrome P450 family protein Similar to Cytochrome P450 91A1 (SP:Q9FG65) [Arabidopsis thaliana];
AT1G25210;AT1G	2.49E-02	0.96	0.90	1.13	1.14	1.18	1.26	1.07	[AT1G25210, UDP-3-O-acyl N-acetylglycosamine deacetylase family protein contains Pfam domain PF03331: UDP-3-O-acyl N-acetylglycosamine deacetylase];[AT1G24880, UDP-3-O-acyl N-acetylglucosamine deacetylase family protein / F-box protein-related contains weak hit to TIGRFAM TIGR01640 : F-box protein interaction domain and weak hit to Pfam PF00646: F-box domain; similar to ESTs dbj AV4426[illegible]
AT1G56380	2.44E-02	0.93	0.91	1.11	1.04	1.18	1.14	0.96	mitochondrial transcription termination factor family protein / mTERF family protein weak similarity to mtDBP protein [Paracentrotus lividus] GI:4584695; contains Pfam profile PF02536: mTERF
AT1G71410	2.00E-02	0.96	0.79	1.13	1.31	1.18	1.67	1.41	protein kinase family protein contains protein kinase domain, Pfam:PF00069
AT1G17230	3.28E-02	0.90	0.91	1.06	1.39	1.18	1.52	1.28	leucine-rich repeat family protein / protein kinase family protein contains protein kinase domain, Pfam:PF00069; contains leucine-rich repeats, Pfam:PF00560
AT1G73560	1.79E-02	0.90	0.95	1.07	1.17	1.18	1.24	1.04	protease inhibitor/seed storage/lipid transfer protein (LTP) family protein similar to GI:2627141 from [Picea abies] (Plant Mol. Biol. 42 (3), 461-478 (2000)); contains Pfam protease inhibitor/seed storage/LTP family domain PF00234
AT3G27240	2.62E-02	1.01	0.90	1.19	1.06	1.18	1.17	0.99	cytochrome c1, putative cytochrome c1, heme protein, mitochondrial precursor (Clone PC13III) [Solanum tuberosum] SWISS-PROT:P25076
AT5G08450	4.63E-02	0.95	0.91	1.12	1.08	1.18	1.19	1.00	expressed protein KED, Nicotiana tabacum, EMBL:AB009883
AT1G07460	3.24E-02	0.95	0.92	1.13	1.17	1.18	1.27	1.07	legume lectin family protein contains Legume lectins beta-chain signature, PROSITE:PS00307
AT5G02350;AT2G	2.16E-02	0.93	0.94	1.10	1.03	1.18	1.09	0.92	[AT5G02350, DC1 domain-containing protein contains Pfam profile PF03107: DC1 domain];[AT2G13900, DC1 domain-containing protein contains Pfam protein PF03107 DC1 domain]
AT4G16230	6.77E-03	0.95	0.93	1.12	1.07	1.18	1.16	0.98	GDSL-motif lipase/hydrolase family protein similar to SP|P40602 Anther-specific proline-rich protein APG precursor [Arabidopsis thaliana]; contains Pfam profile PF00657: GDSL-like Lipase/Acylhydrolase
AT4G12130	1.25E-02	0.92	0.94	1.08	1.06	1.18	1.12	0.95	glycine cleavage T family protein / aminomethyl transferase family protein contains Pfam profile: PF01571 glycine cleavage T-protein (aminomethyl transferase)
AT4G14820	2.64E-02	0.90	0.93	1.06	1.11	1.18	1.19	1.01	pentatricopeptide (PPR) repeat-containing protein contains Pfam profile PF01535: PPR repeat
AT5G11700	4.88E-02	0.96	0.91	1.13	1.30	1.18	1.44	1.22	glycine-rich protein predicted protein, Arabidopsis thaliana
AT5G10820	4.47E-04	0.95	0.84	1.12	1.08	1.18	1.28	1.09	integral membrane transporter family protein contains 11 transmembrane domains; similar to folate/methotrexate transporter FT1 (GI:5813863) [Leishmania donovani]; lignostilbene-alpha,beta-dioxygenase gene, Synechococcus PCC7942, EMBL:AF055873
AT3G13784	1.38E-02	0.94	0.88	1.12	1.06	1.18	1.20	1.01	beta-fructosidase, putative / beta-fructofuranosidase, putative / cell wall invertase, putative similar to beta-fructofuranosidase GI:402740 from [Arabidopsis thaliana]
AT3G42880	9.94E-03	0.94	0.91	1.11	1.09	1.18	1.20	1.02	leucine-rich repeat transmembrane protein kinase, putative receptor-like protein kinase PRK1, Lycopersicon esculentum, PIR:T07865
AT5G02460	4.19E-04	0.93	0.89	1.10	1.22	1.18	1.36	1.15	Dof-type zinc finger domain-containing protein zinc finger protein OBP3, Arabidopsis thaliana, EMBL:AF155818
AT2G02020	4.27E-02	0.91	0.95	1.07	1.32	1.18	1.39	1.17	proton-dependent oligopeptide transport (POT) family protein contains Pfam profile: PF00854 POT family
AT4G19300;AT5G	4.12E-02	0.95	0.89	1.12	1.13	1.18	1.26	1.07	[AT4G19300, hypothetical protein includes At2g10370, At5g36050, At1g35090, At1g44860, At4g19300, At1g42400, At3g43040, At3g42500];[AT5G36050, hypothetical protein includes At2g10370, At5g36050, At1g35090, At1g44860, At4g19300, At1g42400, At3g43040, At3g42500]
AT2G43790	2.59E-02	0.68	1.18	0.80	1.40	1.18	1.19	1.01	mitogen-activated protein kinase, putative / MAPK, putative (MPK6) identical to mitogen-activated protein kinase homolog 6 (AtMPK6)[Arabidopsis thaliana[SWISS-PROT:Q39026; PMID:12119167
AT1G34770	2.09E-02	0.89	0.99	1.05	1.19	1.18	1.20	1.02	MAGE-8 antigen-related contains weak similarity to Swiss-Prot:P43361 melanoma-associated antigen 8 (MAGE-8 antigen) [Homo sapiens]
AT4G04900	1.20E-02	0.95	0.93	1.12	1.04	1.18	1.12	0.94	p21-rho-binding domain-containing protein contains Pfam PF00786: P21-Rho-binding domain
AT1G15640	4.28E-03	0.95	0.89	1.12	1.12	1.18	1.26	1.06	hypothetical protein
AT1G31670	8.59E-03	0.92	0.85	1.08	1.05	1.18	1.24	1.05	copper amine oxidase, putative similar to amine oxidase [copper-containing] precursor [Pisum sativum] SWISS-PROT:Q43077
AT5G07830	4.67E-02	0.92	0.93	1.09	1.22	1.18	1.30	1.10	glycosyl hydrolase family 79 N-terminal domain-containing protein similar to beta-glucuronidase GI:8918740 from [Scutellaria baicalensis]
AT2G41590	3.79E-02	0.94	0.96	1.11	1.07	1.18	1.12	0.95	expressed protein similar to zinc finger protein [Arabidopsis thaliana] GI:976277
AT4G24400	4.68E-02	0.88	0.99	1.04	1.26	1.18	1.26	1.07	CBL-interacting protein kinase 8 (CIPK8) identical to CBL-interacting protein kinase 8 [Arabidopsis thaliana] GP|13249115|gb|AAK16683; contains Pfam profiles PF00069: Protein kinase domain and PF03822: NAF domain
AT3G54920	1.86E-02	1.01	0.76	1.20	1.09	1.18	1.44	1.22	pectate lyase, putative / powdery mildew susceptibility protein (PMR6) identical to powdery mildew susceptibility protein [Arabidopsis thaliana] GI:22506901; similar to pectate lyase 2 GP:6606534 from [Musa acuminata]
AT1G16560	1.76E-02	0.92	0.90	1.08	1.13	1.18	1.26	1.07	Per1-like family protein contains Pfam profile PF04080: Per1-like
AT2G38290	1.83E-02	0.62	1.46	0.73	2.18	1.18	1.50	1.27	ammonium transporter 2 (AMT2) identical to SP|Q9M6N7 Ammonium transporter 2 (AtAMT2) [Arabidopsis thaliana]
AT1G43910	4.52E-02	0.90	0.96	1.06	1.17	1.18	1.22	1.03	AAA-type ATPase family protein contains Pfam profile: ATPase family PF00004
AT1G05180	4.37E-02	0.96	0.97	1.13	1.22	1.18	1.26	1.07	auxin-resistance protein AXR1 (AXR1) identical to SP|P42744 Auxin-resistance protein AXR1 [Arabidopsis thaliana]; contains Pfam profile PF00899: ThiF family; identical to cDNA ubiquitin activating enzyme E1-related protein (AXR1) GI:304103
AT1G61900	1.89E-02	1.04	0.84	1.22	1.01	1.18	1.21	1.03	expressed protein contains similarity to glutamic acid/alanine-rich protein GI:6707830 from [Trypanosoma congolense]

AT1G79730	9.18E-03	0.93	0.94	1.10	1.22	1.18	1.31	1.11	hydroxyproline-rich glycoprotein family protein contains proline-rich extensin domains, INTERPRO:IPR002965
AT3G61440	1.52E-03	0.94	0.92	1.11	1.17	1.18	1.27	1.08	cysteine synthase, putative / O-acetylserine (thiol)-lyase, putative / O-acetylserine sulfhydrylase, putative identical to cysteine synthase (EC 4.2.99.8) [Arabidopsis thaliana] GI:5824334; contains Pfam profile PF00291: Pyridoxal-phosphate dependent enzyme
AT4G21890	3.36E-02	0.93	0.93	1.10	1.05	1.18	1.13	0.96	expressed protein
AT2G19900	3.36E-02	0.93	0.94	1.09	1.10	1.18	1.18	1.00	malate oxidoreductase, putative similar to NADP-dependent malic enzyme (EC 1.1.1.40) (NADP-ME) (SP:P51615) [Vitis vinifera]
AT2G31460	7.76E-03	0.95	0.87	1.12	1.17	1.18	1.34	1.14	hypothetical protein contains Pfam profile PF03754: Domain of unknown function (DUF313)
AT3G49380	1.96E-02	0.92	0.91	1.08	1.09	1.18	1.21	1.02	calmodulin-binding family protein
AT1G56160	2.77E-02	0.96	0.98	1.13	1.13	1.18	1.15	0.98	myb family transcription factor (MYB72) contains Pfam profile: PF00249 myb-like DNA-binding domain; identical to cDNA putative transcription factor (MYB72) mRNA, partial cds GI:3941501
AT2G29820	3.35E-03	0.89	0.92	1.05	1.14	1.18	1.24	1.06	kelch repeat-containing F-box family protein similar to SKP1 interacting partner 6 [Arabidopsis thaliana] GI:10716957; contains Pfam profiles PF01344: Kelch motif, PF00646: F-box domain
AT5G47460	5.42E-03	0.92	0.92	1.08	1.17	1.18	1.28	1.08	pentatricopeptide (PPR) repeat-containing protein contains Pfam profile PF01535: PPR repeat
AT1G05160	3.12E-02	0.91	0.91	1.07	1.06	1.18	1.17	1.00	ent-kaurenoic acid hydroxylase (KAO1) / cytochrome P450 88A3, putative (CYP88A3) identical to Cytochrome P450 88A3 (SP:O23051) [Arabidopsis thaliana]; nearly identical to ent-kaurenoic acid hydroxylase (KAO1) GI:13021852 from [Arabidopsis thaliana]
AT1G77840	3.08E-02	0.96	0.86	1.13	1.19	1.18	1.38	1.17	eukaryotic translation initiation factor 5, putative / eIF-5, putative similar to SP|P55876 Eukaryotic translation initiation factor 5 (eIF-5) {Zea mays}; contains Pfam profiles PF02020: eIF4-gamma/eIF5/eIF2-epsilon, PF01873: Domain found in IF2B/IF5
AT1G63020	4.24E-02	0.92	0.97	1.08	1.34	1.18	1.39	1.18	DNA-directed RNA polymerase alpha subunit family protein low similarity to RNA polymerase IIA largest subunit [Trypanosoma brucei] GI:162215; contains InterPro accession IPR000722: RNA polymerase, alpha subunit
AT3G53170	1.70E-02	0.91	0.81	1.08	1.10	1.18	1.36	1.15	pentatricopeptide (PPR) repeat-containing protein low similarity to fertility restorer [Petunia x hybrida] GI:22128587; contains Pfam profile PF01535: PPR repeat
AT1G24000	3.40E-02	0.94	0.92	1.11	1.09	1.18	1.18	1.01	Bet v I allergen family protein contains Pfam profile PF00407: Pathogenesis-related protein Bet v I family
AT5G45970	2.65E-02	1.00	0.93	1.18	1.06	1.18	1.14	0.97	Rac-like GTP-binding protein (ARAC2) identical to RAC-like GTP binding protein ARAC2 SP:Q38903
AT2G47760	9.19E-03	0.66	1.27	0.77	1.61	1.18	1.27	1.08	ALG3 family protein contains Pfam profile: PF05208 ALG3 protein
AT1G69220	1.87E-02	0.97	0.77	1.14	1.16	1.18	1.50	1.27	serine/threonine protein kinase, putative identical to serine/threonine kinase [Arabidopsis thaliana] gi|2352084|gb|AAB68776
AT2G03360	3.30E-02	0.95	0.89	1.12	1.04	1.18	1.17	1.00	hypothetical protein contains Pfam domain, PF04577: Protein of unknown function (DUF563)
AT5G20310	2.31E-02	0.92	0.90	1.08	1.23	1.18	1.37	1.17	hypothetical protein
AT5G45480	1.45E-02	0.95	0.90	1.11	1.66	1.18	1.84	1.56	expressed protein contains Pfam domain, PF04578: Protein of unknown function, DUF594
AT4G30150	1.12E-02	0.94	0.88	1.11	1.21	1.18	1.37	1.17	expressed protein
AT1G46912	3.03E-02	0.93	0.91	1.09	1.06	1.18	1.16	0.99	F-box family protein-related contains weak hit to TIGRFAM TIGR01640 : F-box protein interaction domain; similar to hypothetical protein GI:4589954 from [Arabidopsis thaliana] contains weak hit to TIGRFAM TIGR01640 : F-box protein interaction domain;
AT4G19410	9.72E-03	0.93	0.87	1.09	1.14	1.18	1.31	1.11	pectinacetylesterase, putative similar to pectinacetylesterase precursor GI:1431629 from [Vigna radiata]
AT4G21590	3.25E-02	0.95	0.90	1.11	1.06	1.18	1.17	1.00	bifunctional nuclease, putative similar to bifunctional nuclease [Zinnia elegans] gi|4099833|gb|AAD00694
AT2G29740	4.78E-02	0.96	0.91	1.13	1.10	1.18	1.20	1.02	UDP-glucoronosyl/UDP-glucosyl transferase family protein contains Pfam profile: PF00201 UDP-glucoronosyl and UDP-glucosyl transferase
AT1G61000	3.22E-02	0.95	0.94	1.12	1.21	1.17	1.28	1.09	Nuf2 family protein contains Pfam PF03800: Nuf2 family domain; similar to Myosin-like protein NUF2 (Nuclear filament-containing protein 2) (Nuclear division protein nuf2) (Swiss-Prot:Q10173) [Schizosaccharomyces pombe]
AT5G58350	3.67E-02	0.84	1.06	0.98	1.73	1.17	1.63	1.39	protein kinase family protein contains protein kinase domain, Pfam:PF00069
AT3G56700	2.91E-02	1.00	0.91	1.18	1.06	1.17	1.16	0.99	male sterility protein, putative similar to SP|Q08891 Male sterility protein 2 {Arabidopsis thaliana}; contains Pfam profile PF03015: Male sterility protein
AT4G27880	3.90E-02	1.00	0.88	1.18	0.99	1.17	1.13	0.96	seven in absentia (SINA) family protein similar to siah-1A protein [Mus musculus] GI:297035; contains Pfam profile PF03145: Seven in absentia protein family
AT3G17410	1.58E-02	0.91	0.93	1.06	1.18	1.17	1.27	1.08	serine/threonine protein kinase, putative similar to Pto kinase interactor 1 GB:AAC61805 from [Lycopersicon esculentum]
AT3G43750	2.68E-02	0.94	0.97	1.10	1.08	1.17	1.12	0.96	zinc finger (C3HC4-type RING finger) family protein contains a zinc finger, C3HC4 type (RING finger), signature, PROSITE:PS00518
AT1G67030	4.88E-02	0.94	0.91	1.10	1.00	1.17	1.10	0.94	zinc finger (C2H2 type) family protein (ZFP6) identical to zinc finger protein, ZFP6 gi|790683|gb|AAA87302; contains Pfam domain, PF00096: Zinc finger, C2H2 type
AT4G25440	6.39E-03	0.97	0.90	1.14	1.13	1.17	1.25	1.07	temperature sensing protein-related contains weak similarity to D123 (GI:1236114) [Rattus norvegicus]
AT3G43170	4.15E-02	0.88	0.91	1.03	1.09	1.17	1.19	1.01	hypothetical protein contains Pfam profile PF03478: Protein of unknown function (DUF295)
AT3G03230	1.95E-03	0.99	0.89	1.17	1.01	1.17	1.13	0.96	esterase/lipase/thioesterase family protein contains Interpro entry IPR000379
AT1G54320	9.16E-03	0.97	0.90	1.14	1.05	1.17	1.17	1.00	LEM3 (ligand-effect modulator 3) family protein / CDC50 family protein Similar to GI:11994416; GI:4966357; GI:4835763; GI:9757735 from [Arabidopsis thaliana]
AT5G28420	3.59E-02	0.96	0.93	1.13	1.05	1.17	1.12	0.96	hypothetical protein predicted proteins - Arabidopsis thaliana
AT2G23730	9.70E-03	0.93	0.92	1.09	1.10	1.17	1.19	1.01	expressed protein
AT3G19350	2.83E-02	1.01	0.92	1.18	1.01	1.17	1.10	0.94	polyadenylate-binding protein-related / PABP-related similar to poly(A)-binding protein [Cucumis sativus] GI:7528270; contains Pfam profile PF00658: Poly-adenylate binding protein, unique domain
AT2G44350	2.63E-02	0.74	1.20	0.86	1.62	1.17	1.35	1.15	citrate synthase, mitochondrial, putative strong similarity to SP|P20115 Citrate synthase, mitochondrial precursor {Arabidopsis thaliana}; contains Pfam profile PF00285: Citrate synthase
AT1G79180	4.89E-02	0.93	0.91	1.09	1.07	1.17	1.18	1.00	myb family transcription factor (MYB63) similar to myb-related protein GI:1370139 from [Lycopersicon esculentum]
AT1G50230	3.46E-02	0.93	0.97	1.09	1.14	1.17	1.17	1.00	protein kinase family protein contains protein kinase domain, Pfam:PF00069
AT1G30000	2.08E-02	0.96	0.92	1.12	1.21	1.17	1.32	1.13	glycoside hydrolase family 47 protein similar to GI:5579331 from [Homo sapiens]; contains Pfam profile PF01532: Glycosyl hydrolase family 47
AT4G16900	4.11E-02	0.93	0.88	1.09	1.18	1.17	1.33	1.14	hydroxyproline-rich glycoprotein family protein
AT2G23550	1.14E-02	0.98	0.97	1.14	1.11	1.17	1.14	0.98	hydrolase, alpha/beta fold family protein similar to ethylene-induced esterase [Citrus sinensis] GI:14279437, polyneuridine aldehyde esterase [Rauvolfia serpentina] GI:6651393; contains Pfam profile PF00561: hydrolase, alpha/beta fold family
AT3G53460	2.35E-02	0.96	0.89	1.12	1.12	1.17	1.26	1.07	29 kDa ribonucleoprotein, chloroplast / RNA-binding protein cp 29 nearly identical to SP|Q43349 29 kDa ribonucleoprotein, chloroplast precursor (RNA-binding protein cp29) {Arabidopsis thaliana}
AT5G04560	3.80E-02	0.92	0.85	1.08	1.07	1.17	1.25	1.07	DEMETER protein (DME) identical to DEMETER protein [Arabidopsis thaliana] GI:21743571; contains Pfam profile PF00730: HhH-GPD superfamily base excision DNA repair protein
AT2G04070	3.77E-02	0.99	0.92	1.16	1.08	1.17	1.18	1.01	MATE efflux family protein similar to ripening regulated protein DDTFR18 [Lycopersicon esculentum] GI:12231296; contains Pfam profile PF01554: Uncharacterized membrane protein family
AT4G03030	4.52E-02	0.99	0.84	1.16	1.09	1.17	1.29	1.10	kelch repeat-containing F-box family protein contains Pfam profiles PF01344: Kelch motif, PF00646: F-box domain

AT1G78130	2.32E-02	0.96	0.92	1.08	1.13	1.13	1.23	1.09	transporter-related low similarity to spinster type III [Drosophila melanogaster] GI:12003974; contains Pfam profile: PF00083 major facilitator superfamily protein
AT3G46220	2.80E-02	0.96	0.85	1.09	1.26	1.13	1.49	1.32	expressed protein
AT4G37950	4.13E-02	0.99	0.86	1.11	1.07	1.13	1.25	1.11	expressed protein
AT3G59950;AT3G	1.89E-02	0.94	0.97	1.06	1.11	1.13	1.15	1.02	[AT3G59950, autophagy 4b (APG4b) identical to autophagy 4b [Arabidopsis thaliana] gi|19912145|dbj|BAB88384|];[AT3G59940, kelch repeat-containing F-box family protein contains Pfam profiles PF01344: Kelch motif, PF00646: F-box domain]
AT4G39430	2.60E-02	0.97	0.94	1.10	1.03	1.13	1.09	0.96	expressed protein ; expression supported by MPSS
AT3G17880	4.61E-02	0.99	0.94	1.12	1.08	1.13	1.15	1.02	tetratricoredoxin (TDX) identical to tetratricoredoxin [Arabidopsis thaliana] GI:18041544; similar to SP|Q42443 Thioredoxin H-type (TRX-H) (Phloem sap 13 kDa protein-1) (Oryza sativa); contains Pfam profile: PF00085 Thioredoxin
AT3G21480	2.09E-02	0.91	0.90	1.03	1.13	1.13	1.26	1.11	transcription activation domain-interacting protein-related contains weak similarity to Pax transcription activation domain interacting protein PTIP (GI:4336734) [Mus musculus]
AT1G09795	1.54E-03	0.96	0.91	1.08	1.04	1.13	1.14	1.01	ATP phosphoribosyl transferase 2 (ATP-PRT2) identical to ATP phosphoribosyl transferase (AtATP-PRT2) [Arabidopsis thaliana] GI:6683619; supporting cDNA gi|6683618|dbj|AB025250.1|
AT5G64090	2.87E-02	0.98	0.89	1.11	1.09	1.13	1.23	1.09	expressed protein
AT1G14220	2.52E-02	0.99	0.92	1.12	1.03	1.13	1.13	1.00	ribonuclease T2 family protein contains similarity to S-like ribonuclease PD1 GI:9957752 from [Prunus dulcis]; contains ribonuclease T2 family histidine protein motif
AT4G10780	3.20E-02	0.91	0.90	1.03	1.07	1.13	1.18	1.05	disease resistance protein (CC-NBS-LRR class), putative domain signature CC-NBS-LRR exists, suggestive of a disease resistance protein.
AT3G01560	1.21E-02	0.99	0.85	1.12	1.09	1.13	1.28	1.14	proline-rich family protein contains proline rich extensin domains, INTERPRO:IPR002965
AT5G38440	4.71E-02	0.97	0.90	1.09	1.03	1.13	1.14	1.01	self-incompatibility protein-related low similarity to self-incompatibility [Papaver nudicaule] GI:3097262, S3 self-incompatibility protein [Papaver rhoeas] GI:1107841
AT5G18880	2.90E-02	0.96	0.93	1.08	1.17	1.13	1.26	1.11	expressed protein ; expression supported by MPSS
AT5G21040	4.48E-02	0.97	0.89	1.09	1.14	1.13	1.28	1.14	F-box family protein / WD-40 repeat family protein contains G-protein beta WD-40 repeats
AT1G61420	2.70E-02	0.93	0.96	1.05	1.19	1.13	1.24	1.10	S-locus lectin protein kinase family protein contains Pfam domains, PF00954: S-locus glycoprotein family, PF00069: Protein kinase domain, and PF01453: Lectin (probable mannose binding)
AT1G01920	4.13E-02	0.92	0.94	1.04	1.09	1.13	1.17	1.04	SET domain-containing protein low similarity to SP|Q43088 Ribulose-1,5 bisphosphate carboxylase/oxygenase large subunit N- methyltransferase, chloroplast precursor (EC 2.1.1.127) (Pisum sativum]; contains Pfam profile PF00856: SET domain
AT3G46880	4.12E-02	0.97	0.91	1.09	1.04	1.13	1.14	1.01	expressed protein ; expression supported by MPSS
AT2G44560	4.10E-02	0.98	0.95	1.11	0.98	1.13	1.03	0.91	glycosyl hydrolase family 9 protein
AT5G50230	1.44E-02	0.96	0.90	1.08	1.12	1.13	1.24	1.10	transducin family protein / WD-40 repeat family protein contains 7 WD-40 repeats (PF00400); similar to TIPD PROTEIN (SP:O15736)[Dictyostelium discoideum]
AT4G18080	4.10E-02	0.96	0.88	1.08	1.03	1.13	1.16	1.03	hypothetical protein
AT5G24380;AT5G	1.88E-02	0.98	0.93	1.10	1.12	1.13	1.20	1.07	[AT5G24380, transporter, putative similar to iron-phytosiderophore transporter protein yellow stripe 1 [Zea mays] GI:10770865; contains Pfam profile PF03169: OPT oligopeptide transporter protein];[AT5G24390, RabGAP/TBC domain-containing protein similar to GTPase activating protein [Yarrowia lipolytica] GI:2370595; contains Pfam profile PF00566: T[illegible]
AT3G55440	3.28E-02	0.95	0.92	1.08	1.01	1.13	1.09	0.97	triosephosphate isomerase, cytosolic, putative strong similarity to triosephosphate isomerase, cytosolic from Petunia hybrida [SP|P48495], from Coptis japonica [SP|P21820]
AT1G20180	3.41E-02	0.95	0.91	1.07	1.05	1.13	1.16	1.03	hypothetical protein similar to At14a (GI:11994571 and GI:11994573) [Arabidopsis thaliana]
AT1G80770	4.70E-02	0.90	0.95	1.01	1.11	1.13	1.18	1.04	expressed protein
AT3G13160	1.63E-02	0.98	0.89	1.10	1.04	1.13	1.17	1.04	pentatricopeptide (PPR) repeat-containing protein contains Pfam profile PF01535: PPR repeat
AT1G77590	4.81E-02	0.98	0.92	1.11	1.11	1.13	1.20	1.07	long-chain-fatty-acid--CoA ligase family protein / long-chain acyl-CoA synthetase family protein (LACS9) similar to LACS 3 [SP|O95573] from Homo Sapiens, LACS 3 [SP|Q63151] from Rattus norvegicus; contains Pfam HMM hit: AMP-binding enzymes PF00501
AT1G80360	2.90E-02	0.95	0.83	1.07	1.10	1.13	1.32	1.17	aminotransferase class I and II family protein low similarity to GI:14278621 Aromatic Aminotransferase from Pyrococcus horikoshii
AT5G58160	1.23E-02	0.96	0.93	1.08	1.07	1.13	1.16	1.03	formin homology 2 domain-containing protein / FH2 domain-containing protein low similarity to SP|Q05858 Formin (Limb deformity protein) (Gallus gallus); contains Pfam profile PF02181: Formin Homology 2(FH2) Domain
AT2G29940	1.02E-02	0.98	0.96	1.10	1.46	1.13	1.52	1.35	ABC transporter family protein similar to ABC1 protein GI:14331118 from [Nicotiana plumbaginifolia]
AT2G28160	1.74E-02	0.98	0.94	1.10	1.04	1.13	1.12	0.99	basic helix-loop-helix (bHLH) family protein
AT2G22530	1.38E-02	0.90	0.97	1.01	1.31	1.13	1.35	1.20	phosphatidylinositolglycan-related similar to Pig-o [Mus musculus] GI:8099973
AT5G52170	4.43E-02	1.03	0.86	1.15	1.09	1.13	1.27	1.12	homeobox-leucine zipper family protein / lipid-binding START domain-containing protein similar to Anthocyaninless2 (ANL2) (GP:5702094) [Arabidopsis thaliana[; contains Pfam PF00046: Homeobox domain and Pfam PF01852: START domain
AT5G66340	4.27E-02	0.97	0.87	1.09	1.05	1.13	1.20	1.07	hypothetical protein
AT5G47635	1.77E-02	0.94	0.96	1.06	1.15	1.13	1.20	1.06	expressed protein
AT2G47970	3.76E-02	0.99	0.90	1.12	1.12	1.12	1.24	1.11	NPL4 family protein contains Pfam domain, PF05021: NPL4 family
AT5G15270	3.65E-02	0.93	0.91	1.05	1.12	1.12	1.24	1.10	KH domain-containing protein various predicted proteins, Arabidopsis thaliana and Oryza sativa
AT1G68110	2.04E-02	1.07	0.65	1.20	0.88	1.12	1.34	1.19	epsin N-terminal homology (ENTH) domain-containing protein / clathrin assembly protein-related similar to clathrin assembly protein AP180 (GI:6492344) [Xenopus laevis]; contains Pfam profile: PF01417 ENTH domain, suggesting involvement in endocytosis or cytoskeletal machinery
AT4G27850	9.91E-03	0.91	0.92	1.03	1.11	1.12	1.20	1.07	proline-rich family protein contains proline-rich extensin domains, INTERPRO:IPR002965
AT1G52470	2.64E-02	0.97	0.93	1.09	1.10	1.12	1.17	1.04	hypothetical protein
AT1G53645	4.31E-02	0.93	0.93	1.05	1.30	1.12	1.41	1.25	hydroxyproline-rich glycoprotein family protein
AT1G67500	4.33E-02	0.88	0.97	0.99	1.19	1.12	1.24	1.10	DNA polymerase family B protein similar to SP|Q61493 DNA polymerase zeta catalytic subunit (EC 2.7.7.7) (Mus musculus); contains Pfam profile PF00136: DNA polymerase family B
AT5G23000	4.25E-02	0.91	1.00	1.02	1.14	1.12	1.14	1.02	myb family transcription factor (MYB37) contains PFAM profile: myb DNA binding domain PF00249;
AT2G22320	1.32E-02	0.96	0.95	1.08	1.02	1.12	1.08	0.96	hypothetical protein
AT3G62280	2.34E-02	0.96	0.90	1.07	1.20	1.12	1.33	1.18	GDSL-motif lipase/hydrolase family protein similar to Enod8.1 [Medicago truncatula] GI:18390045; contains InterPro Entry IPR001087 Lipolytic enzyme, G-D-S-L family
AT5G61460	1.45E-02	0.90	0.96	1.02	1.16	1.12	1.22	1.08	structural maintenance of chromosomes (SMC) family protein very strong similarity to SMC-like protein (MIM) [Arabidopsis thaliana] GI:5880614; contains Pfam profile PF02463: RecF/RecN/SMC N terminal domain
[illegible]	[illegible]	[illegible]	[illegible]	[illegible]	[illegible]	[illegible]	[illegible]	[illegible]	[illegible]

AT5G04050	3.30E-02	1.00	0.88	1.04	1.28	1.03	1.44	1.40	maturase-related contains similarity to maturase proteins from several species
AT3G49370	2.91E-02	0.96	0.93	0.98	1.24	1.03	1.33	1.29	calcium-dependent protein kinase, putative / CDPK, putative similar to calcium/calmodulin-dependent protein kinase CaMK1 [Nicotiana tabacum] gi|16904222|gb|AAL30818
AT4G24500	4.88E-02	0.95	0.97	0.98	1.18	1.03	1.21	1.18	hydroxyproline-rich glycoprotein family protein
AT5G02370	9.72E-03	1.07	0.79	1.10	0.95	1.02	1.21	1.18	kinesin motor protein-related kinesin, Xenopus laevis, EMBL:XLA249840
AT5G60870	4.71E-02	0.99	0.95	1.01	1.22	1.02	1.29	1.26	regulator of chromosome condensation (RCC1) family protein weak similarity to UVB-resistance protein UVR8 [Arabidopsis thaliana] GI:5478530; contains Pfam profile PF00415: Regulator of chromosome condensation (RCC1)
AT1G53790	2.74E-02	1.04	0.84	1.06	0.94	1.02	1.11	1.09	F-box family protein contains Pfam PF00646: F-box domain; contains TIGRFAM TIGR01640 : F-box protein interaction domain
AT3G43480;AT3G	2.79E-02	1.00	0.85	1.01	1.10	1.02	1.29	1.27	[AT3G43480, hypothetical protein hypothetical protein At2g15180 - Arabidopsis thaliana, EMBL:AC006298];[AT3G43470, hypothetical protein];[AT2G15185, zinc knuckle (CCHC-type) protein-related supported by tandem duplication of zinc knuckle (CCHC type) familyprotein (GI:4585917) (TIGR_Ath1:At2g15180) [Arabidopsis thaliana]]
AT1G07620	3.26E-02	1.01	0.81	1.02	1.07	1.01	1.33	1.31	GTP1/OBG family protein similar to SP|P20964 Spo0B-associated GTP-binding protein (Bacillus subtilis); contains Pfam profile PF01018: GTP1/OBG family
AT5G27660	8.74E-03	1.00	0.93	1.01	1.13	1.01	1.21	1.20	DegP protease, putative similar to Serine protease HTRA2, mitochondrial precursor (High temperature requirement protein A2) (HtrA2) (Omi stress-regulated endoprotease) (Serine proteinase OMI) (SP:O43464) (Homo sapiens)
AT1G79490	3.01E-02	1.01	0.83	1.02	1.34	1.01	1.61	1.60	pentatricopeptide (PPR) repeat-containing protein low similarity to fertility restorer [Petunia x hybrida] GI:22128587; contains Pfam profile PF01535: PPR repeat
AT3G13140	4.76E-02	1.00	0.89	1.01	1.16	1.00	1.30	1.30	hydroxyproline-rich glycoprotein family protein
AT3G42340	3.01E-02	1.00	0.95	0.99	1.18	0.99	1.24	1.25	hypothetical protein various predicted Helicases, Arabidopsis thaliana
AT2G33490	3.72E-02	0.96	1.01	0.95	1.28	0.99	1.27	1.28	hydroxyproline-rich glycoprotein family protein Common family member:At3g26910 [Arabidopsis thaliana]
AT5G56370	3.04E-02	1.01	0.88	0.98	1.31	0.97	1.49	1.53	F-box family protein contains F-box domain Pfam:PF00646
AT1G30500	4.27E-02	1.09	1.04	1.05	0.61	0.97	0.59	0.61	CCAAT-binding transcription factor (CBF-B/NF-YA) family protein contains Pfam profile: PF02045 CCAAT-binding transcription factor (CBF-B/NF-YA)
AT3G44735	4.63E-02	1.09	1.25	1.05	0.58	0.96	0.46	0.48	phytosulfokines-related contains similarities to phytosulfokines from [Arabidopsis thaliana]
AT5G14970	3.60E-02	1.11	0.96	1.06	0.73	0.96	0.76	0.79	expressed protein
AT2G40230	4.93E-02	0.87	1.38	0.83	1.05	0.96	0.76	0.80	transferase family protein similar to taxadienol acetyl transferase from Taxus cuspidata [gi:6978038]; contains Pfam transferase family domain PF002458
AT5G62460	1.84E-02	1.05	1.01	1.00	0.77	0.95	0.76	0.80	zinc finger (C3HC4-type RING finger) family protein contains Pfam domain, PF00097: Zinc finger, C3HC4 type (RING finger)
AT2G20750	1.92E-02	1.06	1.17	1.00	0.86	0.94	0.74	0.78	beta-expansin, putative (EXPB1) identical to beta-expansin [Arabidopsis thaliana] gi|2224913|gb|AAB61709; similar to SP:O04701 major pollen allergen, Bermuda grass [Cynodon dactylon]; beta-expansin gene family, PMID:11641069
AT5G15770	4.33E-02	1.09	1.02	1.02	0.70	0.94	0.68	0.73	GCN5-related N-acetyltransferase (GNAT) family protein similar to SP|O93806 Glucosamine-phosphate N-acetyltransferase (EC 2.3.1.4) (Phosphoglucosamine transacetylase) (Phosphoglucosamine acetylase) (Candida albicans); contains Pfam profile PF00583: acetyltransferase, GNAT family
AT5G13120	3.35E-02	1.15	0.97	1.08	0.80	0.94	0.83	0.88	peptidyl-prolyl cis-trans isomerase cyclophilin-type family protein contains Pfam domain, PF00160: peptidyl-prolyl cis-trans isomerase, cyclophilin-type
AT5G51260	2.48E-02	1.06	1.02	0.99	0.75	0.94	0.74	0.79	acid phosphatase, putative similar to acid phosphatase-1(1); Apase-1(1) [Lycopersicon esculentum] GI:7705154, acid phosphatase [Glycine max] GI:3341443; contains Pfam profile PF03767: HAD superfamily (subfamily IIIB) phosphatase
AT5G08415	1.21E-02	1.09	1.01	1.01	0.77	0.93	0.76	0.82	lipoic acid synthase family protein similar to lipoic acid synthase from Arabidopsis thaliana [gi:3928758], from Mus musculus [gi:14669826] Pfam profile PF04055: radical SAM domain protein
AT3G06950	4.27E-02	1.06	1.06	0.99	0.85	0.93	0.81	0.87	tRNA pseudouridine synthase family protein similar to SP|P07649 tRNA pseudouridine synthase A (EC 4.2.1.70) (Uracil hydrolyase) (Escherichia coli); contains Pfam profile PF01416: tRNA pseudouridine synthase
AT5G48590	3.85E-02	1.05	1.01	0.97	0.87	0.93	0.86	0.93	expressed protein
AT2G46110	2.49E-02	0.89	1.76	0.82	1.22	0.92	0.69	0.75	ketopantoate hydroxymethyltransferase family protein similar to SP|Q9Y7B6 3-methyl-2-oxobutanoate hydroxymethyltransferase (EC 2.1.2.11) (Ketopantoate hydroxymethyltransferase) (Emericella nidulans); contains Pfam profile PF02548: Ketopantoate hydroxymethyltransferase
AT3G01500	3.03E-02	1.05	1.04	0.97	0.92	0.92	0.89	0.97	carbonic anhydrase 1, chloroplast / carbonate dehydratase 1 (CA1) nearly identical to SP|P27140 Carbonic anhydrase, chloroplast precursor (EC 4.2.1.1) (Carbonate dehydratase) (Arabidopsis thaliana)
AT5G45940	4.61E-02	1.04	1.14	0.96	0.81	0.92	0.72	0.78	MutT/nudix family protein contains Pfam profile PF00293: NUDIX domain
AT3G17170	4.76E-02	1.11	1.00	1.02	0.69	0.92	0.70	0.76	ribosomal protein S6 family protein (RFC3) annotation temporarily based on supporting cDNA gi|15620809|dbj|AB057424.1|; contains TIGRfam TIGR00166 and Pfam PF01250 profiles ribosomal protein S6.
AT1G66230	3.66E-02	1.00	1.24	0.92	0.85	0.92	0.69	0.75	myb family transcription factor (MYB20) similar to myb-related transcription factor GI:1430846 from [Lycopersicon esculentum]; contains PFAM profile: Myb DNA binding domain PF00249
AT3G55040	1.20E-02	1.10	1.04	1.01	0.73	0.92	0.71	0.77	In2-1 protein, putative similar to In2-1 protein, Zea mays, P49248
AT2G26930	1.83E-02	1.13	1.04	1.03	0.84	0.91	0.81	0.89	4-diphosphocytidyl-2-C-methyl-D-erythritol kinase (CMK) (CDPMEK) identical to 4-diphosphocytidyl-2-C-methyl-D-erythritol kinase, chloroplast precursor (CMK) (4-(cytidine-5'-diphospho)-2-C-methyl- D-erythritol kinase)(CDPMEK) [Arabidopsis thaliana] SWISS-PROT:O81014
AT5G01750	3.10E-02	1.06	1.02	0.96	0.83	0.91	0.82	0.89	expressed protein contains Pfam profile PF04525: Protein of unknown function (DUF567)
AT4G39780	1.87E-02	1.06	1.34	0.97	0.80	0.91	0.59	0.65	AP2 domain-containing transcription factor, putative similar to AP2 domain containing protein RAP2.4,Arabidopsis thaliana
AT5G37750;AT5G	3.63E-02	1.11	1.00	1.01	0.78	0.91	0.78	0.86	[AT5G37750, DNAJ heat shock N-terminal domain-containing protein contains Pfam profile PF00226 DnaJ domain];[AT5G37440, DNAJ heat shock N-terminal domain-containing protein contains Pfam profile PF00226 DnaJ domain]
AT5G43700	1.26E-02	1.15	1.00	1.04	0.57	0.90	0.56	0.62	auxin-responsive protein / indoleacetic acid-induced protein 4 (IAA4) / auxin-induced protein (AUX2-11) identical to SP|P33077 Auxin-responsive protein IAA4 (Indoleacetic acid-induced protein 4) (Auxin-induced protein AUX2-11) (Arabidopsis thaliana)
AT2G21240	4.07E-02	1.14	0.98	1.03	0.84	0.90	0.86	0.95	expressed protein
AT2G39250	7.20E-03	0.98	1.55	0.89	1.09	0.90	0.70	0.78	AP2 domain-containing transcription factor, putative AP2_ARATH Floral homeotic protein APETALA2.(SP:P47927)(Arabidopsis thaliana)
AT5G27560	4.33E-02	1.09	1.01	0.99	0.84	0.90	0.83	0.91	expressed protein hypothetical protein slr1702 - Synechocystis sp., PIR:S75312
AT2G41310	2.06E-02	0.92	1.79	0.83	1.14	0.90	0.64	0.71	two-component responsive regulator / response reactor 3 (RR3) identical to response reactor 3 GI:3273200 from [Arabidopsis thaliana]; contains Pfam profile: PF00072 response regulator receiver domain
AT5G07840;AT5G	1.32E-02	1.14	1.01	1.02	0.91	0.90	0.90	1.00	[AT5G07840, ankyrin repeat family protein contains ankyrin repeats, Pfam:PF00023];[AT5G07830, glycosyl hydrolase family 79 N-terminal domain-containing protein similar to beta-glucuronidase GI:8918740 from [Scutellaria baicalensis]]
AT3G43400	1.14E-02	1.04	1.16	0.93	0.71	0.90	0.62	0.69	phagocytosis and cell motility protein ELMO1-related contains weak similarity to ELMO1 [Mus musculus] gi|16118551|gb|AAL14464
AT2G02230	9.14E-03	1.08	1.01	0.96	0.80	0.90	0.79	0.88	F-box family protein / SKP1 interacting partner 3-related contains similarity to SKP1 interacting partner 3 GI:10716951 from [Arabidopsis thaliana]
AT3G01590	2.71E-02	1.12	0.95	1.00	0.83	0.90	0.87	0.97	aldose 1-epimerase family protein similar to apospory-associated protein C; APOC [Chlamydomonas reinhardtii] GI:6970044 Pfam profile PF01263: Aldose 1-epimerase
AT2G20230	2.80E-02	1.05	1.06	0.94	0.90	0.89	0.85	0.95	expressed protein
AT1G19660	3.44E-02	1.04	1.05	0.93	0.96	0.89	0.91	1.02	wound-responsive family protein similar to wound inducive gene [GI:8096273][Nicotiana tabacum]
AT2G33310	6.18E-03	1.08	1.02	0.96	0.77	0.89	0.75	0.85	auxin-responsive protein / indoleacetic acid-induced protein 13 (IAA13) identical to SP|Q38831 Auxin-responsive protein IAA13 (Indoleacetic acid-induced protein 13) (Arabidopsis thaliana)
AT1G61660	3.03E-02	1.04	1.23	0.93	0.94	0.89	0.77	0.87	basic helix-loop-helix (bHLH) family protein contains Pfam domain, PF00010: Helix-loop-helix DNA-binding domain
AT5G27930	1.24E-02	1.13	0.96	1.00	0.80	0.89	0.84	0.95	protein phosphatase 2C, putative / PP2C, putative protein phosphatase-2C, Mesembryanthemum crystallinum, EMBL:AF075581
AT1G10150	4.54E-02	1.03	1.04	0.91	0.93	0.89	0.89	1.01	expressed protein similar to ESTs gb|T20511, gb|T45308, gb|H36493, and gb|AA651176
AT4G25080	3.39E-02	1.06	1.00	0.94	0.93	0.89	0.93	1.05	magnesium-protoporphyrin O-methyltransferase, putative / magnesium-protoporphyrin IX methyltransferase, putative similar to SP|Q55467 Magnesium-protoporphyrin O-methyltransferase (EC 2.1.1.11) (Magnesium-protoporphyrin IX methyltransferase) (Synechocystis sp.)
AT1G67050	4.70E-02	1.11	1.05	0.98	0.74	0.89	0.71	0.80	expressed protein
AT2G43360	8.76E-03	0.95	1.49	0.84	1.14	0.88	0.77	0.87	biotin synthase (BioB) (BIO2) identical to SP|P54967 Pfam profile PF04055: radical SAM domain protein
AT3G04940	4.41E-02	1.15	0.97	1.01	0.80	0.88	0.82	0.93	cysteine synthase, putative / O-acetylserine (thiol)-lyase, putative / O-acetylserine sulfhydrylase, putative similar to O-acetylserine(thiol) lyase (EC 4.2.99.8) [Brassica juncea] GI:2245144; contains Pfam profile PF00291: Pyridoxal-phosphate dependent enzyme
AT3G61770	3.30E-02	1.12	1.02	0.98	0.78	0.88	0.76	0.87	expressed protein contains Pfam domain, PF02681: Uncharacterized BCR, COG1963
AT3G06410	4.71E-02	1.12	1.05	0.99	0.84	0.88	0.81	0.92	zinc finger (CCCH-type) family protein contains Pfam domain, PF00642: Zinc finger C-x8-C-x5-C-x3-H type (and similar)
AT1G33930	1.43E-02	1.06	1.13	0.93	0.81	0.88	0.72	0.82	avirulence-responsive family protein / avirulence induced gene (AIG1) family protein similar to SP|P54120 AIG1 protein [Arabidopsis thaliana]; contains Pfam profile PF04548: AIG1 family
AT1G12900	2.11E-02	1.04	1.02	0.92	0.93	0.88	0.91	1.03	glyceraldehyde 3-phosphate dehydrogenase, chloroplast, putative / NADP-dependent glyceraldehydephosphate dehydrogenase, putative similar to SP|P25856 Glyceraldehyde 3-phosphate dehydrogenase A, chloroplast precursor (EC 1.2.1.13) (NADP-dependent glyceraldehydephosphate dehydrogenase subunit A) [Arabidopsis thaliana]; contains Pfam profiles PF02800: Glyceraldehyde 3-phosphate dehydrogenase C-terminal domain, PF00044: Glyceraldehyde 3-phosphate dehydrogenase NAD binding domain
AT1G13090	4.96E-02	1.22	1.02	1.07	0.71	0.88	0.70	0.79	cytochrome P450 71B28, putative (CYP71B28) identical to Cytochrome P450 (SP:Q9SAE3) [Arabidopsis thaliana]; strong similarity to gb|X97864 cytochrome P450 from Arabidopsis thaliana and is a member of the PF|00067 Cytochrome P450 family. ESTs gb|N65665, gb|T14112, gb|T76255, gb|T20906 and gb|AI100027 come from this gene
AT1G27090	1.87E-02	1.19	0.99	1.04	0.83	0.88	0.84	0.96	expressed protein similar to gda-1 [Pisum sativum] GI:2765418
AT5G48220	2.88E-03	1.15	0.99	1.01	0.81	0.88	0.82	0.94	indole-3-glycerol phosphate synthase, putative similar to SP|P49572
AT2G32160	2.68E-02	1.05	1.22	0.92	0.76	0.88	0.62	0.71	expressed protein
AT1G48100;AT1G	4.72E-02	1.04	1.45	0.91	1.07	0.88	0.74	0.84	[AT1G48100, glycoside hydrolase family 28 protein / polygalacturonase (pectinase) family protein similar to polygalacturonase PG1 GI:5669846, PG2 GI:5669848 from [Glycine max]; contains PF00295: Glycosyl hydrolases family 28 (polygalacturonases)];[AT1G48110, expressed protein contains Pfam profile PF04146: YTS21-B-like family]
AT1G69790	4.53E-02	1.03	1.20	0.90	1.00	0.88	0.83	0.95	protein kinase, putative similar to protein kinase APK1A [Arabidopsis thaliana] SWISS-PROT:Q06548
AT5G46110	4.61E-02	1.06	1.02	0.93	0.94	0.87	0.93	1.06	phosphate/triose-phosphate translocator, putative identical to phosphate/triose-phosphate translocator precursor [Arabidopsis thaliana] gi|3983125|gb|AAC83815; similar to triose phosphate/phosphate translocator, chloroplast precursor (CTPT)[Cauliflower][Brassica oleracea] SWISS-PROT:P52177
AT2G06530	3.21E-02	1.19	0.98	1.04	0.71	0.87	0.72	0.82	SNF7 family protein contains Pfam domain, PF03357: SNF7 family
AT1G20830	8.12E-03	1.12	0.99	0.98	0.78	0.87	0.79	0.90	expressed protein
AT2G45310	2.27E-02	1.05	1.34	0.91	1.02	0.87	0.76	0.87	NAD-dependent epimerase/dehydratase family protein similar to nucleotide sugar epimerase from Vibrio vulnificus GI:3093975 [PID:g3093975], WbnF [Escherichia coli] GI:5739472, CAPI protein [Staphylococcus aureus] SP|P39858; contains Pfam profile PF01370 NAD dependent epimerase/dehydratase family
AT1G70310	1.68E-02	1.17	0.94	1.02	0.81	0.87	0.86	0.99	spermidine synthase 2 (SPDSYN2) / putrescine aminopropyltransferase 2 identical to SP|O48661 Spermidine synthase 2 (EC 2.5.1.16) (Putrescine aminopropyltransferase 2) (SPDSY 2) (Arabidopsis thaliana)
AT2G41950	3.03E-02	0.95	1.55	0.83	1.05	0.87	0.68	0.78	expressed protein
AT4G09010	2.32E-02	1.14	1.07	0.99	0.90	0.87	0.85	0.97	L-ascorbate peroxidase, chloroplast, putative identical to SP|P82281 (TL29_ARATH (EC 1.11.1.11) [Arabidopsis thaliana]; ascorbate peroxidase - Spinacia oleracea, [gi:1669585]; contains Pfam domain PF00141: Peroxidase; contains TIGRfam domain TIGR01409: Tat (twin-arginine translocation) pathway signal sequence; identical to ascorbate peroxidase APX4 (AT4g09010) mRNA, partial cds GI:31980499
AT5G24490	4.29E-02	1.18	1.01	1.02	0.91	0.87	0.90	1.03	30S ribosomal protein, putative similar to SP|P19954 Plastid-specific 30S ribosomal protein 1, chloroplast precursor (CS-S5) (CS5) (S22) (Ribosomal protein 1) (PSRP-1) (Spinacia oleracea); contains Pfam profile PF02482: Sigma 54 modulation protein / S30EA ribosomal protein
[illegible]	3.92E-02	1.17	1.18	1.01	0.81	0.87	0.69	0.79	ethylene-responsive element-binding family protein similar to SP|O80339 Ethylene responsive element binding factor 3 (AtERF3) [Arabidopsis thaliana]; contains Pfam profile PF00847: AP2 domain
AT5G12130	2.98E-02	1.20	1.02	1.04	0.81	0.87	0.80	0.92	integral membrane TerC family protein contains Pfam profile PF03741: Integral membrane protein TerC family
[illegible]	2.52E-02	1.16	1.09	1.01	0.79	0.87	0.73	0.84	signal peptidase I family protein contains Pfam profile: PF00461 signal peptidase I
AT1G67660	1.87E-02	1.15	1.06	1.00	0.79	0.87	0.75	0.87	expressed protein
AT5G43440	2.98E-02	1.12	1.03	0.97	0.77	0.87	0.75	0.86	2-oxoglutarate-dependent dioxygenase, putative similar to 2A6 (GI:599622) and tomato ethylene synthesis regulatory protein E8 (SP|P10967)
AT2G39870	4.00E-02	0.85	1.80	0.74	1.34	0.87	0.75	0.86	expressed protein
AT1G77850	8.58E-04	1.09	1.06	0.95	0.81	0.87	0.76	0.88	transcriptional factor B3 family protein similar to auxin response factor 10 GI:6165644 from [Arabidopsis thaliana]; contains Pfam profile PF02362: B3 DNA binding domain
AT1G02410	3.46E-02	1.11	1.08	0.96	0.85	0.87	0.79	0.91	cytochrome c oxidase assembly protein CtaG / Cox11 family similar to cytochrome c oxidase assembly protein cox11 GI:1244782 from [Saccharomyces cerevisiae]; similar to Cytochrome c oxidase assembly protein COX11, mitochondrial precursor (SP:Q9Y6N1)(Homo sapiens); contains Pfam profile PF04442: Cytochrome c oxidase assembly protein CtaG / Cox11
AT3G52470	2.93E-02	1.12	1.26	0.96	0.87	0.86	0.69	0.80	harpin-induced family protein / HIN1 family protein / harpin-responsive family protein similar to harpin-induced protein hin1 (GI:1619321) [Nicotiana tabacum]
[illegible]	4.51E-02	1.10	1.01	0.95	0.86	0.86	0.87	1.00	Ran-binding protein 1b (RanBP1b) nearly identical to atranbp1b [Arabidopsis thaliana] GI:2058284
AT3G20500	3.73E-02	1.20	1.04	1.04	0.79	0.86	0.76	0.88	calcineurin-like phosphoesterase family protein contains Pfam profile: PF00149 calcineurin-like phosphoesterase
[illegible]	2.26E-02	1.19	1.03	1.02	0.62	0.86	0.60	0.70	auxin-responsive protein / indoleacetic acid-induced protein 3 (IAA3) identical to SP|Q38822 Auxin-responsive protein IAA3 (Indoleacetic acid-induced protein 3) (Arabidopsis thaliana); EST gb|T04296 comes from this gene
[illegible]	2.27E-02	1.12	1.04	0.96	0.80	0.86	0.73	0.85	expressed protein predicted pectate-lyase, Arabidopsis thaliana, PIR:T06728
AT5G51840	3.43E-02	1.09	1.04	0.94	0.76	0.86	0.73	0.85	expressed protein
AT5G44200	4.36E-02	1.17	1.03	1.00	0.82	0.86	0.80	0.92	nuclear cap-binding protein, putative similar to SP|P52298 20 kDa nuclear cap binding protein (CBP20) (NCBP interacting protein 1) (Homo sapiens); non-consensus AT donor splice site at exon 4, AC acceptor splice site at exon 5; contains InterPro entry IPR000504: RNA-binding region RNP-1 (RNA recognition motif) (RRM)
[illegible]	4.30E-02	1.09	1.06	0.94	0.80	0.86	0.75	0.87	no apical meristem (NAM) family protein contains Pfam PF02365: No apical meristem (NAM) domain
AT1G06830	3.27E-02	0.99	1.25	0.85	0.70	0.86	0.56	0.65	glutaredoxin family protein contains INTERPRO Domain IPR002109, Glutaredoxin (thioltransferase)
[illegible]	[illegible]	[illegible]	[illegible]	[illegible]	[illegible]	[illegible]	[illegible]	[illegible]	[illegible]

AT1G75390	1.14E-02	1.21	1.02	0.94	0.88	0.78	0.86	1.11	bZIP transcription factor family protein contains Pfam profile: PF00170 bZIP transcription factor
AT4G33580	1.18E-02	1.29	1.04	1.00	0.78	0.78	0.75	0.96	carbonic anhydrase family protein / carbonate dehydratase family protein similar to SP|P46512 Carbonic anhydrase 1 (EC 4.2.1.1) (Carbonate dehydratase 1) [Flaveria linearis]; contains Pfam profile PF00484: Carbonic anhydrase
AT1G15980	2.19E-02	1.16	1.10	0.90	0.83	0.78	0.75	0.97	expressed protein
AT5G47330	1.98E-03	1.13	1.74	0.88	0.92	0.78	0.53	0.68	palmitoyl protein thioesterase family protein
AT3G25710	1.39E-02	1.08	1.18	0.84	0.93	0.78	0.79	1.02	basic helix-loop-helix (bHLH) family protein contains Pfam profile: PF00010 helix-loop-helix DNA-binding domain
AT3G28080	1.23E-02	1.16	1.14	0.90	0.71	0.78	0.62	0.80	nodulin MtN21 family protein similar to MtN21 GI:2598575 (root nodule development) from [Medicago truncatula]
AT4G13010	1.08E-02	1.21	1.13	0.94	0.79	0.78	0.70	0.90	oxidoreductase, zinc-binding dehydrogenase family protein low similarity to probable NADP-dependent oxidoreductase (zeta-crystallin homolog) P1 [SP|Q39172][gi:886428] and P2 [SP|Q39173][gi:886430]; contains Pfam profile PF00107: oxidoreductase, zinc-binding dehydrogenase family
AT4G34050	3.57E-02	1.21	1.06	0.94	0.82	0.78	0.77	1.00	caffeoyl-CoA 3-O-methyltransferase, putative nearly identical to GI:2960356 [Populus balsamifera subsp. trichocarpa], GI:684942 [Medicago sativa subsp. sativa] isoform contains a GT-TG intron which removes an internal segment of the protein.
AT1G19330	3.60E-02	1.25	1.32	0.97	0.91	0.78	0.69	0.90	expressed protein
AT1G20440	6.75E-04	1.31	0.99	1.01	0.62	0.78	0.63	0.81	dehydrin (COR47) identical to dehydrin COR47 (Cold-induced COR47 protein) [Arabidopsis thaliana] SWISS-PROT:P31168
AT5G20580	1.02E-02	1.08	1.07	0.84	0.91	0.78	0.85	1.10	expressed protein predicted protein, Arabidopsis thaliana
AT2G16630	1.59E-02	1.08	1.27	0.84	0.93	0.78	0.74	0.95	proline-rich family protein contains proline-rich extensin domains, INTERPRO:IPR002965
AT5G66530	1.43E-02	1.30	1.07	1.00	0.81	0.77	0.76	0.99	aldose 1-epimerase family protein similar to apospory-associated protein C; APOC [Chlamydomonas reinhardtii] GI:6970044; contains Pfam profile PF01263: Aldose 1-epimerase
AT5G01240	2.14E-02	1.26	1.10	0.98	0.85	0.77	0.77	1.00	amino acid permease, putative strong similarity to AUX1 GI:1531758 from [Arabidopsis thaliana]; contains Pfam profile PF01490: Transmembrane amino acid transporter protein
AT1G24400	1.75E-02	0.91	1.10	0.71	1.02	0.77	0.93	1.20	lysine and histidine specific transporter, putative similar to lysine and histidine specific transporter GI:2576361 from [Arabidopsis thaliana]; contains Pfam profile PF01490: Transmembrane amino acid transporter protein
AT2G44920	3.36E-02	0.90	1.61	0.70	1.30	0.77	0.81	1.05	thylakoid lumenal 15 kDa protein, chloroplast identical to SP|O22160 Thylakoid lumenal 15 kDa protein, chloroplast precursor (p15) [Arabidopsis thaliana]; contains 8 pentapeptide repeats
AT2G15310	2.68E-03	1.17	1.21	0.91	0.81	0.77	0.67	0.86	ADP-ribosylation factor, putative similar to ADP-ribosylation factor (GI:861205) [Chlamydomonas reinhardtii]
AT1G62380	1.80E-04	1.26	1.03	0.97	0.84	0.77	0.81	1.05	1-aminocyclopropane-1-carboxylate oxidase, putative / ACC oxidase, putative nearly identical to ACC oxidase (ACC ox1) GI:587086 from [Brassica oleracea]
AT4G38320;AT4G	7.45E-03	1.08	1.14	0.83	0.91	0.77	0.80	1.04	[AT4G38320, expressed protein contains Pfam domain, PF03006: Uncharacterised protein family (Hly-III / UPF0073)] ;[AT4G37680, expressed protein contains Pfam domain, PF03006: Uncharacterised protein family (Hly-III / UPF0073)]
AT2G30420	3.82E-02	1.17	1.33	0.90	0.70	0.77	0.53	0.68	myb family transcription factor contains Pfam profile: PF00249 myb-like DNA-binding domain
AT5G41810	1.45E-02	1.06	1.27	0.82	0.85	0.77	0.67	0.87	expressed protein
AT1G63050	1.09E-02	1.04	1.08	0.81	0.93	0.77	0.86	1.12	membrane bound O-acyl transferase (MBOAT) family protein low similarity to porcupine from [Xenopus laevis] GI:6714514, GI:6714520, GI:6714518, GI:6714516; contains Pfam profile PF03062: MBOAT family
AT5G03700	4.31E-03	0.96	1.31	0.74	1.01	0.77	0.77	1.00	PAN domain-containing protein contains Pfam profile: PF00024 PAN domain
AT5G48640	4.04E-02	1.13	1.07	0.87	0.82	0.77	0.76	0.99	cyclin family protein similar to SP|P55168 Cyclin C [Gallus gallus]; contains Pfam profile PF00134: Cyclin, N-terminal domain
AT1G32190	1.17E-03	1.05	1.25	0.81	0.88	0.77	0.70	0.91	expressed protein
AT3G43670	3.81E-02	1.03	1.12	0.79	0.98	0.77	0.87	1.13	copper amine oxidase, putative similar to copper amine oxidase [Cicer arietinum] gi|3819099|emb|CAA08855
AT5G11410	1.01E-02	1.13	1.31	0.87	0.89	0.77	0.68	0.89	protein kinase family protein contains eukaryotic protein kinase domain, INTERPRO:IPR000719
AT4G26760	3.68E-02	1.33	1.10	1.02	0.82	0.77	0.74	0.96	microtubule associated protein (MAP65/ASE1) family protein low similarity to protein regulating cytokinesis 1 (PRC1) [Homo sapiens] GI:2865521; contains Pfam profile PF03999: Microtubule associated protein (MAP65/ASE1 family)
AT3G44120;AT2G	1.07E-02	1.33	1.02	1.02	0.74	0.77	0.72	0.94	[AT3G44120, F-box family protein contains Pfam:PF00646 F-box domain ; contains TIGRFAM TIGR01640: F-box protein interaction domain ; contains TIGRFAM TIGR01640: F-box protein interaction domain];[AT2G07140, F-box family protein contains Pfam:PF00646 F-box domain ; contains TIGRFAM TIGR01640: F-box protein interaction domain ; contains TIGRFAM TIGR01640: F-box protein interaction domain];[AT3G18320, F-box family protein contains Pfam PF00646: F-box domain; contains TIGRFAM TIGR01640: F-box protein interaction domain; similar to F-box protein family, AtFBX9 (GI:20197985) [Arab[illegible]
AT1G30250	4.51E-02	1.18	0.98	0.91	0.98	0.77	0.99	1.29	expressed protein
AT5G45750	4.13E-02	1.27	1.25	0.97	0.65	0.77	0.52	0.67	Ras-related GTP-binding protein, putative similar to GTP-binding protein GI:303744 from [Pisum sativum]
AT2G25080	2.58E-02	1.29	1.05	1.00	0.71	0.77	0.68	0.88	phospholipid hydroperoxide glutathione peroxidase, chloroplast / PHGPx (GPX1) identical to SP|P52032 Phospholipid hydroperoxide glutathione peroxidase, chloroplast precursor (EC 1.11.1.9) (PHGPx) [Arabidopsis thaliana]; contains Glutathione peroxidases signatures, Glutathione_Peroxid_1 [GKVMLIVNVASRCGLT], Glutathione_Peroxid_2 [LAFPCNQF]; contains EST gb:T43669, N38679, R30227, H37043, AA042773; identical to cDNA chloroplast mRNA for glutathione peroxidase GI:2274856
AT4G25910	2.19E-02	1.23	1.12	0.95	0.66	0.77	0.59	0.76	nitrogen fixation protein, putative nitrogen fixation protein nifU (SP:Q43885) [Anabaena sp.]; contains Pfam profile: PF01106 NifU-like domain
AT5G54930	3.32E-02	1.11	1.14	0.85	0.93	0.77	0.81	1.06	AT hook motif-containing protein contains Pfam profile PF02178: AT hook motif
AT2G01150	4.02E-02	1.17	1.23	0.90	0.82	0.77	0.67	0.87	zinc finger (C3HC4-type RING finger) family protein contains Pfam profile: PF00097 zinc finger, C3HC4 type (RING finger)
AT2G21890;AT2G	8.24E-04	0.88	1.81	0.68	1.17	0.77	0.65	0.84	[AT2G21890, mannitol dehydrogenase, putative similar to ELI3-2 (SP|Q02972), sinapyl alcohol dehydrogenase (Populus tremuloides) (gi:14279694); contains Pfam zinc-binding dehydrogenase domain PF00107];[AT2G21730, mannitol dehydrogenase, putative similar to ELI3-2 (SP|Q02972), sinapyl alcohol dehydrogenase (Populus tremuloides) (gi:14279694); contains Pfam zinc-binding dehydrogenase domain PF00107]
AT1G08540	4.00E-02	1.21	1.03	0.93	0.83	0.77	0.81	1.05	RNA polymerase sigma subunit SigB (sigB) / sigma factor 2 (SIG2) identical to sigma factor SigB [Arabidopsis thaliana] GI:5478446, plastid RNA polymerase sigma-subunit [Arabidopsis thaliana] GI:2879922, sigma factor [Arabidopsis thaliana] GI:2597831, sigma factor 2 (SIG2) [Arabidopsis thaliana] GI:2353173; contains Pfam profiles PF04545: Sigma-70, region 4, PF04539: Sigma-70 region 3, PF04542: Sigma-70 region 2
AT3G06160	2.87E-02	1.04	1.20	0.80	0.87	0.77	0.72	0.94	transcriptional factor B3 family protein contains Pfam profile PF02362: B3 DNA binding domain
AT5G41400	8.31E-03	1.19	1.35	0.91	0.71	0.77	0.53	0.69	zinc finger (C3HC4-type RING finger) family protein similar to RING-H2 finger protein RHA1a [Arabidopsis thaliana] GI:3790554; contains Pfam profile PF00097: Zinc finger, C3HC4 type (RING finger)
AT1G67730	4.67E-03	1.20	1.01	0.92	0.81	0.77	0.80	1.04	b-keto acyl reductase, putative (GLOSSY8) similar to b-keto acyl reductase GI:2586127 from [Hordeum vulgare]
AT2G23420	8.05E-03	1.11	1.14	0.85	0.79	0.77	0.70	0.91	nicotinate phosphoribosyltransferase family protein / NAPRTase family protein contains Pfam domain PF04095: Nicotinate phosphoribosyltransferase (NAPRTase)
AT1G27090	8.47E-03	1.14	1.15	0.88	0.97	0.77	0.85	1.11	glycine-rich protein
AT1G80440	3.21E-02	1.09	1.17	0.84	0.99	0.77	0.85	1.11	kelch repeat-containing F-box family protein similar to SP|Q9ER30 Kelch-related protein 1 (Sarcosin) [Rattus norvegicus]; contains Pfam profiles PF01344: Kelch motif, PF00646: F-box domain
AT4G15500	2.09E-02	1.14	1.11	0.88	0.95	0.77	0.85	1.11	UDP-glucoronosyl/UDP-glucosyl transferase family protein contains Pfam profile: PF00201 UDP-glucoronosyl and UDP-glucosyl transferase
AT4G04630	4.29E-02	1.17	1.10	0.89	0.72	0.77	0.66	0.86	expressed protein contains Pfam profile PF04520: Protein of unknown function, DUF584
AT3G61950	1.73E-02	1.18	1.09	0.90	0.95	0.77	0.87	1.13	basic helix-loop-helix (bHLH) family protein contains Pfam profile: PF00010 helix-loop-helix DNA-binding domain
AT3G05750	4.57E-02	1.08	1.08	0.83	0.99	0.77	0.92	1.20	expressed protein
AT3G22530	4.61E-02	1.28	1.10	0.98	0.77	0.77	0.70	0.91	expressed protein contains Pfam profile:PF00011 HSP20:Hsp20/alpha crystallin family
AT5G64770	1.56E-02	1.21	1.16	0.93	0.51	0.77	0.44	0.57	expressed protein
AT3G13510	4.29E-02	1.13	1.01	0.87	0.92	0.77	0.91	1.18	expressed protein Pfam profile PF03080: Arabidopsis proteins of unknown function
AT4G34680	4.08E-02	1.17	1.18	0.90	0.91	0.77	0.77	1.01	GATA transcription factor 3, putative (GATA-3) identical to GATA-binding transcription factor GATA-3 [Arabidopsis thaliana] gi|2959734|emb|CAA74001; identical to cDNA for GATA transcription factor 3 GI:2959733
AT2G30620	4.33E-03	1.11	1.06	0.85	0.84	0.77	0.80	1.05	histone H1.2 nearly identical to SP|P26569 Histone H1.2 [Arabidopsis thaliana]
AT4G18910	1.56E-02	1.06	1.18	0.81	0.81	0.77	0.69	0.90	aquaglyceroporin / NOD26-like major intrinsic protein 2 (NLM2) contains Pfam profile: MIP PF00230; similar to SP:P08995 (Glycine max) Nodulin-26 (N-26); identical to cDNA aquaglyceroporin (nlm2 gene) GI:11071655, aquaglyceroporin [Arabidopsis thaliana] GI:11071656
AT5G55050	2.99E-03	1.09	1.36	0.83	0.71	0.77	0.52	0.68	GDSL-motif lipase/hydrolase family protein similar to family II lipases EXL3 GI:15054386, EXL1 GI:15054382, EXL2 GI:15054384 from [Arabidopsis thaliana]; contains Pfam profile PF00657: GDSL-like Lipase/Acylhydrolase
AT5G06270	3.03E-02	1.44	0.90	1.10	0.66	0.76	0.73	0.95	expressed protein
AT4G00820	2.74E-02	1.14	1.03	0.87	0.91	0.76	0.89	1.16	calmodulin-binding protein-related contains Pfam profile PF00612: IQ calmodulin-binding motif
AT1G76900	1.14E-02	1.06	1.13	0.81	0.88	0.76	0.78	1.02	F-box family protein / tubby family protein similar to Tubby protein (SP:P50586) [Mus musculus]; similar to Chain A, C-Terminal Domain Of Mouse Brain Tubby Protein (GI:6730158) [Mus musculus]; similar to Tubby related protein 1 (Tubby-like protein 1) (Swiss-Prot:O00294) [Homo sapiens]; similar to phosphodiesterase (GI:467578) [Mus musculus]; contains Pfam profile: PF01167: Tub family; contains Pfam PF00646: F-box domain
[illegible]	[illegible]	[illegible]	[illegible]	[illegible]	[illegible]	[illegible]	[illegible]	[illegible]	[illegible]

AT3G30300	2.55E-02	1.16	1.06	0.79	0.87	0.68	0.82	1.21	expressed protein contains Pfam PF03138: Plant protein family. The function of this family of plant proteins is unknown; previously annotated as 'auxin-independent growth promoter -related' based on similarity to axi 1 protein (GB:X80301) (GI:559920) from [Nicotiana [illegible]
AT3G52430	3.09E-03	1.17	1.53	0.80	0.82	0.68	0.53	0.79	phytoalexin-deficient 4 protein (PAD4) identical to phytoalexin-deficient 4 protein [Arabidopsis thaliana] GI:6457331; contains Pfam profile PF01764: Lipase
AT4G29070	9.50E-03	1.33	1.13	0.90	0.70	0.68	0.62	0.91	expressed protein
AT5G15580	3.22E-02	1.41	1.02	0.96	0.79	0.68	0.78	1.14	expressed protein unknown protein F14P3.18 - Arabidopsis thaliana, EMBL:AC009755
AT5G45670	1.46E-02	1.26	1.22	0.86	0.74	0.68	0.61	0.89	GDSL-motif lipase/hydrolase family protein similar to family II lipases EXL3 GI:15054386, EXL1 GI:15054382, EXL2 GI:15054384 from [Arabidopsis thaliana]; contains Pfam profile PF00657: GDSL-like Lipase/Acylhydrolase
AT2G01890	1.42E-02	1.28	1.36	0.87	0.82	0.68	0.60	0.89	purple acid phosphatase, putative contains Pfam profile: PF00149 calcineurin-like phosphoesterase; identical to purple acid phosphatase (GI:20257479) [Arabidopsis thaliana];
AT1G17220	5.00E-02	1.18	1.09	0.80	0.94	0.68	0.86	1.27	translation initiation factor IF-2, chloroplast, putative similar to SP|P57997|IF2C_PHAVU Translation initiation factor IF-2, chloroplast precursor (PvIF2cp) (Phaseolus vulgaris)
AT1G72910;AT1G	1.39E-03	1.16	1.24	0.79	0.63	0.68	0.51	0.75	[AT1G72910, disease resistance protein (TIR-NBS class), putative domain signature TIR-NBS exists, suggestive of a disease resistance protein.];[AT1G72930, Toll-Interleukin-Resistance (TIR) domain-containing protein domain signature TIR exists, suggestive of a disease [illegible]
AT1G04350	3.02E-02	1.35	1.16	0.92	0.77	0.68	0.66	0.97	2-oxoglutarate-dependent dioxygenase, putative Similar to Arabidopsis 2A6 (gb|X83096) and to tomato ethylene synthesis regulatory protein E8 (SP|P10967); EST gb|T76913 comes from this gene
AT5G25840	4.13E-02	1.34	1.35	0.91	0.78	0.68	0.58	0.85	expressed protein
AT4G01680	1.24E-02	1.40	1.08	0.95	0.75	0.68	0.69	1.02	myb family transcription factor (MYB55)
AT5G62430	1.78E-02	1.33	1.32	0.90	0.86	0.68	0.65	0.95	Dof-type zinc finger domain-containing protein similar to H-protein promoter binding factor-2a GI:3386546 from [Arabidopsis thaliana]
AT5G03430	2.91E-02	1.33	1.08	0.90	0.95	0.68	0.88	1.30	phosphoadenosine phosphosulfate (PAPS) reductase family protein low similarity to SP|P38913 FAD synthetase (EC 2.7.7.2) (Saccharomyces cerevisiae); contains Pfam profiles PF01507: Phosphoadenosine phosphosulfate reductase family, PF00994: Probable molybdopterin [illegible]
AT1G10340	4.09E-04	1.26	1.25	0.85	0.69	0.68	0.55	0.82	ankyrin repeat family protein contains ankyrin repeat domains, Pfam:PF00023
AT4G30080	6.80E-04	1.26	1.05	0.86	0.78	0.68	0.74	1.10	transcriptional factor B3 family protein contains Pfam profile: PF02362 B3 DNA binding domain
AT5G11460	7.40E-03	1.26	1.12	0.85	0.89	0.68	0.80	1.18	senescence-associated protein-related similar to senescence-associated protein SAG102 (GI:22331931) [Arabidopsis thaliana]
AT5G58260	2.38E-02	1.23	1.23	0.83	0.73	0.68	0.59	0.87	expressed protein
AT1G55910	3.09E-03	1.28	1.56	0.86	0.84	0.68	0.54	0.79	metal transporter, putative (ZIP11) similar to putative zinc transporter ZIP2 [Arabidopsis thaliana] gi|3252868|gb|AAC24198; member of the Zinc (Zn2+)-Iron (Fe2+) permease (ZIP) family, PMID:11500563
AT2G32640	2.27E-02	1.12	1.14	0.75	0.89	0.68	0.78	1.15	expressed protein
AT5G56860	9.48E-03	1.26	1.61	0.85	0.89	0.68	0.56	0.82	zinc finger (GATA type) family protein similar to unknown protein (pir |T04270)
AT3G18110	4.11E-02	1.17	1.07	0.79	0.95	0.68	0.89	1.32	pentatricopeptide (PPR) repeat-containing protein contains Pfam profile: PF01535 PPR repeat
AT3G12710	2.32E-02	1.41	1.27	0.95	0.74	0.68	0.58	0.86	methyladenine glycosylase family protein similar to SP|P05100 DNA-3-methyladenine glycosylase I (EC 3.2.2.20) (3-methyladenine-DNA glycosylase I, constitutive) (Escherichia coli); contains Pfam profile PF03352: Methyladenine glycosylase
AT2G38120	6.16E-03	0.98	1.42	0.66	1.06	0.68	0.75	1.11	amino acid permease, putative (AUX1) identical to AUX1 GI:1531758 from [Arabidopsis thaliana]
AT1G54200	3.29E-02	1.18	1.34	0.80	0.86	0.68	0.64	0.94	expressed protein
AT5G25210	5.06E-03	1.32	1.23	0.89	0.74	0.67	0.60	0.89	expressed protein
AT5G04150	1.06E-02	1.28	1.26	0.86	0.76	0.67	0.61	0.90	basic helix-loop-helix (bHLH) family protein contains Pfam profile: PF00010 helix-loop-helix DNA-binding domain; PMID: 12679534
AT1G59700	2.65E-02	1.47	1.28	0.99	0.67	0.67	0.52	0.78	glutathione S-transferase, putative similar to glutathione S-transferase GB:AAF29773 GI:6856103 from [Gossypium hirsutum]
AT4G09900	5.62E-04	1.26	1.17	0.85	0.75	0.67	0.64	0.96	hydrolase, alpha/beta fold family protein similar to ethylene-induced esterase [Citrus sinensis] GI:14279437, polyneuridine aldehyde esterase [Rauvolfia serpentina] GI:6651393, SP|Q40708 PIR7A protein (Oryza sativa); contains Pfam profile PF00561: hydrolase, alpha/beta [illegible]
AT1G67970	3.15E-02	1.36	1.22	0.92	0.83	0.67	0.68	1.01	heat shock factor protein, putative (HSF5) / heat shock transcription factor, putative (HSTF5) identical to heat shock transcription factor 5 (HSF5) SP:Q9S7U5 from [Arabidopsis thaliana]; contains Pfam profile: PF00447 HSF-type DNA-binding domain
AT2G32950	1.27E-02	1.29	1.07	0.87	0.74	0.67	0.69	1.03	COP1 regulatory protein photomorphogenesis repressor; identical to COP1 regulatory protein/FUSCA protein FUS1 GI:402685 SP:P43254
AT1G23205	3.87E-03	1.29	1.33	0.87	0.82	0.67	0.62	0.92	invertase/pectin methylesterase inhibitor family protein low similarity to pectinesterase from Phaseolus vulgaris SP|Q43111, Lycopersicon esculentum SP|Q43143; contains Pfam profile PF04043: Plant invertase/pectin methylesterase inhibitor
AT4G29740	3.33E-02	1.08	1.35	0.73	0.86	0.67	0.64	0.95	FAD-binding domain-containing protein / cytokinin oxidase family protein similar to cytokinin oxidase, Zea mays [gi:3882018] [gi:3441978]
AT2G21650	2.78E-03	1.40	1.06	0.94	0.42	0.67	0.39	0.58	myb family transcription factor contains PFAM profile: PF00249 myb-like DNA-binding domain
AT5G49630	8.31E-03	1.06	1.37	0.71	0.93	0.67	0.68	1.01	amino acid permease 6 (AAP6) identical to amino acid permease 6 (AAP6) [Arabidopsis thaliana] GI:1769887
AT5G48490	3.76E-02	1.06	1.34	0.71	0.66	0.67	0.49	0.73	protease inhibitor/seed storage/lipid transfer protein (LTP) family protein contains Pfam protease inhibitor/seed storage/LTP family domain PF00234
AT4G37760	2.65E-02	1.12	1.13	0.75	0.89	0.67	0.79	1.17	squalene monooxygenase, putative / squalene epoxidase, putative similar to SP|O65404 (SE 1,1), SP|O65402 (SE 1,2), SP|O65403 (SE 2)
AT3G26570	3.26E-02	1.07	1.36	0.72	1.00	0.67	0.74	1.10	phosphate transporter family protein contains Pfam profile: PF01384 phosphate transporter family
AT5G01220	9.32E-03	1.08	1.09	0.73	0.92	0.67	0.84	1.25	UDP-sulfoquinovose:DAG sulfoquinovosyltransferase / sulfolipid synthase (SQD2) identical to GI:20302857
AT2G42690	3.40E-03	0.84	1.80	0.56	1.23	0.67	0.69	1.02	lipase, putative similar to lipase [Dianthus caryophyllus] GI:4103627; contains Pfam profile PF01764: Lipase
AT5G46570	2.30E-03	1.16	1.09	0.78	0.82	0.67	0.75	1.11	protein kinase family protein contains protein kinase domain, Pfam:PF00069
AT1G68670	4.67E-02	1.27	1.21	0.85	0.76	0.67	0.63	0.94	myb family transcription factor contains Pfam domain, PF00249: Myb-like DNA-binding domain
AT4G38430	1.10E-02	1.23	1.08	0.83	0.85	0.67	0.79	1.18	expressed protein contains Pfam profile PF03759: Domain of unknown function (DUF315)
AT5G02480	3.38E-02	1.33	1.03	0.89	0.75	0.67	0.73	1.09	expressed protein p
AT1G05200	2.27E-02	1.12	1.14	0.75	1.02	0.67	0.90	1.35	glutamate receptor family protein (GLR3.4) plant glutamate receptor family, PMID:11379626
AT3G04010	3.71E-02	1.50	1.09	1.00	0.77	0.67	0.71	1.07	glycosyl hydrolase family 17 protein similar to beta-1,3-glucanase GB:S12402 [Nicotiana sp], GB:CAA03908 [Citrus sinensis], GB:S44364 [Lycopersicon esculentum]
AT4G18970	1.08E-02	1.25	1.31	0.84	0.75	0.67	0.57	0.86	GDSL-motif lipase/hydrolase family protein similar to family II lipases EXL3 GI:15054386, EXL1 GI:15054382, EXL2 GI:15054384 from [Arabidopsis thaliana]; contains Pfam profile PF00657: GDSL-like Lipase/Acylhydrolase
AT3G29760	9.53E-03	1.31	1.11	0.87	0.90	0.67	0.81	1.21	NLI interacting factor (NIF) family protein contains Pfam profile PF03031: NLI interacting factor
AT5G15210	3.46E-02	1.13	1.32	0.76	1.00	0.67	0.76	1.14	zinc finger homeobox family protein / ZF-HD homeobox family protein various predicted proteins, Arabidopsis thaliana
AT3G15090	8.45E-03	1.30	1.15	0.87	0.78	0.67	0.68	1.02	oxidoreductase, zinc-binding dehydrogenase family protein low similarity to NOGO-interacting mitochondrial protein from Mus musculus [gi:14522884]; contains Pfam profile: PF00107 zinc-binding dehydrogenases
AT5G66080	1.51E-02	1.04	1.11	0.69	0.90	0.67	0.81	1.21	protein phosphatase 2C family protein / PP2C family protein similar to Ser/Thr protein phosphatase 2C (PP2C6) (GI:15020818) [Arabidopsis thaliana]; similar to protein phosphatase 2C (GI:3608412) [Mesembryanthemum crystallinum]; contains Pfam PF00481 : Protein [illegible]
AT2G39220	9.08E-03	0.96	1.61	0.64	1.11	0.67	0.69	1.04	patatin family protein similar to patatin-like latex allergen [Hevea brasiliensis][PMID:10589016]; contains patatin domain PF01734
AT3G16940	2.65E-03	1.31	1.14	0.87	0.69	0.67	0.61	0.91	expressed protein contains Pfam profile PF04828: Protein of unknown function (DUF636)
AT1G68780	1.07E-02	1.29	1.17	0.86	0.74	0.67	0.63	0.95	leucine-rich repeat family protein contains leucine rich-repeat (LRR) domains Pfam:PF00560, INTERPRO:IPR001611; contains similarity to disease resistance protein [Lycopersicon esculentum] gi|3894383|gb|AAC78591
AT5G15050	6.95E-03	1.31	1.14	0.87	0.83	0.66	0.72	1.09	glycosyltransferase family 14 protein / core-2/I-branching enzyme family protein contains Pfam profile: PF02485 Core-2/I-Branching enzyme
AT5G65310	4.33E-03	1.40	1.04	0.93	0.70	0.66	0.67	1.01	homeobox-leucine zipper protein 5 (HB-5) / HD-ZIP transcription factor 5 identical to homeobox-leucine zipper protein ATHB-5 (HD-ZIP protein ATHB-5) (SP:P46667) [Arabidopsis thaliana]
AT2G30150	1.31E-02	1.31	1.06	0.87	0.84	0.66	0.79	1.19	UDP-glucoronosyl/UDP-glucosyl transferase family protein contains Pfam profile: PF00201 UDP-glucoronosyl and UDP-glucosyl transferase
AT5G45430	1.60E-02	1.18	1.38	0.78	1.02	0.66	0.74	1.12	protein kinase, putative contains similarity to male germ cell-associated kinase [Homo sapiens] gi|23268497|gb|AAN16405
AT5G52900	1.05E-02	1.26	1.57	0.84	0.56	0.66	0.36	0.54	expressed protein
AT1G06040	5.64E-03	1.36	1.25	0.90	0.82	0.66	0.65	0.99	zinc finger (B-box type) family protein / salt-tolerance protein (STO) identical to SP|Q96288 Salt-tolerance protein [Arabidopsis thaliana]; contains Pfam profile PF00643: B-box zinc finger
AT2G25680	2.78E-02	1.38	1.15	0.91	0.75	0.66	0.65	0.99	expressed protein ; expression supported by MPSS
AT1G05140	4.77E-03	1.29	1.15	0.85	0.84	0.66	0.73	1.11	membrane-associated zinc metalloprotease, putative similar to Hypothetical zinc metalloprotease All3971 (SP:Q8YQ64) [strain PCC 7120] (Anabaena sp.); Similar to Synechocystis hypothetical protein (gb|D90908);contains Pfam PF00595: PDZ domain (Also known as DHR or [illegible]
AT3G10840	8.26E-03	1.28	1.24	0.84	0.81	0.66	0.65	0.98	hydrolase, alpha/beta fold family protein low similarity to hydrolase [Terrabacter sp. DBF63] GI:14196240; contains Pfam profile: PF00561 alpha/beta hydrolase fold
AT3G45730	6.90E-03	1.51	1.00	1.00	0.45	0.66	0.45	0.67	expressed protein
AT5G38510	1.49E-02	1.41	1.20	0.93	0.77	0.66	0.64	0.97	rhomboid family protein contains Pfam profile PF01694: Rhomboid family
AT2G37450	9.19E-03	0.89	1.39	0.59	1.15	0.66	0.83	1.25	nodulin MtN21 family protein similar to MtN21 GI:2598575 (root nodule development) from [Medicago truncatula]
AT3G21700	9.55E-03	1.07	1.09	0.71	0.94	0.66	0.86	1.31	expressed protein
AT5G55740	4.04E-02	1.21	1.19	0.80	0.82	0.66	0.69	1.04	pentatricopeptide (PPR) repeat-containing protein contains INTERPRO:IPR002885 PPR repeats
[illegible]									

AT1G13650	4.40E-03	1.36	1.18	0.76	0.70	0.56	0.59	1.06	expressed protein
AT1G60800	2.85E-02	1.18	1.10	0.66	1.03	0.56	0.93	1.66	leucine-rich repeat family protein / protein kinase family protein contains Pfam domains PF00560: Leucine Rich Repeat and PF00069: Protein kinase domain
AT2G21560	5.82E-03	1.58	1.18	0.89	0.85	0.56	0.72	1.29	expressed protein contains weak similarity to reticulocyte-binding protein 2 homolog A [Plasmodium falciparum] gi|9754767|gb|AAF98066
AT5G59130	4.41E-02	1.18	1.67	0.66	1.04	0.56	0.62	1.10	subtilase family protein contains similarity to prepro-cucumisin GI:807698 from [Cucumis melo]
AT3G45210	7.42E-03	1.46	2.02	0.82	0.86	0.56	0.43	0.76	expressed protein contains Pfam profile PF04520: Protein of unknown function, DUF584
AT2G30520	1.28E-02	1.31	1.29	0.73	0.79	0.56	0.61	1.10	signal transducer of phototropic response (RPT2) identical to RPT2 [Arabidopsis thaliana] gi|6959488|gb|AAF33112
AT5G67030	3.98E-02	1.32	1.51	0.74	1.13	0.56	0.75	1.33	zeaxanthin epoxidase (ZEP) (ABA1) identical to GI:9857296 AtABA1; controls Pfam profiles PF01360: Monooxygenase and PF00498: FHA domain; identical to cDNA AtABA1, GI:9857295
AT5G54510	2.08E-02	1.41	1.13	0.79	0.95	0.56	0.84	1.51	auxin-responsive GH3 protein, putative (DFL-1) identical to auxin-responsive GH3 homologue [Arabidopsis thaliana] GI:11041726; similar to auxin-responsive GH3 product [Glycine max] GI:18591; contains Pfam profile PF03321: GH3 auxin-responsive promoter
AT1G62510	8.23E-03	1.64	1.24	0.92	0.47	0.56	0.38	0.67	protease inhibitor/seed storage/lipid transfer protein (LTP) family protein contains Pfam protease inhibitor/seed storage/LTP family domain PF00234
AT1G53035	4.37E-04	1.50	1.08	0.84	0.49	0.56	0.46	0.81	expressed protein
AT4G34220	5.94E-03	1.08	1.20	0.60	0.78	0.56	0.65	1.18	leucine-rich repeat transmembrane protein kinase, putative protein kinase TMKL1, Arabidopsis thaliana, PID:E353150
AT1G52830	6.19E-03	1.56	1.88	0.86	0.66	0.55	0.35	0.63	auxin-responsive protein / indoleacetic acid-induced protein 6 (IAA6) nearly identical to SP|Q38824 Auxin-responsive protein IAA6 (Indoleacetic acid-induced protein 6) (Arabidopsis thaliana)
AT1G79270	2.62E-03	1.40	1.18	0.78	0.59	0.55	0.50	0.90	expressed protein contains Pfam profile PF04146: YTS21-B-like family
AT3G02910	8.19E-04	1.48	1.13	0.82	0.50	0.55	0.44	0.80	expressed protein contains Pfam domain PF03674: Uncharacterised protein family (UPF0131)
AT1G26560	1.81E-04	1.25	1.32	0.69	0.69	0.55	0.52	0.95	glycosyl hydrolase family 1 protein contains Pfam PF00232 : Glycosyl hydrolase family 1 domain; TIGRFAM TIGR01233: 6-phospho-beta-galactosidase; similar to amygdalin hydrolase isoform AH I precursor (GI:16757966) [Prunus serotina]
AT3G54720	2.77E-03	1.23	1.49	0.68	0.96	0.55	0.65	1.17	glutamate carboxypeptidase, putative (AMP1) identical to GI:15624092 glutamate carboxypeptidase [Arabidopsis thaliana]; ileal peptidase, Rattus norvegicus, EMBL:AF009921; identical to cDNA glutamate carboxypeptidase (AMP1) GI:15624091; contains Pfam profiles PF04389: Peptidase family M28, PF04253: Transferrin receptor-like dimerisation domain and PF02225 PA domain
AT1G66330	4.97E-04	1.34	1.46	0.74	0.83	0.55	0.57	1.03	senescence-associated family protein similar to senescence-associated protein (GI:12836895) [Ipomoea batatas]
AT3G25600	3.57E-03	1.37	1.38	0.75	0.64	0.55	0.47	0.85	calmodulin, putative similar to calmodulin GI:239841 from [Paramecium tetraurelia]
AT5G59070	8.11E-04	1.54	2.46	0.85	0.84	0.55	0.34	0.63	glycosyl transferase family 1 protein contains Pfam profile: PF00534 Glycosyl transferases group 1
AT4G36540	1.12E-04	1.43	1.41	0.79	0.59	0.55	0.42	0.77	basic helix-loop-helix (bHLH) family protein contains Pfam domain, PF00010: Helix-loop-helix DNA-binding domain
AT5G55970	4.42E-04	1.27	1.26	0.69	0.90	0.55	0.71	1.30	zinc finger (C3HC4-type RING finger) family protein contains Pfam profile: PF00097 zinc finger, C3HC4 type (RING finger)
AT2G29300;AT2G	2.53E-03	1.28	1.53	0.70	0.75	0.55	0.49	0.89	[AT2G29300, tropinone reductase, putative / tropine dehydrogenase, putative similar to tropinone reductase SP:P50165 from [Datura stramonium]];[AT2G29320, tropinone reductase, putative / tropine dehydrogenase, putative similar to tropinone reductase SP:P50165 from [Datura stramonium]]
AT3G57040	4.82E-04	1.38	1.38	0.76	0.62	0.55	0.45	0.82	two-component responsive regulator / response reactor 4 (RR4) identical to responce reactor4 GI:3273202 from [Arabidopsis thaliana]; contains Pfam profile: PF00072 response regulator receiver domain
AT2G03530	4.31E-04	1.61	1.27	0.88	0.73	0.55	0.57	1.05	F-box family protein-related similar to A3 protein (Swiss-Prot:Q41706)(unknown function) [Vigna unguiculata]; contains 9 transmembrane domains; supported by tandem duplication of F-box family protein (GI:3805763) (TIGR_Ath1:At2g07140) [Arabidopsis thaliana]
AT5G67390	6.70E-03	1.54	1.58	0.84	0.82	0.55	0.52	0.96	expressed protein
AT2G15480	4.99E-02	1.42	1.19	0.78	0.95	0.55	0.80	1.46	UDP-glucoronosyl/UDP-glucosyl transferase family protein contains Pfam profile: PF00201 UDP-glucoronosyl and UDP-glucosyl transferase
AT1G11410	7.53E-04	1.07	1.12	0.58	0.83	0.54	0.75	1.37	S-locus protein kinase, putative similar to receptor-like protein kinase [Arabidopsis thaliana] gi|4008008|gb|AAC95352; contains S-locus glycoprotein family domain, Pfam:PF00954
AT5G02830	1.01E-02	1.24	1.27	0.68	0.92	0.54	0.72	1.33	pentatricopeptide (PPR) repeat-containing protein contains Pfam profile PF01535: PPR repeat
AT3G22420	8.05E-04	1.46	1.20	0.79	0.79	0.54	0.66	1.21	protein kinase family protein contains protein kinase domain, Pfam:PF00069
AT1G44446	4.87E-03	1.15	1.40	0.63	0.90	0.54	0.64	1.18	chlorophyll a oxygenase (CAO) / chlorophyll b synthase identical to chlorophyll a oxygenase GI:5853117 from [Arabidopsis thaliana]; contains Pfam PF00355 Rieske [2Fe-2S] domain
AT3G61220	4.26E-03	1.47	1.17	0.80	0.57	0.54	0.48	0.89	short-chain dehydrogenase/reductase (SDR) family protein similar to carbonyl reductase GI:1049108 from [Mus musculus]
AT5G52780	3.68E-03	1.33	1.38	0.72	0.51	0.54	0.37	0.69	expressed protein similar to unknown protein (pir||T04431)
AT3G16800	7.78E-05	1.22	1.61	0.66	0.80	0.54	0.50	0.92	protein phosphatase 2C, putative / PP2C, putative similar to protein phosphatase-2C GB:AAC36699 from [Mesembryanthemum crystallinum]
AT4G27260	6.85E-04	1.24	1.21	0.67	0.74	0.54	0.61	1.13	auxin-responsive GH3 family protein similar to auxin-responsive GH3 product [Glycine max] GI:18591; contains Pfam profile PF03321: GH3 auxin-responsive promoter
AT3G56880	1.37E-02	1.41	1.43	0.76	0.64	0.54	0.45	0.83	VQ motif-containing protein contains PF05678: VQ motif
AT5G58770	3.22E-03	1.47	1.53	0.79	0.49	0.54	0.32	0.60	dehydrodolichyl diphosphate synthase, putative / DEDOL-PP synthase, putative similar to GI:796076
AT2G32540	1.12E-03	1.42	1.49	0.76	0.84	0.54	0.56	1.04	cellulose synthase family protein similar to cellulose synthase catalytic subunit from Arabidopsis thaliana [gi:5230423], cellulose synthase-5 from Zea mays [gi:9622882]
AT3G01440	2.02E-03	1.53	1.24	0.82	0.50	0.54	0.41	0.76	oxygen evolving enhancer 3 (PsbQ) family protein photosystem II oxygen-evolving complex protein; contains Pfam profile PF05757: Oxygen evolving enhancer protein 3 (PsbQ)
AT5G15160	8.97E-03	1.54	1.33	0.82	0.50	0.54	0.38	0.71	bHLH family protein
AT2G43290	5.40E-03	1.32	2.42	0.71	0.72	0.53	0.30	0.55	calmodulin-like protein (MSS3) identical to calmodulin-like MSS3 from GI:9965747 [Arabidopsis thaliana]
AT1G73630	2.24E-03	1.36	1.35	0.73	0.59	0.53	0.44	0.82	calcium-binding protein, putative similar to calcium binding protein GI:14589311 from [Sesbania rostrata]; contains Pfam profile: PF00036 EF hand (4 copies)
AT3G46600	2.92E-02	1.39	1.17	0.74	0.91	0.53	0.78	1.47	scarecrow transcription factor family protein scarecrow-like 11 - Arabidopsis thaliana, EMBL:AF036307
AT1G10640	3.08E-04	1.48	1.23	0.79	0.67	0.53	0.54	1.02	polygalacturonase, putative / pectinase, putative similar to polygalacturonase PG1 GI:5669846, PG2 GI:5669848 from [Glycine max]; contains PF00295: Glycosyl hydrolases family 28 (polygalacturonases)
AT1G01390	5.76E-03	1.33	1.39	0.71	0.79	0.53	0.57	1.07	UDP-glucoronosyl/UDP-glucosyl transferase family protein contains Pfam profile: PF00201 UDP-glucoronosyl and UDP-glucosyl transferase
AT3G26310	3.09E-03	1.31	1.22	0.70	0.83	0.53	0.68	1.28	cytochrome P450 family protein contains Pfam profile: PF00067 cytochrome P450
AT1G23740	2.68E-03	1.40	1.56	0.75	0.85	0.53	0.55	1.03	oxidoreductase, zinc-binding dehydrogenase family protein contains Pfam profile PF00107: oxidoreductase, zinc-binding dehydrogenase family
AT5G11260	9.72E-03	1.71	1.18	0.91	0.72	0.53	0.61	1.14	bZIP protein HY5 (HY5) identical to HY5 protein GI:2251085 from [Arabidopsis thaliana]
AT1G64950	3.49E-02	1.86	0.94	0.99	0.59	0.53	0.63	1.18	cytochrome P450, putative similar to cytochrome P450 89A2 (CYPLXXXIX) (SP:Q42602) [Arabidopsis thaliana];similar to cytochrome P450 (GI:438242) [Solanum melongena]
AT1G06980	4.12E-04	1.66	1.24	0.88	0.65	0.53	0.53	0.99	expressed protein similar to hypothetical protein GI:2347189 from [Arabidopsis thaliana]
AT1G11210	2.95E-03	1.40	1.38	0.74	0.62	0.53	0.45	0.84	expressed protein contains similarity to cotton fiber expressed protein 1 [Gossypium hirsutum] gi|3264828|gb|AAC33276
AT1G48330	1.46E-02	1.97	1.37	1.04	0.51	0.53	0.37	0.70	expressed protein similar to hypothetical protein GI:9294146 from [Arabidopsis thaliana]
AT2G30520	1.01E-02	1.29	1.30	0.69	0.82	0.53	0.63	1.19	signal transducer of phototropic response (RPT2) identical to RPT2 [Arabidopsis thaliana] gi|6959488|gb|AAF33112
AT1G23710	3.78E-02	1.27	1.44	0.67	0.64	0.53	0.45	0.85	expressed protein
AT4G25480	3.05E-02	1.63	1.37	0.86	0.78	0.53	0.57	1.08	DRE-binding protein (DREB1A) / CRT/DRE-binding factor 3 (CBF3) identical to DREB1A GI:3738224 from [Arabidopsis thaliana], DREB1A [Arabidopsis thaliana] GI:3660548, CRT/DRE binding factor 3 [Arabidopsis thaliana] GI:4091983; contains Pfam profile PF00847: AP2 domain; identical to cDNA CRT/DRE binding factor 3 (CBF3) GI:4322229
AT3G17790;AT3G	3.09E-03	1.16	1.21	0.61	0.67	0.53	0.56	1.05	[AT3G17790, acid phosphatase type 5 (ACP5) contains Pfam profile: PF00149 calcineurin-like phosphoesterase; nearly identical to acid phosphatase type 5 (GI:10278031) [Arabidopsis thaliana]];[AT3G17800, expressed protein]
AT3G56710	2.18E-02	1.56	1.53	0.82	0.67	0.53	0.44	0.83	sigA-binding protein identical to SigA binding protein [Arabidopsis thaliana] gi|6980074|gb|AAF34713; contains Pfam PF05678: VQ motif
AT1G69526	4.12E-03	1.35	1.27	0.71	0.82	0.53	0.65	1.22	UbiE/COQ5 methyltransferase family protein low similarity to SP|Q05197 Phosphatidylethanolamine N-methyltransferase (EC 2.1.1.17) [Rhodopseudomonas sphaeroides] (Rhodobacter sphaeroides); contains Pfam profile PF01209: methlytransferase, UbiE/COQ5 family
AT1G31650	8.12E-03	1.22	1.08	0.65	0.89	0.53	0.82	1.56	expressed protein contains Pfam profile PF03759: Domain of unknown function (DUF315)
AT1G78995	1.68E-03	1.36	1.27	0.72	0.53	0.53	0.42	0.79	expressed protein
AT1G69730;AT1G	2.62E-02	1.35	1.77	0.71	1.06	0.53	0.60	1.14	[AT1G69730, protein kinase family protein contains Pfam profile: PF00069 Eukaryotic protein kinase domain];[AT1G69720, heme oxygenase 3 (HO3) similar to heme oxygenase 3 [Arabidopsis thaliana] gi|14485563|gb|AAK63006]
AT3G47430	5.55E-03	1.48	1.48	0.78	0.57	0.53	0.38	0.73	peroxisomal biogenesis factor 11 family protein / PEX11 family protein contains Pfam PF05648: Peroxisomal biogenesis factor 11 (PEX11)
AT1G49010	6.85E-03	1.33	1.32	0.70	0.75	0.52	0.57	1.09	myb family transcription factor contains Pfam profile: PF00249 myb-like DNA-binding domain
AT1G80850	2.39E-03	1.39	1.46	0.73	0.75	0.52	0.51	0.98	methyladenine glycosylase family protein similar to SP|P05100 DNA-3-methyladenine glycosylase I (EC 3.2.2.20) (3-methyladenine-DNA glycosylase I, constitutive) (Escherichia coli); contains Pfam profile PF03352: Methyladenine glycosylase
AT2G29650	3.77E-03	1.27	1.37	0.67	0.79	0.52	0.58	1.10	inorganic phosphate transporter, putative similar to brain specific Na+-dependent inorganic phosphate cotransporter [Rattus norvegicus] GI:507415; contains Pfam profile PF00083: major facilitator superfamily protein
AT4G26520;AT4G	9.81E-04	1.43	1.24	0.75	0.69	0.52	0.56	1.06	[AT4G26520, fructose-bisphosphate aldolase, cytoplasmic identical to SP|P22197 Fructose-bisphosphate aldolase, cytoplasmic isozyme (EC 4.1.2.13) (Arabidopsis thaliana)];[AT4G26530, fructose-bisphosphate aldolase, putative strong similarity to SP|P22197 Fructose-bisphosphate aldolase, cytoplasmic isozyme (EC 4.1.2.13) (Arabidopsis thaliana)]
AT5G02760	1.73E-03	1.60	1.46	0.84	0.70	0.52	0.48	0.92	protein phosphatase 2C family protein / PP2C family protein similar to Ser/Thr protein phosphatase 2C (PP2C6) (GI:15020818) [Arabidopsis thaliana]; similar to protein phosphatase 2C (GI:3608412) [Mesembryanthemum crystallinum]; contains Pfam PF00481 : Protein phosphatase 2C domain
AT1G69160	3.95E-03	1.54	1.45	0.80	0.84	0.52	0.58	1.11	expressed protein
AT1G76360	7.78E-05	1.41	1.43	0.73	0.71	0.52	0.50	0.96	protein kinase, putative similar to protein kinase APK1B, SWISS-PROT:P46573; contains protein kinase domain, Pfam:PF00069
AT1G10470	2.93E-05	1.41	1.39	0.73	0.56	0.52	0.40	0.78	two-component responsive regulator / response regulator 4 (ARR4) identical to responce regulator1 GI:3273195 from [Arabidopsis thaliana]; identical to cDNA ARR4 mRNA for response regulator 4 GI:3953596
AT4G32980	7.87E-03	1.52	1.21	0.79	0.75	0.52	0.62	1.21	homeobox protein (ATH1) identical to SWISS-PROT:P48731 homeobox protein ATH1. [Arabidopsis thaliana]
AT5G42250	5.99E-04	1.70	1.07	0.88	0.50	0.52	0.47	0.90	alcohol dehydrogenase, putative similar to alcohol dehydrogenase ADH GI:7705214 from [Lycopersicon esculentum]; contains Pfam zinc-binding dehydrogenase domain PF00107
AT1G64900	1.69E-02	1.79	1.31	0.92	0.74	0.52	0.56	1.09	cytochrome P450, putative similar to cytochrome p450 GI:438240 from [Solanum melongena]
AT1G09240	3.24E-02	2.09	0.95	1.08	0.56	0.52	0.59	1.14	nicotianamine synthase, putative similar to nicotianamine synthase [Lycopersicon esculentum][GI:4753801], nicotianamine synthase 2 [Hordeum vulgare][GI:4894912]
AT5G62570	1.41E-02	1.27	1.40	0.66	0.92	0.52	0.65	1.27	calmodulin-binding protein similar to calmodulin-binding protein TCB60 GI:1698548 from [Nicotiana tabacum]
AT3G54500	9.48E-03	1.34	1.37	0.69	0.93	0.52	0.68	1.31	expressed protein
AT4G26520;AT4G	4.97E-04	1.35	1.38	0.70	0.72	0.52	0.52	1.01	[AT4G26520, fructose-bisphosphate aldolase, cytoplasmic identical to SP|P22197 Fructose-bisphosphate aldolase, cytoplasmic isozyme (EC 4.1.2.13) (Arabidopsis thaliana)];[AT4G26530, fructose-bisphosphate aldolase, putative strong similarity to SP|P22197 Fructose-bisphosphate aldolase, cytoplasmic isozyme (EC 4.1.2.13) (Arabidopsis thaliana)]
AT3G55630	1.03E-03	1.33	1.36	0.69	0.71	0.51	0.52	1.01	dihydrofolate synthetase/folylpolyglutamate synthetase (DHFS/FPGS4) nearly identical to folylpolyglutamate-dihydrofolate synthetase [Arabidopsis thaliana] GI:17976761
AT1G29450	4.04E-03	1.44	1.43	0.74	0.62	0.51	0.43	0.84	auxin-responsive protein, putative similar to auxin-induced protein 6B (SP:P33083) [Glycine max]
AT2G45560	6.09E-03	0.91	1.64	0.47	1.00	0.51	0.61	1.19	cytochrome P450 family protein
AT1G49840	1.00E-03	1.53	1.54	0.78	0.72	0.51	0.47	0.92	expressed protein contains Pfam profile PF04788: Protein of unknown function (DUF620)
AT1G34040	1.34E-04	0.98	1.80	0.50	0.94	0.51	0.52	1.03	alliinase family protein contains Pfam profiles: PF04864 alliinase C-terminal domain, PF04863 alliinase EGF-like domain
AT2G39650	4.05E-02	1.19	2.29	0.61	1.02	0.51	0.45	0.88	expressed protein contains Pfam profile PF04720: Protein of unknown function (DUF506)
AT3G15570	8.01E-04	1.41	1.38	0.72	0.69	0.51	0.50	0.99	phototropic-responsive NPH3 family protein contains NPH3 family domain, Pfam:PF03000
AT4G30410	3.15E-03	1.81	1.16	0.91	0.53	0.51	0.45	0.90	expressed protein similar to cDNA bHLH transcription factor (bHLH eta gene) gi:32563007
AT5G01790	2.89E-02	1.72	1.34	0.87	0.71	0.51	0.53	1.05	expressed protein
AT2G41250	4.47E-04	0.99	2.28	0.50	0.94	0.50	0.41	0.82	haloacid dehalogenase-like hydrolase family protein low similarity to SP|Q94915 Rhythmically expressed gene 2 protein (DREG-2) [Drosophila melanogaster]; contains InterPro accession IPR005834: Haloacid dehalogenase-like hydrolase
AT5G16990;AT5G	1.91E-02	2.16	0.89	1.09	0.55	0.50	0.61	1.22	[AT5G16990, NADP-dependent oxidoreductase, putative strong similarity to probable NADP-dependent oxidoreductase (zeta-crystallin homolog) P1 [SP|Q39172][gi:886428] and P2 [SP|Q39173][gi:886430], Arabidopsis thaliana];[AT5G17000, NADP-dependent oxidoreductase, putative strong similarity to probable NADP-dependent oxidoreductase (zeta-crystallin homolog) P1 [SP|Q39172][gi:886428] and P2 [SP|Q39173][gi:886430], Arabidopsis thaliana];[AT5G16980, NADP-dependent oxidoreductase, putative strong similarity to probable NADP-dependent oxidoreductase (zeta-crystallin homolog) P1 [SP|Q39172][gi:886428] and P2 [SP|Q39173][gi:886430], Arabidopsis thaliana];[AT5G16970, NADP-dependent oxidoreductase, putative (P1) identical to probable NADP-dependent oxidoreductase P1, zeta-crystallin homolog [SP|Q39172][gi:886428], Arabidopsis thaliana; similar to allyl alcohol dehydrogenase [Nicotiana tabacum] GI:6692816; contains Pfam profile PF00107: oxidoreductase, zinc-binding dehydrogenase family]
AT5G01540	2.23E-02	1.04	1.68	0.52	1.00	0.50	0.60	1.18	lectin protein kinase, putative similar to receptor lectin kinase 3 [Arabidopsis thaliana] gi|4100060|gb|AAD00733; contains protein kinase domain, Pfam:PF00069; contains legume lectins alpha and beta domains, Pfam:PF00138 and Pfam:PF00139
AT5G57780	9.79E-03	1.51	1.58	0.76	0.70	0.50	0.45	0.89	expressed protein similar to unknown protein (emb CAB79759.1)
AT2G46400	2.19E-02	1.29	2.33	0.65	0.74	0.50	0.32	0.63	WRKY family transcription factor
AT3G48200	1.18E-02	1.20	1.26	0.60	0.94	0.50	0.74	1.48	expressed protein
AT5G44190	2.97E-02	1.53	1.47	0.77	0.96	0.50	0.65	1.29	myb family transcription factor (GLK2) contains Pfam profile: PF00249 myb-like DNA-binding domain
AT5G41050	2.74E-03	1.61	1.51	0.80	0.73	0.50	0.49	0.98	expressed protein
AT5G02180	2.13E-04	1.32	1.31	0.66	0.67	0.50	0.51	1.02	amino acid transporter family protein belongs to INTERPRO:IPR002422 amino acid/polyamine transporter, family II
AT1G51700	1.83E-02	1.29	1.90	0.64	0.74	0.50	0.39	0.79	Dof-type zinc finger domain-containing protein (ADOF1) identical to cDNA adof1 mRNA for dof zinc finger protein, GI:3608260; contains Pfam profile PF02701: Dof domain, zinc finger
AT1G75100	4.55E-04	1.23	1.68	0.61	0.94	0.50	0.56	1.13	expressed protein low similarity to SP|O14976 Cyclin G-associated kinase (EC 2.7.1.-) (Homo sapiens)
AT5G46240	1.37E-02	1.41	1.25	0.70	0.78	0.50	0.63	1.26	inward rectifying potassium channel (KAT1) identical to K+ channel [Arabidopsis thaliana] gi|1165000|emb|CAA63601; Shaker-type channel (1P/6TM), PMID:11500563
AT5G56850	2.28E-03	1.21	1.64	0.60	0.85	0.49	0.51	1.04	expressed protein
AT1G66130	3.35E-03	1.59	1.55	0.79	0.72	0.49	0.46	0.94	oxidoreductase N-terminal domain-containing protein similar to AX110P [Daucus carota] GI:285739; contains Pfam profile PF01408: Oxidoreductase family NAD-binding Rossmann fold
AT3G17330	3.65E-04	1.39	1.38	0.68	0.72	0.49	0.52	1.06	expressed protein contains Pfam profile PF04146: YTS21-B-like family
AT2G37970	2.58E-04	1.28	2.62	0.63	0.87	0.49	0.33	0.67	SOUL heme-binding family protein weak similarity to SOUL protein [Mus musculus] GI:4886906; contains Pfam profile PF04832: SOUL heme-binding protein
AT1G70560	1.73E-03	1.46	1.52	0.72	0.87	0.49	0.58	1.17	alliinase C-terminal domain-containing protein contains Pfam profiles: PF04864 alliinase C-terminal domain, PF04863 alliinase EGF-like domain
AT5G03720	2.93E-05	1.58	1.18	0.78	0.58	0.49	0.49	0.99	heat shock transcription factor family protein contains Pfam profile: PF00447 HSF-type DNA-binding domain
AT2G30070	1.17E-03	1.31	1.15	0.64	0.78	0.49	0.68	1.38	potassium transporter (KUP1) identical to potassium transporter [Arabidopsis thaliana] gi|2654088|gb|AAB87687; KUP/HAK/KT Transporter family member, PMID:11500563
AT5G46470	2.45E-02	1.25	1.34	0.61	0.97	0.49	0.72	1.48	disease resistance protein (TIR-NBS-LRR class), putative domain signature TIR-NBS-LRR exists, suggestive of a disease resistance protein.
AT3G52720	2.19E-03	1.80	1.23	0.88	0.61	0.49	0.50	1.02	carbonic anhydrase family protein low similarity to storage protein (dioscorin) [Dioscorea cayenensis] GI:433463; contains Pfam profile PF00194: Eukaryotic-type carbonic anhydrase
AT5G24120	3.15E-03	1.49	1.74	0.73	0.82	0.49	0.47	0.97	RNA polymerase sigma subunit SigE (sigE) / sigma-like factor (SIG5) identical to RNA polymerase sigma subunit SigE [Arabidopsis thaliana] GI:4972299, sigma-like factor [Arabidopsis thaliana] GI:4033838; contains Pfam profiles PF04545: Sigma-70, region 4, PF04539: Sigma-70 region 3, PF04542: Sigma-70 region 2
AT4G27820	3.82E-03	1.63	1.41	0.80	0.83	0.49	0.59	1.21	glycosyl hydrolase family 1 protein contains Pfam PF00232 : Glycosyl hydrolase family 1 domain; TIGRFAM TIGR01233: 6-phospho-beta-galactosidase; similar to hydroxyisourate hydrolase (GI:19569603) [Glycine max]
AT5G45800	2.39E-03	1.12	1.18	0.55	0.84	0.49	0.71	1.45	leucine-rich repeat transmembrane protein kinase, putative
AT5G52760	1.17E-03	1.35	1.48	0.66	0.44	0.49	0.30	0.61	heavy-metal-associated domain-containing protein contains Pfam profile PF00403: Heavy-metal-associated domain
AT4G33666;AT4G	4.29E-02	1.41	1.18	0.68	0.68	0.48	0.58	1.20	[AT4G33666, expressed protein];[AT4G33670, L-galactose dehydrogenase (L-GalDH) identical to L-galactose dehydrogenase [Arabidopsis thaliana] GI:16555790; similar to L-fucose dehydrogenase [Pseudomonas sp.] GI:829054; contains Pfam profile PF00248: oxidoreductase, aldo/keto reductase family]
AT3G47420	2.11E-02	0.99	1.59	0.48	1.09	0.48	0.68	1.42	glycerol-3-phosphate transporter, putative / glycerol 3-phosphate permease, putative similar to cAMP inducible 2 protein [Mus musculus] GI:4580997, glycerol-3-phosphate transporter (glycerol 3-phosphate permease) [Homo sapiens] GI:7543982; contains Pfam profile PF00083: major facilitator superfamily protein
AT5G02540	1.34E-04	1.80	1.36	0.87	0.54	0.48	0.40	0.83	short-chain dehydrogenase/reductase (SDR) family protein contains INTERPRO family IPR002198 Short-chain dehydrogenase/reductase (SDR) superfamily
AT5G03230	1.46E-03	1.46	1.50	0.70	0.65	0.48	0.43	0.89	expressed protein contains Pfam profile PF04520: Protein of unknown function, DUF584
AT1G22640	2.17E-04	1.57	1.40	0.76	0.60	0.48	0.43	0.90	myb family transcription factor (MYB4) similar to myb-related protein GI:1020155 from [Arabidopsis thaliana]
AT1G01720	3.59E-03	1.74	1.31	0.84	0.76	0.48	0.58	1.20	no apical meristem (NAM) family protein contains Pfam PF02365: No apical meristem (NAM) domain; similar to NAC domain protein NAM GB:AAD17313 GI:4325282 from [Arabidopsis thaliana]
AT3G44630	3.80E-02	1.30	1.50	0.63	1.06	0.48	0.71	1.46	disease resistance protein RPP1-WsB-like (TIR-NBS-LRR class), putative domain signature TIR-NBS-LRR exists, suggestive of a disease resistance protein., closest Col-0 homolog to RPP1-WsB
AT4G34630	3.19E-02	1.48	1.29	0.71	0.83	0.48	0.64	1.34	expressed protein
AT1G72030	3.03E-03	1.30	1.78	0.62	0.81	0.48	0.45	0.94	GCN5-related N-acetyltransferase (GNAT) family protein contains Pfam profile PF00583: acetyltransferase, GNAT family
AT1G31920	6.01E-03	1.24	1.44	0.59	0.76	0.48	0.52	1.10	pentatricopeptide (PPR) repeat-containing protein contains Pfam profile PF01535: PPR repeat
AT1G13080	2.98E-03	1.50	1.64	0.71	0.75	0.48	0.46	0.96	cytochrome P450 family protein identical to gb|D78605 cytochrome P450 monooxygenase from Arabidopsis thaliana and is a member of the PF|00067 Cytochrome P450 family. ESTs gb|Z18072, gb|Z35218 and gb|T43466 come from this gene
AT5G35970	3.00E-02	1.39	1.33	0.66	0.99	0.48	0.74	1.55	DNA-binding protein, putative similar to SWISS-PROT:Q60560 DNA-binding protein SMUBP-2 (immunoglobulin MU binding protein 2, SMUBP-2) [Mesocricetus auratus]
AT1G14345	2.88E-03	1.56	1.23	0.74	0.50	0.48	0.41	0.86	expressed protein contains one transmembrane domain
AT2G03540	1.53E-03	1.71	1.35	0.81	0.75	0.47	0.55	1.17	expressed protein ; expression supported by MPSS
AT4G16780	1.51E-04	1.59	1.47	0.75	0.66	0.47	0.45	0.94	homeobox-leucine zipper protein 4 (HAT4) / HD-ZIP protein 4 SP|Q05466|HAT4_ARATH Homeobox-leucine zipper protein HAT4 (HD-ZIP protein 4) (SP:Q05466) [Arabidopsis thaliana] (HD-ZIP homeotic protein Athb-2
AT3G50060	2.32E-02	1.91	1.37	0.90	0.74	0.47	0.54	1.13	myb family transcription factor contains Pfam profile: PF00249 myb-like DNA-binding domain; identical to cDNA MYB-related protein (1107 bp) GI:1263096
AT3G48100	1.77E-02	2.06	1.38	0.97	0.44	0.47	0.32	0.67	two-component responsive regulator / response regulator 5 (ARR5) / response reactor 2 (RR2) identical to responce reactor2 (AtRR2) [Arabidopsis thaliana] GI:3273198, response regulator 5 (ARR5) [Arabidopsis thaliana] GI:3953599; contains Pfam profile: PF00072 response regulator receiver domain
AT1G78070	2.17E-04	1.68	1.65	0.79	0.69	0.47	0.42	0.89	WD-40 repeat family protein contains Pfam profile PF00400: WD domain, G-beta repeat
AT4G01460	1.39E-04	1.63	1.80	0.77	0.75	0.47	0.42	0.89	basic helix-loop-helix (bHLH) family protein contains Pfam profile: PF00010 helix-loop-helix DNA-binding domain
AT1G19050	2.63E-02	1.82	1.93	0.85	0.67	0.47	0.35	0.74	two-component responsive regulator / response regulator 7 (ARR7) identical to response regulator 7 GI:3953603 from [Arabidopsis thaliana]
AT1G27480	4.34E-04	1.70	1.36	0.80	0.67	0.47	0.50	1.06	lecithin:cholesterol acyltransferase family protein / LACT family protein similar to LCAT-like lysophospholipase (LLPL) [Homo sapiens] GI:4589720; contains Pfam profile PF02450: Lecithin:cholesterol acyltransferase (phosphatidylcholine-sterol acyltransferase)
AT3G11090	1.10E-02	1.59	1.45	0.74	0.60	0.47	0.41	0.88	LOB domain family protein / lateral organ boundaries domain family protein (LBD21) identical to SP|Q9SRL8 Putative LOB domain protein 21 [Arabidopsis thaliana]; similar to lateral organ boundaries (LOB) domain-containing proteins from Arabidopsis thaliana
AT2G31380	8.74E-03	1.66	1.70	0.77	0.75	0.46	0.44	0.95	zinc finger (B-box type) family protein / salt tolerance-like protein (STH) contains Pfam profile PF00643: B-box zinc finger; identical to cDNA B-box zinc finger protein STH GI:12698721, SP|Q9SID1 Salt tolerance-like protein (Arabidopsis thaliana)
AT2G21320	4.42E-03	1.38	1.93	0.64	0.72	0.46	0.37	0.80	zinc finger (B-box type) family protein
AT5G16030	1.15E-04	1.32	1.44	0.61	0.71	0.46	0.49	1.07	expressed protein
AT4G23290	4.31E-04	1.34	1.88	0.62	0.66	0.46	0.35	0.77	protein kinase family protein contains Pfam domain PF00069: Protein kinase domain
AT1G29430	5.42E-03	1.61	1.49	0.74	0.41	0.46	0.28	0.60	auxin-responsive family protein similar to auxin-induced protein 10a 6B (SP:P33083) [Glycine max]
AT1G20190	2.82E-04	1.76	1.28	0.81	0.48	0.46	0.37	0.82	expansin, putative (EXP11) similar to GB:U30460 from [Cucumis sativus]; alpha-expansin gene family, PMID:11641069
AT2G20570	1.37E-03	1.26	1.52	0.58	0.80	0.46	0.52	1.15	golden2-like transcription factor (GLK1) identical to golden2-like transcription factor GI:13311003 from [Arabidopsis thaliana]; contains Pfam profile: PF00249 Myb-like DNA-binding domain
AT3G49670	6.81E-03	1.42	1.16	0.65	0.81	0.45	0.70	1.54	leucine-rich repeat transmembrane protein kinase, putative CLAVATA1 receptor kinase, Arabidopsis thaliana, EMBL:ATU96879
AT1G64780	2.31E-03	1.88	1.55	0.85	0.78	0.45	0.50	1.11	ammonium transporter 1, member 2 (AMT1.2) nearly identical to SP|Q9ZPJ8 Ammonium transporter 1, member 2 (AtAMT1;2) (Arabidopsis thaliana)
AT4G37370	1.95E-02	1.05	1.55	0.47	1.08	0.45	0.70	1.55	cytochrome P450, putative similar to Cytochrome P450 91A1 (SP:Q9FG65)[Arabidopsis thaliana]; cytochrome P450, Glycyrrhiza echinata, AB001379
